# PathoFact 2.0: an integrative pipeline for the prediction of antimicrobial resistance genes, virulence factors, toxins and toxin-associated proteins, and biosynthetic gene clusters in metagenomes

**DOI:** 10.1093/gigascience/giag062

**Published:** 2026-05-22

**Authors:** Luis F Delgado, Júlia Ortís Sunyer, Cedric C Laczny, Oskar Hickl, Patrick May, Paul Wilmes

**Affiliations:** Luxembourg Centre for Systems Biomedicine, University of Luxembourg, Esch-sur-Alzette, Luxembourg; Luxembourg Centre for Systems Biomedicine, University of Luxembourg, Esch-sur-Alzette, Luxembourg; Luxembourg Centre for Systems Biomedicine, University of Luxembourg, Esch-sur-Alzette, Luxembourg; Luxembourg Centre for Systems Biomedicine, University of Luxembourg, Esch-sur-Alzette, Luxembourg; Luxembourg Centre for Systems Biomedicine, University of Luxembourg, Esch-sur-Alzette, Luxembourg; Luxembourg Centre for Systems Biomedicine, University of Luxembourg, Esch-sur-Alzette, Luxembourg; Department of Health, Medicine and Life Sciences, Faculty of Science, Technology and Medicine, University of Luxembourg, Esch-sur-Alzette, Luxembourg

**Keywords:** antimicrobial resistance genes, virulence factors, toxin-associated proteins, biosynthetic gene clusters, metagenomes, machine learning

## Abstract

**Background:**

Antimicrobial resistance genes (ARGs) and virulence factors (VFs) are central contributors to the global health crisis surrounding drug-resistant infections.

**Findings:**

We introduce PathoFact 2.0, an enhanced pipeline for improved ARG, VF, toxin, and biosynthetic gene clusters (BGCs) prediction. Key improvements include an updated machine learning (ML) model for VF identification, expanded hidden Markov model profiles for VFs and toxin-associated proteins, a new ML model for toxin and toxin-associated proteins identification, and the integration of antiSMASH 7.0 for predicting BGCs.

**Conclusions:**

Our upgrades make PathoFact 2.0 a more powerful and user-friendly platform for predicting microbiome-based pathogenicity and resistance, providing a crucial tool for better understanding and addressing the challenges posed by antimicrobial resistance and infectious diseases.

PathoFact 2.0 is available at https://gitlab.com/uniluxembourg/lcsb/systems-ecology/pathofact2. It is compatible with Linux operating systems.

## Introduction

Microbiomes are highly complex and diverse ecological communities composed of bacteria, archaea, viruses, and microeukaryotes. These communities include both commensal microorganisms, which can contribute to host health, and pathogenic or opportunistic microorganisms that can cause disease under specific conditions. Microbial communities generally exist in synergistic relationships with their hosts, playing critical roles in maintaining physiological homeostasis and regulating immune function. However, disruption of this balanced microbial ecosystem, known as microbial dysbiosis, can impair normal body functions and has been associated with the development of various diseases, including cardiovascular diseases, cancers, and respiratory disorders [1].

Moreover, these microorganisms play a critical role in the development of antibiotic-resistant infections through the presence of antimicrobial resistance genes (ARGs) and virulence factors (VFs) [2, 3]. ARGs are genetic elements that confer bacterial resistance to antibiotics. Many ARGs are encoded on mobile genetic elements (MGEs) and are therefore often horizontally transmitted [4]. ARGs can be divided into categories based on the antibiotics to which they confer resistance [5]. The Antibiotic Resistance Ontology (ARO) contains information on ARGs, the mutations that cause them, their products, mechanisms, associated phenotypes, antibiotics, and molecular targets [6].

Bacterial pathogens use specific genes, known as VFs, to attach to and invade host tissues, survive within the host, spread, and ultimately cause damage. The harm inflicted can vary, ranging from minor disruptions to severe or even fatal outcomes [7]. VFs can be classified as secretory, membrane-associated, or cytosolic. Cytosolic VFs promote rapid adaptive shifts in bacterial metabolism, physiology, and morphology, enhancing survival and proliferation within the host. Membrane-associated factors contribute to bacterial adhesion and immune evasion at the host–cell interface. Secretory factors constitute a critical part of the bacterial armamentarium, enabling bacteria to counteract innate and adaptive immune defences. Secretory VFs often exhibit synergistic effects and induce cytotoxicity in host cells [8]. VFs are often located on MGEs, such as transposons, plasmids, and phages, facilitating their transfer between bacterial cells [9, 10].

Bacterial toxins play a crucial role in the development of infectious diseases, alongside various VFs employed by pathogens. They disrupt host processes and manipulate immune responses. Some toxins impair protein synthesis, destroy blood cells, or affect the nervous system. Bacterial toxins can be divided into 2 main categories: cell-associated endotoxins and extracellular, diffusible exotoxins. Endotoxins, such as lipopolysaccharides, are found in the outer membranes of Gram-negative bacteria and serve as potent inflammatory mediators that can induce systemic toxicity and septic shock in infected hosts [11]. Exotoxins, which are typically polypeptides and proteins, can stimulate a range of host responses by either acting directly on cell receptors or through enzymatic modulation [12, 13]. Many bacterial toxins are secreted proteins that require signal peptides. Signal peptides are short amino acid sequences at the N-terminus of proteins that direct them to specific cellular compartments, such as the periplasm [14, 15].

Biosynthetic gene clusters (BGCs) are responsible for synthesizing specialized metabolites (SMs). Some SMs can increase pathogenicity; for example, clinical isolates of *Pseudomonas aeruginosa* produce siderophores, rhamnolipids, quinolones, and phenazines [16]. Similarly, *Burkholderia* strains produce VFs, such as toxoflavin from *Burkholderia glumae* [17]. Notably, pyocyanin, a redox-active phenazine produced by *Pseudomonas aeruginosa*, plays a crucial role as a VF in lung infections [18].

The threat that ARGs, VFs, and toxins pose to human health is significant. The United Nations has identified antimicrobial resistance as a global threat, with estimates attributing 1.27 million deaths annually to drug-resistant infections, potentially rising to 10 million by 2050 if unaddressed [19, 20]. Thus, accurately predicting potential ARG and VF profiles is essential for early intervention, enabling anticipation of infection severity, improving treatment strategies, and ultimately reducing mortality rates from disease-causing pathogens.

Predicting and annotating ARGs, VFs, and toxins is challenging due to limited well-annotated data [21] and complex mechanisms involving gene transfer, mutations, and multifactorial interactions. Traditional annotation methods, which rely on sequence similarity, may overlook novel ARGs, VFs, and toxins. In contrast, machine learning (ML) offers robust solutions through pattern recognition, enabling accurate predictions even with limited training data.

An integrated bioinformatics pipeline enhances analysis by simultaneously examining ARGs, VFs, toxins, signal peptides, and BGCs from a single metagenomic sample. This comprehensive approach provides a more complete view of bacterial pathogenicity by capturing the full spectrum of virulence mechanisms, including antimicrobial resistance, toxin production, and secondary metabolic capabilities. This holistic analysis improves insights into pathogenicity and resistance, streamlines workflows, and simplifies data interpretation.

PathoFact 1.0, a pipeline first introduced in 2020, integrates ARG, VF, and bacterial toxin prediction from metagenomic data into a single tool [22]. Since the publication of PathoFact, several tools have been implemented to predict ARGs, VFs, and bacterial toxins [23–25]. HyperVR [24] has attempted to predict them simultaneously, analogous to PathoFact. However, HyperVR’s repository is no longer available online, and the Zenodo archive from its original submission lacks the necessary databases, hence rendering it unusable. gSpreadComp [26] recently proposed a workflow that integrates comparative genomics, plasmid-mediated transfer assessment, and resistance-virulence risk-ranking to facilitate hypothesis generation for targeted experimental validation by identifying concerning resistant hotspots in complex microbial datasets. However, while gSpreadComp is valuable for comparative, community-level prioritization, ARGs are predicted using only one tool, DeepARG, and VF are annotated using databases and alignment-based approaches rather than predictive models and ML frameworks. In addition, it lacks a signal peptide or a BGC annotation framework needed for comprehensive microbiome-based pathogenicity profiling, and the pipeline accepts only DNA sequences as input.

**Figure 1:**
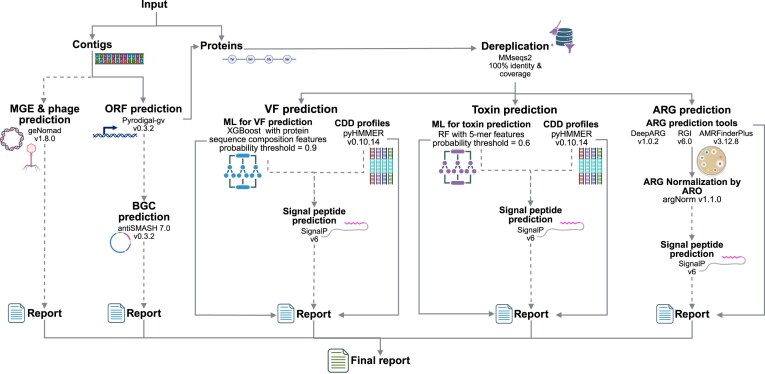
Schematic representation of PathoFact 2.0. Solid lines denote core modules, while dotted lines indicate optional user selection. The input is a FASTA file with either contig or protein sequences. If the input is a FASTA file containing contigs, open reading frames (ORFs) are predicted using Pyrodigal-gv. If the BGC option is selected, antiSMASH will use the GBK file for BGC prediction. GeNomad is used for MGE and phage prediction, producing a FASTA file of protein sequences. Protein sequences are dereplicated using MMseqs2 to retain non-redundant sequences (based on 100% identity and coverage). After dereplication, antimicrobial resistance genes (ARGs), VFs, and toxins and their associated proteins are predicted using their respective modules. SignalP predicts the presence of signal peptides and their cleavage sites in proteins from archaea, bacteria, and eukarya. Individual reports are generated for each module, and an integrated report is produced that combines all module reports. Created in BioRender. Ortís Sunyer, J. (2026) https://BioRender.com/9ztc4ki.

Here, we present PathoFact 2.0 (Fig. 1). It enhances the previous version by supporting protein sequences or contigs as input and by updating the ML VF model and the hidden Markov model (HMM) profiles of the conserved domain databases (CDD) [27] for VF and toxin-associated protein annotation. We have also introduced the ability to predict BGCs using antiSMASH 7.0 [28]. antiSMASH is a tool that identifies, annotates, and analyses secondary metabolite BGCs across genomes.

## Pipeline structure

Unlike version 1.0, which supports only contigs, PathoFact 2.0 accepts nucleotide sequences of contigs and protein sequence FASTA files, with proteins dereplicated by our tool to retain only non-redundant sequences (based on 100% identity and coverage). For contig-based inputs, open reading frames are predicted using Pyrodigal-gv (version 0.3.2; [29, 30]; https://github.com/althonos/pyrodigal-gv), a Python library that binds to Prodigal [22], followed by the detection of MGEs and phages using geNomad (version 1.8.0; [30]). GeNomad processes only nucleotide sequences; therefore, MGEs and phages are not detected in protein sequence inputs. Based on user configuration, the pipeline then analyses the processed sequences using the ARG, VF, toxin-associated, and BGC (via antiSMASH) prediction modules. The information is compiled into individual module reports and an integrated report, also incorporating details from SignalP and geNomad (Fig. 1). Additionally, PathoFact 2.0 generates a FASTA file of proteins identified as ARGs, VFs, or toxin-associated proteins.

## Pipeline installation

PathoFact 2.0 is implemented using Snakemake (version 7.25.0; [31]). An installation script simplifies the setup by installing the required software and downloading databases with a single command. PathoFact 2.0 is open-source (GNU General License v3.0 or later) and freely available at https://gitlab.com/uniluxembourg/lcsb/systems-ecology/pathofact2, where detailed instructions for pipeline installation, configuration, and output are provided.

## Pipeline updates

**Figure 2:**
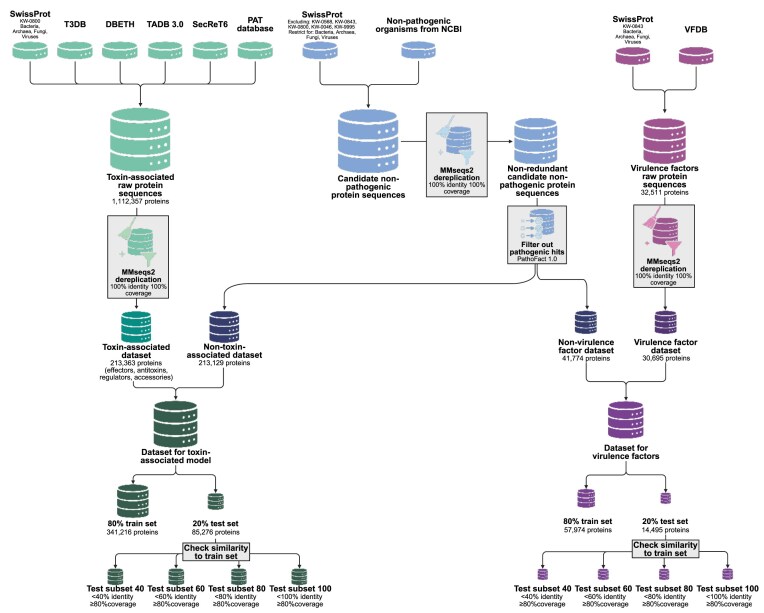
Schematic representation of the datasets used for PathoFact 2.0 toxin-associated and VFs modules training and testing. Created in BioRender. Ortís Sunyer, J. (2026) https://BioRender.com/x9fn4zn.

We implemented thorough updates across all PathoFact modules. Notably, we developed two new ML models: one to predict VFs and another to identify toxin-associated proteins. A schematic diagram of the construction of the training and test datasets is shown in Fig. 2. In the sections below, we detail the updates to each module.

### Generalities about the “non-pathogenic” dataset and the ML training setup

The “non-pathogenic” dataset for the ML models was constructed by selecting SwissProt [32] sequences lacking ARG, VF, and toxin keywords [KW-0568 (pathogenesis-related protein), KW-0843 (virulence), KW-0800 (toxin), KW-0046 (antibiotic resistance), KW-9995 (disease)] and limited to bacteria (taxonomy_id 2), archaea (taxonomy_id 2157), fungi (taxonomy_id 4751), and viruses (taxonomy_id 10239) (Fig. 2). Additionally, proteins from non-pathogenic organisms to humans (Supplementary Table S1; Fig. 2) were included from NCBI. MMseqs2 (version 15.6f452; [33]) was used to obtain a set of non-redundant (clustered at 100% identity and coverage) protein sequences (Fig. 2).

ML models were trained using 80% of the sequences for training and 20% of the sequences for testing. The synthetic minority oversampling technique (SMOTE) was employed to address the dataset’s imbalance [34]. Using the XGBoost Python package (https://xgboost.readthedocs.io/en/stable/index.html) and the Random Forest (RF) scikit-learn (version 1.5.2; [35]), several ML models were trained and tested using *k*-mers (*k* = 3–8) or protein sequence composition features (amino acid composition [AAC], dipeptide composition [DPC], composition [CTDC], transition [CTDT], and distribution [CTDD] [36]) as features. Hyperparameter optimization was performed, using a 5-fold cross-validation with HalvingGridSearchCV from scikit-learn [35]. The best-performing model was selected based on the Matthews correlation coefficient (MCC) score.

### Toxin-associated protein prediction updates

Compared to version 1.0, the toxin prediction module now employs an ML model instead of a purely alignment-based bit score threshold. Curated training data was obtained from SwissProt [32], filtered for bacterial (taxonomy_id 2), archaeal (taxonomy_id 2157), fungal (taxonomy_id 4751), and viral (taxonomy_id 10239) toxin sequences (KW-0800, toxin) (Fig. 2). The dataset was supplemented with entries from toxin-specific databases such as the Toxin Exposome Database (T3DB) [12], which catalogues bacterial protein toxins; the Database for Bacterial ExoToxins (DBETH) [13]; TADB version 3.0, which includes protein sequences of bacterial toxin–antitoxin (TA) pairs from types I to VIII [37]; sequences from SecReT6 [38], encompassing T6SS gene cluster components, T6SS regulator (T6SR), accessory proteins (T6SA), effectors (T6SE), and immunity proteins (T6SI); and the prokaryotic antimicrobial toxins (PAT) database [39] (Fig. 2).

MMseqs2 (version 15.6f452; [33]) was used to dereplicate the dataset of 1,112,357 protein sequences (100% identity and coverage), yielding 213,363 unique protein sequences, corresponding to the “toxin-associated” dataset (Fig. 2). It is essential to note that this dataset encompasses both effector toxin proteins and their associated proteins, including antitoxins, regulators, and accessory proteins. This offers three main benefits: (1) recent reports suggest that the same bacterial toxins can function as part of self-inhibiting toxin–antitoxin modules within one organism, while in another organism, they have evolved into toxin effectors that are injected into target cells [40, 41]. (2) In bacteria, genes located in close proximity frequently exhibit functional associations, such as those co-transcribed within operons. A comprehensive toxin dataset, including both toxins and their associated proteins, facilitates the identification of novel toxins and related genes through their genomic context, referred to as “toxin islands.” These islands may be involved in toxin biosynthesis, processing, or secretion, and may also confer immunity or facilitate horizontal gene transfer among bacterial populations. Notably, they are often rich in MGEs [42]. (3) A large database improves the performance of ML classification methods [43].

HMM profiles were built using the conserved-domain FASTA files (https://ftp.ncbi.nih.gov/pub/mmdb/cdd/fasta.tar.gz) from CDD [27]. The 213,363 unique protein sequences in the “toxin-associated” dataset were annotated using the CDD HMM profiles. Those with a bitscore above 25 were chosen as HMM profiles for toxin and toxin-associated protein annotation and incorporated into PathoFact 2.0 for protein annotation.

Although there is no standard for creating negative datasets, they play a crucial role in influencing model performance. Therefore, to improve the quality of our “non-toxin” dataset, potential ARGs, VFs (with high probability), and toxins were filtered out of the “non-pathogenic” dataset using PathoFact 1.0 predictions. The final “non-toxin” dataset consists of 213,129 non-redundant protein sequences (Fig. 2).

The toxin-associated ML model is an RF with 5-mer features (default hyperparameter setting, i.e, number of trees in the forest [*n*_estimators] = 100, maximum depth of the tree [max_depth] = None, minimum number of samples required to split an internal node [min_samples_split] = 2).

The toxin-associated protein prediction module generates a report containing the protein ID, protein domains, bitscore, toxin-associated ML probability, other identical proteins found in the sample, and optionally SignalP, plasmid marker, and virus marker information.

### VF prediction updates

The VF prediction model was refined and updated with new HMM profiles. Training data were derived from SwissProt [32], selecting sequences annotated with the virulence keyword (KW-0843) and expanded using the Virulence Factor Database (VFDB; [44]) (Fig. 2). After dereplication (with 100% identity and coverage), the original set of 32,511 sequences, using MMseqs2, comprised 30,695 non-redundant sequences, corresponding to the “VF dataset” (Fig. 2). We searched the “VF dataset” against the CDD HMM profiles, selecting those with a bit score of 25 or higher as VF HMM profiles for PathoFact 2.0. The HMM profile dataset annotates the predicted VF domains rather than using them as input to the classification, as in the previous version.

To create the “non-VF” dataset for the ML VF model, we filtered out any potential VFs (with high and low probabilities), ARGs, and toxins based on PathoFact 1.0 predictions from the “non-pathogenic” dataset. This resulted in a dataset of 41,774 VF protein sequences (Fig. 2).

The VF model uses XGBoost with protein sequence composition features (step size shrinkage used in update to prevent overfitting [learning_rate] = 0.1, number of trees '*n*_estimators] = 2,000). The VF module generates a report containing the protein ID, protein domains, bitscore, VF ML probability, other identical proteins found in the sample, and optionally SignalP, plasmid marker, and virus marker information.

### ARG prediction updates

ARG prediction in PathoFact 2.0 integrates DeepARG (version 1.0.2; [45]), RGI (version 6; [6]), and AMRFinderPlus (version 3.12.8; [46]). DeepARG and RGI have received updates from their developers since the release of PathoFact 1.0, which have been incorporated into PathoFact 2.0. In addition, AMRFinderPlus has been newly integrated into PathoFact 2.0. Each tool has distinct strengths: DeepARG offers high precision and recall; RGI provides robust predictions based on an extensive database, utilizing homology and single-nucleotide polymorphism (SNP) models; and AMRFinderPlus efficiently identifies resistance genes and mutations using NCBI resources.

The ARG prediction module (Fig. 1) report includes protein IDs, ARG classes, prediction probabilities, database accession numbers, and optional data on signal peptides, plasmids, and virus markers. PathoFact 2.0 uses argNorm [47] to map detected genes to the ARO, thereby facilitating comparison of ARG annotation outputs by ensuring standardized and comparable results. Supplementary Figs S1A and S2A compare the performance of PathoFact 2.0 with that of its predecessor, PathoFact, in identifying ARGs.

### Additional functionalities

PathoFact 2.0 integrates SignalP (version 6; [24]) and antiSMASH (version 7.0; [28]), both of which are optional features that accommodate diverse research needs. SignalP is designed to predict the presence and location of signal peptides in protein sequences. It requires a separate license and must be requested by the user individually. AntiSMASH is designed to identify and annotate BGCs in bacterial and fungal genomes. Since AntiSMASH is a resource-intensive tool, we set it up as an optional module and provide the option to run it in chunks.

## Evaluation of the performance of the PathoFact 2.0 pipeline

**Figure 3:**
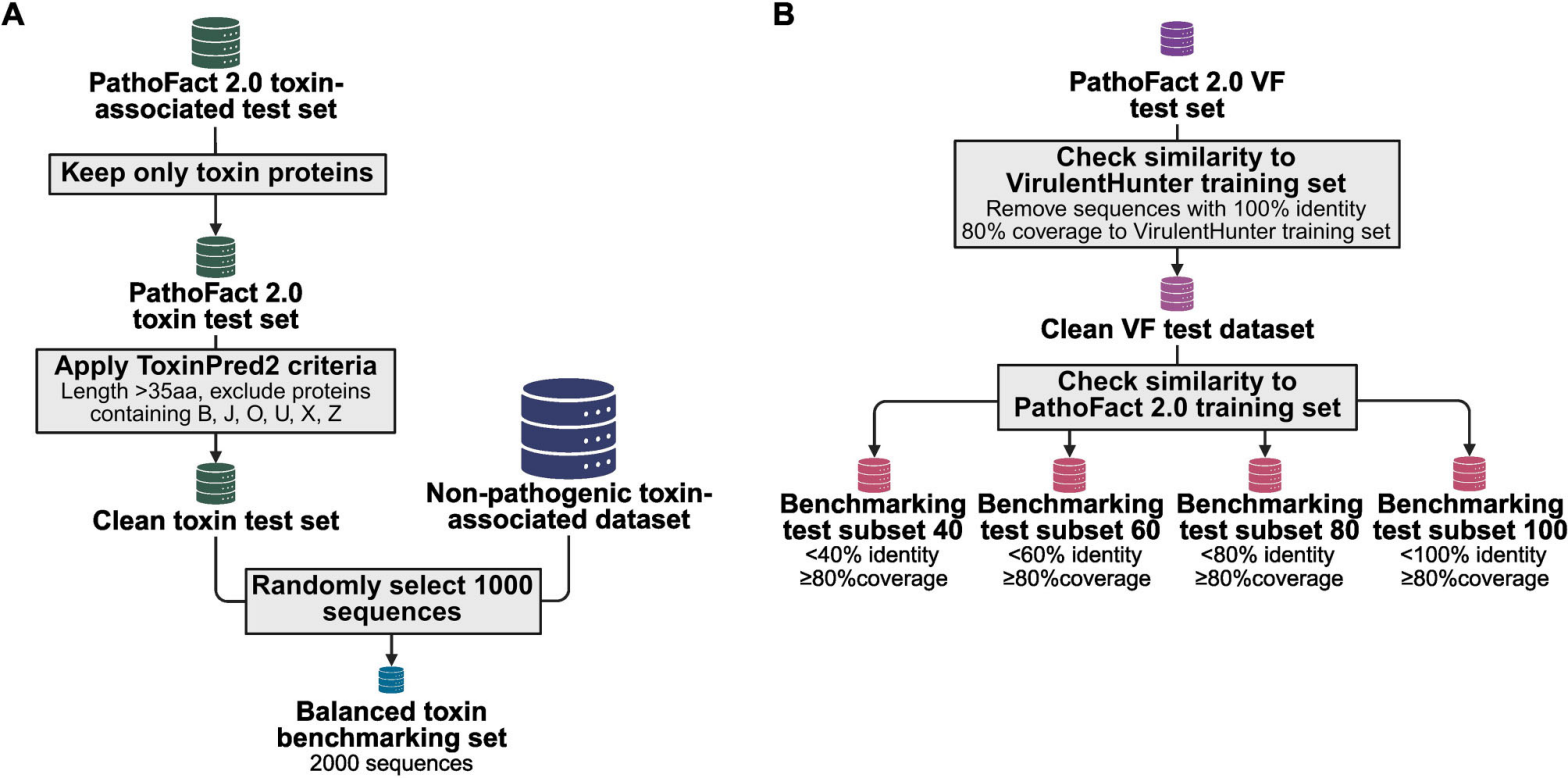
Schematic representation of the construction of the datasets used for benchmarking. (A) Toxin benchmarking dataset. (B) VF benchmarking dataset. Created in BioRender. Ortís Sunyer, J. (2026) https://BioRender.com/x9fn4zn.

We evaluated the performance of PathoFact 2.0 and the new toxin-associated and VF modules using the test datasets described above. We did not include ARGs and BGCs in the validation step, as the respective modules are based on existing tools that have already demonstrated high accuracy [6, 28, 45, 46]. Figure 3 provides a schematic overview of the benchmarking datasets used for the toxin-associated and VF modules.

### VFs and toxin-associated protein prediction

**Figure 4:**
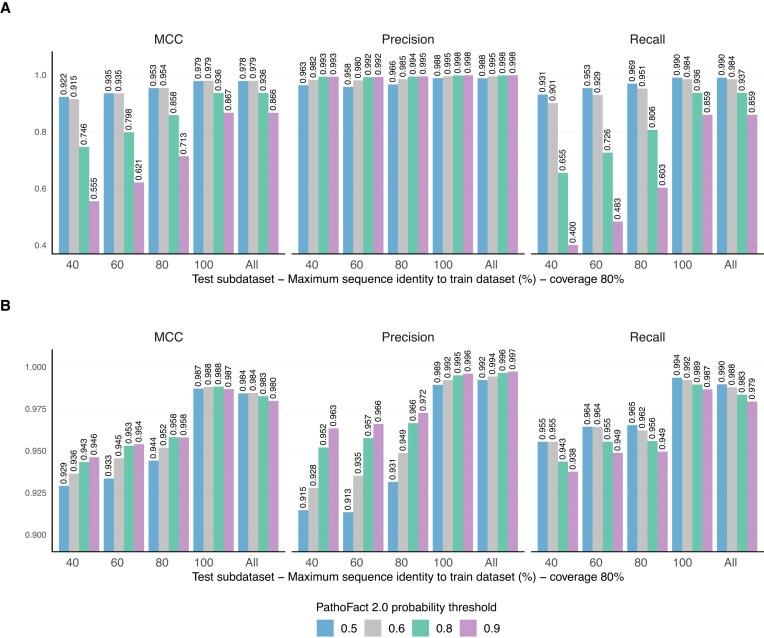
Performance evaluation of toxin-associated and VF prediction modules across probability thresholds. (A) Toxin-associated prediction module evaluation. (B) VFs prediction module evaluation. The modules were evaluated across a range of predicted class probabilities (0.5–0.9). The entire test dataset (All) and subsets of the test datasets were used for evaluation. These subset datasets were created based on sequence similarity to the training dataset, with similarity levels of 40, 60, 80, and 100%, and an 80% coverage threshold. Only sequences with similarity below these percentages were included in the respective test subsets.

The VF and toxin-associated modules (Fig. 1) were evaluated across different ML models-predicted probability thresholds, corresponding to the model’s confidence in the positive class (VF or toxin-associated). Analyses were performed on the full test dataset and on subsets of the test dataset. These subset datasets were created based on sequence similarity to the training dataset, with a range of 40–100% similarity and 80% coverage (Fig. 2). This approach aimed to assess prediction accuracy on proteins in the testing dataset with low similarity to the training dataset, specifically including only sequences with less than 40–100% identity to any training sequence. The performance evaluation is based on the MCC and the precision (to reduce the number of false positive VF and toxin-associated predictions), taking into account the dataset imbalance (a higher number of “non-toxin” and “non-VF” sequences compared to “toxin-associated” and “VF” sequences in the test subsets). The MCC is a more reliable statistical measure that yields a high score only when the prediction performs well across all four categories of the confusion matrix (true positives, false negatives, true negatives, and false positives), and it is proportional to both the number of positive and negative elements in the dataset [48]. We found that predicted probabilities of 0.6 for toxin-associated proteins and 0.9 for VFs provide a good balance between high MCC and precision across different test subsets (Fig. 4 and Supplementary Tables S3 and S4).

#### Benchmarking

The PathoFact 2.0 VF prediction module was compared to VirulentHunter [49], using the default parameters. VirulentHunter is a deep learning framework that simultaneously identifies and classifies VFs directly from protein sequences, which outperforms other VF predictors (MP4 [50], VirulentPred 2.0 [51], and DeepVF [52]). A notable feature of VirulentHunter is that it provides VF category classification; however, it takes about 2 min to analyse 500 protein sequences (using one GPU), which is a drawback for metagenomic sample analysis, where thousands to millions of proteins are predicted from a single sample. PathoFact 2.0 requires only 4 s (using one CPU, with the option to utilize more CPUs) to analyse 500 protein sequences (Table 1).

**Table 1:** Runtime comparison of PathoFact 2.0 and VirulentHunter.

	VirulentHunter	PathoFact 2.0				
Number of protein sequences	1 GPU	1 CPU	2 CPU	4 CPU	6 CPU	8 CPU
500	2 min 21 s	3.7 s	2.6 s	2.0 s	1.9 s	1.8 s
5,500	25 min 42 s	29.9 s	17.4 s	10.2 s	8.1 s	7.4 s
10,000	54 min 11 s	55.4 s	29.3 s	16.7 s	13.1 s	11.5 s
30,000	2 h 45 min 50 s	2 min 45 s	1 min 32.9 s	52.1 s	40.3 s	36.0 s

**Figure 5:**
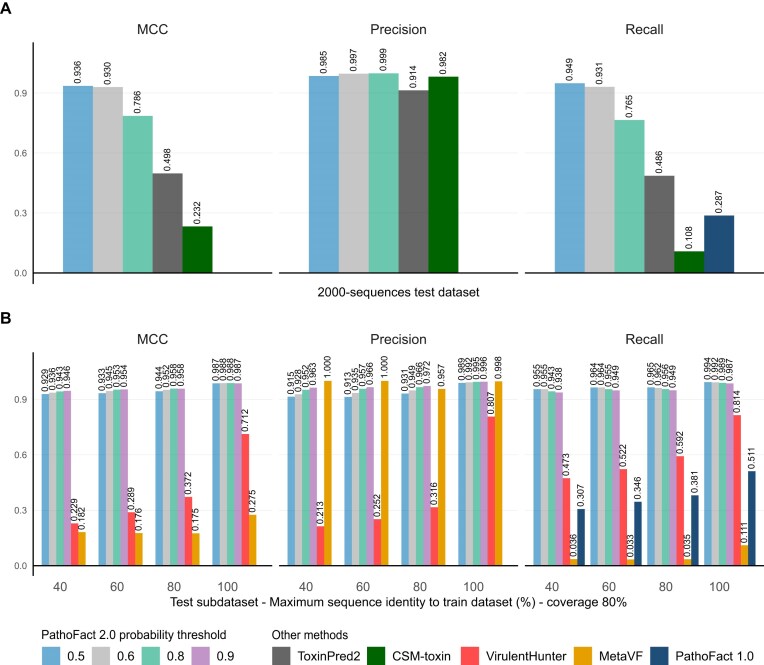
Benchmarking of toxin and VF prediction performance. (A) Toxin-associated module benchmarking. The PathoFact 2.0 toxin-associated module was compared with ToxinPred2 (Hybrid: RF+BLAST+MERCI, threshold = 0.6) using its web-based version and CSM-toxin v1.0.1. A balanced toxin test dataset (1,000 toxin and 1,000 non-toxin sequences) was built from the PathoFact 2.0 toxin-associated test dataset, selecting only sequences from curated toxin sources (TADB, SecReT6, T3DB, DBETH, SwissProt), applying ToxinPred2’s filtering criteria. Several predicted probability cutoffs, 0.5, 0.6, and 0.8, of the PathoFact 2.0 toxin-associated module were evaluated. MCC, precision, and recall are shown. (B) VFs module benchmarking. The PathoFact 2.0 VF module was compared to VirulentHunter. Sequences identical (100% identity, ≥80% coverage) to VirulentHunter’s training data were removed from the PathoFact 2.0 test dataset. Then, test subset datasets were created based on 40–100% similarity to the PathoFact 2.0 training set. These subset datasets were created based on sequence similarity to the training dataset, with similarity levels of 40, 60, 80, and 100%, and an 80% coverage threshold. Only sequences with similarity below these percentages were included in the respective test subsets. Several predicted probability cut-offs (0.5, 0.6, 0.8, and 0.9) of the PathoFact 2.0 VF module were evaluated. MCC, precision, and recall are presented for each test subset. Since the negative dataset was built and then filtered using PathoFact 1.0, the precision is 1 for PathoFact 1.0, therefore affecting MCC calculation. To avoid biased comparisons, we report recall only for PathoFact 1.0.

Since VirulentHunter and PathoFact 2.0 employ a similar method to generate the “VF dataset” for model training, we removed sequences from the PathoFact 2.0 test dataset that have 100% identity (≥80% coverage) to the VirulentHunter training dataset, resulting in a “clean VF test dataset” (Fig. 3). This ensures that neither model used the test sequences for training. We applied the same test-subset approach described earlier: the subset datasets were created based on sequence similarity to the PathoFact 2.0 training dataset, with similarity ranging from 40 to 100% and 80% coverage of the “clean VF test dataset” (Fig. 3). By stratifying test sets by decreasing sequence similarity to the training data, we explicitly evaluated model performance across progressively more divergent proteins. This study demonstrates that PathoFact 2.0 maintains consistent performance even when test sequences share 40% similarity or less with the training set (Fig. 5, Supplementary Fig. S3). In addition to VirulentHunter, we benchmarked PathoFact 2.0 against the VF prediction module of PathoFact 1.0 and against metaVF [23], an alignment-based toolkit (based on BLAST) that identifies species-level VFs associated with pathobionts. DNA gene sequences from the test subsets were used as input, since both tools accept only contigs.

The PathoFact 2.0 toxin-associated module was compared with ToxinPred2 [42] using the default parameters, i.e., Hybrid (RF+BLAST+MERCI) with a threshold of 0.6. The ToxinPred2 website restricts predictions to a certain number of proteins (around 2,000). Since ToxinPred2 is designed to predict protein toxicity, we selected sequences from the PathoFact 2.0 “toxin-associated” test dataset that are directly linked to toxins and removed the remaining “toxin-associated” proteins (Fig. 3). In short, using the header information from the PathoFact 2.0 “toxin-associated” test dataset, we kept only toxin sequences from the toxin–antitoxin sequences from the TADB, the effector factor sequence from the SecReT6 database, bacterial protein toxins from T3DB, bacterial exotoxins from DBETH, and sequences from Swissprot (KW-0800, toxin), as previously described. Additionally, we kept sequences longer than 35 amino acids and excluded protein sequences containing the non-standard amino acids “BJOUXZ” as the ToxinPred2 dataset was created using these criteria [53] (Fig. 3). From these, we randomly selected 1,000 sequences. Then, we randomly selected 1,000 sequences from the PathoFact 2.0 “non-toxin” test dataset (Fig. 3). This resulted in a total of 2,000 sequences for benchmarking PathoFact 2.0 against the ToxinPred2 webserver. Due to the limited number of sequences, we did not use the test-subset approach to evaluate ToxinPred2 and PathoFact 2.0 toxin-associated modules on this 2,000-sequence test dataset (Fig. 3). We also use the same test dataset to benchmark CSM-toxin v1.0.1, a deep learning model for toxin prediction [54], as well as the toxin prediction module from PathoFact 1.0. DNA gene sequences from the test dataset were used as input.

As shown in Fig. 5 (and in the Supplementary Tables S5 and S6 and the AUROC curves in Supplementary Figs S3 and S4), PathoFact 2.0 VF and toxin-associated modules exhibited higher MCC values across different test subsets compared to VirulentHunter, ToxinPred2, and CSM-toxin. Because the negative dataset was built and then filtered using PathoFact 1.0, the precision for PathoFact 1.0 is 1, which affects the MCC calculation. To avoid biased comparisons, we report recall only for PathoFact 1.0. Figure 5 shows that PathoFact 2.0 VF and toxin-associated modules achieved higher recall across test subsets than PathoFact 1.0, and better recall and MCC than MetaVF.

### Virulence factors and toxin-associated protein prediction with contig sequences as input

**Figure 6:**
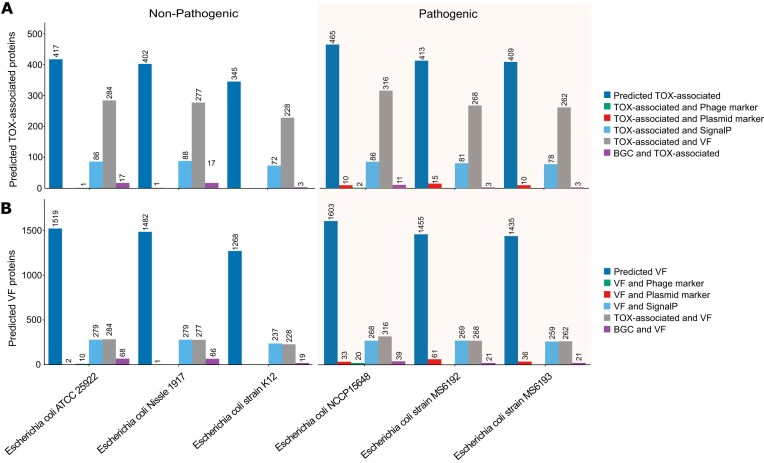
Comparative analysis of toxin-associated and VF profiles in non-pathogenic and pathogenic *E. coli* strains. Bar charts represent the distribution of predicted toxin-associated (A) and virulence-associated (B) proteins across non-pathogenic (left panel) and pathogenic (right panel) *E. coli* strains. The categories include total predicted VFs/toxin-associated proteins (dark blue), those associated with plasmid markers (red), those associated with phage markers (green), and proteins predicted by SignalP to be secreted (light blue). Additional categories include toxin-associated VFs (TOX-associated and VF, grey) and BGCs overlapping (purple). Numerical values above each bar indicate the total count of proteins identified in each category for the corresponding strain.

To evaluate PathoFact 2.0 at the contig level, we analysed publicly available complete genomes from pathogenic and non-pathogenic bacteria, including various *Escherichia coli* strains. Figure 6 shows distinct differences in virulence and toxin-related profiles between pathogenic and non-pathogenic *E. coli* strains, especially regarding virulence- and toxin-associated proteins encoded on MGEs, such as plasmids and prophages. Nonetheless, analysis of individual VF predictions reveals considerable overlap in the number of VF genes detected across both pathogenic and non-pathogenic strains. This highlights that the presence of a VF gene is not a reliable marker of pathogenicity and emphasizes the importance of considering genomic and functional context when assessing virulence potential.

It is well known that VFs of pathogenic *E. coli* are often encoded on genetic elements, such as plasmids, bacteriophages, transposons, and pathogenicity islands, which can be mobilized into different strains to create novel combinations of VFs [55, 56]. The same pattern is observed in pathogenic strains of several genera compared to non-pathogenic strains (Supplementary Fig. S5), particularly for *Klebsiella pneumoniae* and *Salmonella enterica*. These findings highlight the importance of examining virulence from a systems perspective rather than focusing solely on the presence or absence of individual factors. A comprehensive assessment should consider not only whether a virulence- or toxin-associated protein is encoded within an MGE but also its functional context, such as whether it is secreted or part of a BGC.

**Figure 7:**
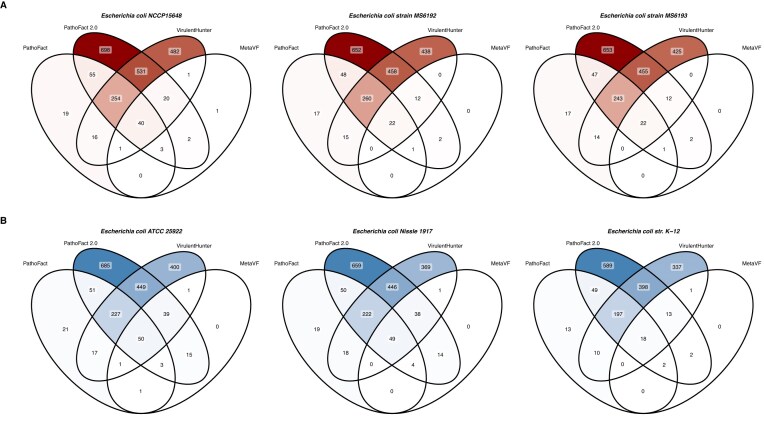
Performance comparison of VF prediction tools in pathogenic (A) and non-pathogenic (B) strains. Venn diagrams comparing the predictions of PathoFact 2.0, PathoFact 1.0, metaVF, and VirulentHunter in predicting VFs.

The PathoFact 2.0 VF module was compared to PathoFact 1.0 and metaVF. To our knowledge, no other method is available to predict VF from contig sequences and identify plasmid-encoded or prophage-associated VF. However, we included VirulentHunter in the comparison. Because VirulentHunter does not accept contig sequences as input, protein-coding genes were first predicted from contigs using Pyrodigal-gv, and the resulting protein sequences were subsequently analysed. PathoFact 2.0 consistently predicted a greater number of VFs than both PathoFact 1.0 and MetaVF (Fig. 7, Supplementary Figs S6–S8). Notably, MetaVF failed to identify any VFs in 5 of the 10 pathogenic reference strains tested, highlighting its limited ability to detect VFs and demonstrating the advantages of ML-based models over homology-based approaches. While VirulentHunter produced substantially more hits than metaVF, yielding predictions comparable in number to PathoFact 2.0, PathoFact 2.0 generally identified more VFs overall (Fig. 7, Supplementary Figs S6–S8). Exceptions were observed for non-pathogenic strains *Bifidobacterium animalis, B. bifidum, Heyndrickxia coagulans*, and for the pathogenic *Ralstonia mannitolilytica* strain *Guangzhou-RMAB10*, where VirulentHunter predicted slightly more VFs (Supplementary Figs S6 and S8).

**Figure 8:**
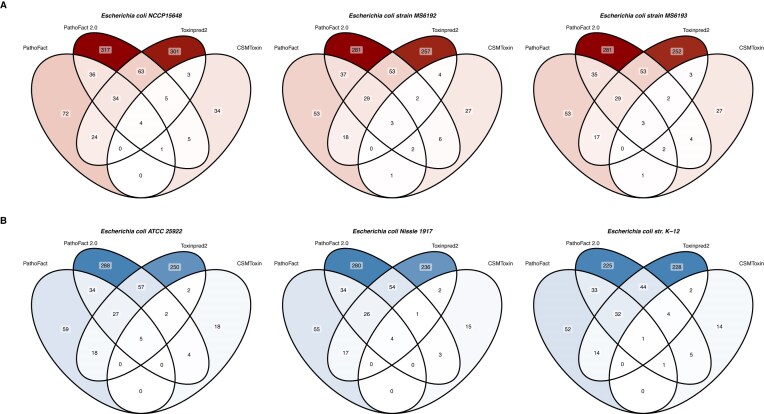
Performance comparison of toxin prediction tools in pathogenic (A) and non-pathogenic (B) strains. Venn diagrams comparing the predictions of PathoFact 2.0, PathoFact 1.0, CSM-Toxin, and ToxinPred2 in predicting toxins and toxin-associated proteins.

In addition, the PathoFact 2.0 toxin-associated module was compared to PathoFact 1.0 as well as CSM-Toxin and ToxinPred2 (Fig. 8, Supplementary Figs S1B, S2B, and S9). As CSM-Toxin and ToxinPred2 do not accept contig sequences as input, protein sequences were first predicted from contigs using Pyrodigal-gv. PathoFact 2.0 predicted more toxin-associated proteins than PathoFact 1.0, demonstrating improved detection capacity. Compared to external tools, CSM-Toxin identified substantially fewer toxins across the tested reference strains. In contrast, ToxinPred2 predicted a comparable number of toxins overall. However, for non-pathogenic strains *B. bifidum, H. coagulans, Lactobacillus acidophilus*, and for pathogenic strains *Streptococcus gallolyticus* subsp. *gallolyticus* and *S. parasuis* B26, ToxinPred2 identified more predicted toxins than PathoFact 2.0 (Supplementary Figs S2B and S9).

## PathoFact 2.0 output structure

PathoFact 2.0 creates a structured output directory that summarizes predictions from all analysis modules, including VFs, toxin-associated proteins, ARGs, MGEs, and BGCs. Each module generates dedicated result files corresponding to the underlying prediction tools (Supplementary File S1).

The primary summary file, combined_report.tsv, provides an integrated overview of high-confidence predictions across all modules in a tabular format. This table includes key information such as protein identifiers, bit scores (from HMM profiles), ML prediction scores, and outputs from DeepARG, RGI, SignalP, geNomad, and antiSMASH, thereby supporting downstream interpretation and candidate prioritization.

Proteins with prediction probabilities below user-defined thresholds but containing conserved domains identified by toxin-associated or VF HMM profiles are reported in ambiguous_TOX_hits_rep_prot.tsv and ambiguous_VF_hits_rep_prot.tsv. These lower-confidence candidates may warrant further investigation in comparative or experimental analyses.

High-confidence predictions are reported in amr_hits_rep_prot.tsv, TOX_hits_rep_prot.tsv, and VF_hits_rep_prot.tsv, which summarize features exceeding user-defined probability thresholds and include protein identifiers, bit scores, ML predictions, signal peptide predictions, and genomic context information, such as association with prophages or plasmids identified by geNomad.

In addition, PathoFact 2.0 generates a dedicated Group_of_sequence directory containing FASTA files of representative protein sequences grouped by functional category (VFs, toxin-associated proteins, antimicrobial resistance genes, and combined hits), together with conserved domain (CDD) annotation tables for predicted VFs and toxin-associated proteins. These files are designed to facilitate downstream analyses, including comparative genomics and functional characterization.

## Limitations of PathoFact 2.0

It is well established that non-pathogenic bacterial strains can also carry genes annotated as VFs or toxins [7]. Consequently, PathoFact 2.0 is most effective as an initial screening tool to identify potential candidates, which can then be examined in comparative studies to distinguish confirmed pathogenic cases from controls. The pipeline provides a probability score indicating whether a protein is likely to be VF- or toxin-associated; however, establishing a definitive link between predicted candidates and infectious disease requires experimental validation.

PathoFact 2.0 does not directly classify specific VF or toxin types (e.g., adhesins or genotoxins). Instead, it reports detailed annotations of conserved protein domains from CDD [18], allowing users to infer functional roles when available. This design emphasizes contextual interpretation rather than categorical assignment.

The inclusion of housekeeping genes from non-pathogenic microorganisms reflects a deliberate methodological choice rather than a limitation. Multiple well-characterized housekeeping proteins have been shown to exhibit virulence-associated “moonlighting” functions in pathogenic bacteria, including roles in adhesion, immune modulation, and tissue invasion [57, 58]. Notable examples include glyceraldehyde-3-phosphate dehydrogenase (VF0015 in VFDB) [59], enolase [58, 57], elongation factor Tu (VF0460 in VFDB) [60, 61], GroEL [57, 62, 63], and DnaK [57, 64]. This approach maintains a biologically realistic negative dataset while reducing the risk of misclassifications within the intended scope of PathoFact 2.0.

The divergence between PathoFact 2.0, PathoFact 1.0, VirulentHunter, MetaVF, ToxinPred2, and CSM-Toxin predictions reflects fundamental differences in model design, training datasets, prediction thresholds, and biological scope, rather than sensitivity alone. PathoFact 2.0 was developed as a conservative metagenomic screening framework and therefore applies stringent ML probability thresholds (0.9 for VFs and 0.6 for toxin-associated proteins), selected based on the MCC and precision benchmarking. In contrast, VirulentHunter uses a lower default threshold (0.5), which increases the number of positive predictions, including those with lower confidence. Similarly, PathoFact 1.0 was built using a positive subset of known VF sequences retrieved from the Virulence Factors Database (included 8,945 sequences), while the negative subset of the training set consisted of protein sequences retrieved from the Database of Essential Genes (included 7,995 sequences) [65]. The PathoFact 1.0 VF score is based on a combination of ML output and HMM homology, whereas the toxin prediction is based solely on HMM homology.

The prediction scope also differs substantially between methods. ToxinPred2 focuses on toxin protein prediction using a hybrid framework that combines ML, BLAST similarity, and MERCI motif detection. CSM-Toxin is a deep learning approach for protein toxicity classification that relies on the protein’s primary sequence. PathoFact 2.0 targets a broader class of toxin-associated proteins, including toxins, antitoxins, secretion-associated effectors, regulators, and accessory proteins. Similarly, VirulentHunter was developed as a VF category classifier trained on a relatively small, imbalanced dataset. These methodological differences result in partially overlapping but distinct prediction spaces.

PathoFact 2.0 additionally employs a highly curated negative dataset of non-pathogenic microorganisms, including housekeeping proteins, while filtering out potential ARGs, VFs, and toxins. This conservative strategy is designed to reduce false-positive predictions in metagenomic datasets, particularly given the documented moonlighting virulence functions of several canonical housekeeping proteins. Consequently, proteins uniquely predicted by VirulentHunter, PathoFact 1.0, CSM-Toxin, or ToxinPred2 likely include borderline or lower-confidence candidates that are excluded by the stricter classification framework of PathoFact 2.0.

PathoFact 2.0 is designed for metagenomic samples; most prediction modules and phenotypes are bacterial-centric. Virulence, toxin-associated, and antimicrobial resistance predictions are particularly interpreted in the context of human pathogens.

## Conclusions

ARGs, VFs, and toxins represent major threats to global health. Therefore, accurate detection of these elements is crucial for assessing the presence and potential risks of pathogenic microorganisms in microbiomes and for identifying reservoirs of pathogenicity. Our improved pipeline, PathoFact 2.0, offers significant improvements over PathoFact (its predecessor), ToxinPred2, CSM-toxin, VirulentHunter, and metaVF. SignalP has been upgraded and made optional to further optimize performance, providing users with flexibility based on their requirements. Additionally, antiSMASH 7.0 facilitates the prediction of BGCs, recognizing emerging evidence that some BGC-encoded factors might increase virulence. Furthermore, we have integrated geNomad, a cutting-edge tool for identifying MGEs, including plasmids and phages linked to ARGs, VFs, toxins, and toxin-associated proteins across various bacterial species. The PathoFact 2.0 update improves the accuracy and sensitivity of analyses while enhancing computational efficiency.

PathoFact 2.0 represents a major advance in metagenomic analysis by integrating the detection of ARGs, VFs, toxins, and toxin-associated proteins, signal peptides, MGEs, and BGCs within a single, streamlined pipeline. Unlike existing tools that focus on individual aspects of pathogenicity, PathoFact 2.0 provides a comprehensive, multi-layered view that captures both gene presence and genomic context, improving interpretability and enabling a holistic assessment of microbial pathogenic potential.

## Methods

### Databases used for the PathoFact 2.0 dataset construction

- SwissProt [31] is the expertly curated part of UniProtKB [66]. It offers high-quality protein sequences with detailed functional annotations, including keywords for pathogenesis, virulence, toxins, and antibiotic resistance.
- VFDB [44], the VFDB, is a comprehensive reference for curating information on VFs of bacterial pathogens.
- T3DB [12], the toxin and toxin-target database, is a resource cataloguing thousands of toxins and their protein targets, with detailed mechanisms, structures, and toxicity data, including bacterial protein toxins.
- DBETH [13], the DBETH, is a specialized database of bacterial exotoxins pathogenic to humans, classified into 24 mechanistic and activity types from 26 bacterial genera.
- TADB [37], the toxin–antitoxin database, is a repository of bacterial toxin–antitoxin loci across types I–VIII, including experimentally validated pairs, predicted loci, and associations with MGEs.
- SecReT6 [38] is a database containing known and predicted type VI secretion systems, including effectors, immunity proteins, regulators, and accessory proteins from bacterial genomes.
- PAT [39] is the PAT database and contains a collection of antimicrobial toxins, including bacteriocins and effectors from secretion systems.

### Clustering parameters

MMseqs2 [33] was used for dereplication; sequences were clustered at 100% identity and 100% coverage using the parameters -c 1.0 and --min-seq-id 1.0. It removes exact duplicates and retains representative sequences to generate non-redundant datasets. To create the test subsets, a coverage of 80% (-c 0.8) and identities ranging from 40 to 100% (--min-seq-id 0.4, --min-seq-id 0.6, --min-seq-id 0.8, --min-seq-id 1.0) were used, and then sequences with similarity higher than min-seq-id were removed for each test subset. In all cases, the cluster mode and coverage mode used were 0 (--cov-mode 0 --cluster-mode 0). When --cov-mode 0 is specified in combination with -c values ranging from 0.0 to 1.0, sequences are assigned to the same cluster only if the alignment spans at least a fraction *c* of the length of the longer sequence. According to the developers of MMSeq2, this coverage criterion is particularly suitable for clustering full-length protein sequences [33].

### Protein composition features

Protein sequence composition features were extracted to represent each protein as fixed-length vectors derived from its primary amino acid sequence [36]. These included AAC, DPC, CTDC, CTDT, and CTDD. AAC captures the relative frequency of each of the 20 amino acids in a sequence, whereas DPC captures the relative frequency of all adjacent amino-acid pairs (400 possible dipeptides). CTDC represents the percentage of amino acids belonging to each of 3 predefined groups (polar, neutral, hydrophobic) in the entire protein sequence. CTDT represents the percentage frequency with which a residue of one group is followed by a residue of a different group along the sequence. CTDD represents the distribution of each amino acid group, measuring the spatial position, where the first, 25, 50, 75, and 100% of the residues of a specific class are located.

### Performance and evaluation metrics

To assess the models’ performance, we used a confusion matrix comprising true positives (TP), true negatives (TN), false positives (FP), and false negatives (FN), computed on the test datasets described above. TP corresponds to truly positive instances correctly predicted as positive by the model, whereas TN corresponds to truly negative instances correctly predicted as negative. FP are truly negative instances incorrectly predicted as positive, and FN are truly positive instances incorrectly predicted as negative. From these values, we calculated the following metrics:


\begin{eqnarray*}
{\mathrm{ Accuracy}}\ = \ \left( {\mathrm{ TP}\ + \ \mathrm{ TN}} \right)/\left( {\mathrm{ TP} + \mathrm{ TN} + \mathrm{ FP} + \mathrm{ FN}} \right),
\end{eqnarray*}

\begin{eqnarray*}
{\mathrm{ Precision}}\ = \ \mathrm{ TP}/\left( {\mathrm{ TP}\ + \mathrm{ FP}} \right),
\end{eqnarray*}

\begin{eqnarray*}
{\mathrm{ Recall}}\ = \ \mathrm{ TP}/\left( {\mathrm{ TP} + \mathrm{ FN}} \right),
\end{eqnarray*}

\begin{eqnarray*}
\mathrm{ MCC} & =& \left( {\mathrm{ TP} \times \mathrm{ TN} - \mathrm{ FP} \times \mathrm{ FN}} \right)/\\&& \sqrt {\left( {\mathrm{ TP} + \mathrm{ FP}} \right)\left( {\mathrm{ TP} + \mathrm{ FN}} \right)\left( {\mathrm{ TN} + \mathrm{ FP}} \right)\left( {\mathrm{ TN} + \mathrm{ FN}} \right)}.
\end{eqnarray*}

### HMM profiles

Profile HMMs are probabilistic models built from a multiple sequence alignment that encode, for each alignment position, the position-specific probabilities of residues and insertions/deletions, turning the alignment into a position-specific scoring system for detecting homologous sequences [67]. The FASTA files of conserved-domain multiple sequence alignments for each CDD [26] family (https://ftp.ncbi.nih.gov/pub/mmdb/cdd/fasta.tar.gz) were downloaded. Pyhmmer v0.10.14 [68] was used to obtain HMM profiles for each CDD family and to perform protein sequence searches against the CDD HMM family profiles.

### Benchmarking datasets

This study utilized publicly available datasets containing complete genomes from pathogenic and non-pathogenic bacteria, including various *E. coli* strains, from NCBI. The accession numbers of the bacteria used are indicated in Supplementary Table S2.

## Availability of source code and requirements

Project name: PathoFact 2.0

Project homepage: https://gitlab.com/uniluxembourg/lcsb/systems-ecology/pathofact2

Operating system(s): Linux

Programming language: Python, R, bash

Other requirements: Snakemake, Mamba, conda.

License: GNU General License v3.0 or later

Biotools ID: pathofact2

RRID:SCR_027650

Workflowhub: https://doi.org/10.48546/workflowhub.workflow.2087.1

## Additional files

**Supplementary Table S1:** List of microorganisms non-pathogenic to humans and their total protein count obtained from the NCBI Database.

**Supplementary Table S2:** Bacterial strains used in this study, including their classification as pathogenic or non-pathogenic, species/strain information, genome assembly or reference version, and corresponding accession numbers.

**Supplementary Table S3:** Evaluation of the PathoFact 2.0 toxin-associated protein prediction module. The table presents performance across test subsets defined by sequence similarity to the training set. Metrics reported include class distributions (negative, non-toxin; positive, toxin-associated), confusion matrix counts (true negatives, false positives, true positives, false negatives), and performance measures (accuracy, precision, recall, *F*1 score, Matthews correlation coefficient).

**Supplementary Table S4:** Evaluation of the PathoFact 2.0 virulence factor prediction module. The table summarizes performance across test subsets defined by sequence similarity to the training set. Reported metrics include class distributions (negative, non-VF; positive, VF), confusion matrix counts, and performance measures (accuracy, precision, recall, *F*1 score, Matthews correlation coefficient).

**Supplementary Table S5:** Comparison of virulence factor prediction performance for PathoFact 2.0 at varying probability cutoffs and for VirulenHunter, evaluated across test subsets stratified by sequence similarity to the training set. The table reports class distributions, confusion matrix counts, and performance metrics (accuracy, precision, recall, *F*1 score, Matthews correlation coefficient).

**Supplementary Table S6:** Comparison of toxin protein prediction performance for PathoFact 2.0 at different prediction probability cutoffs and for ToxinPred2. The table reports the number of proteins classified as negative (non-toxin) and positive (toxin), confusion matrix counts, and associated performance metrics (accuracy, precision, recall, *F*1 score, and Matthews correlation coefficient).

**Supplementary Figure S1:** Performance comparison of PathoFact 2.0 versus PathoFact for ARG and toxin prediction in *Escherichia coli*. (A) Comparative performance of PathoFact 2.0 (blue) versus PathoFact (green) in predicting antimicrobial resistance genes (ARGs) in non-pathogenic (left) and pathogenic (right) *E. coli* strains. (B) Comparative performance of PathoFact 2.0 (blue) versus PathoFact (green) in predicting toxin-associated proteins in non-pathogenic (left) and pathogenic (right) *E. coli* strains. The top panel shows the total number of predicted toxin-associated proteins, while the bottom panel shows those that contain signal peptides, as identified by SignalP.

**Supplementary Figure S2:** Performance comparison of PathoFact 2.0 versus PathoFact for ARG and toxin prediction in bacterial strains. (A) Comparative performance of PathoFact 2.0 (blue) versus PathoFact (green) in predicting antimicrobial resistance genes (ARGs) in non-pathogenic (left) and pathogenic (right) bacterial strains. (B) Comparative performance of PathoFact 2.0 (blue) versus PathoFact (green) in predicting toxin-associated proteins in non-pathogenic (left) and pathogenic (right) bacterial strains. The top panel shows the total number of predicted toxin-associated proteins, while the bottom panel shows those that contain signal peptides, as identified by SignalP.

**Supplementary Figure S3:** Area under the receiver operating characteristic (AUROC) curves comparing the virulence factor prediction performance of PathoFact 2.0 (A) and Virulent Hunter (B) on the benchmark datasets. Curves represent performance across different benchmark subsets: 40 (red), 60 (yellow), 80 (green), 100 (blue), and the complete dataset (All, purple). AUROC curves are not available with PathoFact 1.0 because it uses a combination of machine learning and HMM classification. MetaVF is an alignment-based algorithm, and it does not generate probability scores; therefore, AUROC curves are not possible.

**Supplementary Figure S4:** Area under the receiver operating characteristic (AUROC) curves comparing the virulence factor prediction performance of PathoFact 2.0 (A) and CSM-toxin (B) on the benchmark dataset. Curves correspond to PathoFact 2.0 (blue) and CSM-toxin (red). AUROC curves are not possible with Toxinpred2, since its predictions are based on a combination of factors rather than on machine-learning probabilities alone. Nor are they possible with PathoFact 1.0, which also uses a combination of machine learning and HMM classification.

**Supplementary Figure S5:** Comparative analysis of virulence factor profiles in non-pathogenic and pathogenic bacterial strains. Bar charts represent the distribution of predicted toxin-associated (A) and virulence-associated (B) proteins across non-pathogenic (left panel) and pathogenic (right panel) strains. The categories include total predicted virulence factors/toxin-associated proteins (dark blue), associated with plasmid markers (red), phage markers (green), and proteins predicted by SignalP to be secreted (light blue). Additional categories include toxin-associated virulence factors (TOX-related ∩ VF, grey) and biosynthetic gene clusters overlapping (purple). Numerical values above each bar indicate the total count of proteins identified in each category for the corresponding strain.

**Supplementary Figure S6:** Venn diagrams comparing the predictions of PathoFact 2.0, PathoFact, metaVF, and VirulentHunter in predicting virulence factors in pathogenic (A) and non-pathogenic (B) strains.

**Supplementary Figure S7:** Comparative performance of PathoFact 2.0 (blue) versus PathoFact1 (green) and metaVF (red) in predicting virulence factors (VFs) in non-pathogenic (left) and pathogenic (right) *Escherichia coli* strains. The top panel shows the total number of predicted VFs. The second panel depicts the subset predicted to be secreted. The third panel shows VFs predicted to be plasmid-encoded, while the fourth panel presents those predicted to be prophage-associated.

**Supplementary Figure S8:** Comparative performance of PathoFact 2.0 (blue) versus PathoFact (green) and metaVF (red) in predicting virulence factors (VFs) in non-pathogenic (left) and pathogenic (right) bacterial strains. The top panel shows the total number of predicted VFs. The second panel depicts the subset predicted to be secreted. The third panel shows VFs predicted to be plasmid-encoded, while the fourth panel presents those predicted to be prophage-associated.

**Supplementary Figure S9:** Venn diagrams comparing the predictions of PathoFact 2.0, PathoFact, Toxinpred2, and CSM-Toxin in predicting toxins and toxin-associated proteins in pathogenic (A) and non-pathogenic (B) strains.

## Abbreviations

AAC, amino acid composition; ARGs, antimicrobial resistance genes; ARO, antibiotic resistance ontology; BGCs, biosynthetic gene clusters; CDD, conserved domains database; CTDC, (composition, transition, distribution)-composition; CTDD, (composition, transition, distribution)-distribution; CTDT, (composition, transition, distribution)-transition; DBETH, database for bacterial exotoxins; DPC, dipeptide composition; HMMs, hidden Markov models; MCC, Matthews correlation coefficient; MGEs, mobile genetic elements; ML, machine learning; ORF, open reading frame; PAT, prokaryotic antimicrobial toxins database; RF, random forest; SM, specialized metabolites; SMOTE, synthetic minority oversampling technique; SNP, single nucleotide polymorphisms; T3DB, toxin exposome database; VFDB, virulence factor database; VFs, virulence factors.

## Supplementary Material

giag062_Supplemental_Files

giag062_Authors_Response_To_Reviewer_Comments_original_submission

giag062_Authors_Response_To_Reviewer_Comments_revision_1

giag062_Authors_Response_To_Reviewer_Comments_revision_2

giag062_GIGA-D-25-00455_original_submission

giag062_GIGA-D-25-00455_revision_1

giag062_GIGA-D-25-00455_revision_2

giag062_GIGA-D-25-00455_revision_3

giag062_Reviewer_1_Report_original_submission Reviewer 1 -- 12/10/2025

giag062_Reviewer_2_Report_original_submission Reviewer 2 -- 12/28/2025

giag062_Reviewer_2_Report_revision_1 Reviewer 2 -- 4/6/2026

giag062_Reviewer_2_Report_revision_2 Reviewer 2 -- 5/7/2026

giag062_Reviewer_3_Report_original_submission Reviewer 3 -- 1/3/2026

## Data Availability

PathoFact 2.0 is accessible at https://gitlab.com/uniluxembourg/lcsb/systems-ecology/pathofact2. Additionally, the core databases required to run the pipeline can be found at https://zenodo.org/records/14192463. The ML datasets used for training, validation, and benchmarking of PathoFact 2.0 can be found at https://zenodo.org/records/17647372.

## References

[1] Hou K, Wu Z-X, Chen X-Y et al. Microbiota in health and diseases. Sig Transduct Target Ther. 2022;7:135. 10.1038/s41392-022-00974-4. PMC9034083 35461318

[2] Inda-Díaz J S, Lund D, Parras-Moltó M et al. Latent antibiotic resistance genes are abundant, diverse, and mobile in human, animal, and environmental microbiomes. Microbiome. 2023;11:44. 10.1186/s40168-023-01479-0. 36882798 PMC9993715

[3] Beceiro A, Tomás M, Bou G. Antimicrobial resistance and virulence: a successful or deleterious association in the bacterial world?. Clin Microbiol Rev. 2013;26:185–230. 10.1128/CMR.00059-12. 23554414 PMC3623377

[4] Zhu C, Wu L, Ning D et al. Global diversity and distribution of antibiotic resistance genes in human wastewater treatment systems. Nat Commun. 2025;16:4006. 10.1038/s41467-025-59019-3. 40301344 PMC12041579

[5] Jian Z, Zeng L, Xu T et al. Antibiotic resistance genes in bacteria: occurrence, spread, and control. J Basic Microbiol. 2021;61:1049–70. 10.1002/jobm.202100201. 34651331

[6] Alcock B P, Huynh W, Chalil R et al. CARD 2023: expanded curation, support for machine learning, and resistome prediction at the Comprehensive Antibiotic Resistance Database. Nucleic Acids Res. 2023;51:D690–99. 10.1093/nar/gkac920. 36263822 PMC9825576

[7] Niu C, Yu D, Wang Y et al. Common and pathogen-specific virulence factors are different in function and structure. Virulence. 2013;4:473–82. 10.4161/viru.25730. 23863604 PMC5359729

[8] Sharma A K, Dhasmana N, Dubey N et al. Bacterial virulence factors: secreted for survival. Indian J Microbiol. 2017;57:1–10. 10.1007/s12088-016-0625-1. 28148975 PMC5243249

[9] Blair JMA, Webber M A, Baylay A J et al. Molecular mechanisms of antibiotic resistance. Nat Rev Micro. 2015;13:42–51. 10.1038/nrmicro3380. 25435309

[10] Rodríguez-Beltrán J, DelaFuente J, León-Sampedro R et al. Beyond horizontal gene transfer: the role of plasmids in bacterial evolution. Nat Rev Micro. 2021;19:347–59. 10.1038/s41579-020-00497-1. 33469168

[11] Galanos C, Freudenberg M A. Bacterial endotoxins: biological properties and mechanisms of action. Mediators Inflamm. 1993;2:S11–16. 10.1155/S0962935193000687. 18475562 PMC2365449

[12] Wishart D, Arndt D, Pon A et al. T3DB: the toxic exposome database. Nucleic Acids Res. 2015;43:D928–34. 10.1093/nar/gku1004. 25378312 PMC4383875

[13] Chakraborty A, Ghosh S, Chowdhary G et al. DBETH: a database of bacterial ExoToxins for human. Nucleic Acids Res. 2012;40:D615–20. 10.1093/nar/gkr942. 22102573 PMC3244994

[14] Green E R, Mecsas J. Bacterial secretion systems: an overview. Microbiol Spectr. 2016;4:1. 10.1128/microbiolspec.VMBF-0012-2015. PMC4804464 26999395

[15] Kaushik S, He H, Dalbey R E. Bacterial signal peptides—navigating the journey of proteins. Front Physiol. 2022;13: 933153. 10.3389/fphys.2022.933153. 35957980 PMC9360617

[16] Lybbert A C, Williams J L, Raghuvanshi R et al. Mining public mass spectrometry data to characterize the diversity and ubiquity of *P. aeruginosa* specialized metabolites. Metabolites. 2020;10:445. 10.3390/metabo10110445. 33167332 PMC7694397

[17] Elshafie H S, Camele I. An overview of metabolic activity, beneficial and pathogenic aspects of *Burkholderia* spp. Metabolites. 2021;11:321. 10.3390/metabo11050321. 34067834 PMC8156019

[18] Lau G W, Hassett D J, Ran H et al. The role of pyocyanin in *Pseudomonas aeruginosa* infection. Trends Mol Med. 2004;10:599–606. 10.1016/j.molmed.2004.10.002. 15567330

[19] Ambassadors G . Patrons: World Leaders Commit to Decisive Action on Antimicrobial Resistance. UN Environment. https://www.unep.org/news-and-stories/press-release/world-leaders-commit-decisive-action-antimicrobial-resistance. 2024. Accessed 7 October 2025.

[20] Environment UN: Antimicrobial Resistance (AMR) . UNEP—UN Environment Programme. https://www.unep.org/topics/chemicals-and-pollution-action/pollution-and-health/antimicrobial-resistance-amr, 2024. Accessed 7 October 2025.

[21] Bansal M A, Sharma D R, Kathuria D M. A systematic review on data scarcity problem in deep learning: solution and applications. ACM Comput Surv. 2022;54:1–29. 10.1145/3502287.

[22] de Nies L, Lopes S, Busi S B et al. PathoFact: a pipeline for the prediction of virulence factors and antimicrobial resistance genes in metagenomic data. Microbiome. 2021;9:49. 10.1186/s40168-020-00993-9. 33597026 PMC7890817

[23] Dong W, Fan X, Guo Y et al. An expanded database and analytical toolkit for identifying bacterial virulence factors and their associations with chronic diseases. Nat Commun. 2024;15:8084. 10.1038/s41467-024-51864-y. 39278950 PMC11402979

[24] Ji B, Pi W, Liu W et al. HyperVR: a hybrid deep ensemble learning approach for simultaneously predicting virulence factors and antibiotic resistance genes. NAR Genom Bioinform. 2023;5:lqad012. 10.1093/nargab/lqad012. 36789031 PMC9918863

[25] Rathore A S, Choudhury S, Arora A et al. ToxinPred 3.0: an improved method for predicting the toxicity of peptides. Comput Biol Med. 2024;179:108926. 10.1016/j.compbiomed.2024.108926. 39038391

[26] Kasmanas J C, Magnúsdóttir S, Zhang J et al. Integrating comparative genomics and risk classification by assessing virulence, antimicrobial resistance, and plasmid spread in microbial communities with gSpreadComp. Gigascience. 2025;14:giaf072. 10.1093/gigascience/giaf072. PMC12199706 40569694

[27] Wang J, Chitsaz F, Derbyshire M K et al. The conserved domain database in 2023. Nucleic Acids Res. 2023;51:D384–88. 10.1093/nar/gkac1096. 36477806 PMC9825596

[28] Blin K, Shaw S, Augustijn H E et al. antiSMASH 7.0: new and improved predictions for detection, regulation, chemical structures and visualisation. Nucleic Acids Res. 2023;51:W46–50. 10.1093/nar/gkad344. 37140036 PMC10320115

[29] Pyrodigal: python bindings and interface to Prodigal, an efficient method for gene prediction in prokaryotes. J Open Source Softw. 7:4296. 10.21105/joss.04296.

[30] Camargo A P, Roux S, Schulz F et al. Identification of mobile genetic elements with geNomad. Nat Biotechnol. 2024;42:1303–12. 10.1038/s41587-023-01953-y. 37735266 PMC11324519

[31] Köster J, Rahmann S. Snakemake—a scalable bioinformatics workflow engine. Bioinformatics. 2012;28:2520–22. 10.1093/bioinformatics/bts480. 22908215

[32] UniProt Consortium . UniProt: the universal protein knowledgebase in 2023. Nucleic Acids Res. 2023;51:D523–31. 10.1093/nar/gkac1052. 36408920 PMC9825514

[33] Steinegger M, Söding J. MMseqs2 enables sensitive protein sequence searching for the analysis of massive data sets. Nat Biotechnol. 2017;35:1026–28. 10.1038/nbt.3988. 29035372

[34] Chawla N V, Bowyer K W, Hall L O et al. SMOTE: synthetic minority over-sampling technique. J Artif Intell Res. 2002;16:321–57. 10.1613/jair.953.

[35] Pedregosa F, Varoquaux G, Gramfort A et al. Scikit-learn: machine learning in Python. J Mach Learn Res. 2011;12:2825–30.

[36] Chen Z, Zhao P, Li F et al. iFeature: a Python package and web server for features extraction and selection from protein and peptide sequences. Bioinformatics. 2018;34:2499–02. 10.1093/bioinformatics/bty140. 29528364 PMC6658705

[37] Guan J, Chen Y, Goh Y-X et al. TADB 3.0: an updated database of bacterial toxin–antitoxin loci and associated mobile genetic elements. Nucleic Acids Res. 2024;52:D784–90. 10.1093/nar/gkad962. 37897352 PMC10767807

[38] Zhang J, Guan J, Wang M et al. SecReT6 update: a comprehensive resource of bacterial type VI Secretion Systems. Sci China Life Sci. 2023;66:626–34. 10.1007/s11427-022-2172-x. 36346548

[39] Liu Y, Liu S, Pan Z et al. PAT: a comprehensive database of prokaryotic antimicrobial toxins. Nucleic Acids Res. 2023;51:D452–59. 10.1093/nar/gkac879. 36243963 PMC9825508

[40] Harms A, Liesch M, Körner J et al. A bacterial toxin–antitoxin module is the origin of inter-bacterial and inter-kingdom effectors of *Bartonella*. PLoS Genet. 2017;13:e1007077. 10.1371/journal.pgen.1007077. 29073136 PMC5675462

[41] Yadav S K, Magotra A, Ghosh S et al. Immunity proteins of dual nuclease T6SS effectors function as transcriptional repressors. EMBO Rep. 2021;22:e51857. 10.15252/embr.202051857. 33786997 PMC8183406

[42] Danov A, Segev O, Bograd A et al. Toxinome—the bacterial protein toxin database. mBio. 2024;15:e0191123. 10.1128/mbio.01911-23. 38117054 PMC10790787

[43] Sordo M, Zeng Q. On sample size and classification accuracy: a performance comparison. In: Oliveira J L, Maojo V, Martín-Sánchez F, Pereira A S, eds. Biological and Medical Data Analysis. Berlin: Springer; 2005.; 10.1007/11573067_20

[44] Liu B, Zheng D, Zhou S et al. VFDB 2022: a general classification scheme for bacterial virulence factors. Nucleic Acids Res. 2022;50:D912–17. 10.1093/nar/gkab1107. 34850947 PMC8728188

[45] Arango-Argoty G, Garner E, Pruden A et al. DeepARG: a deep learning approach for predicting antibiotic resistance genes from metagenomic data. Microbiome. 2018;6:23. 10.1186/s40168-018-0401-z. 29391044 PMC5796597

[46] Feldgarden M, Brover V, Gonzalez-Escalona N et al. AMRFinderPlus and the Reference Gene Catalog facilitate examination of the genomic links among antimicrobial resistance, stress response, and virulence. Sci Rep. 2021;11:12728. 10.1038/s41598-021-91456-0. 34135355 PMC8208984

[47] Ugarcina P erovic S, Ramji V, Chong H et al. argNorm: normalization of antibiotic resistance gene annotations to the Antibiotic Resistance ontology (ARO). Bioinformatics. 2025;41:btaf173. 10.1093/bioinformatics/btaf173. 40238188 PMC12064170

[48] Chicco D, Jurman G. The advantages of the Matthews correlation coefficient (MCC) over F1 score and accuracy in binary classification evaluation. BMC Genomics. 2020;21:6. 10.1186/s12864-019-6413-7. 31898477 PMC6941312

[49] Chen C, Xu Y, Ouyang J et al. VirulentHunter: deep learning-based virulence factor predictor illuminates pathogenicity in diverse microbial contexts. Brief Bioinform. 2025;26:bbaf271. 10.1093/bib/bbaf271. 40518950 PMC12167765

[50] Gupta A, Malwe A S, Srivastava G N et al. MP4: a machine learning based classification tool for prediction and functional annotation of pathogenic proteins from metagenomic and genomic datasets. BMC Bioinf. 2022;23:507. 10.1186/s12859-022-05061-7. PMC9703692 36443666

[51] Sharma A, Garg A, Ramana J et al. VirulentPred 2.0: an improved method for prediction of virulent proteins in bacterial pathogens. Protein Sci. 2023;32:e4808. 10.1002/pro.4808. 37872744 PMC10659933

[52] Xie R, Li J, Wang J et al. DeepVF: a deep learning-based hybrid framework for identifying virulence factors using the stacking strategy. Brief Bioinform. 2021;22. 10.1093/bib/bbaa125. PMC8138837 32599617

[53] Sharma N, Naorem L D, Jain S et al. ToxinPred2: an improved method for predicting toxicity of proteins. Brief Bioinform. 2022;23:bbac174. 10.1093/bib/bbac174. 35595541

[54] Morozov V, Rodrigues CHM, Ascher D B. CSM-toxin: a web-server for predicting protein toxicity. Pharmaceutics. 2023;15:431. 10.3390/pharmaceutics15020431. 36839752 PMC9966851

[55] Kaper J B, Nataro J P, Mobley H L. Pathogenic *Escherichia coli*. Nat Rev Micro. 2004;2:123–40. 10.1038/nrmicro818. 15040260

[56] Johnson T J, Nolan L K. Pathogenomics of the virulence plasmids of *Escherichia coli*. Microbiol Mol Biol Rev. 2009;73:750–74. 10.1128/MMBR.00015-09. 19946140 PMC2786578

[57] Henderson B, Martin A. Bacterial virulence in the moonlight: multitasking bacterial moonlighting proteins are virulence determinants in infectious disease. Infect Immun. 2011;79:3476–91. 10.1128/IAI.00179-11. 21646455 PMC3165470

[58] Henderson B, Martin A. Bacterial moonlighting proteins and bacterial virulence. Curr Top Microbiol Immunol. 2013;358:155–213. 10.1007/82_2011_188. 22143554

[59] Pancholi V, Fischetti V A. A major surface protein on group A streptococci is a glyceraldehyde-3-phosphate-dehydrogenase with multiple binding activity. J Exp Med. 1992;176:415–26. 10.1084/jem.176.2.415. 1500854 PMC2119316

[60] Barel M, Charbit A. Detection of the interaction between host and bacterial proteins: eukaryotic nucleolin interacts with *Francisella* elongation factor Tu. Methods Mol Biol. 2014;1197:123–39. 10.1007/978-1-4939-1261-2_7. 25172278

[61] Granato D, Bergonzelli G E, Pridmore R D et al. Cell surface-associated elongation factor tu mediates the attachment of *Lactobacillus johnsonii* NCC533 (La1) to human intestinal cells and mucins. Infect Immun. 2004;72:2160–69. 10.1128/iai.72.4.2160-2169.2004. 15039339 PMC375183

[62] Kamiya S, Yamaguchi H, Osaki T et al. A virulence factor of *Helicobacter pylori*: role of heat shock protein in mucosal inflammation after *H. pylori* infection. J Clin Gastroenterol. 1998;27:S35–S39. 10.1097/00004836-199800001-00007. 9872496

[63] Hickey TBM, Ziltener H J, Speert D P et al. Mycobacterium tuberculosis employs Cpn60.2 as an adhesin that binds CD43 on the macrophage surface: *M. tuberculosis* Cpn60.2 mediates macrophage binding via CD43. Cell Microbiol. 2010;12:1634–47. 10.1111/j.1462-5822.2010.01496.x. 20633027

[64] Lehner T, Bergmeier L A, Wang Y et al. Heat shock proteins generate β-chemokines which function as innate adjuvants enhancing adaptive immunity. Eur J Immunol. 2000;30:594–603. 10.1002/1521-4141(200002)30:2≤594::AID-IMMU594≥3.0.CO;2-1. 10671216

[65] Zhang R, Ou H-Y, Zhang C-T. DEG: a database of essential genes. Nucleic Acids Res. 2004;32:271D–272. 10.1093/nar/gkh024. 14681410 PMC308758

[66] UP Consortium . UniProt: the universal protein knowledgebase in 2025. Nucleic Acids Res. 2025;53:D609–17. 10.1093/nar/gkae1010. 39552041 PMC11701636

[67] Eddy S R . Profile hidden Markov models. Bioinformatics. 1998;14:755–63. 10.1093/bioinformatics/14.9.755. 9918945

[68] Larralde M, Zeller G. PyHMMER: a Python library binding to HMMER for efficient sequence analysis. Bioinformatics. 2023;39:btad214. 10.1093/bioinformatics/btad214. 37074928 PMC10159651

[69] Varrette S, Cartiaux H, Peter S et al. Management of an academic HPC & research computing facility: the ULHPC experience 2.0. In: Proceedings of the 2022 6th High Performance Computing and Cluster Technologies Conference. New York, NY: ACM, 2022.

