# Supplementary material for: PathoFact 2.0: an integrative pipeline for the prediction of antimicrobial resistance genes, virulence factors, toxins and toxin-associated proteins, and biosynthetic gene clusters in metagenomes
Source: Gigascience. 2026 May 22;15:giag062. doi: 10.1093/gigascience/giag062 (PMC13224393; doi:10.1093/gigascience/giag062)
Supplement: giag062_Supplemental_Files [file giag062_supplemental_files.zip › FigureS1_supplementary_material.pdf]

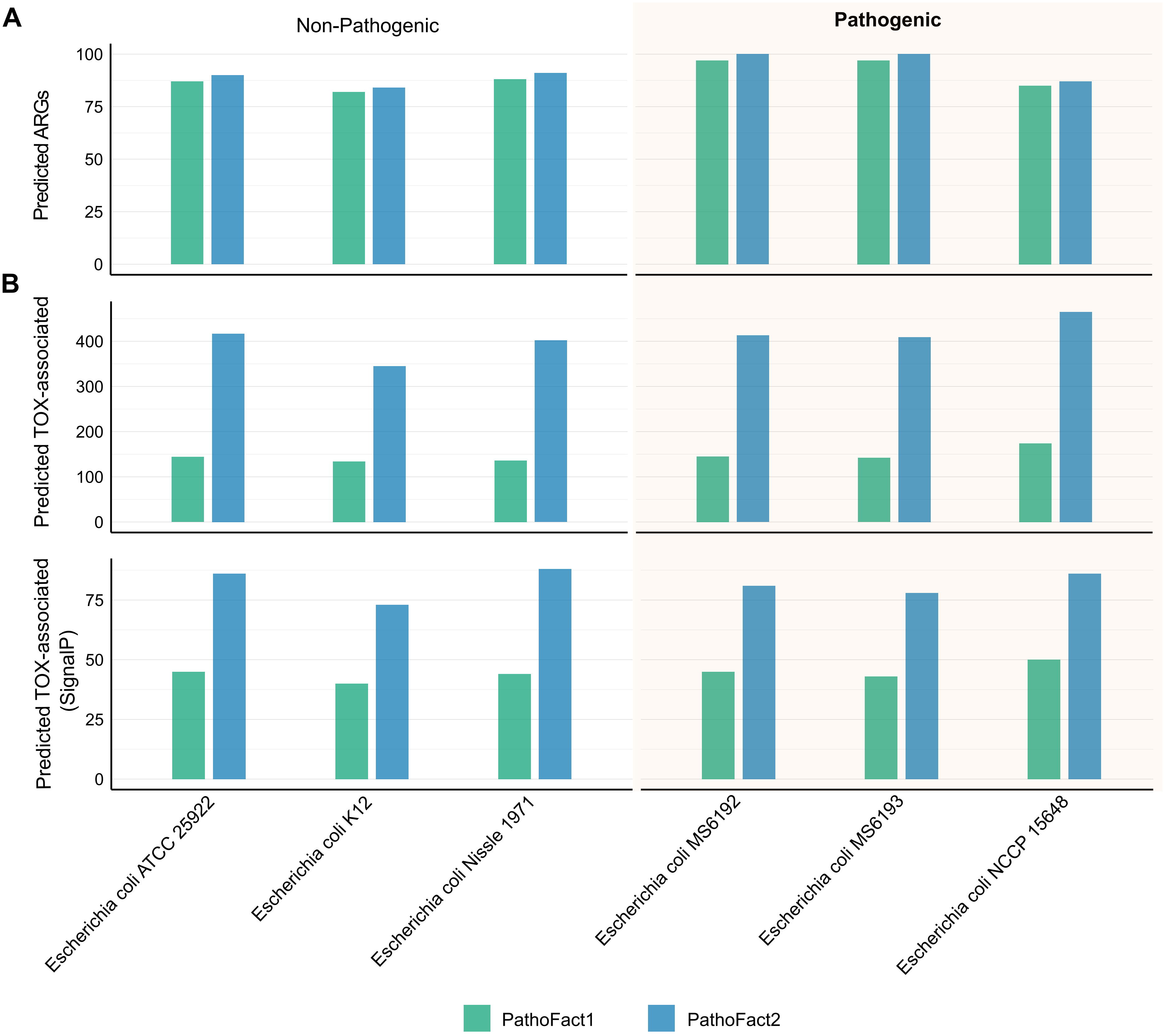

A
B
Non-Pathogenic
Pathogenic
Predicted ARGs
Predicted TOX-associated
Predicted TOX-associated (SignalP)
100
75
50
25
0
400
300
200
100
Escherichia coli ATCC 25922
Escherichia coli K12
Escherichia coli Nissle 1971
Escherichia coli MS6192
Escherichia coli MS6193
Escherichia coli NCCP 15648
PathoFact1
PathoFact2
