# Supplementary material for: PathoFact 2.0: an integrative pipeline for the prediction of antimicrobial resistance genes, virulence factors, toxins and toxin-associated proteins, and biosynthetic gene clusters in metagenomes
Source: Gigascience. 2026 May 22;15:giag062. doi: 10.1093/gigascience/giag062 (PMC13224393; doi:10.1093/gigascience/giag062)
Supplement: giag062_Supplemental_Files [file giag062_supplemental_files.zip › FigureS5_supplementary_material.pdf]

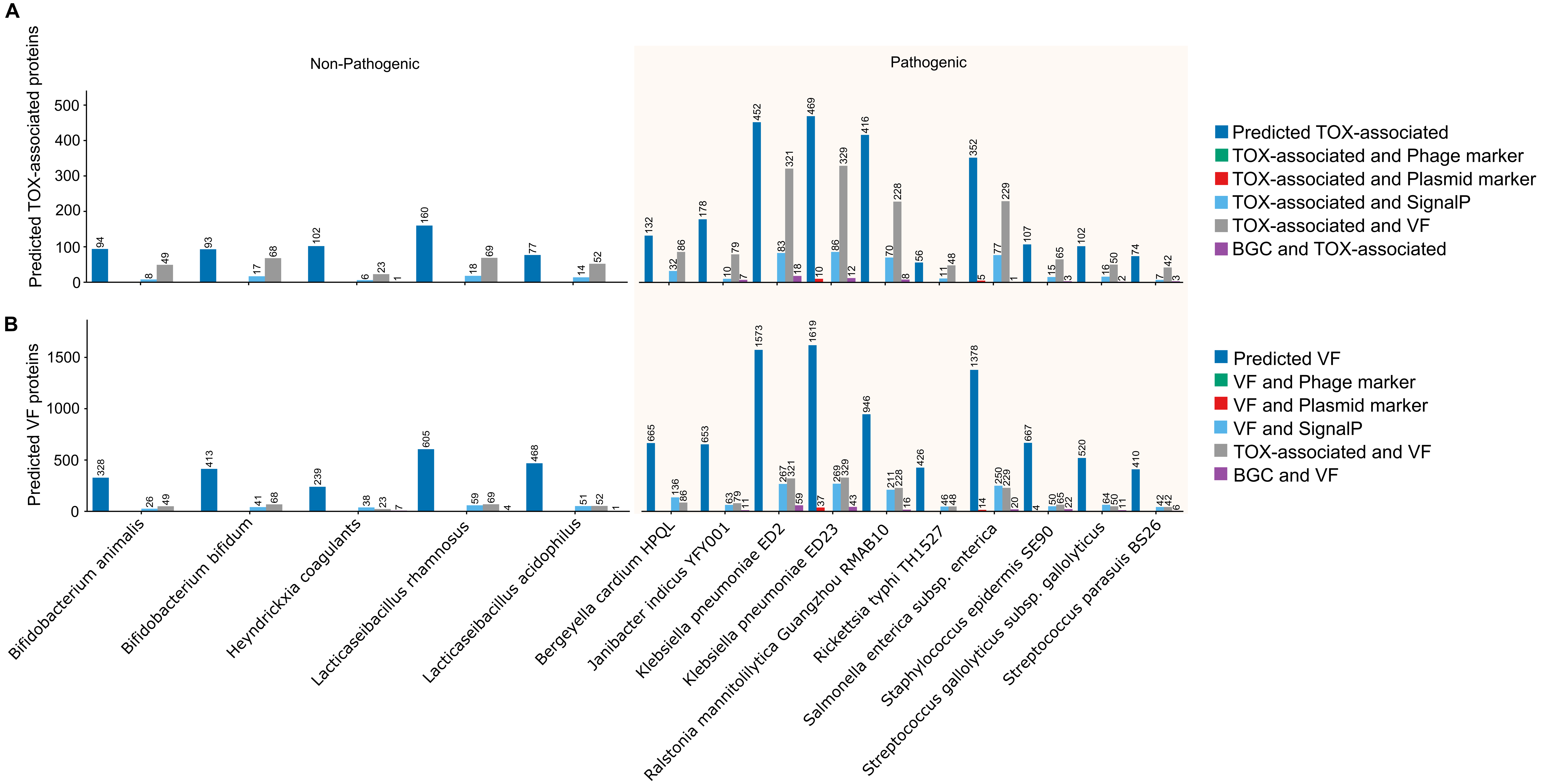

A
Non-Pathogenic
Pathogenic
Predicted TOX-associated proteins
Predicted TOX-associated
TOX-associated and Phage marker
TOX-associated and Plasmid marker
TOX-associated and SignalP
TOX-associated and VF
BGC and TOX-associated
B
Predicted VF proteins
Predicted VF
VF and Phage marker
VF and Plasmid marker
VF and SignalP
TOX-associated and VF
BGC and VF
Bifidobacterium animalis
Bifidobacterium bifidum
Heyndrickxia coagulants
Lacticaseibacillus rhamnosus
Lacticaseibacillus acidophilus
Bergeyella cardium HPQL
Janibacter indicus YFY001
Klebsiella pneumoniae ED2
Klebsiella pneumoniae ED23
Ralstonia mannitolilytica Guangzhou RMAB10
Rickettsia typhi TH1527
Salmonella enterica subsp. enterica
Staphylococcus epidermis SE90
Streptococcus gallolyticus subsp. gallolyticus
Streptococcus parasuis BS26
