# Supplementary material for: PathoFact 2.0: an integrative pipeline for the prediction of antimicrobial resistance genes, virulence factors, toxins and toxin-associated proteins, and biosynthetic gene clusters in metagenomes
Source: Gigascience. 2026 May 22;15:giag062. doi: 10.1093/gigascience/giag062 (PMC13224393; doi:10.1093/gigascience/giag062)
Supplement: giag062_Supplemental_Files [file giag062_supplemental_files.zip › TableS1_supplementary_material.pdf]

Supplementary Table S1.

List of microorganisms non-pathogenic to humans and their total protein count obtained from the NCBI Database.

Species	Number of proteins
Abelson murine leukemia virus	1
Agrobacterium tumefaciens	5,469
Bacillus atrophaeus	4,026
Bacillus badius	8,06
Bacillus licheniformis	8,473
Bacillus megaterium	5,674
Bacillus mojavensis	3,959
Bacillus mycoides	5,447
Border disease virus	1
Brevundimonas vesicularis	3,35
Canine distemper virus	7
Canine parvovirus	2
Carnobacterium divergens	2,421
Clostridium butyricum	8,237
Clostridium tertium	7,061
Collinsella intestinalis	1,522
Corynebacterium accolens	2,208
Corynebacterium macginleyi	4,376
Corynebacterium propinquum	2,124
Corynebacterium tuberculostearicum	2,219
Corynebacterium urealyticum	1,97
Dermacoccus nishinomiyaensis	2,748
Enterococcus avium	4,335
Enterococcus durans	2,67
Equine infectious anemia virus	4
Flavobacterium columnare	2,705
Flavobacterium psychrophilum	2,421
Haemophilus parasuis	2,126
Ictalurid herpesvirus 1	90
Infectious bursal disease virus	6
Kocuria rosea	3,565
Lactococcus garvieae	1,981
Listeria innocua	5,622
Listeria welshimeri	2,737
Minute virus of mice	6
Moraxella bovis	2,724
Mycoplasma gallisepticum	731
Mycoplasma hyorhinis	687
Obesumbacterium proteus	4,182
Penicillium expansum	11,075
Penicillium verrucosum	11,555
Porcine parvovirus	6
Porphyromonas endodontalis	3,376
Porphyromonas gulae	2,005
Pseudomonas alcaligenes	4,076
Pseudomonas fluorescens	5,823
Pseudomonas syringae	5,056
Saccharomyces carlsbergensis	13
Saccharomyces pastorianus	13
Shewanella putrefaciens	7,61
Staphylococcus carnosus	4,827
Streptomyces albus	6,528
Tetragenococcus halophilus	2,203
Vagococcus fluvialis	4,078
Yersinia ruckeri	3,334
Zygosaccharomyces rouxii	4,991
TOTAL	192,516
