# Supplementary material for: PathoFact 2.0: an integrative pipeline for the prediction of antimicrobial resistance genes, virulence factors, toxins and toxin-associated proteins, and biosynthetic gene clusters in metagenomes
Source: Gigascience. 2026 May 22;15:giag062. doi: 10.1093/gigascience/giag062 (PMC13224393; doi:10.1093/gigascience/giag062)
Supplement: giag062_Supplemental_Files [file giag062_supplemental_files.zip › TableS2_supplementary_material.pdf]

Supplementary Table S2. Bacterial strains used in this study, including their classification as pathogenic or non-pathogenic, species/strain information, genome assembly or reference version, and corresponding accession numbers.

Bacteria Group	Species / Strain	Genome Assembly / Reference	Accession Number
Escherichia coli non-pathogenic	K12 substrain MG1655	Reference genome ASM584v2	GCF_000005845.2
Escherichia coli non-pathogenic	ATCC 25922	Genome assembly ASM74325v1	GCF_000743255.1
Escherichia coli non-pathogenic	Nissle 1917	Genome assembly ASM2155983v1	GCF_021559835.1
Escherichia coli pathogenic	NCCP15648	Genome assembly ASM343327v1	GCA_003433275.1
Escherichia coli pathogenic	MS6193	ASM1331708v1	GCA_013317085.1
Escherichia coli pathogenic	MS6192	ASM1335734v1	GCA_013357345.1
Other Bacteria non-pathogenic	*Lacticaseibacillus rhamnosus*	Reference genome ASM615190v1	GCF_006151905.1
Other Bacteria non-pathogenic	*Bifidobacterium animalis subsp. lactis DSM 10140*	Reference genome ASM2296v1	GCF_000022965.1
Other Bacteria non-pathogenic	*Lactobacillus acidophilus*	Reference genome ASM3429813v1	GCF_034298135.1
Other Bacteria non-pathogenic	*Bifidobacterium bifidum CNCM I-4319*	Reference genome ASM1789432v1	GCF_017894325.1
Other Bacteria non-pathogenic	*Heyndrickxia coagulans DSM 1 = ATCC 7050*	Reference genome ASM83290v1	GCF_000832905.1
Other Bacteria pathogenic	*Rickettsia typhi str. TH1527*	Genome assembly ASM27728v1	GCF_000277285.1
Other Bacteria pathogenic	*Janibacter indicus*	Genome assembly ASM188912v1	GCF_001889125.1
Other Bacteria pathogenic	*Ralstonia mannitolilytica*	Genome assembly ASM1104451v1	GCA_011044515.1
Other Bacteria pathogenic	*Staphylococcus epidermidis*	Genome assembly ASM274945v1	GCA_002749455.1
Other Bacteria pathogenic	*Salmonella enterica subsp. enterica serovar Enteritidis*	Genome assembly ASM61232v1	GCF_000612325.1
Other Bacteria pathogenic	*Streptococcus parasuis*	Genome assembly ASM1907688v1	GCA_019076885.1
Other Bacteria pathogenic	*Bergeyella cardium*	Reference genome ASM991466v1	GCA_009914665.1
Other Bacteria pathogenic	*Streptococcus gallolyticus subsp. gallolyticus TX20005*	Genome assembly ASM1902180v1	GCA_019021805.1
Other Bacteria pathogenic	*Klebsiella pneumoniae ED2*	Genome assembly ASM170824v1	GCF_001708245.1
Other Bacteria pathogenic	*Klebsiella pneumoniae ED23*	ASM170822v1	GCF_001708225.1
