# Supplementary material for: PathoFact 2.0: an integrative pipeline for the prediction of antimicrobial resistance genes, virulence factors, toxins and toxin-associated proteins, and biosynthetic gene clusters in metagenomes
Source: Gigascience. 2026 May 22;15:giag062. doi: 10.1093/gigascience/giag062 (PMC13224393; doi:10.1093/gigascience/giag062)
Supplement: giag062_Supplemental_Files [file giag062_supplemental_files.zip › TableS3_supplementary_material.pdf]

Supplementary Table S3. Evaluation of the PathoFact 2.0 toxin-associated protein prediction module. The table presents performance across test subsets defined by sequence similarity to the training set. Metrics reported include class distributions (Negative, non-toxin; Positive, toxin-associated), confusion matrix counts (true negatives, false positives, true positives, false negatives), and performance measures (accuracy, precision, recall, F1 score, Matthews correlation coefficient).

Method	Sub_Test_dataset [%AAI_cov_80%]	Number of Proteins						Accuracy	Precision	Recall	F1 score	MCC
		gative (non-Toxin)	Positive (Toxin-associated)	True Negative	False Positive	True Positive	False Negative					
**PathoFact2_cutoff_0.5**	40	6316	3001	6209	107	2793	208	0.966	0.963	0.931	0.947	0.922
**PathoFact2_cutoff_0.6**	40	6316	3001	6267	49	2703	298	0.963	0.982	0.901	0.94	0.915
**PathoFact2_cutoff_0.8**	40	6316	3001	6303	13	1965	1036	0.887	0.993	0.655	0.789	0.746
**PathoFact2_cutoff_0.9**	40	6316	3001	6308	8	1201	1800	0.806	0.993	0.4	0.571	0.555
**PathoFact2_cutoff_0.5**	60	13467	6163	13207	260	5875	288	0.972	0.958	0.953	0.955	0.935
**PathoFact2_cutoff_0.6**	60	13467	6163	13353	114	5727	436	0.972	0.98	0.929	0.954	0.935
**PathoFact2_cutoff_0.8**	60	13467	6163	13430	37	4474	1689	0.912	0.992	0.726	0.838	0.798
**PathoFact2_cutoff_0.9**	60	13467	6163	13444	23	2978	3185	0.837	0.992	0.483	0.65	0.621
**PathoFact2_cutoff_0.5**	80	26452	11613	26057	395	11251	362	0.98	0.966	0.969	0.967	0.953
**PathoFact2_cutoff_0.6**	80	26452	11613	26280	172	11049	564	0.981	0.985	0.951	0.968	0.954
**PathoFact2_cutoff_0.8**	80	26452	11613	26395	57	9362	2251	0.939	0.994	0.806	0.89	0.858
**PathoFact2_cutoff_0.9**	80	26452	11613	26414	38	6997	4616	0.878	0.995	0.603	0.75	0.713
**PathoFact2_cutoff_0.5**	100	42703	42538	42200	503	42126	412	0.989	0.988	0.99	0.989	0.979
**PathoFact2_cutoff_0.6**	100	42703	42538	42477	226	41876	662	0.99	0.995	0.984	0.99	0.979
**PathoFact2_cutoff_0.8**	100	42703	42538	42614	89	39836	2702	0.967	0.998	0.936	0.966	0.936
**PathoFact2_cutoff_0.9**	100	42703	42538	42644	59	36550	5988	0.929	0.998	0.859	0.924	0.867
**PathoFact2_cutoff_0.5**	All	42725	42574	42208	517	42162	412	0.989	0.988	0.99	0.989	0.978
**PathoFact2_cutoff_0.6**	All	42725	42574	42495	230	41912	662	0.99	0.995	0.984	0.989	0.979
**PathoFact2_cutoff_0.8**	All	42725	42574	42633	92	39871	2703	0.967	0.998	0.937	0.966	0.936
**PathoFact2_cutoff_0.9**	All	42725	42574	42663	62	36585	5989	0.929	0.998	0.859	0.924	0.866
