# Supplementary material for: PathoFact 2.0: an integrative pipeline for the prediction of antimicrobial resistance genes, virulence factors, toxins and toxin-associated proteins, and biosynthetic gene clusters in metagenomes
Source: Gigascience. 2026 May 22;15:giag062. doi: 10.1093/gigascience/giag062 (PMC13224393; doi:10.1093/gigascience/giag062)
Supplement: giag062_Supplemental_Files [file giag062_supplemental_files.zip › TableS4_supplementary_material.pdf]

Supplementary Table S4. Evaluation of the PathoFact 2.0 virulence factor prediction module. The table summarises performance across test subsets defined by sequence similarity to the training set. Reported metrics include class distributions (Negative, non-VF; Positive, VF), confusion matrix counts, and performance measures (accuracy, precision, recall, F1 score, Matthews correlation coefficient).

Method	Sub_Test_dataset [%AAI_cov_80%]	Number of Proteins						Accuracy	Precision	Recall	F1 score	MCC
		ative (non-VF)	itive (VF)	True Negative	False Positive	True Positive	False Negative					
**PathoFact2_cutoff_0.5**	40	3904	336	3874	30	321	15	0.989	0.915	0.955	0.934	0.929
**PathoFact2_cutoff_0.6**	40	3904	336	3879	25	321	15	0.991	0.928	0.955	0.941	0.936
**PathoFact2_cutoff_0.8**	40	3904	336	3888	16	317	19	0.992	0.952	0.943	0.948	0.943
**PathoFact2_cutoff_0.9**	40	3904	336	3892	12	315	21	0.992	0.963	0.938	0.950	0.946
**PathoFact2_cutoff_0.5**	60	5617	448	5576	41	432	16	0.991	0.913	0.964	0.938	0.933
**PathoFact2_cutoff_0.6**	60	5617	448	5587	30	432	16	0.992	0.935	0.964	0.949	0.945
**PathoFact2_cutoff_0.8**	60	5617	448	5598	19	428	20	0.994	0.957	0.955	0.956	0.953
**PathoFact2_cutoff_0.9**	60	5617	448	5602	15	425	23	0.994	0.966	0.949	0.957	0.954
**PathoFact2_cutoff_0.5**	80	8082	633	8037	45	611	22	0.992	0.931	0.965	0.948	0.944
**PathoFact2_cutoff_0.6**	80	8082	633	8049	33	609	24	0.993	0.949	0.962	0.955	0.952
**PathoFact2_cutoff_0.8**	80	8082	633	8061	21	605	28	0.994	0.966	0.956	0.961	0.958
**PathoFact2_cutoff_0.9**	80	8082	633	8065	17	601	32	0.994	0.972	0.949	0.961	0.958
**PathoFact2_cutoff_0.5**	100	8106	4171	8061	45	4144	27	0.994	0.989	0.994	0.991	0.987
**PathoFact2_cutoff_0.6**	100	8106	4171	8073	33	4138	33	0.995	0.992	0.992	0.992	0.988
**PathoFact2_cutoff_0.8**	100	8106	4171	8085	21	4127	44	0.995	0.995	0.989	0.992	0.988
**PathoFact2_cutoff_0.9**	100	8106	4171	8089	17	4115	56	0.994	0.996	0.987	0.991	0.987
**PathoFact2_cutoff_0.5**	All	8358	6137	8310	48	6073	64	0.992	0.992	0.990	0.991	0.984
**PathoFact2_cutoff_0.6**	All	8358	6137	8323	35	6062	75	0.992	0.994	0.988	0.991	0.984
**PathoFact2_cutoff_0.8**	All	8358	6137	8336	22	6035	102	0.991	0.996	0.983	0.990	0.983
**PathoFact2_cutoff_0.9**	All	8358	6137	8341	17	6010	127	0.990	0.997	0.979	0.988	0.980
