# Supplementary material for: PathoFact 2.0: an integrative pipeline for the prediction of antimicrobial resistance genes, virulence factors, toxins and toxin-associated proteins, and biosynthetic gene clusters in metagenomes
Source: Gigascience. 2026 May 22;15:giag062. doi: 10.1093/gigascience/giag062 (PMC13224393; doi:10.1093/gigascience/giag062)
Supplement: giag062_Supplemental_Files [file giag062_supplemental_files.zip › TableS5_supplementary_material.pdf]

Supplementary Table S5. Comparison of virulence factor prediction performance for PathoFact 2.0 at varying probability cutoffs and for VirulenHunter, evaluated across test subsets stratified by sequence similarity to the training set. The table reports class distributions, confusion matrix counts, and performance metrics (accuracy, precision, recall, F1 score, Matthews correlation coefficient)

Method	Sub_Test_dataset [%AAI_cov_80%]	Number of Proteins						Accuracy	Precision	Recall	F1 score	MCC
		ative (non-VF)	tive (VF)	True Negative	False Positive	True Positive	False Negative					
**MetaVF**	40	3904	336	3904	0	12	324	0.924	1.000	0.036	0.069	0.182
**PathoFact1**	40	3904	336	3904	0	103	233	NA	NA	0.307	NA	NA
**VirulentHunter**	40	3904	336	3316	588	159	177	0.820	0.213	0.473	0.294	0.229
**PathoFact2_cutoff_0.5**	40	3904	336	3874	30	321	15	0.989	0.915	0.955	0.934	0.929
**PathoFact2_cutoff_0.6**	40	3904	336	3879	25	321	15	0.991	0.928	0.955	0.941	0.936
**PathoFact2_cutoff_0.8**	40	3904	336	3888	16	317	19	0.992	0.952	0.943	0.948	0.943
**PathoFact2_cutoff_0.9**	40	3904	336	3892	12	315	21	0.992	0.963	0.938	0.950	0.946
**MetaVF**	60	5617	448	5617	0	15	433	0.929	1.000	0.033	0.065	0.176
**PathoFact1**	60	5617	448	5617	0	155	293	NA	NA	0.346	NA	NA
**VirulentHunter**	60	5617	448	4921	696	234	214	0.850	0.252	0.522	0.340	0.289
**PathoFact2_cutoff_0.5**	60	5617	448	5576	41	432	16	0.991	0.913	0.964	0.938	0.933
**PathoFact2_cutoff_0.6**	60	5617	448	5587	30	432	16	0.992	0.935	0.964	0.949	0.945
**PathoFact2_cutoff_0.8**	60	5617	448	5598	19	428	20	0.994	0.957	0.955	0.956	0.953
**PathoFact2_cutoff_0.9**	60	5617	448	5602	15	425	23	0.994	0.966	0.949	0.957	0.954
**MetaVF**	80	8082	633	8081	1	22	611	0.930	0.957	0.035	0.067	0.175
**PathoFact1**	80	8082	633	8082	0	241	392	NA	NA	0.381	NA	NA
**VirulentHunter**	80	8082	633	7270	812	375	258	0.877	0.316	0.592	0.412	0.372
**PathoFact2_cutoff_0.5**	80	8082	633	8037	45	611	22	0.992	0.931	0.965	0.948	0.944
**PathoFact2_cutoff_0.6**	80	8082	633	8049	33	609	24	0.993	0.949	0.962	0.955	0.952
**PathoFact2_cutoff_0.8**	80	8082	633	8061	21	605	28	0.994	0.966	0.956	0.961	0.958
**PathoFact2_cutoff_0.9**	80	8082	633	8065	17	601	32	0.994	0.972	0.949	0.961	0.958
**MetaVF**	100	8106	4171	8105	1	462	3709	0.698	0.998	0.111	0.199	0.275
**PathoFact1**	100	8106	4171	8106	0	2132	2039	NA	NA	0.511	NA	NA
**VirulentHunter**	100	8106	4171	7292	814	3397	774	0.871	0.807	0.814	0.811	0.712
**PathoFact2_cutoff_0.5**	100	8106	4171	8061	45	4144	27	0.994	0.989	0.994	0.991	0.987
**PathoFact2_cutoff_0.6**	100	8106	4171	8073	33	4138	33	0.995	0.992	0.992	0.992	0.988
**PathoFact2_cutoff_0.8**	100	8106	4171	8085	21	4127	44	0.995	0.995	0.989	0.992	0.988
**PathoFact2_cutoff_0.9**	100	8106	4171	8089	17	4115	56	0.994	0.996	0.987	0.991	0.987
