# Supplementary material for: PathoFact 2.0: an integrative pipeline for the prediction of antimicrobial resistance genes, virulence factors, toxins and toxin-associated proteins, and biosynthetic gene clusters in metagenomes
Source: Gigascience. 2026 May 22;15:giag062. doi: 10.1093/gigascience/giag062 (PMC13224393; doi:10.1093/gigascience/giag062)
Supplement: giag062_Supplemental_Files [file giag062_supplemental_files.zip › TableS6_supplementary_material.pdf]

Supplementary Table S6. Comparison of toxin protein prediction performance for PathoFact 2.0 at different prediction probability cutoffs and for ToxinPred2. The table reports the number of proteins classified as Negative (non-toxin) and Positive (toxin), confusion matrix counts, and associated performance metrics (accuracy, precision, recall, F1 score, and Matthews correlation coefficient).

Method	Number of Proteins						Accuracy	Precision	Recall	F1 score	MCC
	legative (non-Toxin)	itive (Toxin)	True Negative	False Positive	True Positive	False Negative					
**PathoFact2_cutoff_0.5**	1000	1000	986	14	949	51	0.968	0.985	0.949	0.967	0.936
**PathoFact2_cutoff_0.6**	1000	1000	997	3	931	69	0.964	0.997	0.931	0.963	0.930
**PathoFact2_cutoff_0.8**	1000	1000	999	1	765	235	0.882	0.999	0.765	0.866	0.786
**ToxinPred2**	1000	1000	954	46	486	514	0.720	0.914	0.486	0.634	0.498
**PathoFact 1.0**	1000	1000	1000	0	287	713	NA	NA	0.287	NA	NA
**CSM-toxin**	1000	1000	998	2	108	892	0.553	0.982	0.108	0.195	0.232
