# Supplementary figures and images for: PathoFact 2.0: an integrative pipeline for the prediction of antimicrobial resistance genes, virulence factors, toxins and toxin-associated proteins, and biosynthetic gene clusters in metagenomes
Source: Gigascience. 2026 May 22;15:giag062. doi: 10.1093/gigascience/giag062 (PMC13224393; doi:10.1093/gigascience/giag062)

**A**

Non-Pathogenic

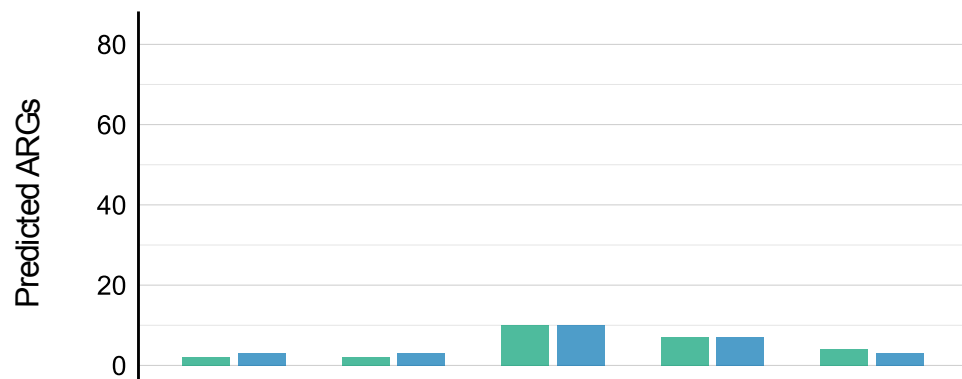

Pathogenic

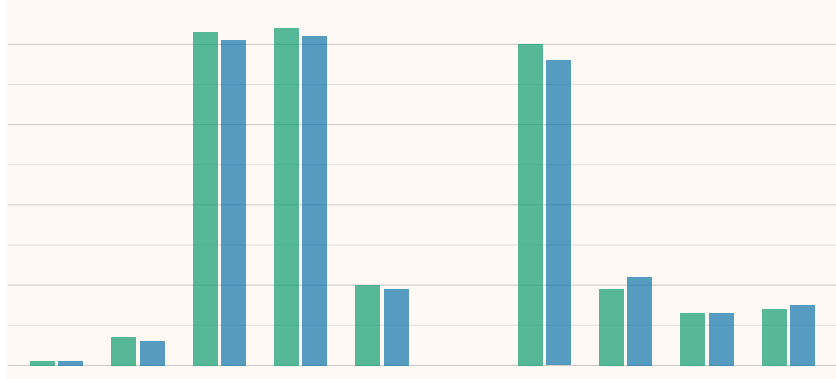**B**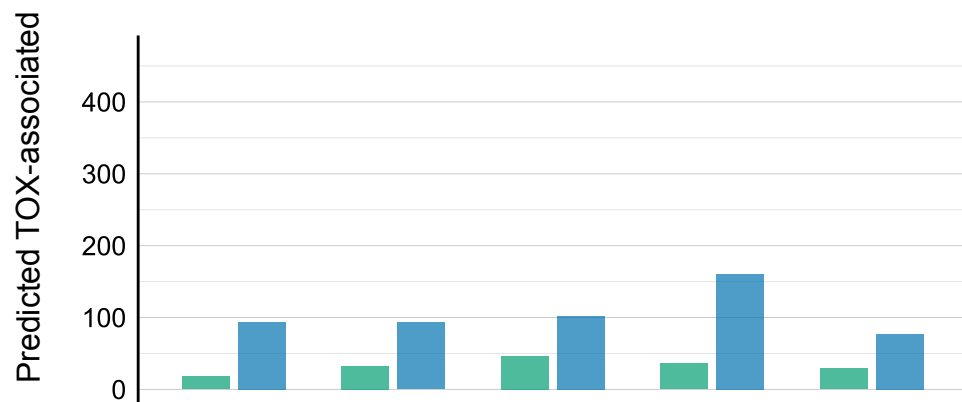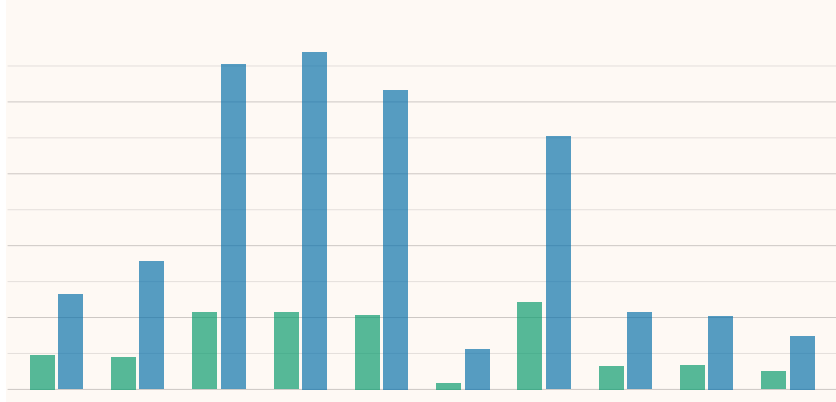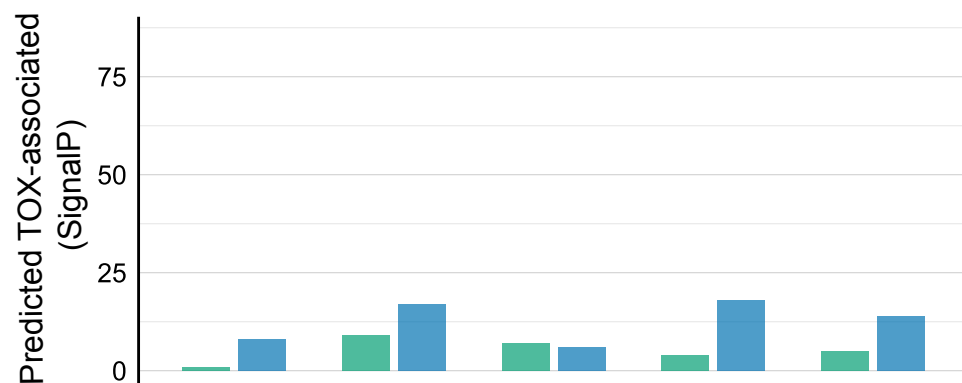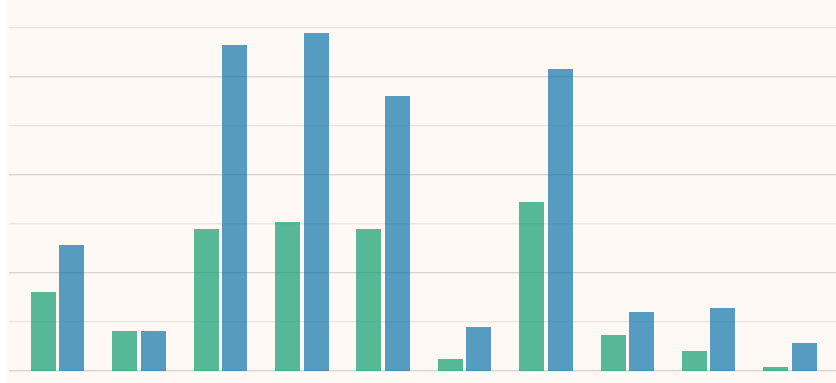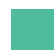

PathoFact1

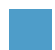

PathoFact2

Supplement: giag062_Supplemental_Files [file giag062_supplemental_files.zip › FigureS2_supplementary_material.pdf]

**A**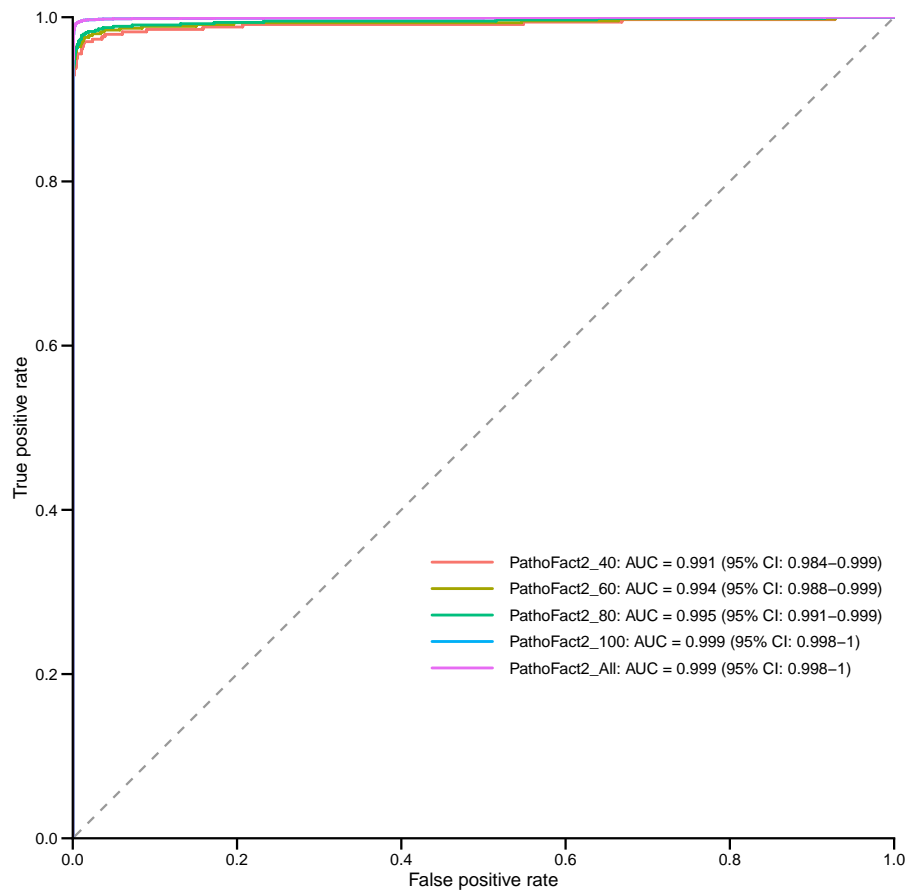**B**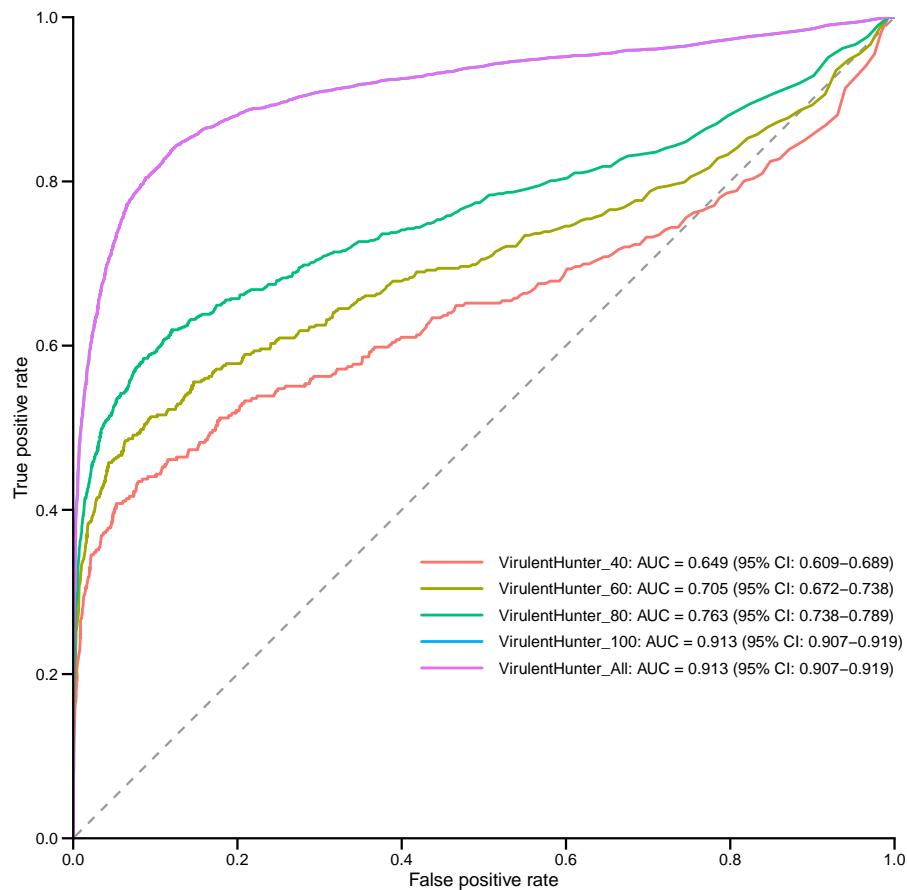

Supplement: giag062_Supplemental_Files [file giag062_supplemental_files.zip › FigureS3_supplementary_material.pdf]

**A**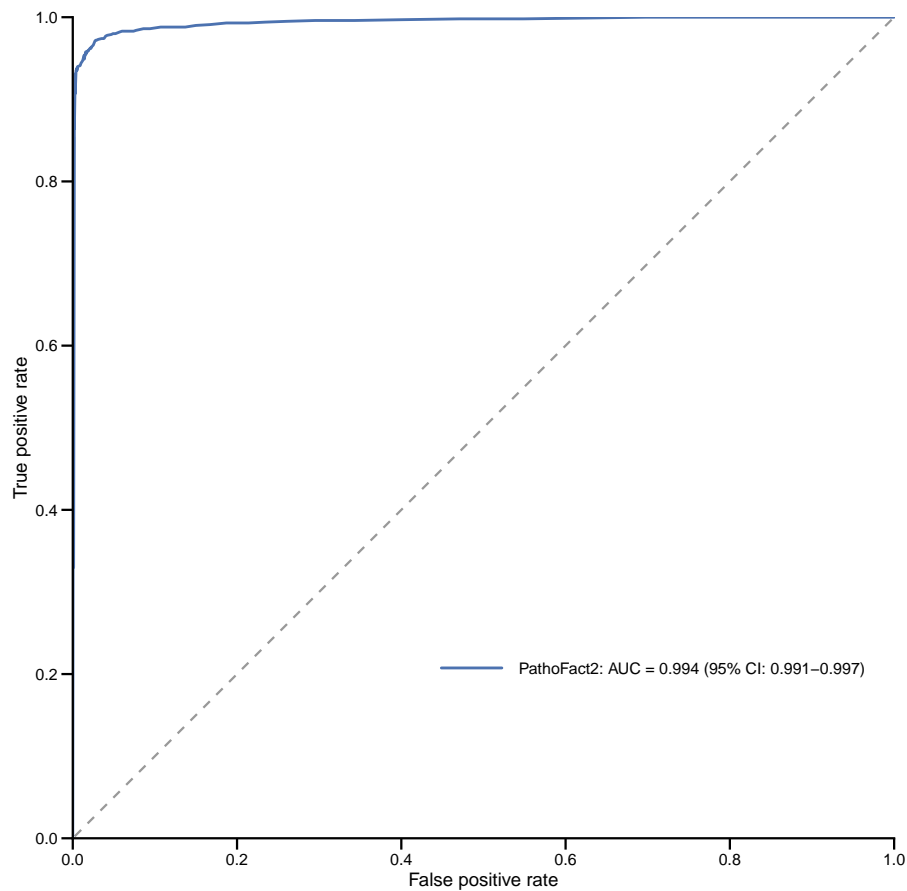**B**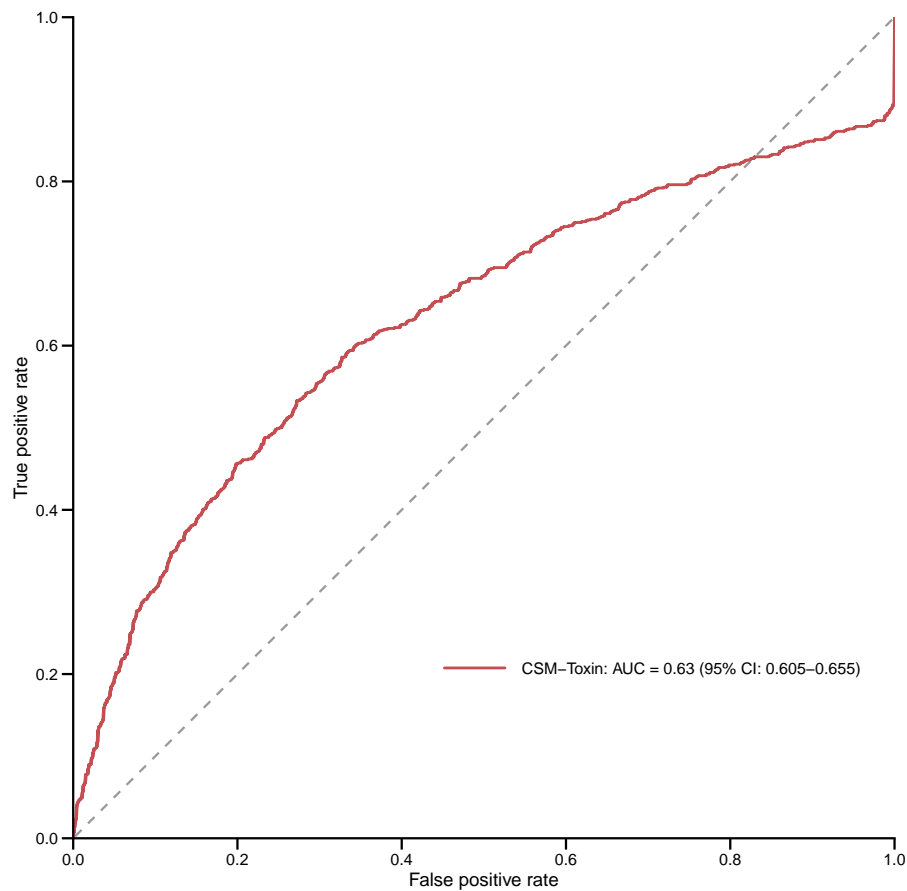

Supplement: giag062_Supplemental_Files [file giag062_supplemental_files.zip › FigureS4_supplementary_material.pdf]

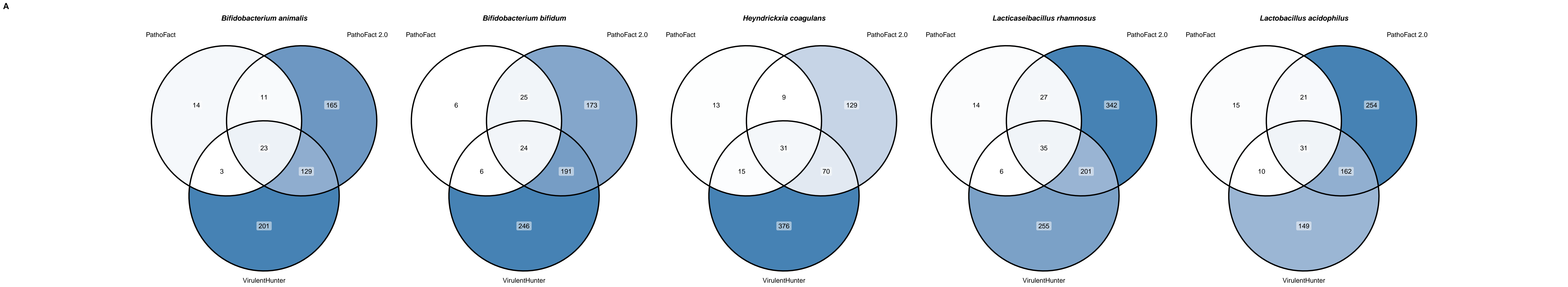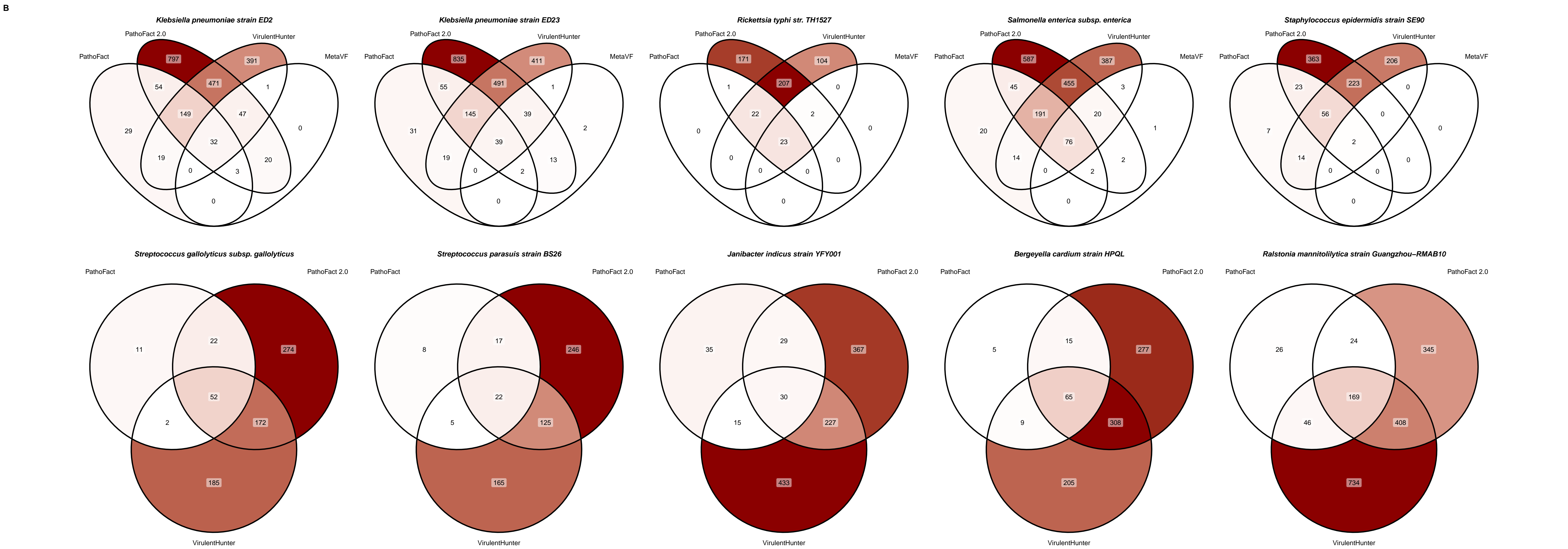

Supplement: giag062_Supplemental_Files [file giag062_supplemental_files.zip › FigureS6_supplementary_material.pdf]

Non-Pathogenic

Pathogenic

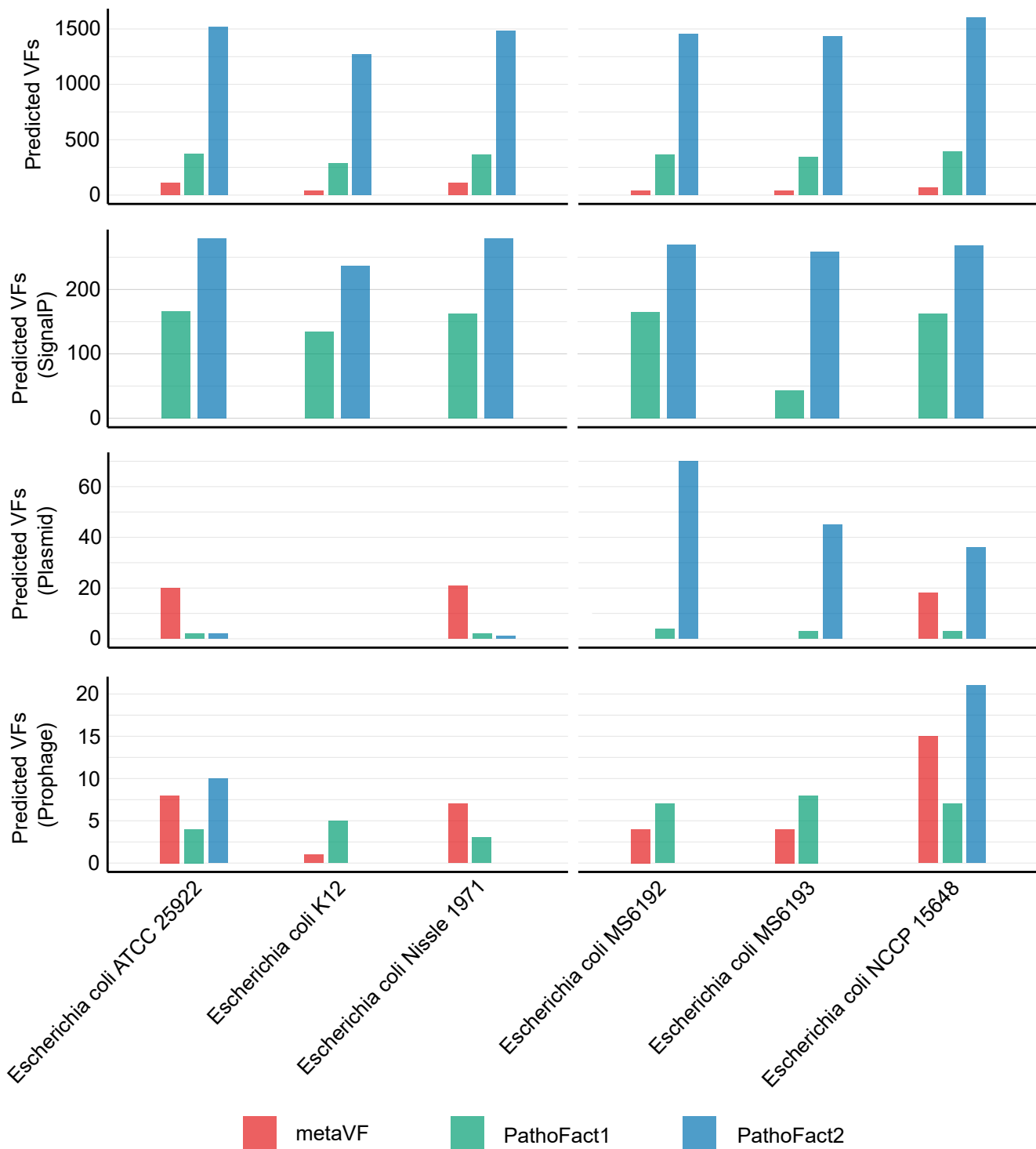

Supplement: giag062_Supplemental_Files [file giag062_supplemental_files.zip › FigureS7_supplementary_material.pdf]

Non-Pathogenic

Pathogenic

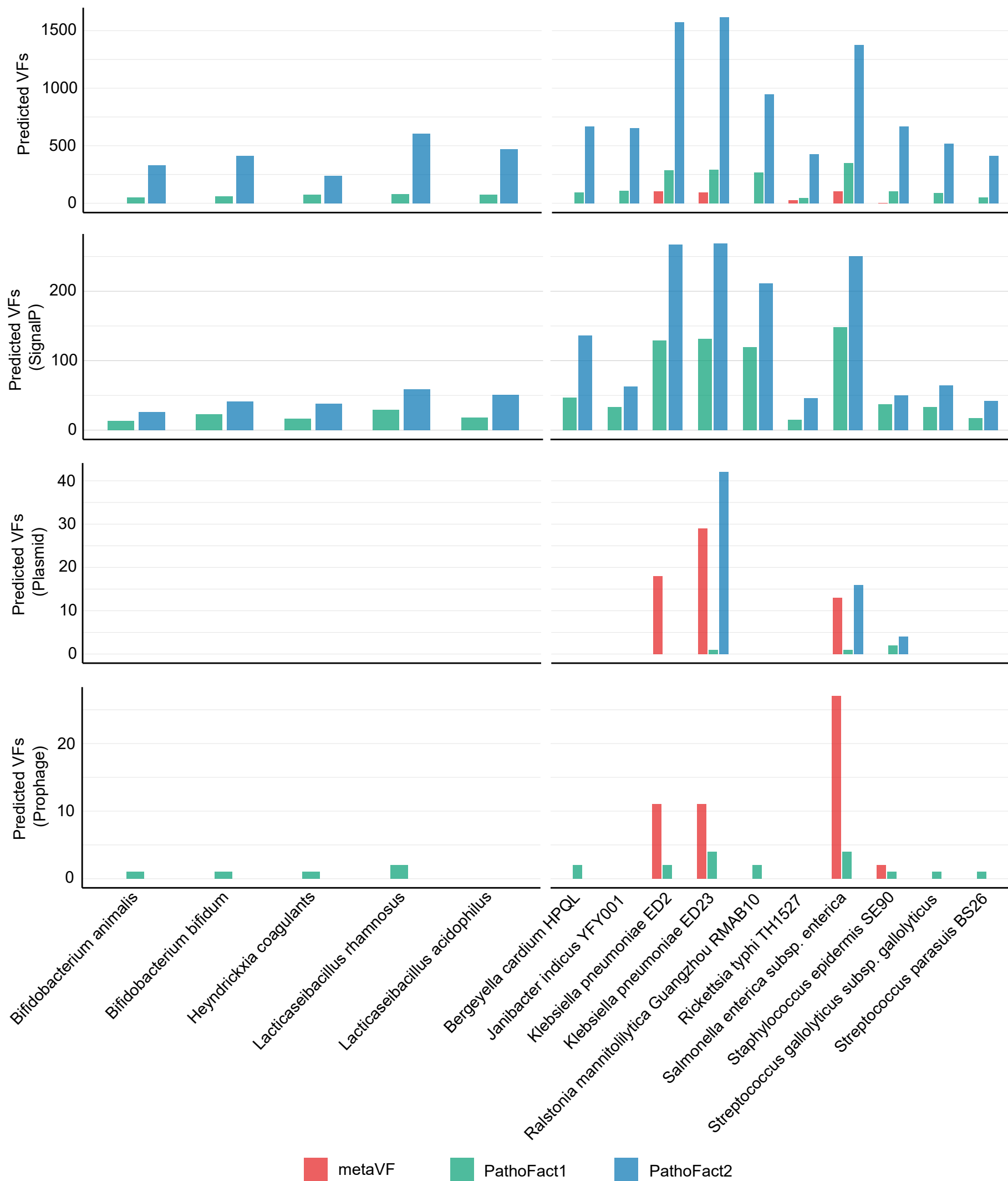

Supplement: giag062_Supplemental_Files [file giag062_supplemental_files.zip › FigureS8_supplementary_material.pdf]

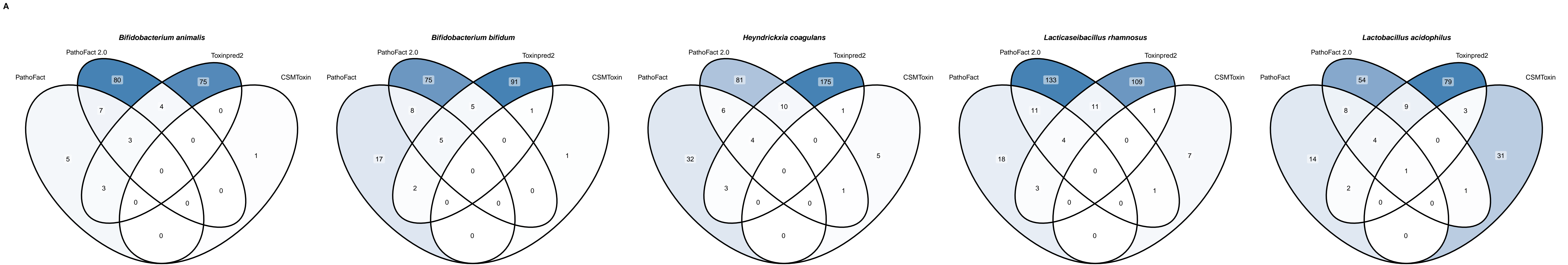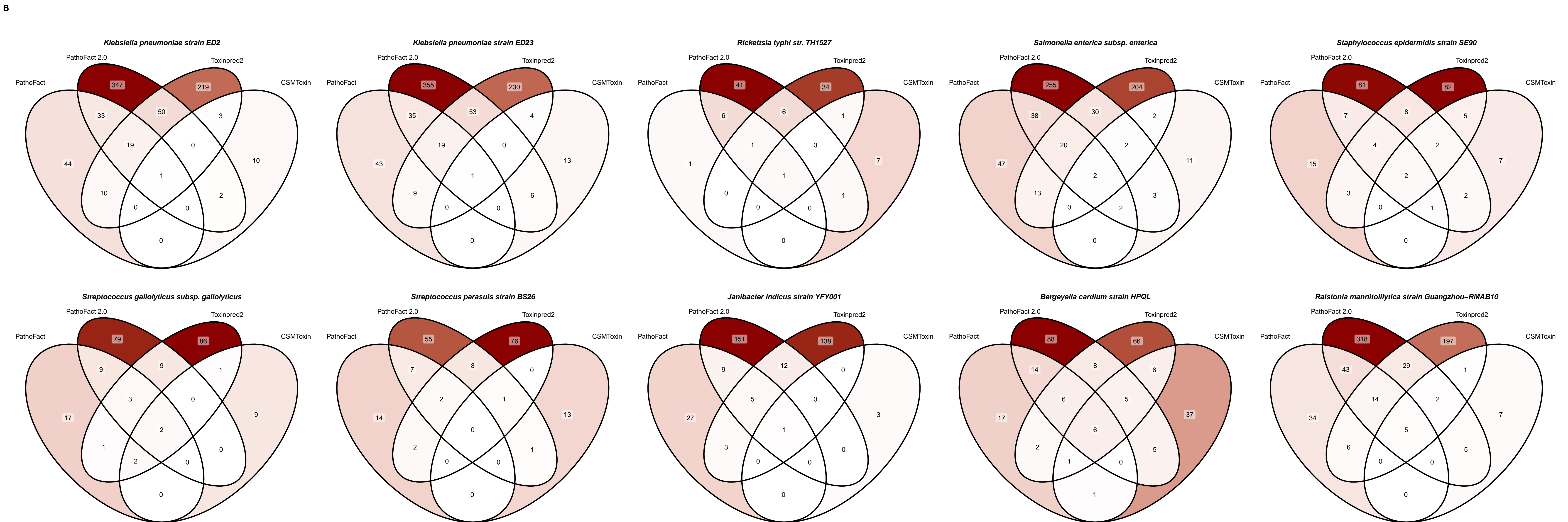

Supplement: giag062_Supplemental_Files [file giag062_supplemental_files.zip › FigureS9_supplementary_material.pdf]
