# Supplementary material for: PathoFact 2.0: an integrative pipeline for the prediction of antimicrobial resistance genes, virulence factors, toxins and toxin-associated proteins, and biosynthetic gene clusters in metagenomes
Source: Gigascience. 2026 May 22;15:giag062. doi: 10.1093/gigascience/giag062 (PMC13224393; doi:10.1093/gigascience/giag062)
Supplement: giag062_GIGA-D-25-00455_original_submission [file giag062_giga-d-25-00455_original_submission.pdf]

GigaScience

PathoFact 2.0: An Integrative Pipeline for Antimicrobial Resistance Genes, Virulence Factors, Toxins, and Biosynthetic Gene Clusters Prediction in Metagenomes

--Manuscript Draft--

Manuscript Number:	GIGA-D-25-00455
Full Title:	PathoFact 2.0: An Integrative Pipeline for Antimicrobial Resistance Genes, Virulence Factors, Toxins, and Biosynthetic Gene Clusters Prediction in Metagenomes
Article Type:	Technical Note
Funding Information:	Fondation du Pélican de Mie et Pierre Hippert-Faber (Pélican Grant) — Miss Júlia Ortís Sunyer<br>Fonds National de la Recherche Luxembourg (FNR CORE/23/BM/15886415) — Dr. Paul Wilmes<br>European Research Council (ERC-CoG 863664) — Dr. Paul Wilmes
Abstract:	Antimicrobial resistance (ARG) and virulence factors (VFs) are central contributors to the global health crisis surrounding drug-resistant infections.<br>We introduce PathoFact 2.0, an enhanced pipeline for improved ARG, VF, and toxin prediction. Key updates include an updated machine learning (ML) model for VF identification, expanded hidden Markov model profiles for VFs and toxin-related proteins, a new ML model for toxin (and toxin-associated proteins) identification, and the integration of antiSMASH 7.0 for predicting biosynthetic gene clusters.<br>Our upgrades make PathoFact 2.0 a more powerful and user-friendly platform for predicting microbiome-based pathogenicity and resistance, providing a crucial tool for better understanding and addressing the challenges posed by antimicrobial resistance and infectious diseases.<br>PathoFact 2.0 is available at https://gitlab.com/uniluxembourg/lcsb/systems-ecology/pathofact2. It is compatible with Linux operating systems.
Corresponding Author:	Luis Fernando Delgado, PhD<br>University of Luxembourg Luxembourg Centre for Systems Biomedicine: Universite du Luxembourg Luxembourg Centre for Systems Biomedicine<br>LUXEMBOURG
Corresponding Author Secondary Information:	
Corresponding Author's Institution:	University of Luxembourg Luxembourg Centre for Systems Biomedicine: Universite du Luxembourg Luxembourg Centre for Systems Biomedicine
Corresponding Author's Secondary Institution:	
First Author:	Luis Fernando Delgado, PhD
First Author Secondary Information:	
Order of Authors:	Luis Fernando Delgado, PhD<br>Júlia Ortís Sunyer, MSc<br>Cedric Christian Laczny, PhD<br>Oskar Hickl, PhD<br>Patrick May, PhD<br>Paul Wilmes, PhD
Order of Authors Secondary Information:	
Additional Information:	
**Question**	**Response**

Powered by Editorial Manager® and ProduXion Manager® from Aries Systems Corporation

Are you submitting this manuscript to a special series or article collection?	No
**Experimental design and statistics**<br><br>Full details of the experimental design and statistical methods used should be given in the Methods section, as detailed in our Minimum Standards Reporting Checklist. Information essential to interpreting the data presented should be made available in the figure legends.<br><br>Have you included all the information requested in your manuscript?	Yes
**Resources**<br><br>A description of all resources used, including antibodies, cell lines, animals and software tools, with enough information to allow them to be uniquely identified, should be included in the Methods section. Authors are strongly encouraged to cite Research Resource Identifiers (RRIDs) for antibodies, model organisms and tools, where possible.<br><br>Have you included the information requested as detailed in our Minimum Standards Reporting Checklist?	Yes
**Availability of data and materials**<br><br>All datasets and code on which the conclusions of the paper rely must be either included in your submission or deposited in publicly available repositories (where available and ethically appropriate), referencing such data using a unique identifier in the references and in the "Availability of Data and Materials" section of your manuscript.<br><br>Have you have met the above requirement as detailed in our Minimum	Yes

Standards Reporting Checklist?	
GigaScience has policies and guidelines in place for the use of generative AI-writing tools such as ChatGPT. If you have used such writing tools to assist with writing the manuscript this must be declared and cited in the text. Authors should not list AI-writing tools and other AI-assisted technologies as an author or co-author and should acknowledge that they are fully responsible for text generated or refined by AI-writing tools.<p><br><br>A summary of use (particularly in the introduction or among methods) needs to be included at the end of the paper, and the outputs should also be included as a supplementary file hosted in GigaDB or other open repositories. Please read our guidelines for more information. <p><br><br>By submitting to GigaScience, you are aware of the journal's AI-writing tools policy, and if you have declared use of such tools below, you have acknowledged this where appropriate in your manuscript and have made a summary of use and outputs available. <p><br><b>AI-assisted writing tools have been used in the preparation of this manuscript?	No

Powered by Editorial Manager® and ProduXion Manager® from Aries Systems Corporation

Manuscript

Click here to access/download;Manuscript;Delgado_OrtisSunyer_PathoFact

PathoFact 2.0: An Integrative Pipeline for Antimicrobial Resistance Genes, Virulence Factors, Toxins, and Biosynthetic Gene Clusters Prediction in Metagenomes

Luis F. Delgado (luis.delgado@uni.lu)*[1][0000-0001-7850-5285], Júlia Ortís Sunyer (julia.ortissunyer@uni.lu)*[1][0000-0002-2714-7067], Cedric C. Laczny (cedric.laczny@uni.lu)[1][0000-0002-1100-1282], Oskar Hickl (oskar.hickl@lih.lu)[1][0000-0001-9959-8767], Patrick May (patrick.may@uni.lu)[1][0000-0001-8698-3770] & Paul Wilmes (paul.wilmes@uni.lu)[1,2][0000-0002-6478-2924]

1. Luxembourg Centre for Systems Biomedicine, University of Luxembourg, Esch-sur-Alzette, Luxembourg
2. Department of Life Sciences and Medicine, Faculty of Science, Technology and Medicine, University of Luxembourg, Esch-sur-Alzette, Luxembourg

Contact: Paul Wilmes (paul.wilmes@uni.lu)

*These authors contributed equally

Abstract

Background

Antimicrobial resistance (ARG) and virulence factors (VFs) are central contributors to the global health crisis surrounding drug-resistant infections.

Findings

We introduce PathoFact 2.0, an enhanced pipeline for improved ARG, VF, and toxin prediction. Key updates include an updated machine learning (ML) model for VF identification, expanded hidden Markov model profiles for VFs and toxin-related proteins, a new ML model for toxin (and toxin-associated proteins) identification, and the integration of antiSMASH 7.0 for predicting biosynthetic gene clusters.

Conclusions

Our upgrades make PathoFact 2.0 a more powerful and user-friendly platform for predicting microbiome-based pathogenicity and resistance, providing a crucial tool for better understanding and addressing the challenges posed by antimicrobial resistance and infectious diseases.

PathoFact 2.0 is available at https://gitlab.com/uniluxembourg/lcsb/systems-ecology/pathofact2. It is compatible with Linux operating systems.

Keywords

Antimicrobial resistance genes, virulence factors, toxin-related proteins, biosynthetic gene clusters, metagenomes, machine learning

Findings

Introduction

Microbiomes represent highly complex and diverse ecological communities of bacteria, fungi, viruses, and archaea, many of which possess the potential to cause disease. Human, animal, and environmental microbiomes harbour commensal and pathogenic microorganisms, contributing to the emergence of infectious diseases. Moreover, these microorganisms play a critical role in the development of antibiotic-resistant infections through the presence of antimicrobial resistance genes (ARGs) and virulence factors (VFs)[1,2].

ARGs are genetic elements that confer bacterial resistance to antibiotics, acquired via mutations or horizontal gene transfer. Resistance genes can be divided into categories based on the antibiotics to which they confer resistance [3]. The Antibiotic Resistance Ontology (ARO) contains information on antibiotic resistance genes, the mutations that cause them, their products, mechanisms, associated phenotypes, antibiotics, and molecular targets [4].

Bacterial pathogens use specific genes, known as VFs, during their interactions with hosts. These factors help them attach to and invade host tissues, survive within the host, spread, and ultimately cause damage. The harm inflicted can vary, ranging from minor disruptions to severe or even fatal outcomes [5]. VFs can be classified as secretory, membrane-associated, or cytosolic. Cytosolic virulence factors promote rapid adaptive shifts in bacterial metabolism, physiology, and morphology, enhancing survival and proliferation within the host. Membrane-associated factors contribute to bacterial adhesion and immune evasion at the host-cell interface. Secretory factors constitute a critical part of the bacterial armamentarium, enabling bacteria to counteract innate and adaptive immune defences. Secretory virulence factors often exhibit synergistic effects and induce cytotoxicity in host cells [6]. ARGs and VFs are often located on mobile genetic elements (MGEs), such as transposons, plasmids and phages, facilitating their transfer between bacterial cells [7,8].

Bacterial toxins play a crucial role in the development of infectious diseases, alongside various virulence factors employed by pathogens. They disrupt host processes and manipulate immune responses. Some toxins impair protein synthesis, destroy blood cells, or affect the nervous system. These toxins can be divided into two main categories: cell-associated endotoxins and extracellular, diffusible exotoxins. Endotoxins, such as lipopolysaccharides, are found in the outer membranes of Gram-negative bacteria and serve as potent inflammatory mediators that can induce systemic toxicity and septic shock in infected hosts [9]. Exotoxins, which are typically polypeptides and proteins, can stimulate a range of host responses by either acting directly on cell receptors or through enzymatic modulation [10,11]. Many bacterial toxins are secreted proteins that require signal peptides. Signal peptides are short amino acid sequences at the N-terminus of proteins that direct them to specific cellular compartments, such as the periplasm [12,13].

Biosynthetic gene clusters (BGCs) are responsible for synthesising specialised metabolites (SMs). Some SMs can increase pathogenicity; for example, clinical isolates of *Pseudomonas aeruginosa* produce siderophores, rhamnolipids, quinolones, and phenazines [14]. Similarly, *Burkholderia* strains produce virulence factors, such as toxoflavin, from *Burkholderia glumae* [15]. Notably, pyocyanin, a redox-active phenazine produced by *Pseudomonas aeruginosa*, plays a crucial role as a virulence factor in lung infections [16].

The threat that ARGs, VFs and toxins pose to human health is significant. The United Nations have identified antimicrobial resistance as a global threat, with estimates attributing 1.27 million deaths

annually to drug-resistant infections, potentially rising to 10 million by 2050 if unaddressed [17,18]. Thus, accurately predicting potential ARG and VF profiles is essential for early intervention, enabling anticipation of infection severity, improving treatment strategies, and ultimately reducing mortality rates from disease-causing pathogens.

Predicting and annotating ARGs, VFs, and toxins is challenging due to limited well-annotated data [19] and complex mechanisms involving gene transfer, mutations, and multifactorial interactions. Traditional annotation methods, which rely on sequence similarity, may overlook novel ARGs, VFs and toxins. In contrast, machine learning offers robust solutions through pattern recognition, enabling accurate predictions even with limited training data. An integrated bioinformatics pipeline enhances analysis by simultaneously examining ARGs, VFs, toxins, signal peptides, and BGCs from a single metagenomic sample. This comprehensive approach provides a more complete view of bacterial pathogenicity by capturing the full spectrum of virulence mechanisms, including antimicrobial resistance, toxin production, and secondary metabolic capabilities. This holistic analysis improves insights into pathogenicity and resistance, streamlines workflows, and simplifies data interpretation.

PathoFact, a pipeline first introduced in 2020, integrates ARG, VF, and bacterial toxin prediction from metagenomic data into a single tool [20]. Since the publication of PathoFact, several tools have been implemented to predict ARGs, VFs, and bacterial toxins [21–23]. Only one tool, HyperVR [22], has attempted to predict them simultaneously. However, HyperVR's repository is no longer available online, and the Zenodo archive from its original submission lacks the necessary databases.

Here, we present PathoFact 2.0 (Figure 1). It enhances the previous version by supporting protein sequences or contigs as input and by updating the ML VF model and the hidden Markov model (HMM) profiles of the conserved domain databases (CDD) [24] for VF and toxin-related protein annotation. We have also introduced the ability to predict BGCs using antiSMASH 7.0 [25]. antiSMASH is a tool that identifies, annotates, and analyses secondary metabolite BGCs across genomes.

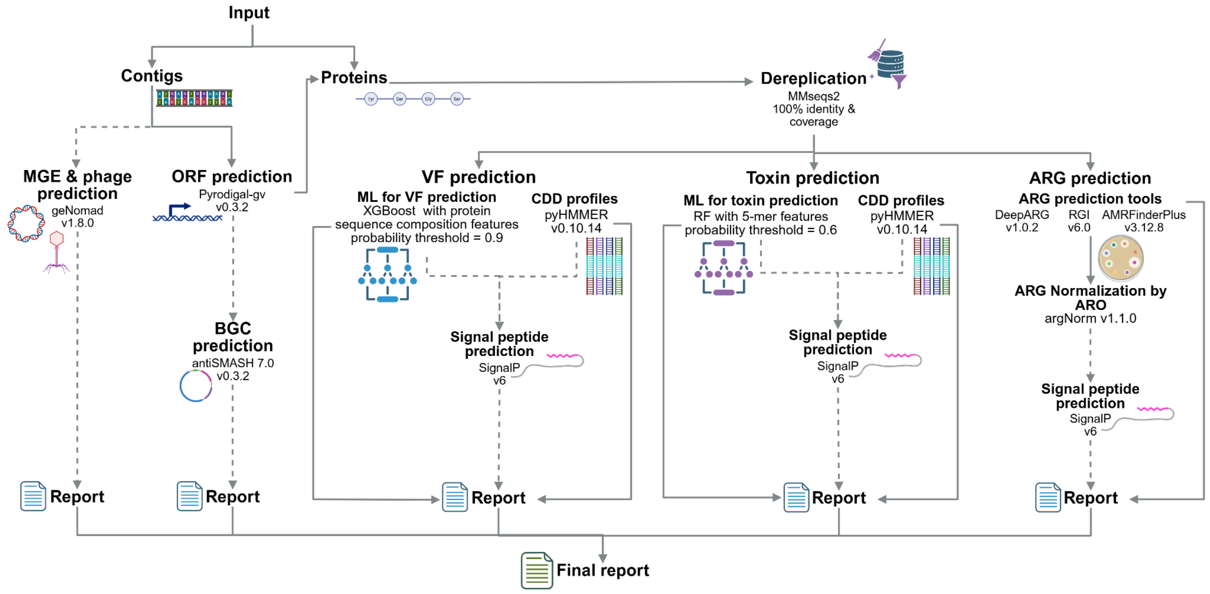

Figure 1. Schematic representation of PathoFact 2.0. Solid lines denote core modules, while dotted lines indicate optional user selection. The input is a FASTA file with either contig or protein sequences. If the input is a FASTA file containing contigs, open reading frames (ORFs) are predicted using Pyrodigal-gv. If the BGC option is selected, antiSMASH will use the GBK file for BGC prediction. GeNomad is used for MGE and phage predictions, outputting a FASTA file with protein sequences. Protein sequences are dereplicated using MMseqs2. After dereplication, ARGs, VFs and toxins are predicted using their respective modules. SignalP predicts the presence of signal peptides and their cleavage sites in proteins

from archaea, bacteria and eukarya. Individual reports are generated for each module, and an integrated report is produced that combines all module reports.

Pipeline Structure

Unlike version 1.0, which only supports contigs, PathoFact 2.0 supports the input of nucleotide sequences of contigs and protein sequence FASTA files, with proteins dereplicated to retain non-redundant sequences (based on 100% identity and coverage). For contig-based inputs, open reading frames are predicted using Pyrodigal-gv (version 0.3.2; [26,27]; https://github.com/althonos/pyrodigal-gv), a Python library that binds to Prodigal [22], followed by the detection of MGEs and phages using geNomad (version 1.8.0; [27]). GeNomad only processes nucleotide sequences; hence, MGEs and phages are not detected in protein sequence inputs. Based on user configuration, the pipeline then analyses the processed sequences using the BGC, ARG, VF, and toxin, and BGC (via antiSMASH) prediction modules. The information is compiled into individual module reports and an integrated report, also incorporating details from SignalP and geNomad (Figure 1). Additionally, PathoFact 2.0 generates a FASTA file of proteins identified as ARGs, VFs, or toxins.

Pipeline Installation

PathoFact 2.0 is implemented using Snakemake (version 7.25.0; [28]). An installation script simplifies the setup by installing the required software and downloading databases with a single command. PathoFact 2.0 is open-source (GNU General License v3.0 or later) and freely available at https://gitlab.com/uniluxembourg/lcsb/systems-ecology/pathofact2, where detailed instructions for pipeline installation, configuration, and output are provided.

ARG Prediction Updates

ARG prediction in PathoFact 2.0 integrates DeepARG (version 1.0.2; [29]), RGI (version 6; [4]), and AMRFinderPlus (version 3.12.8; [30]). While DeepARG and RGI have been updated from PathoFact 1.0, AMRFinderPlus has been newly incorporated. Each tool has distinct strengths: DeepARG offers high precision and recall; RGI provides robust predictions based on an extensive database, utilising homology and single-nucleotide polymorphism (SNP) models; and AMRFinderPlus efficiently identifies resistance genes and mutations using NCBI resources. The ARG prediction module (Figure 1) report includes protein IDs, ARG classes, prediction probabilities, database accession numbers, and optional data on signal peptides, plasmids, and virus markers. PathoFact 2.0 uses argNorm [31] to map detected genes to the ARO, thereby facilitating comparison of ARG annotation outputs by ensuring standardised and comparable results.

Generalities about Machine learning training set-up and “non-pathogenic” dataset

The “non-pathogenic” dataset for the ML models was constructed by selecting SwissProt sequences lacking ARG, VF, and toxin keywords [KW-0568 (pathogenesis-related protein), KW-0843 (virulence), KW-0800 (toxin), KW-0046 (antibiotic resistance), KW-9995 (disease)] and restricted to bacteria (taxonomy_id 2), archaea (taxonomy_id 2157), fungi (taxonomy_id 4751), and viruses (taxonomy_id 10239). Additionally, proteins from non-pathogenic organisms to humans (Supplementary Table S1) were included from NCBI. MMseqs2 (version 15.6f452; [32]) was used to obtain a set of dereplicated

protein sequences.

The ML model was trained using an 80/20 split of the dataset, with 80% for training and 20% for testing. The Synthetic Minority Oversampling Technique (SMOTE) was employed to address the dataset's imbalance [33]. Several ML models were built using Scikit-learn (version 1.5.2; [34] and tested: RandomForest (RF) and XGBoost, using k-mers 3 to 8 or protein sequence composition features (amino acid composition (AAC), dipeptide composition (DPC), composition (CTDC), transition (CTDT), and distribution (CTDD) [35]) as features. Hyperparameter optimisation was performed, using a 5-fold cross-validation with HalvingGridSearchCV from scikit-learn [34]. The best-performing model was selected based on the Matthews correlation coefficient (MCC) score.

Toxin Prediction Updates

Compared to version 1.0, the toxin prediction module now employs a ML model instead of a purely alignment-based bit score threshold, enhancing detection accuracy (Table 1). Curated training data was obtained from SwissProt [36], filtered for bacterial (taxonomy_id 2), archaeal (taxonomy_id 2157), fungal (taxonomy_id 4751) and viral (taxonomy_id 10239) toxin sequences (KW-0800, toxin). The dataset was supplemented with entries from toxin-specific databases such as the Toxin Exposome Database (T3DB) [10], which catalogues bacterial protein toxins; the Database for Bacterial ExoToxins (DBETH) [11]; TADB version 3.0, which includes protein sequences of bacterial toxin–antitoxin (TA) pairs from types I to VIII [37]; sequences from SecReT6 [38], encompassing T6SS gene cluster components, T6SS regulator (T6SR), accessory proteins (T6SA), effectors (T6SE), and immunity proteins (T6SI); and the prokaryotic antimicrobial toxins (PAT) database [39].

MMseqs2 (version 15.6f452; [32]) was used to dereplicate the dataset of 1,112,357 protein sequences (100% identity and coverage), yielding 213,363 unique protein sequences, corresponding to the “toxin-related” dataset. It is essential to note that this dataset encompasses both effector toxin proteins and their associated proteins, including antitoxins, regulators, and accessory proteins. This offers three main benefits: 1) Recent reports suggest that the same bacterial toxins can function as part of self-inhibiting toxin-antitoxin modules within one organism, while in another organism, they have evolved into toxin effectors that are injected into target cells [40,41]. 2) In bacteria, genes located close together often share a functional relationship, such as those co-transcribed in an operon. A comprehensive toxin dataset can help identify new toxins and related genes through their genomic context, known as "toxin Islands". These islands may be involved in toxin synthesis, processing, or secretion and may also confer immunity or facilitate horizontal gene transfer among bacteria. In fact, they are often abundant in mobile genetic elements [42]. 3) A large database improves the performance of machine learning classification methods [43].

HMM profiles were built using the conserved-domain FASTA files (https://ftp.ncbi.nih.gov/pub/mmdb/cdd/fasta.tar.gz) from CDD [24]. The 213,363 unique protein sequences in the “toxin-related” dataset were annotated using the CDD HMM profiles. Those with a bitscore above 25 were chosen as HMM profiles for toxin annotation and incorporated into Pathofact 2.0 for protein annotation.

We acknowledge that, although there is no standard for creating negative datasets, they play a crucial role in influencing model performance. Therefore, to improve the quality of our “non-toxin” dataset, potential ARGs, VFs (with high probability), and toxins were filtered out of the “non-pathogenic” dataset using PathoFact 1.0 predictions. The “non-toxin” consists of 213,129 non-redundant protein sequences.

The Toxin ML model is a RF with 5-mer features (default hyperparameter setting:

n_estimators=100,max_depth=None, min_samples_split=2).

The toxin prediction module generates a report containing the proteinID, protein domains, bitscore, toxin ML probability, other identical proteins found in the sample, and optionally SignalP, plasmid marker, and virus marker information.

VF Prediction Updates

The VF prediction model was refined and updated with new HMM profiles. Training data was derived from SwissProt [36], selecting sequences annotated with the virulence keyword (KW-0843) and expanded using the Virulence Factor Database (VFDB; [44]). After dereplication (with 100% identity and coverage), the original set of 32,511 sequences, using MMseqs2, comprised 30,695 non-redundant sequences, corresponding to the "VF dataset". We performed a search of the "VF dataset" against the CDD HMM profiles, and those with a bit score of 25 and higher were selected as VF HMM profiles for PathoFact 2.0. The HMM profile dataset annotates the predicted VF domains instead of using them as input for the classification, as the previous version did.

To create the "non-VF" dataset for the ML VF model, we filtered out any potential VFs (with high and low probabilities), ARGs, and toxins based on PathoFact 1.0 predictions from the "non-pathogenic" dataset. This resulted in a dataset consisting of 41,774 VF protein sequences.

The VF model is a XGBoost with protein sequence composition features (learning_rate': 0.1, 'n_estimators': 2000). This module generates a report containing the proteinID, protein domains, bitscore, virulence factor ML probability, other identical proteins found in the sample, and optionally SignalP, plasmid marker, and virus marker information.

Additional Functionalities

PathoFact 2.0 integrates SignalP (version 6; [24]) and antiSMASH (version 7.0; [19]), both of which are optional features that accommodate diverse research needs. SignalP is designed to predict the presence and location of signal peptides in protein sequences. It requires a separate license and must be requested. AntiSMASH is designed to identify and annotate BGCs in bacterial and fungal genomes.

Evaluation of the performance of the PathoFact 2.0 pipeline

We evaluated the performance of PathoFact 2.0 and the new VF and toxin modules using the test datasets described above. We did not include ARGs in the validation step, as the respective module is based on existing tools that have already demonstrated high accuracy [4,29,30].

Virulence factors and toxin-related protein prediction

The VF and toxin-related modules (Figure 1) were evaluated across various probability thresholds on the entire test dataset and on subsets of the test dataset. These subset datasets were created based on sequence similarity to the training dataset, with a range of 40% to 100% similarity and 80% coverage. This approach aimed to assess prediction accuracy on proteins in the testing dataset with low similarity to the training dataset, specifically including only sequences with less than 40-100% identity to any training sequence. The performance evaluation is based on the Matthew correlation coefficient (MCC) and the precision (to reduce the number of false positive VF and toxin-related predictions), taking into account the dataset imbalance (a higher number of "non-toxin" and "non-VF" sequences compared to

"toxin-related" and "VF" sequences in the test subsets). The MCC is a more reliable statistical measure that yields a high score only when the prediction performs well across all four categories of the confusion matrix (true positives, false negatives, true negatives, and false positives), and it is proportional to both the number of positive and negative elements in the dataset [45]. We found that probabilities of 0.9 for VFs and 0.6 for toxins provide a good balance between high MCC and precision across different test subsets (Figure 2).

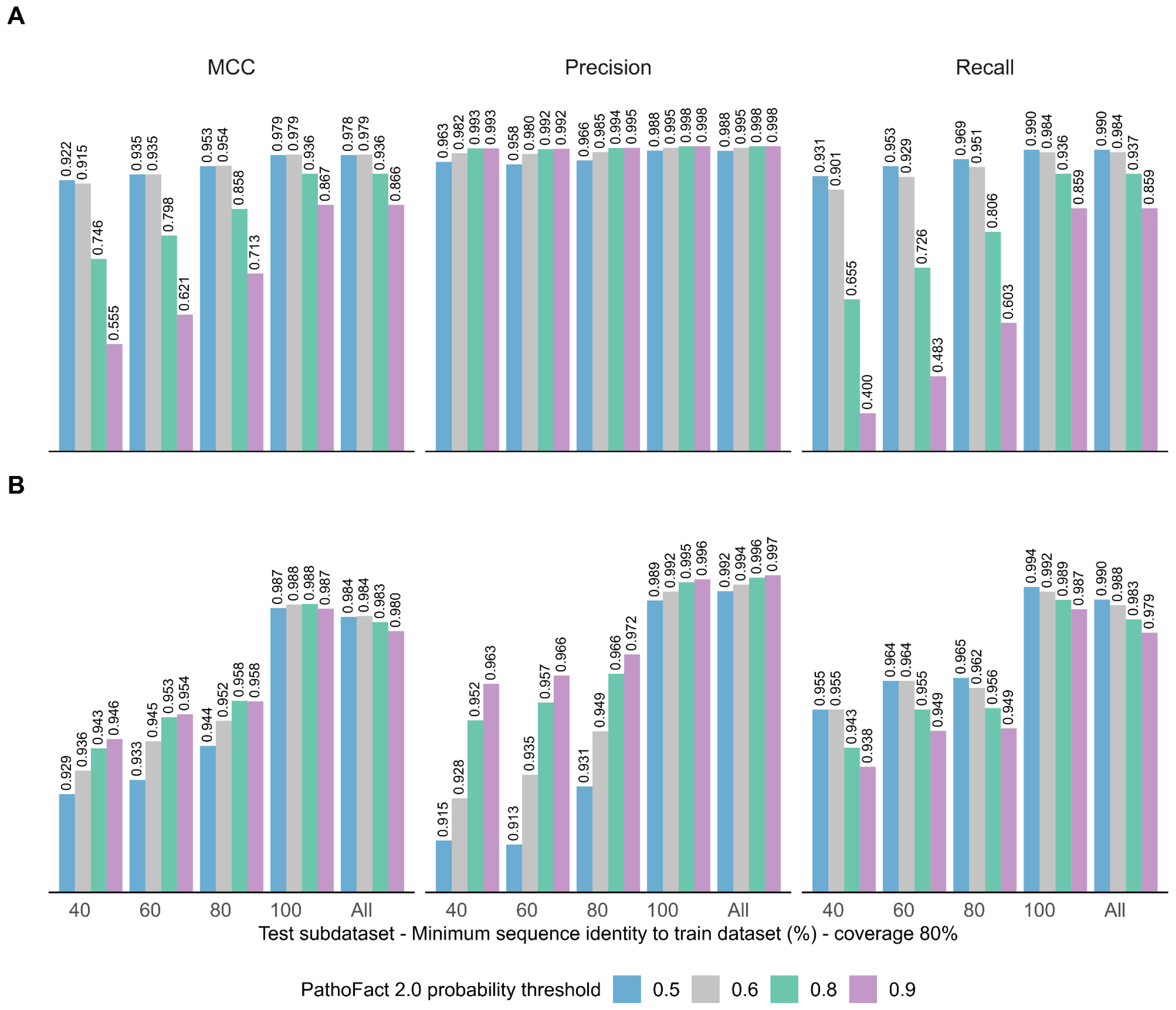

Figure 2. Performance evaluation of toxin and virulence factor prediction modules across probability thresholds. A) Toxin-related prediction module evaluation. **B)** Virulence factors prediction module evaluation. The modules were evaluated across a range of probability predictions (0.5-0.9). The entire test dataset (All) and subsets of the test datasets were used for evaluation. These subset datasets were created based on sequence similarity to the training dataset, with similarity levels of 40%, 60%, 80%, and 100%, and an 80% coverage threshold. Only sequences with similarity below these percentages were included in the respective test subsets.

Benchmarking

The Pathofact 2.0 virulence factor prediction module was compared to VirulentHunter [46], using the default parameters. VirulentHunter is a deep learning framework that simultaneously identifies and classifies VF directly from protein sequences, which outperforms other virulence factor predictors (MP4 [47], VirulentPred 2.0 [48], and DeepVF [49]). A notable feature of VirulentHunter is that it provides VF

category classification; however, it takes about 2 minutes to analyse 500 protein sequences (using 1 GPU), which is a drawback for metagenomic sample analysis, where thousands to millions of proteins are predicted from a single sample, whereas PathoFact 2.0 requires only 4 seconds (using 1 CPU, with the option to utilise more CPUs) to analyse 500 protein sequences (Table 1).

Table 1. Runtime comparison of PathoFact 2.0 and VirulentHunter

Number of protein sequences	VirulentHunter	PathoFact 2.0				
	1 GPU	1 CPU	2 CPU	4 CPU	6 CPU	8 CPU
500	2 min 21 s	3.7 s	2.6 s	2.0 s	1.9 s	1.8 s
5500	25 min 42 s	29.9 s	17.4 s	10.2 s	8.1 s	7.4 s
10000	54 min 11 s	55.4 s	29.3 s	16.7 s	13.1 s	11.5 s
30000	2 h 45 min 50 s	2 min 45 s	1 min 32.9 s	52.1 s	40.3 s	36.0 s

Since VirulentHunter and Pathofact 2.0 use a similar approach to obtain the “VF dataset” for model training, we have removed sequences from the Pathofact 2.0 test dataset that have 100% identity (>=80% coverage) to the VirulentHunter training dataset, resulting in a “clean VF test dataset”. We used the same test subset approach previously described: the subset datasets were created based on sequence similarity to the Pathofact 2.0 training dataset, with a range of 40% to 100% similarity and 80% coverage to the “clean VF test dataset”.

The Pathofact 2.0 toxin-related module was compared with ToxinPred2 [42] using the default parameters, i.e., Hybrid (RF+BLAST+MERCI) with a threshold of 0.6. The ToxinPred2 website restricts predictions to approximately 2000 proteins. Since ToxinPred2 is designed to predict protein toxicity, we selected sequences from the Pathofact 2.0 “toxin-related” test dataset that are directly linked to toxins and removed the remaining “toxin-related” proteins. In short, using the header information from the Pathofact 2.0 “toxin-related” test dataset, we kept only toxin sequences from the toxin-antitoxin sequences from the TADB, the effector factor sequence from the SecReT6 database, bacterial protein toxins from T3DB, bacterial exotoxins from DBETH, and sequences from Swissprot (KW-0800, toxin), as previously described. Additionally, we kept sequences longer than 35 amino acids and excluded protein sequences containing ‘BJOUXZ’ as the ToxinPred2 dataset was created using these criteria [50]. From these, we randomly chose 1000 sequences. Then, we randomly selected 1000 sequences from the Pathofact 2.0 “non-toxin” test dataset. Due to the limited number of sequences, we did not use the test-subset approach to evaluate ToxinPred2 and Pathofact 2.0 toxin-related modules on this 2,000-sequence test dataset.

As shown in Figure 3, Pathofact 2.0 VF and toxin-related modules exhibited higher MCC values across different test subsets compared to VirulentHunter and ToxinPred2, respectively.

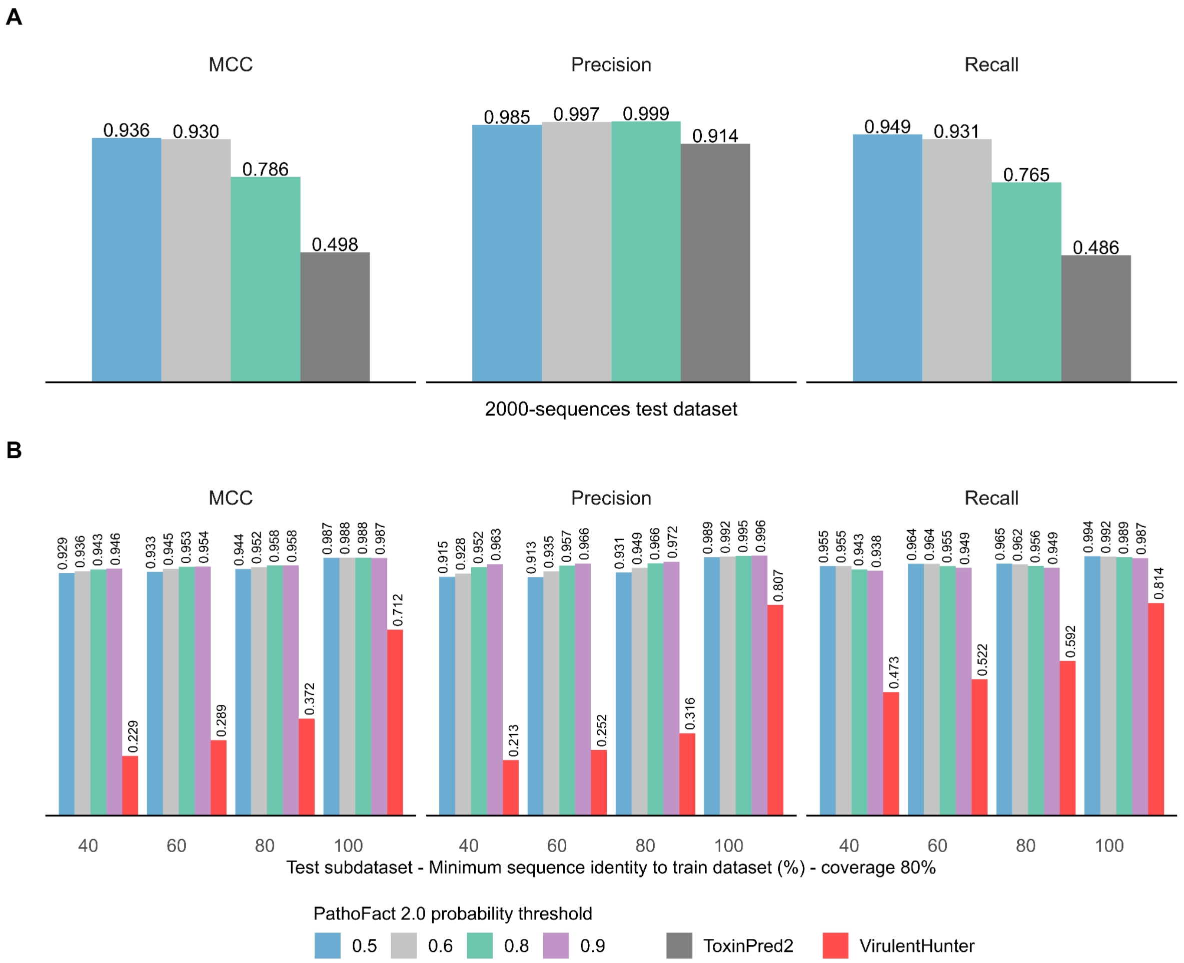

Figure 3. Comparative benchmarking of toxin and virulence factor prediction modules. A) Toxin-related module benchmarking. The Pathofact 2.0 toxin-related module was compared to ToxinPred2 (Hybrid: RF+BLAST+MERCI, threshold = 0.6) using the web version. A balanced toxin test dataset (1,000 toxin and 1,000 non-toxin sequences) was built from the Pathofact 2.0 toxin-related test dataset, selecting only sequences from curated toxin sources (TADB, SecReT6, T3DB, DBETH, SwissProt), applying ToxinPred2's filtering criteria. Several probability cutoffs, 0.5, 0.6 and 0.8, of the Pathofact 2.0 toxin-related module were evaluated. MCC, precision, and recall are shown. **B)** Virulence factors module benchmarking. The Pathofact 2.0 VF module was compared to VirulentHunter. Sequences identical (100% identity, ≥80% coverage) to VirulentHunter's training data were removed from the Pathofact 2.0 test dataset. Then, test subset datasets were created based on 40–100% similarity to the Pathofact 2.0 training set. These subset datasets were created based on sequence similarity to the training dataset, with similarity levels of 40%, 60%, 80%, and 100%, and an 80% coverage threshold. Only sequences with similarity below these percentages were included in the respective test subsets. Several probability cut-offs (0.5, 0.6, 0.8, and 0.9) of the Pathofact 2.0 VF module were evaluated.MCC, precision, and recall are presented for each test subset.

Virulence factors and toxin-related protein prediction with contig sequences as input

To evaluate PathoFact 2.0 at the contig level, we analysed publicly available complete genomes from pathogenic and non-pathogenic bacteria, including various *Escherichia coli* strains. Figure 4 illustrates clear differences in virulence and toxin profiles between non-pathogenic and pathogenic E. coli strains,

specifically regarding the virulence- and toxin-related proteins present in MGEs, including plasmids and prophages. It is well known that VFs of pathogenic *E. coli* are often encoded on genetic elements, such as plasmids, bacteriophages, transposons, and pathogenicity islands, which can be mobilised into different strains to create novel combinations of virulence factors [51,52]. The same pattern is observed in pathogenic strains of several genera compared to non-pathogenic strains (Supplementary Figure S1), particularly for *Klebsiella pneumoniae* and *Salmonella enterica*. These findings highlight the importance of examining virulence from a systems perspective rather than focusing solely on the presence or absence of individual factors. A thorough assessment should consider not only whether a virulence- or toxin-related protein is encoded within a MGE, but also its functional context, such as whether it is secreted or part of a BGC.

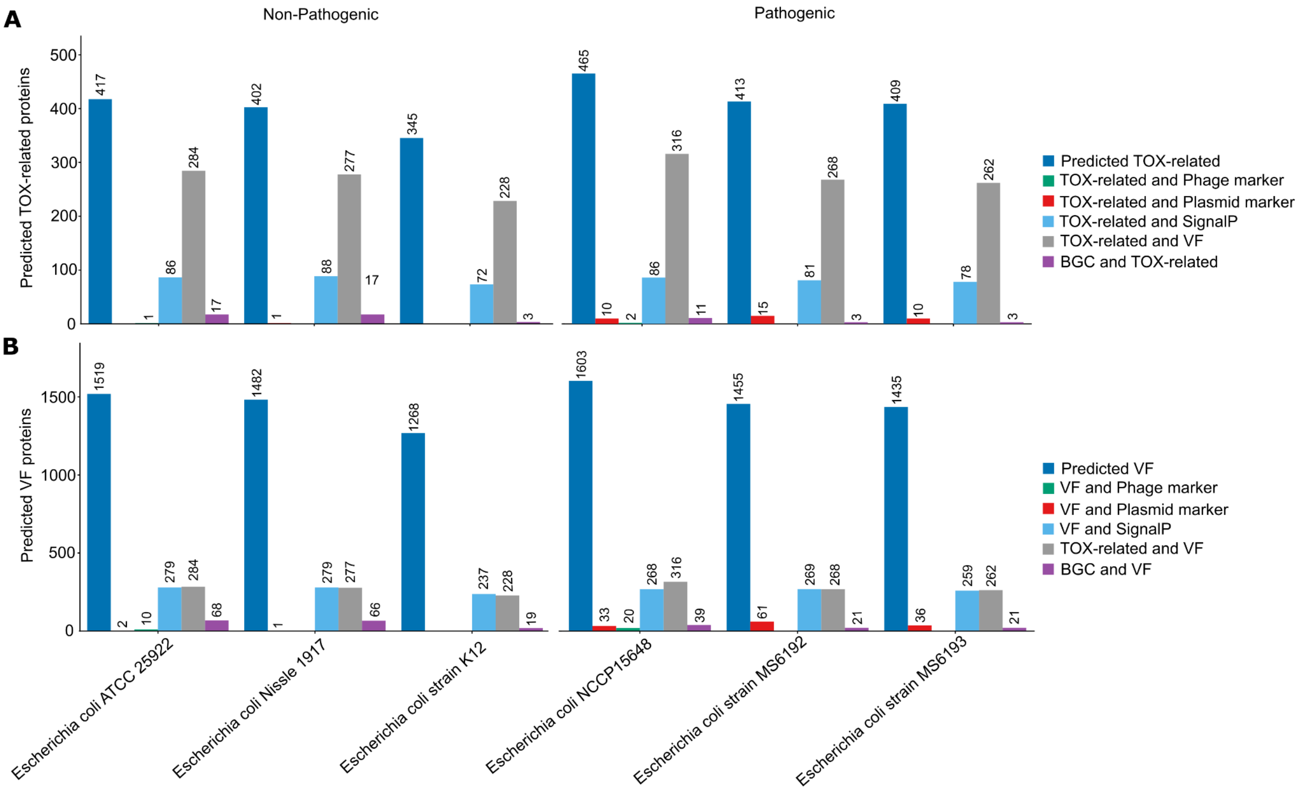

Figure 4. Comparative analysis of toxin-related (A) and virulence factor (B) profiles in non-pathogenic and pathogenic *Escherichia coli* strains. Bar charts represent the distribution of predicted toxin-related (A) and virulence-associated (B) proteins across non-pathogenic (left panel) and pathogenic (right panel) *E. coli* strains. The categories include total predicted virulence factors/toxin-related proteins (dark blue), those associated with plasmid markers (red), those associated with phage markers (green), and proteins predicted by SignalP to be secreted (light blue). Additional categories include toxin-related virulence factors (TOX-related ∩ VF, grey) and biosynthetic gene clusters overlapping (purple). Numerical values above each bar indicate the total count of proteins identified in each category for the corresponding strain.

Benchmarking

The PathoFact 2.0 VF module was compared to PathoFact 1.0 and metaVF [21], an alignment-based toolkit that identifies species-level VFs associated with pathobionts. To our knowledge, no other method is available to predict VF from contig sequences. PathoFact 2.0 consistently detected a greater number of VFs than both PathoFact 1.0 and MetaVF (Figure 5, Supplementary Figures S2–S3). Notably, MetaVF failed to identify any VF in 5 out of the 10 pathogenic reference strains tested (Figure 5), highlighting its limited ability to detect VFs. In addition, the PathoFact 2.0 toxin-related module was compared to

PathoFact 1.0 (Supplementary Figures S4–S5), predicting more toxin-related proteins than its predecessor.

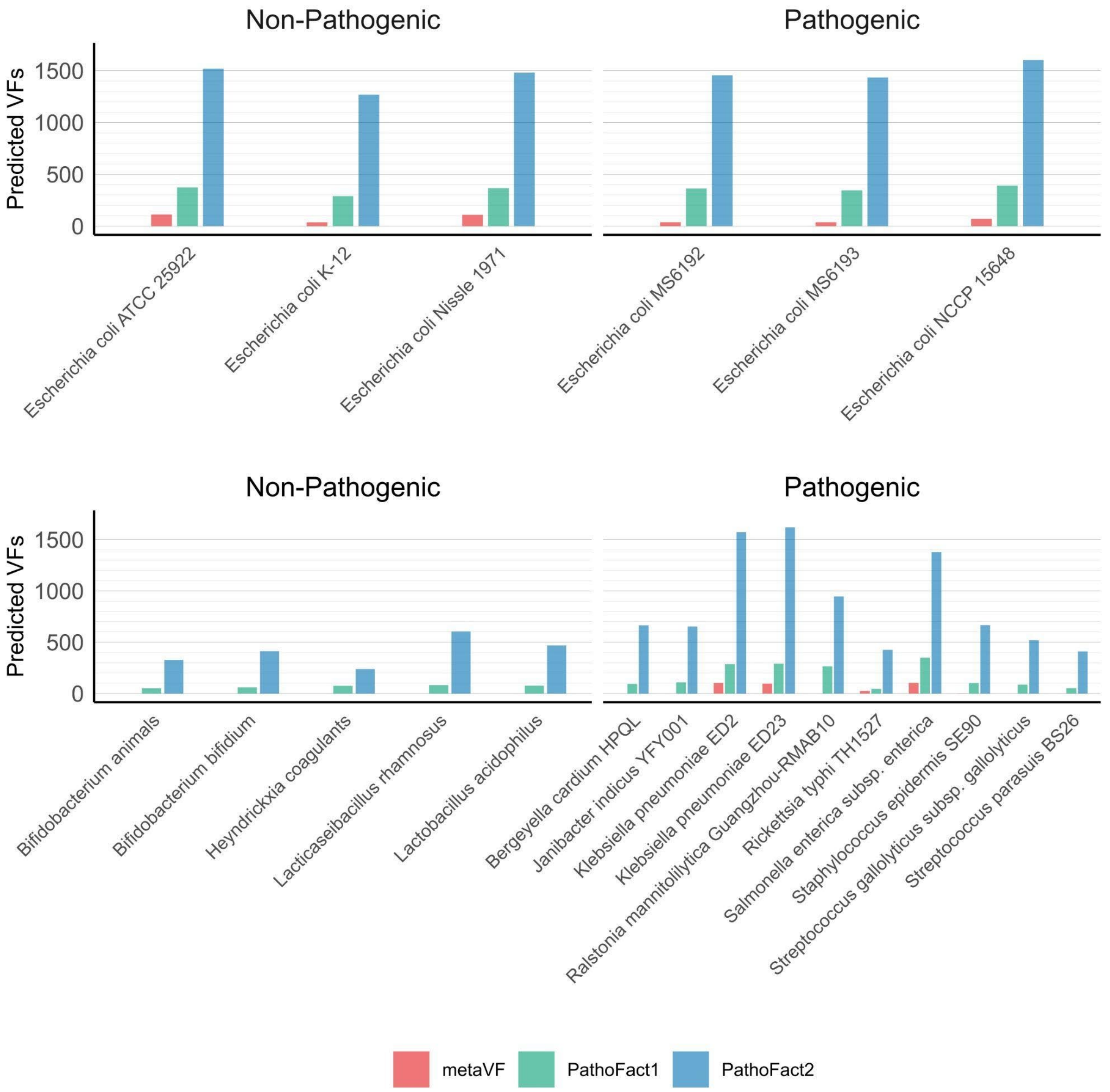

Figure 5. Comparative performance of Pathofact 2.0 (blue) versus PathoFact (green) and metaVF (red) in predicting virulence factors (VFs) in non-pathogenic (left) and pathogenic (right) strains.

Limitations of PathoFact 2.0

It is well known that non-pathogenic strains also harbour genes for VFs and toxins [5]. Therefore, PathoFact 2.0 is most effective as an initial screening tool to detect potential genes, which can subsequently be used in comparative studies to distinguish confirmed pathogenic cases from control groups. Pathofact 2.0 provides a probability score indicating whether a protein is likely to be a VF or toxin-related; however, confirming the true link between the predicted candidates' pathogenic potential and infectious diseases requires experimental validation. Finally, PathoFact 2.0 does not directly classify VF or toxin types (e.g., adhesion or genotoxin); however, it provides detailed descriptions of conserved protein domains from CDD [18], indicating their functions when available.

Conclusions

ARGs, VFs, and toxins represent major threats to global health. Therefore, accurately detecting these elements is crucial for assessing the presence and potential risks of pathogenic microorganisms in microbiomes and identifying reservoirs of pathogenicity. Our improved pipeline, PathoFact 2.0, offers significant improvements over PathoFact (its predecessor), ToxinPred2, VirulentHunter, and metaVF. SignalP has been upgraded and made optional to further optimise performance, providing users with flexibility based on their requirements. Additionally, antiSMASH 7.0 facilitates the prediction of BGCs, recognising emerging evidence that some BGC-encoded factors might increase virulence. Furthermore, we have integrated geNomad, a cutting-edge tool for identifying MGEs, including plasmids and phages linked to ARGs, VFs, and toxins across various bacterial species. The update of PathoFact 2.0 improves the accuracy and sensitivity of analyses, while boosting computational efficiency.

Methods

Datasets

This study utilised publicly available datasets containing complete genomes from pathogenic and non-pathogenic bacteria, including various *Escherichia coli* strains, from NCBI. The accession numbers of the bacteria used are indicated in Supplementary Table S2.

Availability of Supporting Source Code and Requirements

Project name: Pathofact 2.0
Project homepage: https://gitlab.com/uniluxembourg/lcsb/systems-ecology/pathofact2
Operating system(s): Linux
Programming language: Python, Snakemake
Other requirements: Mamba, conda.
License: GNU General License v3.0 or later
Biotools: -
RRID: SCR_027650

Additional Files

Supplementary Table S1. List of microorganisms non-pathogenic to humans and their total protein count obtained from the NCBI Database.

Supplementary Table S2. Bacterial strains used in this study, including their classification as pathogenic or non-pathogenic, species/strain information, genome assembly or reference version, and corresponding accession numbers.

Supplementary Figure S1. Comparative analysis of toxin-related and virulence factor profiles in non-pathogenic and pathogenic bacterial strains. Bar charts represent the distribution of predicted toxin-related (A) and virulence-associated (B) proteins across non-pathogenic (left panel) and pathogenic (right panel) *E. coli* strains. The categories include total predicted virulence factors/Toxin-related proteins (dark blue), associated with plasmid markers (red), phage markers (Green), and proteins predicted by SignalP to be secreted (light blue). Additional categories include toxin-related virulence factors (TOX-related ∩ VF, grey) and biosynthetic gene clusters overlapping (purple). Numerical values above each bar indicate the total count of proteins identified in each category for the corresponding strain.

Supplementary Figure S2. Comparative performance of Pathofact 2.0 (blue) versus PathoFact1 (green) and metaVF (red) in predicting virulence factors (VFs) in non-pathogenic (left) and pathogenic (right) *Escherichia coli* strains. The top panel shows the total number of predicted VFs. The second panel depicts the subset predicted to be secreted. The third panel shows VFs predicted to be plasmid-encoded, while the fourth panel presents those predicted to be prophage-associated.

Supplementary Figure S3. Comparative performance of Pathofact 2.0 (blue) versus PathoFact (green) and metaVF (red) in predicting virulence factors (VFs) in non-pathogenic (left) and pathogenic (right) bacterial strains. The top panel shows the total number of predicted VFs. The second panel depicts the subset predicted to be secreted. The third panel shows VFs predicted to be plasmid-encoded, while the fourth panel presents those predicted to be prophage-associated.

Supplementary Figure S4. Performance comparison of PathoFact 2.0 versus PathoFact for ARG and toxin prediction in Escherichia coli. A) Comparative performance of PathoFact 2.0 (blue) versus PathoFact (green) in predicting antimicrobial resistance genes (ARGs) in non-pathogenic (left) and pathogenic (right) *E. coli* strains. **B)** Comparative performance of PathoFact 2.0 (blue) versus PathoFact (green) in predicting toxin-associated proteins in non-pathogenic (left) and pathogenic (right) *E. coli* strains. The top panel shows the total number of predicted toxin-related proteins, while the bottom panel shows those that contain signal peptides, as identified by SignalP.

Supplementary Figure S5. Performance comparison of PathoFact 2.0 versus PathoFact for ARG and toxin prediction in bacterial strains. A) Comparative performance of PathoFact 2.0 (blue) versus PathoFact (green) in predicting antimicrobial resistance genes (ARGs) in non-pathogenic (left) and pathogenic (right) bacterial strains. **B)** Comparative performance of PathoFact 2.0 (blue) versus PathoFact (green) in predicting toxin-associated proteins in non-pathogenic (left) and pathogenic (right) bacterial strains. The top panel shows the total number of predicted toxin-related proteins, while the bottom panel shows those that contain signal peptides, as identified by SignalP.

Abbreviations

AAC, amino acid composition; ARO, antibiotic resistance ontology; ARGs, antimicrobial resistance genes; BGCs, biosynthetic gene clusters; CDD, conserved domains database; CTDC, (Composition, Transition, Distribution)-composition; CTDD, (Composition, Transition, Distribution)-distribution; CTDT, (Composition, Transition, Distribution)-transition; DBETH, database for bacterial exotoxins; DPC, dipeptide composition; HMMs, hidden Markov models; MCC, Matthews correlation coefficient; MGEs, mobile genetic elements; ML, machine learning; ORF, open reading frame; PAT, prokaryotic

antimicrobial toxins database; RF, random forest; SM, specialised metabolites; SMOTE, synthetic minority oversampling technique; SNP, single nucleotide polymorphisms; T3DB, toxin exposome database; VFDB, virulence factor database; VFs, virulence factors.

Acknowledgements

The experiments presented in this paper were carried out using the HPC facilities of the University of Luxembourg (Varrette et al., 2022). The manuscript also passed the Luxembourg Centre for Systems Biomedicine internal pre-publication check designed to ensure FAIRness and reproducibility.

Author Contributions

P.W. initiated the study, which involved the overall design and objective, and was led by L.F.D. and J.O.S. in the development of PathoFact 2.0. O.H. contributed to early brainstorming discussions on workflow design and database strategy. P.M. and C.C.L. contributed to the overall discussions. L.F.D. and J.O.S. wrote the draft manuscript. All authors read and commented on the manuscript.

Funding

This work has been supported by the Pélican grant from the Mie and Pierre Hippert-Faber Pélican Foundation under the aegis of Fondation de Luxembourg to JOS, as well as by the Luxembourg National Research Fund (FNR CORE/23/BM/15886415) and the European Research Council (ERC-CoG 863664) to PW. The Luxembourg Government further supported the work through the CoVaLux program.
This research was funded in whole, or in part, by the Luxembourg National Research Fund (FNR), grant reference (FNR CORE/23/BM/15886415). For the purpose of open access, and in fulfilment of the obligations arising from the grant agreement, the author has applied a Creative Commons Attribution 4.0 International (CC BY 4.0) license to any Author Accepted Manuscript version arising from this submission.

Data Availability

Pathofact 2.0 is accessible at https://gitlab.com/uniluxembourg/lcsb/systems-ecology/pathofact2.
Additionally, the core databases required to run the pipeline can be found at https://zenodo.org/records/14192463. The ML datasets used for training, validation, and benchmarking of PathoFact 2.0 can be found in https://zenodo.org/records/17647372.

Competing Interests

None declared.

References

1. Inda-Díaz JS, Lund D, Parras-Moltó M, Johnning A, Bengtsson-Palme J, Kristiansson E. Latent antibiotic resistance genes are abundant, diverse, and mobile in human, animal, and environmental microbiomes. Microbiome. 2023;11: 44. doi:10.1186/s40168-023-01479-0

2. Beceiro A, Tomás M, Bou G. Antimicrobial resistance and virulence: a successful or deleterious association in the bacterial world? Clin Microbiol Rev. 2013;26: 185–230.

doi:10.1128/CMR.00059-12

3. Jian Z, Zeng L, Xu T, Sun S, Yan S, Yang L, et al. Antibiotic resistance genes in bacteria: Occurrence, spread, and control. J Basic Microbiol. 2021;61: 1049–1070. doi:10.1002/jobm.202100201

4. Alcock BP, Huynh W, Chalil R, Smith KW, Raphenya AR, Wlodarski MA, et al. CARD 2023: expanded curation, support for machine learning, and resistome prediction at the Comprehensive Antibiotic Resistance Database. Nucleic Acids Res. 2023;51: D690–D699. doi:10.1093/nar/gkac920

5. Niu C, Yu D, Wang Y, Ren H, Jin Y, Zhou W, et al. Common and pathogen-specific virulence factors are different in function and structure. Virulence. 2013;4: 473–482. doi:10.4161/viru.25730

6. Sharma AK, Dhasmana N, Dubey N, Kumar N, Gangwal A, Gupta M, et al. Bacterial virulence factors: Secreted for survival. Indian J Microbiol. 2017;57: 1–10. doi:10.1007/s12088-016-0625-1

7. Blair JMA, Webber MA, Baylay AJ, Ogbolu DO, Piddock LJV. Molecular mechanisms of antibiotic resistance. Nat Rev Microbiol. 2015;13: 42–51. doi:10.1038/nrmicro3380

8. Rodríguez-Beltrán J, DelaFuente J, León-Sampedro R, MacLean RC, San Millán Á. Beyond horizontal gene transfer: the role of plasmids in bacterial evolution. Nat Rev Microbiol. 2021;19: 347–359. doi:10.1038/s41579-020-00497-1

9. Galanos C, Freudenberg MA. Bacterial endotoxins: biological properties and mechanisms of action. Mediators Inflamm. 1993;2: S11–6. doi:10.1155/S0962935193000687

10. Wishart D, Arndt D, Pon A, Sajed T, Guo AC, Djoumbou Y, et al. T3DB: the toxic exposome database. Nucleic Acids Res. 2015;43: D928–34. doi:10.1093/nar/gku1004

11. Chakraborty A, Ghosh S, Chowdhary G, Maulik U, Chakrabarti S. DBETH: A database of Bacterial ExoToxins for human. Nucleic Acids Res. 2012;40: D615–20. doi:10.1093/nar/gkr942

12. Green ER, Mecsas J. Bacterial secretion systems: An overview. Microbiol Spectr. 2016;4. doi:10.1128/microbiolspec.VMBF-0012-2015

13. Kaushik S, He H, Dalbey RE. Bacterial signal peptides- navigating the journey of proteins. Front Physiol. 2022;13: 933153. doi:10.3389/fphys.2022.933153

14. Lybbert AC, Williams JL, Raghuvanshi R, Jones AD, Quinn RA. Mining public mass spectrometry data to characterize the diversity and ubiquity of P. aeruginosa specialized metabolites. Metabolites. 2020;10: 445. doi:10.3390/metabo10110445

15. Elshafie HS, Camele I. An overview of metabolic activity, beneficial and pathogenic aspects of Burkholderia spp. Metabolites. 2021;11: 321. doi:10.3390/metabo11050321

16. Lau GW, Hassett DJ, Ran H, Kong F. The role of pyocyanin in Pseudomonas aeruginosa infection. Trends Mol Med. 2004;10: 599–606. doi:10.1016/j.molmed.2004.10.002

17. Ambassadors G, Patrons. World leaders commit to decisive action on antimicrobial resistance. In: UN Environment [Internet]. 26 Sep 2024 [cited 7 Oct 2025]. Available: https://www.unep.org/news-and-stories/press-release/world-leaders-commit-decisive-action-antimicrobial-resistance

18. Environment UN. Antimicrobial Resistance (AMR). In: UNEP - UN Environment Programme [Internet]. 18 Sep 2024 [cited 7 Oct 2025]. Available: https://www.unep.org/topics/chemicals-and-pollution-action/pollution-and-health/antimicrobial-resistance-amr

19. Bansal MA, Sharma DR, Kathuria DM. A systematic review on data scarcity problem in deep learning: Solution and applications. ACM Comput Surv. 2022;54: 1–29. doi:10.1145/3502287

20. de Nies L, Lopes S, Busi SB, Galata V, Heintz-Buschart A, Laczny CC, et al. PathoFact: a pipeline for the prediction of virulence factors and antimicrobial resistance genes in metagenomic data. Microbiome. 2021;9: 49. doi:10.1186/s40168-020-00993-9

21. Dong W, Fan X, Guo Y, Wang S, Jia S, Lv N, et al. An expanded database and analytical toolkit for identifying bacterial virulence factors and their associations with chronic diseases. Nat Commun. 2024;15: 8084. doi:10.1038/s41467-024-51864-y

22. Ji B, Pi W, Liu W, Liu Y, Cui Y, Zhang X, et al. HyperVR: a hybrid deep ensemble learning approach for simultaneously predicting virulence factors and antibiotic resistance genes. NAR Genom Bioinform. 2023;5: lqad012. doi:10.1093/nargab/lqad012

23. Rathore AS, Choudhury S, Arora A, Tijare P, Raghava GPS. ToxinPred 3.0: An improved method for predicting the toxicity of peptides. Comput Biol Med. 2024;179: 108926. doi:10.1016/j.compbiomed.2024.108926

24. Wang J, Chitsaz F, Derbyshire MK, Gonzales NR, Gwadz M, Lu S, et al. The conserved domain database in 2023. Nucleic Acids Res. 2023;51: D384–D388. doi:10.1093/nar/gkac1096

25. Blin K, Shaw S, Augustijn HE, Reitz ZL, Biermann F, Alanjary M, et al. antiSMASH 7.0: new and improved predictions for detection, regulation, chemical structures and visualisation. Nucleic Acids Res. 2023;51: W46–W50. doi:10.1093/nar/gkad344

26. Pyrodigal: Python bindings and interface to Prodigal, an efficient method for gene prediction in prokaryotes. Journal of Open Source Software. 7. doi:10.21105/joss.04296

27. Camargo AP, Roux S, Schulz F, Babinski M, Xu Y, Hu B, et al. Identification of mobile genetic elements with geNomad. Nat Biotechnol. 2024;42: 1303–1312. doi:10.1038/s41587-023-01953-y

28. Köster J, Rahmann S. Snakemake--a scalable bioinformatics workflow engine. Bioinformatics. 2012;28: 2520–2522. doi:10.1093/bioinformatics/bts480

29. Arango-Argoty G, Garner E, Pruden A, Heath LS, Vikesland P, Zhang L. DeepARG: a deep learning approach for predicting antibiotic resistance genes from metagenomic data. Microbiome. 2018;6: 23. doi:10.1186/s40168-018-0401-z

30. Feldgarden M, Brover V, Gonzalez-Escalona N, Frye JG, Haendiges J, Haft DH, et al. AMRFinderPlus and the Reference Gene Catalog facilitate examination of the genomic links among antimicrobial resistance, stress response, and virulence. Sci Rep. 2021;11: 12728. doi:10.1038/s41598-021-91456-0

31. Ugarcina Perovic S, Ramji V, Chong H, Duan Y, Maguire F, Coelho LP. argNorm: normalization of antibiotic resistance gene annotations to the Antibiotic Resistance Ontology (ARO). Bioinformatics. 2025;41. doi:10.1093/bioinformatics/btaf173

32. Steinegger M, Söding J. MMseqs2 enables sensitive protein sequence searching for the analysis

of massive data sets. Nat Biotechnol. 2017;35: 1026–1028. doi:10.1038/nbt.3988

33. Chawla NV, Bowyer KW, Hall LO, Kegelmeyer WP. SMOTE: Synthetic minority over-sampling technique. J Artif Intell Res. 2002;16: 321–357. doi:10.1613/jair.953

34. Pedregosa F, Varoquaux G, Gramfort A, Michel V, Thirion B, Grisel O, et al. Scikit-learn: Machine Learning in Python. arXiv [cs.LG]. 2012. Available: http://arxiv.org/abs/1201.0490

35. Chen Z, Zhao P, Li F, Leier A, Marquez-Lago TT, Wang Y, et al. iFeature: a Python package and web server for features extraction and selection from protein and peptide sequences. Bioinformatics. 2018;34: 2499–2502. doi:10.1093/bioinformatics/bty140

36. UniProt Consortium. UniProt: The universal protein knowledgebase in 2023. Nucleic Acids Res. 2023;51: D523–D531. doi:10.1093/nar/gkac1052

37. Guan J, Chen Y, Goh Y-X, Wang M, Tai C, Deng Z, et al. TADB 3.0: an updated database of bacterial toxin-antitoxin loci and associated mobile genetic elements. Nucleic Acids Res. 2024;52: D784–D790. doi:10.1093/nar/gkad962

38. Zhang J, Guan J, Wang M, Li G, Djordjevic M, Tai C, et al. SecReT6 update: a comprehensive resource of bacterial Type VI Secretion Systems. Sci China Life Sci. 2023;66: 626–634. doi:10.1007/s11427-022-2172-x

39. Liu Y, Liu S, Pan Z, Ren Y, Jiang Y, Wang F, et al. PAT: a comprehensive database of prokaryotic antimicrobial toxins. Nucleic Acids Res. 2023;51: D452–D459. doi:10.1093/nar/gkac879

40. Harms A, Liesch M, Körner J, Québatte M, Engel P, Dehio C. A bacterial toxin-antitoxin module is the origin of inter-bacterial and inter-kingdom effectors of Bartonella. PLoS Genet. 2017;13: e1007077. doi:10.1371/journal.pgen.1007077

41. Yadav SK, Magotra A, Ghosh S, Krishnan A, Pradhan A, Kumar R, et al. Immunity proteins of dual nuclease T6SS effectors function as transcriptional repressors. EMBO Rep. 2021;22: e51857. doi:10.15252/embr.202051857

42. Danov A, Segev O, Bograd A, Ben Eliyahu Y, Dotan N, Kaplan T, et al. Toxinome-the bacterial protein toxin database. MBio. 2024;15: e0191123. doi:10.1128/mbio.01911-23

43. Sordo M, Zeng Q. On sample size and classification accuracy: A performance comparison. Biological and Medical Data Analysis. Berlin, Heidelberg: Springer Berlin Heidelberg; 2005. pp. 193–201. doi:10.1007/11573067_20

44. Liu B, Zheng D, Zhou S, Chen L, Yang J. VFDB 2022: a general classification scheme for bacterial virulence factors. Nucleic Acids Res. 2022;50: D912–D917. doi:10.1093/nar/gkab1107

45. Chicco D, Jurman G. The advantages of the Matthews correlation coefficient (MCC) over F1 score and accuracy in binary classification evaluation. BMC Genomics. 2020;21: 6. doi:10.1186/s12864-019-6413-7

46. Chen C, Xu Y, Ouyang J, Xiong X, Łabaj PP, Chmielarczyk A, et al. VirulentHunter: deep learning-based virulence factor predictor illuminates pathogenicity in diverse microbial contexts. Brief Bioinform. 2025;26: bbaf271. doi:10.1093/bib/bbaf271

47. Gupta A, Malwe AS, Srivastava GN, Thoudam P, Hibare K, Sharma VK. MP4: a machine learning based classification tool for prediction and functional annotation of pathogenic proteins from

metagenomic and genomic datasets. BMC Bioinformatics. 2022;23. doi:10.1186/s12859-022-05061-7

48. Sharma A, Garg A, Ramana J, Gupta D. VirulentPred 2.0: An improved method for prediction of virulent proteins in bacterial pathogens. Protein Sci. 2023;32. doi:10.1002/pro.4808

49. Xie R, Li J, Wang J, Dai W, Leier A, Marquez-Lago TT, et al. DeepVF: a deep learning-based hybrid framework for identifying virulence factors using the stacking strategy. Brief Bioinform. 2021;22. doi:10.1093/bib/bbaa125

50. Sharma N, Naorem LD, Jain S, Raghava GPS. ToxinPred2: an improved method for predicting toxicity of proteins. Brief Bioinform. 2022;23: bbac174. doi:10.1093/bib/bbac174

51. Kaper JB, Nataro JP, Mobley HL. Pathogenic Escherichia coli. Nat Rev Microbiol. 2004;2: 123–140. doi:10.1038/nrmicro818

52. Johnson TJ, Nolan LK. Pathogenomics of the virulence plasmids of Escherichia coli. Microbiol Mol Biol Rev. 2009;73: 750–774. doi:10.1128/MMBR.00015-09

Table 1. Runtime Comparison of PathoFact 2.0 and VirulentHunter

Number of protein sequences	VirulentHunter	PathoFact 2.0				
	1 GPU	1 CPU	2 CPU	4 CPU	6 CPU	8 CPU
500	2 min 21 s	3.7 s	2.6 s	2.0 s	1.9 s	1.8 s
5500	25 min 42 s	29.9 s	17.4 s	10.2 s	8.1 s	7.4 s
10000	54 min 11 s	55.4 s	29.3 s	16.7 s	13.1 s	11.5 s
30000	2 h 45 min 50 s	2 min 45 s	1 min 32.9 s	52.1 s	40.3 s	36.0 s

Figure 1. Schematic representation of PathoFact2

Click here to access/download;Figure;Figure1.pdf

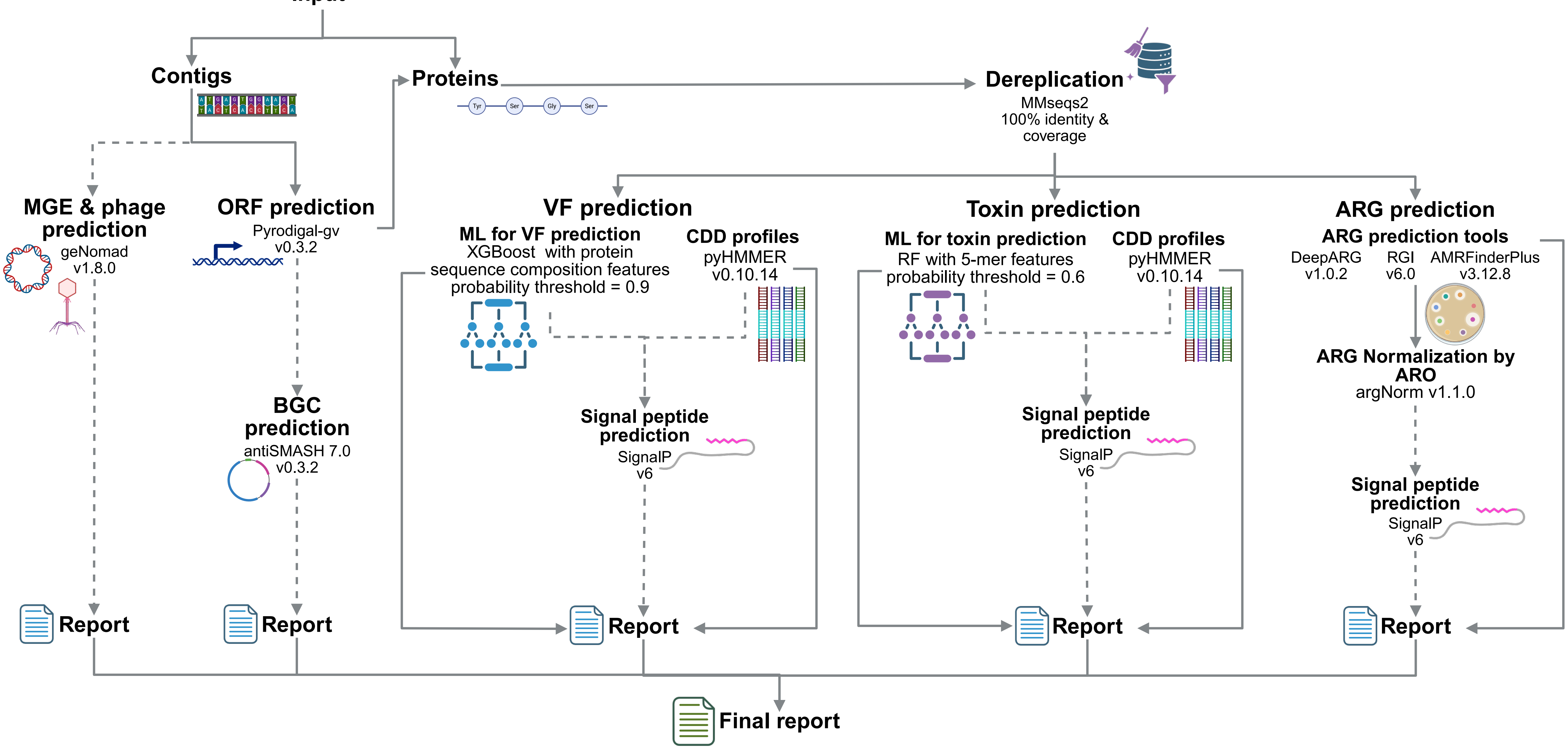

Figure 2. Performance evaluation of toxin and virulence factor prediction modules across probability thresholds

Click here to access/download;Figure;Figure2.pdf

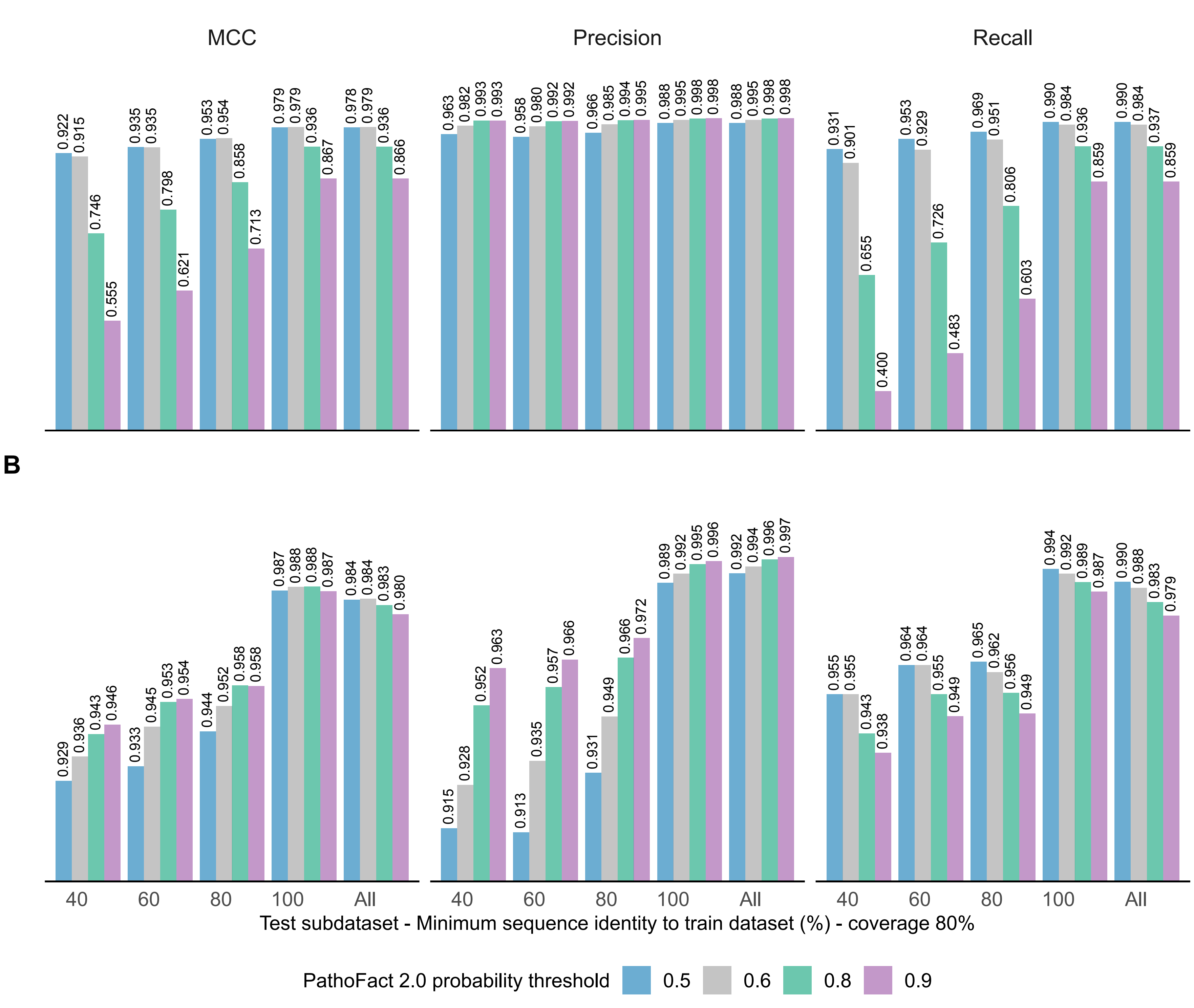

Figure 3. Comparative benchmarking of toxin and virulence factor prediction modules

Click here to access/download;Figure;Figure3.pdf

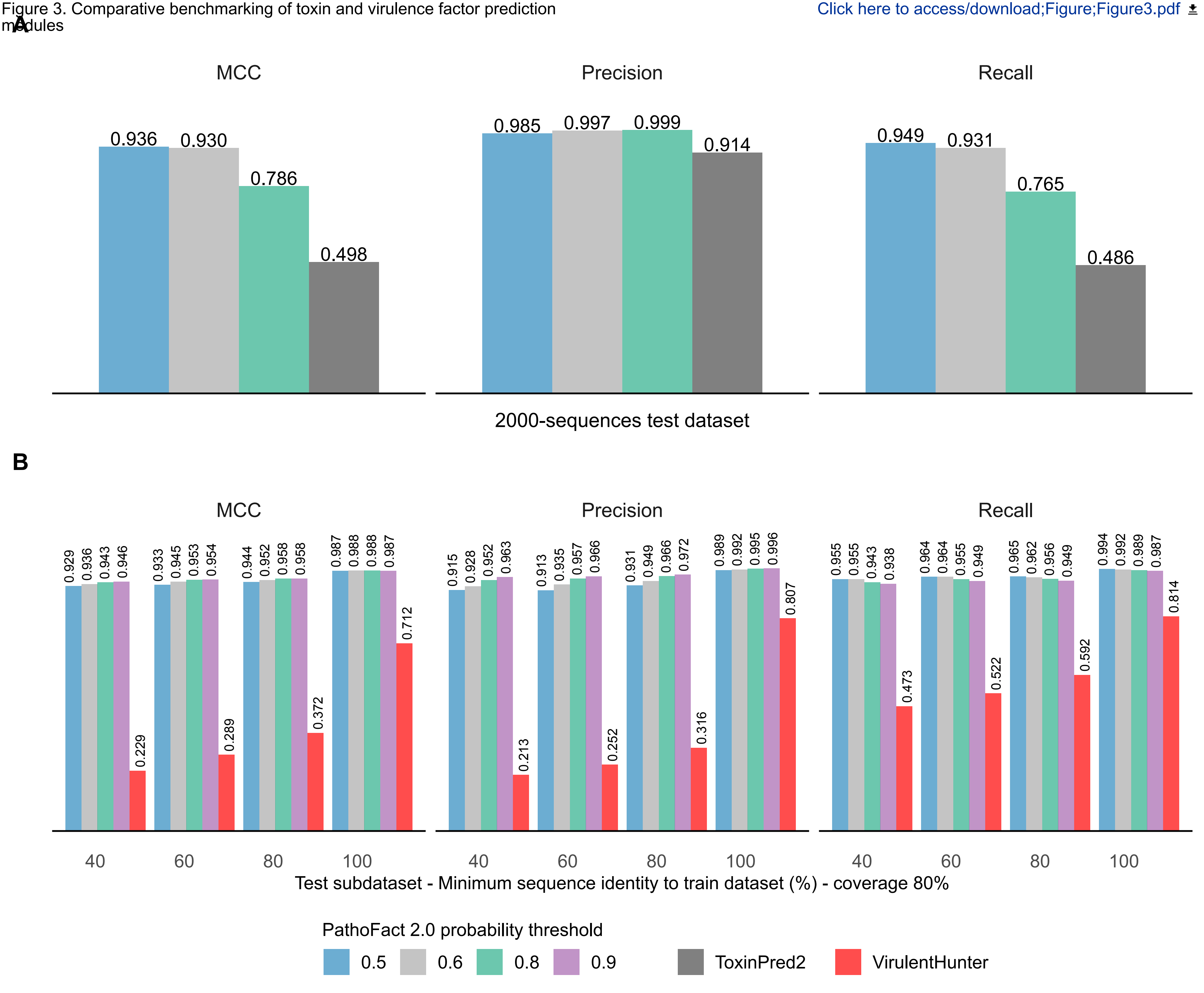

Figure 4. Comparative analysis of toxin-related and virulence factor profiles in non-pathogenic Escherichia coli strains

Click here to access/download;Figure;Figure4.pdf

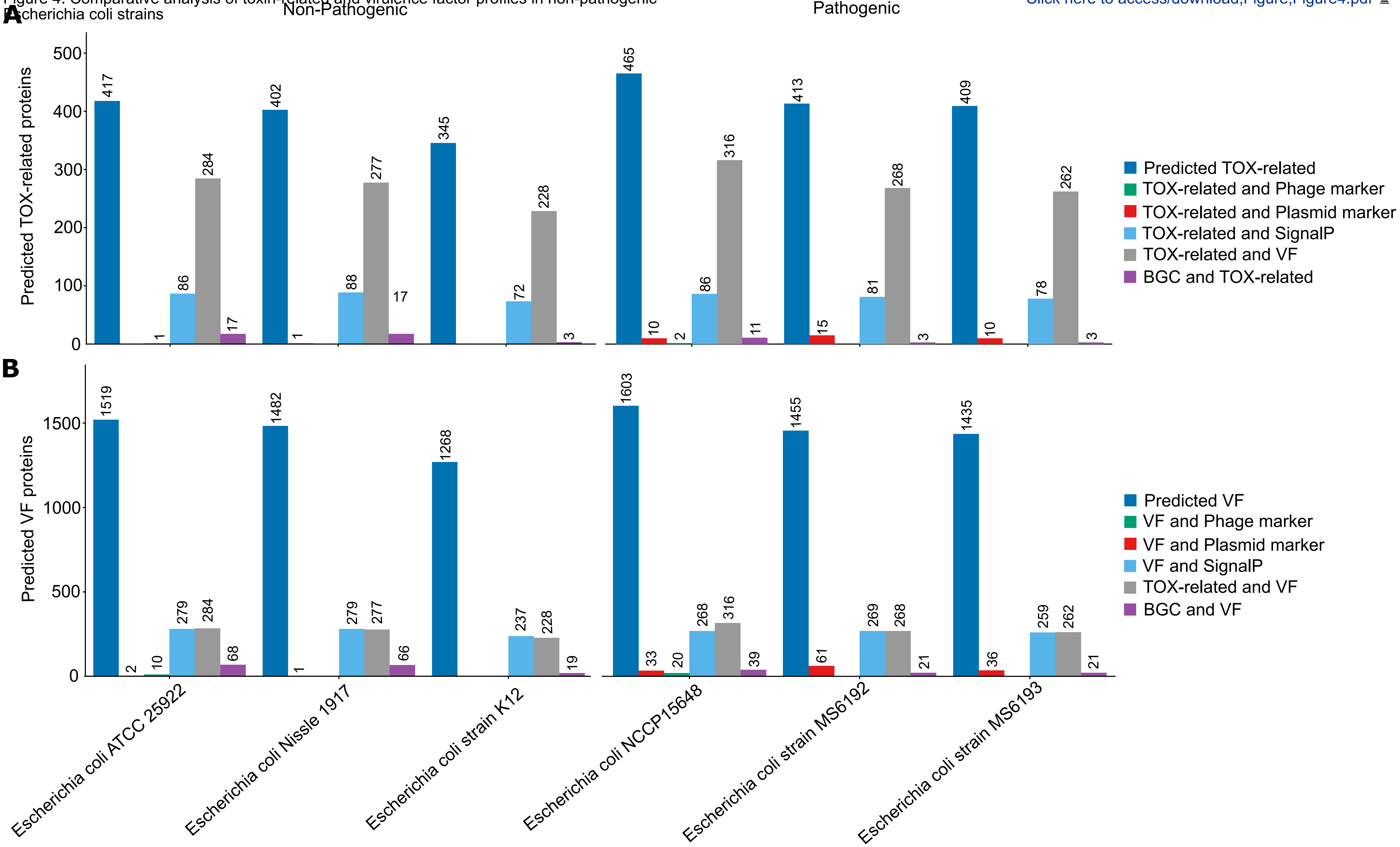

Figure 5. Comparative performance of PathoFact 2.0 versus PathoFact and metaVF in predicting virulence

Click here to access/download;Figure;Figure5.pdf

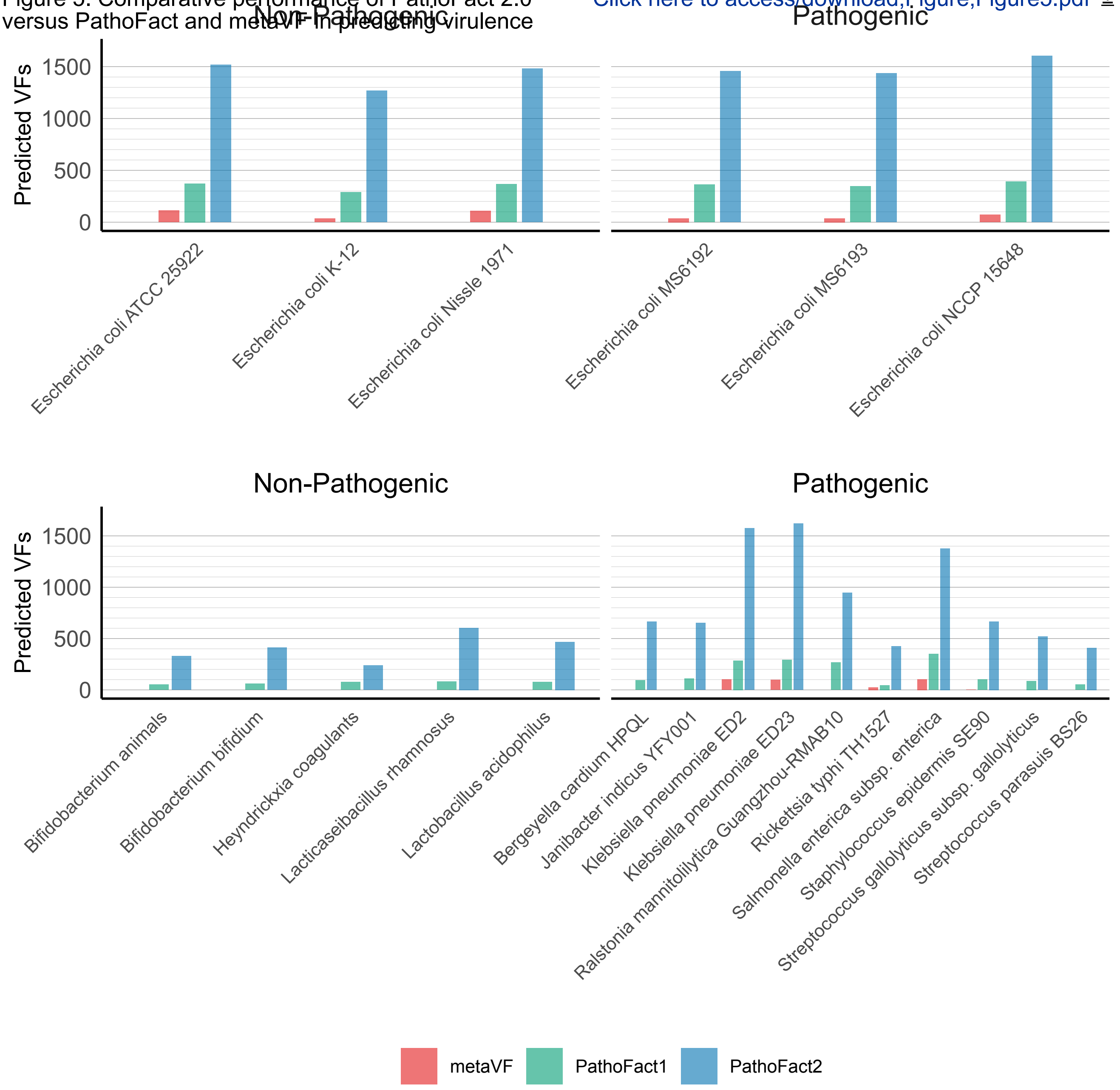

Supplementary Figure S1. Comparative analysis of toxin-related and virulence factor profiles in non-pathogenic and pathogenic

Click here to access/download
Supplementary Material
SupplementaryFigureS1.pdf

Supplementary Figure S2. Comparative performance of Pathofact 2.0 (blue) versus PathoFact1 (green) and metaVF (red) in

Click here to access/download
Supplementary Material
SupplementaryFigureS2.pdf

Supplementary Figure S3. Comparative performance of Pathofact 2.0 (blue) versus PathoFact (green) and metaVF (red) in predicting

Click here to access/download
Supplementary Material
SupplementaryFigureS3.pdf

Supplementary Figure S4. Performance comparison of PathoFact 2.0 versus PathoFact for ARG and toxin prediction in Escherichia

Click here to access/download
Supplementary Material
SupplementaryFigureS4.pdf

Supplementary Figure S5. Performance comparison of PathoFact 2.0 versus PathoFact for ARG and toxin prediction in bacterial

Click here to access/download
Supplementary Material
SupplementaryFigureS5.pdf

Supplementary Table S1. List of microorganisms non-pathogenic to humans and their total protein count obtained from the NCBI

Click here to access/download

Supplementary Material

SupplementaryTable1_ListOfMicroorganismsFromNCBI.tsv

Supplementary Table S2. Bacterial strains used in this study,
including their classification as pathogenic or non-pathogenic,

Click here to access/download

Supplementary Material

SupplementaryTable2_BacterialStrains_with_AccessionNumbers.tsv

Personal Cover Click here to access/download;Personal Cover;PathoFact2_CoverLetter_LCSB.pdf

Luxembourg Centre for Systems Biomedicine

UNIVERSITÉ DU LUXEMBOURG

Paul Wilmes
Professor of Systems Ecology
Luxembourg Centre for Systems Biomedicine
University of Luxembourg
7, avenue des Hauts-Fourneaux
L-4362 Esch-sur-Alzette
Luxembourg

30th October 2025

Dear Editor,

Please receive our manuscript entitled "**PathoFact 2.0: An Integrative Pipeline for Predicting Antimicrobial Resistance Genes, Virulence Factors, Toxins and Biosynthetic Gene Clusters in Metagenomes**" for consideration as an *Technical Note* in ***GigaScience***.

Microbiomes harbour complex communities of microorganisms, which can act as reservoirs for antimicrobial resistance genes (ARGs), virulence factors (VFs), and toxins, contributing to the emergence of infectious diseases and antibiotic resistance. ARGs and VFs can be disseminated through mobile genetic elements, and their accurate detection is crucial for understanding pathogen potential and informing treatment strategies. Additionally, biosynthetic gene clusters (BGCs) can impact virulence through secondary metabolites (Lau et al., 2004; Lybbert et al., 2020), further underscoring the need for integrated analysis tools.

PathoFact, introduced in 2020, was the first bioinformatics pipeline to predict ARGs, VFs, and toxins from metagenomic data (de Nies et al., 2021). PathoFact 2.0 expands this foundation with enhanced accuracy, new machine learning models for VFs and toxins, an expanded hidden Markov model database, and the added capability of BGC prediction, enabling a more integrated approach to pathogen profiling.

PathoFact 2.0 represents a groundbreaking advancement in microbial genomics, addressing a critical need for accurate and comprehensive tools to assess pathogenicity and antibiotic resistance across diverse microbiomes, including human, animal, and environmental ecosystems. Unlike its predecessor, PathoFact 2.0 is not a mere evolution but a transformative leap forward, incorporating novel methodologies and significantly enhanced algorithms that improve accuracy and scalability. Performance benchmarking demonstrates that PathoFact 2.0 achieves a notable increase in predictive precision and recall compared to PathoFact 1.0, ensuring more reliable detection of pathogenicity and resistance determinants. We believe this robust platform will be an indispensable resource for scientists working in microbial pathogenicity, antibiotic resistance, and epidemiology, playing a pivotal role in addressing global health challenges.

The authors confirm that this manuscript has not been previously published and is not under consideration by any other journal. The authors have no conflict of interest, financial or otherwise. Additionally, the manuscript has passed the Luxembourg Centre for Systems Biomedicine internal publication process designed to ensure FAIRness and reproducibility. The authors have approved the contents of this paper and have agreed to the submission policies of *GigaScience*.

Thank you very much in advance for considering our submission. We look forward to hearing from you.

Yours sincerely,

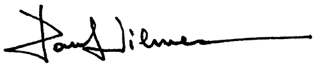

P. Wilmes, corresponding author
E-mail: paul.wilmes@uni.lu

LCSB-Luxembourg Centre for Systems Biomedicine
University of Luxembourg
7, avenue des Hauts-Fourneaux, L-4362, Esch-sur-Alzette
Luxembourg

T: +352 / 46 66 44-6188
F : +352 / 46 66 44-6949
E : lcsb@uni.lu
