# Supplementary material for: PathoFact 2.0: an integrative pipeline for the prediction of antimicrobial resistance genes, virulence factors, toxins and toxin-associated proteins, and biosynthetic gene clusters in metagenomes
Source: Gigascience. 2026 May 22;15:giag062. doi: 10.1093/gigascience/giag062 (PMC13224393; doi:10.1093/gigascience/giag062)
Supplement: giag062_GIGA-D-25-00455_revision_1 [file giag062_giga-d-25-00455_revision_1.pdf]

GigaScience

PathoFact 2.0: An Integrative Pipeline for the Prediction of Antimicrobial Resistance Genes, Virulence Factors, Toxins and Toxin-associated Proteins, and Biosynthetic Gene Clusters in Metagenomes

--Manuscript Draft--

**Manuscript Number:**	GIGA-D-25-00455R1
**Full Title:**	PathoFact 2.0: An Integrative Pipeline for the Prediction of Antimicrobial Resistance Genes, Virulence Factors, Toxins and Toxin-associated Proteins, and Biosynthetic Gene Clusters in Metagenomes
**Article Type:**	Technical Note
**Funding Information:**	Fondation du Pélican de Mie et Pierre Hippert-Faber (Pélican Grant) — Miss Júlia Ortís Sunyer<br>Fonds National de la Recherche Luxembourg (FNR CORE/23/BM/15886415) — Dr. Paul Wilmes<br>European Research Council (ERC-CoG 863664) — Dr. Paul Wilmes
**Abstract:**	Background<br>Antimicrobial resistance genes (ARG) and virulence factors (VFs) are central contributors to the global health crisis surrounding drug-resistant infections.<br>Findings<br>We introduce PathoFact 2.0, an enhanced pipeline for improved ARG, VF, toxin prediction, and biosynthetic gene clusters (BGC). Key improvements include an updated machine learning (ML) model for VF identification, expanded hidden Markov model profiles for VFs and toxin-associated proteins, a new ML model for toxin and toxin-associated proteins identification, and the integration of antiSMASH 7.0 for predicting biosynthetic gene clusters.<br>Conclusions<br>Our upgrades make PathoFact 2.0 a more powerful and user-friendly platform for predicting microbiome-based pathogenicity and resistance, providing a crucial tool for better understanding and addressing the challenges posed by antimicrobial resistance and infectious diseases.<br>PathoFact 2.0 is available at https://gitlab.com/uniluxembourg/lcsb/systems-ecology/pathofact2. It is compatible with Linux operating systems.
**Corresponding Author:**	Paul Wilmes<br>University of Luxembourg Luxembourg Centre for Systems Biomedicine: Universite du Luxembourg Luxembourg Centre for Systems Biomedicine<br>Esch-sur-Alzette, LUXEMBOURG
**Corresponding Author Secondary Information:**	
**Corresponding Author's Institution:**	University of Luxembourg Luxembourg Centre for Systems Biomedicine: Universite du Luxembourg Luxembourg Centre for Systems Biomedicine
**Corresponding Author's Secondary Institution:**	
**First Author:**	Luis Fernando Delgado, PhD
**First Author Secondary Information:**	
**Order of Authors:**	Luis Fernando Delgado, PhD
	Júlia Ortís Sunyer, MSc
	Cedric Christian Laczny, PhD
	Oskar Hickl, PhD

Powered by Editorial Manager® and ProduXion Manager® from Aries Systems Corporation

	Patrick May, PhD
	Paul Wilmes, PhD
**Order of Authors Secondary Information:**	
**Response to Reviewers:**	Reviewer reports:<br><br>We would like to thank all the reviewers for their thorough review of the manuscript. We appreciate their comments and believe that these contributions have significantly improved the paper. Below are our responses to each comment, along with the corresponding manuscript text for readability. In addition to the clean, updated manuscript submitted with our reviewer response, we have included an updated version that highlights our edits and additions to make the changes easy to identify.<br><br>Editor comments:<br><br>1. In addition, please register any new software application in the bio.tools and SciCrunch.org databases to receive RRID (Research Resource Identification Initiative ID) and biotoolsID identifiers, and include these in your manuscript. Computational workflows should be registered in workflowhub.eu and the DOIs cited in the relevant places in the manuscript. These will facilitate tracking, reproducibility and re-use of your tool.<br><br>R/ We thank you for this suggestion. We have now included the RRID, which is SCR_027650. We have also included the bio.tools ID: pathofact2 and the DOI for workflowhub.eu: 10.48546/workflowhub.workflow.2087.1<br><br>Reviewer #1:<br>The pipeline should be very useful on shotgun metagenomics data analysis.<br>Aside the ARGs and VFs, the features on signal peptides, toxin predictions, and BGCs in particular for specialised metabolites predictions, are welcome for detailed analysis and understanding of various transmission mechanisms.<br>I find the approaches very appealing and I think the pipeline could be welcomed by the community.<br><br>R/ We acknowledge and thank the reviewer for their recognition of our work and their overall positive feedback.<br><br>I only have some minor observations:<br>2. The Methods section should be placed next to the described methods. As it is, at the end of the manuscript, under Methods chapter you can only find Datasets, so a proper formatting of the Methods is required<br><br>R/ We thank the reviewer for the comment. We followed the journal's Technical Application Guidance: "For Technical Notes, this section is specific for including any additional methods used in the manuscript that are not part of the new work being described in the manuscript."<br>Nonetheless and to account for the reviewer's comment, we have expanded the Methods section accordingly (lines 494-558):<br><br>"Databases used for the PathoFact 2.0 Dataset Construction<br>SwissProt [31] is the expertly curated part of UniProtKB [66]. It offers high-quality protein sequences with detailed functional annotations, including keywords for pathogenesis, virulence, toxins, and antibiotic resistance.<br>VFDB [43], the Virulence Factor Database, is a comprehensive reference for curating information on virulence factors of bacterial pathogens.<br>T3DB [12], the toxin and toxin-target database, is a resource cataloguing thousands of toxins and their protein targets, with detailed mechanisms, structures, and toxicity data, including bacterial protein toxins.<br>DBETH [13], the database for bacterial exotoxins, is a specialised database of bacterial exotoxins pathogenic to humans, classified into 24 mechanistic and activity types from 26 bacterial genera.<br>TADB [36], the toxin-antitoxin database, is a repository of bacterial toxin-antitoxin loci across types I-VIII, including experimentally validated pairs, predicted loci, and

associations with mobile genetic elements.
SecReT6 [37] is a database containing known and predicted type VI secretion systems, including effectors, immunity proteins, regulators, and accessory proteins from bacterial genomes.
PAT [38] is the prokaryotic antimicrobial toxin database and contains a collection of antimicrobial toxins, including bacteriocins and effectors from secretion systems.

Clustering Parameters
MMseqs2 [32] is an ultra-fast open-source software suite for sensitive protein and nucleotide sequence searching and clustering, up to 10,000 times faster than BLAST while retaining comparable sensitivity. For dereplication, it clusters sequences at 100% identity and 100% coverage using the parameters -c 1.0 and --min-seq-id 1.0. It removes exact duplicates and retains representative sequences to generate non-redundant datasets. To create the test subsets, a coverage of 80% (-c 0.8) and identities ranging from 40 - 100% (--min-seq-id 0.4, --min-seq-id 0.6, --min-seq-id 0.8, --min-seq-id 1.0) were used, and then sequences with similarity higher than min-seq-id were removed for each test subset. In all cases, the cluster mode and coverage mode used were 0 (--cov-mode 0 --cluster-mode 0). When --cov-mode 0 is specified in combination with -c values ranging from 0.0 to 1.0, sequences are assigned to the same cluster only if the alignment spans at least a fraction c of the length of the longer sequence. According to the developers of MMSeq2, this coverage criterion is particularly suitable for clustering full-length protein sequences.
Protein Composition Features
Protein sequence composition features were extracted to represent each protein as fixed-length vectors derived from its primary amino acid sequence [35]. These included amino acid composition (AAC), dipeptide composition (DPC), composition (CTDC), transition (CTDT) and distribution (CTDD). AAC captures the relative frequency of each of the 20 amino acids in a sequence, whereas DPC captures the relative frequency of all adjacent amino-acid pairs (400 possible dipeptides). CTDC represents the percentage of amino acids belonging to each of three predefined groups (polar, neutral, hydrophobic) in the entire protein sequence. CTDT represents the percentage frequency with which a residue of one group is followed by a residue of a different group along the sequence. CTDD represents the distribution of each amino acid group, measuring the spatial position, where the first, 25%, 50%, 75% and 100% of the residues of a specific class are located.

Performance and Evaluation Metrics
To assess the models' performance, we used a confusion matrix comprising true positives (TP), true negatives (TN), false positives (FP), and false negatives (FN), computed on the test datasets described above. TP corresponds to truly positive instances correctly predicted as positive by the model, whereas TN corresponds to truly negative instances correctly predicted as negative. FP are truly negative instances incorrectly predicted as positive, and FN are truly positive instances incorrectly predicted as negative. From these values, we calculated the following metrics:

Accuracy = (TP + TN)/(TP+TN+FP+FN)

Precision = TP/(TP +FP)

Recall = TP/(TP+FN)

MCC =(TPTN-FPFN)/(TP+FP)(TP+FN)(TN+FP)(TN+FN)

HMM Profiles
Profile hidden Markov models are probabilistic models built from a multiple sequence alignment that encode, for each alignment position, the position-specific probabilities of residues and insertions/deletions, turning the alignment into a position-specific scoring system for detecting homologous sequences [67]. The FASTA files of conserved-domain multiple sequence alignments for each CDD [26] family (https://ftp.ncbi.nih.gov/pub/mmdb/cdd/fasta.tar.gz) were downloaded. Pyhmmer v0.10.14 [68] was used to obtain HMM profiles for each CDD family and to perform protein sequence searches against the CDD HMM family profiles.

3. there is a 70 blank pages buffer between References and supplementary data

Powered by Editorial Manager® and ProduXion Manager® from Aries Systems Corporation

R/ This occurred during the submission of the manuscript to Gigascience. This is therefore outside of our control.

4. could you add some future prospects in the manuscript? How well is it going to be maintained - I noticed the update are quite old.

R/ We thank the reviewer for this helpful comment. Updates are prompted by user-reported issues, newly identified errors, and the availability of enhanced reference datasets or prediction models. A new version of PathoFact is scheduled for the future, which will include two new modules, one for biocide and environmental pollutants resistance and another for heavy metal resistance genes.

5. I would also add some more details to the limitations. For instance it is clear that the pipeline is installable on Linux platforms, but did you considered making it available also for Apple silicon series? More and more researchers use this technology, and it works as good as the Linux distributions. I also tried an install on a M series Apple silicon, but unfortunately, most of the tools in the pipeline lead to multiple errors related to python versions (most of which are old), missing old dependencies versions, libraries, etc.

R/ We thank the reviewer for the comment. PathoFact 2.0 integrates several third-party tools whose compatibility with Apple Silicon (ARM architecture) is currently limited. Because these components are developed and maintained by external groups, addressing compatibility issues is outside the scope of PathoFact 2.0.
PathoFact 2.0 uses Mamba/Conda, with most packages from the Bioconda channel, which has been actively expanding support for ARM architecture. However, not all required dependencies are currently available or stable for Apple Silicon systems, leading to installation and runtime errors related to Python version mismatches and missing legacy libraries, as noted by the reviewer.
For these reasons, we have chosen to officially support Linux platforms, where all dependencies are stable, well-maintained, and widely adopted within the bioinformatics community. Once the required third-party tools and their dependencies are fully supported on ARM architectures through Bioconda or other channels, PathoFact 2.0 can be made compatible with Apple Silicon systems. It is important to note that metagenomic data is usually large in size. To efficiently run bioinformatic metagenomic workflows and to store the data and results, they are typically kept on dedicated servers or high-performance computing (HPC) clusters. Therefore, PathoFact 2.0 has been designed to be run on HPCs.

Reviewer #2:
The authors present the pipeline PathoFact 2.0, which combines external modules and machine learning algorithms in order to find genes that provoke antimicrobial resistance, virulence and toxicity. They present their work, the improvement from the previous version, as a Technical Note.
For what the authors say, it is the only pipeline available with those characteristics, which makes it clearly a relevant software and article. However, I believe the article requires refinement, as well as new tests that support the authors claims.

R/ We acknowledge and thank the reviewer for their recognition of our work and their overall positive feedback. We further thank the reviewer for their constructive criticisms, which have allowed us to strengthen the work and the resulting manuscript.

Refinements on the article
6. In the abstract, ARG is used as an abbreviation for Antimicrobial Resistance, not Antimicrobial Resistance Genes.

R/ We thank the reviewer for the comment. This has now been corrected (lines 20-21):

“Antimicrobial resistance genes (ARG) and virulence factors (VFs) are central contributors to the global health crisis surrounding drug-resistant infections.”

7. Overall, the article should be more clear in what the pipeline is made for. It mentions fungi, viral, etc… sequences (which might be found on a metagenomic sample, of

Powered by Editorial Manager® and ProduXion Manager® from Aries Systems Corporation

course), but to my understanding, all the tools and phenotypes searched for are mostly characteristic of bacteria.

R/ We thank the reviewer for this comment.
PathoFact 2.0 is explicitly designed for the analysis of metagenomic datasets, as stated in the title. In such datasets, prokaryotes (bacteria and archaea) represent the dominant fraction of organisms, and accordingly, most prediction modules in PathoFact 2.0 are optimised for bacterial proteins. The virulence factors, toxin-associated proteins, antimicrobial resistance genes, and secretion-related features targeted by the pipeline are therefore primarily bacterial in nature.

At the same time, metagenomic assemblies may contain sequences from viruses and eukaryotes (including fungi). For this reason, PathoFact 2.0 includes tools that can process non-bacterial sequences where appropriate. For example, protein prediction is performed using Prodigal-gv, which supports both prokaryotic and viral genomes; SignalP predicts signal peptides in proteins from archaea, bacteria, and eukaryotes; and antiSMASH includes dedicated modes for bacterial and fungal biosynthetic gene clusters. These components enable the pipeline to handle the mixed characteristics of metagenomic data.

We acknowledge that this duality, broad input acceptance versus primarily bacterial biological interpretation, was not sufficiently explicit in the current version of the manuscript.

We have added to the limitations of PathoFact 2.0 section the following paragraph (lines 471-473):

“PathoFact 2.0 is designed for metagenomic samples; most prediction modules and phenotypes are bacterial-centric. Virulence, toxin-associated, and antimicrobial resistance predictions are particularly interpreted in the context of human pathogens”

8. While the introduction offers a good resume of the genes of interest, there are some descriptions that are not particularly accurate. "Human, animal, and environmental microbiomes harbour commensal and pathogenic microorganisms, contributing to the emergence of infectious diseases" seems to say that commensal microorganisms contribute to the emergence of infectious diseases;

R/ We thank the reviewer for the comment; it has been revised. We have modified the paragraph (Lines 40-47):

“Microbiomes are highly complex and diverse ecological communities composed of bacteria, archaea, viruses, and microeukaryotes. These communities include both commensal microorganisms, which can contribute to host health, and pathogenic or opportunistic microorganisms that can cause disease under specific conditions. Microbial communities generally exist in synergistic relationships with their hosts, playing critical roles in maintaining physiological homeostasis and regulating immune function. However, disruption of this balanced microbial ecosystem, known as microbial dysbiosis, can impair normal body functions and has been associated with the development of various diseases, including cardiovascular diseases, cancers, and respiratory disorders [1].”

9. "ARGs are genetic elements that confer bacterial resistance to antibiotics, acquired via mutations or horizontal gene transfer." seems to say that antimicrobial resistance genes are acquired via mutation (they are not, there is a difference between resistance to antimicrobials provoked by mutations and by genes). I recommend a thorough rewriting of the 6 first paragraphs.

R/ We thank the reviewer for this relevant comment; It has been revised. The paragraph was modified (lines 50-51):

“ARGs are genetic elements that confer bacterial resistance to antibiotics. Many ARGs are found on mobile genetic elements (MGEs) and are therefore often horizontally transmitted [4]”.

10. The graphs in Figures 1 and 2 have different Y axes, which are also not shown. This is, to say it lightly, very misleading.

R/ We thank the reviewer for the comment; Figure 1 is a schema, so it seems the reviewer is referring to Figure 2 (A and B) in the previous manuscript's version (now Figure 4). Figure 4 has been updated to clearly show the Y-axis labels as figure titles.

11. Table 1 would be much more clear as a Figure. Table 1 cited in line 211 does not exist.

R/ We thank the reviewer for the comment. After trying out different figure types, we concluded that a table better represented our results than a figure. The reference to Table 1 has been removed (lines 179-180).

12. A short description of "dereplication" would help users lacking that knowledge.

R/ We thank the reviewer for the comment. In the first appearance of dereplication (both in figure 1 and pipeline structure section), and on several occasions in the manuscript, we included the following text: "proteins dereplicated by our tool to retain only non-redundant sequences (based on 100% identity and coverage). " Lines 131-132.

13. The description of the parameters of the machine learning modules are a copy-paste of the variables used by scikit-learn (lines 248 and 268). The description of the machine learning models should be clearer and more detailed, as well as not force the reader to go to the instructions of scikit-learn to check what is the meaning of those parameters.

R/ We thank the reviewer for the comment. As written in the manuscript (lines 170-172), we used XGBoost Python package (https://xgboost.readthedocs.io/en/stable/index.html) and the RandomForest (RF) Scikit-learn (version 1.5.2; [34]) to build our ML models. For the Random Forest model, the default hyperparameters provided by scikit-learn were used; for the XGBoost model, only the hyperparameters that differed from the defaults were explicitly specified. The parameter names were intentionally kept identical to those used in scikit-learn and the XGBoost Python package to ensure precision and reproducibility. These are standard hyperparameters of well-established models, and a detailed explanation of each is beyond the scope of this manuscript. The full definitions and usage of these parameters are clearly documented in the referenced XGBoost Python package as well as the scikit-learn documentation [34](RandomForest: https://scikit-learn.org/stable/modules/generated/sklearn.ensemble.RandomForestClassifier.html#sklearn.ensemble.RandomForestClassifier; XGBoost: https://xgboost.readthedocs.io/en/stable/parameter.html), to which the reader is explicitly directed.
In the manuscript lines 217-218:
"The toxin-associated ML model is a RF with 5-mer features (default hyperparameter setting, i.e, n_estimators=100, max_depth=None, min_samples_split=2)."

and lines 237-238:
"The VF model uses XGBoost with protein sequence composition features (learning_rate': 0.1, 'n_estimators': 2000)."

14. The authors analyze the performance of the models depending on the "probability" (a term that could definitely use a better introduction) using barplots. A standard to analyze the probability of a model is a ROC curve.

R/ We thank the reviewer for the comment. However, we respectfully point out that ROC curves are not always the most appropriate evaluation metric for highly imbalanced datasets, as is the case for virulence factor and toxin prediction.

In our study, the models produce a predicted class probability (referred to as "probability" in the manuscript) that reflects the model's likelihood that a given sequence belongs to the positive class.
ROC curves primarily assess the trade-off between sensitivity and specificity across

decision thresholds, but they can be misleading under strong class imbalance because a high true negative rate can inflate performance estimates even when positive class prediction is poor. We relied on the Matthews Correlation Coefficient (MCC), which is widely recognised as a robust and informative metric for imbalanced binary classification, as it accounts for all four confusion matrix components. As written in the manuscript (lines 280-284):

“The performance evaluation is 280 based on the Matthew correlation coefficient (MCC) and the precision (to reduce the number of false positive VF and toxin-associated predictions), taking into account the dataset imbalance (a higher number of “non-toxin” and “non-VF” sequences compared to “toxin-associated” and “VF” sequences in the test subsets).”

Our bar plot across confidence score thresholds shows how prediction quality (measured by MCC and precision) changes as probability cutoffs are applied. This approach directly addresses the practical use case of selecting an operating threshold that balances sensitivity and precision in metagenomic discovery settings, rather than providing a single aggregate curve.

We believe this evaluation strategy is appropriate given the data characteristics and the use case addressed in this study.

We have added the following text to the manuscript to clarify the probability concept (Lines 274-276):

“The VF and toxin-associated modules (Figure 1) were evaluated across different ML models-predicted probability thresholds, corresponding to the model's confidence in the positive class (VF or toxin-associated). Analyses were performed on the full test dataset and on subsets of the test dataset.”

Improvements
15. The claims of the authors about the machine learning models for VF and toxin prediction being more accurate than similarity models is, to my understanding, not proved in the article. If it is only compared to PathoFact, which was created with a dataset made years ago, the higher performance could easily be because of more complete datasets. A fair comparison of an improved performance should be done with the same dataset (PathoFact but with the dataset collected for PathoFact 2.0). Moreover, the results only show that PathoFact 2.0 predicts more toxins and virulence factors than PathoFact.

R/ We thank the reviewer for the comment. PathoFact 2.0 supports two distinct types of input, and the benchmarking strategy differs accordingly:

Protein sequence input
For protein-level predictions, we benchmarked the machine-learning models implemented in PathoFact 2.0 against state-of-the-art tools for the same task: VirulentHunter (for virulence factors), ToxinPred2 and CSM-toxin (for toxins). These comparisons were conducted on independent test datasets to evaluate model generalisation.
Our toxin model is designed to predict toxin-associated proteins, rather than toxin proteins alone. We have made this distinction clear in the title of the manuscript (i.e., Toxin-associated) and throughout the manuscript. To our knowledge, no existing model addresses this broader prediction task. For benchmarking, we compared our approach with a model that predicts only toxin proteins. To ensure a fair comparison, we limited the test dataset to toxin proteins for this evaluation. ToxinPred2 is widely used and designed for protein sequences, whereas ToxinPred3 is for peptide sequences; therefore, following the ToxinPred developers' recommendation, we used ToxinPred 2.0. In addition, we also used the same test dataset to benchmark the CSM-toxin.
As described in the Toxin-Associated Prediction Updates, our dataset intentionally includes both effector toxin proteins and toxin-associated proteins, such as antitoxins, regulatory factors, and accessory proteins (lines 192-203):
“It is essential to note that this dataset encompasses both effector toxin proteins and their associated proteins, including antitoxins, regulators, and accessory proteins. This

Powered by Editorial Manager® and ProduXion Manager® from Aries Systems Corporation

offers three main benefits: 1) Recent reports suggest that the same bacterial toxins can function as part of self-inhibiting toxin-antitoxin modules within one organism, while in another organism, they have evolved into toxin effectors that are injected into target cells [39,40]. 2) In bacteria, genes located in close proximity frequently exhibit functional associations, such as those co-transcribed within operons. A comprehensive toxin dataset, including both toxins and their associated proteins, facilitates the identification of novel toxins and related genes through their genomic context, referred to as "toxin islands." These islands may be involved in toxin biosynthesis, processing, or secretion, and may also confer immunity or facilitate horizontal gene transfer among bacterial populations. Notably, they are often rich in mobile genetic elements [41]. 3) A large database improves the performance of machine learning classification methods [42]".

Contig-level input
For contig-based analyses, the available tool landscape is much more limited. MetaVF and PathoFact1.0 are, to our knowledge, the only tools that directly support the prediction from assembled contigs. As explained in the manuscript (lines 394-396):

"To our knowledge, no other method is available to predict VFs from contig sequences and identify plasmid-encoded or prophage-associated VFs".

MetaVF relies primarily on similarity-based approaches, whereas PathoFact1.0 is indeed the previous version of our pipeline. The comparison at the contig level, therefore, serves two purposes: (i) to benchmark against the only available similarity-based method, and (ii) to demonstrate the improvements introduced in the updated PathoFact 2.0 framework. As explained in lines 400-402, MetaVF cannot detect VF in known pathogens, whereas PathoFact 2 can, demonstrating the limitations of the homology-based approach and the advantages of machine-learning-based models and approaches.

Following the reviewer's comment and to include more tools for comparison, we predicted protein sequences from the contigs and used them as input to VirulentHunter, Toxinpred2, and CSM-toxin, and included these results in Figure 7 and the new Figure 8. The following lines, 394-407, describe the performance of VirulentHunter:

"To our knowledge, no other method is available to predict VF from contig sequences and identify plasmid-encoded or prophage-associated VF. However, we included VirulentHunter in the comparison. Because VirulentHunter does not accept contig sequences as input, protein-coding genes were first predicted from contigs using Pyrodigal-gv and the resulting protein sequences were subsequently analysed. PathoFact 2.0 consistently detected a greater number of VFs than both PathoFact 1.0 and MetaVF (Figure 7, Supplementary Figures S2–S3). Notably, MetaVF failed to identify any VFs in five of the ten pathogenic reference strains tested, highlighting its limited ability to detect VFs and demonstrating the advantages of machine-learning-based models over homology-based approaches. While VirulentHunter produced substantially more hits than metaVF, yielding predictions comparable in number to PathoFact 2.0, PathoFact 2.0 generally identified more VFs overall (Figure 7). Exceptions were observed for non-pathogenic strains Bifidobacterium animalis, Bifidobacterium bifidum, Heyndrickxia coagulans, and for the pathogenic Ralstonia mannitolilytica strain Guangzhou-RMAB10, where VirulentHunter predicted slightly more VFs."

and lines 414-423, describing the performance of CSM-Toxin and Toxinpred2, were included in the manuscript:

"In addition, the PathoFact 2.0 toxin-associated module was compared to PathoFact 1.0 (Figure 8, Supplementary Figures S4–S5) as well as CSM-Toxin and ToxinPred2 (Figure 8). As CSM-Toxin and ToxinPred2 do not accept contig sequences as input, protein sequences were first predicted from contigs using Pyrodigal-gv. PathoFact 2.0 predicted more toxin-associated proteins than PathoFact 1.0, demonstrating improved detection capacity. Compared to external tools, CSM-Toxin identified substantially fewer toxins across the tested reference strains. In contrast, ToxinPred2 predicted a comparable number of toxins overall. However, for non-pathogenic strains

Powered by Editorial Manager® and ProduXion Manager® from Aries Systems Corporation

Bifidobacterium bifidum, Heyndrickxia coagulans, Lactobacillus acidophilus, and for pathogenic strains Streptococcus gallolyticus subsp. gallolyticus and Streptococcus parasuis B26, ToxinPred2 identified more predicted toxins than PathoFact 2.0."

The conclusions regarding the performance of the machine-learning models are therefore based on comparisons with contemporary ML-based approaches (VirulentHunter, ToxinPred2, CSM-Toxin) and MetaVF, not solely based on comparisons with the results of PathoFact.

16. The creation of the dataset, for training and most importantly for testing, is rather unclear and described all over the article. I recommend creating a section for it, to understand better the filtering, maybe a figure (could go in the supplementary material, if necessary), and include the amount of data in each test set. The authors seem to have put a lot of effort on the testing sets (including trying to avoid testing with the same data that the models are trained with) but it gets diluted in the article and, in consequence, the test results are difficult to evaluate.

R/We thank the reviewer for the helpful comment. To address concerns about clarity regarding dataset creation, we have now added a new section, Pipeline Updates, and two new figures, Figures 2 and 3, lines 148-151:

"We implemented thorough updates across all PathoFact modules. Notably, we developed two new machine-learning models: one to predict virulence factors and another to identify toxin-associated proteins. A schematic diagram of the dataset construction is shown in Figure 2. In the sections below, we detail the updates to each module."

and 266-268 in the manuscript:

"Figure 3 provides a schematic overview of the benchmarking datasets used for the VF and toxin-associated modules."

Figure 2 illustrates the construction of the toxin-associated, virulence factor, and non-toxin/non-VF datasets, and Figure 3 shows the generation of the different test subset datasets. These figures provide a clear overview of the filtering steps and subset creation, ensuring transparency in how the training and test data were defined and the benchmarking was performed.
In addition, we have included comprehensive performance data for the PathoFact 2.0 toxin-associated and virulence factor prediction modules in the Supplementary Tables. Supplementary Table S3 reports evaluation of the toxin-associated module, including class distributions (Negative, non-toxin; Positive, toxin-associated), confusion matrix counts alongside performance metrics—accuracy, precision, recall, F1 score, and Matthews correlation coefficient—across test subsets stratified by sequence similarity to the training set. Supplementary Table S4 provides corresponding data for the virulence factor module. To facilitate benchmarking against other tools, Supplementary Table S5 summarises virulence factor prediction performance for PathoFact 2.0 at various predicted probability cutoffs compared with VirulentHunter. At the same time, Supplementary Table S6 presents the performance of PathoFact 2.0 for predicting toxin proteins at different cutoffs, alongside that of ToxinPred2.
Together, these additions clarify the dataset construction, provide detailed information on the test sets, and present all relevant performance results, addressing the reviewer's concern regarding the evaluation of test results.

17. The results against VirulentHunter are impressive, outperforming a fine-tuned language model. While I do not doubt that the authors are thorough in their methods, such claims require more testing. Testing using external databases (not created by the authors, maybe the same used by VirulentHunter or other models validated experimentally such as pLM4VF) would support such claims.

R/ We thank the reviewer for the comment.

VirulentHunter is a sophisticated framework that not only predicts whether a protein is a virulence factor but also assigns it to specific functional categories. To our knowledge, this is the only tool that performs both tasks and is based on deep learning.

Powered by Editorial Manager® and ProduXion Manager® from Aries Systems Corporation

Furthermore, as reported by the VirulentHunter authors, VirulentHunter outperformed other virulence factor predictors (MP4 [49], VirulentPred 2.0 [50], and DeepVF [51]).

In addition, several other VF prediction tools either do not provide their training and test datasets or are no longer actively maintained (e.g., VF-Pred https://pubmed.ncbi.nlm.nih.gov/37979206/, DeepVF http://deepvf.erc.monash.edu/), limiting their suitability for benchmarking.

For these reasons, we selected VirulentHunter for benchmarking. However, this multi-class prediction is trained on a relatively small dataset, increasing the risk of overfitting. This behaviour is evident in Figure 5B, where VirulentHunter's performance decreases substantially as sequence similarity to the training set drops from 100% to 80%, indicating reduced generalisation to more divergent sequences.

We realised that the positive (virulence factor) datasets used by VirulentHunter and PathoFact 2.0 are highly similar and derived from the same sources. Using these datasets without removing similar sequences for external validation would therefore not provide an independent benchmark. Moreover, our negative dataset (i.e., "non-VF") includes proteins from known non-human pathogenic organisms (bacteria and viruses). We further curated the dataset (i.e., "non-VF") by removing any proteins predicted as virulence factors or toxins by PathoFact1. This approach reduces the likelihood of false negatives and broadens taxonomic coverage, including bacterial and viral proteins.

To ensure a fair and reliable comparison, we developed independent subset-based test datasets in which any sequence present in the VirulentHunter training set (100% identity, ≥80% coverage) was removed. This ensures that neither model was trained on sequences that appear in the test set. In addition, by stratifying test sets by decreasing levels of sequence similarity to the PathoFact 2.0 training data, we were able to explicitly assess model performance across increasingly divergent proteins. This study demonstrates that PathoFact 2.0 maintains consistent performance even when test sequences share no more than 40% similarity with the training set.
Regarding pLM4VF, we note that the publicly available experimentally validated test datasets for Gram-positive and Gram-negative bacteria contain only 922 sequences in total (461 positives and 461 controls). After removing sequences with greater than 99% similarity and coverage of 80% relative to the PathoFact 2.0 and VirulentHunter training datasets, only 73 VF sequences and 452 non-VF sequences remain.
We examined additional test datasets from VirulentPred2 (https://bioinfo.icgeb.res.in/virulent2/down.html) and the MSA-VF predictor (https://github.com/kimtaegyuu/MSA-VFpredictor/tree/main/data). After removing sequences with over 99% similarity and at least 80% coverage relative to the training datasets of PathoFact 2.0 and VirulentHunter, only 10 VF sequences and 561 non-VF sequences remained in VirulentPred2.0, while the MSA-VF predictor dataset retained 48 VF sequences and 561 non-VF sequences.
Although these datasets are valuable, their limited size prevents the subset-based similarity stratification needed for a robustness analysis comparable to the one reported, which utilises between 336 and 4,171 VF sequences and 3,904 to 8,106 non-VF sequences across various subset test datasets.
We believe that our evaluation strategy, based on independent, curated test datasets and explicit control of sequence similarity, provides a rigorous and conservative assessment of model generalisation. To account for this and the reviewer's concern, we have modified the paragraph to clarify this (lines 314-323):

"Since VirulentHunter and Pathofact 2.0 employ a similar method to generate the "VF dataset" for model training, we removed sequences from the Pathofact 2.0 test dataset that have 100% identity (≥80% coverage) to the VirulentHunter training dataset, resulting in a "clean VF test dataset" (Figure 3). This ensures that neither model used the test sequences for training. We applied the same test-subset approach described earlier: the subset datasets were created based on sequence similarity to the Pathofact 2.0 training dataset, with similarity ranging from 40% to 100% and 80% coverage of the "clean VF test dataset" (Figure 3). By stratifying test sets by decreasing sequence similarity to the training data, we explicitly evaluated model performance across progressively more divergent proteins. This study demonstrates that PathoFact 2.0 maintains consistent performance even when test sequences share no more than 40% similarity with the training set (Figure 5B)".

Lines 343 -345:

“As shown in Figure 5 (and in the Supplementary Tables S5 and S6), Pathofact 2.0 VF and toxin-associated modules exhibited higher MCC values across different test subsets compared to VirulentHunter, ToxinPred2 and CSM-toxin.”

18. The comparative on different bacterial strains gives more questions than answers. Are all those VF and toxins found on E. coli experimentally validated? How much overlap is there between pathogenic and non-pathogenic E. coli? Are all of the same type?

R/ We thank the reviewer for the comment. However, we think that a full biological interpretation of virulence factors (VFs) across diverse E. coli strains would constitute an independent study. The aim of our analysis is therefore not to exhaustively characterise virulence in E. coli, but to use this well-studied species as a representative example to illustrate the complexity of VF prediction and interpretation.

Not all virulence factors and toxins identified in our analysis have been experimentally validated in E. coli. This limitation is inherent to current VF databases, which typically include both experimentally confirmed factors and homologs inferred from sequence similarity. It is known that non-pathogenic E. coli strains can harbour genes annotated as virulence factors without causing disease, indicating that the presence of VF genes alone is insufficient to infer pathogenicity.

There is substantial overlap in predicted VF repertoires between pathogenic and non-pathogenic E. coli, and the strains analysed do not belong to a single pathotype. This overlap is well documented and reflects that many virulence-associated genes are variably distributed, are frequently located on mobile genetic elements (MGEs), and require specific genomic and regulatory contexts to contribute to disease.
This complexity is precisely one of the motivations for our study. As shown in Figure 6, pathogenic E. coli strains are enriched for virulence and toxin-associated proteins associated with MGEs, including plasmids and prophages, compared to non-pathogenic strains. These observations emphasise that distinguishing VFs relevant to infection requires more than standalone VF prediction.
PathoFact 2.0 addresses this challenge by integrating multiple layers of contextual information, including MGE association, secretion signals, and biosynthetic gene cluster localisation. This allows users to move beyond binary VF presence/absence predictions toward a more biologically meaningful interpretation of virulence potential.
Our results highlight that the relationship between predicted virulence factors and disease is more complex than often assumed, and that integrated, comparative approaches are essential for robust interpretation.
The following lines were added to the manuscript to highlight this aspect (lines 365-371):

“Figure 6 shows distinct differences in virulence and toxin-related profiles between pathogenic and non-pathogenic E. coli strains, especially regarding virulence- and toxin-associated proteins encoded on MGEs, such as plasmids and prophages. Nonetheless, analysis of individual VF predictions reveals considerable overlap in the number of VF genes detected across both pathogenic and non-pathogenic strains. This highlights that the presence of a VF gene is not a reliable marker of pathogenicity and emphasises the importance of considering genomic and functional context when assessing virulence potential”.

19. Overall, there is a good amount of work on this project, but the article still has a lot of unanswered questions. It is a bit unclear the strengths of PathoFact2, as well as its weaknesses (any model has). Could be its speed, could be having plenty of tools contained in a pipeline.

R/ We thank the reviewer for the comment.
PathoFact 2.0 represents a clear advance in metagenomic analysis by integrating the detection of antimicrobial resistance genes (ARGs), virulence factors (VFs), toxin-associated proteins, signal peptides, mobile genetic elements (MGEs), and biosynthetic gene clusters (BGCs) within a single, streamlined pipeline. Unlike many

existing tools that focus on individual aspects of pathogenicity, PathoFact 2.0 provides a comprehensive, multi-layered view that captures both gene presence and genomic context, improving interpretability and enabling a holistic assessment of microbial pathogenic potential. The pipeline's strengths, including its integrated design and computational efficiency, are illustrated in Figures 1, 4, 5 and Table 1.
We acknowledge several inherent limitations (lines 452-473). Non-pathogenic bacterial strains can also encode genes annotated as VFs or toxins, which makes PathoFact 2.0 most useful as an initial screening tool to identify candidates for further comparative analyses. While the pipeline provides probability scores for VF- or toxin-associated proteins, establishing a definitive link to pathogenicity requires experimental validation. PathoFact 2.0 does not directly classify specific VF or toxin types (e.g., adhesins or genotoxins), but it provides detailed annotations of conserved protein domains (CDD) to facilitate functional inference. Additionally, the pipeline is designed primarily for bacteria-enriched metagenomic data, with predictions interpreted particularly in the context of human pathogens.
Despite these expected limitations, PathoFact 2.0's integrated approach, speed, and multi-layered contextual analysis make it a versatile and powerful tool for studying microbial virulence and resistance in complex microbial communities. The following lines were added to the manuscript (lines 487-492):

"PathoFact 2.0 represents a major advance in metagenomic analysis by integrating the detection of ARGs, VFs, toxins and toxin-associated proteins, signal peptides, MGEs, and BGCs within a single, streamlined pipeline. Unlike existing tools that focus on individual aspects of pathogenicity, PathoFact 2.0 provides a comprehensive, multi-layered view that captures both gene presence and genomic context, improving interpretability and enabling a holistic assessment of microbial pathogenic potential".

20. I would also appreciate a better description of the report that PathoFact 2.0 produces. If its strength is the virulence and toxin prediction, more tests must be performed (as described above). This would be very beneficial for possible users of the model.

R/ We thank the reviewer for highlighting the need for a clearer description of the reports produced by PathoFact 2.0 and their utility for users. In response, we have added a dedicated paragraph to the manuscript (Lines 430–451; PathoFact 2.0 Output Structure):

"PathoFact 2.0 Output Structure
"PathoFact 2.0 creates a structured output directory that summarises predictions from all analysis modules, including VFs, toxin-associated proteins, ARGs, MGEs and BGCs. Each module generates dedicated result files corresponding to the underlying prediction tools (Supplementary File S1).
The primary summary file, combined_report.tsv, provides an integrated overview of high-confidence predictions across all modules in a tabular format. This table includes key information such as protein identifiers, bit scores (from HMM profiles), machine-learning prediction scores, and outputs from DeepARG, RGI, SignalP, GenoMad, and antiSMASH, thereby supporting downstream interpretation and candidate prioritisation. Proteins with prediction probabilities below user-defined thresholds but containing conserved domains identified by toxin-associated or VF HMM profiles are reported in ambiguous_TOX_hits_rep_prot.tsv and ambiguous_VF_hits_rep_prot.tsv. These lower-confidence candidates may warrant further investigation in comparative or experimental analyses.
High-confidence predictions are reported in amr_hits_rep_prot.tsv, TOX_hits_rep_prot.tsv, and VF_hits_rep_prot.tsv, which summarise features exceeding user-defined probability thresholds and include protein identifiers, bit scores, machine-learning predictions, signal peptide predictions, and genomic context information, such as association with prophages or plasmids identified by GenoMad.
In addition, PathoFact 2.0 generates a dedicated Group_of_sequence directory containing FASTA files of representative protein sequences grouped by functional category (VFs, toxin-associated proteins, antimicrobial resistance genes, and combined hits), together with conserved domain (CDD) annotation tables for predicted VFs and toxin-associated proteins. These files are designed to facilitate downstream analyses, including comparative genomics and functional characterisation."

Powered by Editorial Manager® and ProduXion Manager® from Aries Systems Corporation

This paragraph clearly describes the organisation and content of the PathoFact 2.0 output directory. We have also included a supplementary figure that illustrates the output directory structure (Supplementary File S1). This section explicitly details the key output files generated for virulence factor, toxin, and antimicrobial resistance predictions. It describes the integrated combined_report.tsv and explains the separation of high-confidence and ambiguous predictions based on user-defined probability thresholds.
We further clarify that PathoFact 2.0 generates a dedicated Group_of_sequence directory. This directory contains FASTA files of representative protein sequences grouped by functional category: virulence factors, toxins, and antimicrobial resistance genes. It also includes conserved domain (CDD) annotation tables for predicted virulence factors and toxins. As described in the revised manuscript, the pipeline provides high-confidence prediction tables and lower-confidence candidate lists that are supported by conserved-domain evidence. It also offers integrated contextual information, including secretion signals and associations with mobile genetic elements. This structured reporting enables efficient interpretation of results, candidate prioritisation, and downstream analyses, including comparative genomics and functional characterisation. Together with the output directory schema provided in Supplementary File S1, these additions clearly demonstrate the strengths of PathoFact 2.0's reporting and substantially enhance its usability for the community. It thus represents a strength of PathoFact 2.0 in addition to the strengths highlighted in our response to the comment 19.

21. Moreover, in a more technical note, I recommend the authors to add a test sample for easy testing of the model in their repository.

R/ We thank the reviewer for the comment. As specified in the Data Availability section of the original and revised manuscripts, the datasets for training and testing the VF and Toxin modules are available on Zenodo (https://zenodo.org/records/17593961) because they are too large to store on GitLab. Additionally, in our GitLab repository, we have included a sample test to facilitate easy pipeline testing (to confirm that the pipeline works).

Reviewer #3:

R/ We thank Reviewer 3 for their careful review and constructive criticisms. Their comments have helped us improve the methodological clarity and robustness of the manuscript.

22. *Several methods are available to predict ARGs, VFs, Toxins, and Biosynthetic Gene Clusters. However, the authors selected only a few tools to benchmark PathoFact 2.0. I find this point lacking in the manuscript. To be useful to the scientific community, a more rigorous performance evaluation is needed.

R/ We thank the reviewer for this comment. PathoFact 2.0 is an integrated pipeline, rather than a single prediction model. In this update, we introduce two new prediction modules for virulence factors and toxin-associated proteins, alongside existing modules for antimicrobial resistance genes (ARGs), biosynthetic gene clusters, signal peptides, and functional annotation. The pipeline supports both protein and contig-level inputs.
We think benchmarking against all available tools is not feasible, as it would require evaluating multiple tools for each functional category and input type, resulting in an impractical number of comparisons. In addition, few tools support contig-level input, and several do not provide accessible training or test datasets, limiting the ability to conduct fair and unbiased benchmarking. We therefore selected representative, widely used and state-of-the-art tools for evaluation, which are VirulentHunter and metaVF for VFs and ToxinPred2 for toxins. Alongside Toxinpred2, we have now included a new tool for benchmarking toxin prediction, CSM-toxin.
PathoFact 2.0 further extends existing approaches by predicting toxin-associated proteins, rather than toxin proteins alone, consistent with current strategies for virulence factor prediction (e.g, regulatory proteins are also included). The principal contribution of PathoFact 2.0 is its integrative design, enabling simultaneous prediction of ARGs, virulence factors, toxin-associated proteins, biosynthetic gene clusters, and signal peptides, with results summarised in coherent output tables. This integration

	supports comprehensive pathogenicity profiling of genomic and metagenomic data.<br>In addition, as written in the manuscript (lines 104-109):<br><br>"PathoFact, a pipeline first introduced in 2020, integrates ARG, VF, and bacterial toxin prediction from metagenomic data into a single tool [22]. Since the publication of PathoFact, several tools have been implemented to predict ARGs, VFs, and bacterial toxins [23–25]. Only one tool, HyperVR [24], has attempted to predict them simultaneously. However, HyperVR's repository is no longer available online, and the Zenodo archive from its original submission lacks the necessary databases, hence rendering it unusable."<br><br>Specifically, for VFs and Toxins, PathoFact 2.0 supports two distinct types of input, and our benchmarking strategy reflects this distinction:<br>1. Protein sequence input<br>For protein-level predictions, we benchmarked the machine-learning ...
**Additional Information:**	
**Question**	**Response**
Are you submitting this manuscript to a special series or article collection?	No
**Experimental design and statistics**<br><br>Full details of the experimental design and statistical methods used should be given in the Methods section, as detailed in our Minimum Standards Reporting Checklist. Information essential to interpreting the data presented should be made available in the figure legends.<br><br>Have you included all the information requested in your manuscript?	Yes
**Resources**<br><br>A description of all resources used, including antibodies, cell lines, animals and software tools, with enough information to allow them to be uniquely identified, should be included in the Methods section. Authors are strongly encouraged to cite Research Resource Identifiers (RRIDs) for antibodies, model organisms and tools, where possible.<br><br>Have you included the information requested as detailed in our Minimum Standards Reporting Checklist?	Yes

Powered by Editorial Manager® and ProduXion Manager® from Aries Systems Corporation

**Availability of data and materials**<br><br>All datasets and code on which the conclusions of the paper rely must be either included in your submission or deposited in publicly available repositories (where available and ethically appropriate), referencing such data using a unique identifier in the references and in the "Availability of Data and Materials" section of your manuscript.<br><br>Have you have met the above requirement as detailed in our Minimum Standards Reporting Checklist?	Yes
GigaScience has policies and guidelines in place for the use of generative AI-writing tools such as ChatGPT. If you have used such writing tools to assist with writing the manuscript this must be declared and cited in the text. Authors should not list AI-writing tools and other AI-assisted technologies as an author or co-author and should acknowledge that they are fully responsible for text generated or refined by AI-writing tools.<p><br><br>A summary of use (particularly in the introduction or among methods) needs to be included at the end of the paper, and the outputs should also be included as a supplementary file hosted in GigaDB or other open repositories. Please read our guidelines for more information. <p><br><br>By submitting to GigaScience, you are aware of the journal's AI-writing tools policy, and if you have declared use of such tools below, you have acknowledged this where appropriate in your manuscript and have made a summary of use and outputs available. <p><br><b>AI-assisted writing tools have been used in the preparation of this manuscript?	No

Revised Manuscript

Click here to access/download;Manuscript;PathoFact_resubmission_revw.p

PathoFact 2.0: An Integrative Pipeline for the Prediction of Antimicrobial Resistance Genes, Virulence Factors, Toxins and Toxin-associated Proteins, and Biosynthetic Gene Clusters in Metagenomes

Luis F. Delgado (luis.delgado@uni.lu)*[1][0000-0001-7850-5285], Júlia Ortís Sunyer (julia.ortissunyer@uni.lu)*[1] [0000-0002-2714-7067], Cedric C. Laczny (cedric.laczny@uni.lu)[1][0000-0002-1100-1282], Oskar Hickl (oskar.hickl@lih.lu)[1][0000-0001-9959-8767], Patrick May (patrick.may@uni.lu)[1][0000-0001-8698-3770] & Paul Wilmes (paul.wilmes@uni.lu)[1,2][0000-0002-6478-2924]

1. Luxembourg Centre for Systems Biomedicine, University of Luxembourg, Esch-sur-Alzette, Luxembourg
2. Department of Life Sciences and Medicine, Faculty of Science, Technology and Medicine, University of Luxembourg, Esch-sur-Alzette, Luxembourg

Contact: Paul Wilmes (paul.wilmes@uni.lu)

*These authors contributed equally

Abstract

Background

Antimicrobial resistance genes (ARG) and virulence factors (VFs) are central contributors to the global health crisis surrounding drug-resistant infections.

Findings

We introduce PathoFact 2.0, an enhanced pipeline for improved ARG, VF, toxin prediction, and biosynthetic gene clusters (BGC). Key improvements include an updated machine learning (ML) model for VF identification, expanded hidden Markov model profiles for VFs and toxin-associated proteins, a new ML model for toxin and toxin-associated proteins identification, and the integration of antiSMASH 7.0 for predicting biosynthetic gene clusters.

Conclusions

Our upgrades make PathoFact 2.0 a more powerful and user-friendly platform for predicting microbiome-based pathogenicity and resistance, providing a crucial tool for better understanding and addressing the challenges posed by antimicrobial resistance and infectious diseases.

PathoFact 2.0 is available at https://gitlab.com/uniluxembourg/lcsb/systems-ecology/pathofact2. It is compatible with Linux operating systems.

Keywords

Antimicrobial resistance genes, virulence factors, toxin-associated proteins, biosynthetic gene clusters, metagenomes, machine learning

Findings

Introduction

Microbiomes are highly complex and diverse ecological communities composed of bacteria, archaea, viruses, and microeukaryotes. These communities include both commensal microorganisms, which can contribute to host health, and pathogenic or opportunistic microorganisms that can cause disease under specific conditions. Microbial communities generally exist in synergistic relationships with their hosts, playing critical roles in maintaining physiological homeostasis and regulating immune function. However, disruption of this balanced microbial ecosystem, known as microbial dysbiosis, can impair normal body functions and has been associated with the development of various diseases, including cardiovascular diseases, cancers, and respiratory disorders [1].

Moreover, these microorganisms play a critical role in the development of antibiotic-resistant infections through the presence of antimicrobial resistance genes (ARGs) and virulence factors (VFs)[2,3]. ARGs are genetic elements that confer bacterial resistance to antibiotics. Many ARGs are encoded on mobile genetic elements (MGEs) and are therefore often horizontally transmitted [4]. ARGs can be divided into categories based on the antibiotics to which they confer resistance [5]. The Antibiotic Resistance Ontology (ARO) contains information on ARGs, the mutations that cause them, their products, mechanisms, associated phenotypes, antibiotics, and molecular targets [6].

Bacterial pathogens use specific genes, known as VFs, to attach to and invade host tissues, survive within the host, spread, and ultimately cause damage. The harm inflicted can vary, ranging from minor disruptions to severe or even fatal outcomes [7]. VFs can be classified as secretory, membrane-associated, or cytosolic. Cytosolic virulence factors promote rapid adaptive shifts in bacterial metabolism, physiology, and morphology, enhancing survival and proliferation within the host. Membrane-associated factors contribute to bacterial adhesion and immune evasion at the host-cell interface. Secretory factors constitute a critical part of the bacterial armamentarium, enabling bacteria to counteract innate and adaptive immune defences. Secretory VFs often exhibit synergistic effects and induce cytotoxicity in host cells [8]. VFs are often located on MGEs, such as transposons, plasmids and phages, facilitating their transfer between bacterial cells [9,10].

Bacterial toxins play a crucial role in the development of infectious diseases, alongside various virulence factors employed by pathogens. They disrupt host processes and manipulate immune responses. Some toxins impair protein synthesis, destroy blood cells, or affect the nervous system. Bacterial toxins can be divided into two main categories: cell-associated endotoxins and extracellular, diffusible exotoxins. Endotoxins, such as lipopolysaccharides, are found in the outer membranes of Gram-negative bacteria and serve as potent inflammatory mediators that can induce systemic toxicity and septic shock in infected hosts [11]. Exotoxins, which are typically polypeptides and proteins, can stimulate a range of host responses by either acting directly on cell receptors or through enzymatic modulation [12,13]. Many bacterial toxins are secreted proteins that require signal peptides. Signal peptides are short amino acid sequences at the N-terminus of proteins that direct them to specific cellular compartments, such as the periplasm [14,15].

Biosynthetic gene clusters (BGCs) are responsible for synthesising specialised metabolites (SMs). Some SMs can increase pathogenicity; for example, clinical isolates of *Pseudomonas aeruginosa* produce siderophores, rhamnolipids, quinolones, and phenazines [16]. Similarly, *Burkholderia* strains produce virulence factors, such as toxoflavin from *Burkholderia glumae* [17]. Notably, pyocyanin, a redox-active phenazine produced by *Pseudomonas aeruginosa*, plays a crucial role as a virulence factor in lung infections [18].

The threat that ARGs, VFs and toxins pose to human health is significant. The United Nations have identified antimicrobial resistance as a global threat, with estimates attributing 1.27 million deaths annually to drug-resistant infections, potentially rising to 10 million by 2050 if unaddressed [19,20]. Thus, accurately predicting potential ARG and VF profiles is essential for early intervention, enabling anticipation of infection severity, improving treatment strategies, and ultimately reducing mortality rates from disease-causing pathogens.

Predicting and annotating ARGs, VFs, and toxins is challenging due to limited well-annotated data [21] and complex mechanisms involving gene transfer, mutations, and multifactorial interactions. Traditional annotation methods, which rely on sequence similarity, may overlook novel ARGs, VFs and toxins. In contrast, machine learning offers robust solutions through pattern recognition, enabling accurate predictions even with limited training data.

An integrated bioinformatics pipeline enhances analysis by simultaneously examining ARGs, VFs, toxins, signal peptides, and BGCs from a single metagenomic sample. This comprehensive approach provides a more complete view of bacterial pathogenicity by capturing the full spectrum of virulence mechanisms, including antimicrobial resistance, toxin production, and secondary metabolic capabilities. This holistic analysis improves insights into pathogenicity and resistance, streamlines workflows, and simplifies data interpretation.

PathoFact, a pipeline first introduced in 2020, integrates ARG, VF, and bacterial toxin prediction from metagenomic data into a single tool [22]. Since the publication of PathoFact, several tools have been implemented to predict ARGs, VFs, and bacterial toxins [23–25]. Only one tool, HyperVR [24], has attempted to predict them simultaneously, analogous to PathoFact. However, HyperVR's repository is no longer available online, and the Zenodo archive from its original submission lacks the necessary databases, hence rendering it unusable.

Here, we present PathoFact 2.0 (Figure 1). It enhances the previous version by supporting protein sequences or contigs as input and by updating the ML VF model and the hidden Markov model (HMM) profiles of the conserved domain databases (CDD) [26] for VF and toxin-associated protein annotation. We have also introduced the ability to predict BGCs using antiSMASH 7.0 [27]. antiSMASH is a tool that identifies, annotates, and analyses secondary metabolite BGCs across genomes.

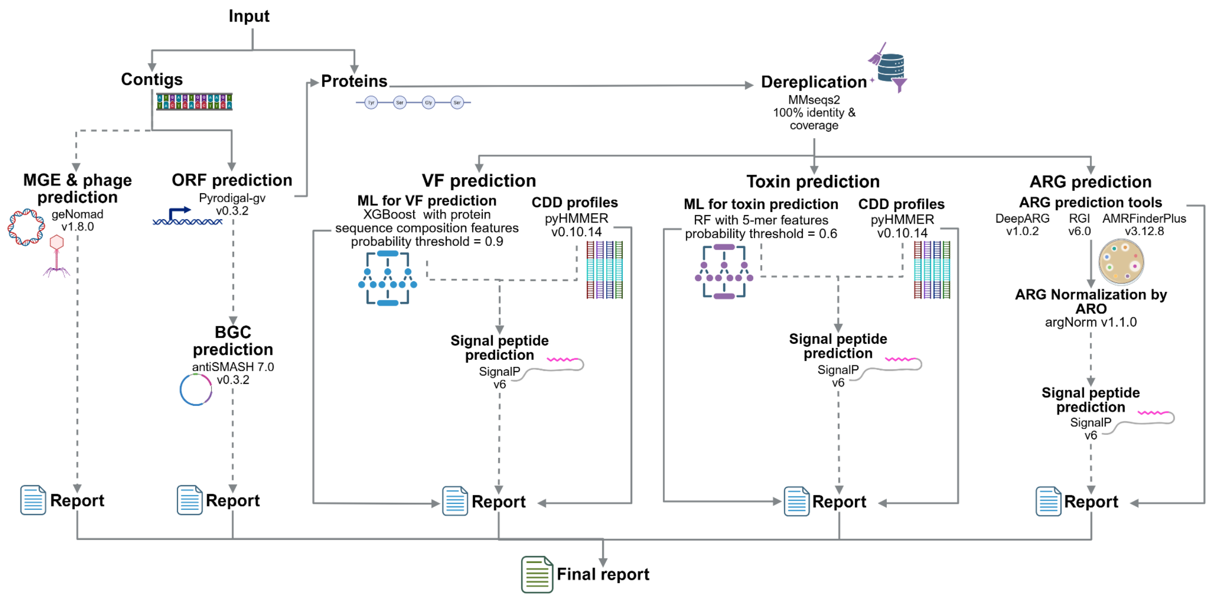

Figure 1. Schematic representation of PathoFact 2.0. Solid lines denote core modules, while dotted lines indicate optional user selection. The input is a FASTA file with either contig or protein sequences.

If the input is a FASTA file containing contigs, open reading frames (ORFs) are predicted using Pyrodigal-gv. If the biosynthetic gene cluster (BGC) option is selected, antiSMASH will use the GBK file for BGC prediction. GeNomad is used for MGE and phage prediction, producing a FASTA file of protein sequences. Protein sequences are dereplicated using MMseqs2 to retain non-redundant sequences (based on 100% identity and coverage). After dereplication, antimicrobial resistance genes (ARGs), virulence factors (VFs) and toxins and their associated proteins are predicted using their respective modules. SignalP predicts the presence of signal peptides and their cleavage sites in proteins from archaea, bacteria and eukarya. Individual reports are generated for each module, and an integrated report is produced that combines all module reports.

Pipeline Structure

Unlike version 1.0, which supports only contigs, PathoFact 2.0 accepts nucleotide sequences of contigs and protein sequence FASTA files, with proteins dereplicated by our tool to retain only non-redundant sequences (based on 100% identity and coverage). For contig-based inputs, open reading frames are predicted using Pyrodigal-gv (version 0.3.2; [28,29]; https://github.com/althonos/pyrodigal-gv), a Python library that binds to Prodigal [22], followed by the detection of MGEs and phages using geNomad (version 1.8.0; [29]). GeNomad processes only nucleotide sequences; therefore, MGEs and phages are not detected in protein sequence inputs. Based on user configuration, the pipeline then analyses the processed sequences using the BGC, ARG, VF, toxin-associated, and BGC (via antiSMASH) prediction modules. The information is compiled into individual module reports and an integrated report, also incorporating details from SignalP and geNomad (Figure 1). Additionally, PathoFact 2.0 generates a FASTA file of proteins identified as ARGs, VFs, or toxin-associated proteins.

Pipeline Installation

PathoFact 2.0 is implemented using Snakemake (version 7.25.0; [30]). An installation script simplifies the setup by installing the required software and downloading databases with a single command. PathoFact 2.0 is open-source (GNU General License v3.0 or later) and freely available at https://gitlab.com/uniluxembourg/lcsb/systems-ecology/pathofact2, where detailed instructions for pipeline installation, configuration, and output are provided.

Pipeline Updates

We implemented thorough updates across all PathoFact modules. Notably, we developed two new machine-learning models: one to predict virulence factors and another to identify toxin-associated proteins. A schematic diagram of the construction of the training and test datasets is shown in Figure 2. In the sections below, we detail the updates to each module.

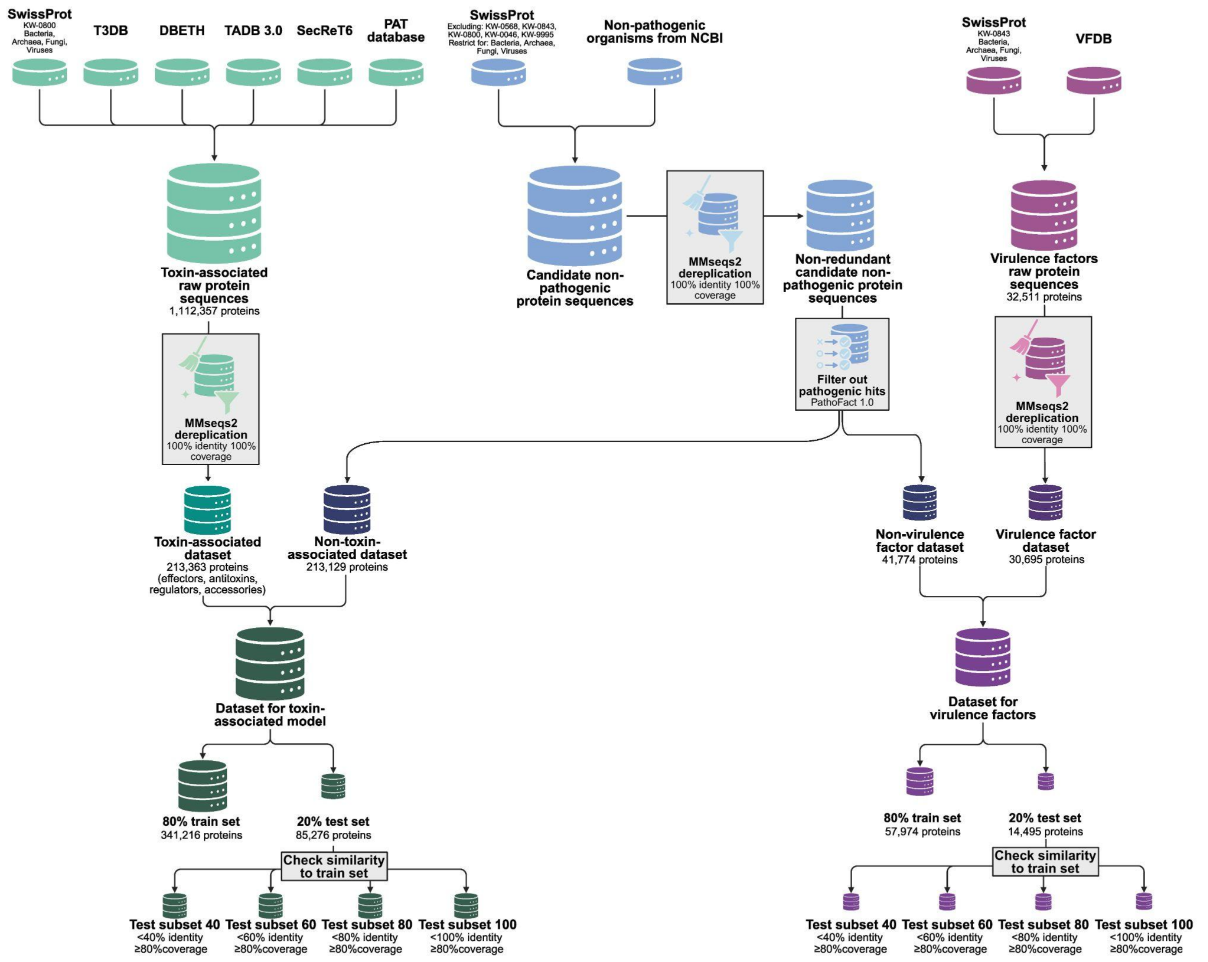

Figure 2. Schematic representation of the datasets used for PathoFact 2.0 toxin-associated and virulence factors modules training and testing. Shades of green represent the toxin-associated datasets; shades of blue represent the non-pathogenic dataset; and shades of purple represent the virulence factor datasets.

Generalities about the "non-pathogenic" dataset and the machine learning training setup

The "non-pathogenic" dataset for the ML models was constructed by selecting SwissProt [31] sequences lacking ARG, VF, and toxin keywords [KW-0568 (pathogenesis-related protein), KW-0843 (virulence), KW-0800 (toxin), KW-0046 (antibiotic resistance), KW-9995 (disease)] and limited to bacteria (taxonomy_id 2), archaea (taxonomy_id 2157), fungi (taxonomy_id 4751), and viruses (taxonomy_id 10239) (Figure 2). Additionally, proteins from non-pathogenic organisms to humans (Supplementary Table S1; Figure 2) were included from NCBI. MMseqs2 (version 15.6f452; [32]) was used to obtain a set of non-redundant (clustered at 100% identity and coverage) protein sequences (Figure 2).

ML models were trained using 80% of the sequences for training and 20% of the sequences for testing. The Synthetic Minority Oversampling Technique (SMOTE) was employed to address the dataset's imbalance [33]. Using the XGBoost Python package (https://xgboost.readthedocs.io/en/stable/index.html) and the RandomForest (RF) Scikit-learn (version 1.5.2; [34]), several ML models were trained and tested using k-mers (k=3 to 8) or protein sequence composition features (amino acid composition (AAC), dipeptide composition (DPC), composition (CTDC), transition (CTDT), and distribution (CTDD) [35]) as features. Hyperparameter

optimisation was performed, using a 5-fold cross-validation with HalvingGridSearchCV from scikit-learn [34]. The best-performing model was selected based on the Matthews correlation coefficient (MCC) score.

Toxin-Associated Protein Prediction Updates

Compared to version 1.0, the toxin prediction module now employs a ML model instead of a purely alignment-based bit score threshold. Curated training data was obtained from SwissProt [31], filtered for bacterial (taxonomy_id 2), archaeal (taxonomy_id 2157), fungal (taxonomy_id 4751) and viral (taxonomy_id 10239) toxin sequences (KW-0800, toxin)(Figure 2). The dataset was supplemented with entries from toxin-specific databases such as the Toxin Exposome Database (T3DB) [12], which catalogues bacterial protein toxins; the Database for Bacterial ExoToxins (DBETH) [13]; TADB version 3.0, which includes protein sequences of bacterial toxin–antitoxin (TA) pairs from types I to VIII [36]; sequences from SecReT6 [37], encompassing T6SS gene cluster components, T6SS regulator (T6SR), accessory proteins (T6SA), effectors (T6SE), and immunity proteins (T6SI); and the prokaryotic antimicrobial toxins (PAT) database [38] (Figure 2).

MMseqs2 (version 15.6f452; [32]) was used to dereplicate the dataset of 1,112,357 protein sequences (100% identity and coverage), yielding 213,363 unique protein sequences, corresponding to the "toxin-associated" dataset (Figure 2). It is essential to note that this dataset encompasses both effector toxin proteins and their associated proteins, including antitoxins, regulators, and accessory proteins. This offers three main benefits: 1) Recent reports suggest that the same bacterial toxins can function as part of self-inhibiting toxin-antitoxin modules within one organism, while in another organism, they have evolved into toxin effectors that are injected into target cells [39,40]. 2) In bacteria, genes located in close proximity frequently exhibit functional associations, such as those co-transcribed within operons. A comprehensive toxin dataset, including both toxins and their associated proteins, facilitates the identification of novel toxins and related genes through their genomic context, referred to as "toxin islands." These islands may be involved in toxin biosynthesis, processing, or secretion, and may also confer immunity or facilitate horizontal gene transfer among bacterial populations. Notably, they are often rich in mobile genetic elements [41]. 3) A large database improves the performance of machine learning classification methods [42].

HMM profiles were built using the conserved-domain FASTA files (https://ftp.ncbi.nih.gov/pub/mmdb/cdd/fasta.tar.gz) from CDD [26]. The 213,363 unique protein sequences in the "toxin-associated" dataset were annotated using the CDD HMM profiles. Those with a bitscore above 25 were chosen as HMM profiles for toxin and toxin-associated protein annotation and incorporated into Pathofact 2.0 for protein annotation.

Although there is no standard for creating negative datasets, they play a crucial role in influencing model performance. Therefore, to improve the quality of our "non-toxin" dataset, potential ARGs, VFs (with high probability), and toxins were filtered out of the "non-pathogenic" dataset using PathoFact 1.0 predictions. The final "non-toxin" dataset consists of 213,129 non-redundant protein sequences (Figure 2).

The toxin-associated ML model is a RF with 5-mer features (default hyperparameter setting, i.e, n_estimators=100, max_depth=None, min_samples_split=2).

The toxin-associated protein prediction module generates a report containing the proteinID, protein domains, bitscore, toxin-associated ML probability, other identical proteins found in the sample, and optionally SignalP, plasmid marker, and virus marker information.

VF Prediction Updates

The VF prediction model was refined and updated with new HMM profiles. Training data was derived from SwissProt [31], selecting sequences annotated with the virulence keyword (KW-0843) and expanded using the Virulence Factor Database (VFDB; [43]) (Figure 2). After dereplication (with 100% identity and coverage), the original set of 32,511 sequences, using MMseqs2, comprised 30,695 non-redundant sequences, corresponding to the "VF dataset" (Figure 2). We searched the "VF dataset" against the CDD HMM profiles, selecting those with a bit score of 25 or higher as VF HMM profiles for PathoFact 2.0. The HMM profile dataset annotates the predicted VF domains rather than using them as input to the classification, as in the previous version.

To create the "non-VF" dataset for the ML VF model, we filtered out any potential VFs (with high and low probabilities), ARGs, and toxins based on PathoFact 1.0 predictions from the "non-pathogenic" dataset. This resulted in a dataset of 41,774 VF protein sequences (Figure 2).

The VF model uses XGBoost with protein sequence composition features (learning_rate': 0.1, 'n_estimators': 2000). The VF module generates a report containing the proteinID, protein domains, bitscore, virulence factor ML probability, other identical proteins found in the sample, and optionally SignalP, plasmid marker, and virus marker information.

ARG Prediction Updates

ARG prediction in PathoFact 2.0 integrates DeepARG (version 1.0.2; [44]), RGI (version 6; [6]), and AMRFinderPlus (version 3.12.8; [45]). DeepARG and RGI have received updates from their developers since the release of PathoFact 1.0, which have been incorporated into PathoFact 2.0. In addition, AMRFinderPlus has been newly integrated into PathoFact 2.0. Each tool has distinct strengths: DeepARG offers high precision and recall; RGI provides robust predictions based on an extensive database, utilising homology and single-nucleotide polymorphism (SNP) models; and AMRFinderPlus efficiently identifies resistance genes and mutations using NCBI resources.

The ARG prediction module (Figure 1) report includes protein IDs, ARG classes, prediction probabilities, database accession numbers, and optional data on signal peptides, plasmids, and virus markers. PathoFact 2.0 uses argNorm [46] to map detected genes to the ARO, thereby facilitating comparison of ARG annotation outputs by ensuring standardised and comparable results. Supplementary Figures S4 and S5 compare the performance of PathoFact 2.0 with that of its predecessor, PathoFact, in identifying ARGs.

Additional Functionalities

PathoFact 2.0 integrates SignalP (version 6; [24]) and antiSMASH (version 7.0; [27]), both of which are optional features that accommodate diverse research needs. SignalP is designed to predict the presence and location of signal peptides in protein sequences. It requires a separate license and must be requested by the user individually. AntiSMASH is designed to identify and annotate BGCs in bacterial and fungal genomes. Since AntiSMASH is a resource-intensive tool, we set it up as an optional module and provide the option to run it in chunks.

Evaluation of the performance of the PathoFact 2.0 pipeline

We evaluated the performance of PathoFact 2.0 and the new VF and toxin-associated modules using the test datasets described above. We did not include ARGs and BGCs in the validation step, as the respective modules are based on existing tools that have already demonstrated high accuracy [6,27,44,45]. Figure

3 provides a schematic overview of the benchmarking datasets used for the VF and toxin-associated modules.

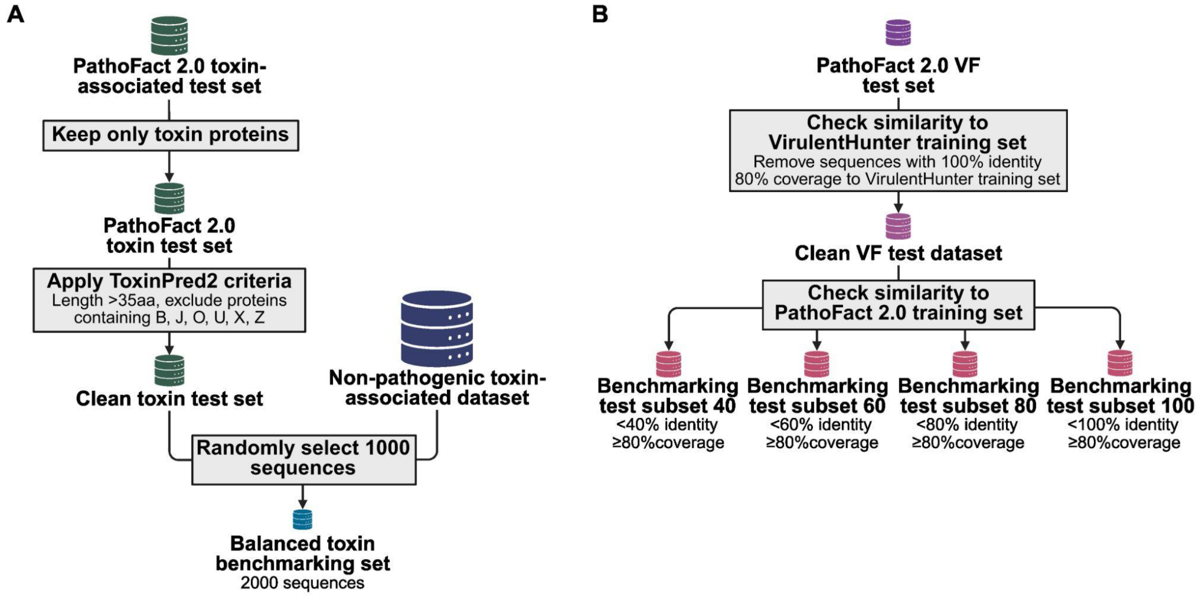

Figure 3. Schematic representation of the construction of the datasets used for benchmarking. A) Toxin benchmarking dataset. B) Virulence factor benchmarking dataset.

Virulence factors and toxin-associated protein prediction

The VF and toxin-associated modules (Figure 1) were evaluated across different ML models-predicted probability thresholds, corresponding to the model's confidence in the positive class (VF or toxin-associated). Analyses were performed on the full test dataset and on subsets of the test dataset. These subset datasets were created based on sequence similarity to the training dataset, with a range of 40% to 100% similarity and 80% coverage (Figure 2). This approach aimed to assess prediction accuracy on proteins in the testing dataset with low similarity to the training dataset, specifically including only sequences with less than 40-100% identity to any training sequence. The performance evaluation is based on the Matthew correlation coefficient (MCC) and the precision (to reduce the number of false positive VF and toxin-associated predictions), taking into account the dataset imbalance (a higher number of “non-toxin” and “non-VF” sequences compared to “toxin-associated” and “VF” sequences in the test subsets). The MCC is a more reliable statistical measure that yields a high score only when the prediction performs well across all four categories of the confusion matrix (true positives, false negatives, true negatives, and false positives), and it is proportional to both the number of positive and negative elements in the dataset [47]. We found that predicted probabilities of 0.6 for toxin-associated proteins and 0.9 for VFs provide a good balance between high MCC and precision across different test subsets (Figure 4 and Supplementary Tables S3 and S4).

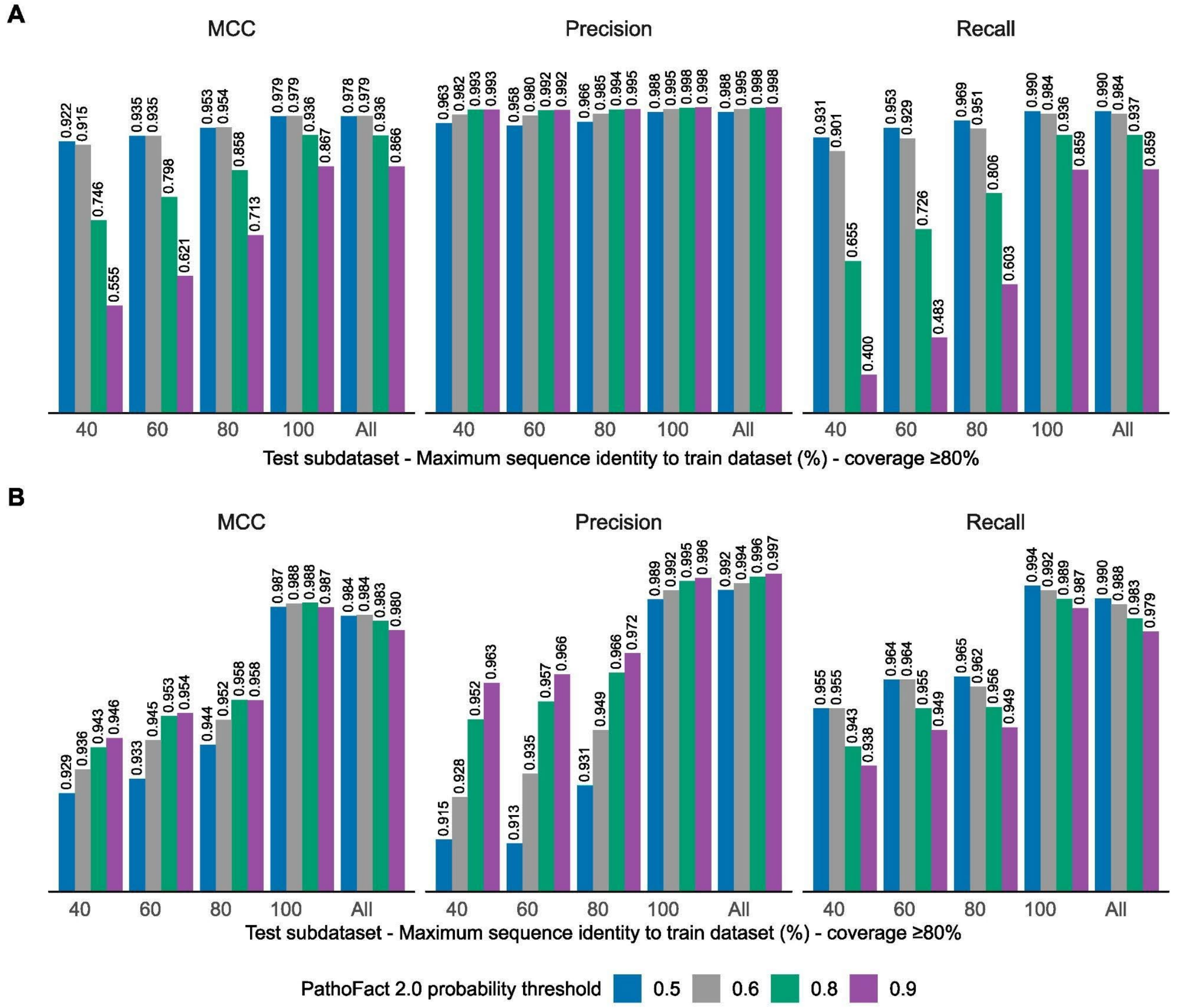

Figure 4. Performance evaluation of toxin-associated and virulence factor prediction modules across probability thresholds. A) Toxin-associated prediction module evaluation. **B)** Virulence factors prediction module evaluation. The modules were evaluated across a range of predicted class probabilities (0.5-0.9). The entire test dataset (All) and subsets of the test datasets were used for evaluation. These subset datasets were created based on sequence similarity to the training dataset, with similarity levels of 40%, 60%, 80%, and 100%, and an 80% coverage threshold. Only sequences with similarity below these percentages were included in the respective test subsets.

Benchmarking

The Pathofact 2.0 VF prediction module was compared to VirulentHunter [48], using the default parameters. VirulentHunter is a deep learning framework that simultaneously identifies and classifies VFs directly from protein sequences, which outperforms other virulence factor predictors (MP4 [49], VirulentPred 2.0 [50], and DeepVF [51]). A notable feature of VirulentHunter is that it provides VF category classification; however, it takes about 2 minutes to analyse 500 protein sequences (using 1 GPU), which is a drawback for metagenomic sample analysis, where thousands to millions of proteins are predicted from a single sample. PathoFact 2.0 requires only 4 seconds (using 1 CPU, with the option to utilise more CPUs) to analyse 500 protein sequences (Table 1).

Table 1. Runtime comparison of PathoFact 2.0 and VirulentHunter

Number of protein sequences	VirulentHunter	PathoFact 2.0				
	1 GPU	1 CPU	2 CPU	4 CPU	6 CPU	8 CPU
500	2 min 21 s	3.7 s	2.6 s	2.0 s	1.9 s	1.8 s
5500	25 min 42 s	29.9 s	17.4 s	10.2 s	8.1 s	7.4 s
10000	54 min 11 s	55.4 s	29.3 s	16.7 s	13.1 s	11.5 s
30000	2 h 45 min 50 s	2 min 45 s	1 min 32.9 s	52.1 s	40.3 s	36.0 s

Since VirulentHunter and Pathofact 2.0 employ a similar method to generate the “VF dataset” for model training, we removed sequences from the Pathofact 2.0 test dataset that have 100% identity (≥80% coverage) to the VirulentHunter training dataset, resulting in a “clean VF test dataset” (Figure 3). This ensures that neither model used the test sequences for training. We applied the same test-subset approach described earlier: the subset datasets were created based on sequence similarity to the Pathofact 2.0 training dataset, with similarity ranging from 40% to 100% and 80% coverage of the “clean VF test dataset” (Figure 3). By stratifying test sets by decreasing sequence similarity to the training data, we explicitly evaluated model performance across progressively more divergent proteins. This study demonstrates that PathoFact 2.0 maintains consistent performance even when test sequences share 40% similarity or less with the training set (Figure 5B).

The Pathofact 2.0 toxin-associated module was compared with ToxinPred2 [42] using the default parameters, i.e., Hybrid (RF+BLAST+MERCI) with a threshold of 0.6. The ToxinPred2 website restricts predictions to a certain number of proteins (around 2000). Since ToxinPred2 is designed to predict protein toxicity, we selected sequences from the Pathofact 2.0 “toxin-associated” test dataset that are directly linked to toxins and removed the remaining “toxin-associated” proteins (Figure 3). In short, using the header information from the Pathofact 2.0 “toxin-associated” test dataset, we kept only toxin sequences from the toxin-antitoxin sequences from the TADB, the effector factor sequence from the SecReT6 database, bacterial protein toxins from T3DB, bacterial exotoxins from DBETH, and sequences from Swissprot (KW-0800, toxin), as previously described. Additionally, we kept sequences longer than 35 amino acids and excluded protein sequences containing the non-standard amino acids ‘BJOUXZ’ as the ToxinPred2 dataset was created using these criteria [52] (Figure 3). From these, we randomly selected 1000 sequences. Then, we randomly selected 1000 sequences from the Pathofact 2.0 “non-toxin” test dataset (Figure 3). This resulted in a total of 2000 sequences for benchmarking PathoFact 2.0 against the ToxinPred2 webserver. Due to the limited number of sequences, we did not use the test-subset approach to evaluate ToxinPred2 and Pathofact 2.0 toxin-associated modules on this 2000-sequence test dataset (Figure 3). We also use the same test dataset to benchmark CSM-toxin v1.0.1, a deep learning model for toxin prediction [53].

As shown in Figure 5 (and in the Supplementary Tables S5 and S6), Pathofact 2.0 VF and toxin-associated modules exhibited higher MCC values across different test subsets compared to VirulentHunter, ToxinPred2 and CSM-toxin.

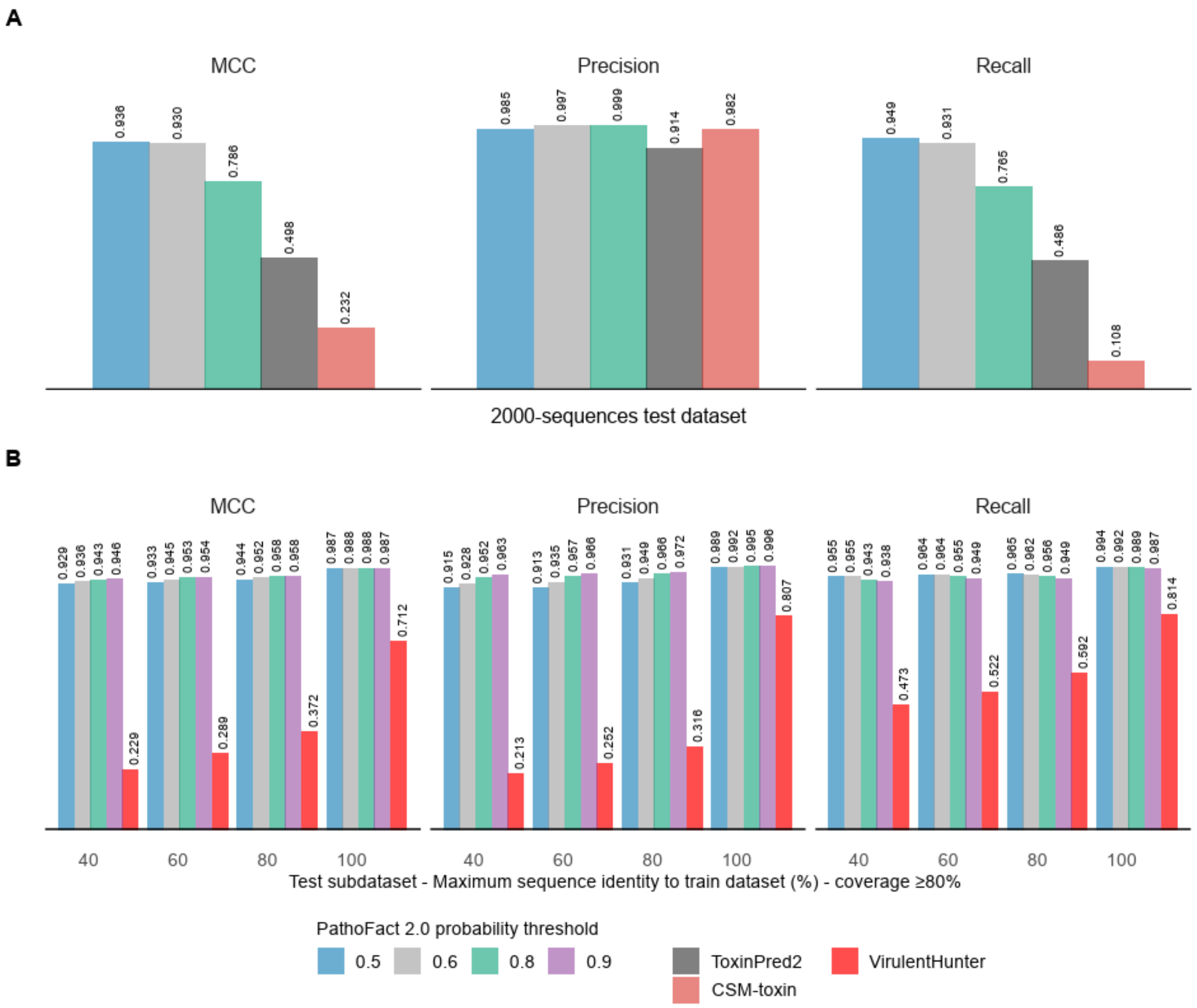

Figure 5. Benchmarking of toxin and virulence factor prediction performance. A) Toxin-associated module benchmarking. The Pathofact 2.0 toxin-associated module was compared with ToxinPred2 (Hybrid: RF+BLAST+MERCI, threshold = 0.6) using its web-based version and CSM-toxin v 1.0.1. A balanced toxin test dataset (1,000 toxin and 1,000 non-toxin sequences) was built from the Pathofact 2.0 toxin-associated test dataset, selecting only sequences from curated toxin sources (TADB, SecReT6, T3DB, DBETH, SwissProt), applying ToxinPred2's filtering criteria. Several predicted probability cutoffs, 0.5, 0.6 and 0.8, of the Pathofact 2.0 toxin-associated module were evaluated. MCC, precision, and recall are shown. **B)** Virulence factors module benchmarking. The Pathofact 2.0 VF module was compared to VirulentHunter. Sequences identical (100% identity, ≥80% coverage) to VirulentHunter's training data were removed from the Pathofact 2.0 test dataset. Then, test subset datasets were created based on 40–100% similarity to the Pathofact 2.0 training set. These subset datasets were created based on sequence similarity to the training dataset, with similarity levels of 40%, 60%, 80%, and 100%, and an 80% coverage threshold. Only sequences with similarity below these percentages were included in the respective test subsets. Several predicted probability cut-offs (0.5, 0.6, 0.8, and 0.9) of the Pathofact 2.0 VF module were evaluated.MCC, precision, and recall are presented for each test subset.

Virulence factors and toxin-associated protein prediction with contig sequences as input

To evaluate PathoFact 2.0 at the contig level, we analysed publicly available complete genomes from pathogenic and non-pathogenic bacteria, including various *Escherichia coli* strains. Figure 6 shows distinct differences in virulence and toxin-related profiles between pathogenic and non-pathogenic *E. coli* strains, especially regarding virulence- and toxin-associated proteins encoded on MGEs, such as

plasmids and prophages. Nonetheless, analysis of individual VF predictions reveals considerable overlap in the number of VF genes detected across both pathogenic and non-pathogenic strains. This highlights that the presence of a VF gene is not a reliable marker of pathogenicity and emphasises the importance of considering genomic and functional context when assessing virulence potential.

It is well known that VFs of pathogenic *E. coli* are often encoded on genetic elements, such as plasmids, bacteriophages, transposons, and pathogenicity islands, which can be mobilised into different strains to create novel combinations of virulence factors [54,55]. The same pattern is observed in pathogenic strains of several genera compared to non-pathogenic strains (Supplementary Figure S1), particularly for *Klebsiella pneumoniae* and *Salmonella enterica*. These findings highlight the importance of examining virulence from a systems perspective rather than focusing solely on the presence or absence of individual factors. A comprehensive assessment should consider not only whether a virulence- or toxin-associated protein is encoded within an MGE but also its functional context, such as whether it is secreted or part of a BGC.

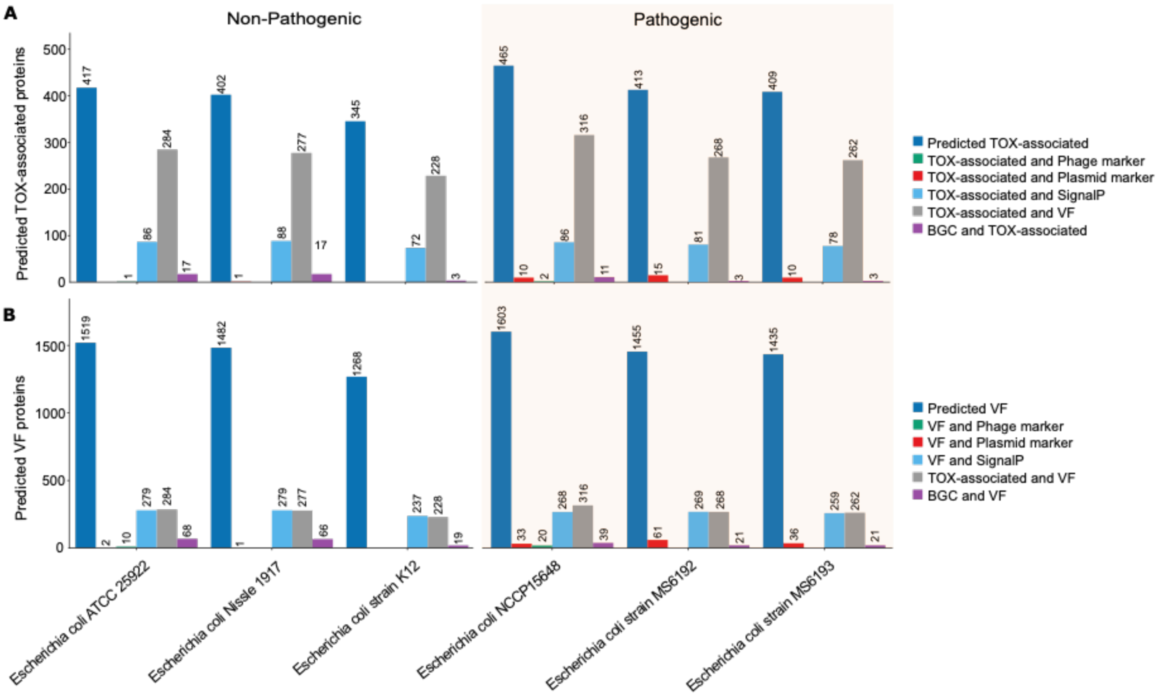

Figure 6. Comparative analysis of toxin-associated and virulence factor profiles in non-pathogenic and pathogenic *Escherichia coli* strains. Bar charts represent the distribution of predicted toxin-associated (A) and virulence-associated (B) proteins across non-pathogenic (left panel) and pathogenic (right panel) *E. coli* strains. The categories include total predicted virulence factors/toxin-associated proteins (dark blue), those associated with plasmid markers (red), those associated with phage markers (green), and proteins predicted by SignalP to be secreted (light blue). Additional categories include toxin-associated virulence factors (TOX-associated and VF, grey) and biosynthetic gene clusters overlapping (purple). Numerical values above each bar indicate the total count of proteins identified in each category for the corresponding strain.

Benchmarking

The PathoFact 2.0 VF module was compared to PathoFact 1.0 and metaVF [23], an alignment-based toolkit (based on BLAST) that identifies species-level VFs associated with pathobionts. To our knowledge, no other method is available to predict VF from contig sequences and identify plasmid-encoded or

prophage-associated VF. However, we included VirulentHunter in the comparison. Because VirulentHunter does not accept contig sequences as input, protein-coding genes were first predicted from contigs using Pyrodigal-gv and the resulting protein sequences were subsequently analysed. PathoFact 2.0 consistently detected a greater number of VFs than both PathoFact 1.0 and MetaVF (Figure 7, Supplementary Figures S2–S3). Notably, MetaVF failed to identify any VFs in five of the ten pathogenic reference strains tested, highlighting its limited ability to detect VFs and demonstrating the advantages of machine-learning-based models over homology-based approaches. While VirulentHunter produced substantially more hits than metaVF, yielding predictions comparable in number to PathoFact 2.0, PathoFact 2.0 generally identified more VFs overall (Figure 7). Exceptions were observed for non-pathogenic strains *Bifidobacterium animalis*, *Bifidobacterium bifidum*, *Heyndrickxia coagulans*, and for the pathogenic *Ralstonia mannitolilytica strain Guangzhou-RMAB10*, where VirulentHunter predicted slightly more VFs.

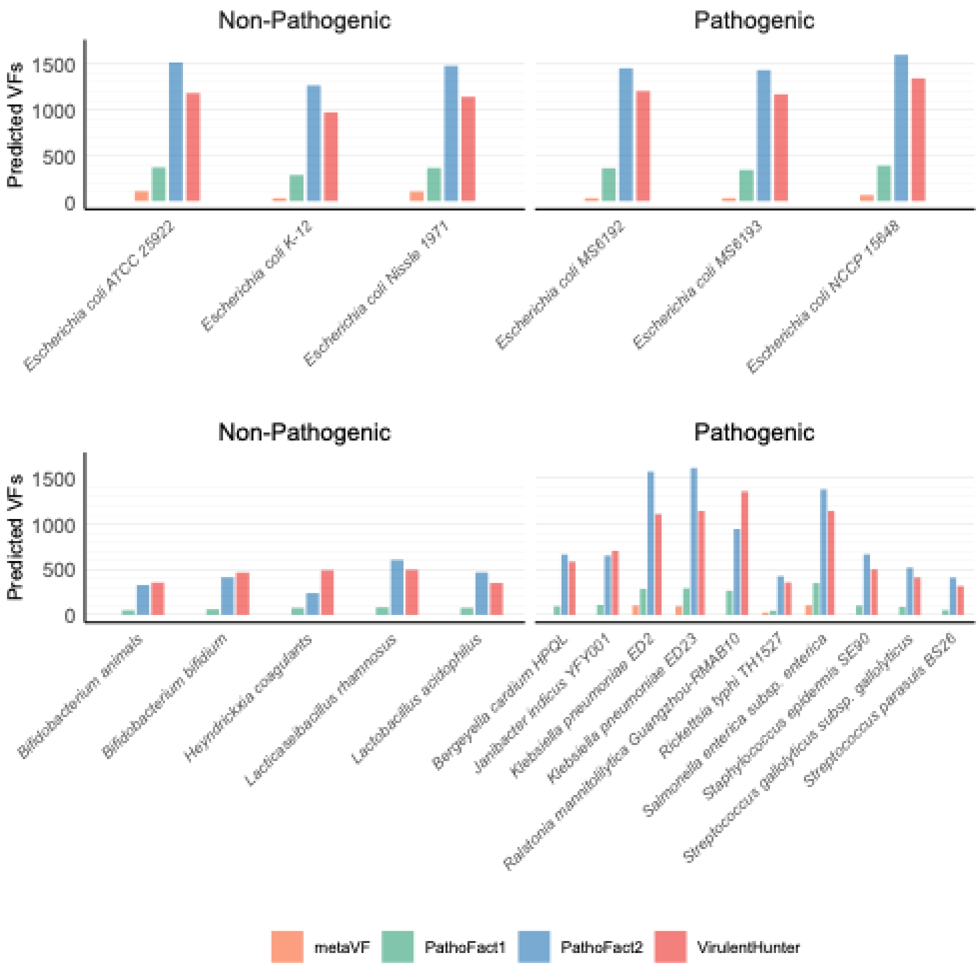

Figure 7. Performance comparison of virulence factor prediction tools in pathogenic and non-pathogenic strains. Comparative performance of PathoFact 2.0 (blue), PathoFact (green), metaVF (orange), and VirulentHunter (red) in predicting virulence factors (VFs). Results are shown separately for non-pathogenic (left) and pathogenic (right) strains.

In addition, the PathoFact 2.0 toxin-associated module was compared to PathoFact 1.0 (Figure 8, Supplementary Figures S4–S5) as well as CSM-Toxin and ToxinPred2 (Figure 8). As CSM-Toxin and ToxinPred2 do not accept contig sequences as input, protein sequences were first predicted from contigs using Pyrodigal-gv. PathoFact 2.0 predicted more toxin-associated proteins than PathoFact 1.0, demonstrating improved detection capacity. Compared to external tools, CSM-Toxin identified substantially fewer toxins across the tested reference strains. In contrast, ToxinPred2 predicted a

comparable number of toxins overall. However, for non-pathogenic strains *Bifidobacterium bifidum*, *Heyndrickxia coagulans*, *Lactobacillus acidophilus*, and for pathogenic strains *Streptococcus gallolyticus subsp. gallolyticus* and Streptococcus parasuis B26, ToxinPred2 identified more predicted toxins than PathoFact 2.0.

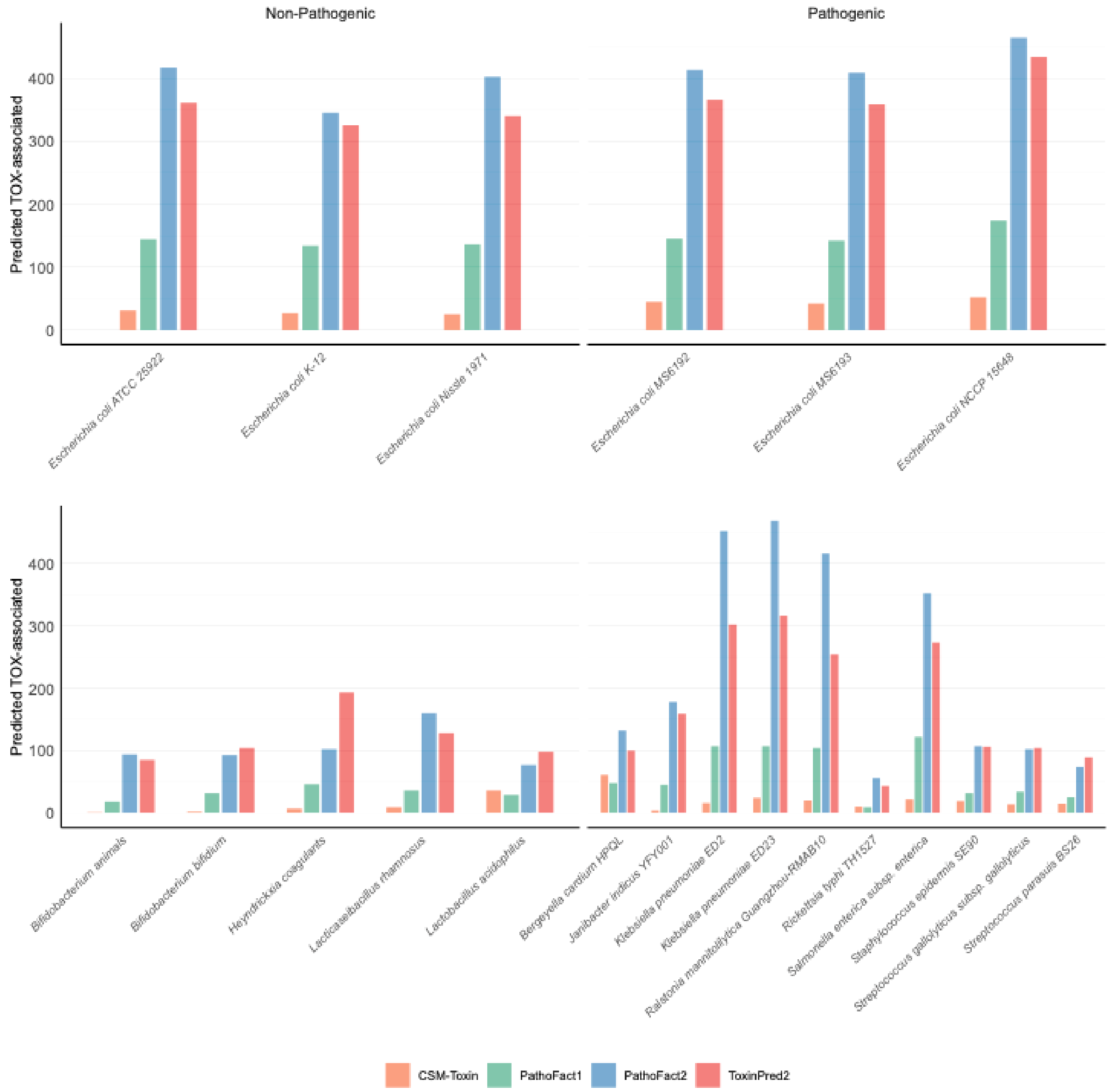

Figure 8. Performance comparison of toxin prediction tools in pathogenic and non-pathogenic strains. Comparative performance of PathoFact 2.0 (blue), PathoFact (green), CSM-Toxin (orange), and ToxinPred2 (red) in predicting virulence factors (VFs). Results are shown separately for non-pathogenic (left) and pathogenic (right) strains.

PathoFact 2.0 Output Structure

PathoFact 2.0 creates a structured output directory that summarises predictions from all analysis modules, including VFs, toxin-associated proteins, ARGs, MGEs and BGCs. Each module generates dedicated result files corresponding to the underlying prediction tools (Supplementary File S1).

The primary summary file, combined_report.tsv, provides an integrated overview of high-confidence predictions across all modules in a tabular format. This table includes key information such as protein identifiers, bit scores (from HMM profiles), machine-learning prediction scores, and outputs from DeepARG, RGI, SignalP, GenoMad, and antiSMASH, thereby supporting downstream interpretation and candidate prioritisation.

Proteins with prediction probabilities below user-defined thresholds but containing conserved domains identified by toxin-associated or VF HMM profiles are reported in ambiguous_TOX_hits_rep_prot.tsv and ambiguous_VF_hits_rep_prot.tsv. These lower-confidence candidates may warrant further investigation in comparative or experimental analyses.

High-confidence predictions are reported in amr_hits_rep_prot.tsv, TOX_hits_rep_prot.tsv, and VF_hits_rep_prot.tsv, which summarise features exceeding user-defined probability thresholds and include protein identifiers, bit scores, machine-learning predictions, signal peptide predictions, and genomic context information, such as association with prophages or plasmids identified by GenoMad.

In addition, PathoFact 2.0 generates a dedicated Group_of_sequence directory containing FASTA files of representative protein sequences grouped by functional category (VFs, toxin-associated proteins, antimicrobial resistance genes, and combined hits), together with conserved domain (CDD) annotation tables for predicted VFs and toxin-associated proteins. These files are designed to facilitate downstream analyses, including comparative genomics and functional characterisation.

Limitations of PathoFact 2.0

It is well established that non-pathogenic bacterial strains can also carry genes annotated as VFs or toxins [7]. Consequently, PathoFact 2.0 is most effective as an initial screening tool to identify potential candidates, which can then be examined in comparative studies to distinguish confirmed pathogenic cases from controls. The pipeline provides a probability score indicating whether a protein is likely to be VF- or toxin-associated; however, establishing a definitive link between predicted candidates and infectious disease requires experimental validation.

PathoFact 2.0 does not directly classify specific VF or toxin types (e.g., adhesins or genotoxins). Instead, it reports detailed annotations of conserved protein domains from CDD [18], allowing users to infer functional roles when available. This design emphasises contextual interpretation rather than categorical assignment.

The inclusion of housekeeping genes from non-pathogenic microorganisms reflects a deliberate methodological choice rather than a limitation. Multiple well-characterised housekeeping proteins have been shown to exhibit virulence-associated “moonlighting” functions in pathogenic bacteria, including roles in adhesion, immune modulation, and tissue invasion [56,57]. Notable examples include glyceraldehyde-3-phosphate dehydrogenase (VF0015 in VFDB) [58], enolase [59,60], elongation factor Tu (VF0460 in VFDB) [61,62], GroEL [60,63,64], and DnaK [60,65]. This approach maintains a biologically realistic negative dataset while reducing the risk of misclassifications within the intended scope of PathoFact 2.0.

PathoFact 2.0 is designed for metagenomic samples; most prediction modules and phenotypes are bacterial-centric. Virulence, toxin-associated, and antimicrobial resistance predictions are particularly interpreted in the context of human pathogens.

Conclusions

ARGs, VFs, and toxins represent major threats to global health. Therefore, accurate detection of these elements is crucial for assessing the presence and potential risks of pathogenic microorganisms in microbiomes and for identifying reservoirs of pathogenicity. Our improved pipeline, PathoFact 2.0, offers significant improvements over PathoFact (its predecessor), ToxinPred2, CSM-toxin, VirulentHunter, and metaVF. SignalP has been upgraded and made optional to further optimise performance, providing users with flexibility based on their requirements. Additionally, antiSMASH 7.0 facilitates the prediction of BGCs, recognising emerging evidence that some BGC-encoded factors might increase virulence. Furthermore, we have integrated geNomad, a cutting-edge tool for identifying MGEs, including plasmids and phages linked to ARGs, VFs, toxins and toxin-associated proteins across various bacterial species. The PathoFact 2.0 update improves the accuracy and sensitivity of analyses while enhancing computational efficiency.

PathoFact 2.0 represents a major advance in metagenomic analysis by integrating the detection of ARGs, VFs, toxins and toxin-associated proteins, signal peptides, MGEs, and BGCs within a single, streamlined pipeline. Unlike existing tools that focus on individual aspects of pathogenicity, PathoFact 2.0 provides a comprehensive, multi-layered view that captures both gene presence and genomic context, improving interpretability and enabling a holistic assessment of microbial pathogenic potential.

Methods

Databases used for the PathoFact 2.0 Dataset Construction

- SwissProt [31] is the expertly curated part of UniProtKB [66]. It offers high-quality protein sequences with detailed functional annotations, including keywords for pathogenesis, virulence, toxins, and antibiotic resistance.
- VFDB [43], the Virulence Factor Database, is a comprehensive reference for curating information on virulence factors of bacterial pathogens.
- T3DB [12], the toxin and toxin-target database, is a resource cataloguing thousands of toxins and their protein targets, with detailed mechanisms, structures, and toxicity data, including bacterial protein toxins.
- DBETH [13], the database for bacterial exotoxins, is a specialised database of bacterial exotoxins pathogenic to humans, classified into 24 mechanistic and activity types from 26 bacterial genera.
- TADB [36], the toxin-antitoxin database, is a repository of bacterial toxin-antitoxin loci across types I-VIII, including experimentally validated pairs, predicted loci, and associations with mobile genetic elements.
- SecReT6 [37] is a database containing known and predicted type VI secretion systems, including effectors, immunity proteins, regulators, and accessory proteins from bacterial genomes.
- PAT [38] is the prokaryotic antimicrobial toxin database and contains a collection of antimicrobial toxins, including bacteriocins and effectors from secretion systems.

Clustering Parameters

MMseqs2 [32] was used for dereplication; sequences were clustered at 100% identity and 100% coverage using the parameters -c 1.0 and --min-seq-id 1.0. It removes exact duplicates and retains representative sequences to generate non-redundant datasets. To create the test subsets, a coverage of 80% (-c 0.8) and identities ranging from 40 - 100% (--min-seq-id 0.4, --min-seq-id 0.6, --min-seq-id 0.8, --min-seq-id 1.0) were used, and then sequences with similarity higher than min-seq-id were removed for each test subset. In all cases, the cluster mode and coverage mode used were 0 (--cov-mode 0 --cluster-mode 0). When --cov-mode 0 is specified in combination with −c values ranging from 0.0 to 1.0, sequences are assigned to the same cluster only if the alignment spans at least a fraction *c* of the length of the longer sequence. According to the developers of MMSeq2, this coverage criterion is particularly suitable for clustering full-length protein sequences [32].

Protein Composition Features

Protein sequence composition features were extracted to represent each protein as fixed-length vectors derived from its primary amino acid sequence [35]. These included amino acid composition (AAC), dipeptide composition (DPC), composition (CTDC), transition (CTDT) and distribution (CTDD). AAC captures the relative frequency of each of the 20 amino acids in a sequence, whereas DPC captures the relative frequency of all adjacent amino-acid pairs (400 possible dipeptides). CTDC represents the percentage of amino acids belonging to each of three predefined groups (polar, neutral, hydrophobic) in the entire protein sequence. CTDT represents the percentage frequency with which a residue of one group is followed by a residue of a different group along the sequence. CTDD represents the distribution of each amino acid group, measuring the spatial position, where the first, 25%, 50%, 75% and 100% of the residues of a specific class are located.

Performance and Evaluation Metrics

To assess the models' performance, we used a confusion matrix comprising true positives (TP), true negatives (TN), false positives (FP), and false negatives (FN), computed on the test datasets described above. TP corresponds to truly positive instances correctly predicted as positive by the model, whereas TN corresponds to truly negative instances correctly predicted as negative. FP are truly negative instances incorrectly predicted as positive, and FN are truly positive instances incorrectly predicted as negative. From these values, we calculated the following metrics:

$$Accuracy = (TP + TN)/(TP + TN + FP + FN)$$

$$Precision = TP/(TP + FP)$$

$$Recall = TP/(TP + FN)$$

$$MCC = (TP \times TN - FP \times FN)/\sqrt{(TP + FP)(TP + FN)(TN + FP)(TN + FN)}$$

HMM Profiles

Profile hidden Markov models are probabilistic models built from a multiple sequence alignment that encode, for each alignment position, the position-specific probabilities of residues and insertions/deletions, turning the alignment into a position-specific scoring system for detecting homologous sequences [67]. The FASTA files of conserved-domain multiple sequence alignments for each CDD [26] family (https://ftp.ncbi.nih.gov/pub/mmdb/cdd/fasta.tar.gz) were downloaded. Pyhmmer v0.10.14 [68] was used to obtain HMM profiles for each CDD family and to perform protein sequence searches against the CDD HMM family profiles.

Benchmarking Datasets

This study utilised publicly available datasets containing complete genomes from pathogenic and non-pathogenic bacteria, including various *Escherichia coli* strains, from NCBI. The accession numbers of the bacteria used are indicated in Supplementary Table S2.

Availability of Supporting Source Code and Requirements

Project name: Pathofact 2.0
Project homepage: https://gitlab.com/uniluxembourg/lcsb/systems-ecology/pathofact2
Operating system(s): Linux
Programming language: Python, R, bash
Other requirements: Snakemake, Mamba, conda.
License: GNU General License v3.0 or later
Biotools ID: pathofact2
RRID: SCR_027650
workflowhubDOI: 10.48546/workflowhub.workflow.2087.1

Additional Files

Supplementary Table S1. List of microorganisms non-pathogenic to humans and their total protein count obtained from the NCBI Database.

Supplementary Table S2. Bacterial strains used in this study, including their classification as pathogenic or non-pathogenic, species/strain information, genome assembly or reference version, and corresponding accession numbers.

Supplementary Table S3. Evaluation of the PathoFact 2.0 toxin-associated protein prediction module. The table presents performance across test subsets defined by sequence similarity to the training set. Metrics reported include class distributions (Negative, non-toxin; Positive, toxin-associated), confusion matrix counts (true negatives, false positives, true positives, false negatives), and performance measures (accuracy, precision, recall, F1 score, Matthews correlation coefficient).

Supplementary Table S4. Evaluation of the PathoFact 2.0 virulence factor prediction module. The table summarises performance across test subsets defined by sequence similarity to the training set. Reported metrics include class distributions (Negative, non-VF; Positive, VF), confusion matrix counts, and performance measures (accuracy, precision, recall, F1 score, Matthews correlation coefficient).

Supplementary Table S5. Comparison of virulence factor prediction performance for PathoFact 2.0 at varying probability cutoffs and for VirulenHunter, evaluated across test subsets stratified by sequence similarity to the training set. The table reports class distributions, confusion matrix counts, and performance metrics (accuracy, precision, recall, F1 score, Matthews correlation coefficient).

Supplementary Table S6. Comparison of toxin protein prediction performance for PathoFact 2.0 at different prediction probability cutoffs and for ToxinPred2. The table reports the number of proteins classified as Negative (non-toxin) and Positive (toxin), confusion matrix counts, and associated performance metrics (accuracy, precision, recall, F1 score, and Matthews correlation coefficient).

Supplementary Figure S1. Comparative analysis of toxin-associated and virulence factor profiles in non-pathogenic and pathogenic bacterial strains. Bar charts represent the distribution of predicted toxin-associated (A) and virulence-associated (B) proteins across non-pathogenic (left panel) and pathogenic (right panel) *E. coli* strains. The categories include total predicted virulence factors/toxin-associated proteins (dark blue), associated with plasmid markers (red), phage markers (Green), and proteins predicted by SignalP to be secreted (light blue). Additional categories include toxin-associated virulence factors (TOX-related ∩ VF, grey) and biosynthetic gene clusters overlapping (purple). Numerical values above each bar indicate the total count of proteins identified in each category for the corresponding strain.

Supplementary Figure S2. Comparative performance of Pathofact 2.0 (blue) versus PathoFact1 (green) and metaVF (red) in predicting virulence factors (VFs) in non-pathogenic (left) and pathogenic (right) *Escherichia coli* strains. The top panel shows the total number of predicted VFs. The second panel depicts the subset predicted to be secreted. The third panel shows VFs predicted to be plasmid-encoded, while the fourth panel presents those predicted to be prophage-associated.

Supplementary Figure S3. Comparative performance of Pathofact 2.0 (blue) versus PathoFact (green) and metaVF (red) in predicting virulence factors (VFs) in non-pathogenic (left) and pathogenic (right) bacterial strains. The top panel shows the total number of predicted VFs. The second panel depicts the subset predicted to be secreted. The third panel shows VFs predicted to be plasmid-encoded, while the fourth panel presents those predicted to be prophage-associated.

Supplementary Figure S4. Performance comparison of PathoFact 2.0 versus PathoFact for ARG and toxin prediction in Escherichia coli. A) Comparative performance of PathoFact 2.0 (blue) versus PathoFact (green) in predicting antimicrobial resistance genes (ARGs) in non-pathogenic (left) and pathogenic (right) *E. coli* strains. **B)** Comparative performance of PathoFact 2.0 (blue) versus PathoFact (green) in predicting toxin-associated proteins in non-pathogenic (left) and pathogenic (right) *E. coli* strains. The top panel shows the total number of predicted toxin-associated proteins, while the bottom panel shows those that contain signal peptides, as identified by SignalP.

Supplementary Figure S5. Performance comparison of PathoFact 2.0 versus PathoFact for ARG and toxin prediction in bacterial strains. A) Comparative performance of PathoFact 2.0 (blue) versus PathoFact (green) in predicting antimicrobial resistance genes (ARGs) in non-pathogenic (left) and pathogenic (right) bacterial strains. **B)** Comparative performance of PathoFact 2.0 (blue) versus PathoFact (green) in predicting toxin-associated proteins in non-pathogenic (left) and pathogenic (right) bacterial strains. The top panel shows the total number of predicted toxin-associated proteins, while the bottom panel shows those that contain signal peptides, as identified by SignalP.

Abbreviations

AAC, amino acid composition; ARO, antibiotic resistance ontology; ARGs, antimicrobial resistance genes; BGCs, biosynthetic gene clusters; CDD, conserved domains database; CTDC, (Composition,

Transition, Distribution)-composition; CTDD, (Composition, Transition, Distribution)-distribution; CTDT, (Composition, Transition, Distribution)-transition; DBETH, database for bacterial exotoxins; DPC, dipeptide composition; HMMs, hidden Markov models; MCC, Matthews correlation coefficient; MGEs, mobile genetic elements; ML, machine learning; ORF, open reading frame; PAT, prokaryotic antimicrobial toxins database; RF, random forest; SM, specialised metabolites; SMOTE, synthetic minority oversampling technique; SNP, single nucleotide polymorphisms; T3DB, toxin exposome database; VFDB, virulence factor database; VFs, virulence factors.

Acknowledgements

The experiments presented in this paper were carried out using the HPC facilities of the University of Luxembourg (Varrette et al., 2022). The manuscript also passed the Luxembourg Centre for Systems Biomedicine internal pre-publication check designed to ensure FAIRness and reproducibility.

Author Contributions

P.W. initiated the study, which involved the overall design and objective, and was led by L.F.D. and J.O.S. in the development of PathoFact 2.0. O.H. contributed to early brainstorming discussions on workflow design and database strategy. P.M. and C.C.L. contributed to the overall discussions. L.F.D. and J.O.S. wrote the draft manuscript. All authors read and commented on the manuscript.

Funding

This work has been supported by the Pélican grant from the Mie and Pierre Hippert-Faber Pélican Foundation under the aegis of Fondation de Luxembourg to JOS, as well as by the Luxembourg National Research Fund (FNR CORE/23/BM/15886415) and the European Research Council (ERC-CoG 863664) to PW. The Luxembourg Government further supported the work through the CoVaLux program.
This research was funded in whole, or in part, by the Luxembourg National Research Fund (FNR), grant reference (FNR CORE/23/BM/15886415). For the purpose of open access, and in fulfilment of the obligations arising from the grant agreement, the author has applied a Creative Commons Attribution 4.0 International (CC BY 4.0) license to any Author Accepted Manuscript version arising from this submission.

Data Availability

Pathofact 2.0 is accessible at https://gitlab.com/uniluxembourg/lcsb/systems-ecology/pathofact2. Additionally, the core databases required to run the pipeline can be found at https://zenodo.org/records/14192463. The ML datasets used for training, validation, and benchmarking of PathoFact 2.0 can be found in https://zenodo.org/records/17647372.

Competing Interests

None declared.

References

1. Hou K, Wu Z-X, Chen X-Y, Wang J-Q, Zhang D, Xiao C, et al.. Microbiota in health and diseases.

Signal Transduct Target Ther. Springer Science and Business Media LLC; 7:1352022;

2. Inda-Díaz JS, Lund D, Parras-Moltó M, Johnning A, Bengtsson-Palme J, Kristiansson E. Latent antibiotic resistance genes are abundant, diverse, and mobile in human, animal, and environmental microbiomes. *Microbiome*. Springer Science and Business Media LLC; 11:442023;

3. Beceiro A, Tomás M, Bou G. Antimicrobial resistance and virulence: a successful or deleterious association in the bacterial world? *Clin Microbiol Rev*. American Society for Microbiology; 26:185–2302013;

4. Zhu C, Wu L, Ning D, Tian R, Gao S, Zhang B, et al.. Global diversity and distribution of antibiotic resistance genes in human wastewater treatment systems. *Nat Commun*. Springer Science and Business Media LLC; 16:40062025;

5. Jian Z, Zeng L, Xu T, Sun S, Yan S, Yang L, et al.. Antibiotic resistance genes in bacteria: Occurrence, spread, and control. *J Basic Microbiol*. Wiley; 61:1049–702021;

6. Alcock BP, Huynh W, Chalil R, Smith KW, Raphenya AR, Wlodarski MA, et al.. CARD 2023: expanded curation, support for machine learning, and resistome prediction at the Comprehensive Antibiotic Resistance Database. *Nucleic Acids Res*. Oxford University Press (OUP); 51:D690–92023;

7. Niu C, Yu D, Wang Y, Ren H, Jin Y, Zhou W, et al.. Common and pathogen-specific virulence factors are different in function and structure. *Virulence*. Informa UK Limited; 4:473–822013;

8. Sharma AK, Dhasmana N, Dubey N, Kumar N, Gangwal A, Gupta M, et al.. Bacterial virulence factors: Secreted for survival. *Indian J Microbiol*. 57:1–102017;

9. Blair JMA, Webber MA, Baylay AJ, Ogbolu DO, Piddock LJV. Molecular mechanisms of antibiotic resistance. *Nat Rev Microbiol*. Springer Science and Business Media LLC; 13:42–512015;

10. Rodríguez-Beltrán J, DelaFuente J, León-Sampedro R, MacLean RC, San Millán Á. Beyond horizontal gene transfer: the role of plasmids in bacterial evolution. *Nat Rev Microbiol*. Springer Science and Business Media LLC; 19:347–592021;

11. Galanos C, Freudenberg MA. Bacterial endotoxins: biological properties and mechanisms of action. *Mediators Inflamm*. Wiley; 2:S11–61993;

12. Wishart D, Arndt D, Pon A, Sajed T, Guo AC, Djoumbou Y, et al.. T3DB: the toxic exposome database. *Nucleic Acids Res*. Oxford University Press (OUP); 43:D928–342015;

13. Chakraborty A, Ghosh S, Chowdhary G, Maulik U, Chakrabarti S. DBETH: A database of Bacterial ExoToxins for human. *Nucleic Acids Res*. Oxford University Press (OUP); 40:D615–202012;

14. Green ER, Mecsas J. Bacterial secretion systems: An overview. *Microbiol Spectr*. 2016; doi: 10.1128/microbiolspec.VMBF-0012-2015.

15. Kaushik S, He H, Dalbey RE. Bacterial signal peptides- navigating the journey of proteins. *Front Physiol*. Frontiers Media SA; 13:9331532022;

16. Lybbert AC, Williams JL, Raghuvanshi R, Jones AD, Quinn RA. Mining public mass spectrometry data to characterize the diversity and ubiquity of P. aeruginosa specialized metabolites. *Metabolites*. MDPI AG; 10:4452020;

17. Elshafie HS, Camele I. An overview of metabolic activity, beneficial and pathogenic aspects of

Burkholderia spp. *Metabolites*. MDPI AG; 11:3212021;

18. Lau GW, Hassett DJ, Ran H, Kong F. The role of pyocyanin in Pseudomonas aeruginosa infection. *Trends Mol Med*. Elsevier BV; 10:599–6062004;

19. Ambassadors G, Patrons: World leaders commit to decisive action on antimicrobial resistance. UN Environment. https://www.unep.org/news-and-stories/press-release/world-leaders-commit-decisive-action-antimicrobial-resistance (2024). Accessed 2025 Oct 7.

20. Environment UN: Antimicrobial Resistance (AMR). UNEP - UN Environment Programme. https://www.unep.org/topics/chemicals-and-pollution-action/pollution-and-health/antimicrobial-resistance-amr (2024). Accessed 2025 Oct 7.

21. Bansal MA, Sharma DR, Kathuria DM. A systematic review on data scarcity problem in deep learning: Solution and applications. *ACM Comput Surv*. Association for Computing Machinery (ACM); 54:1–292022;

22. de Nies L, Lopes S, Busi SB, Galata V, Heintz-Buschart A, Laczny CC, et al.. PathoFact: a pipeline for the prediction of virulence factors and antimicrobial resistance genes in metagenomic data. *Microbiome*. Springer Science and Business Media LLC; 9:492021;

23. Dong W, Fan X, Guo Y, Wang S, Jia S, Lv N, et al.. An expanded database and analytical toolkit for identifying bacterial virulence factors and their associations with chronic diseases. *Nat Commun*. Springer Science and Business Media LLC; 15:80842024;

24. Ji B, Pi W, Liu W, Liu Y, Cui Y, Zhang X, et al.. HyperVR: a hybrid deep ensemble learning approach for simultaneously predicting virulence factors and antibiotic resistance genes. *NAR Genom Bioinform*. 5:lqad0122023;

25. Rathore AS, Choudhury S, Arora A, Tijare P, Raghava GPS. ToxinPred 3.0: An improved method for predicting the toxicity of peptides. *Comput Biol Med*. Elsevier BV; 179:1089262024;

26. Wang J, Chitsaz F, Derbyshire MK, Gonzales NR, Gwadz M, Lu S, et al.. The conserved domain database in 2023. *Nucleic Acids Res*. Oxford University Press (OUP); 51:D384–82023;

27. Blin K, Shaw S, Augustijn HE, Reitz ZL, Biermann F, Alanjary M, et al.. antiSMASH 7.0: new and improved predictions for detection, regulation, chemical structures and visualisation. *Nucleic Acids Res*. Oxford University Press (OUP); 51:W46–502023;

28. . Pyrodigal: Python bindings and interface to Prodigal, an efficient method for gene prediction in prokaryotes. *Journal of Open Source Software*. doi: 10.21105/joss.04296.

29. Camargo AP, Roux S, Schulz F, Babinski M, Xu Y, Hu B, et al.. Identification of mobile genetic elements with geNomad. *Nat Biotechnol*. Springer Science and Business Media LLC; 42:1303–122024;

30. Köster J, Rahmann S. Snakemake--a scalable bioinformatics workflow engine. *Bioinformatics*. Oxford University Press (OUP); 28:2520–22012;

31. UniProt Consortium. UniProt: The universal protein knowledgebase in 2023. *Nucleic Acids Res*. Oxford University Press (OUP); 51:D523–312023;

32. Steinegger M, Söding J. MMseqs2 enables sensitive protein sequence searching for the analysis of massive data sets. *Nat Biotechnol*. 35:1026–82017;

33. Chawla NV, Bowyer KW, Hall LO, Kegelmeyer WP. SMOTE: Synthetic minority over-sampling technique. *J Artif Intell Res*. AI Access Foundation; 16:321–572002;

34. Pedregosa F, Varoquaux G, Gramfort A, Michel V, Thirion B, Grisel O, et al.. Scikit-learn: Machine Learning in Python. arXiv [cs.LG].

35. Chen Z, Zhao P, Li F, Leier A, Marquez-Lago TT, Wang Y, et al.. iFeature: a Python package and web server for features extraction and selection from protein and peptide sequences. *Bioinformatics*. 34:2499–5022018;

36. Guan J, Chen Y, Goh Y-X, Wang M, Tai C, Deng Z, et al.. TADB 3.0: an updated database of bacterial toxin-antitoxin loci and associated mobile genetic elements. *Nucleic Acids Res*. Oxford University Press (OUP); 52:D784–902024;

37. Zhang J, Guan J, Wang M, Li G, Djordjevic M, Tai C, et al.. SecReT6 update: a comprehensive resource of bacterial Type VI Secretion Systems. *Sci China Life Sci*. Springer Science and Business Media LLC; 66:626–342023;

38. Liu Y, Liu S, Pan Z, Ren Y, Jiang Y, Wang F, et al.. PAT: a comprehensive database of prokaryotic antimicrobial toxins. *Nucleic Acids Res*. Oxford University Press (OUP); 51:D452–92023;

39. Harms A, Liesch M, Körner J, Québatte M, Engel P, Dehio C. A bacterial toxin-antitoxin module is the origin of inter-bacterial and inter-kingdom effectors of Bartonella. *PLoS Genet*. 13:e10070772017;

40. Yadav SK, Magotra A, Ghosh S, Krishnan A, Pradhan A, Kumar R, et al.. Immunity proteins of dual nuclease T6SS effectors function as transcriptional repressors. *EMBO Rep*. EMBO; 22:e518572021;

41. Danov A, Segev O, Bograd A, Ben Eliyahu Y, Dotan N, Kaplan T, et al.. Toxinome-the bacterial protein toxin database. *MBio*. 15:e01911232024;

42. Sordo M, Zeng Q. On sample size and classification accuracy: A performance comparison. *Biological and Medical Data Analysis*. Berlin, Heidelberg: Springer Berlin Heidelberg; p. 193–201.

43. Liu B, Zheng D, Zhou S, Chen L, Yang J. VFDB 2022: a general classification scheme for bacterial virulence factors. *Nucleic Acids Res*. Oxford University Press (OUP); 50:D912–72022;

44. Arango-Argoty G, Garner E, Pruden A, Heath LS, Vikesland P, Zhang L. DeepARG: a deep learning approach for predicting antibiotic resistance genes from metagenomic data. *Microbiome*. 6:232018;

45. Feldgarden M, Brover V, Gonzalez-Escalona N, Frye JG, Haendiges J, Haft DH, et al.. AMRFinderPlus and the Reference Gene Catalog facilitate examination of the genomic links among antimicrobial resistance, stress response, and virulence. *Sci Rep*. Springer Science and Business Media LLC; 11:127282021;

46. Ugarcina Perovic S, Ramji V, Chong H, Duan Y, Maguire F, Coelho LP. argNorm: normalization of antibiotic resistance gene annotations to the Antibiotic Resistance Ontology (ARO). *Bioinformatics*. Oxford University Press (OUP); 2025; doi: 10.1093/bioinformatics/btaf173.

47. Chicco D, Jurman G. The advantages of the Matthews correlation coefficient (MCC) over F1 score and accuracy in binary classification evaluation. *BMC Genomics*. Springer Science and Business Media LLC; 21:62020;

48. Chen C, Xu Y, Ouyang J, Xiong X, Łabaj PP, Chmielarczyk A, et al.. VirulentHunter: deep learning-based virulence factor predictor illuminates pathogenicity in diverse microbial contexts. *Brief*

Bioinform. Oxford University Press (OUP); 26:bbaf2712025;

49. Gupta A, Malwe AS, Srivastava GN, Thoudam P, Hibare K, Sharma VK. MP4: a machine learning based classification tool for prediction and functional annotation of pathogenic proteins from metagenomic and genomic datasets. *BMC Bioinformatics*. Springer Science and Business Media LLC; 2022; doi: 10.1186/s12859-022-05061-7.

50. Sharma A, Garg A, Ramana J, Gupta D. VirulentPred 2.0: An improved method for prediction of virulent proteins in bacterial pathogens. *Protein Sci*. Wiley; 2023; doi: 10.1002/pro.4808.

51. Xie R, Li J, Wang J, Dai W, Leier A, Marquez-Lago TT, et al.. DeepVF: a deep learning-based hybrid framework for identifying virulence factors using the stacking strategy. *Brief Bioinform*. Oxford University Press (OUP); 2021; doi: 10.1093/bib/bbaa125.

52. Sharma N, Naorem LD, Jain S, Raghava GPS. ToxinPred2: an improved method for predicting toxicity of proteins. *Brief Bioinform*. Oxford University Press (OUP); 23:bbac1742022;

53. Morozov V, Rodrigues CHM, Ascher DB. CSM-Toxin: A web-server for predicting protein toxicity. *Pharmaceutics*. MDPI AG; 15:4312023;

54. Kaper JB, Nataro JP, Mobley HL. Pathogenic Escherichia coli. *Nat Rev Microbiol*. Springer Science and Business Media LLC; 2:123–402004;

55. Johnson TJ, Nolan LK. Pathogenomics of the virulence plasmids of Escherichia coli. *Microbiol Mol Biol Rev*. American Society for Microbiology; 73:750–742009;

56. Henderson B, Martin A. Bacterial virulence in the moonlight: multitasking bacterial moonlighting proteins are virulence determinants in infectious disease. *Infect Immun*. American Society for Microbiology; 79:3476–912011;

57. Henderson B, Martin A. Bacterial moonlighting proteins and bacterial virulence. *Curr Top Microbiol Immunol*. 358:155–2132013;

58. Pancholi V, Fischetti VA. A major surface protein on group A streptococci is a glyceraldehyde-3-phosphate-dehydrogenase with multiple binding activity. *J Exp Med*. Rockefeller University Press; 176:415–261992;

59. Henderson B, Martin A. Bacterial moonlighting proteins and bacterial virulence. *Curr Top Microbiol Immunol*. Curr Top Microbiol Immunol; 358:155–2132013;

60. Henderson B, Martin A. Bacterial virulence in the moonlight: multitasking bacterial moonlighting proteins are virulence determinants in infectious disease. *Infect Immun*. American Society for Microbiology; 79:3476–912011;

61. Barel M, Charbit A. Detection of the interaction between host and bacterial proteins: eukaryotic nucleolin interacts with Francisella elongation factor Tu. *Methods Mol Biol*. Methods Mol Biol; 1197:123–392014;

62. Granato D, Bergonzelli GE, Pridmore RD, Marvin L, Rouvet M, Corthésy-Theulaz IE. Cell surface-associated elongation factor Tu mediates the attachment of Lactobacillus johnsonii NCC533 (La1) to human intestinal cells and mucins. *Infection and immunity*. Infect Immun; 2004; doi: 10.1128/iai.72.4.2160-2169.2004.

63. Kamiya S, Yamaguchi H, Osaki T, Taguchi H. A virulence factor of Helicobacter pylori: role of heat

shock protein in mucosal inflammation after H. pylori infection. *J Clin Gastroenterol*. 27 Suppl 1:S35–91998;

64. Hickey TBM, Ziltener HJ, Speert DP, Stokes RW. Mycobacterium tuberculosis employs Cpn60.2 as an adhesin that binds CD43 on the macrophage surface: M. tuberculosis Cpn60.2 mediates macrophage binding via CD43. *Cell Microbiol*. Hindawi Limited; 12:1634–472010;

65. Lehner T, Bergmeier LA, Wang Y, Tao L, Sing M, Spallek R, et al.. Heat shock proteins generate β-chemokines which function as innate adjuvants enhancing adaptive immunity. *Eur J Immunol*. 30:594–6032000;

66. UniProt Consortium. UniProt: The universal protein knowledgebase in 2025. *Nucleic Acids Res*. Oxford University Press (OUP); 53:D609–172025;

67. Eddy SR. Profile hidden Markov models. *Bioinformatics*. Oxford University Press (OUP); 14:755–631998;

68. Larralde M, Zeller G. PyHMMER: a Python library binding to HMMER for efficient sequence analysis. *Bioinformatics*. Oxford Academic; 39:btad2142023;

 Click here to access/download;Table;Table1.pdf

Table 1. Runtime Comparison of PathoFact 2.0 and VirulentHunter

Number of protein sequences	VirulentHunter	PathoFact 2.0				
	1 GPU	1 CPU	2 CPU	4 CPU	6 CPU	8 CPU
500	2 min 21 s	3.7 s	2.6 s	2.0 s	1.9 s	1.8 s
5500	25 min 42 s	29.9 s	17.4 s	10.2 s	8.1 s	7.4 s
10000	54 min 11 s	55.4 s	29.3 s	16.7 s	13.1 s	11.5 s
30000	2 h 45 min 50 s	2 min 45 s	1 min 32.9 s	52.1 s	40.3 s	36.0 s

Figure 1. Schematic representation of PathoFact2

Click here to access/download;Figure;Figure1.pdf

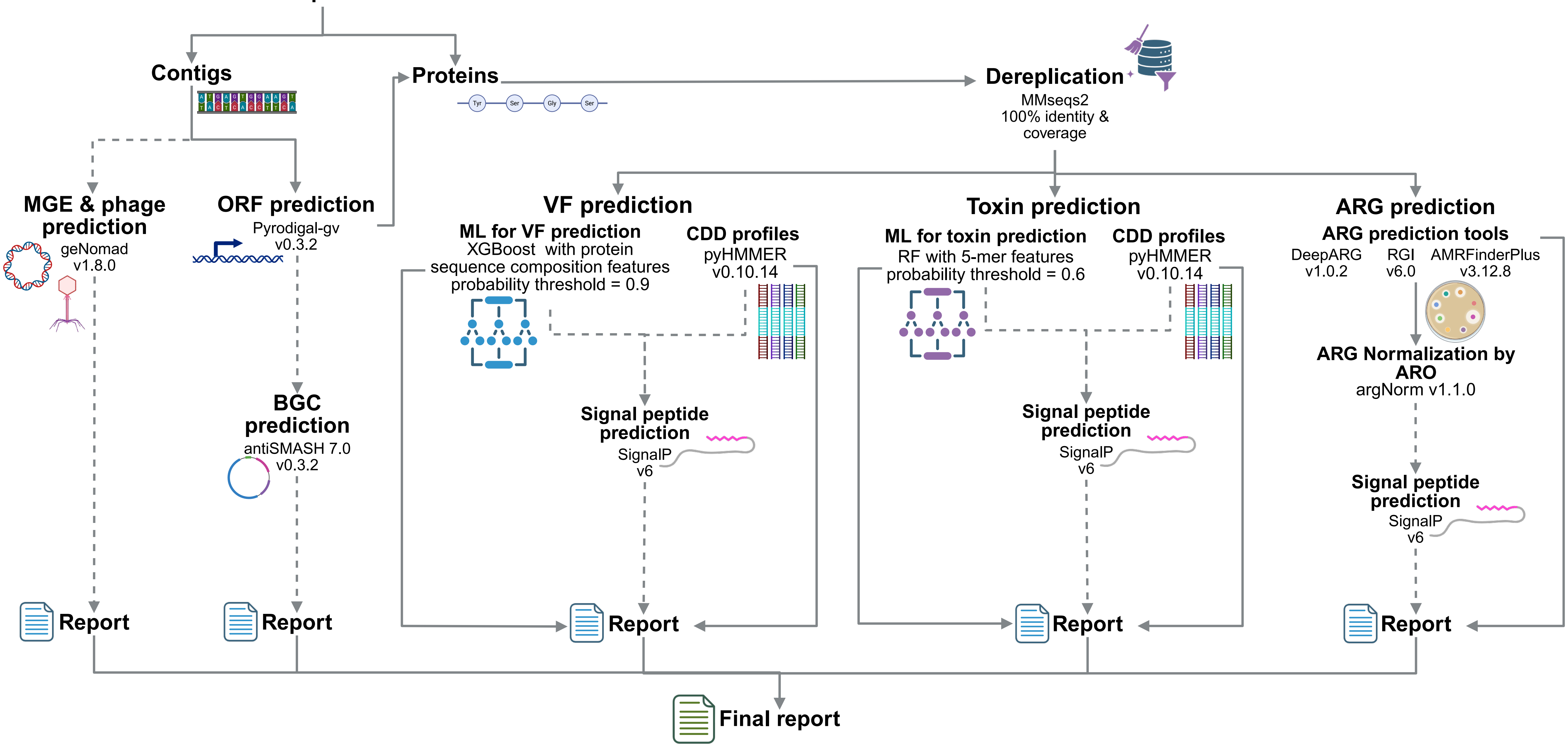

Figure 2. Schematic representation of the datasets used for PathoFact 2.0 toxin-associated and virulence factors modules training and testing.

Click here to access/download;Figure;Figure2.pdf

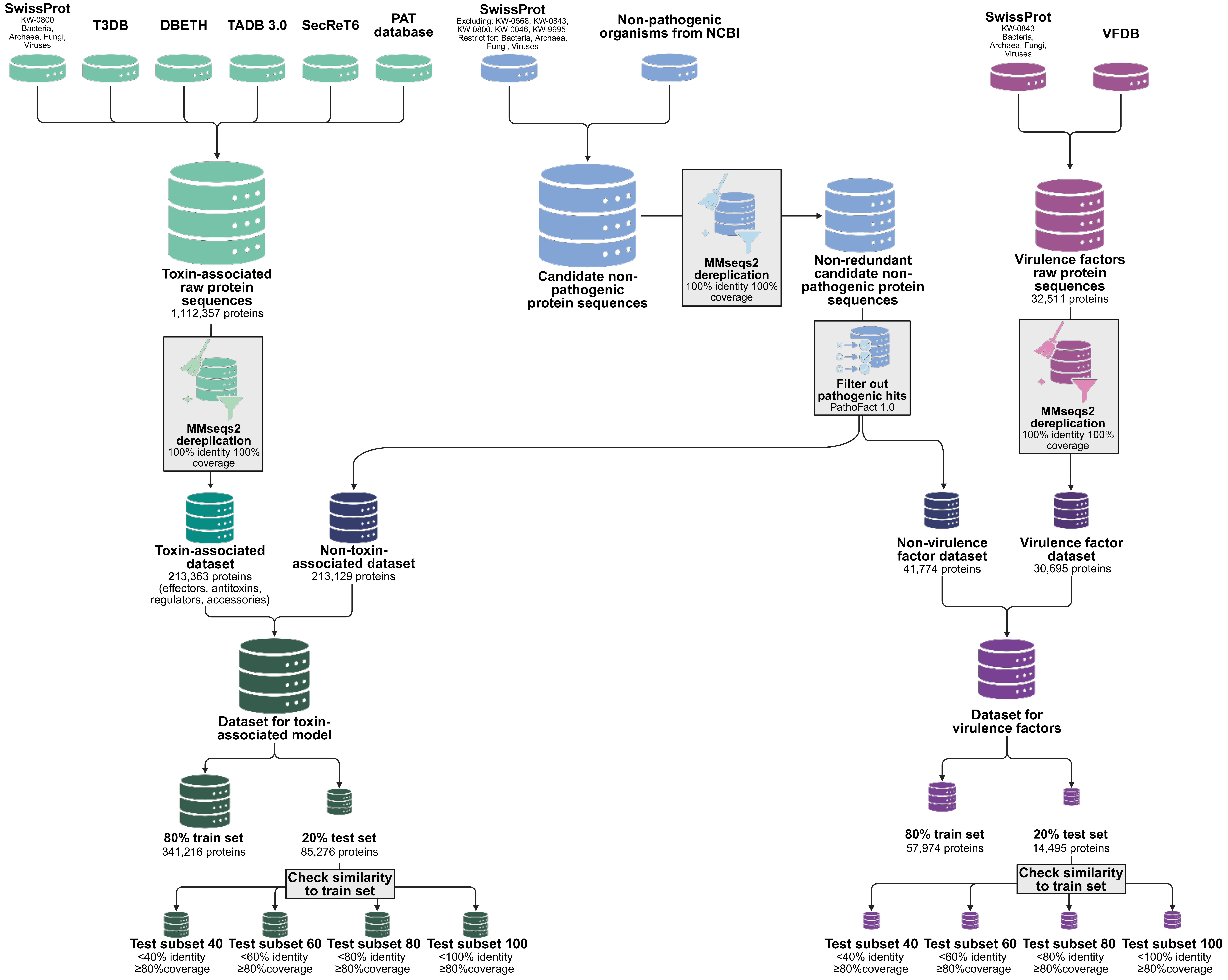

Figure 3. Schematic representation of the construction of the datasets used for benchmarking

Click here to access/download;Figure;Figure3.jpg

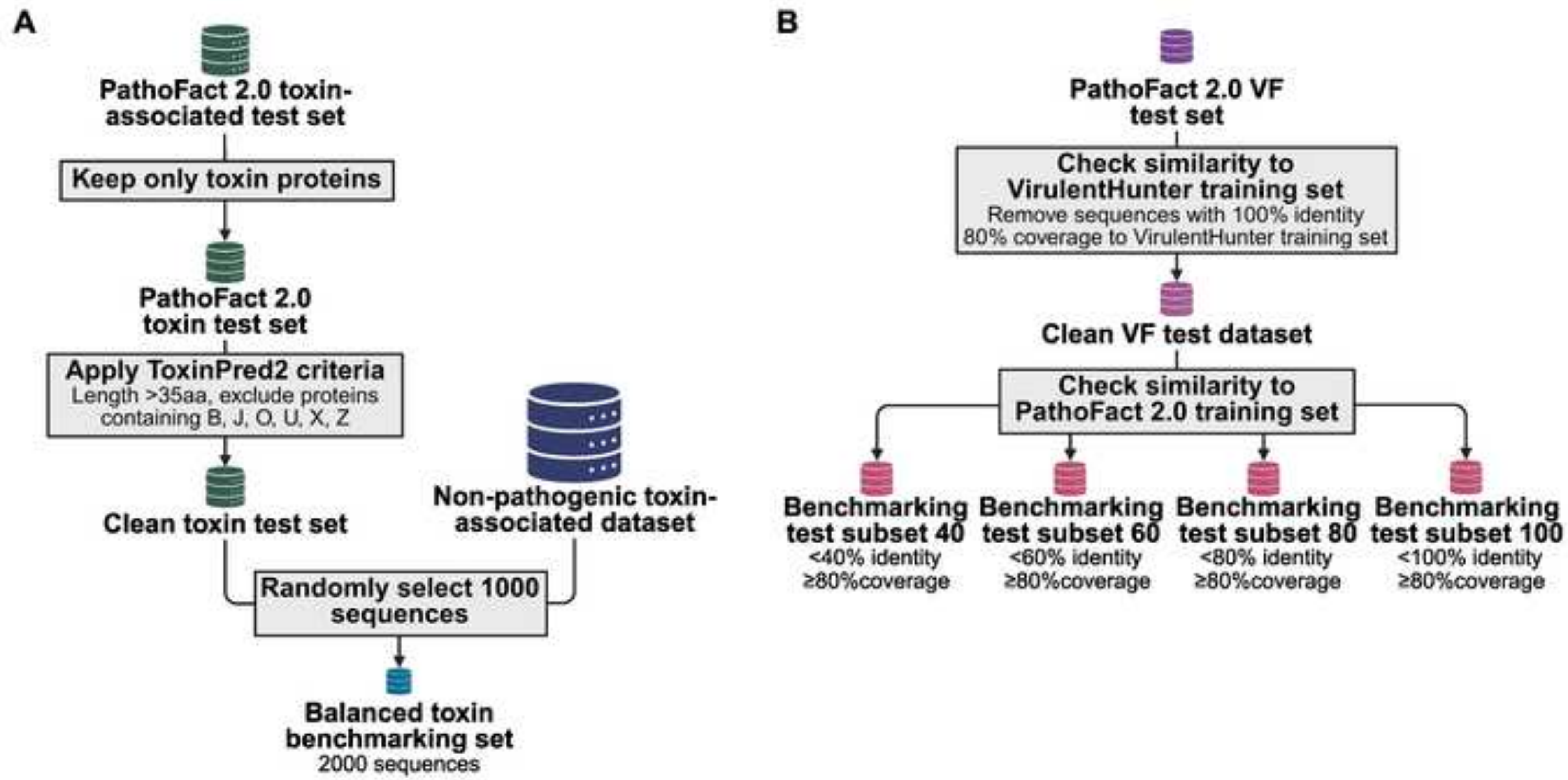

Figure 4. Performance evaluation of toxin-associated and virulence factor prediction modules across probability thresholds

Click here to access/download;Figure;Figure4.pdf

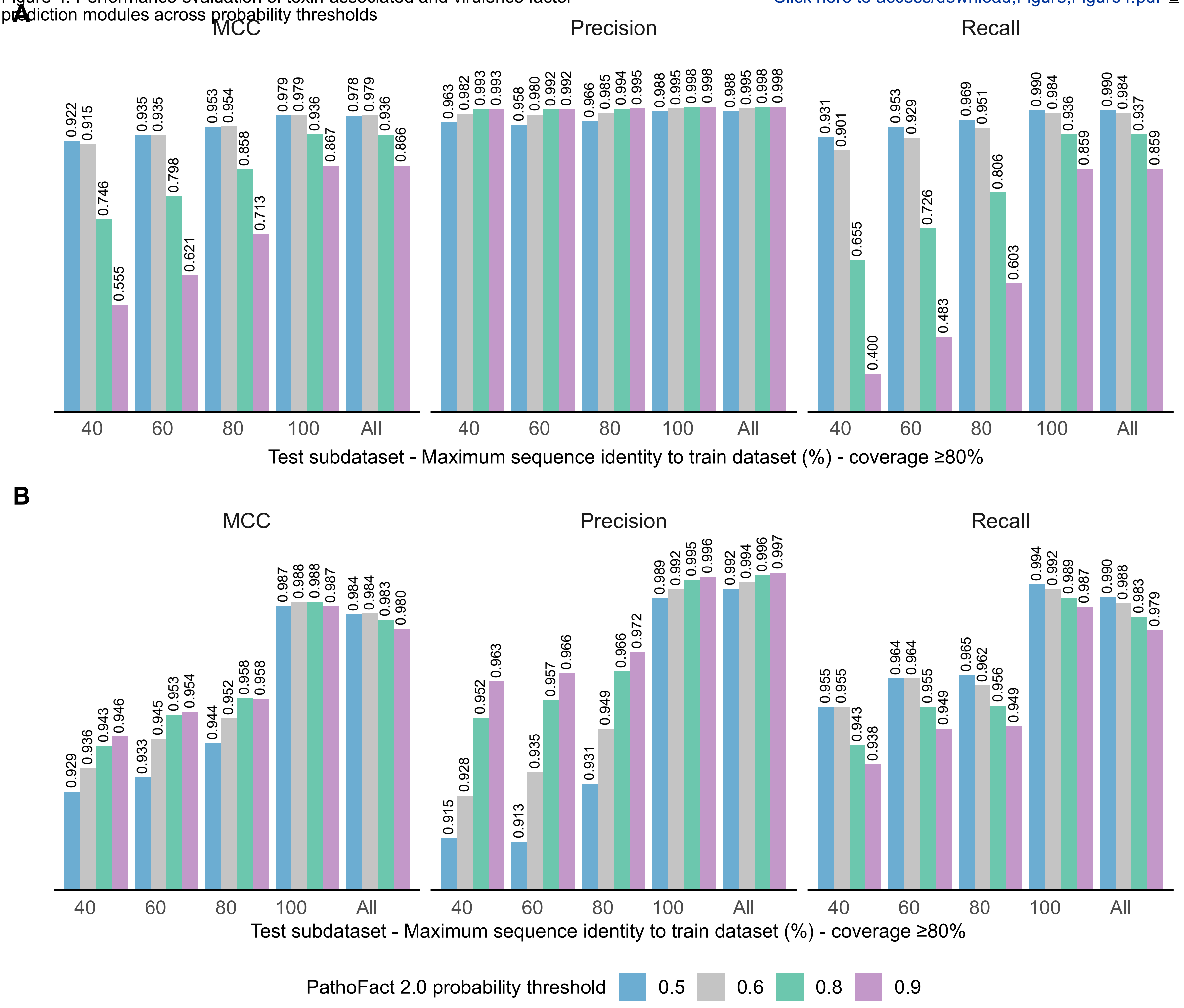

Figure 5. Benchmarking of toxin and virulence factor prediction performance.

Click here to access/download;Figure;Figure5.pdf

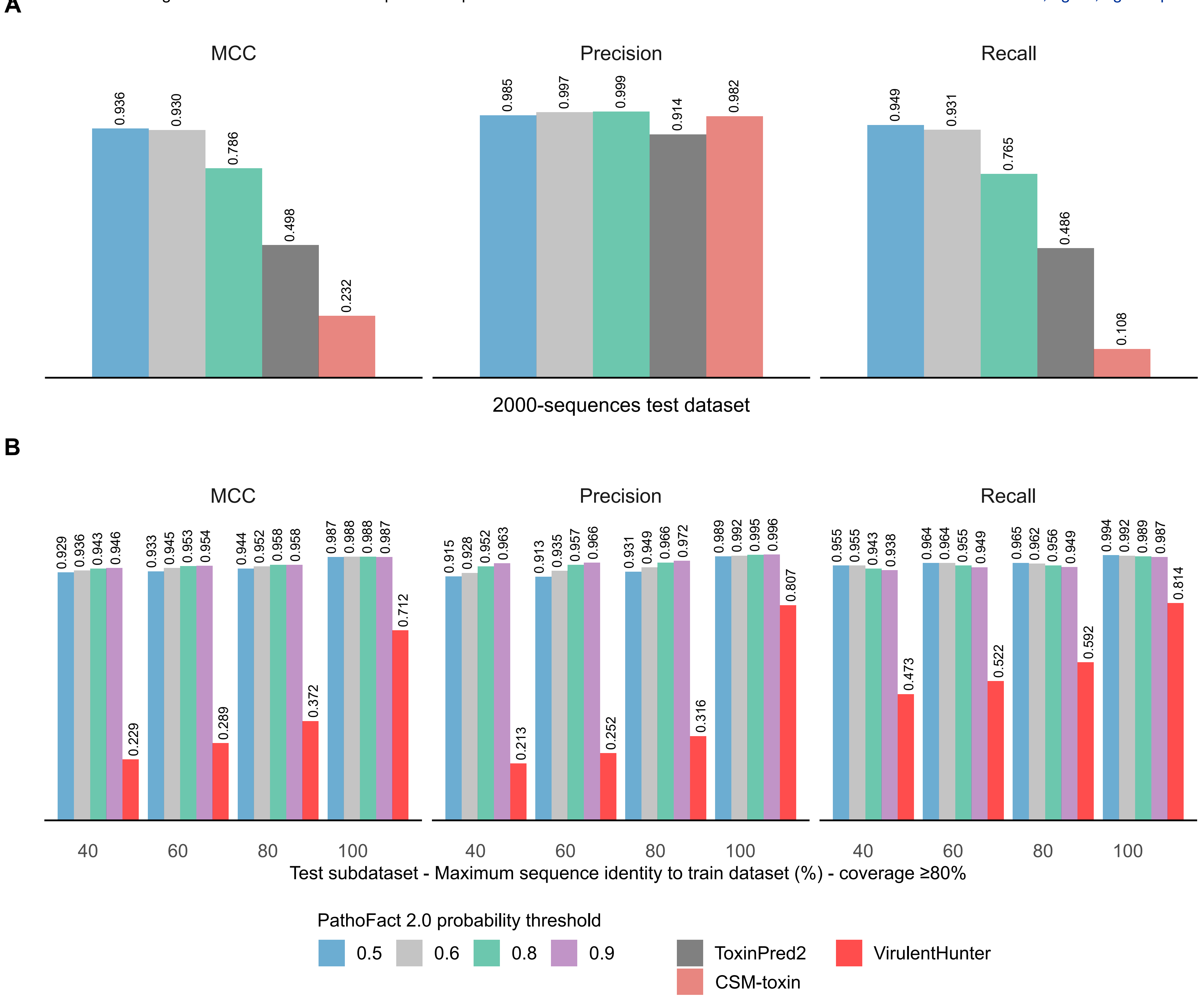

Figure 6. Comparative analysis of toxin-associated and virulence factor profiles in non-pathogenic and pathogenic Escherichia coli strains

Click here to access/download;Figure;Figure6.pdf

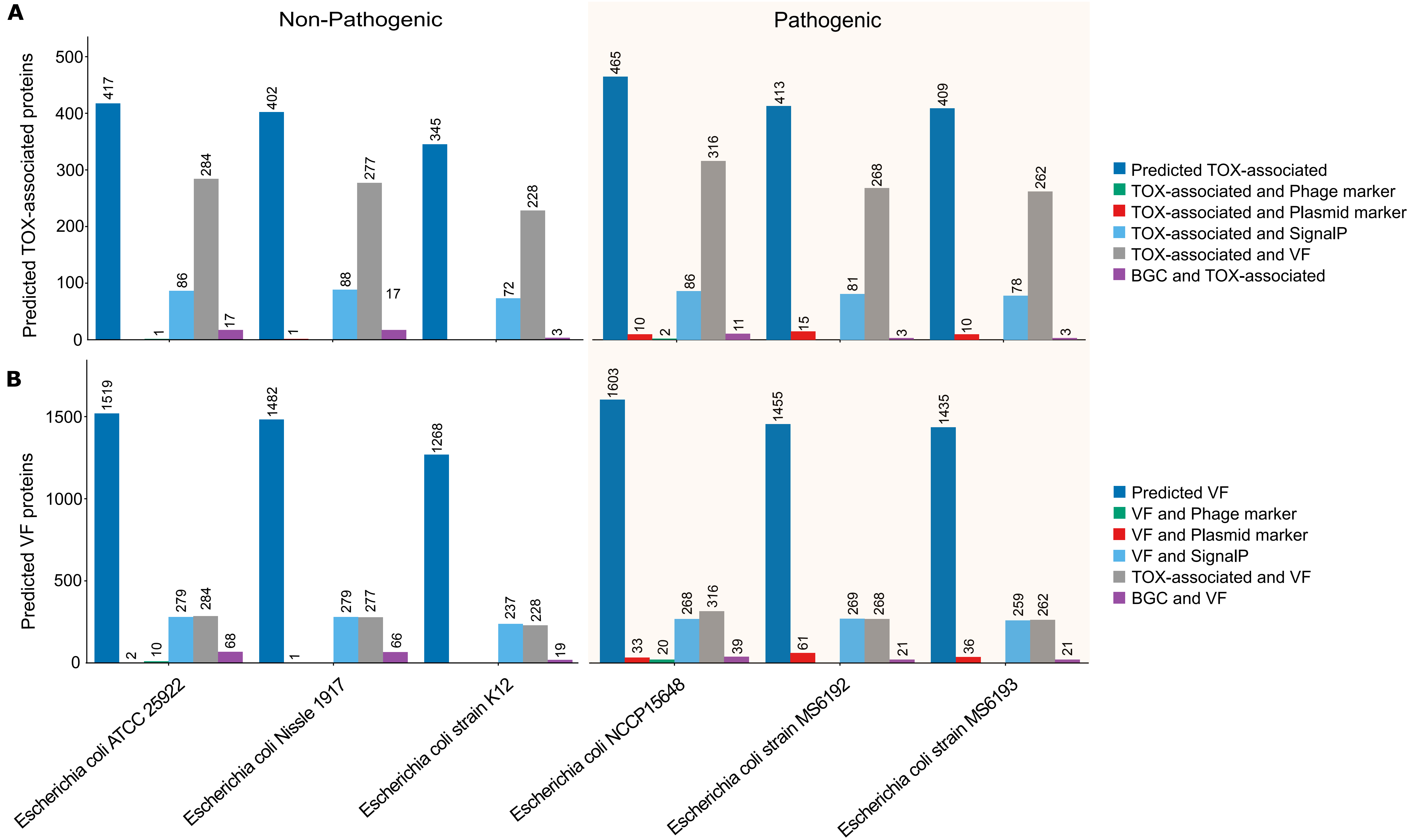

Figure 7. Performance comparison of virulence factor prediction tools in pathogenic and non-pathogenic

Click here to access/download;Figure;Figure7.pdf

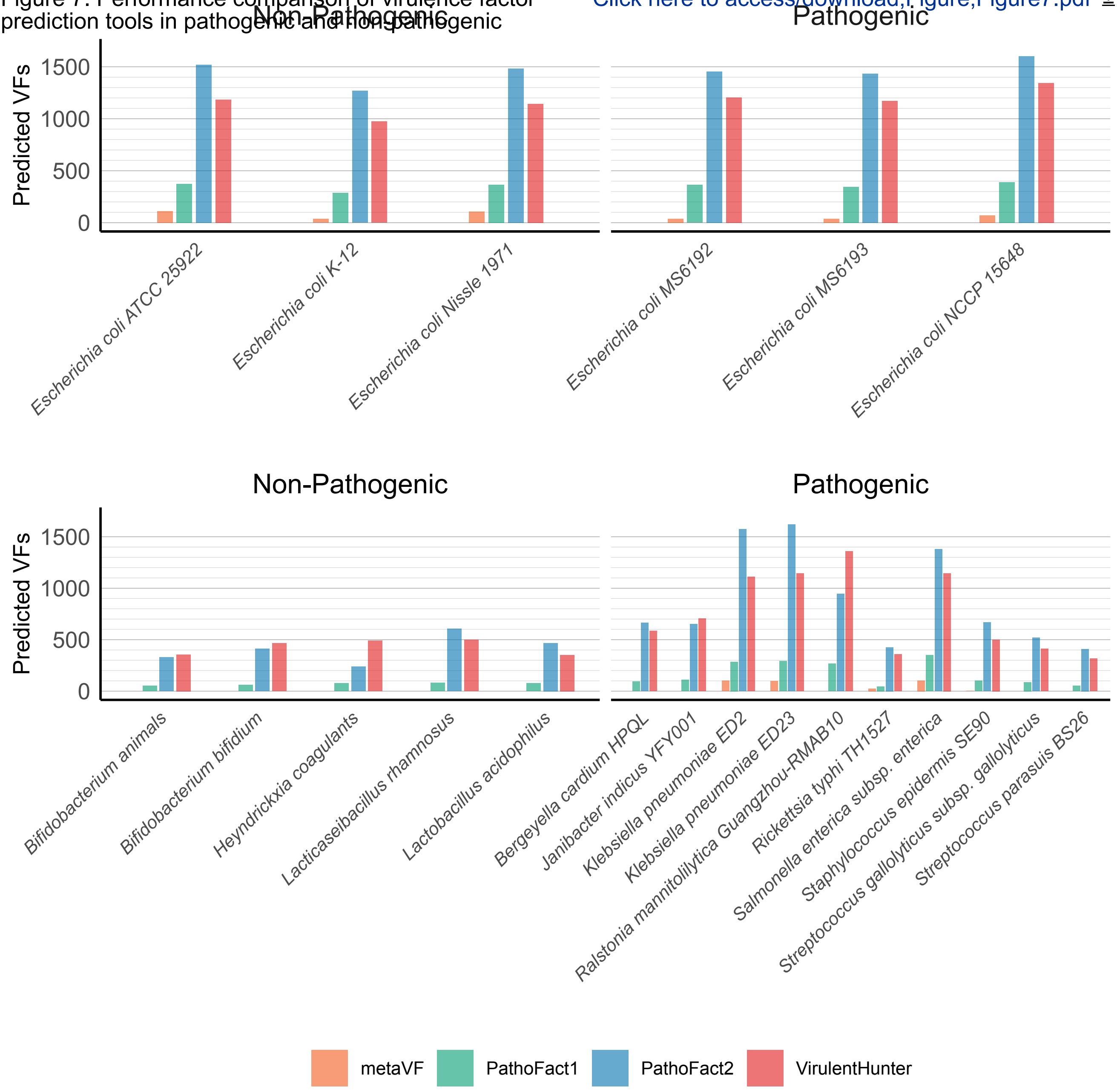

Figure 8. Performance comparison of toxin prediction tools in pathogenic and non-pathogenic strains.

Click here to access/download;Figure;Figure8.pdf

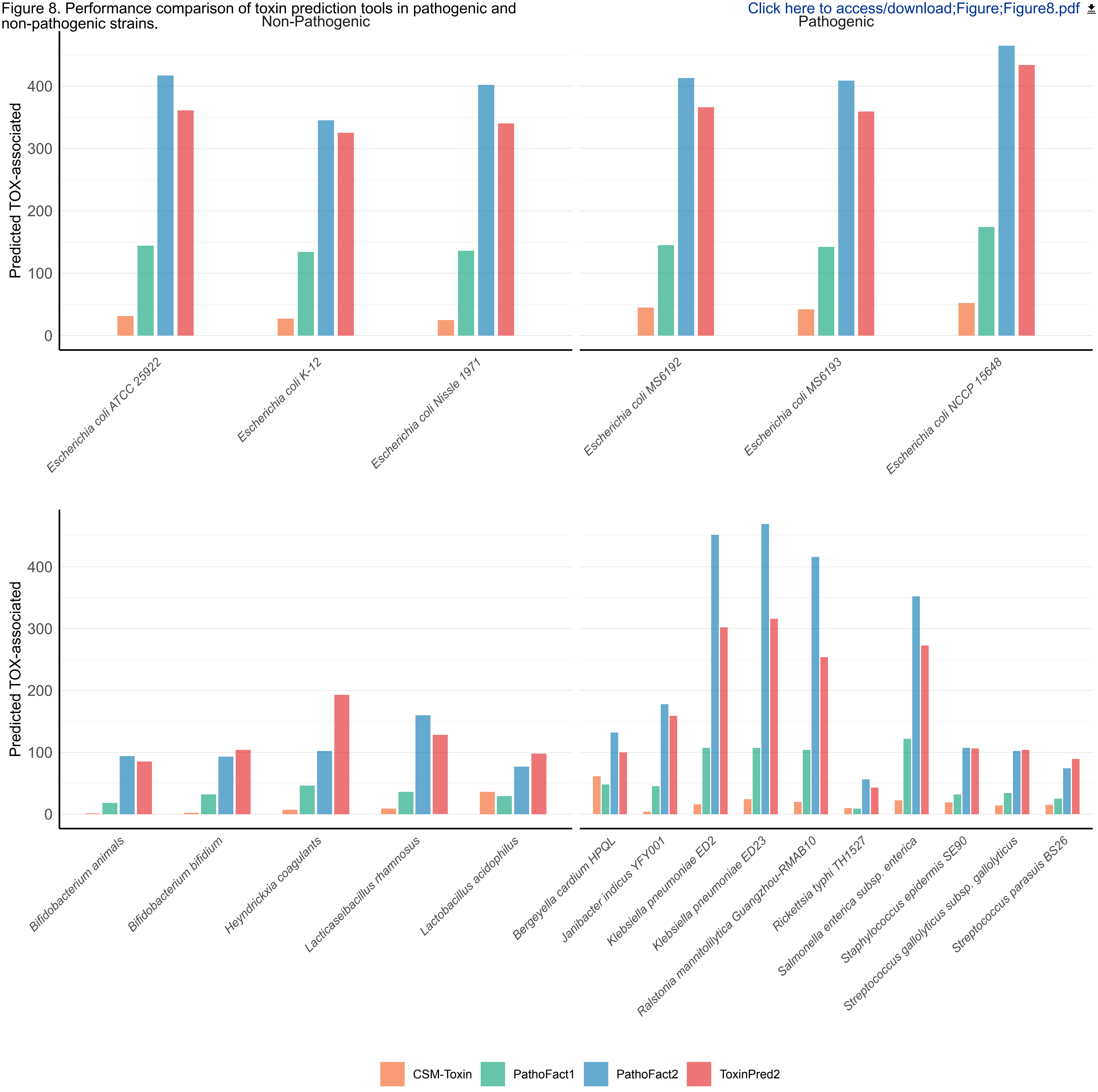

Supplementary Table S1. List of microorganisms non-pathogenic to humans and their total protein count obtained from the NCBI

Click here to access/download

Supplementary Material

SupplementaryTableS1_ListOfMicroorganismsFromNCBI.xlsx

Supplementary Table S2. Bacterial strains used in this study,
including their classification as pathogenic or non-pathogenic,

Click here to access/download
Supplementary Material
SupplementaryTableS2_BacterialStrains_with_AccessionNumbers.tsv

Supplementary Table S3. Evaluation of the PathoFact 2.0 toxin prediction module

Click here to access/download
Supplementary Material
SupplementaryTableS3_PathoFact2_subset_Test_module_Toxin.xlsx

Supplementary Table S4. Evaluation of the PathoFact 2.0 virulence factor prediction module. The table summarises

Click here to access/download
Supplementary Material
SupplementaryTableS4_PathoFact2_subset_Test_module_VF.xlsx

Supplementary Table S5. Comparison of virulence factor prediction performance for PathoFact 2.0 at varying probability

Click here to access/download
Supplementary Material
SupplementaryTableS5_Benchmarking_VF_protein_input.xlsx

Supplementary Table S6. Comparison of toxin protein prediction performance for PathoFact 2.0 at different prediction probability

Click here to access/download
Supplementary Material
SupplementaryTableS6_Benchmarking_Toxins_protein_input.xlsx

Supplementary Figure S1. Comparative analysis of toxin-
associated and virulence factor profiles in non-pathogenic and

Click here to access/download
Supplementary Material
SupplementaryFigureS1.pdf

Supplementary Figure S2. Comparative performance of Pathofact 2.0 (blue) versus PathoFact1 (green) and metaVF (red) in

Click here to access/download
Supplementary Material
SupplementaryFigureS2.pdf

Supplementary Figure S3. Comparative performance of Pathofact 2.0 (blue) versus PathoFact (green) and metaVF (red) in predicting

Click here to access/download
Supplementary Material
SupplementaryFigureS3.pdf

Supplementary Figure S4. Performance comparison of PathoFact 2.0 versus PathoFact for ARG and toxin prediction in Escherichia

Click here to access/download
Supplementary Material
SupplementaryFigureS4.pdf

Supplementary Figure S5. Performance comparison of PathoFact 2.0 versus PathoFact for ARG and toxin prediction in bacterial

Click here to access/download
Supplementary Material
SupplementaryFigureS5.pdf

Personal Cover

Click here to access/download;Personal Cover;CoverLetter_PathoFact.pdf

Dr. Weixue Mu
Editor
GigaScience
BGI Shenzhen
China

Paul Wilmes
Professor of Systems Ecology
Luxembourg Centre for Systems Biomedicine
University of Luxembourg
7, avenue des Hauts-Fourneaux
L-4362 Esch-sur-Alzette
Luxembourg

6 March 2026

Dear Dr. Mu,

We thank you and the reviewers for the careful and constructive evaluation of our manuscript entitled "**PathoFact 2.0: An Integrative Pipeline for the Prediction of Antimicrobial Resistance Genes, Virulence Factors, Toxins and Toxin-associated Proteins, and Biosynthetic Gene Clusters in Metagenomes**". We are grateful for the detailed feedback, which has significantly improved the clarity, rigor, and usability of both the manuscript and the software.

We are pleased to submit a revised version of the manuscript that addresses all reviewers' and editor's comments. We refined the text for accuracy and scope, improved figure/table clarity and formatting, and expanded/streamlined the Methods to more clearly describe dataset construction and benchmarking.

As requested, we registered the software and workflow. We have added the identifiers to the manuscript: RRID SCR027650, bio.tools ID pathofact2, and WorkflowHub DOI 10.48546/workflowhub.workflow.2087.1. We also clarified limitations and maintenance plans, including current Linux-first support due to third-party dependency constraints on Apple silicon chips and planned future module additions.

We believe these revisions fully address the reviewers' concerns and strengthen the manuscript. We confirm that the work is original, not under consideration elsewhere, and that all authors have approved the revised submission.

Thank you for your consideration.

Yours sincerely,

Paul Wilmes, corresponding author
Email: paul.wilmes@uni.lu

Reviewer reports:

We would like to thank all the reviewers for their thorough review of the manuscript. We appreciate their comments and believe that these contributions have significantly improved the paper. Below are our responses to each comment, along with the corresponding manuscript text for readability. In addition to the clean, updated manuscript submitted with our reviewer response, we have included an updated version that highlights our edits and additions to make the changes easy to identify.

Editor comments:

1. In addition, please register any new software application in the bio.tools and SciCrunch.org databases to receive RRID (Research Resource Identification Initiative ID) and biotoolsID identifiers, and include these in your manuscript. Computational workflows should be registered in workflowhub.eu and the DOIs cited in the relevant places in the manuscript. These will facilitate tracking, reproducibility and re-use of your tool.

R/ We thank you for this suggestion. We have now included the RRID, which is SCR_027650. We have also included the bio.tools ID: pathofact2 and the DOI for workflowhub.eu: 10.48546/workflowhub.workflow.2087.1

Reviewer #1:
The pipeline should be very useful on shotgun metagenomics data analysis.
Aside the ARGs and VFs, the features on signal peptides, toxin predictions, and BGCs in particular for specialised metabolites predictions, are welcome for detailed analysis and understanding of various transmission mechanisms.
I find the approaches very appealing and I think the pipeline could be welcomed by the community.

R/ We acknowledge and thank the reviewer for their recognition of our work and their overall positive feedback.

I only have some minor observations:
2. The Methods section should be placed next to the described methods. As it is, at the end of the manuscript, under Methods chapter you can only find Datasets, so a proper formatting of the Methods is required

R/ We thank the reviewer for the comment. We followed the journal's Technical Application Guidance: "For Technical Notes, this section is specific for including any additional methods used in the manuscript that are not part of the new work being described in the manuscript."
Nonetheless and to account for the reviewer's comment, we have expanded the Methods section accordingly (lines 494-558):

> *"Databases used for the PathoFact 2.0 Dataset Construction*
> - SwissProt [31] is the expertly curated part of UniProtKB [66]. It offers high-quality protein sequences with detailed functional annotations, including keywords for pathogenesis, virulence, toxins, and antibiotic resistance.

- VFDB [43], the Virulence Factor Database, is a comprehensive reference for curating information on virulence factors of bacterial pathogens.
- T3DB [12], the toxin and toxin-target database, is a resource cataloguing thousands of toxins and their protein targets, with detailed mechanisms, structures, and toxicity data, including bacterial protein toxins.
- DBETH [13], the database for bacterial exotoxins, is a specialised database of bacterial exotoxins pathogenic to humans, classified into 24 mechanistic and activity types from 26 bacterial genera.
- TADB [36], the toxin-antitoxin database, is a repository of bacterial toxin-antitoxin loci across types I-VIII, including experimentally validated pairs, predicted loci, and associations with mobile genetic elements.
- SecReT6 [37] is a database containing known and predicted type VI secretion systems, including effectors, immunity proteins, regulators, and accessory proteins from bacterial genomes.
- PAT [38] is the prokaryotic antimicrobial toxin database and contains a collection of antimicrobial toxins, including bacteriocins and effectors from secretion systems.

Clustering Parameters

MMseqs2 [32] is an ultra-fast open-source software suite for sensitive protein and nucleotide sequence searching and clustering, up to 10,000 times faster than BLAST while retaining comparable sensitivity. For dereplication, it clusters sequences at 100% identity and 100% coverage using the parameters -c 1.0 and --min-seq-id 1.0. It removes exact duplicates and retains representative sequences to generate non-redundant datasets. To create the test subsets, a coverage of 80% (-c 0.8) and identities ranging from 40 - 100% (--min-seq-id 0.4, --min-seq-id 0.6, --min-seq-id 0.8, --min-seq-id 1.0) were used, and then sequences with similarity higher than min-seq-id were removed for each test subset. In all cases, the cluster mode and coverage mode used were 0 (--cov-mode 0 --cluster-mode 0). When --cov-mode 0 is specified in combination with -c values ranging from 0.0 to 1.0, sequences are assigned to the same cluster only if the alignment spans at least a fraction *c* of the length of the longer sequence. According to the developers of MMSeq2, this coverage criterion is particularly suitable for clustering full-length protein sequences.

Protein Composition Features

Protein sequence composition features were extracted to represent each protein as fixed-length vectors derived from its primary amino acid sequence [35]. These included amino acid composition (AAC), dipeptide composition (DPC), composition (CTDC), transition (CTDT) and distribution (CTDD). AAC captures the relative frequency of each of the 20 amino acids in a sequence, whereas DPC captures the relative frequency of all adjacent amino-acid pairs (400 possible dipeptides). CTDC represents the percentage of amino acids belonging to each of three predefined groups (polar, neutral, hydrophobic) in the entire protein sequence. CTDT represents the percentage frequency with which a residue of one group is followed by a residue of a different group along the sequence. CTDD represents the distribution of each amino acid group, measuring the spatial position, where the first, 25%, 50%, 75% and 100% of the residues of a specific class are located.

Performance and Evaluation Metrics

To assess the models' performance, we used a confusion matrix comprising true positives (TP), true negatives (TN), false positives (FP), and false negatives (FN), computed on the test datasets described above. TP corresponds to truly positive instances correctly predicted as positive by the model, whereas TN corresponds to truly negative instances correctly predicted as negative. FP are truly negative instances incorrectly predicted as positive, and FN are truly positive instances incorrectly predicted as negative. From these values, we calculated the following metrics:

$$Accuracy = (TP + TN)/(TP + TN + FP + FN)$$

$$Precision = TP/(TP + FP)$$

$$Recall = TP/(TP + FN)$$

$$MCC = (TP \times TN - FP \times FN)/\sqrt{(TP + FP)(TP + FN)(TN + FP)(TN + FN)}$$

HMM Profiles

Profile hidden Markov models are probabilistic models built from a multiple sequence alignment that encode, for each alignment position, the position-specific probabilities of residues and insertions/deletions, turning the alignment into a position-specific scoring system for detecting homologous sequences [67]. The FASTA files of conserved-domain multiple sequence alignments for each CDD [26] family (https://ftp.ncbi.nih.gov/pub/mmdb/cdd/fasta.tar.gz) were downloaded. Pyhmmer v0.10.14 [68] was used to obtain HMM profiles for each CDD family and to perform protein sequence searches against the CDD HMM family profiles.

3. there is a 70 blank pages buffer between References and supplementary data

R/ This occurred during the submission of the manuscript to Gigascience. This is therefore outside of our control.

4. could you add some future prospects in the manuscript? How well is it going to be maintained - I noticed the update are quite old.

R/ We thank the reviewer for this helpful comment. Updates are prompted by user-reported issues, newly identified errors, and the availability of enhanced reference datasets or prediction models. A new version of PathoFact is scheduled for the future, which will include two new modules, one for biocide and environmental pollutants resistance and another for heavy metal resistance genes.

5. I would also add some more details to the limitations. For instance it is clear that the pipeline is installable on Linux platforms, but did you considered making it available also for Apple silicon series? More and more researchers use this technology, and it works as good as the Linux distributions. I also tried an install on a M series Apple silicon, but unfortunately, most of the tools in the pipeline lead to multiple errors related to python versions (most of which are old), missing old dependencies versions, libraries, etc.

R/ We thank the reviewer for the comment. PathoFact 2.0 integrates several third-party tools whose compatibility with Apple Silicon (ARM architecture) is currently limited. Because these components are developed and maintained by external groups, addressing compatibility issues is outside the scope of PathoFact 2.0.

PathoFact 2.0 uses Mamba/Conda, with most packages from the Bioconda channel, which has been actively expanding support for ARM architecture. However, not all required dependencies are currently available or stable for Apple Silicon systems, leading to installation and runtime errors related to Python version mismatches and missing legacy libraries, as noted by the reviewer.

For these reasons, we have chosen to officially support Linux platforms, where all dependencies are stable, well-maintained, and widely adopted within the bioinformatics community. Once the required third-party tools and their dependencies are fully supported on ARM architectures through Bioconda or other channels, PathoFact 2.0 can be made compatible with Apple Silicon systems. It is important to note that metagenomic data is usually large in size. To efficiently run bioinformatic metagenomic workflows and to store the data and results, they are typically kept on dedicated servers or high-performance computing (HPC) clusters. Therefore, PathoFact 2.0 has been designed to be run on HPCs.

Reviewer #2:
The authors present the pipeline PathoFact 2.0, which combines external modules and machine learning algorithms in order to find genes that provoke antimicrobial resistance, virulence and toxicity. They present their work, the improvement from the previous version, as a Technical Note.
For what the authors say, it is the only pipeline available with those characteristics, which makes it clearly a relevant software and article. However, I believe the article requires refinement, as well as new tests that support the authors claims.

R/ We acknowledge and thank the reviewer for their recognition of our work and their overall positive feedback. We further thank the reviewer for their constructive criticisms, which have allowed us to strengthen the work and the resulting manuscript.

Refinements on the article
6. In the abstract, ARG is used as an abbreviation for Antimicrobial Resistance, not Antimicrobial Resistance Genes.

R/ We thank the reviewer for the comment. This has now been corrected (lines 20-21):

> "Antimicrobial resistance genes (ARG) and virulence factors (VFs) are central contributors to the global health crisis surrounding drug-resistant infections."

7. Overall, the article should be more clear in what the pipeline is made for. It mentions fungi, viral, etc… sequences (which might be found on a metagenomic sample, of course), but to my understanding, all the tools and phenotypes searched for are mostly characteristic of bacteria.

R/ We thank the reviewer for this comment.

PathoFact 2.0 is explicitly designed for the analysis of metagenomic datasets, as stated in the title. In such datasets, prokaryotes (bacteria and archaea) represent the dominant fraction of organisms, and accordingly, most prediction modules in PathoFact 2.0 are optimised for bacterial proteins. The virulence factors, toxin-associated proteins, antimicrobial resistance genes, and secretion-related features targeted by the pipeline are therefore primarily bacterial in nature.

At the same time, metagenomic assemblies may contain sequences from viruses and eukaryotes (including fungi). For this reason, PathoFact 2.0 includes tools that can process non-bacterial sequences where appropriate. For example, protein prediction is performed using Prodigal-gv, which supports both prokaryotic and viral genomes; SignalP predicts signal peptides in proteins from archaea, bacteria, and eukaryotes; and antiSMASH includes dedicated modes for bacterial and fungal biosynthetic gene clusters. These components enable the pipeline to handle the mixed characteristics of metagenomic data.

We acknowledge that this duality, broad input acceptance versus primarily bacterial biological interpretation, was not sufficiently explicit in the current version of the manuscript.

We have added to the limitations of PathoFact 2.0 section the following paragraph (lines 471-473):

> “PathoFact 2.0 is designed for metagenomic samples; most prediction modules and phenotypes are bacterial-centric. Virulence, toxin-associated, and antimicrobial resistance predictions are particularly interpreted in the context of human pathogens”

8. While the introduction offers a good resume of the genes of interest, there are some descriptions that are not particularly accurate. "Human, animal, and environmental microbiomes harbour commensal and pathogenic microorganisms, contributing to the emergence of infectious diseases" seems to say that commensal microorganisms contribute to the emergence of infectious diseases;

R/ We thank the reviewer for the comment; it has been revised. We have modified the paragraph (Lines 40-47):

> “Microbiomes are highly complex and diverse ecological communities composed of bacteria, archaea, viruses, and microeukaryotes. These communities include both commensal microorganisms, which can contribute to host health, and pathogenic or opportunistic microorganisms that can cause disease under specific conditions. Microbial communities generally exist in synergistic relationships with their hosts, playing critical roles in maintaining physiological homeostasis and regulating immune function. However, disruption of this balanced microbial ecosystem, known as microbial dysbiosis, can impair normal body functions and has been associated with the development of various diseases, including cardiovascular diseases, cancers, and respiratory disorders [1].”

9. "ARGs are genetic elements that confer bacterial resistance to antibiotics, acquired via mutations or horizontal gene transfer." seems to say that antimicrobial resistance genes are acquired via mutation (they are not, there is a difference between resistance to antimicrobials provoked by mutations and by genes). I recommend a thorough rewriting of the 6 first paragraphs.

R/ We thank the reviewer for this relevant comment; It has been revised. The paragraph was modified (lines 50-51):

> "ARGs are genetic elements that confer bacterial resistance to antibiotics. Many ARGs are found on mobile genetic elements (MGEs) and are therefore often horizontally transmitted [4]".

10. The graphs in Figures 1 and 2 have different Y axes, which are also not shown. This is, to say it lightly, very misleading.

R/ We thank the reviewer for the comment; Figure 1 is a schema, so it seems the reviewer is referring to Figure 2 (A and B) in the previous manuscript's version (now Figure 4). Figure 4 has been updated to clearly show the Y-axis labels as figure titles.

11. Table 1 would be much more clear as a Figure. Table 1 cited in line 211 does not exist.

R/ We thank the reviewer for the comment. After trying out different figure types, we concluded that a table better represented our results than a figure. The reference to Table 1 has been removed (lines 179-180).

12. A short description of "dereplication" would help users lacking that knowledge.

R/ We thank the reviewer for the comment. In the first appearance of dereplication (both in figure 1 and pipeline structure section), and on several occasions in the manuscript, we included the following text: "proteins dereplicated by our tool to retain only non-redundant sequences (based on 100% identity and coverage). " Lines 131-132.

13. The description of the parameters of the machine learning modules are a copy-paste of the variables used by scikit-learn (lines 248 and 268). The description of the machine learning models should be clearer and more detailed, as well as not force the reader to go to the instructions of scikit-learn to check what is the meaning of those parameters.

R/ We thank the reviewer for the comment. As written in the manuscript (lines 170-172), we used XGBoost Python package (https://xgboost.readthedocs.io/en/stable/index.html) and the RandomForest (RF) Scikit-learn (version 1.5.2; [34]) to build our ML models. For the Random Forest model, the default hyperparameters provided by scikit-learn were used; for the XGBoost model, only the hyperparameters that differed from the defaults were explicitly specified. The parameter names were intentionally kept identical to those used in scikit-learn and the XGBoost Python package to ensure precision and reproducibility. These are standard hyperparameters of well-established models, and a detailed explanation of each is beyond the scope of this manuscript. The full definitions and usage of these parameters are clearly documented in the referenced XGBoost Python package as well as the scikit-learn documentation [34](RandomForest: https://scikit-learn.org/stable/modules/generated/sklearn.ensemble.RandomForestClassifier.html#sk

learn.ensemble.RandomForestClassifier; XGBoost: https://xgboost.readthedocs.io/en/stable/parameter.html), to which the reader is explicitly directed. In the manuscript lines 217-218:

> "The toxin-associated ML model is a RF with 5-mer features (default hyperparameter setting, i.e, n_estimators=100, max_depth=None, min_samples_split=2)."

and lines 237-238:

> "The VF model uses XGBoost with protein sequence composition features (learning_rate': 0.1, 'n_estimators': 2000)."

14. The authors analyze the performance of the models depending on the "probability" (a term that could definitely use a better introduction) using barplots. A standard to analyze the probability of a model is a ROC curve.

R/ We thank the reviewer for the comment. However, we respectfully point out that ROC curves are not always the most appropriate evaluation metric for highly imbalanced datasets, as is the case for virulence factor and toxin prediction.

In our study, the models produce a predicted class probability (referred to as "probability" in the manuscript) that reflects the model's likelihood that a given sequence belongs to the positive class. ROC curves primarily assess the trade-off between sensitivity and specificity across decision thresholds, but they can be misleading under strong class imbalance because a high true negative rate can inflate performance estimates even when positive class prediction is poor. We relied on the Matthews Correlation Coefficient (MCC), which is widely recognised as a robust and informative metric for imbalanced binary classification, as it accounts for all four confusion matrix components. As written in the manuscript (lines 280-284):

> "The performance evaluation is 280 based on the Matthew correlation coefficient (MCC) and the precision (to reduce the number of false positive VF and toxin-associated predictions), taking into account the dataset imbalance (a higher number of "non-toxin" and "non-VF" sequences compared to "toxin-associated" and "VF" sequences in the test subsets)."

Our bar plot across confidence score thresholds shows how prediction quality (measured by MCC and precision) changes as probability cutoffs are applied. This approach directly addresses the practical use case of selecting an operating threshold that balances sensitivity and precision in metagenomic discovery settings, rather than providing a single aggregate curve.

We believe this evaluation strategy is appropriate given the data characteristics and the use case addressed in this study.

We have added the following text to the manuscript to clarify the probability concept (Lines 274-276):

> "The VF and toxin-associated modules (Figure 1) were evaluated across different ML models-predicted probability thresholds, corresponding to the model's confidence in the

> positive class (VF or toxin-associated). Analyses were performed on the full test dataset and on subsets of the test dataset."

Improvements

15. The claims of the authors about the machine learning models for VF and toxin prediction being more accurate than similarity models is, to my understanding, not proved in the article. If it is only compared to PathoFact, which was created with a dataset made years ago, the higher performance could easily be because of more complete datasets. A fair comparison of an improved performance should be done with the same dataset (PathoFact but with the dataset collected for PathoFact 2.0). Moreover, the results only show that PathoFact 2.0 predicts more toxins and virulence factors than PathoFact.

R/ We thank the reviewer for the comment. PathoFact 2.0 supports two distinct types of input, and the benchmarking strategy differs accordingly:

Protein sequence input

For protein-level predictions, we benchmarked the machine-learning models implemented in PathoFact 2.0 against state-of-the-art tools for the same task: VirulentHunter (for virulence factors), ToxinPred2 and CSM-toxin (for toxins). These comparisons were conducted on independent test datasets to evaluate model generalisation.

Our toxin model is designed to predict toxin-associated proteins, rather than toxin proteins alone. We have made this distinction clear in the title of the manuscript (i.e., Toxin-associated) and throughout the manuscript. To our knowledge, no existing model addresses this broader prediction task. For benchmarking, we compared our approach with a model that predicts only toxin proteins. To ensure a fair comparison, we limited the test dataset to toxin proteins for this evaluation. ToxinPred2 is widely used and designed for protein sequences, whereas ToxinPred3 is for peptide sequences; therefore, following the ToxinPred developers' recommendation, we used ToxinPred 2.0. In addition, we also used the same test dataset to benchmark the CSM-toxin.

As described in the *Toxin-Associated Prediction Updates*, our dataset intentionally includes both effector toxin proteins and toxin-associated proteins, such as antitoxins, regulatory factors, and accessory proteins (lines 192-203):

> "It is essential to note that this dataset encompasses both effector toxin proteins and their associated proteins, including antitoxins, regulators, and accessory proteins. This offers three main benefits: 1) Recent reports suggest that the same bacterial toxins can function as part of self-inhibiting toxin-antitoxin modules within one organism, while in another organism, they have evolved into toxin effectors that are injected into target cells [39,40]. 2) In bacteria, genes located in close proximity frequently exhibit functional associations, such as those co-transcribed within operons. A comprehensive toxin dataset, including both toxins and their associated proteins, facilitates the identification of novel toxins and related genes through their genomic context, referred to as "toxin islands." These islands may be involved in toxin biosynthesis, processing, or secretion, and may also confer immunity or facilitate horizontal gene transfer among bacterial populations. Notably, they are often rich in mobile

> genetic elements [41]. 3) A large database improves the performance of machine learning classification methods [42]".

Contig-level input

For contig-based analyses, the available tool landscape is much more limited. MetaVF and PathoFact1.0 are, to our knowledge, the only tools that directly support the prediction from assembled contigs. As explained in the manuscript (lines 394-396):

> "To our knowledge, no other method is available to predict VFs from contig sequences and identify plasmid-encoded or prophage-associated VFs".

MetaVF relies primarily on similarity-based approaches, whereas PathoFact1.0 is indeed the previous version of our pipeline. The comparison at the contig level, therefore, serves two purposes: (i) to benchmark against the only available similarity-based method, and (ii) to demonstrate the improvements introduced in the updated PathoFact 2.0 framework. As explained in lines 400-402, MetaVF cannot detect VF in known pathogens, whereas PathoFact 2 can, demonstrating the limitations of the homology-based approach and the advantages of machine-learning-based models and approaches.

Following the reviewer's comment and to include more tools for comparison, we predicted protein sequences from the contigs and used them as input to VirulentHunter, Toxinpred2, and CSM-toxin, and included these results in Figure 7 and the new Figure 8. The following lines, 394-407, describe the performance of VirulentHunter:

> "To our knowledge, no other method is available to predict VF from contig sequences and identify plasmid-encoded or prophage-associated VF. However, we included VirulentHunter in the comparison. Because VirulentHunter does not accept contig sequences as input, protein-coding genes were first predicted from contigs using Pyrodigal-gv and the resulting protein sequences were subsequently analysed. PathoFact 2.0 consistently detected a greater number of VFs than both PathoFact 1.0 and MetaVF (Figure 7, Supplementary Figures S2–S3). Notably, MetaVF failed to identify any VFs in five of the ten pathogenic reference strains tested, highlighting its limited ability to detect VFs and demonstrating the advantages of machine-learning-based models over homology-based approaches. While VirulentHunter produced substantially more hits than metaVF, yielding predictions comparable in number to PathoFact 2.0, PathoFact 2.0 generally identified more VFs overall (Figure 7). Exceptions were observed for non-pathogenic strains *Bifidobacterium animalis*, *Bifidobacterium bifidum*, *Heyndrickxia coagulans*, and for the pathogenic *Ralstonia mannitolilytica strain Guangzhou-RMAB10*, where VirulentHunter predicted slightly more VFs."

and lines 414-423, describing the performance of CSM-Toxin and Toxinpred2, were included in the manuscript:

> "In addition, the PathoFact 2.0 toxin-associated module was compared to PathoFact 1.0 (Figure 8, Supplementary Figures S4–S5) as well as CSM-Toxin and ToxinPred2 (Figure 8). As

> CSM-Toxin and ToxinPred2 do not accept contig sequences as input, protein sequences were first predicted from contigs using Pyrodigal-gv. PathoFact 2.0 predicted more toxin-associated proteins than PathoFact 1.0, demonstrating improved detection capacity. Compared to external tools, CSM-Toxin identified substantially fewer toxins across the tested reference strains. In contrast, ToxinPred2 predicted a comparable number of toxins overall. However, for non-pathogenic strains *Bifidobacterium bifidum*, *Heyndrickxia coagulans*, *Lactobacillus acidophilus*, and for pathogenic strains *Streptococcus gallolyticus subsp. gallolyticus* and Streptococcus parasuis B26, ToxinPred2 identified more predicted toxins than PathoFact 2.0."

The conclusions regarding the performance of the machine-learning models are therefore based on comparisons with contemporary ML-based approaches (VirulentHunter, ToxinPred2, CSM-Toxin) and MetaVF, not solely based on comparisons with the results of PathoFact.

16. The creation of the dataset, for training and most importantly for testing, is rather unclear and described all over the article. I recommend creating a section for it, to understand better the filtering, maybe a figure (could go in the supplementary material, if necessary), and include the amount of data in each test set. The authors seem to have put a lot of effort on the testing sets (including trying to avoid testing with the same data that the models are trained with) but it gets diluted in the article and, in consequence, the test results are difficult to evaluate.

R/We thank the reviewer for the helpful comment. To address concerns about clarity regarding dataset creation, we have now added a new section, Pipeline Updates, and two new figures, Figures 2 and 3, lines 148-151:

> "We implemented thorough updates across all PathoFact modules. Notably, we developed two new machine-learning models: one to predict virulence factors and another to identify toxin-associated proteins. A schematic diagram of the dataset construction is shown in Figure 2. In the sections below, we detail the updates to each module."

and 266-268 in the manuscript:

> "Figure 3 provides a schematic overview of the benchmarking datasets used for the VF and toxin-associated modules."

Figure 2 illustrates the construction of the toxin-associated, virulence factor, and non-toxin/non-VF datasets, and Figure 3 shows the generation of the different test subset datasets. These figures provide a clear overview of the filtering steps and subset creation, ensuring transparency in how the training and test data were defined and the benchmarking was performed.

In addition, we have included comprehensive performance data for the PathoFact 2.0 toxin-associated and virulence factor prediction modules in the Supplementary Tables. Supplementary Table S3 reports evaluation of the toxin-associated module, including class distributions (Negative, non-toxin; Positive, toxin-associated), confusion matrix counts alongside performance metrics—accuracy, precision, recall, F1 score, and Matthews correlation

coefficient—across test subsets stratified by sequence similarity to the training set. Supplementary Table S4 provides corresponding data for the virulence factor module. To facilitate benchmarking against other tools, Supplementary Table S5 summarises virulence factor prediction performance for PathoFact 2.0 at various predicted probability cutoffs compared with VirulentHunter. At the same time, Supplementary Table S6 presents the performance of PathoFact 2.0 for predicting toxin proteins at different cutoffs, alongside that of ToxinPred2.

Together, these additions clarify the dataset construction, provide detailed information on the test sets, and present all relevant performance results, addressing the reviewer's concern regarding the evaluation of test results.

17. The results against VirulentHunter are impressive, outperforming a fine-tuned language model. While I do not doubt that the authors are thorough in their methods, such claims require more testing. Testing using external databases (not created by the authors, maybe the same used by VirulentHunter or other models validated experimentally such as pLM4VF) would support such claims.

R/ We thank the reviewer for the comment.

VirulentHunter is a sophisticated framework that not only predicts whether a protein is a virulence factor but also assigns it to specific functional categories. To our knowledge, this is the only tool that performs both tasks and is based on deep learning. Furthermore, as reported by the VirulentHunter authors, VirulentHunter outperformed other virulence factor predictors (MP4 [49], VirulentPred 2.0 [50], and DeepVF [51]).

In addition, several other VF prediction tools either do not provide their training and test datasets or are no longer actively maintained (e.g., VF-Pred https://pubmed.ncbi.nlm.nih.gov/37979206/, DeepVF http://deepvf.erc.monash.edu/), limiting their suitability for benchmarking.

For these reasons, we selected VirulentHunter for benchmarking. However, this multi-class prediction is trained on a relatively small dataset, increasing the risk of overfitting. This behaviour is evident in Figure 5B, where VirulentHunter's performance decreases substantially as sequence similarity to the training set drops from 100% to 80%, indicating reduced generalisation to more divergent sequences.

We realised that the positive (virulence factor) datasets used by VirulentHunter and PathoFact 2.0 are highly similar and derived from the same sources. Using these datasets without removing similar sequences for external validation would therefore not provide an independent benchmark. Moreover, our negative dataset (i.e., "non-VF") includes proteins from known non-human pathogenic organisms (bacteria and viruses). We further curated the dataset (i.e., "non-VF") by removing any proteins predicted as virulence factors or toxins by PathoFact1. This approach reduces the likelihood of false negatives and broadens taxonomic coverage, including bacterial and viral proteins.

To ensure a fair and reliable comparison, we developed independent subset-based test datasets in which any sequence present in the VirulentHunter training set (100% identity, ≥80% coverage) was

removed. This ensures that neither model was trained on sequences that appear in the test set. In addition, by stratifying test sets by decreasing levels of sequence similarity to the PathoFact 2.0 training data, we were able to explicitly assess model performance across increasingly divergent proteins. This study demonstrates that PathoFact 2.0 maintains consistent performance even when test sequences share no more than 40% similarity with the training set.

Regarding pLM4VF, we note that the publicly available experimentally validated test datasets for Gram-positive and Gram-negative bacteria contain only 922 sequences in total (461 positives and 461 controls). After removing sequences with greater than 99% similarity and coverage of 80% relative to the PathoFact 2.0 and VirulentHunter training datasets, only 73 VF sequences and 452 non-VF sequences remain.

We examined additional test datasets from VirulentPred2 (https://bioinfo.icgeb.res.in/virulent2/down.html) and the MSA-VF predictor (https://github.com/kimtaegyuu/MSA-VFpredictor/tree/main/data). After removing sequences with over 99% similarity and at least 80% coverage relative to the training datasets of PathoFact 2.0 and VirulentHunter, only 10 VF sequences and 561 non-VF sequences remained in VirulentPred2.0, while the MSA-VF predictor dataset retained 48 VF sequences and 561 non-VF sequences.

Although these datasets are valuable, their limited size prevents the subset-based similarity stratification needed for a robustness analysis comparable to the one reported, which utilises between 336 and 4,171 VF sequences and 3,904 to 8,106 non-VF sequences across various subset test datasets.

We believe that our evaluation strategy, based on independent, curated test datasets and explicit control of sequence similarity, provides a rigorous and conservative assessment of model generalisation. To account for this and the reviewer's concern, we have modified the paragraph to clarify this (lines 314-323):

> "Since VirulentHunter and Pathofact 2.0 employ a similar method to generate the "VF dataset" for model training, we removed sequences from the Pathofact 2.0 test dataset that have 100% identity (≥80% coverage) to the VirulentHunter training dataset, resulting in a "clean VF test dataset" (Figure 3). This ensures that neither model used the test sequences for training. We applied the same test-subset approach described earlier: the subset datasets were created based on sequence similarity to the Pathofact 2.0 training dataset, with similarity ranging from 40% to 100% and 80% coverage of the "clean VF test dataset" (Figure 3). By stratifying test sets by decreasing sequence similarity to the training data, we explicitly evaluated model performance across progressively more divergent proteins. This study demonstrates that PathoFact 2.0 maintains consistent performance even when test sequences share no more than 40% similarity with the training set (Figure 5B)".

Lines 343 -345:

> "As shown in Figure 5 (and in the Supplementary Tables S5 and S6), Pathofact 2.0 VF and toxin-associated modules exhibited higher MCC values across different test subsets compared to VirulentHunter, ToxinPred2 and CSM-toxin."

18. The comparative on different bacterial strains gives more questions than answers. Are all those VF and toxins found on E. coli experimentally validated? How much overlap is there between pathogenic and non-pathogenic E. coli? Are all of the same type?

R/ We thank the reviewer for the comment. However, we think that a full biological interpretation of virulence factors (VFs) across diverse *E. coli* strains would constitute an independent study. The aim of our analysis is therefore not to exhaustively characterise virulence in *E. coli*, but to use this well-studied species as a representative example to illustrate the complexity of VF prediction and interpretation.

Not all virulence factors and toxins identified in our analysis have been experimentally validated in *E. coli*. This limitation is inherent to current VF databases, which typically include both experimentally confirmed factors and homologs inferred from sequence similarity. It is known that non-pathogenic *E. coli* strains can harbour genes annotated as virulence factors without causing disease, indicating that the presence of VF genes alone is insufficient to infer pathogenicity.

There is substantial overlap in predicted VF repertoires between pathogenic and non-pathogenic *E. coli*, and the strains analysed do not belong to a single pathotype. This overlap is well documented and reflects that many virulence-associated genes are variably distributed, are frequently located on mobile genetic elements (MGEs), and require specific genomic and regulatory contexts to contribute to disease.

This complexity is precisely one of the motivations for our study. As shown in Figure 6, pathogenic *E. coli* strains are enriched for virulence and toxin-associated proteins associated with MGEs, including plasmids and prophages, compared to non-pathogenic strains. These observations emphasise that distinguishing VFs relevant to infection requires more than standalone VF prediction.

PathoFact 2.0 addresses this challenge by integrating multiple layers of contextual information, including MGE association, secretion signals, and biosynthetic gene cluster localisation. This allows users to move beyond binary VF presence/absence predictions toward a more biologically meaningful interpretation of virulence potential.

Our results highlight that the relationship between predicted virulence factors and disease is more complex than often assumed, and that integrated, comparative approaches are essential for robust interpretation.

The following lines were added to the manuscript to highlight this aspect (lines 365-371):

> “Figure 6 shows distinct differences in virulence and toxin-related profiles between pathogenic and non-pathogenic *E. coli* strains, especially regarding virulence- and toxin-associated proteins encoded on MGEs, such as plasmids and prophages. Nonetheless, analysis of individual VF predictions reveals considerable overlap in the number of VF genes detected across both pathogenic and non-pathogenic strains. This highlights that the presence of a VF gene is not a reliable marker of pathogenicity and emphasises the importance of considering genomic and functional context when assessing virulence potential”.

19. Overall, there is a good amount of work on this project, but the article still has a lot of unanswered questions. It is a bit unclear the strengths of PathoFact2, as well as its weaknesses (any model has). Could be its speed, could be having plenty of tools contained in a pipeline.

R/ We thank the reviewer for the comment.

PathoFact 2.0 represents a clear advance in metagenomic analysis by integrating the detection of antimicrobial resistance genes (ARGs), virulence factors (VFs), toxin-associated proteins, signal peptides, mobile genetic elements (MGEs), and biosynthetic gene clusters (BGCs) within a single, streamlined pipeline. Unlike many existing tools that focus on individual aspects of pathogenicity, PathoFact 2.0 provides a comprehensive, multi-layered view that captures both gene presence and genomic context, improving interpretability and enabling a holistic assessment of microbial pathogenic potential. The pipeline's strengths, including its integrated design and computational efficiency, are illustrated in Figures 1, 4, 5 and Table 1.

We acknowledge several inherent limitations (lines 452-473). Non-pathogenic bacterial strains can also encode genes annotated as VFs or toxins, which makes PathoFact 2.0 most useful as an initial screening tool to identify candidates for further comparative analyses. While the pipeline provides probability scores for VF- or toxin-associated proteins, establishing a definitive link to pathogenicity requires experimental validation. PathoFact 2.0 does not directly classify specific VF or toxin types (e.g., adhesins or genotoxins), but it provides detailed annotations of conserved protein domains (CDD) to facilitate functional inference. Additionally, the pipeline is designed primarily for bacteria-enriched metagenomic data, with predictions interpreted particularly in the context of human pathogens.

Despite these expected limitations, PathoFact 2.0's integrated approach, speed, and multi-layered contextual analysis make it a versatile and powerful tool for studying microbial virulence and resistance in complex microbial communities. The following lines were added to the manuscript (lines 487-492):

> "PathoFact 2.0 represents a major advance in metagenomic analysis by integrating the detection of ARGs, VFs, toxins and toxin-associated proteins, signal peptides, MGEs, and BGCs within a single, streamlined pipeline. Unlike existing tools that focus on individual aspects of pathogenicity, PathoFact 2.0 provides a comprehensive, multi-layered view that captures both gene presence and genomic context, improving interpretability and enabling a holistic assessment of microbial pathogenic potential".

20. I would also appreciate a better description of the report that PathoFact 2.0 produces. If its strength is the virulence and toxin prediction, more tests must be performed (as described above). This would be very beneficial for possible users of the model.

R/ We thank the reviewer for highlighting the need for a clearer description of the reports produced by PathoFact 2.0 and their utility for users. In response, we have added a dedicated paragraph to the manuscript (Lines 430–451; *PathoFact 2.0 Output Structure*):

“*PathoFact 2.0 Output Structure*

“PathoFact 2.0 creates a structured output directory that summarises predictions from all analysis modules, including VFs, toxin-associated proteins, ARGs, MGEs and BGCs. Each module generates dedicated result files corresponding to the underlying prediction tools (Supplementary File S1).

The primary summary file, combined_report.tsv, provides an integrated overview of high-confidence predictions across all modules in a tabular format. This table includes key information such as protein identifiers, bit scores (from HMM profiles), machine-learning prediction scores, and outputs from DeepARG, RGI, SignalP, GenoMad, and antiSMASH, thereby supporting downstream interpretation and candidate prioritisation.

Proteins with prediction probabilities below user-defined thresholds but containing conserved domains identified by toxin-associated or VF HMM profiles are reported in ambiguous_TOX_hits_rep_prot.tsv and ambiguous_VF_hits_rep_prot.tsv. These lower-confidence candidates may warrant further investigation in comparative or experimental analyses.

High-confidence predictions are reported in amr_hits_rep_prot.tsv, TOX_hits_rep_prot.tsv, and VF_hits_rep_prot.tsv, which summarise features exceeding user-defined probability thresholds and include protein identifiers, bit scores, machine-learning predictions, signal peptide predictions, and genomic context information, such as association with prophages or plasmids identified by GenoMad.

In addition, PathoFact 2.0 generates a dedicated Group_of_sequence directory containing FASTA files of representative protein sequences grouped by functional category (VFs, toxin-associated proteins, antimicrobial resistance genes, and combined hits), together with conserved domain (CDD) annotation tables for predicted VFs and toxin-associated proteins. These files are designed to facilitate downstream analyses, including comparative genomics and functional characterisation.”

This paragraph clearly describes the organisation and content of the PathoFact 2.0 output directory. We have also included a supplementary figure that illustrates the output directory structure (Supplementary File S1). This section explicitly details the key output files generated for virulence factor, toxin, and antimicrobial resistance predictions. It describes the integrated combined_report.tsv and explains the separation of high-confidence and ambiguous predictions based on user-defined probability thresholds.

We further clarify that PathoFact 2.0 generates a dedicated Group_of_sequence directory. This directory contains FASTA files of representative protein sequences grouped by functional category: virulence factors, toxins, and antimicrobial resistance genes. It also includes conserved domain (CDD) annotation tables for predicted virulence factors and toxins. As described in the revised manuscript, the pipeline provides high-confidence prediction tables and lower-confidence candidate lists that are supported by conserved-domain evidence. It also offers integrated contextual information, including secretion signals and associations with mobile genetic elements.

This structured reporting enables efficient interpretation of results, candidate prioritisation, and downstream analyses, including comparative genomics and functional characterisation. Together with the output directory schema provided in Supplementary File S1, these additions clearly demonstrate the strengths of PathoFact 2.0's reporting and substantially enhance its usability for the community. It thus represents a strength of PathoFact 2.0 in addition to the strengths highlighted in our response to the comment 19.

21. Moreover, in a more technical note, I recommend the authors to add a test sample for easy testing of the model in their repository.

R/ We thank the reviewer for the comment. As specified in the Data Availability section of the original and revised manuscripts, the datasets for training and testing the VF and Toxin modules are available on Zenodo (https://zenodo.org/records/17593961) because they are too large to store on GitLab. Additionally, in our GitLab repository, we have included a sample test to facilitate easy pipeline testing (to confirm that the pipeline works).

Reviewer #3:

R/ We thank Reviewer 3 for their careful review and constructive criticisms. Their comments have helped us improve the methodological clarity and robustness of the manuscript.

22. *Several methods are available to predict ARGs, VFs, Toxins, and Biosynthetic Gene Clusters. However, the authors selected only a few tools to benchmark PathoFact 2.0. I find this point lacking in the manuscript. To be useful to the scientific community, a more rigorous performance evaluation is needed.

R/ We thank the reviewer for this comment. PathoFact 2.0 is an integrated pipeline, rather than a single prediction model. In this update, we introduce two new prediction modules for virulence factors and toxin-associated proteins, alongside existing modules for antimicrobial resistance genes (ARGs), biosynthetic gene clusters, signal peptides, and functional annotation. The pipeline supports both protein and contig-level inputs.

We think benchmarking against all available tools is not feasible, as it would require evaluating multiple tools for each functional category and input type, resulting in an impractical number of comparisons. In addition, few tools support contig-level input, and several do not provide accessible training or test datasets, limiting the ability to conduct fair and unbiased benchmarking. We therefore selected representative, widely used and state-of-the-art tools for evaluation, which are VirulentHunter and metaVF for VFs and ToxinPred2 for toxins. Alongside Toxinpred2, we have now included a new tool for benchmarking toxin prediction, CSM-toxin.

PathoFact 2.0 further extends existing approaches by predicting toxin-associated proteins, rather than toxin proteins alone, consistent with current strategies for virulence factor prediction (e.g, regulatory proteins are also included). The principal contribution of PathoFact 2.0 is its integrative design, enabling simultaneous prediction of ARGs, virulence factors, toxin-associated proteins, biosynthetic gene clusters, and signal peptides, with results summarised in coherent output tables. This integration supports comprehensive pathogenicity profiling of genomic and metagenomic data.

In addition, as written in the manuscript (lines 104-109):

> "PathoFact, a pipeline first introduced in 2020, integrates ARG, VF, and bacterial toxin prediction from metagenomic data into a single tool [22]. Since the publication of PathoFact, several tools have been implemented to predict ARGs, VFs, and bacterial toxins [23–25]. Only one tool, HyperVR [24], has attempted to predict them simultaneously. However, HyperVR's repository is no longer available online, and the Zenodo archive from its original submission lacks the necessary databases, hence rendering it unusable."

Specifically, for VFs and Toxins, PathoFact 2.0 supports two distinct types of input, and our benchmarking strategy reflects this distinction:

1. Protein sequence input

For protein-level predictions, we benchmarked the machine-learning models implemented in PathoFact 2.0 against state-of-the-art tools: VirulentHunter (virulence factors), ToxinPred2.0 (toxins) and CSM-Toxin (toxins).

Furthermore, our evaluation strategy goes beyond a simple tool comparison. We constructed independent, curated test datasets in which any sequence present in the VirulentHunter training set (100% identity, ≥80% coverage) was removed, ensuring that no test sequence was seen during model training. By stratifying test sets according to decreasing sequence similarity to the PathoFact 2.0 training data, we explicitly assessed performance across increasingly divergent proteins. Remarkably, PathoFact 2.0 maintains consistent performance even when test sequences share ≤40% similarity with the training set.

This methodology provides a rigorous, conservative, and reproducible assessment of model generalisation.

2. Contig-level input

For contig-based analyses, the available tool landscape is much more limited. To our knowledge, MetaVF and PathoFact are the only tools capable of predicting directly from assembled contigs. MetaVF relies primarily on similarity-based approaches, whereas PathoFact represents the previous version of our pipeline. Benchmarking at the contig level thus serves two purposes: (i) to compare against the only available similarity-based method, and (ii) to demonstrate the improvements introduced in PathoFact 2.0. As noted in lines 400–402, MetaVF fails to detect VFs in certain known pathogens, whereas PathoFact 2.0 succeeds, highlighting the limitations of homology-based methods and the advantages of our machine-learning approach.

We benchmarked PathoFact 2.0 to PathoFact (its predecessor), ToxinPred2, CSM-Toxin, VirulentHunter, and metaVF. We believe this evaluation approach yields results that are reliable, scientifically significant, and practically applicable to the community.

23. *It is not fully clear how the "false" sequences were chosen. Ideally, they should be similar to known resistance genes, but should not confer resistance.
R/ We thank the reviewer for the comment. We would like to clarify that PathoFact 2.0 does not benchmark ARG prediction tools. ARG prediction is carried out using three widely used, established tools.

24. *Details of the parameters used to create the HMM models are not mentioned in the manuscript. The performance of the updated HMMs in comparison to the older version is not shown.

R/ We thank the reviewer for the comment. The creation of the HMMs was introduced in lines 205-206 of the manuscript. We have also added a new section in Methods, HMM profiles, lines 551-558:

> "Profile hidden Markov models are probabilistic models built from a multiple sequence alignment that encode, for each alignment position, the position-specific probabilities of residues and insertions/deletions, turning the alignment into a position-specific scoring system for detecting homologous sequences [67]. The FASTA files of conserved-domain multiple sequence alignments for each CDD [26] family (https://ftp.ncbi.nih.gov/pub/mmdb/cdd/fasta.tar.gz) were downloaded. Pyhmmer v0.10.14 [68] was used to obtain HMM profiles for each CDD family and to perform protein sequence searches against the CDD HMM family profiles."

Unlike PathoFact, PathoFact 2.0 does not use HMMs for predictions; instead, we rely on machine-learning models. The HMMs in PathoFact 2.0 are used solely for functional annotation, and therefore, a direct performance comparison with the previous version is not applicable.

25. *It would be interesting to show how updates in DeepARG, RGI, and AMRFinderPlus have improved the performance of PathoFact 2.0 over version 1.0.

R/ We thank the reviewer for the comment. The performance comparison for updates to DeepARG, RGI, and AMRFinderPlus is presented in Supplementary Figures S4 and S5. We have added the following lines to the manuscript in the section *ARG Prediction Updates* (lines 253-255):

> "Supplementary Figures S4 and S5 compare the performance of PathoFact 2.0 with that of its predecessor, PathoFact, in identifying ARGs."

26. *I believe the non-pathogenic dataset was constructed using sequences other than those mentioned in the section "Generalities about Machine learning training set-up and 'non-pathogenic". This means that sequences that do not contain the mentioned keyword were used as the negative dataset. These sequences include housekeeping genes, which are also too distant from the ARG, VF, etc. The real test of an ML model occurs with data from the grey zone, which has properties of both negative and positive examples. The authors can benchmark the ML model using the grey-zone data to show the efficiency of the ML model.

R/ We thank the reviewer for the comment.

We constructed the negative dataset to approximate a biologically realistic non-pathogenic background by including complete protein sets from organisms reported as non-pathogenic to humans.

In addition, we selected SwissProt sequences lacking ARG, VF, and toxin keywords [KW-0568 (pathogenesis-related protein), KW-0843 (virulence), KW-0800 (toxin), KW-0046 (antibiotic resistance), KW-9995 (disease)] and limited to bacteria (taxonomy_id 2), archaea (taxonomy_id 2157), fungi (taxonomy_id 4751), and viruses (taxonomy_id 10239). To reduce the inclusion of proteins with potential virulence-associated functions that are not captured by keyword-based annotation alone, we further filtered the dataset by removing proteins predicted as virulence factors or toxins by PathoFact 1.0.

Notably, the inclusion of housekeeping genes from non-pathogenic microorganisms can be considered a feature rather than a limitation, as several well-characterised housekeeping proteins in pathogenic bacteria have been shown to perform virulence-associated functions ("moonlighting" proteins), including adhesion, immune modulation, and tissue invasion[1,2]. Representative examples include glyceraldehyde-3-phosphate dehydrogenase (VF0015 in VFDB)[3], enolase [1,2], elongation factor Tu (VF0460 in VFDB) [4,5], GroEL [1,6,7], and DnaK [1,8]. This approach therefore maintains a realistic negative dataset while minimising false negatives within PathoFact's operational scope.

1. Henderson B, Martin A. Bacterial virulence in the moonlight: multitasking bacterial moonlighting proteins are virulence determinants in infectious disease. *Infect Immun*. American Society for Microbiology; 79:3476–912011;

2. Henderson B, Martin A. Bacterial moonlighting proteins and bacterial virulence. *Curr Top Microbiol Immunol*. Curr Top Microbiol Immunol; 358:155–2132013;

3. Pancholi V, Fischetti VA. A major surface protein on group A streptococci is a glyceraldehyde-3-phosphate-dehydrogenase with multiple binding activity. *J Exp Med*. Rockefeller University Press; 176:415–261992;

4. Barel M, Charbit A. Detection of the interaction between host and bacterial proteins: eukaryotic nucleolin interacts with Francisella elongation factor Tu. *Methods Mol Biol*. Methods Mol Biol; 1197:123–392014;

5. Granato D, Bergonzelli GE, Pridmore RD, Marvin L, Rouvet M, Corthésy-Theulaz IE. Cell surface-associated elongation factor Tu mediates the attachment of Lactobacillus johnsonii NCC533 (La1) to human intestinal cells and mucins. *Infection and immunity*. Infect Immun; 2004; doi: 10.1128/iai.72.4.2160-2169.2004.

6. Kamiya S, Yamaguchi H, Osaki T, Taguchi H. A virulence factor of Helicobacter pylori: role of heat shock protein in mucosal inflammation after H. pylori infection. *J Clin Gastroenterol*. 27 Suppl 1:S35–91998;

7. Hickey TBM, Ziltener HJ, Speert DP, Stokes RW. Mycobacterium tuberculosis employs Cpn60.2 as an adhesin that binds CD43 on the macrophage surface: M. tuberculosis Cpn60.2 mediates macrophage binding via CD43. *Cell Microbiol*. Hindawi Limited; 12:1634–472010;

8. Lehner T, Bergmeier LA, Wang Y, Tao L, Sing M, Spallek R, et al.. Heat shock proteins generate β-chemokines which function as innate adjuvants enhancing adaptive immunity. *Eur J Immunol*. 30:594–6032000;

The following text was added to the revised manuscript (lines 463-470):

“The inclusion of housekeeping genes from non-pathogenic microorganisms reflects a deliberate methodological choice rather than a limitation. Multiple well-characterised housekeeping proteins have been shown to exhibit virulence-associated “moonlighting” functions in pathogenic bacteria, including roles in adhesion, immune modulation, and tissue invasion [56,57]. Notable examples include glyceraldehyde-3-phosphate dehydrogenase (VF0015 in VFDB) [58], enolase [59,60], elongation factor Tu (VF0460 in VFDB) [61,62], GroEL [60,63,64], and DnaK [60,65]. This approach maintains a biologically realistic negative dataset while reducing the risk of misclassifications within the intended scope of PathoFact 2.0.”

PathoFact 2.0: An Integrative Pipeline for the Prediction of Antimicrobial Resistance Genes, Virulence Factors, Toxins and Toxin-associated Proteins, and Biosynthetic Gene Clusters in Metagenomes

Luis F. Delgado (luis.delgado@uni.lu)*[1][0000-0001-7850-5285], Júlia Ortís Sunyer (julia.ortissunyer@uni.lu)*[1][0000-0002-2714-7067], Cedric C. Laczny (cedric.laczny@uni.lu)[1][0000-0002-1100-1282], Oskar Hickl (oskar.hickl@lih.lu)[1][0000-0001-9959-8767], Patrick May (patrick.may@uni.lu)[1][0000-0001-8698-3770] & Paul Wilmes (paul.wilmes@uni.lu)[1,2][0000-0002-6478-2924]

1. Luxembourg Centre for Systems Biomedicine, University of Luxembourg, Esch-sur-Alzette, Luxembourg
2. Department of Life Sciences and Medicine, Faculty of Science, Technology and Medicine, University of Luxembourg, Esch-sur-Alzette, Luxembourg

Contact: Paul Wilmes (paul.wilmes@uni.lu)

*These authors contributed equally

// Abstract

Background

Antimicrobial resistance genes (ARG) and virulence factors (VFs) are central contributors to the global health crisis surrounding drug-resistant infections.

Findings

We introduce PathoFact 2.0, an enhanced pipeline for improved ARG, VF, toxin prediction, and biosynthetic gene clusters (BGC). Key improvements include an updated machine learning (ML) model for VF identification, expanded hidden Markov model profiles for VFs and toxin-associated proteins, a new ML model for toxin and toxin-associated proteins identification, and the integration of antiSMASH 7.0 for predicting biosynthetic gene clusters.

Conclusions

Our upgrades make PathoFact 2.0 a more powerful and user-friendly platform for predicting microbiome-based pathogenicity and resistance, providing a crucial tool for better understanding and addressing the challenges posed by antimicrobial resistance and infectious diseases.

PathoFact 2.0 is available at https://gitlab.com/uniluxembourg/lcsb/systems-ecology/pathofact2. It is compatible with Linux operating systems.

Keywords

Antimicrobial resistance genes, virulence factors, toxin-associated proteins, biosynthetic gene clusters, metagenomes, machine learning

Findings

Introduction

Microbiomes are highly complex and diverse ecological communities composed of bacteria, archaea, viruses, and microeukaryotes. These communities include both commensal microorganisms, which can contribute to host health, and pathogenic or opportunistic microorganisms that can cause disease under specific conditions. Microbial communities generally exist in synergistic relationships with their hosts, playing critical roles in maintaining physiological homeostasis and regulating immune function. However, disruption of this balanced microbial ecosystem, known as microbial dysbiosis, can impair normal body functions and has been associated with the development of various diseases, including cardiovascular diseases, cancers, and respiratory disorders [1].

Moreover, these microorganisms play a critical role in the development of antibiotic-resistant infections through the presence of antimicrobial resistance genes (ARGs) and virulence factors (VFs)[2,3]. ARGs are genetic elements that confer bacterial resistance to antibiotics. Many ARGs are encoded on mobile genetic elements (MGEs) and are therefore often horizontally transmitted [4]. ARGs can be divided into categories based on the antibiotics to which they confer resistance [5]. The Antibiotic Resistance Ontology (ARO) contains information on ARGs, the mutations that cause them, their products, mechanisms, associated phenotypes, antibiotics, and molecular targets [6].

Bacterial pathogens use specific genes, known as VFs, to attach to and invade host tissues, survive within the host, spread, and ultimately cause damage. The harm inflicted can vary, ranging from minor disruptions to severe or even fatal outcomes [7]. VFs can be classified as secretory, membrane-associated, or cytosolic. Cytosolic virulence factors promote rapid adaptive shifts in bacterial metabolism, physiology, and morphology, enhancing survival and proliferation within the host. Membrane-associated factors contribute to bacterial adhesion and immune evasion at the host-cell interface. Secretory factors constitute a critical part of the bacterial armamentarium, enabling bacteria to counteract innate and adaptive immune defences. Secretory VFs often exhibit synergistic effects and induce cytotoxicity in host cells [8]. VFs are often located on MGEs, such as transposons, plasmids and phages, facilitating their transfer between bacterial cells [9,10].

Bacterial toxins play a crucial role in the development of infectious diseases, alongside various virulence factors employed by pathogens. They disrupt host processes and manipulate immune responses. Some toxins impair protein synthesis, destroy blood cells, or affect the nervous system. Bacterial toxins can be divided into two main categories: cell-associated endotoxins and extracellular, diffusible exotoxins. Endotoxins, such as lipopolysaccharides, are found in the outer membranes of Gram-negative bacteria and serve as potent inflammatory mediators that can induce systemic toxicity and septic shock in infected hosts [11]. Exotoxins, which are typically polypeptides and proteins, can stimulate a range of host responses by either acting directly on cell receptors or through enzymatic modulation [12,13]. Many bacterial toxins are secreted proteins that require signal peptides. Signal peptides are short amino acid sequences at the N-terminus of proteins that direct them to specific cellular compartments, such as the periplasm [14,15].

Biosynthetic gene clusters (BGCs) are responsible for synthesising specialised metabolites (SMs). Some SMs can increase pathogenicity; for example, clinical isolates of *Pseudomonas aeruginosa* produce siderophores, rhamnolipids, quinolones, and phenazines [16]. Similarly, *Burkholderia* strains produce virulence factors, such as toxoflavin from *Burkholderia glumae* [17]. Notably, pyocyanin, a redox-active phenazine produced by *Pseudomonas aeruginosa*, plays a crucial role as a virulence factor in lung infections [18].

The threat that ARGs, VFs and toxins pose to human health is significant. The United Nations have identified antimicrobial resistance as a global threat, with estimates attributing 1.27 million deaths annually to drug-resistant infections, potentially rising to 10 million by 2050 if unaddressed [19,20]. Thus, accurately predicting potential ARG and VF profiles is essential for early intervention, enabling anticipation of infection severity, improving treatment strategies, and ultimately reducing mortality rates from disease-causing pathogens.

Predicting and annotating ARGs, VFs, and toxins is challenging due to limited well-annotated data [21] and complex mechanisms involving gene transfer, mutations, and multifactorial interactions. Traditional annotation methods, which rely on sequence similarity, may overlook novel ARGs, VFs and toxins. In contrast, machine learning offers robust solutions through pattern recognition, enabling accurate predictions even with limited training data.

An integrated bioinformatics pipeline enhances analysis by simultaneously examining ARGs, VFs, toxins, signal peptides, and BGCs from a single metagenomic sample. This comprehensive approach provides a more complete view of bacterial pathogenicity by capturing the full spectrum of virulence mechanisms, including antimicrobial resistance, toxin production, and secondary metabolic capabilities. This holistic analysis improves insights into pathogenicity and resistance, streamlines workflows, and simplifies data interpretation.

PathoFact, a pipeline first introduced in 2020, integrates ARG, VF, and bacterial toxin prediction from metagenomic data into a single tool [22]. Since the publication of PathoFact, several tools have been implemented to predict ARGs, VFs, and bacterial toxins [23–25]. Only one tool, HyperVR [24], has attempted to predict them simultaneously, analogous to PathoFact. However, HyperVR's repository is no longer available online, and the Zenodo archive from its original submission lacks the necessary databases, hence rendering it unusable.

Here, we present PathoFact 2.0 (Figure 1). It enhances the previous version by supporting protein sequences or contigs as input and by updating the ML VF model and the hidden Markov model (HMM) profiles of the conserved domain databases (CDD) [26] for VF and toxin-associated protein annotation. We have also introduced the ability to predict BGCs using antiSMASH 7.0 [27]. antiSMASH is a tool that identifies, annotates, and analyses secondary metabolite BGCs across genomes.

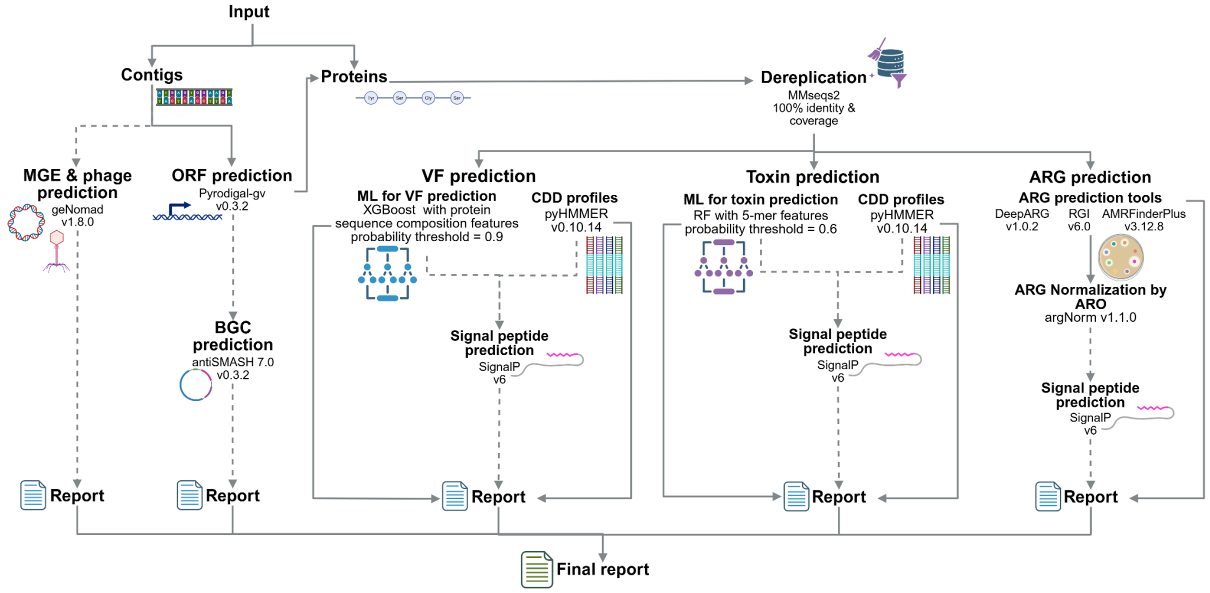

Figure 1. Schematic representation of PathoFact 2.0. Solid lines denote core modules, while dotted lines indicate optional user selection. The input is a FASTA file with either contig or protein sequences.

If the input is a FASTA file containing contigs, open reading frames (ORFs) are predicted using Pyrodigal-gv. If the biosynthetic gene cluster (BGC) option is selected, antiSMASH will use the GBK file for BGC prediction. GeNomad is used for MGE and phage prediction, producing a FASTA file of protein sequences. Protein sequences are dereplicated using MMseqs2 to retain non-redundant sequences (based on 100% identity and coverage). After dereplication, antimicrobial resistance genes (ARGs), virulence factors (VFs) and toxins and their associated proteins are predicted using their respective modules. SignalP predicts the presence of signal peptides and their cleavage sites in proteins from archaea, bacteria and eukarya. Individual reports are generated for each module, and an integrated report is produced that combines all module reports.

Pipeline Structure

Unlike version 1.0, which supports only contigs, PathoFact 2.0 accepts nucleotide sequences of contigs and protein sequence FASTA files, with proteins dereplicated by our tool to retain only non-redundant sequences (based on 100% identity and coverage). For contig-based inputs, open reading frames are predicted using Pyrodigal-gv (version 0.3.2; [28,29]; https://github.com/althonos/pyrodigal-gv), a Python library that binds to Prodigal [22], followed by the detection of MGEs and phages using geNomad (version 1.8.0; [29]). GeNomad processes only nucleotide sequences; therefore, MGEs and phages are not detected in protein sequence inputs. Based on user configuration, the pipeline then analyses the processed sequences using the BGC, ARG, VF, toxin-associated, and BGC (via antiSMASH) prediction modules. The information is compiled into individual module reports and an integrated report, also incorporating details from SignalP and geNomad (Figure 1). Additionally, PathoFact 2.0 generates a FASTA file of proteins identified as ARGs, VFs, or toxin-associated proteins.

Pipeline Installation

PathoFact 2.0 is implemented using Snakemake (version 7.25.0; [30]). An installation script simplifies the setup by installing the required software and downloading databases with a single command. PathoFact 2.0 is open-source (GNU General License v3.0 or later) and freely available at https://gitlab.com/uniluxembourg/lcsb/systems-ecology/pathofact2, where detailed instructions for pipeline installation, configuration, and output are provided.

Pipeline Updates

We implemented thorough updates across all PathoFact modules. Notably, we developed two new machine-learning models: one to predict virulence factors and another to identify toxin-associated proteins. A schematic diagram of the construction of the training and test datasets is shown in Figure 2. In the sections below, we detail the updates to each module.

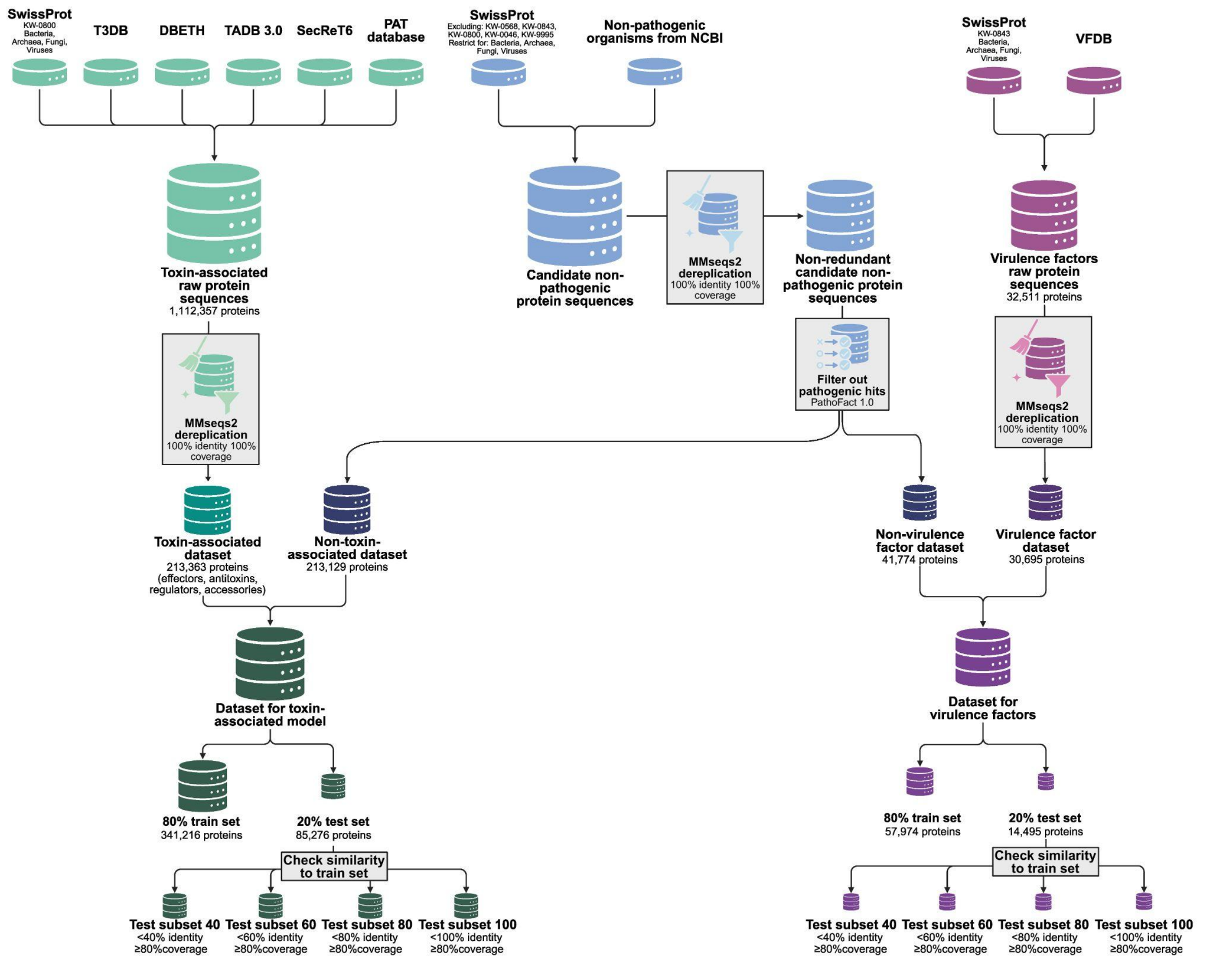

Figure 2. Schematic representation of the datasets used for PathoFact 2.0 toxin-associated and virulence factors modules training and testing. Shades of green represent the toxin-associated datasets; shades of blue represent the non-pathogenic dataset; and shades of purple represent the virulence factor datasets.

Generalities about the "non-pathogenic" dataset and the machine learning training setup

The "non-pathogenic" dataset for the ML models was constructed by selecting SwissProt [31] sequences lacking ARG, VF, and toxin keywords [KW-0568 (pathogenesis-related protein), KW-0843 (virulence), KW-0800 (toxin), KW-0046 (antibiotic resistance), KW-9995 (disease)] and limited to bacteria (taxonomy_id 2), archaea (taxonomy_id 2157), fungi (taxonomy_id 4751), and viruses (taxonomy_id 10239) (Figure 2). Additionally, proteins from non-pathogenic organisms to humans (Supplementary Table S1; Figure 2) were included from NCBI. MMseqs2 (version 15.6f452; [32]) was used to obtain a set of non-redundant (clustered at 100% identity and coverage) protein sequences (Figure 2).

ML models were trained using 80% of the sequences for training and 20% of the sequences for testing. The Synthetic Minority Oversampling Technique (SMOTE) was employed to address the dataset's imbalance [33]. Using the XGBoost Python package (https://xgboost.readthedocs.io/en/stable/index.html) and the RandomForest (RF) Scikit-learn (version 1.5.2; [34]), several ML models were trained and tested using k-mers (k=3 to 8) or protein sequence composition features (amino acid composition (AAC), dipeptide composition (DPC), composition (CTDC), transition (CTDT), and distribution (CTDD) [35]) as features. Hyperparameter

optimisation was performed, using a 5-fold cross-validation with HalvingGridSearchCV from scikit-learn [34]. The best-performing model was selected based on the Matthews correlation coefficient (MCC) score.

Toxin-Associated Protein Prediction Updates

Compared to version 1.0, the toxin prediction module now employs a ML model instead of a purely alignment-based bit score threshold. Curated training data was obtained from SwissProt [31], filtered for bacterial (taxonomy_id 2), archaeal (taxonomy_id 2157), fungal (taxonomy_id 4751) and viral (taxonomy_id 10239) toxin sequences (KW-0800, toxin)(Figure 2). The dataset was supplemented with entries from toxin-specific databases such as the Toxin Exposome Database (T3DB) [12], which catalogues bacterial protein toxins; the Database for Bacterial ExoToxins (DBETH) [13]; TADB version 3.0, which includes protein sequences of bacterial toxin–antitoxin (TA) pairs from types I to VIII [36]; sequences from SecReT6 [37], encompassing T6SS gene cluster components, T6SS regulator (T6SR), accessory proteins (T6SA), effectors (T6SE), and immunity proteins (T6SI); and the prokaryotic antimicrobial toxins (PAT) database [38] (Figure 2).

MMseqs2 (version 15.6f452; [32]) was used to dereplicate the dataset of 1,112,357 protein sequences (100% identity and coverage), yielding 213,363 unique protein sequences, corresponding to the "toxin-associated" dataset (Figure 2). It is essential to note that this dataset encompasses both effector toxin proteins and their associated proteins, including antitoxins, regulators, and accessory proteins. This offers three main benefits: 1) Recent reports suggest that the same bacterial toxins can function as part of self-inhibiting toxin-antitoxin modules within one organism, while in another organism, they have evolved into toxin effectors that are injected into target cells [39,40]. 2) In bacteria, genes located in close proximity frequently exhibit functional associations, such as those co-transcribed within operons. A comprehensive toxin dataset, including both toxins and their associated proteins, facilitates the identification of novel toxins and related genes through their genomic context, referred to as "toxin islands." These islands may be involved in toxin biosynthesis, processing, or secretion, and may also confer immunity or facilitate horizontal gene transfer among bacterial populations. Notably, they are often rich in mobile genetic elements [41]. 3) A large database improves the performance of machine learning classification methods [42].

HMM profiles were built using the conserved-domain FASTA files (https://ftp.ncbi.nih.gov/pub/mmdb/cdd/fasta.tar.gz) from CDD [26]. The 213,363 unique protein sequences in the "toxin-associated" dataset were annotated using the CDD HMM profiles. Those with a bitscore above 25 were chosen as HMM profiles for toxin and toxin-associated protein annotation and incorporated into Pathofact 2.0 for protein annotation.

Although there is no standard for creating negative datasets, they play a crucial role in influencing model performance. Therefore, to improve the quality of our "non-toxin" dataset, potential ARGs, VFs (with high probability), and toxins were filtered out of the "non-pathogenic" dataset using PathoFact 1.0 predictions. The final "non-toxin" dataset consists of 213,129 non-redundant protein sequences (Figure 2).

The toxin-associated ML model is a RF with 5-mer features (default hyperparameter setting, i.e, n_estimators=100, max_depth=None, min_samples_split=2).

The toxin-associated protein prediction module generates a report containing the proteinID, protein domains, bitscore, toxin-associated ML probability, other identical proteins found in the sample, and optionally SignalP, plasmid marker, and virus marker information.

VF Prediction Updates

The VF prediction model was refined and updated with new HMM profiles. Training data was derived from SwissProt [31], selecting sequences annotated with the virulence keyword (KW-0843) and expanded using the Virulence Factor Database (VFDB; [43]) (Figure 2). After dereplication (with 100% identity and coverage), the original set of 32,511 sequences, using MMseqs2, comprised 30,695 non-redundant sequences, corresponding to the “VF dataset” (Figure 2). We searched the “VF dataset” against the CDD HMM profiles, selecting those with a bit score of 25 or higher as VF HMM profiles for PathoFact 2.0. The HMM profile dataset annotates the predicted VF domains rather than using them as input to the classification, as in the previous version.

To create the “non-VF” dataset for the ML VF model, we filtered out any potential VFs (with high and low probabilities), ARGs, and toxins based on PathoFact 1.0 predictions from the “non-pathogenic” dataset. This resulted in a dataset of 41,774 VF protein sequences (Figure 2).

The VF model uses XGBoost with protein sequence composition features (learning_rate': 0.1, 'n_estimators': 2000). The VF module generates a report containing the proteinID, protein domains, bitscore, virulence factor ML probability, other identical proteins found in the sample, and optionally SignalP, plasmid marker, and virus marker information.

ARG Prediction Updates

ARG prediction in PathoFact 2.0 integrates DeepARG (version 1.0.2; [44]), RGI (version 6; [6]), and AMRFinderPlus (version 3.12.8; [45]). DeepARG and RGI have received updates from their developers since the release of PathoFact 1.0, which have been incorporated into PathoFact 2.0. In addition, AMRFinderPlus has been newly integrated into PathoFact 2.0. Each tool has distinct strengths: DeepARG offers high precision and recall; RGI provides robust predictions based on an extensive database, utilising homology and single-nucleotide polymorphism (SNP) models; and AMRFinderPlus efficiently identifies resistance genes and mutations using NCBI resources.

The ARG prediction module (Figure 1) report includes protein IDs, ARG classes, prediction probabilities, database accession numbers, and optional data on signal peptides, plasmids, and virus markers. PathoFact 2.0 uses argNorm [46] to map detected genes to the ARO, thereby facilitating comparison of ARG annotation outputs by ensuring standardised and comparable results. Supplementary Figures S4 and S5 compare the performance of PathoFact 2.0 with that of its predecessor, PathoFact, in identifying ARGs.

Additional Functionalities

PathoFact 2.0 integrates SignalP (version 6; [24]) and antiSMASH (version 7.0; [27]), both of which are optional features that accommodate diverse research needs. SignalP is designed to predict the presence and location of signal peptides in protein sequences. It requires a separate license and must be requested by the user individually. AntiSMASH is designed to identify and annotate BGCs in bacterial and fungal genomes. Since AntiSMASH is a resource-intensive tool, we set it up as an optional module and provide the option to run it in chunks.

Evaluation of the performance of the PathoFact 2.0 pipeline

We evaluated the performance of PathoFact 2.0 and the new VF and toxin-associated modules using the test datasets described above. We did not include ARGs and BGCs in the validation step, as the respective modules are based on existing tools that have already demonstrated high accuracy [6,27,44,45]. Figure

3 provides a schematic overview of the benchmarking datasets used for the VF and toxin-associated modules.

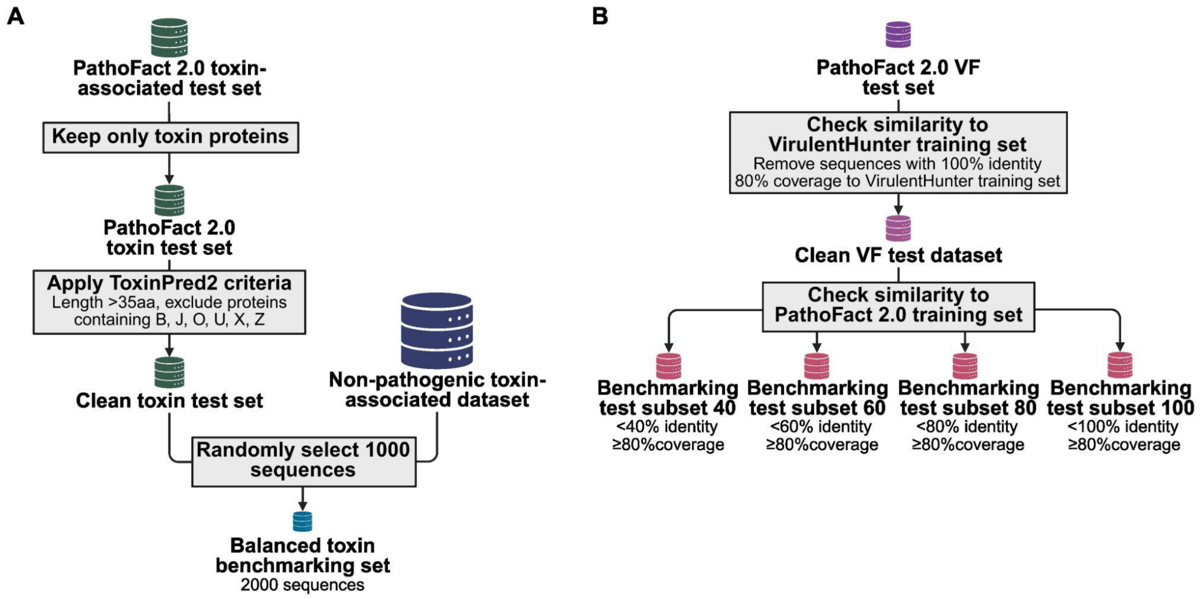

Figure 3. Schematic representation of the construction of the datasets used for benchmarking. A) Toxin benchmarking dataset. B) Virulence factor benchmarking dataset.

Virulence factors and toxin-associated protein prediction

The VF and toxin-associated modules (Figure 1) were evaluated across different ML models-predicted probability thresholds, corresponding to the model's confidence in the positive class (VF or toxin-associated). Analyses were performed on the full test dataset and on subsets of the test dataset. These subset datasets were created based on sequence similarity to the training dataset, with a range of 40% to 100% similarity and 80% coverage (Figure 2). This approach aimed to assess prediction accuracy on proteins in the testing dataset with low similarity to the training dataset, specifically including only sequences with less than 40-100% identity to any training sequence. The performance evaluation is based on the Matthew correlation coefficient (MCC) and the precision (to reduce the number of false positive VF and toxin-associated predictions), taking into account the dataset imbalance (a higher number of “non-toxin” and “non-VF” sequences compared to “toxin-associated” and “VF” sequences in the test subsets). The MCC is a more reliable statistical measure that yields a high score only when the prediction performs well across all four categories of the confusion matrix (true positives, false negatives, true negatives, and false positives), and it is proportional to both the number of positive and negative elements in the dataset [47]. We found that predicted probabilities of 0.6 for toxin-associated proteins and 0.9 for VFs provide a good balance between high MCC and precision across different test subsets (Figure 4 and Supplementary Tables S3 and S4).

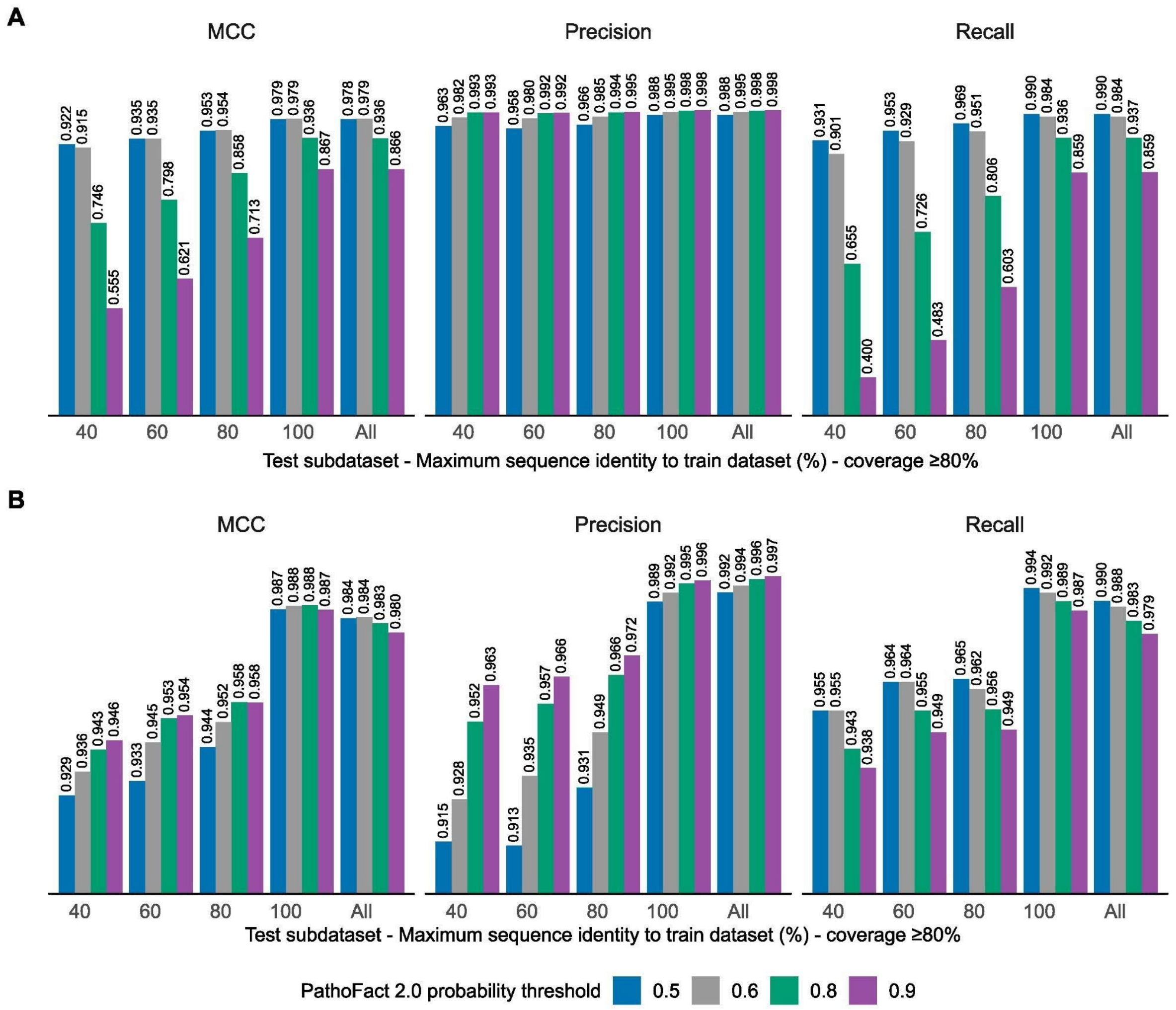

Figure 4. Performance evaluation of toxin-associated and virulence factor prediction modules across probability thresholds. A) Toxin-associated prediction module evaluation. **B)** Virulence factors prediction module evaluation. The modules were evaluated across a range of predicted class probabilities (0.5-0.9). The entire test dataset (All) and subsets of the test datasets were used for evaluation. These subset datasets were created based on sequence similarity to the training dataset, with similarity levels of 40%, 60%, 80%, and 100%, and an 80% coverage threshold. Only sequences with similarity below these percentages were included in the respective test subsets.

Benchmarking

The Pathofact 2.0 VF prediction module was compared to VirulentHunter [48], using the default parameters. VirulentHunter is a deep learning framework that simultaneously identifies and classifies VFs directly from protein sequences, which outperforms other virulence factor predictors (MP4 [49], VirulentPred 2.0 [50], and DeepVF [51]). A notable feature of VirulentHunter is that it provides VF category classification; however, it takes about 2 minutes to analyse 500 protein sequences (using 1 GPU), which is a drawback for metagenomic sample analysis, where thousands to millions of proteins are predicted from a single sample. PathoFact 2.0 requires only 4 seconds (using 1 CPU, with the option to utilise more CPUs) to analyse 500 protein sequences (Table 1).

Table 1. Runtime comparison of PathoFact 2.0 and VirulentHunter

Number of protein sequences	VirulentHunter	PathoFact 2.0				
	1 GPU	1 CPU	2 CPU	4 CPU	6 CPU	8 CPU
500	2 min 21 s	3.7 s	2.6 s	2.0 s	1.9 s	1.8 s
5500	25 min 42 s	29.9 s	17.4 s	10.2 s	8.1 s	7.4 s
10000	54 min 11 s	55.4 s	29.3 s	16.7 s	13.1 s	11.5 s
30000	2 h 45 min 50 s	2 min 45 s	1 min 32.9 s	52.1 s	40.3 s	36.0 s

Since VirulentHunter and Pathofact 2.0 employ a similar method to generate the "VF dataset" for model training, we removed sequences from the Pathofact 2.0 test dataset that have 100% identity (≥80% coverage) to the VirulentHunter training dataset, resulting in a "clean VF test dataset" (Figure 3). This ensures that neither model used the test sequences for training. We applied the same test-subset approach described earlier: the subset datasets were created based on sequence similarity to the Pathofact 2.0 training dataset, with similarity ranging from 40% to 100% and 80% coverage of the "clean VF test dataset" (Figure 3). By stratifying test sets by decreasing sequence similarity to the training data, we explicitly evaluated model performance across progressively more divergent proteins. This study demonstrates that PathoFact 2.0 maintains consistent performance even when test sequences share 40% similarity or less with the training set (Figure 5B).

The Pathofact 2.0 toxin-associated module was compared with ToxinPred2 [42] using the default parameters, i.e., Hybrid (RF+BLAST+MERCI) with a threshold of 0.6. The ToxinPred2 website restricts predictions to a certain number of proteins (around 2000). Since ToxinPred2 is designed to predict protein toxicity, we selected sequences from the Pathofact 2.0 "toxin-associated" test dataset that are directly linked to toxins and removed the remaining "toxin-associated" proteins (Figure 3). In short, using the header information from the Pathofact 2.0 "toxin-associated" test dataset, we kept only toxin sequences from the toxin-antitoxin sequences from the TADB, the effector factor sequence from the SecReT6 database, bacterial protein toxins from T3DB, bacterial exotoxins from DBETH, and sequences from Swissprot (KW-0800, toxin), as previously described. Additionally, we kept sequences longer than 35 amino acids and excluded protein sequences containing the non-standard amino acids 'BJOUXZ' as the ToxinPred2 dataset was created using these criteria [52] (Figure 3). From these, we randomly selected 1000 sequences. Then, we randomly selected 1000 sequences from the Pathofact 2.0 "non-toxin" test dataset (Figure 3). This resulted in a total of 2000 sequences for benchmarking PathoFact 2.0 against the ToxinPred2 webserver. Due to the limited number of sequences, we did not use the test-subset approach to evaluate ToxinPred2 and Pathofact 2.0 toxin-associated modules on this 2000-sequence test dataset (Figure 3). We also use the same test dataset to benchmark CSM-toxin v1.0.1, a deep learning model for toxin prediction [53].

As shown in Figure 5 (and in the Supplementary Tables S5 and S6), Pathofact 2.0 VF and toxin-associated modules exhibited higher MCC values across different test subsets compared to VirulentHunter, ToxinPred2 and CSM-toxin.

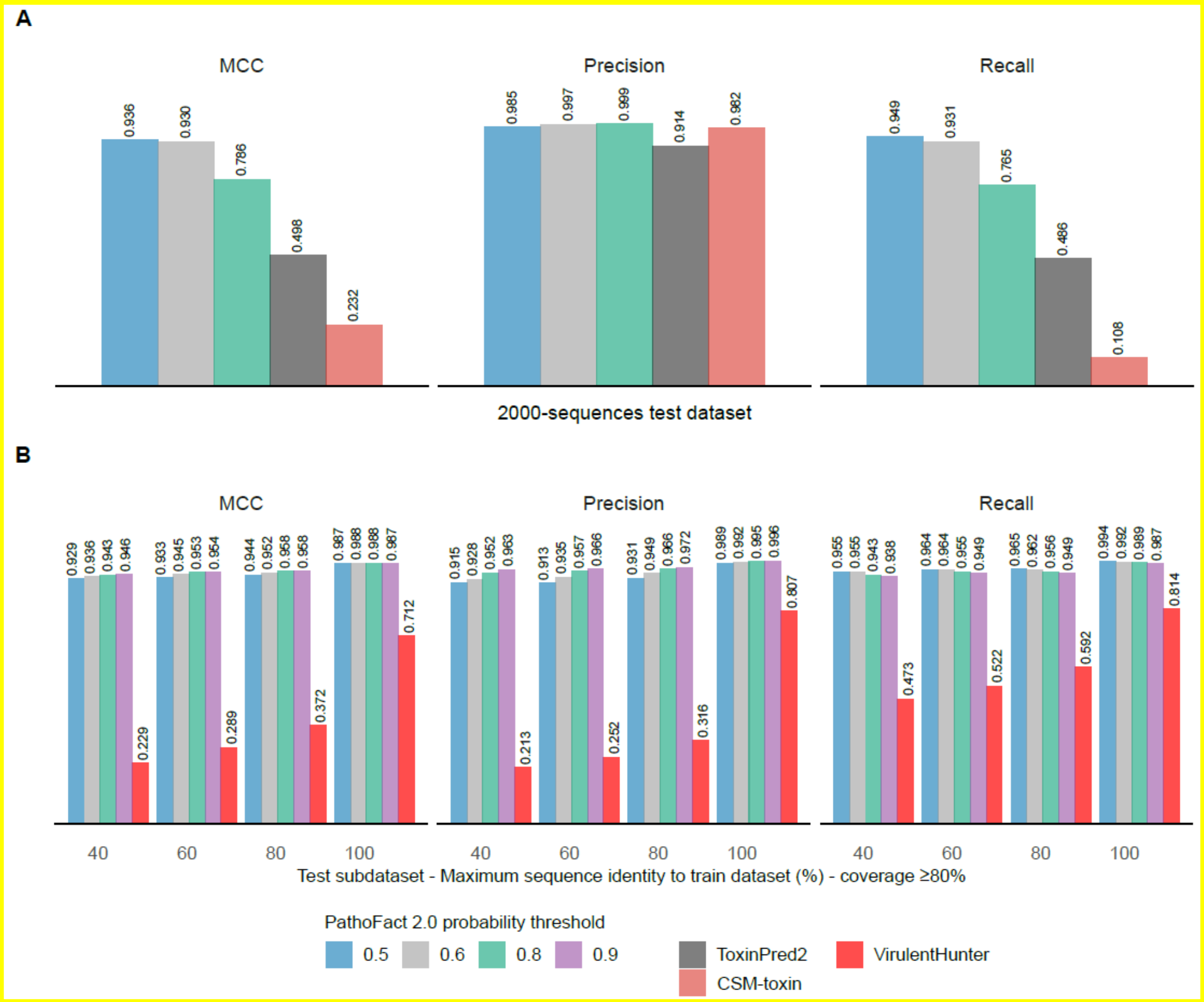

Figure 5. Benchmarking of toxin and virulence factor prediction performance. A) Toxin-associated module benchmarking. The Pathofact 2.0 toxin-associated module was compared with ToxinPred2 (Hybrid: RF+BLAST+MERCI, threshold = 0.6) using its web-based version and CSM-toxin v 1.0.1. A balanced toxin test dataset (1,000 toxin and 1,000 non-toxin sequences) was built from the Pathofact 2.0 toxin-associated test dataset, selecting only sequences from curated toxin sources (TADB, SecReT6, T3DB, DBETH, SwissProt), applying ToxinPred2's filtering criteria. Several predicted probability cutoffs, 0.5, 0.6 and 0.8, of the Pathofact 2.0 toxin-associated module were evaluated. MCC, precision, and recall are shown. **B)** Virulence factors module benchmarking. The Pathofact 2.0 VF module was compared to VirulentHunter. Sequences identical (100% identity, ≥80% coverage) to VirulentHunter's training data were removed from the Pathofact 2.0 test dataset. Then, test subset datasets were created based on 40–100% similarity to the Pathofact 2.0 training set. These subset datasets were created based on sequence similarity to the training dataset, with similarity levels of 40%, 60%, 80%, and 100%, and an 80% coverage threshold. Only sequences with similarity below these percentages were included in the respective test subsets. Several predicted probability cut-offs (0.5, 0.6, 0.8, and 0.9) of the Pathofact 2.0 VF module were evaluated.MCC, precision, and recall are presented for each test subset.

Virulence factors and toxin-associated protein prediction with contig sequences as input

To evaluate PathoFact 2.0 at the contig level, we analysed publicly available complete genomes from pathogenic and non-pathogenic bacteria, including various *Escherichia coli* strains. Figure 6 shows distinct differences in virulence and toxin-related profiles between pathogenic and non-pathogenic *E. coli* strains, especially regarding virulence- and toxin-associated proteins encoded on MGEs, such as

plasmids and prophages. Nonetheless, analysis of individual VF predictions reveals considerable overlap in the number of VF genes detected across both pathogenic and non-pathogenic strains. This highlights that the presence of a VF gene is not a reliable marker of pathogenicity and emphasises the importance of considering genomic and functional context when assessing virulence potential.

It is well known that VFs of pathogenic *E. coli* are often encoded on genetic elements, such as plasmids, bacteriophages, transposons, and pathogenicity islands, which can be mobilised into different strains to create novel combinations of virulence factors [54,55]. The same pattern is observed in pathogenic strains of several genera compared to non-pathogenic strains (Supplementary Figure S1), particularly for *Klebsiella pneumoniae* and *Salmonella enterica*. These findings highlight the importance of examining virulence from a systems perspective rather than focusing solely on the presence or absence of individual factors. A comprehensive assessment should consider not only whether a virulence- or toxin-associated protein is encoded within an MGE but also its functional context, such as whether it is secreted or part of a BGC.

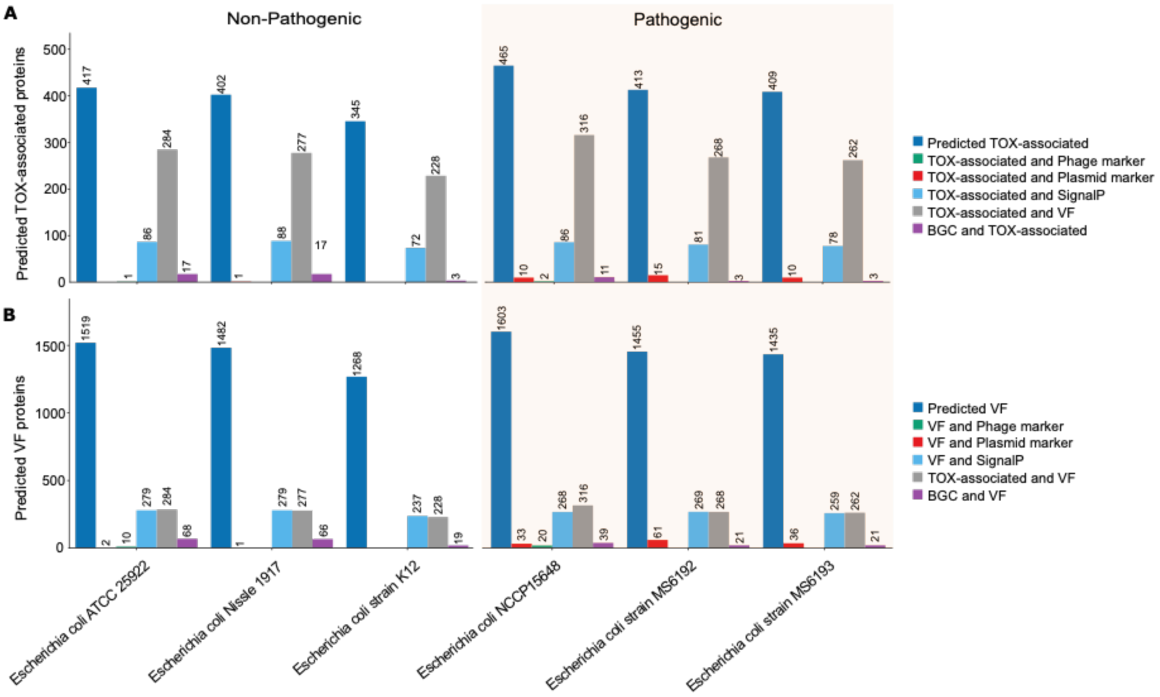

Figure 6. Comparative analysis of toxin-associated and virulence factor profiles in non-pathogenic and pathogenic *Escherichia coli* strains. Bar charts represent the distribution of predicted toxin-associated (A) and virulence-associated (B) proteins across non-pathogenic (left panel) and pathogenic (right panel) *E. coli* strains. The categories include total predicted virulence factors/toxin-associated proteins (dark blue), those associated with plasmid markers (red), those associated with phage markers (green), and proteins predicted by SignalP to be secreted (light blue). Additional categories include toxin-associated virulence factors (TOX-associated and VF, grey) and biosynthetic gene clusters overlapping (purple). Numerical values above each bar indicate the total count of proteins identified in each category for the corresponding strain.

Benchmarking

The PathoFact 2.0 VF module was compared to PathoFact 1.0 and metaVF [23], an alignment-based toolkit (based on BLAST) that identifies species-level VFs associated with pathobionts. To our knowledge, no other method is available to predict VF from contig sequences and identify plasmid-encoded or

prophage-associated VF. However, we included VirulentHunter in the comparison. Because VirulentHunter does not accept contig sequences as input, protein-coding genes were first predicted from contigs using Pyrodigal-gv and the resulting protein sequences were subsequently analysed. PathoFact 2.0 consistently detected a greater number of VFs than both PathoFact 1.0 and MetaVF (Figure 7, Supplementary Figures S2–S3). Notably, MetaVF failed to identify any VFs in five of the ten pathogenic reference strains tested, highlighting its limited ability to detect VFs and demonstrating the advantages of machine-learning-based models over homology-based approaches. While VirulentHunter produced substantially more hits than metaVF, yielding predictions comparable in number to PathoFact 2.0, PathoFact 2.0 generally identified more VFs overall (Figure 7). Exceptions were observed for non-pathogenic strains *Bifidobacterium animalis*, *Bifidobacterium bifidum*, *Heyndrickxia coagulans*, and for the pathogenic *Ralstonia mannitolilytica strain Guangzhou-RMAB10*, where VirulentHunter predicted slightly more VFs.

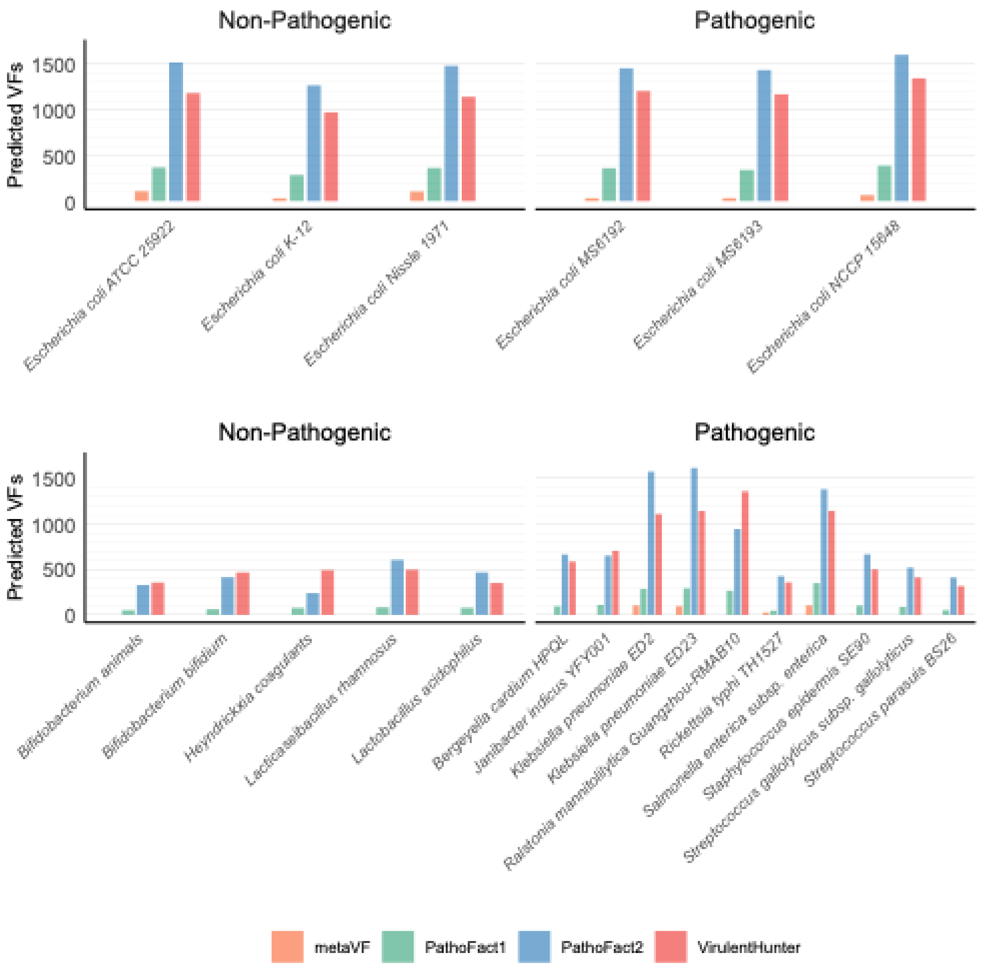

Figure 7. Performance comparison of virulence factor prediction tools in pathogenic and non-pathogenic strains. Comparative performance of PathoFact 2.0 (blue), PathoFact (green), metaVF (orange), and VirulentHunter (red) in predicting virulence factors (VFs). Results are shown separately for non-pathogenic (left) and pathogenic (right) strains.

In addition, the PathoFact 2.0 toxin-associated module was compared to PathoFact 1.0 (Figure 8, Supplementary Figures S4–S5) as well as CSM-Toxin and ToxinPred2 (Figure 8). As CSM-Toxin and ToxinPred2 do not accept contig sequences as input, protein sequences were first predicted from contigs using Pyrodigal-gv. PathoFact 2.0 predicted more toxin-associated proteins than PathoFact 1.0, demonstrating improved detection capacity. Compared to external tools, CSM-Toxin identified substantially fewer toxins across the tested reference strains. In contrast, ToxinPred2 predicted a

comparable number of toxins overall. However, for non-pathogenic strains *Bifidobacterium bifidum*, *Heyndrickxia coagulans*, *Lactobacillus acidophilus*, and for pathogenic strains *Streptococcus gallolyticus subsp. gallolyticus* and Streptococcus parasuis B26, ToxinPred2 identified more predicted toxins than PathoFact 2.0.

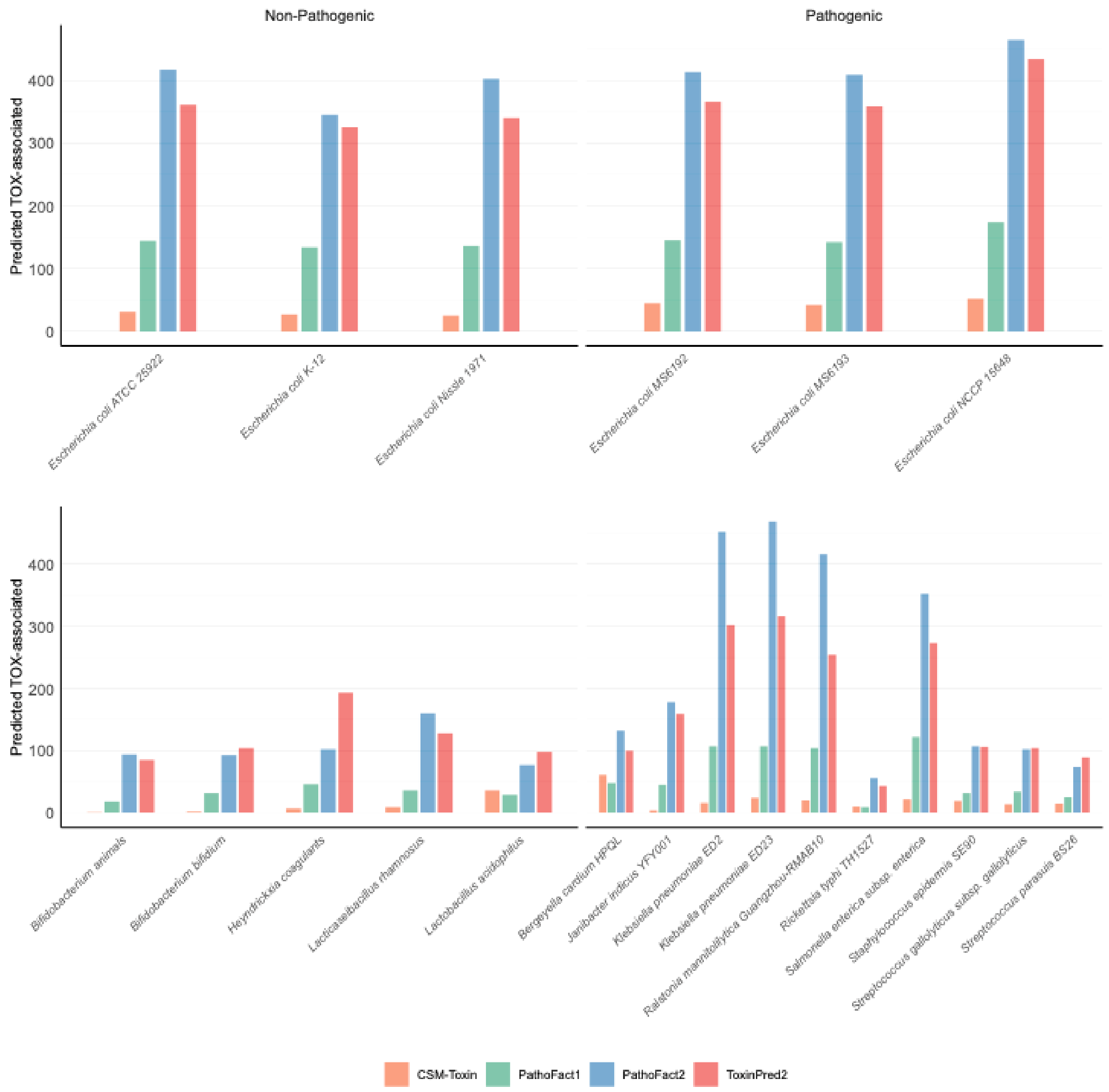

Figure 8. Performance comparison of toxin prediction tools in pathogenic and non-pathogenic strains. Comparative performance of PathoFact 2.0 (blue), PathoFact (green), CSM-Toxin (orange), and ToxinPred2 (red) in predicting virulence factors (VFs). Results are shown separately for non-pathogenic (left) and pathogenic (right) strains.

PathoFact 2.0 Output Structure

PathoFact 2.0 creates a structured output directory that summarises predictions from all analysis modules, including VFs, toxin-associated proteins, ARGs, MGEs and BGCs. Each module generates dedicated result files corresponding to the underlying prediction tools (Supplementary File S1).

The primary summary file, combined_report.tsv, provides an integrated overview of high-confidence predictions across all modules in a tabular format. This table includes key information such as protein identifiers, bit scores (from HMM profiles), machine-learning prediction scores, and outputs from DeepARG, RGI, SignalP, GenoMad, and antiSMASH, thereby supporting downstream interpretation and candidate prioritisation.

Proteins with prediction probabilities below user-defined thresholds but containing conserved domains identified by toxin-associated or VF HMM profiles are reported in ambiguous_TOX_hits_rep_prot.tsv and ambiguous_VF_hits_rep_prot.tsv. These lower-confidence candidates may warrant further investigation in comparative or experimental analyses.

High-confidence predictions are reported in amr_hits_rep_prot.tsv, TOX_hits_rep_prot.tsv, and VF_hits_rep_prot.tsv, which summarise features exceeding user-defined probability thresholds and include protein identifiers, bit scores, machine-learning predictions, signal peptide predictions, and genomic context information, such as association with prophages or plasmids identified by GenoMad.

In addition, PathoFact 2.0 generates a dedicated Group_of_sequence directory containing FASTA files of representative protein sequences grouped by functional category (VFs, toxin-associated proteins, antimicrobial resistance genes, and combined hits), together with conserved domain (CDD) annotation tables for predicted VFs and toxin-associated proteins. These files are designed to facilitate downstream analyses, including comparative genomics and functional characterisation.

Limitations of PathoFact 2.0

It is well established that non-pathogenic bacterial strains can also carry genes annotated as VFs or toxins [7]. Consequently, PathoFact 2.0 is most effective as an initial screening tool to identify potential candidates, which can then be examined in comparative studies to distinguish confirmed pathogenic cases from controls. The pipeline provides a probability score indicating whether a protein is likely to be VF- or toxin-associated; however, establishing a definitive link between predicted candidates and infectious disease requires experimental validation.

PathoFact 2.0 does not directly classify specific VF or toxin types (e.g., adhesins or genotoxins). Instead, it reports detailed annotations of conserved protein domains from CDD [18], allowing users to infer functional roles when available. This design emphasises contextual interpretation rather than categorical assignment.

The inclusion of housekeeping genes from non-pathogenic microorganisms reflects a deliberate methodological choice rather than a limitation. Multiple well-characterised housekeeping proteins have been shown to exhibit virulence-associated “moonlighting” functions in pathogenic bacteria, including roles in adhesion, immune modulation, and tissue invasion [56,57]. Notable examples include glyceraldehyde-3-phosphate dehydrogenase (VF0015 in VFDB) [58], enolase [59,60], elongation factor Tu (VF0460 in VFDB) [61,62], GroEL [60,63,64], and DnaK [60,65]. This approach maintains a biologically realistic negative dataset while reducing the risk of misclassifications within the intended scope of PathoFact 2.0.

PathoFact 2.0 is designed for metagenomic samples; most prediction modules and phenotypes are bacterial-centric. Virulence, toxin-associated, and antimicrobial resistance predictions are particularly interpreted in the context of human pathogens.

Conclusions

ARGs, VFs, and toxins represent major threats to global health. Therefore, accurate detection of these elements is crucial for assessing the presence and potential risks of pathogenic microorganisms in microbiomes and for identifying reservoirs of pathogenicity. Our improved pipeline, PathoFact 2.0, offers significant improvements over PathoFact (its predecessor), ToxinPred2, CSM-toxin, VirulentHunter, and metaVF. SignalP has been upgraded and made optional to further optimise performance, providing users with flexibility based on their requirements. Additionally, antiSMASH 7.0 facilitates the prediction of BGCs, recognising emerging evidence that some BGC-encoded factors might increase virulence. Furthermore, we have integrated geNomad, a cutting-edge tool for identifying MGEs, including plasmids and phages linked to ARGs, VFs, toxins and toxin-associated proteins across various bacterial species. The PathoFact 2.0 update improves the accuracy and sensitivity of analyses while enhancing computational efficiency.

PathoFact 2.0 represents a major advance in metagenomic analysis by integrating the detection of ARGs, VFs, toxins and toxin-associated proteins, signal peptides, MGEs, and BGCs within a single, streamlined pipeline. Unlike existing tools that focus on individual aspects of pathogenicity, PathoFact 2.0 provides a comprehensive, multi-layered view that captures both gene presence and genomic context, improving interpretability and enabling a holistic assessment of microbial pathogenic potential.

Methods

Databases used for the PathoFact 2.0 Dataset Construction

- SwissProt [31] is the expertly curated part of UniProtKB [66]. It offers high-quality protein sequences with detailed functional annotations, including keywords for pathogenesis, virulence, toxins, and antibiotic resistance.
- VFDB [43], the Virulence Factor Database, is a comprehensive reference for curating information on virulence factors of bacterial pathogens.
- T3DB [12], the toxin and toxin-target database, is a resource cataloguing thousands of toxins and their protein targets, with detailed mechanisms, structures, and toxicity data, including bacterial protein toxins.
- DBETH [13], the database for bacterial exotoxins, is a specialised database of bacterial exotoxins pathogenic to humans, classified into 24 mechanistic and activity types from 26 bacterial genera.
- TADB [36], the toxin-antitoxin database, is a repository of bacterial toxin-antitoxin loci across types I-VIII, including experimentally validated pairs, predicted loci, and associations with mobile genetic elements.
- SecReT6 [37] is a database containing known and predicted type VI secretion systems, including effectors, immunity proteins, regulators, and accessory proteins from bacterial genomes.
- PAT [38] is the prokaryotic antimicrobial toxin database and contains a collection of antimicrobial toxins, including bacteriocins and effectors from secretion systems.

Clustering Parameters

MMseqs2 [32] was used for dereplication; sequences were clustered at 100% identity and 100% coverage using the parameters -c 1.0 and --min-seq-id 1.0. It removes exact duplicates and retains representative sequences to generate non-redundant datasets. To create the test subsets, a coverage of 80% (-c 0.8) and identities ranging from 40 - 100% (--min-seq-id 0.4, --min-seq-id 0.6, --min-seq-id 0.8, --min-seq-id 1.0) were used, and then sequences with similarity higher than min-seq-id were removed for each test subset. In all cases, the cluster mode and coverage mode used were 0 (--cov-mode 0 --cluster-mode 0). When --cov-mode 0 is specified in combination with −c values ranging from 0.0 to 1.0, sequences are assigned to the same cluster only if the alignment spans at least a fraction *c* of the length of the longer sequence. According to the developers of MMSeq2, this coverage criterion is particularly suitable for clustering full-length protein sequences [32].

Protein Composition Features

Protein sequence composition features were extracted to represent each protein as fixed-length vectors derived from its primary amino acid sequence [35]. These included amino acid composition (AAC), dipeptide composition (DPC), composition (CTDC), transition (CTDT) and distribution (CTDD). AAC captures the relative frequency of each of the 20 amino acids in a sequence, whereas DPC captures the relative frequency of all adjacent amino-acid pairs (400 possible dipeptides). CTDC represents the percentage of amino acids belonging to each of three predefined groups (polar, neutral, hydrophobic) in the entire protein sequence. CTDT represents the percentage frequency with which a residue of one group is followed by a residue of a different group along the sequence. CTDD represents the distribution of each amino acid group, measuring the spatial position, where the first, 25%, 50%, 75% and 100% of the residues of a specific class are located.

Performance and Evaluation Metrics

To assess the models' performance, we used a confusion matrix comprising true positives (TP), true negatives (TN), false positives (FP), and false negatives (FN), computed on the test datasets described above. TP corresponds to truly positive instances correctly predicted as positive by the model, whereas TN corresponds to truly negative instances correctly predicted as negative. FP are truly negative instances incorrectly predicted as positive, and FN are truly positive instances incorrectly predicted as negative. From these values, we calculated the following metrics:

$$Accuracy = (TP + TN)/(TP + TN + FP + FN)$$

$$Precision = TP/(TP + FP)$$

$$Recall = TP/(TP + FN)$$

$$MCC = (TP \times TN - FP \times FN)/\sqrt{(TP + FP)(TP + FN)(TN + FP)(TN + FN)}$$

HMM Profiles

Profile hidden Markov models are probabilistic models built from a multiple sequence alignment that encode, for each alignment position, the position-specific probabilities of residues and insertions/deletions, turning the alignment into a position-specific scoring system for detecting homologous sequences [67]. The FASTA files of conserved-domain multiple sequence alignments for each CDD [26] family (https://ftp.ncbi.nih.gov/pub/mmdb/cdd/fasta.tar.gz) were downloaded. Pyhmmer v0.10.14 [68] was used to obtain HMM profiles for each CDD family and to perform protein sequence searches against the CDD HMM family profiles.

Benchmarking Datasets

This study utilised publicly available datasets containing complete genomes from pathogenic and non-pathogenic bacteria, including various *Escherichia coli* strains, from NCBI. The accession numbers of the bacteria used are indicated in Supplementary Table S2.

Availability of Supporting Source Code and Requirements

Project name: Pathofact 2.0
Project homepage: https://gitlab.com/uniluxembourg/lcsb/systems-ecology/pathofact2
Operating system(s): Linux
Programming language: Python, R, bash
Other requirements: Snakemake, Mamba, conda.
License: GNU General License v3.0 or later
Biotools ID: pathofact2
RRID: SCR_027650
workflowhubDOI: 10.48546/workflowhub.workflow.2087.1

Additional Files

Supplementary Table S1. List of microorganisms non-pathogenic to humans and their total protein count obtained from the NCBI Database.

Supplementary Table S2. Bacterial strains used in this study, including their classification as pathogenic or non-pathogenic, species/strain information, genome assembly or reference version, and corresponding accession numbers.

Supplementary Table S3. Evaluation of the PathoFact 2.0 toxin-associated protein prediction module. The table presents performance across test subsets defined by sequence similarity to the training set. Metrics reported include class distributions (Negative, non-toxin; Positive, toxin-associated), confusion matrix counts (true negatives, false positives, true positives, false negatives), and performance measures (accuracy, precision, recall, F1 score, Matthews correlation coefficient).

Supplementary Table S4. Evaluation of the PathoFact 2.0 virulence factor prediction module. The table summarises performance across test subsets defined by sequence similarity to the training set. Reported metrics include class distributions (Negative, non-VF; Positive, VF), confusion matrix counts, and performance measures (accuracy, precision, recall, F1 score, Matthews correlation coefficient).

Supplementary Table S5. Comparison of virulence factor prediction performance for PathoFact 2.0 at varying probability cutoffs and for VirulenHunter, evaluated across test subsets stratified by sequence similarity to the training set. The table reports class distributions, confusion matrix counts, and performance metrics (accuracy, precision, recall, F1 score, Matthews correlation coefficient).

Supplementary Table S6. Comparison of toxin protein prediction performance for PathoFact 2.0 at different prediction probability cutoffs and for ToxinPred2. The table reports the number of proteins classified as Negative (non-toxin) and Positive (toxin), confusion matrix counts, and associated performance metrics (accuracy, precision, recall, F1 score, and Matthews correlation coefficient).

Supplementary Figure S1. Comparative analysis of toxin-associated and virulence factor profiles in non-pathogenic and pathogenic bacterial strains. Bar charts represent the distribution of predicted toxin-associated (A) and virulence-associated (B) proteins across non-pathogenic (left panel) and pathogenic (right panel) *E. coli* strains. The categories include total predicted virulence factors/toxin-associated proteins (dark blue), associated with plasmid markers (red), phage markers (Green), and proteins predicted by SignalP to be secreted (light blue). Additional categories include toxin-associated virulence factors (TOX-related ∩ VF, grey) and biosynthetic gene clusters overlapping (purple). Numerical values above each bar indicate the total count of proteins identified in each category for the corresponding strain.

Supplementary Figure S2. Comparative performance of Pathofact 2.0 (blue) versus PathoFact1 (green) and metaVF (red) in predicting virulence factors (VFs) in non-pathogenic (left) and pathogenic (right) *Escherichia coli* strains. The top panel shows the total number of predicted VFs. The second panel depicts the subset predicted to be secreted. The third panel shows VFs predicted to be plasmid-encoded, while the fourth panel presents those predicted to be prophage-associated.

Supplementary Figure S3. Comparative performance of Pathofact 2.0 (blue) versus PathoFact (green) and metaVF (red) in predicting virulence factors (VFs) in non-pathogenic (left) and pathogenic (right) bacterial strains. The top panel shows the total number of predicted VFs. The second panel depicts the subset predicted to be secreted. The third panel shows VFs predicted to be plasmid-encoded, while the fourth panel presents those predicted to be prophage-associated.

Supplementary Figure S4. Performance comparison of PathoFact 2.0 versus PathoFact for ARG and toxin prediction in Escherichia coli. A) Comparative performance of PathoFact 2.0 (blue) versus PathoFact (green) in predicting antimicrobial resistance genes (ARGs) in non-pathogenic (left) and pathogenic (right) *E. coli* strains. **B)** Comparative performance of PathoFact 2.0 (blue) versus PathoFact (green) in predicting toxin-associated proteins in non-pathogenic (left) and pathogenic (right) *E. coli* strains. The top panel shows the total number of predicted toxin-associated proteins, while the bottom panel shows those that contain signal peptides, as identified by SignalP.

Supplementary Figure S5. Performance comparison of PathoFact 2.0 versus PathoFact for ARG and toxin prediction in bacterial strains. A) Comparative performance of PathoFact 2.0 (blue) versus PathoFact (green) in predicting antimicrobial resistance genes (ARGs) in non-pathogenic (left) and pathogenic (right) bacterial strains. **B)** Comparative performance of PathoFact 2.0 (blue) versus PathoFact (green) in predicting toxin-associated proteins in non-pathogenic (left) and pathogenic (right) bacterial strains. The top panel shows the total number of predicted toxin-associated proteins, while the bottom panel shows those that contain signal peptides, as identified by SignalP.

Abbreviations

AAC, amino acid composition; ARO, antibiotic resistance ontology; ARGs, antimicrobial resistance genes; BGCs, biosynthetic gene clusters; CDD, conserved domains database; CTDC, (Composition,

Transition, Distribution)-composition; CTDD, (Composition, Transition, Distribution)-distribution; CTDT, (Composition, Transition, Distribution)-transition; DBETH, database for bacterial exotoxins; DPC, dipeptide composition; HMMs, hidden Markov models; MCC, Matthews correlation coefficient; MGEs, mobile genetic elements; ML, machine learning; ORF, open reading frame; PAT, prokaryotic antimicrobial toxins database; RF, random forest; SM, specialised metabolites; SMOTE, synthetic minority oversampling technique; SNP, single nucleotide polymorphisms; T3DB, toxin exposome database; VFDB, virulence factor database; VFs, virulence factors.

Acknowledgements

The experiments presented in this paper were carried out using the HPC facilities of the University of Luxembourg (Varrette et al., 2022). The manuscript also passed the Luxembourg Centre for Systems Biomedicine internal pre-publication check designed to ensure FAIRness and reproducibility.

Author Contributions

P.W. initiated the study, which involved the overall design and objective, and was led by L.F.D. and J.O.S. in the development of PathoFact 2.0. O.H. contributed to early brainstorming discussions on workflow design and database strategy. P.M. and C.C.L. contributed to the overall discussions. L.F.D. and J.O.S. wrote the draft manuscript. All authors read and commented on the manuscript.

Funding

This work has been supported by the Pélican grant from the Mie and Pierre Hippert-Faber Pélican Foundation under the aegis of Fondation de Luxembourg to JOS, as well as by the Luxembourg National Research Fund (FNR CORE/23/BM/15886415) and the European Research Council (ERC-CoG 863664) to PW. The Luxembourg Government further supported the work through the CoVaLux program.
This research was funded in whole, or in part, by the Luxembourg National Research Fund (FNR), grant reference (FNR CORE/23/BM/15886415). For the purpose of open access, and in fulfilment of the obligations arising from the grant agreement, the author has applied a Creative Commons Attribution 4.0 International (CC BY 4.0) license to any Author Accepted Manuscript version arising from this submission.

Data Availability

Pathofact 2.0 is accessible at https://gitlab.com/uniluxembourg/lcsb/systems-ecology/pathofact2. Additionally, the core databases required to run the pipeline can be found at https://zenodo.org/records/14192463. The ML datasets used for training, validation, and benchmarking of PathoFact 2.0 can be found in https://zenodo.org/records/17647372.

Competing Interests

None declared.

References

1. Hou K, Wu Z-X, Chen X-Y, Wang J-Q, Zhang D, Xiao C, et al.. Microbiota in health and diseases.

Signal Transduct Target Ther. Springer Science and Business Media LLC; 7:1352022;

2. Inda-Díaz JS, Lund D, Parras-Moltó M, Johnning A, Bengtsson-Palme J, Kristiansson E. Latent antibiotic resistance genes are abundant, diverse, and mobile in human, animal, and environmental microbiomes. *Microbiome*. Springer Science and Business Media LLC; 11:442023;

3. Beceiro A, Tomás M, Bou G. Antimicrobial resistance and virulence: a successful or deleterious association in the bacterial world? *Clin Microbiol Rev*. American Society for Microbiology; 26:185–2302013;

4. Zhu C, Wu L, Ning D, Tian R, Gao S, Zhang B, et al.. Global diversity and distribution of antibiotic resistance genes in human wastewater treatment systems. *Nat Commun*. Springer Science and Business Media LLC; 16:40062025;

5. Jian Z, Zeng L, Xu T, Sun S, Yan S, Yang L, et al.. Antibiotic resistance genes in bacteria: Occurrence, spread, and control. *J Basic Microbiol*. Wiley; 61:1049–702021;

6. Alcock BP, Huynh W, Chalil R, Smith KW, Raphenya AR, Wlodarski MA, et al.. CARD 2023: expanded curation, support for machine learning, and resistome prediction at the Comprehensive Antibiotic Resistance Database. *Nucleic Acids Res*. Oxford University Press (OUP); 51:D690–92023;

7. Niu C, Yu D, Wang Y, Ren H, Jin Y, Zhou W, et al.. Common and pathogen-specific virulence factors are different in function and structure. *Virulence*. Informa UK Limited; 4:473–822013;

8. Sharma AK, Dhasmana N, Dubey N, Kumar N, Gangwal A, Gupta M, et al.. Bacterial virulence factors: Secreted for survival. *Indian J Microbiol*. 57:1–102017;

9. Blair JMA, Webber MA, Baylay AJ, Ogbolu DO, Piddock LJV. Molecular mechanisms of antibiotic resistance. *Nat Rev Microbiol*. Springer Science and Business Media LLC; 13:42–512015;

10. Rodríguez-Beltrán J, DelaFuente J, León-Sampedro R, MacLean RC, San Millán Á. Beyond horizontal gene transfer: the role of plasmids in bacterial evolution. *Nat Rev Microbiol*. Springer Science and Business Media LLC; 19:347–592021;

11. Galanos C, Freudenberg MA. Bacterial endotoxins: biological properties and mechanisms of action. *Mediators Inflamm*. Wiley; 2:S11–61993;

12. Wishart D, Arndt D, Pon A, Sajed T, Guo AC, Djoumbou Y, et al.. T3DB: the toxic exposome database. *Nucleic Acids Res*. Oxford University Press (OUP); 43:D928–342015;

13. Chakraborty A, Ghosh S, Chowdhary G, Maulik U, Chakrabarti S. DBETH: A database of Bacterial ExoToxins for human. *Nucleic Acids Res*. Oxford University Press (OUP); 40:D615–202012;

14. Green ER, Mecsas J. Bacterial secretion systems: An overview. *Microbiol Spectr*. 2016; doi: 10.1128/microbiolspec.VMBF-0012-2015.

15. Kaushik S, He H, Dalbey RE. Bacterial signal peptides- navigating the journey of proteins. *Front Physiol*. Frontiers Media SA; 13:9331532022;

16. Lybbert AC, Williams JL, Raghuvanshi R, Jones AD, Quinn RA. Mining public mass spectrometry data to characterize the diversity and ubiquity of P. aeruginosa specialized metabolites. *Metabolites*. MDPI AG; 10:4452020;

17. Elshafie HS, Camele I. An overview of metabolic activity, beneficial and pathogenic aspects of

Burkholderia spp. *Metabolites*. MDPI AG; 11:3212021;

18. Lau GW, Hassett DJ, Ran H, Kong F. The role of pyocyanin in Pseudomonas aeruginosa infection. *Trends Mol Med*. Elsevier BV; 10:599–6062004;

19. Ambassadors G, Patrons: World leaders commit to decisive action on antimicrobial resistance. UN Environment. https://www.unep.org/news-and-stories/press-release/world-leaders-commit-decisive-action-antimicrobial-resistance (2024). Accessed 2025 Oct 7.

20. Environment UN: Antimicrobial Resistance (AMR). UNEP - UN Environment Programme. https://www.unep.org/topics/chemicals-and-pollution-action/pollution-and-health/antimicrobial-resistance-amr (2024). Accessed 2025 Oct 7.

21. Bansal MA, Sharma DR, Kathuria DM. A systematic review on data scarcity problem in deep learning: Solution and applications. *ACM Comput Surv*. Association for Computing Machinery (ACM); 54:1–292022;

22. de Nies L, Lopes S, Busi SB, Galata V, Heintz-Buschart A, Laczny CC, et al.. PathoFact: a pipeline for the prediction of virulence factors and antimicrobial resistance genes in metagenomic data. *Microbiome*. Springer Science and Business Media LLC; 9:492021;

23. Dong W, Fan X, Guo Y, Wang S, Jia S, Lv N, et al.. An expanded database and analytical toolkit for identifying bacterial virulence factors and their associations with chronic diseases. *Nat Commun*. Springer Science and Business Media LLC; 15:80842024;

24. Ji B, Pi W, Liu W, Liu Y, Cui Y, Zhang X, et al.. HyperVR: a hybrid deep ensemble learning approach for simultaneously predicting virulence factors and antibiotic resistance genes. *NAR Genom Bioinform*. 5:lqad0122023;

25. Rathore AS, Choudhury S, Arora A, Tijare P, Raghava GPS. ToxinPred 3.0: An improved method for predicting the toxicity of peptides. *Comput Biol Med*. Elsevier BV; 179:1089262024;

26. Wang J, Chitsaz F, Derbyshire MK, Gonzales NR, Gwadz M, Lu S, et al.. The conserved domain database in 2023. *Nucleic Acids Res*. Oxford University Press (OUP); 51:D384–82023;

27. Blin K, Shaw S, Augustijn HE, Reitz ZL, Biermann F, Alanjary M, et al.. antiSMASH 7.0: new and improved predictions for detection, regulation, chemical structures and visualisation. *Nucleic Acids Res*. Oxford University Press (OUP); 51:W46–502023;

28. . Pyrodigal: Python bindings and interface to Prodigal, an efficient method for gene prediction in prokaryotes. *Journal of Open Source Software*. doi: 10.21105/joss.04296.

29. Camargo AP, Roux S, Schulz F, Babinski M, Xu Y, Hu B, et al.. Identification of mobile genetic elements with geNomad. *Nat Biotechnol*. Springer Science and Business Media LLC; 42:1303–122024;

30. Köster J, Rahmann S. Snakemake--a scalable bioinformatics workflow engine. *Bioinformatics*. Oxford University Press (OUP); 28:2520–22012;

31. UniProt Consortium. UniProt: The universal protein knowledgebase in 2023. *Nucleic Acids Res*. Oxford University Press (OUP); 51:D523–312023;

32. Steinegger M, Söding J. MMseqs2 enables sensitive protein sequence searching for the analysis of massive data sets. *Nat Biotechnol*. 35:1026–82017;

33. Chawla NV, Bowyer KW, Hall LO, Kegelmeyer WP. SMOTE: Synthetic minority over-sampling technique. *J Artif Intell Res*. AI Access Foundation; 16:321–572002;

34. Pedregosa F, Varoquaux G, Gramfort A, Michel V, Thirion B, Grisel O, et al.. Scikit-learn: Machine Learning in Python. arXiv [cs.LG].

35. Chen Z, Zhao P, Li F, Leier A, Marquez-Lago TT, Wang Y, et al.. iFeature: a Python package and web server for features extraction and selection from protein and peptide sequences. *Bioinformatics*. 34:2499–5022018;

36. Guan J, Chen Y, Goh Y-X, Wang M, Tai C, Deng Z, et al.. TADB 3.0: an updated database of bacterial toxin-antitoxin loci and associated mobile genetic elements. *Nucleic Acids Res*. Oxford University Press (OUP); 52:D784–902024;

37. Zhang J, Guan J, Wang M, Li G, Djordjevic M, Tai C, et al.. SecReT6 update: a comprehensive resource of bacterial Type VI Secretion Systems. *Sci China Life Sci*. Springer Science and Business Media LLC; 66:626–342023;

38. Liu Y, Liu S, Pan Z, Ren Y, Jiang Y, Wang F, et al.. PAT: a comprehensive database of prokaryotic antimicrobial toxins. *Nucleic Acids Res*. Oxford University Press (OUP); 51:D452–92023;

39. Harms A, Liesch M, Körner J, Québatte M, Engel P, Dehio C. A bacterial toxin-antitoxin module is the origin of inter-bacterial and inter-kingdom effectors of Bartonella. *PLoS Genet*. 13:e10070772017;

40. Yadav SK, Magotra A, Ghosh S, Krishnan A, Pradhan A, Kumar R, et al.. Immunity proteins of dual nuclease T6SS effectors function as transcriptional repressors. *EMBO Rep*. EMBO; 22:e518572021;

41. Danov A, Segev O, Bograd A, Ben Eliyahu Y, Dotan N, Kaplan T, et al.. Toxinome-the bacterial protein toxin database. *MBio*. 15:e01911232024;

42. Sordo M, Zeng Q. On sample size and classification accuracy: A performance comparison. *Biological and Medical Data Analysis*. Berlin, Heidelberg: Springer Berlin Heidelberg; p. 193–201.

43. Liu B, Zheng D, Zhou S, Chen L, Yang J. VFDB 2022: a general classification scheme for bacterial virulence factors. *Nucleic Acids Res*. Oxford University Press (OUP); 50:D912–72022;

44. Arango-Argoty G, Garner E, Pruden A, Heath LS, Vikesland P, Zhang L. DeepARG: a deep learning approach for predicting antibiotic resistance genes from metagenomic data. *Microbiome*. 6:232018;

45. Feldgarden M, Brover V, Gonzalez-Escalona N, Frye JG, Haendiges J, Haft DH, et al.. AMRFinderPlus and the Reference Gene Catalog facilitate examination of the genomic links among antimicrobial resistance, stress response, and virulence. *Sci Rep*. Springer Science and Business Media LLC; 11:127282021;

46. Ugarcina Perovic S, Ramji V, Chong H, Duan Y, Maguire F, Coelho LP. argNorm: normalization of antibiotic resistance gene annotations to the Antibiotic Resistance Ontology (ARO). *Bioinformatics*. Oxford University Press (OUP); 2025; doi: 10.1093/bioinformatics/btaf173.

47. Chicco D, Jurman G. The advantages of the Matthews correlation coefficient (MCC) over F1 score and accuracy in binary classification evaluation. *BMC Genomics*. Springer Science and Business Media LLC; 21:62020;

48. Chen C, Xu Y, Ouyang J, Xiong X, Łabaj PP, Chmielarczyk A, et al.. VirulentHunter: deep learning-based virulence factor predictor illuminates pathogenicity in diverse microbial contexts. *Brief*

Bioinform. Oxford University Press (OUP); 26:bbaf2712025;

49. Gupta A, Malwe AS, Srivastava GN, Thoudam P, Hibare K, Sharma VK. MP4: a machine learning based classification tool for prediction and functional annotation of pathogenic proteins from metagenomic and genomic datasets. *BMC Bioinformatics*. Springer Science and Business Media LLC; 2022; doi: 10.1186/s12859-022-05061-7.

50. Sharma A, Garg A, Ramana J, Gupta D. VirulentPred 2.0: An improved method for prediction of virulent proteins in bacterial pathogens. *Protein Sci*. Wiley; 2023; doi: 10.1002/pro.4808.

51. Xie R, Li J, Wang J, Dai W, Leier A, Marquez-Lago TT, et al.. DeepVF: a deep learning-based hybrid framework for identifying virulence factors using the stacking strategy. *Brief Bioinform*. Oxford University Press (OUP); 2021; doi: 10.1093/bib/bbaa125.

52. Sharma N, Naorem LD, Jain S, Raghava GPS. ToxinPred2: an improved method for predicting toxicity of proteins. *Brief Bioinform*. Oxford University Press (OUP); 23:bbac1742022;

53. Morozov V, Rodrigues CHM, Ascher DB. CSM-Toxin: A web-server for predicting protein toxicity. *Pharmaceutics*. MDPI AG; 15:4312023;

54. Kaper JB, Nataro JP, Mobley HL. Pathogenic Escherichia coli. *Nat Rev Microbiol*. Springer Science and Business Media LLC; 2:123–402004;

55. Johnson TJ, Nolan LK. Pathogenomics of the virulence plasmids of Escherichia coli. *Microbiol Mol Biol Rev*. American Society for Microbiology; 73:750–742009;

56. Henderson B, Martin A. Bacterial virulence in the moonlight: multitasking bacterial moonlighting proteins are virulence determinants in infectious disease. *Infect Immun*. American Society for Microbiology; 79:3476–912011;

57. Henderson B, Martin A. Bacterial moonlighting proteins and bacterial virulence. *Curr Top Microbiol Immunol*. 358:155–2132013;

58. Pancholi V, Fischetti VA. A major surface protein on group A streptococci is a glyceraldehyde-3-phosphate-dehydrogenase with multiple binding activity. *J Exp Med*. Rockefeller University Press; 176:415–261992;

59. Henderson B, Martin A. Bacterial moonlighting proteins and bacterial virulence. *Curr Top Microbiol Immunol*. Curr Top Microbiol Immunol; 358:155–2132013;

60. Henderson B, Martin A. Bacterial virulence in the moonlight: multitasking bacterial moonlighting proteins are virulence determinants in infectious disease. *Infect Immun*. American Society for Microbiology; 79:3476–912011;

61. Barel M, Charbit A. Detection of the interaction between host and bacterial proteins: eukaryotic nucleolin interacts with Francisella elongation factor Tu. *Methods Mol Biol*. Methods Mol Biol; 1197:123–392014;

62. Granato D, Bergonzelli GE, Pridmore RD, Marvin L, Rouvet M, Corthésy-Theulaz IE. Cell surface-associated elongation factor Tu mediates the attachment of Lactobacillus johnsonii NCC533 (La1) to human intestinal cells and mucins. *Infection and immunity*. Infect Immun; 2004; doi: 10.1128/iai.72.4.2160-2169.2004.

63. Kamiya S, Yamaguchi H, Osaki T, Taguchi H. A virulence factor of Helicobacter pylori: role of heat

shock protein in mucosal inflammation after H. pylori infection. *J Clin Gastroenterol*. 27 Suppl 1:S35–91998;

64. Hickey TBM, Ziltener HJ, Speert DP, Stokes RW. Mycobacterium tuberculosis employs Cpn60.2 as an adhesin that binds CD43 on the macrophage surface: M. tuberculosis Cpn60.2 mediates macrophage binding via CD43. *Cell Microbiol*. Hindawi Limited; 12:1634–472010;

65. Lehner T, Bergmeier LA, Wang Y, Tao L, Sing M, Spallek R, et al.. Heat shock proteins generate β-chemokines which function as innate adjuvants enhancing adaptive immunity. *Eur J Immunol*. 30:594–6032000;

66. UniProt Consortium. UniProt: The universal protein knowledgebase in 2025. *Nucleic Acids Res*. Oxford University Press (OUP); 53:D609–172025;

67. Eddy SR. Profile hidden Markov models. *Bioinformatics*. Oxford University Press (OUP); 14:755–631998;

68. Larralde M, Zeller G. PyHMMER: a Python library binding to HMMER for efficient sequence analysis. *Bioinformatics*. Oxford Academic; 39:btad2142023;
