# Supplementary material for: PathoFact 2.0: an integrative pipeline for the prediction of antimicrobial resistance genes, virulence factors, toxins and toxin-associated proteins, and biosynthetic gene clusters in metagenomes
Source: Gigascience. 2026 May 22;15:giag062. doi: 10.1093/gigascience/giag062 (PMC13224393; doi:10.1093/gigascience/giag062)
Supplement: giag062_GIGA-D-25-00455_revision_2 [file giag062_giga-d-25-00455_revision_2.pdf]

GigaScience

PathoFact 2.0: An Integrative Pipeline for the Prediction of Antimicrobial Resistance Genes, Virulence Factors, Toxins and Toxin-associated Proteins, and Biosynthetic Gene Clusters in Metagenomes

--Manuscript Draft--

**Manuscript Number:**	GIGA-D-25-00455R2
**Full Title:**	PathoFact 2.0: An Integrative Pipeline for the Prediction of Antimicrobial Resistance Genes, Virulence Factors, Toxins and Toxin-associated Proteins, and Biosynthetic Gene Clusters in Metagenomes
**Article Type:**	Technical Note
**Funding Information:**	Fondation du Pélican de Mie et Pierre Hippert-Faber (Pélican Grant) — Miss Júlia Ortís Sunyer<br>Fonds National de la Recherche Luxembourg (FNR CORE/23/BM/15886415) — Dr. Paul Wilmes<br>European Research Council (ERC-CoG 863664) — Dr. Paul Wilmes
**Abstract:**	Background<br>Antimicrobial resistance genes (ARG) and virulence factors (VFs) are central contributors to the global health crisis surrounding drug-resistant infections.<br><br>Findings<br>We introduce PathoFact 2.0, an enhanced pipeline for improved ARG, VF, toxin, and biosynthetic gene clusters (BGC) prediction. Key improvements include an updated machine learning (ML) model for VF identification, expanded hidden Markov model profiles for VFs and toxin-associated proteins, a new ML model for toxin and toxin-associated proteins identification, and the integration of antiSMASH 7.0 for predicting biosynthetic gene clusters.<br><br>Conclusions<br>Our upgrades make PathoFact 2.0 a more powerful and user-friendly platform for predicting microbiome-based pathogenicity and resistance, providing a crucial tool for better understanding and addressing the challenges posed by antimicrobial resistance and infectious diseases.<br>PathoFact 2.0 is available at https://gitlab.com/uniluxembourg/lcsb/systems-ecology/pathofact2. It is compatible with Linux operating systems.
**Corresponding Author:**	Paul Wilmes<br>University of Luxembourg Luxembourg Centre for Systems Biomedicine: Universite du Luxembourg Luxembourg Centre for Systems Biomedicine<br>Esch-sur-Alzette, LUXEMBOURG
**Corresponding Author Secondary Information:**	
**Corresponding Author's Institution:**	University of Luxembourg Luxembourg Centre for Systems Biomedicine: Universite du Luxembourg Luxembourg Centre for Systems Biomedicine
**Corresponding Author's Secondary Institution:**	
**First Author:**	Luis Fernando Delgado, PhD
**First Author Secondary Information:**	
**Order of Authors:**	Luis Fernando Delgado, PhD<br>Júlia Ortís Sunyer, MSc<br>Cedric Christian Laczny, PhD<br>Oskar Hickl, PhD

Powered by Editorial Manager® and ProduXion Manager® from Aries Systems Corporation

	Patrick May, PhD
	Paul Wilmes, PhD
**Order of Authors Secondary Information:**	

Response to Reviewers:

Reviewer reports:

We would like to thank reviewers for their thorough review of the manuscript. We appreciate their comments and believe that these contributions have significantly improved the paper. Below are our responses to each comment, along with the corresponding manuscript text for readability.

Reviewer reports:
Reviewer #2: Overall, the authors have addressed partially my concerns, which is very satisfactory. Some comments have been left unresolved, but that is quite common when communicating by emails/comments. In particular, the article still struggle with comparing to other methods (or previous PathoFacts). It does make sense, in particular with other methods, as for example PathoFact predicts toxin-associated (while to other methods might just predict toxins). I believe my comments below give a clear path of how to have this article ready for publication.

Comments 6-9: Issues solved.

R/ We thank the reviewer for their acknowledgement of our satisfactory earlier revisions.

Comment 10: The issue in Figure 4 persists. The y-axis range are not visualized, and they differ from graph to graph. One would expect that in Precision and Recall, the y-axis would go from 0-1; in MCC, from -1 to 1, but it is also very common just to use 0-1. I must insist that the current layout is misleading.

R/ We thank the reviewer for the comment. In accordance with the comment, we have added the Y-axis range to figures 4 and 5. Although the Y-axis ranges are the same across figures, they have been limited to reduce visual saturation.

Comments 11-12: Addressed. If the authors have tried and they say this is the best, I believe them.

R/ We thank the reviewer for their acknowledgement of our satisfactory earlier revisions.

Comment 13: I was actually not asking for a detailed description of the parameters. I just believe using "maximum depth" is better than "max_depth". But that is also an aesthetic decision, so if the editor is fine with it, it is also fine with me.

R/ We thank the reviewer for the comment. We have added a detailed description of the parameters, lines 224-226 and 244-245.

Comment 14: Unfortunately, I must strongly disagree with the authors. Yes, MCC is a very good approach to evaluate models (particularly on imbalanced datasets); but if the authors defend that threshold fine-tuning is crucial for the model, AUROC measures are necessary. And the conception of AUROC being not a correct measure for imbalanced datasets have been proven wrong in different articles, among:
* McDermott MB, Zhang H, Hansen LH, Angelotti G, Gallifant J (2024) A Closer Look at AUROC and AUPRC under Class Imbalance. In: Advances in Neural Information Processing Systems. pp 44102-44163
https://eur01.safelinks.protection.outlook.com/?url=https%3A%2F%2Fproceedings.neurips.cc%2Fpaper_files%2Fpaper%2F2024%2Fhash%2F4df3510ad02a86d69dc32388d91606f8-Abstract-Conference.html&data=05%7C02%7Cpaul.wilmes%40uni.lu%7C7db2d2e5b64f48209

6f308de9486c817%7C445a9c950f9d49539db1bc4a45dd1220%7C0%7C0%7C639111503821921584%7CUnknown%7CTWFpbGZsb3d8eyJFbXB0eU1hcGkiOnRydWUsIlYiOiIwLjAuMDAwMCIsIlAiOiJXaW4zMiIsIkFOIjoiTWFpbCIsIldUIjoyfQ%3D%3D%7C0%7C%7C%7C&sdata=M7lZ2jn9vdIimLRie4cLhMIX4LYxiXJoNAiy795f698%3D&reserved=0

* Richardson E, Trevizani R, Greenbaum JA, Carter H, Nielsen M, Peters B (2024) The receiver operating characteristic curve accurately assesses imbalanced datasets. Patterns 5(6):100994. https://eur01.safelinks.protection.outlook.com/?url=https%3A%2F%2Fdoi.org%2F10.1016%2Fj.patter.2024.100994&data=05%7C02%7Cpaul.wilmes%40uni.lu%7C7db2d2e5b64f482096f308de9486c817%7C445a9c950f9d49539db1bc4a45dd1220%7C0%7C0%7C639111503821956310%7CUnknown%7CTWFpbGZsb3d8eyJFbXB0eU1hcGkiOnRydWUsIlYiOiIwLjAuMDAwMCIsIlAiOiJXaW4zMiIsIkFOIjoiTWFpbCIsIldUIjoyfQ%3D%3D%7C0%7C%7C%7C&sdata=QkiL2lT8RFC05OR8FDLmfa28N9gFxFNtbXnXE6DGjV0%3D&reserved=0

R/ We thank the reviewer for this important clarification and for highlighting recent literature on the use of AUROC under class imbalance. Our intention was not to suggest that AUROC is inappropriate for imbalanced datasets, but rather to emphasise its limitations for threshold-dependent decision-making.

Here is an example of how the AUROC figures compare PathoFact 2.0 and VirulentHunter (Supplemental Figure S3)

Here is an example of the AUROC figure comparing PathoFact 2.0 and CSM-Toxin (Supplemental Figure S4)

Such AUROC figures are not possible with Toxinpred2, since its predictions are based on a combination of factors rather than on machine-learning probabilities alone. Nor are they possible with PathoFact 1.0, because it also uses a combination of machine learning and HMM classification. MetaVF is an alignment-based algorithm, and it does not generate probability scores; therefore, AUROC curves are not possible.

AUROC evaluates a classifier's ranking performance across all possible thresholds, whereas our study focuses on selecting an operational probability threshold for practical application. In this context, threshold-dependent metrics are required. We chose MCC for this purpose because MCC incorporates all elements of the confusion matrix (TP, TN, FP, FN), providing a balanced evaluation even under strong class imbalance. Nonetheless, the AUROC figures have been included in the manuscript as supplementary figures S3 and S4 (lines 351-352).

Comment 15: The answer from the authors do not have anything to do with my comment. So I will write it more clearly: if you release a new version of PathoFact (PathoFact2), you should benchmark it against your older version (PathoFact1.0). And if it not only includes an updated database, you should test PathoFact2 against PathoFact 1.0 with the new database. Right now there is only a comparison on how many more VF and toxins PathoFact2 detects, which is not a Performance evaluation (see Comment 18). A proper Performance evaluation (accuracy measures, such as MCC, Precision, Recall, AUROC) is necessary. It has been done against other ML methods, but not against the older method.

R/ We thank the reviewer for clarifying this point. We agree that benchmarking PathoFact 2.0 against the previous version (PathoFact 1.0) is important whenever possible.
PathoFact 1.0 accepts contigs as input, whereas PathoFact 2.0 accepts both contigs and protein sequences, representing one of the improvements in the updated pipeline. To enable a comparison, we use the corresponding gene nucleotide sequences as input for PathoFact 1.0. However, a full metric-based comparison on this dataset would be inherently biased. The negative dataset used in the benchmark was originally constructed and filtered using PathoFact 1.0, which suppresses false positives for PathoFact 1.0 by design. As a result, PathoFact 1.0 achieves artificially perfect precision on this dataset, rendering precision-based metrics such as MCC unreliable,

	as they rely on a biased confusion matrix.<br>Furthermore, AUROC cannot be calculated for PathoFact 1.0 predictions. The virulence factor module combines machine-learning probability scores with HMM domain detection, whereas the toxin module relies solely on HMM presence/absence rather than a continuous prediction score, thereby preventing meaningful ROC analysis.<br>To avoid biased interpretation, we therefore reported recall only for PathoFact 1.0 on this benchmark dataset. Under these conditions, PathoFact 1.0 achieved a recall of 0.287 for the toxin module, compared to 0.931 for PathoFact 2.0. For the virulence factor module, PathoFact 1.0 achieved recalls of 0.307, 0.346, 0.381, and 0.511 for test subsets 40, 60, 80, and 100, respectively, which are substantially lower than those obtained with PathoFact 2.0.<br>We have added this benchmarking analysis to the revised manuscript and clarified that the comparison with PathoFact 1.0 is limited to recall only because the benchmark dataset's design does not allow an unbiased evaluation of all performance metrics.<br>Lines 353-357:<br>“Because the negative dataset was built and then filtered using PathoFact 1.0, the precision for PathoFact 1.0 is 1, which affects the MCC calculation. To avoid biased comparisons, we report recall only for PathoFact 1.0. Figure 5 shows that PathoFact 2.0 VF and toxin-associated modules achieved higher recall across test subsets than PathoFact 1.0, and better recall and MCC than metaVF.”<br><br>Comment 16-17: Issues addressed.<br><br>R/ We thank the reviewer for their acknowledgement of our satisfactory earlier revisions.<br><br>Comment 18: Yes, I agree a full biological assessment of VF and toxins in different E. coli would be an independent study. I am more than familiar with the complexity of the strain. However, the authors use Figure 7 and 8 as "Performance Comparison" of PathoFact2.0 against other methods, and that is not what it is. What we have is just the counts of how many times each model detected a VF or a toxin. Besides clearly promoting recall over precision, there is no indication on those predictions being correct. So, statements such as "PathoFact 2.0 consistently detected a greater number of VFs than both PathoFact 1.0 and MetaVF" are incorrect, as what those graphs show is that PathoFact2.0 consistently PREDICTS a greater number of VFs. I am not saying that is a bad thing that it predicts more, but if the authors are going to be using those results the claims have to change and be very explicit that they are not using quantity as a way of Performance comparison or evaluation.<br>These types of claims also get a very vague appearance as "Our improved pipeline, PathoFact 2.0, offers significant improvements over PathoFact (its predecessor), ToxinPred2, CSM-toxin, VirulentHunter, and metaVF" in the Conclusions. The authors should be much more precise in the conclusions about what are the significant improvements. Moreover, if the authors insist in using those results, they would be much more informative showing the overlap of VF and toxins predicted by each method evaluated.<br><br>R/ We thank the reviewer for the comment. We have used DNA (gene) sequences as input for PathoFact 1.0 and MetaVF, and have now included them in Figure 5.<br><br>In addition, we have clearly shown that MetaVF does not predict VF in known pathogenic strains, highlighting a major limitation of this method, with very low recall and MCC compared with PathoFact 2.0.<br><br>PathoFact 2.0 and PathoFact 1.0 are pipelines, and we have demonstrated the advantages of PathoFact 2.0 over PathoFact 1.0 in speed, user-friendliness, updates, new tools (e.g., BGC), and the ability to use protein as input. Now that we have included PathoFact 1.0 in Figure 5, the improvements with PathoFact 2.0 are clearer.<br><br>To address the reviewer's comment, we have modified Figures 5, 7 and 8 and added two new supplementary figures, S6 and S9, which include Venn diagrams showing the overlap of VF and toxins predicted by each evaluated method.

Powered by Editorial Manager® and ProduXion Manager® from Aries Systems Corporation

In addition, we have removed the subtitle benchmarking to avoid confusion (between lines 405 and 406 of the revised manuscript).

We have modified the sentences: “PathoFact 2.0 consistently detected a greater number of VFs than both PathoFact 1.0 and MetaVF" to “PathoFact 2.0 consistently predicted a greater number of VFs than both PathoFact 1.0 and MetaVF”, in lines 410-411.

As written in the manuscript after the sentences "Our improved pipeline, PathoFact 2.0, offers significant improvements over PathoFact (its predecessor), ToxinPred2, CSM-toxin, VirulentHunter, and metaVF", we clearly expose the benefits of PathoFact 2.0 over PathoFact 1.0 (lines 488-493):

“SignalP has been upgraded and made optional to further optimise performance, providing users with flexibility based on their requirements. Additionally, antiSMASH 7.0 facilitates the prediction of BGCs, recognising emerging evidence that some BGC-encoded factors might increase virulence. Furthermore, we have integrated geNomad, a cutting-edge tool for identifying MGEs, including plasmids and phages linked to ARGs, VFs, toxins and toxin-associated proteins across various bacterial species.”

Comment 19: More or less addressed (also by previous comments).
Comment 20-21: Addressed
Comment 22-26: Agree with authors.

R/ We thank the reviewer for their acknowledgement of our satisfactory earlier revisions.

Editor Comments:
Please be informed that Reviewer 3 was unable to accept the invitation to provide comments on your revised manuscript. Instead, Reviewer 2 has assisted in reviewing the changes made in response to Reviewer 3's comments.

In addition, GigaScience has also published a number of relevant papers on metadata standardization, FAIR data practices, and reproducible workflows in biotechnology that may help contextualize your work. We have listed several recent examples below for your reference.

Citing some of these articles—where appropriate in the Background or Discussion—would help frame your contribution within ongoing community efforts and strengthen the presentation of related tools and frameworks.

1. Kasmanas J C, Magnúsdóttir S, Zhang J, et al. Integrating comparative genomics and risk classification by assessing virulence, antimicrobial resistance, and plasmid spread in microbial communities with gSpreadComp[J]. GigaScience, 2025, 14: giaf072.
https://eur01.safelinks.protection.outlook.com/?url=https%3A%2F%2Fdoi.org%2F10.1093%2Fgigascience%2Fgiaf072&data=05%7C02%7Cpaul.wilmes%40uni.lu%7C7db2d2e5b64f482096f308de9486c817%7C445a9c950f9d49539db1bc4a45dd1220%7C0%7C0%7C639111503821991703%7CUnknown%7CTWFpbGZsb3d8eyJFbXB0eU1hcGkiOnRydWUsIlYiOiIwLjAuMDAwMCIsIlAiOiJXaW4zMiIsIkFOIjoiTWFpbCIsIldUIjoyfQ%3D%3D%7C0%7C%7C%7C&sdata=lu3VScoPqNhU85T%2Bral3skRDN0Th9zmsADAob6BnsqA%3D&reserved=0

2. Nachtigall P G, Durham A M, Rokyta D R, et al. ToxCodAn-Genome: an automated pipeline for toxin-gene annotation in genome assembly of venomous lineages[J]. Gigascience, 2024, 13: giad116.
https://eur01.safelinks.protection.outlook.com/?url=https%3A%2F%2Fdoi.org%2F10.1093%2Fgigascience%2Fgiad116&data=05%7C02%7Cpaul.wilmes%40uni.lu%7C7db2d2e5b64f482096f308de9486c817%7C445a9c950f9d49539db1bc4a45dd1220%7C0%7C0%7C639111503822028671%7CUnknown%7CTWFpbGZsb3d8eyJFbXB0eU1hcGkiOnRydWUsIlYiOiIwLjAuMDAwMCIsIlAiOiJXaW4zMiIsIkFOIjoiTWFpbCIsIldUIjoyfQ%3D%3D%7C0%7C%7C%7C&sdata=oprnTmJkrIEzP3z%2Bgm7rmxt3v7iod9BlGhwqm

	O9G0SA%3D&reserved=0<br><br>3. Roach M J, Beecroft S J, Mihindukulasuriya K A, et al. Hecatomb: an integrated software platform for viral metagenomics[J]. GigaScience, 2024, 13: giae020.<br>https://eur01.safelinks.protection.outlook.com/?url=https%3A%2F%2Fdoi.org%2F10.1093%2Fgigascience%2Fgiae020&data=05%7C02%7Cpaul.wilmes%40uni.lu%7C7db2d2e5b64f482096f308de9486c817%7C445a9c950f9d49539db1bc4a45dd1220%7C0%7C0%7C639111503822063704%7CUnknown%7CTWFpbGZsb3d8eyJFbXB0eU1hcGkiOnRydWUsIlYiOiIwLjAuMDAwMCIsIlAiOiJXaW4zMiIsIkFOIjoiTWFpbCIsIldUIjoyfQ%3D%3D%7C0%7C%7C%7C&sdata=At4N2T7tySZC1zzhpHAOn%2BMQLkEbZF%2Ftt1qDODGRpNU%3D&reserved=0<br><br>--<br>Please also take a moment to check our website at<br>https://eur01.safelinks.protection.outlook.com/?url=https%3A%2F%2Fwww.editorialmanager.com%2Fgiga%2Fl.asp%3Fi%3D243133%26l%3D0MN2ZJHN&data=05%7C02%7Cpaul.wilmes%40uni.lu%7C7db2d2e5b64f482096f308de9486c817%7C445a9c950f9d49539db1bc4a45dd1220%7C0%7C0%7C639111503822100221%7CUnknown%7CTWFpbGZsb3d8eyJFbXB0eU1hcGkiOnRydWUsIlYiOiIwLjAuMDAwMCIsIlAiOiJXaW4zMiIsIkFOIjoiTWFpbCIsIldUIjoyfQ%3D%3D%7C0%7C%7C%7C&sdata=LNZe%2FQUjXNSrpawcB%2B91rwZt7mpXYoCMx7prfYfQgSI%3D&reserved=0<br><br>for any additional comments that were saved as attachments.<br><br>R/ We would like to thank the editor for the helpful suggestion. The relevant paper has now been included in the introduction (lines 109 and 117):<br><br>"gSpreadComp (https://doi.org/10.1093/gigascience/giaf072) recently proposed a workflow that integrates comparative genomics, plasmid-mediated transfer assessment, and resistance-virulence risk-ranking to facilitate hypothesis generation for targeted experimental validation by identifying concerning resistant hotspots in complex microbial datasets. However, while gSpreadComp is valuable for comparative, community-level prioritisation, ARGs are predicted using only one tool, DeepARG, and VF are annotated using databases and alignment-based approaches rather than predictive models and machine learning frameworks. In addition, it lacks a signal peptide or a BGC annotation framework needed for comprehensive microbiome-based pathogenicity profiling, and the pipeline accepts only DNA sequences as input"<br><br>We also thank you for suggesting additional references. However, papers 2 and 3 were not incorporated because they do not align with this study's scope. Paper 2 examines venomous snakes, while our research concentrates on predicting toxins and related proteins in prokaryotes, viruses, and microeukaryotes. Paper 3 deals with virome characterisation at both the read and contig levels, which is also beyond the scope of this study.
**Additional Information:**	
**Question**	**Response**
Are you submitting this manuscript to a special series or article collection?	No
**Experimental design and statistics**<br><br>Full details of the experimental design and statistical methods used should be given in the Methods section, as detailed in our Minimum Standards Reporting Checklist. Information essential to interpreting the data presented should be made available in the figure legends.	Yes

Have you included all the information requested in your manuscript?	
**Resources**<br><br>A description of all resources used, including antibodies, cell lines, animals and software tools, with enough information to allow them to be uniquely identified, should be included in the Methods section. Authors are strongly encouraged to cite Research Resource Identifiers (RRIDs) for antibodies, model organisms and tools, where possible.<br><br>Have you included the information requested as detailed in our Minimum Standards Reporting Checklist?	Yes
**Availability of data and materials**<br><br>All datasets and code on which the conclusions of the paper rely must be either included in your submission or deposited in publicly available repositories (where available and ethically appropriate), referencing such data using a unique identifier in the references and in the “Availability of Data and Materials” section of your manuscript.<br><br>Have you have met the above requirement as detailed in our Minimum Standards Reporting Checklist?	Yes
GigaScience has policies and guidelines in place for the use of generative AI-writing tools such as ChatGPT. If you have used such writing tools to assist with writing the manuscript this must be declared and cited in the text. Authors should not list AI-writing tools and other AI-assisted technologies as an author or co-author and should acknowledge that they are fully responsible for text generated or refined by AI-writing tools.<p>	No

A summary of use (particularly in the introduction or among methods) needs to be included at the end of the paper, and the outputs should also be included as a supplementary file hosted in GigaDB or other open repositories. Please read our guidelines for more information. <p><br><br>By submitting to GigaScience, you are aware of the journal's AI-writing tools policy, and if you have declared use of such tools below, you have acknowledged this where appropriate in your manuscript and have made a summary of use and outputs available. <p><br><b>AI-assisted writing tools have been used in the preparation of this manuscript?	

Powered by Editorial Manager® and ProduXion Manager® from Aries Systems Corporation

Manuscript

Click here to access/download;Manuscript;PathoFact2_resubmission_revw2

PathoFact 2.0: An Integrative Pipeline for the Prediction of Antimicrobial Resistance Genes, Virulence Factors, Toxins and Toxin-associated Proteins, and Biosynthetic Gene Clusters in Metagenomes

Luis F. Delgado (luis.delgado@uni.lu)*[1][0000-0001-7850-5285], Júlia Ortís Sunyer (julia.ortissunyer@uni.lu)*[1][0000-0002-2714-7067], Cedric C. Laczny (cedric.laczny@uni.lu)[1][0000-0002-1100-1282], Oskar Hickl (oskar.hickl@lih.lu)[1][0000-0001-9959-8767], Patrick May (patrick.may@uni.lu)[1][0000-0001-8698-3770] & Paul Wilmes (paul.wilmes@uni.lu)[1,2][0000-0002-6478-2924]

1. Luxembourg Centre for Systems Biomedicine, University of Luxembourg, Esch-sur-Alzette, Luxembourg
2. Department of Life Sciences and Medicine, Faculty of Science, Technology and Medicine, University of Luxembourg, Esch-sur-Alzette, Luxembourg

Contact: Paul Wilmes (paul.wilmes@uni.lu)

*These authors contributed equally

Abstract

Background

Antimicrobial resistance genes (ARG) and virulence factors (VFs) are central contributors to the global health crisis surrounding drug-resistant infections.

Findings

We introduce PathoFact 2.0, an enhanced pipeline for improved ARG, VF, toxin, and biosynthetic gene clusters (BGC) prediction. Key improvements include an updated machine learning (ML) model for VF identification, expanded hidden Markov model profiles for VFs and toxin-associated proteins, a new ML model for toxin and toxin-associated proteins identification, and the integration of antiSMASH 7.0 for predicting biosynthetic gene clusters.

Conclusions

Our upgrades make PathoFact 2.0 a more powerful and user-friendly platform for predicting microbiome-based pathogenicity and resistance, providing a crucial tool for better understanding and addressing the challenges posed by antimicrobial resistance and infectious diseases.

PathoFact 2.0 is available at https://gitlab.com/uniluxembourg/lcsb/systems-ecology/pathofact2. It is compatible with Linux operating systems.

Keywords

Antimicrobial resistance genes, virulence factors, toxin-associated proteins, biosynthetic gene clusters, metagenomes, machine learning

Findings

Introduction

Microbiomes are highly complex and diverse ecological communities composed of bacteria, archaea, viruses, and microeukaryotes. These communities include both commensal microorganisms, which can contribute to host health, and pathogenic or opportunistic microorganisms that can cause disease under specific conditions. Microbial communities generally exist in synergistic relationships with their hosts, playing critical roles in maintaining physiological homeostasis and regulating immune function. However, disruption of this balanced microbial ecosystem, known as microbial dysbiosis, can impair normal body functions and has been associated with the development of various diseases, including cardiovascular diseases, cancers, and respiratory disorders [1].

Moreover, these microorganisms play a critical role in the development of antibiotic-resistant infections through the presence of antimicrobial resistance genes (ARGs) and virulence factors (VFs)[2,3]. ARGs are genetic elements that confer bacterial resistance to antibiotics. Many ARGs are encoded on mobile genetic elements (MGEs) and are therefore often horizontally transmitted [4]. ARGs can be divided into categories based on the antibiotics to which they confer resistance [5]. The Antibiotic Resistance Ontology (ARO) contains information on ARGs, the mutations that cause them, their products, mechanisms, associated phenotypes, antibiotics, and molecular targets [6].

Bacterial pathogens use specific genes, known as VFs, to attach to and invade host tissues, survive within the host, spread, and ultimately cause damage. The harm inflicted can vary, ranging from minor disruptions to severe or even fatal outcomes [7]. VFs can be classified as secretory, membrane-associated, or cytosolic. Cytosolic virulence factors promote rapid adaptive shifts in bacterial metabolism, physiology, and morphology, enhancing survival and proliferation within the host. Membrane-associated factors contribute to bacterial adhesion and immune evasion at the host-cell interface. Secretory factors constitute a critical part of the bacterial armamentarium, enabling bacteria to counteract innate and adaptive immune defences. Secretory VFs often exhibit synergistic effects and induce cytotoxicity in host cells [8]. VFs are often located on MGEs, such as transposons, plasmids and phages, facilitating their transfer between bacterial cells [9,10].

Bacterial toxins play a crucial role in the development of infectious diseases, alongside various virulence factors employed by pathogens. They disrupt host processes and manipulate immune responses. Some toxins impair protein synthesis, destroy blood cells, or affect the nervous system. Bacterial toxins can be divided into two main categories: cell-associated endotoxins and extracellular, diffusible exotoxins. Endotoxins, such as lipopolysaccharides, are found in the outer membranes of Gram-negative bacteria and serve as potent inflammatory mediators that can induce systemic toxicity and septic shock in infected hosts [11]. Exotoxins, which are typically polypeptides and proteins, can stimulate a range of host responses by either acting directly on cell receptors or through enzymatic modulation [12,13]. Many bacterial toxins are secreted proteins that require signal peptides. Signal peptides are short amino acid sequences at the N-terminus of proteins that direct them to specific cellular compartments, such as the periplasm [14,15].

Biosynthetic gene clusters (BGCs) are responsible for synthesising specialised metabolites (SMs). Some SMs can increase pathogenicity; for example, clinical isolates of *Pseudomonas aeruginosa* produce siderophores, rhamnolipids, quinolones, and phenazines [16]. Similarly, *Burkholderia* strains produce virulence factors, such as toxoflavin from *Burkholderia glumae* [17]. Notably, pyocyanin, a redox-active phenazine produced by *Pseudomonas aeruginosa*, plays a crucial role as a virulence factor in lung infections [18].

The threat that ARGs, VFs and toxins pose to human health is significant. The United Nations have identified antimicrobial resistance as a global threat, with estimates attributing 1.27 million deaths annually to drug-resistant infections, potentially rising to 10 million by 2050 if unaddressed [19,20]. Thus, accurately predicting potential ARG and VF profiles is essential for early intervention, enabling anticipation of infection severity, improving treatment strategies, and ultimately reducing mortality rates from disease-causing pathogens.

Predicting and annotating ARGs, VFs, and toxins is challenging due to limited well-annotated data [21] and complex mechanisms involving gene transfer, mutations, and multifactorial interactions. Traditional annotation methods, which rely on sequence similarity, may overlook novel ARGs, VFs and toxins. In contrast, machine learning offers robust solutions through pattern recognition, enabling accurate predictions even with limited training data.

An integrated bioinformatics pipeline enhances analysis by simultaneously examining ARGs, VFs, toxins, signal peptides, and BGCs from a single metagenomic sample. This comprehensive approach provides a more complete view of bacterial pathogenicity by capturing the full spectrum of virulence mechanisms, including antimicrobial resistance, toxin production, and secondary metabolic capabilities. This holistic analysis improves insights into pathogenicity and resistance, streamlines workflows, and simplifies data interpretation.

PathoFact 1.0, a pipeline first introduced in 2020, integrates ARG, VF, and bacterial toxin prediction from metagenomic data into a single tool [22]. Since the publication of PathoFact, several tools have been implemented to predict ARGs, VFs, and bacterial toxins [23–25]. HyperVR [24] has attempted to predict them simultaneously, analogous to PathoFact. However, HyperVR's repository is no longer available online, and the Zenodo archive from its original submission lacks the necessary databases, hence rendering it unusable. gSpreadComp [26] recently proposed a workflow that integrates comparative genomics, plasmid-mediated transfer assessment, and resistance-virulence risk-ranking to facilitate hypothesis generation for targeted experimental validation by identifying concerning resistant hotspots in complex microbial datasets. However, while gSpreadComp is valuable for comparative, community-level prioritisation, ARGs are predicted using only one tool, DeepARG, and VF are annotated using databases and alignment-based approaches rather than predictive models and machine learning frameworks. In addition, it lacks a signal peptide or a BGC annotation framework needed for comprehensive microbiome-based pathogenicity profiling, and the pipeline accepts only DNA sequences as input.

Here, we present PathoFact 2.0 (Figure 1). It enhances the previous version by supporting protein sequences or contigs as input and by updating the ML VF model and the hidden Markov model (HMM) profiles of the conserved domain databases (CDD) [27] for VF and toxin-associated protein annotation. We have also introduced the ability to predict BGCs using antiSMASH 7.0 [28]. antiSMASH is a tool that identifies, annotates, and analyses secondary metabolite BGCs across genomes.

Figure 1. Schematic representation of PathoFact 2.0. Solid lines denote core modules, while dotted lines indicate optional user selection. The input is a FASTA file with either contig or protein sequences. If the input is a FASTA file containing contigs, open reading frames (ORFs) are predicted using Pyrodigal-gv. If the biosynthetic gene cluster (BGC) option is selected, antiSMASH will use the GBK file for BGC prediction. GeNomad is used for MGE and phage prediction, producing a FASTA file of protein sequences. Protein sequences are dereplicated using MMseqs2 to retain non-redundant sequences (based on 100% identity and coverage). After dereplication, antimicrobial resistance genes (ARGs), virulence factors (VFs) and toxins and their associated proteins are predicted using their respective modules. SignalP predicts the presence of signal peptides and their cleavage sites in proteins from

archaea, bacteria and eukarya. Individual reports are generated for each module, and an integrated report is produced that combines all module reports.

Pipeline Structure

Unlike version 1.0, which supports only contigs, PathoFact 2.0 accepts nucleotide sequences of contigs and protein sequence FASTA files, with proteins dereplicated by our tool to retain only non-redundant sequences (based on 100% identity and coverage). For contig-based inputs, open reading frames are predicted using Pyrodigal-gv (version 0.3.2; [29,30]; https://github.com/althonos/pyrodigal-gv), a Python library that binds to Prodigal [22], followed by the detection of MGEs and phages using geNomad (version 1.8.0; [30]). GeNomad processes only nucleotide sequences; therefore, MGEs and phages are not detected in protein sequence inputs. Based on user configuration, the pipeline then analyses the processed sequences using the ARG, VF, toxin-associated, and BGC (via antiSMASH) prediction modules. The information is compiled into individual module reports and an integrated report, also incorporating details from SignalP and geNomad (Figure 1). Additionally, PathoFact 2.0 generates a FASTA file of proteins identified as ARGs, VFs, or toxin-associated proteins.

Pipeline Installation

PathoFact 2.0 is implemented using Snakemake (version 7.25.0; [31]). An installation script simplifies the setup by installing the required software and downloading databases with a single command. PathoFact 2.0 is open-source (GNU General License v3.0 or later) and freely available at https://gitlab.com/uniluxembourg/lcsb/systems-ecology/pathofact2, where detailed instructions for pipeline installation, configuration, and output are provided.

Pipeline Updates

We implemented thorough updates across all PathoFact modules. Notably, we developed two new machine-learning models: one to predict virulence factors and another to identify toxin-associated proteins. A schematic diagram of the construction of the training and test datasets is shown in Figure 2. In the sections below, we detail the updates to each module.

Figure 2. Schematic representation of the datasets used for PathoFact 2.0 toxin-associated and virulence factors modules training and testing. Shades of green represent the toxin-associated datasets; shades of blue represent the non-pathogenic dataset; and shades of purple represent the virulence factor datasets.

Generalities about the "non-pathogenic" dataset and the machine learning training setup

The "non-pathogenic" dataset for the ML models was constructed by selecting SwissProt [32] sequences lacking ARG, VF, and toxin keywords [KW-0568 (pathogenesis-related protein), KW-0843 (virulence), KW-0800 (toxin), KW-0046 (antibiotic resistance), KW-9995 (disease)] and limited to bacteria (taxonomy_id 2), archaea (taxonomy_id 2157), fungi (taxonomy_id 4751), and viruses (taxonomy_id 10239) (Figure 2). Additionally, proteins from non-pathogenic organisms to humans (Supplementary Table S1; Figure 2) were included from NCBI. MMseqs2 (version 15.6f452; [33]) was used to obtain a set of non-redundant (clustered at 100% identity and coverage) protein sequences (Figure 2).

ML models were trained using 80% of the sequences for training and 20% of the sequences for testing. The Synthetic Minority Oversampling Technique (SMOTE) was employed to address the dataset's

imbalance [34]. Using the XGBoost Python package (https://xgboost.readthedocs.io/en/stable/index.html) and the RandomForest (RF) Scikit-learn (version 1.5.2; [35]), several ML models were trained and tested using k-mers (k=3 to 8) or protein sequence composition features (amino acid composition (AAC), dipeptide composition (DPC), composition (CTDC), transition (CTDT), and distribution (CTDD) [36]) as features. Hyperparameter optimisation was performed, using a 5-fold cross-validation with HalvingGridSearchCV from scikit-learn [35]. The best-performing model was selected based on the Matthews correlation coefficient (MCC) score.

Toxin-Associated Protein Prediction Updates

Compared to version 1.0, the toxin prediction module now employs a ML model instead of a purely alignment-based bit score threshold. Curated training data was obtained from SwissProt [32], filtered for bacterial (taxonomy_id 2), archaeal (taxonomy_id 2157), fungal (taxonomy_id 4751) and viral (taxonomy_id 10239) toxin sequences (KW-0800, toxin)(Figure 2). The dataset was supplemented with entries from toxin-specific databases such as the Toxin Exposome Database (T3DB) [12], which catalogues bacterial protein toxins; the Database for Bacterial ExoToxins (DBETH) [13]; TADB version 3.0, which includes protein sequences of bacterial toxin–antitoxin (TA) pairs from types I to VIII [37]; sequences from SecReT6 [38], encompassing T6SS gene cluster components, T6SS regulator (T6SR), accessory proteins (T6SA), effectors (T6SE), and immunity proteins (T6SI); and the prokaryotic antimicrobial toxins (PAT) database [39] (Figure 2).

MMseqs2 (version 15.6f452; [33]) was used to dereplicate the dataset of 1,112,357 protein sequences (100% identity and coverage), yielding 213,363 unique protein sequences, corresponding to the “toxin-associated” dataset (Figure 2). It is essential to note that this dataset encompasses both effector toxin proteins and their associated proteins, including antitoxins, regulators, and accessory proteins. This offers three main benefits: 1) Recent reports suggest that the same bacterial toxins can function as part of self-inhibiting toxin-antitoxin modules within one organism, while in another organism, they have evolved into toxin effectors that are injected into target cells [40,41]. 2) In bacteria, genes located in close proximity frequently exhibit functional associations, such as those co-transcribed within operons. A comprehensive toxin dataset, including both toxins and their associated proteins, facilitates the identification of novel toxins and related genes through their genomic context, referred to as "toxin islands." These islands may be involved in toxin biosynthesis, processing, or secretion, and may also confer immunity or facilitate horizontal gene transfer among bacterial populations. Notably, they are often rich in mobile genetic elements [42]. 3) A large database improves the performance of machine learning classification methods [43].

HMM profiles were built using the conserved-domain FASTA files (https://ftp.ncbi.nih.gov/pub/mmdb/cdd/fasta.tar.gz) from CDD [27]. The 213,363 unique protein sequences in the “toxin-associated” dataset were annotated using the CDD HMM profiles. Those with a bitscore above 25 were chosen as HMM profiles for toxin and toxin-associated protein annotation and incorporated into Pathofact 2.0 for protein annotation.

Although there is no standard for creating negative datasets, they play a crucial role in influencing model performance. Therefore, to improve the quality of our “non-toxin” dataset, potential ARGs, VFs (with high probability), and toxins were filtered out of the “non-pathogenic” dataset using PathoFact 1.0 predictions. The final “non-toxin” dataset consists of 213,129 non-redundant protein sequences (Figure 2).

The toxin-associated ML model is a RF with 5-mer features (default hyperparameter setting, i.e, number of trees in the forest [n_estimators]=100, maximum depth of the tree [max_depth]=None, minimum number of samples required to split an internal node [min_samples_split]=2).

The toxin-associated protein prediction module generates a report containing the proteinID, protein domains, bitscore, toxin-associated ML probability, other identical proteins found in the sample, and optionally SignalP, plasmid marker, and virus marker information.

VF Prediction Updates

The VF prediction model was refined and updated with new HMM profiles. Training data was derived from SwissProt [32], selecting sequences annotated with the virulence keyword (KW-0843) and expanded using the Virulence Factor Database (VFDB; [44]) (Figure 2). After dereplication (with 100% identity and coverage), the original set of 32,511 sequences, using MMseqs2, comprised 30,695 non-redundant sequences, corresponding to the "VF dataset" (Figure 2). We searched the "VF dataset" against the CDD HMM profiles, selecting those with a bit score of 25 or higher as VF HMM profiles for PathoFact 2.0. The HMM profile dataset annotates the predicted VF domains rather than using them as input to the classification, as in the previous version.

To create the "non-VF" dataset for the ML VF model, we filtered out any potential VFs (with high and low probabilities), ARGs, and toxins based on PathoFact 1.0 predictions from the "non-pathogenic" dataset. This resulted in a dataset of 41,774 VF protein sequences (Figure 2).

The VF model uses XGBoost with protein sequence composition features (Step size shrinkage used in update to prevent overfitting [learning_rate]= 0.1, number of trees 'n_estimators]= 2000). The VF module generates a report containing the proteinID, protein domains, bitscore, virulence factor ML probability, other identical proteins found in the sample, and optionally SignalP, plasmid marker, and virus marker information.

ARG Prediction Updates

ARG prediction in PathoFact 2.0 integrates DeepARG (version 1.0.2; [45]), RGI (version 6; [6]), and AMRFinderPlus (version 3.12.8; [46]). DeepARG and RGI have received updates from their developers since the release of PathoFact 1.0, which have been incorporated into PathoFact 2.0. In addition, AMRFinderPlus has been newly integrated into PathoFact 2.0. Each tool has distinct strengths: DeepARG offers high precision and recall; RGI provides robust predictions based on an extensive database, utilising homology and single-nucleotide polymorphism (SNP) models; and AMRFinderPlus efficiently identifies resistance genes and mutations using NCBI resources.

The ARG prediction module (Figure 1) report includes protein IDs, ARG classes, prediction probabilities, database accession numbers, and optional data on signal peptides, plasmids, and virus markers. PathoFact 2.0 uses argNorm [47] to map detected genes to the ARO, thereby facilitating comparison of ARG annotation outputs by ensuring standardised and comparable results. Supplementary Figures S1A and S2A compare the performance of PathoFact 2.0 with that of its predecessor, PathoFact, in identifying ARGs.

Additional Functionalities

PathoFact 2.0 integrates SignalP (version 6; [24]) and antiSMASH (version 7.0; [28]), both of which are optional features that accommodate diverse research needs. SignalP is designed to predict the presence and location of signal peptides in protein sequences. It requires a separate license and must be requested by the user individually. AntiSMASH is designed to identify and annotate BGCs in bacterial and fungal genomes. Since AntiSMASH is a resource-intensive tool, we set it up as an optional module and provide the option to run it in chunks.

Evaluation of the performance of the PathoFact 2.0 pipeline

We evaluated the performance of PathoFact 2.0 and the new toxin-associated and VF modules using the test datasets described above. We did not include ARGs and BGCs in the validation step, as the respective modules are based on existing tools that have already demonstrated high accuracy [6,28,45,46]. Figure 3 provides a schematic overview of the benchmarking datasets used for the toxin-associated and VF modules.

Figure 3. Schematic representation of the construction of the datasets used for benchmarking. A) Toxin benchmarking dataset. B) Virulence factor benchmarking dataset.

Virulence factors and toxin-associated protein prediction

The VF and toxin-associated modules (Figure 1) were evaluated across different ML models-predicted probability thresholds, corresponding to the model's confidence in the positive class (VF or toxin-associated). Analyses were performed on the full test dataset and on subsets of the test dataset. These subset datasets were created based on sequence similarity to the training dataset, with a range of 40% to 100% similarity and 80% coverage (Figure 2). This approach aimed to assess prediction accuracy on proteins in the testing dataset with low similarity to the training dataset, specifically including only sequences with less than 40-100% identity to any training sequence. The performance evaluation is based on the Matthew correlation coefficient (MCC) and the precision (to reduce the number of false positive VF and toxin-associated predictions), taking into account the dataset imbalance (a higher number of “non-toxin” and “non-VF” sequences compared to “toxin-associated” and “VF” sequences in the test subsets). The MCC is a more reliable statistical measure that yields a high score only when the prediction performs well across all four categories of the confusion matrix (true positives, false negatives, true negatives, and false positives), and it is proportional to both the number of positive and negative elements in the dataset [48]. We found that predicted probabilities of 0.6 for toxin-associated proteins and 0.9 for VFs provide a good balance between high MCC and precision across different test subsets (Figure 4 and Supplementary Tables S3 and S4).

Figure 4. Performance evaluation of toxin-associated and virulence factor prediction modules across probability thresholds. A) Toxin-associated prediction module evaluation. **B)** Virulence factors prediction module evaluation. The modules were evaluated across a range of predicted class probabilities (0.5-0.9). The entire test dataset (All) and subsets of the test datasets were used for evaluation. These subset datasets were created based on sequence similarity to the training dataset, with similarity levels of 40%, 60%, 80%, and 100%, and an 80% coverage threshold. Only sequences with similarity below these percentages were included in the respective test subsets.

Benchmarking

The Pathofact 2.0 VF prediction module was compared to VirulentHunter [49], using the default parameters. VirulentHunter is a deep learning framework that simultaneously identifies and classifies VFs directly from protein sequences, which outperforms other virulence factor predictors (MP4 [50], VirulentPred 2.0 [51], and DeepVF [52]). A notable feature of VirulentHunter is that it provides VF category classification; however, it takes about 2 minutes to analyse 500 protein sequences (using 1 GPU), which is a drawback for metagenomic sample analysis, where thousands to millions of proteins are predicted from a single sample. PathoFact 2.0 requires only 4 seconds (using 1 CPU, with the option to utilise more CPUs) to analyse 500 protein sequences (Table 1).

Table 1. Runtime comparison of PathoFact 2.0 and VirulentHunter

Number of protein sequences	VirulentHunter	PathoFact 2.0				
	1 GPU	1 CPU	2 CPU	4 CPU	6 CPU	8 CPU
500	2 min 21 s	3.7 s	2.6 s	2.0 s	1.9 s	1.8 s
5500	25 min 42 s	29.9 s	17.4 s	10.2 s	8.1 s	7.4 s
10000	54 min 11 s	55.4 s	29.3 s	16.7 s	13.1 s	11.5 s
30000	2 h 45 min 50 s	2 min 45 s	1 min 32.9 s	52.1 s	40.3 s	36.0 s

Since VirulentHunter and Pathofact 2.0 employ a similar method to generate the “VF dataset” for model training, we removed sequences from the Pathofact 2.0 test dataset that have 100% identity (≥80% coverage) to the VirulentHunter training dataset, resulting in a “clean VF test dataset” (Figure 3). This ensures that neither model used the test sequences for training. We applied the same test-subset approach described earlier: the subset datasets were created based on sequence similarity to the Pathofact 2.0 training dataset, with similarity ranging from 40% to 100% and 80% coverage of the “clean VF test dataset” (Figure 3). By stratifying test sets by decreasing sequence similarity to the training data, we explicitly evaluated model performance across progressively more divergent proteins. This study demonstrates that PathoFact 2.0 maintains consistent performance even when test sequences share 40% similarity or less with the training set (Figure 5B, Supplementary Figure S3). In addition to VirulentHunter, we benchmarked PathoFact 2.0 against the virulence factor prediction module of PathoFact 1.0 and against metaVF [23], an alignment-based toolkit (based on BLAST) that identifies species-level VFs associated with pathobionts. DNA gene sequences from the test subsets were used as input, since both tools accept only contigs.

The Pathofact 2.0 toxin-associated module was compared with ToxinPred2 [42] using the default parameters, i.e., Hybrid (RF+BLAST+MERCI) with a threshold of 0.6. The ToxinPred2 website restricts predictions to a certain number of proteins (around 2000). Since ToxinPred2 is designed to predict protein toxicity, we selected sequences from the Pathofact 2.0 “toxin-associated” test dataset that are directly linked to toxins and removed the remaining “toxin-associated” proteins (Figure 3). In short, using the header information from the Pathofact 2.0 “toxin-associated” test dataset, we kept only toxin sequences from the toxin-antitoxin sequences from the TADB, the effector factor sequence from the SecReT6 database, bacterial protein toxins from T3DB, bacterial exotoxins from DBETH, and sequences from Swissprot (KW-0800, toxin), as previously described. Additionally, we kept sequences longer than 35 amino acids and excluded protein sequences containing the non-standard amino acids ‘BJOUXZ’ as the ToxinPred2 dataset was created using these criteria [53] (Figure 3). From these, we randomly selected 1000 sequences. Then, we randomly selected 1000 sequences from the Pathofact 2.0 “non-toxin” test dataset (Figure 3). This resulted in a total of 2000 sequences for benchmarking PathoFact 2.0 against the ToxinPred2 webserver. Due to the limited number of sequences, we did not use the test-subset approach to evaluate ToxinPred2 and Pathofact 2.0 toxin-associated modules on this 2000-sequence test dataset (Figure 3). We also use the same test dataset to benchmark CSM-toxin v1.0.1, a deep learning model for toxin prediction [54], as well as the toxin prediction module from PathoFact 1.0. DNA gene sequences from the test dataset were used as input.

As shown in Figure 5 (and in the Supplementary Tables S5 and S6 and the AUROC curves in Supplementary Figures S3 and S4), Pathofact 2.0 VF and toxin-associated modules exhibited higher MCC values across different test subsets compared to VirulentHunter, ToxinPred2 and CSM-toxin. Because the negative dataset was built and then filtered using PathoFact 1.0, the precision for PathoFact 1.0 is 1,

which affects the MCC calculation. To avoid biased comparisons, we report recall only for PathoFact 1.0. Figure 5 shows that PathoFact 2.0 VF and toxin-associated modules achieved higher recall across test subsets than PathoFact 1.0, and better recall and MCC than metaVF.

Figure 5. Benchmarking of toxin and virulence factor prediction performance. A) Toxin-associated module benchmarking. The Pathofact 2.0 toxin-associated module was compared with ToxinPred2 (Hybrid: RF+BLAST+MERCI, threshold = 0.6) using its web-based version and CSM-toxin v 1.0.1. A balanced toxin test dataset (1,000 toxin and 1,000 non-toxin sequences) was built from the Pathofact 2.0 toxin-associated test dataset, selecting only sequences from curated toxin sources (TADB, SecReT6, T3DB, DBETH, SwissProt), applying ToxinPred2's filtering criteria. Several predicted probability cutoffs, 0.5, 0.6 and 0.8, of the Pathofact 2.0 toxin-associated module were evaluated. MCC, precision, and recall are shown. **B)** Virulence factors module benchmarking. The Pathofact 2.0 VF module was compared to VirulentHunter. Sequences identical (100% identity, ≥80% coverage) to VirulentHunter's training data were removed from the Pathofact 2.0 test dataset. Then, test subset datasets were created based on 40–100% similarity to the Pathofact 2.0 training set. These subset datasets were created based on sequence similarity to the training dataset, with similarity levels of 40%, 60%, 80%, and 100%, and an 80% coverage threshold. Only sequences with similarity below these percentages were included in the respective test subsets. Several predicted probability cut-offs (0.5, 0.6, 0.8, and 0.9) of the Pathofact 2.0 VF module were evaluated. MCC, precision, and recall are presented for each test subset. Since the negative dataset was built and then filtered using PathoFact 1.0, the precision is 1 for PathoFact 1.0, therefore affecting MCC calculation. To avoid biased comparisons, we report recall only for PathoFact 1.0.

Virulence factors and toxin-associated protein prediction with contig sequences as input

To evaluate PathoFact 2.0 at the contig level, we analysed publicly available complete genomes from pathogenic and non-pathogenic bacteria, including various *Escherichia coli* strains. Figure 6 shows distinct differences in virulence and toxin-related profiles between pathogenic and non-pathogenic *E. coli* strains, especially regarding virulence- and toxin-associated proteins encoded on MGEs, such as plasmids and prophages. Nonetheless, analysis of individual VF predictions reveals considerable overlap in the number of VF genes detected across both pathogenic and non-pathogenic strains. This highlights that the presence of a VF gene is not a reliable marker of pathogenicity and emphasises the importance of considering genomic and functional context when assessing virulence potential.

It is well known that VFs of pathogenic *E. coli* are often encoded on genetic elements, such as plasmids, bacteriophages, transposons, and pathogenicity islands, which can be mobilised into different strains to create novel combinations of virulence factors [55,56]. The same pattern is observed in pathogenic strains of several genera compared to non-pathogenic strains (Supplementary Figure S5), particularly for *Klebsiella pneumoniae* and *Salmonella enterica*. These findings highlight the importance of examining virulence from a systems perspective rather than focusing solely on the presence or absence of individual factors. A comprehensive assessment should consider not only whether a virulence- or toxin-associated protein is encoded within an MGE but also its functional context, such as whether it is secreted or part of a BGC.

Figure 6. Comparative analysis of toxin-associated and virulence factor profiles in non-pathogenic and pathogenic *Escherichia coli* strains. Bar charts represent the distribution of predicted toxin-associated (A) and virulence-associated (B) proteins across non-pathogenic (left panel) and pathogenic (right panel) *E. coli* strains. The categories include total predicted virulence factors/toxin-associated proteins (dark blue), those associated with plasmid markers (red), those associated with phage markers (green), and proteins predicted by SignalP to be secreted (light blue). Additional categories include toxin-associated virulence factors (TOX-associated and VF, grey) and biosynthetic gene clusters

overlapping (purple). Numerical values above each bar indicate the total count of proteins identified in each category for the corresponding strain.

The PathoFact 2.0 VF module was compared to PathoFact 1.0 and metaVF. To our knowledge, no other method is available to predict VF from contig sequences and identify plasmid-encoded or prophage-associated VF. However, we included VirulentHunter in the comparison. Because VirulentHunter does not accept contig sequences as input, protein-coding genes were first predicted from contigs using Pyrodigal-gv and the resulting protein sequences were subsequently analysed. PathoFact 2.0 consistently predicted a greater number of VFs than both PathoFact 1.0 and metaVF (Figure 7, Supplementary Figures S6–S8). Notably, metaVF failed to identify any VFs in five of the ten pathogenic reference strains tested, highlighting its limited ability to detect VFs and demonstrating the advantages of machine-learning-based models over homology-based approaches. While VirulentHunter produced substantially more hits than metaVF, yielding predictions comparable in number to PathoFact 2.0, PathoFact 2.0 generally identified more VFs overall (Figure 7, Supplementary Figures S6–S8). Exceptions were observed for non-pathogenic strains *Bifidobacterium animalis*, *Bifidobacterium bifidum*, *Heyndrickxia coagulans*, and for the pathogenic *Ralstonia mannitolilytica strain Guangzhou-RMAB10*, where VirulentHunter predicted slightly more VFs (Supplementary Figures S6 and S8).

Figure 7. Performance comparison of virulence factor prediction tools in pathogenic (A) and non-pathogenic (B) strains. Venn diagrams comparing the predictions of PathoFact 2.0, PathoFact, metaVF, and VirulentHunter in predicting virulence factors (VFs).

In addition, the PathoFact 2.0 toxin-associated module was compared to PathoFact 1.0 as well as CSM-Toxin and ToxinPred2 (Figure 8, Supplementary Figures S1B, S2B and S9). As CSM-Toxin and ToxinPred2 do not accept contig sequences as input, protein sequences were first predicted from contigs using Pyrodigal-gv. PathoFact 2.0 predicted more toxin-associated proteins than PathoFact 1.0, demonstrating improved detection capacity. Compared to external tools, CSM-Toxin identified substantially fewer toxins across the tested reference strains. In contrast, ToxinPred2 predicted a comparable number of toxins overall. However, for non-pathogenic strains *Bifidobacterium bifidum*, *Heyndrickxia coagulans*, *Lactobacillus acidophilus*, and for pathogenic strains *Streptococcus gallolyticus subsp. gallolyticus* and Streptococcus parasuis B26, ToxinPred2 identified more predicted toxins than PathoFact 2.0 (Supplementary Figures S2B and S9).

Figure 8. Performance comparison of toxin prediction tools in pathogenic (A) and non-pathogenic (B) strains. Venn diagrams comparing the predictions of PathoFact 2.0, PathoFact 1.0, CSM-Toxin, and ToxinPred2 in predicting Toxins and Toxin-associated proteins.

PathoFact 2.0 Output Structure

PathoFact 2.0 creates a structured output directory that summarises predictions from all analysis modules, including VFs, toxin-associated proteins, ARGs, MGEs and BGCs. Each module generates dedicated result files corresponding to the underlying prediction tools (Supplementary File S1).

The primary summary file, combined_report.tsv, provides an integrated overview of high-confidence predictions across all modules in a tabular format. This table includes key information such as protein identifiers, bit scores (from HMM profiles), machine-learning prediction scores, and outputs from DeepARG, RGI, SignalP, GenoMad, and antiSMASH, thereby supporting downstream interpretation and candidate prioritisation.

Proteins with prediction probabilities below user-defined thresholds but containing conserved domains identified by toxin-associated or VF HMM profiles are reported in ambiguous_TOX_hits_rep_prot.tsv and ambiguous_VF_hits_rep_prot.tsv. These lower-confidence candidates may warrant further investigation in comparative or experimental analyses.

High-confidence predictions are reported in amr_hits_rep_prot.tsv, TOX_hits_rep_prot.tsv, and VF_hits_rep_prot.tsv, which summarise features exceeding user-defined probability thresholds and include protein identifiers, bit scores, machine-learning predictions, signal peptide predictions, and genomic context information, such as association with prophages or plasmids identified by GenoMad.

In addition, PathoFact 2.0 generates a dedicated Group_of_sequence directory containing FASTA files of representative protein sequences grouped by functional category (VFs, toxin-associated proteins, antimicrobial resistance genes, and combined hits), together with conserved domain (CDD) annotation tables for predicted VFs and toxin-associated proteins. These files are designed to facilitate downstream analyses, including comparative genomics and functional characterisation.

Limitations of PathoFact 2.0

It is well established that non-pathogenic bacterial strains can also carry genes annotated as VFs or toxins [7]. Consequently, PathoFact 2.0 is most effective as an initial screening tool to identify potential candidates, which can then be examined in comparative studies to distinguish confirmed pathogenic cases from controls. The pipeline provides a probability score indicating whether a protein is likely to be VF- or toxin-associated; however, establishing a definitive link between predicted candidates and infectious disease requires experimental validation.

PathoFact 2.0 does not directly classify specific VF or toxin types (e.g., adhesins or genotoxins). Instead, it reports detailed annotations of conserved protein domains from CDD [18], allowing users to infer functional roles when available. This design emphasises contextual interpretation rather than categorical assignment.

The inclusion of housekeeping genes from non-pathogenic microorganisms reflects a deliberate methodological choice rather than a limitation. Multiple well-characterised housekeeping proteins have been shown to exhibit virulence-associated “moonlighting” functions in pathogenic bacteria, including roles in adhesion, immune modulation, and tissue invasion [57,58]. Notable examples include glyceraldehyde-3-phosphate dehydrogenase (VF0015 in VFDB) [59], enolase [60,61], elongation factor Tu (VF0460 in VFDB) [62,63], GroEL [61,64,65], and DnaK [61,66]. This approach maintains a biologically realistic negative dataset while reducing the risk of misclassifications within the intended scope of PathoFact 2.0.

PathoFact 2.0 is designed for metagenomic samples; most prediction modules and phenotypes are bacterial-centric. Virulence, toxin-associated, and antimicrobial resistance predictions are particularly interpreted in the context of human pathogens.

Conclusions

ARGs, VFs, and toxins represent major threats to global health. Therefore, accurate detection of these elements is crucial for assessing the presence and potential risks of pathogenic microorganisms in microbiomes and for identifying reservoirs of pathogenicity. Our improved pipeline, PathoFact 2.0, offers significant improvements over PathoFact (its predecessor), ToxinPred2, CSM-toxin, VirulentHunter, and metaVF. SignalP has been upgraded and made optional to further optimise performance, providing users with flexibility based on their requirements. Additionally, antiSMASH 7.0 facilitates the prediction of BGCs, recognising emerging evidence that some BGC-encoded factors might increase virulence. Furthermore, we have integrated geNomad, a cutting-edge tool for identifying MGEs, including plasmids and phages linked to ARGs, VFs, toxins and toxin-associated proteins across various bacterial species. The PathoFact 2.0 update improves the accuracy and sensitivity of analyses while enhancing computational efficiency.

PathoFact 2.0 represents a major advance in metagenomic analysis by integrating the detection of ARGs, VFs, toxins and toxin-associated proteins, signal peptides, MGEs, and BGCs within a single, streamlined pipeline. Unlike existing tools that focus on individual aspects of pathogenicity, PathoFact 2.0 provides a comprehensive, multi-layered view that captures both gene presence and genomic context, improving interpretability and enabling a holistic assessment of microbial pathogenic potential.

Methods

Databases used for the PathoFact 2.0 Dataset Construction

- SwissProt [31] is the expertly curated part of UniProtKB [67]. It offers high-quality protein sequences with detailed functional annotations, including keywords for pathogenesis, virulence, toxins, and antibiotic resistance.
- VFDB [44], the Virulence Factor Database, is a comprehensive reference for curating information on virulence factors of bacterial pathogens.
- T3DB [12], the toxin and toxin-target database, is a resource cataloguing thousands of toxins and their protein targets, with detailed mechanisms, structures, and toxicity data, including bacterial protein toxins.
- DBETH [13], the database for bacterial exotoxins, is a specialised database of bacterial exotoxins pathogenic to humans, classified into 24 mechanistic and activity types from 26 bacterial genera.
- TADB [37], the toxin-antitoxin database, is a repository of bacterial toxin-antitoxin loci across types I-VIII, including experimentally validated pairs, predicted loci, and associations with mobile genetic elements.
- SecReT6 [38] is a database containing known and predicted type VI secretion systems, including effectors, immunity proteins, regulators, and accessory proteins from bacterial genomes.
- PAT [39] is the prokaryotic antimicrobial toxin database and contains a collection of antimicrobial toxins, including bacteriocins and effectors from secretion systems.

Clustering Parameters

MMseqs2 [33] was used for dereplication; sequences were clustered at 100% identity and 100% coverage using the parameters -c 1.0 and --min-seq-id 1.0. It removes exact duplicates and retains representative sequences to generate non-redundant datasets. To create the test subsets, a coverage of 80% (-c 0.8) and identities ranging from 40 - 100% (--min-seq-id 0.4, --min-seq-id 0.6, --min-seq-id 0.8, --min-seq-id 1.0) were used, and then sequences with similarity higher than min-seq-id were removed for each test subset. In all cases, the cluster mode and coverage mode used were 0 (--cov-mode 0 --cluster-mode 0). When --cov-mode 0 is specified in combination with `-c` values ranging from 0.0 to 1.0, sequences are assigned to the same cluster only if the alignment spans at least a fraction *c* of the length of the longer sequence. According to the developers of MMSeq2, this coverage criterion is particularly suitable for clustering full-length protein sequences [33].

Protein Composition Features

Protein sequence composition features were extracted to represent each protein as fixed-length vectors derived from its primary amino acid sequence [36]. These included amino acid composition

(AAC), dipeptide composition (DPC), composition (CTDC), transition (CTDT) and distribution (CTDD). AAC captures the relative frequency of each of the 20 amino acids in a sequence, whereas DPC captures the relative frequency of all adjacent amino-acid pairs (400 possible dipeptides). CTDC represents the percentage of amino acids belonging to each of three predefined groups (polar, neutral, hydrophobic) in the entire protein sequence. CTDT represents the percentage frequency with which a residue of one group is followed by a residue of a different group along the sequence. CTDD represents the distribution of each amino acid group, measuring the spatial position, where the first, 25%, 50%, 75% and 100% of the residues of a specific class are located.

Performance and Evaluation Metrics

To assess the models' performance, we used a confusion matrix comprising true positives (TP), true negatives (TN), false positives (FP), and false negatives (FN), computed on the test datasets described above. TP corresponds to truly positive instances correctly predicted as positive by the model, whereas TN corresponds to truly negative instances correctly predicted as negative. FP are truly negative instances incorrectly predicted as positive, and FN are truly positive instances incorrectly predicted as negative. From these values, we calculated the following metrics:

$$Accuracy = (TP + TN)/(TP + TN + FP + FN)$$

$$Precision = TP/(TP + FP)$$

$$Recall = TP/(TP + FN)$$

$$MCC = (TP \times TN - FP \times FN)/\sqrt{(TP + FP)(TP + FN)(TN + FP)(TN + FN)}$$

HMM Profiles

Profile hidden Markov models are probabilistic models built from a multiple sequence alignment that encode, for each alignment position, the position-specific probabilities of residues and insertions/deletions, turning the alignment into a position-specific scoring system for detecting homologous sequences [68]. The FASTA files of conserved-domain multiple sequence alignments for each CDD [26] family (https://ftp.ncbi.nih.gov/pub/mmdb/cdd/fasta.tar.gz) were downloaded. Pyhmmer v0.10.14 [69] was used to obtain HMM profiles for each CDD family and to perform protein sequence searches against the CDD HMM family profiles.

Benchmarking Datasets

This study utilised publicly available datasets containing complete genomes from pathogenic and non-pathogenic bacteria, including various *Escherichia coli* strains, from NCBI. The accession numbers of the bacteria used are indicated in Supplementary Table S2.

Availability of Supporting Source Code and Requirements

Project name: Pathofact 2.0
Project homepage: https://gitlab.com/uniluxembourg/lcsb/systems-ecology/pathofact2
Operating system(s): Linux

Programming language: Python, R, bash
Other requirements: Snakemake, Mamba, conda.
License: GNU General License v3.0 or later
Biotools ID: pathofact2
RRID: SCR_027650
workflowhubDOI: 10.48546/workflowhub.workflow.2087.1

Additional Files

Supplementary Table S1. List of microorganisms non-pathogenic to humans and their total protein count obtained from the NCBI Database.

Supplementary Table S2. Bacterial strains used in this study, including their classification as pathogenic or non-pathogenic, species/strain information, genome assembly or reference version, and corresponding accession numbers.

Supplementary Table S3. Evaluation of the PathoFact 2.0 toxin-associated protein prediction module. The table presents performance across test subsets defined by sequence similarity to the training set. Metrics reported include class distributions (Negative, non-toxin; Positive, toxin-associated), confusion matrix counts (true negatives, false positives, true positives, false negatives), and performance measures (accuracy, precision, recall, F1 score, Matthews correlation coefficient).

Supplementary Table S4. Evaluation of the PathoFact 2.0 virulence factor prediction module. The table summarises performance across test subsets defined by sequence similarity to the training set. Reported metrics include class distributions (Negative, non-VF; Positive, VF), confusion matrix counts, and performance measures (accuracy, precision, recall, F1 score, Matthews correlation coefficient).

Supplementary Table S5. Comparison of virulence factor prediction performance for PathoFact 2.0 at varying probability cutoffs and for VirulenHunter, evaluated across test subsets stratified by sequence similarity to the training set. The table reports class distributions, confusion matrix counts, and performance metrics (accuracy, precision, recall, F1 score, Matthews correlation coefficient).

Supplementary Table S6. Comparison of toxin protein prediction performance for PathoFact 2.0 at different prediction probability cutoffs and for ToxinPred2. The table reports the number of proteins classified as Negative (non-toxin) and Positive (toxin), confusion matrix counts, and associated performance metrics (accuracy, precision, recall, F1 score, and Matthews correlation coefficient).

Supplementary Figure S1. Performance comparison of PathoFact 2.0 versus PathoFact for ARG and toxin prediction in Escherichia coli. A) Comparative performance of PathoFact 2.0 (blue) versus PathoFact (green) in predicting antimicrobial resistance genes (ARGs) in non-pathogenic (left) and pathogenic (right) *E. coli* strains. **B)** Comparative performance of PathoFact 2.0 (blue) versus PathoFact (green) in predicting toxin-associated proteins in non-pathogenic (left) and pathogenic (right) *E. coli* strains. The top panel shows the total number of predicted toxin-associated proteins, while the bottom panel shows those that contain signal peptides, as identified by SignalP.

Supplementary Figure S2. Performance comparison of PathoFact 2.0 versus PathoFact for ARG and toxin prediction in bacterial strains. A) Comparative performance of PathoFact 2.0 (blue) versus PathoFact (green) in predicting antimicrobial resistance genes (ARGs) in non-pathogenic (left) and pathogenic (right) bacterial strains. **B)** Comparative performance of PathoFact 2.0 (blue) versus PathoFact (green) in predicting toxin-associated proteins in non-pathogenic (left) and pathogenic (right) bacterial strains. The top panel shows the total number of predicted toxin-associated proteins, while the bottom panel shows those that contain signal peptides, as identified by SignalP.

Supplementary Figure S3. Area under the receiver operating characteristic (AUROC) curves comparing the virulence factor prediction performance of PathoFact 2.0 (A) and Virulent Hunter (B) on the benchmark datasets. Curves represent performance across different benchmark subsets: 40 (red), 60 (yellow), 80 (green), 100 (blue) and the complete dataset (All, purple). AUROC curves are not available with PathoFact 1.0 because it uses a combination of machine learning and HMM classification. MetaVF is an alignment-based algorithm, and it does not generate probability scores; therefore, AUROC curves are not possible.

Supplementary Figure S4. Area under the receiver operating characteristic (AUROC) curves comparing the virulence factor prediction performance of PathoFact 2.0 (A) and CSM-toxin (B) on the benchmark dataset. Curves correspond to PathoFact 2.0 (blue) and CSM-toxin (red). AUROC curves are not possible with Toxinpred2, since its predictions are based on a combination of factors rather than on machine-learning probabilities alone. Nor are they possible with PathoFact 1.0, which also uses a combination of machine learning and HMM classification.

Supplementary Figure S5. Comparative analysis of virulence factor profiles in non-pathogenic and pathogenic bacterial strains. Bar charts represent the distribution of predicted toxin-associated (A) and virulence-associated (B) proteins across non-pathogenic (left panel) and pathogenic (right panel) strains. The categories include total predicted virulence factors/toxin-associated proteins (dark blue), associated with plasmid markers (red), phage markers (Green), and proteins predicted by SignalP to be secreted (light blue). Additional categories include toxin-associated virulence factors (TOX-related ∩ VF, grey) and biosynthetic gene clusters overlapping (purple). Numerical values above each bar indicate the total count of proteins identified in each category for the corresponding strain.

Supplementary Figure S6. Venn diagrams comparing the predictions of PathoFact 2.0, PathoFact, metaVF, and VirulentHunter in predicting virulence factors in pathogenic (A) and non-pathogenic (B) strains.

Supplementary Figure S7. Comparative performance of Pathofact 2.0 (blue) versus PathoFact1 (green) and metaVF (red) in predicting virulence factors (VFs) in non-pathogenic (left) and pathogenic (right) *Escherichia coli* strains. The top panel shows the total number of predicted VFs. The second panel depicts the subset predicted to be secreted. The third panel shows VFs predicted to be plasmid-encoded, while the fourth panel presents those predicted to be prophage-associated.

Supplementary Figure S8. Comparative performance of Pathofact 2.0 (blue) versus PathoFact (green) and metaVF (red) in predicting virulence factors (VFs) in non-pathogenic (left) and pathogenic (right) bacterial strains. The top panel shows the total number of predicted VFs. The second panel depicts the subset predicted to be secreted. The third panel shows VFs predicted to be plasmid-encoded, while the fourth panel presents those predicted to be prophage-associated.

Supplementary Figure S9. Venn diagrams comparing the predictions of PathoFact 2.0, PathoFact, Toxinpred2, and CSM-Toxin in predicting toxins and toxin-associated proteins in pathogenic (A) and non-pathogenic (B) strains.

Abbreviations

AAC, amino acid composition; ARO, antibiotic resistance ontology; ARGs, antimicrobial resistance genes; BGCs, biosynthetic gene clusters; CDD, conserved domains database; CTDC, (Composition, Transition, Distribution)-composition; CTDD, (Composition, Transition, Distribution)-distribution; CTDT, (Composition, Transition, Distribution)-transition; DBETH, database for bacterial exotoxins; DPC,

dipeptide composition; HMMs, hidden Markov models; MCC, Matthews correlation coefficient; MGEs, mobile genetic elements; ML, machine learning; ORF, open reading frame; PAT, prokaryotic antimicrobial toxins database; RF, random forest; SM, specialised metabolites; SMOTE, synthetic minority oversampling technique; SNP, single nucleotide polymorphisms; T3DB, toxin exposome database; VFDB, virulence factor database; VFs, virulence factors.

Acknowledgements

The experiments presented in this paper were carried out using the HPC facilities of the University of Luxembourg [70]. The manuscript also passed the Luxembourg Centre for Systems Biomedicine internal pre-publication check designed to ensure FAIRness and reproducibility.

Author Contributions

P.W. initiated the study, which involved the overall design and objective, and was led by L.F.D. and J.O.S. in the development of PathoFact 2.0. O.H. contributed to early brainstorming discussions on workflow design and database strategy. P.M. and C.C.L. contributed to the overall discussions. L.F.D. and J.O.S. wrote the draft manuscript. All authors read and commented on the manuscript.

Funding

This work has been supported by the Pélican grant from the Mie and Pierre Hippert-Faber Pélican Foundation under the aegis of Fondation de Luxembourg to JOS, as well as by the Luxembourg National Research Fund (FNR CORE/23/BM/15886415) and the European Research Council (ERC-CoG 863664) to PW. The Luxembourg Government further supported the work through the CoVaLux program.
This research was funded in whole, or in part, by the Luxembourg National Research Fund (FNR), grant reference (FNR CORE/23/BM/15886415). For the purpose of open access, and in fulfilment of the obligations arising from the grant agreement, the author has applied a Creative Commons Attribution 4.0 International (CC BY 4.0) license to any Author Accepted Manuscript version arising from this submission.

Data Availability

Pathofact 2.0 is accessible at https://gitlab.com/uniluxembourg/lcsb/systems-ecology/pathofact2. Additionally, the core databases required to run the pipeline can be found at https://zenodo.org/records/14192463. The ML datasets used for training, validation, and benchmarking of PathoFact 2.0 can be found in https://zenodo.org/records/17647372.

Competing Interests

None declared.

References

1. Hou K, Wu Z-X, Chen X-Y, Wang J-Q, Zhang D, Xiao C, et al.. Microbiota in health and diseases. *Signal Transduct Target Ther*. Springer Science and Business Media LLC; 2022; doi: 10.1038/s41392-022-00974-4.

2. Inda-Díaz JS, Lund D, Parras-Moltó M, Johnning A, Bengtsson-Palme J, Kristiansson E. Latent antibiotic resistance genes are abundant, diverse, and mobile in human, animal, and environmental microbiomes. *Microbiome*. Springer Science and Business Media LLC; 2023; doi: 10.1186/s40168-023-01479-0.

3. Beceiro A, Tomás M, Bou G. Antimicrobial resistance and virulence: a successful or deleterious association in the bacterial world? *Clin Microbiol Rev*. American Society for Microbiology; 2013; doi: 10.1128/CMR.00059-12.

4. Zhu C, Wu L, Ning D, Tian R, Gao S, Zhang B, et al.. Global diversity and distribution of antibiotic resistance genes in human wastewater treatment systems. *Nat Commun*. Springer Science and Business Media LLC; 2025; doi: 10.1038/s41467-025-59019-3.

5. Jian Z, Zeng L, Xu T, Sun S, Yan S, Yang L, et al.. Antibiotic resistance genes in bacteria: Occurrence, spread, and control. *J Basic Microbiol*. Wiley; 2021; doi: 10.1002/jobm.202100201.

6. Alcock BP, Huynh W, Chalil R, Smith KW, Raphenya AR, Wlodarski MA, et al.. CARD 2023: expanded curation, support for machine learning, and resistome prediction at the Comprehensive Antibiotic Resistance Database. *Nucleic Acids Res*. Oxford University Press (OUP); 2023; doi: 10.1093/nar/gkac920.

7. Niu C, Yu D, Wang Y, Ren H, Jin Y, Zhou W, et al.. Common and pathogen-specific virulence factors are different in function and structure. *Virulence*. Informa UK Limited; 2013; doi: 10.4161/viru.25730.

8. Sharma AK, Dhasmana N, Dubey N, Kumar N, Gangwal A, Gupta M, et al.. Bacterial virulence factors: Secreted for survival. *Indian J Microbiol*. 2017; doi: 10.1007/s12088-016-0625-1.

9. Blair JMA, Webber MA, Baylay AJ, Ogbolu DO, Piddock LJV. Molecular mechanisms of antibiotic resistance. *Nat Rev Microbiol*. Springer Science and Business Media LLC; 2015; doi: 10.1038/nrmicro3380.

10. Rodríguez-Beltrán J, DelaFuente J, León-Sampedro R, MacLean RC, San Millán Á. Beyond horizontal gene transfer: the role of plasmids in bacterial evolution. *Nat Rev Microbiol*. Springer Science and Business Media LLC; 2021; doi: 10.1038/s41579-020-00497-1.

11. Galanos C, Freudenberg MA. Bacterial endotoxins: biological properties and mechanisms of action. *Mediators Inflamm*. Wiley; 1993; doi: 10.1155/S0962935193000687.

12. Wishart D, Arndt D, Pon A, Sajed T, Guo AC, Djoumbou Y, et al.. T3DB: the toxic exposome database. *Nucleic Acids Res*. Oxford University Press (OUP); 2015; doi: 10.1093/nar/gku1004.

13. Chakraborty A, Ghosh S, Chowdhary G, Maulik U, Chakrabarti S. DBETH: A database of Bacterial ExoToxins for human. *Nucleic Acids Res*. Oxford University Press (OUP); 2012; doi: 10.1093/nar/gkr942.

14. Green ER, Mecsas J. Bacterial secretion systems: An overview. *Microbiol Spectr*. 2016; doi: 10.1128/microbiolspec.VMBF-0012-2015.

15. Kaushik S, He H, Dalbey RE. Bacterial signal peptides- navigating the journey of proteins. *Front Physiol*. Frontiers Media SA; 2022; doi: 10.3389/fphys.2022.933153.

16. Lybbert AC, Williams JL, Raghuvanshi R, Jones AD, Quinn RA. Mining public mass spectrometry data to characterize the diversity and ubiquity of P. aeruginosa specialized metabolites. *Metabolites*. MDPI AG; 2020; doi: 10.3390/metabo10110445.

17. Elshafie HS, Camele I. An overview of metabolic activity, beneficial and pathogenic aspects of Burkholderia spp. *Metabolites*. MDPI AG; 2021; doi: 10.3390/metabo11050321.

18. Lau GW, Hassett DJ, Ran H, Kong F. The role of pyocyanin in Pseudomonas aeruginosa infection. *Trends Mol Med*. Elsevier BV; 2004; doi: 10.1016/j.molmed.2004.10.002.

19. Ambassadors G, Patrons: World leaders commit to decisive action on antimicrobial resistance. UN Environment. https://www.unep.org/news-and-stories/press-release/world-leaders-commit-decisive-action-antimicrobial-resistance (2024). Accessed 2025 Oct 7.

20. Environment UN: Antimicrobial Resistance (AMR). UNEP - UN Environment Programme. https://www.unep.org/topics/chemicals-and-pollution-action/pollution-and-health/antimicrobial-resistance-amr (2024). Accessed 2025 Oct 7.

21. Bansal MA, Sharma DR, Kathuria DM. A systematic review on data scarcity problem in deep learning: Solution and applications. *ACM Comput Surv*. Association for Computing Machinery (ACM); 2022; doi: 10.1145/3502287.

22. de Nies L, Lopes S, Busi SB, Galata V, Heintz-Buschart A, Laczny CC, et al.. PathoFact: a pipeline for the prediction of virulence factors and antimicrobial resistance genes in metagenomic data. *Microbiome*. Springer Science and Business Media LLC; 2021; doi: 10.1186/s40168-020-00993-9.

23. Dong W, Fan X, Guo Y, Wang S, Jia S, Lv N, et al.. An expanded database and analytical toolkit for identifying bacterial virulence factors and their associations with chronic diseases. *Nat Commun*. Springer Science and Business Media LLC; 2024; doi: 10.1038/s41467-024-51864-y.

24. Ji B, Pi W, Liu W, Liu Y, Cui Y, Zhang X, et al.. HyperVR: a hybrid deep ensemble learning approach for simultaneously predicting virulence factors and antibiotic resistance genes. *NAR Genom Bioinform*. 2023; doi: 10.1093/nargab/lqad012.

25. Rathore AS, Choudhury S, Arora A, Tijare P, Raghava GPS. ToxinPred 3.0: An improved method for predicting the toxicity of peptides. *Comput Biol Med*. Elsevier BV; 2024; doi: 10.1016/j.compbiomed.2024.108926.

26. Kasmanas JC, Magnúsdóttir S, Zhang J, Smalla K, Schloter M, Stadler PF, et al.. Integrating comparative genomics and risk classification by assessing virulence, antimicrobial resistance, and plasmid spread in microbial communities with gSpreadComp. *Gigascience*. 2025; doi: 10.1093/gigascience/giaf072.

27. Wang J, Chitsaz F, Derbyshire MK, Gonzales NR, Gwadz M, Lu S, et al.. The conserved domain database in 2023. *Nucleic Acids Res*. Oxford University Press (OUP); 2023; doi: 10.1093/nar/gkac1096.

28. Blin K, Shaw S, Augustijn HE, Reitz ZL, Biermann F, Alanjary M, et al.. antiSMASH 7.0: new and improved predictions for detection, regulation, chemical structures and visualisation. *Nucleic Acids Res*. Oxford University Press (OUP); 2023; doi: 10.1093/nar/gkad344.

29. . Pyrodigal: Python bindings and interface to Prodigal, an efficient method for gene prediction in prokaryotes. *Journal of Open Source Software*. doi: 10.21105/joss.04296.

30. Camargo AP, Roux S, Schulz F, Babinski M, Xu Y, Hu B, et al.. Identification of mobile genetic elements with geNomad. *Nat Biotechnol*. Springer Science and Business Media LLC; 2024; doi: 10.1038/s41587-023-01953-y.

31. Köster J, Rahmann S. Snakemake--a scalable bioinformatics workflow engine. *Bioinformatics*.

Oxford University Press (OUP); 2012; doi: 10.1093/bioinformatics/bts480.

32. UniProt Consortium. UniProt: The universal protein knowledgebase in 2023. *Nucleic Acids Res*. Oxford University Press (OUP); 2023; doi: 10.1093/nar/gkac1052.

33. Steinegger M, Söding J. MMseqs2 enables sensitive protein sequence searching for the analysis of massive data sets. *Nat Biotechnol*. 2017; doi: 10.1038/nbt.3988.

34. Chawla NV, Bowyer KW, Hall LO, Kegelmeyer WP. SMOTE: Synthetic minority over-sampling technique. *J Artif Intell Res*. AI Access Foundation; 2002; doi: 10.1613/jair.953.

35. Pedregosa F, Varoquaux G, Gramfort A, Michel V, Thirion B, Grisel O, et al.. Scikit-learn: Machine Learning in Python. arXiv [cs.LG].

36. Chen Z, Zhao P, Li F, Leier A, Marquez-Lago TT, Wang Y, et al.. iFeature: a Python package and web server for features extraction and selection from protein and peptide sequences. *Bioinformatics*. 2018; doi: 10.1093/bioinformatics/bty140.

37. Guan J, Chen Y, Goh Y-X, Wang M, Tai C, Deng Z, et al.. TADB 3.0: an updated database of bacterial toxin-antitoxin loci and associated mobile genetic elements. *Nucleic Acids Res*. Oxford University Press (OUP); 2024; doi: 10.1093/nar/gkad962.

38. Zhang J, Guan J, Wang M, Li G, Djordjevic M, Tai C, et al.. SecReT6 update: a comprehensive resource of bacterial Type VI Secretion Systems. *Sci China Life Sci*. Springer Science and Business Media LLC; 2023; doi: 10.1007/s11427-022-2172-x.

39. Liu Y, Liu S, Pan Z, Ren Y, Jiang Y, Wang F, et al.. PAT: a comprehensive database of prokaryotic antimicrobial toxins. *Nucleic Acids Res*. Oxford University Press (OUP); 2023; doi: 10.1093/nar/gkac879.

40. Harms A, Liesch M, Körner J, Québatte M, Engel P, Dehio C. A bacterial toxin-antitoxin module is the origin of inter-bacterial and inter-kingdom effectors of Bartonella. *PLoS Genet*. 2017; doi: 10.1371/journal.pgen.1007077.

41. Yadav SK, Magotra A, Ghosh S, Krishnan A, Pradhan A, Kumar R, et al.. Immunity proteins of dual nuclease T6SS effectors function as transcriptional repressors. *EMBO Rep*. EMBO; 2021; doi: 10.15252/embr.202051857.

42. Danov A, Segev O, Bograd A, Ben Eliyahu Y, Dotan N, Kaplan T, et al.. Toxinome-the bacterial protein toxin database. *MBio*. 2024; doi: 10.1128/mbio.01911-23.

43. Sordo M, Zeng Q. On sample size and classification accuracy: A performance comparison. *Biological and Medical Data Analysis*. Berlin, Heidelberg: Springer Berlin Heidelberg;

44. Liu B, Zheng D, Zhou S, Chen L, Yang J. VFDB 2022: a general classification scheme for bacterial virulence factors. *Nucleic Acids Res*. Oxford University Press (OUP); 2022; doi: 10.1093/nar/gkab1107.

45. Arango-Argoty G, Garner E, Pruden A, Heath LS, Vikesland P, Zhang L. DeepARG: a deep learning approach for predicting antibiotic resistance genes from metagenomic data. *Microbiome*. 2018; doi: 10.1186/s40168-018-0401-z.

46. Feldgarden M, Brover V, Gonzalez-Escalona N, Frye JG, Haendiges J, Haft DH, et al.. AMRFinderPlus and the Reference Gene Catalog facilitate examination of the genomic links among antimicrobial resistance, stress response, and virulence. *Sci Rep*. Springer Science and Business Media LLC; 2021; doi:

10.1038/s41598-021-91456-0.

47. Ugarcina Perovic S, Ramji V, Chong H, Duan Y, Maguire F, Coelho LP. argNorm: normalization of antibiotic resistance gene annotations to the Antibiotic Resistance Ontology (ARO). *Bioinformatics*. Oxford University Press (OUP); 2025; doi: 10.1093/bioinformatics/btaf173.

48. Chicco D, Jurman G. The advantages of the Matthews correlation coefficient (MCC) over F1 score and accuracy in binary classification evaluation. *BMC Genomics*. Springer Science and Business Media LLC; 2020; doi: 10.1186/s12864-019-6413-7.

49. Chen C, Xu Y, Ouyang J, Xiong X, Łabaj PP, Chmielarczyk A, et al.. VirulentHunter: deep learning-based virulence factor predictor illuminates pathogenicity in diverse microbial contexts. *Brief Bioinform*. Oxford University Press (OUP); 2025; doi: 10.1093/bib/bbaf271.

50. Gupta A, Malwe AS, Srivastava GN, Thoudam P, Hibare K, Sharma VK. MP4: a machine learning based classification tool for prediction and functional annotation of pathogenic proteins from metagenomic and genomic datasets. *BMC Bioinformatics*. Springer Science and Business Media LLC; 2022; doi: 10.1186/s12859-022-05061-7.

51. Sharma A, Garg A, Ramana J, Gupta D. VirulentPred 2.0: An improved method for prediction of virulent proteins in bacterial pathogens. *Protein Sci*. Wiley; 2023; doi: 10.1002/pro.4808.

52. Xie R, Li J, Wang J, Dai W, Leier A, Marquez-Lago TT, et al.. DeepVF: a deep learning-based hybrid framework for identifying virulence factors using the stacking strategy. *Brief Bioinform*. Oxford University Press (OUP); 2021; doi: 10.1093/bib/bbaa125.

53. Sharma N, Naorem LD, Jain S, Raghava GPS. ToxinPred2: an improved method for predicting toxicity of proteins. *Brief Bioinform*. Oxford University Press (OUP); 2022; doi: 10.1093/bib/bbac174.

54. Morozov V, Rodrigues CHM, Ascher DB. CSM-Toxin: A web-server for predicting protein toxicity. *Pharmaceutics*. MDPI AG; 2023; doi: 10.3390/pharmaceutics15020431.

55. Kaper JB, Nataro JP, Mobley HL. Pathogenic Escherichia coli. *Nat Rev Microbiol*. Springer Science and Business Media LLC; 2004; doi: 10.1038/nrmicro818.

56. Johnson TJ, Nolan LK. Pathogenomics of the virulence plasmids of Escherichia coli. *Microbiol Mol Biol Rev*. American Society for Microbiology; 2009; doi: 10.1128/MMBR.00015-09.

57. Henderson B, Martin A. Bacterial virulence in the moonlight: multitasking bacterial moonlighting proteins are virulence determinants in infectious disease. *Infect Immun*. American Society for Microbiology; 2011; doi: 10.1128/IAI.00179-11.

58. Henderson B, Martin A. Bacterial moonlighting proteins and bacterial virulence. *Curr Top Microbiol Immunol*. 2013; doi: 10.1007/82_2011_188.

59. Pancholi V, Fischetti VA. A major surface protein on group A streptococci is a glyceraldehyde-3-phosphate-dehydrogenase with multiple binding activity. *J Exp Med*. Rockefeller University Press; 1992; doi: 10.1084/jem.176.2.415.

60. Henderson B, Martin A. Bacterial moonlighting proteins and bacterial virulence. *Curr Top Microbiol Immunol*. Curr Top Microbiol Immunol; 2013; doi: 10.1007/82_2011_188.

61. Henderson B, Martin A. Bacterial virulence in the moonlight: multitasking bacterial moonlighting proteins are virulence determinants in infectious disease. *Infect Immun*. American Society for

Microbiology; 2011; doi: 10.1128/IAI.00179-11.

62. Barel M, Charbit A. Detection of the interaction between host and bacterial proteins: eukaryotic nucleolin interacts with Francisella elongation factor Tu. *Methods Mol Biol*. Methods Mol Biol; 2014; doi: 10.1007/978-1-4939-1261-2_7.

63. Granato D, Bergonzelli GE, Pridmore RD, Marvin L, Rouvet M, Corthésy-Theulaz IE. Cell surface-associated elongation factor Tu mediates the attachment of Lactobacillus johnsonii NCC533 (La1) to human intestinal cells and mucins. *Infection and immunity*. Infect Immun; 2004; doi: 10.1128/iai.72.4.2160-2169.2004.

64. Kamiya S, Yamaguchi H, Osaki T, Taguchi H. A virulence factor of Helicobacter pylori: role of heat shock protein in mucosal inflammation after H. pylori infection. *J Clin Gastroenterol*. 1998; doi: 10.1097/00004836-199800001-00007.

65. Hickey TBM, Ziltener HJ, Speert DP, Stokes RW. Mycobacterium tuberculosis employs Cpn60.2 as an adhesin that binds CD43 on the macrophage surface: M. tuberculosis Cpn60.2 mediates macrophage binding via CD43. *Cell Microbiol*. Hindawi Limited; 2010; doi: 10.1111/j.1462-5822.2010.01496.x.

66. Lehner T, Bergmeier LA, Wang Y, Tao L, Sing M, Spallek R, et al.. Heat shock proteins generate β-chemokines which function as innate adjuvants enhancing adaptive immunity. *Eur J Immunol*. 2000; doi: 10.1002/1521-4141(200002)30:2<594::AID-IMMU594>3.0.CO;2-1.

67. UniProt Consortium. UniProt: The universal protein knowledgebase in 2025. *Nucleic Acids Res*. Oxford University Press (OUP); 2025; doi: 10.1093/nar/gkae1010.

68. Eddy SR. Profile hidden Markov models. *Bioinformatics*. Oxford University Press (OUP); 1998; doi: 10.1093/bioinformatics/14.9.755.

69. Larralde M, Zeller G. PyHMMER: a Python library binding to HMMER for efficient sequence analysis. *Bioinformatics*. Oxford Academic; 2023; doi: 10.1093/bioinformatics/btad214.

70. Varrette S, Cartiaux H, Peter S, Kieffer E, Valette T, Olloh A. Management of an academic HPC & research computing facility: The ULHPC experience 2.0. *Proceedings of the 2022 6th High Performance Computing and Cluster Technologies Conference*. New York, NY, USA: ACM; doi: https://doi.org/10.1145/3560442.3560445

Manuscript with highlights

Click here to access/download;Manuscript;PathoFact2_resubmission_revw2

PathoFact 2.0: An Integrative Pipeline for the Prediction of Antimicrobial Resistance Genes, Virulence Factors, Toxins and Toxin-associated Proteins, and Biosynthetic Gene Clusters in Metagenomes

Luis F. Delgado (luis.delgado@uni.lu)*[1][0000-0001-7850-5285], Júlia Ortís Sunyer (julia.ortissunyer@uni.lu)*[1] [0000-0002-2714-7067], Cedric C. Laczny (cedric.laczny@uni.lu)[1][0000-0002-1100-1282], Oskar Hickl (oskar.hickl@lih.lu)[1][0000-0001-9959-8767], Patrick May (patrick.may@uni.lu)[1][0000-0001-8698-3770] & Paul Wilmes (paul.wilmes@uni.lu)[1,2][0000-0002-6478-2924]

1. Luxembourg Centre for Systems Biomedicine, University of Luxembourg, Esch-sur-Alzette, Luxembourg
2. Department of Life Sciences and Medicine, Faculty of Science, Technology and Medicine, University of Luxembourg, Esch-sur-Alzette, Luxembourg

Contact: Paul Wilmes (paul.wilmes@uni.lu)

*These authors contributed equally

Abstract

Background

Antimicrobial resistance genes (ARG) and virulence factors (VFs) are central contributors to the global health crisis surrounding drug-resistant infections.

Findings

We introduce PathoFact 2.0, an enhanced pipeline for improved ARG, VF, toxin, and biosynthetic gene clusters (BGC) prediction. Key improvements include an updated machine learning (ML) model for VF identification, expanded hidden Markov model profiles for VFs and toxin-associated proteins, a new ML model for toxin and toxin-associated proteins identification, and the integration of antiSMASH 7.0 for predicting biosynthetic gene clusters.

Conclusions

Our upgrades make PathoFact 2.0 a more powerful and user-friendly platform for predicting microbiome-based pathogenicity and resistance, providing a crucial tool for better understanding and addressing the challenges posed by antimicrobial resistance and infectious diseases.

PathoFact 2.0 is available at https://gitlab.com/uniluxembourg/lcsb/systems-ecology/pathofact2. It is compatible with Linux operating systems.

Keywords

Antimicrobial resistance genes, virulence factors, toxin-associated proteins, biosynthetic gene clusters, metagenomes, machine learning

Findings

Introduction

Microbiomes are highly complex and diverse ecological communities composed of bacteria, archaea, viruses, and microeukaryotes. These communities include both commensal microorganisms, which can contribute to host health, and pathogenic or opportunistic microorganisms that can cause disease under specific conditions. Microbial communities generally exist in synergistic relationships with their hosts, playing critical roles in maintaining physiological homeostasis and regulating immune function. However, disruption of this balanced microbial ecosystem, known as microbial dysbiosis, can impair normal body functions and has been associated with the development of various diseases, including cardiovascular diseases, cancers, and respiratory disorders [1].

Moreover, these microorganisms play a critical role in the development of antibiotic-resistant infections through the presence of antimicrobial resistance genes (ARGs) and virulence factors (VFs)[2,3]. ARGs are genetic elements that confer bacterial resistance to antibiotics. Many ARGs are encoded on mobile genetic elements (MGEs) and are therefore often horizontally transmitted [4]. ARGs can be divided into categories based on the antibiotics to which they confer resistance [5]. The Antibiotic Resistance Ontology (ARO) contains information on ARGs, the mutations that cause them, their products, mechanisms, associated phenotypes, antibiotics, and molecular targets [6].

Bacterial pathogens use specific genes, known as VFs, to attach to and invade host tissues, survive within the host, spread, and ultimately cause damage. The harm inflicted can vary, ranging from minor disruptions to severe or even fatal outcomes [7]. VFs can be classified as secretory, membrane-associated, or cytosolic. Cytosolic virulence factors promote rapid adaptive shifts in bacterial metabolism, physiology, and morphology, enhancing survival and proliferation within the host. Membrane-associated factors contribute to bacterial adhesion and immune evasion at the host-cell interface. Secretory factors constitute a critical part of the bacterial armamentarium, enabling bacteria to counteract innate and adaptive immune defences. Secretory VFs often exhibit synergistic effects and induce cytotoxicity in host cells [8]. VFs are often located on MGEs, such as transposons, plasmids and phages, facilitating their transfer between bacterial cells [9,10].

Bacterial toxins play a crucial role in the development of infectious diseases, alongside various virulence factors employed by pathogens. They disrupt host processes and manipulate immune responses. Some toxins impair protein synthesis, destroy blood cells, or affect the nervous system. Bacterial toxins can be divided into two main categories: cell-associated endotoxins and extracellular, diffusible exotoxins. Endotoxins, such as lipopolysaccharides, are found in the outer membranes of Gram-negative bacteria and serve as potent inflammatory mediators that can induce systemic toxicity and septic shock in infected hosts [11]. Exotoxins, which are typically polypeptides and proteins, can stimulate a range of host responses by either acting directly on cell receptors or through enzymatic modulation [12,13]. Many bacterial toxins are secreted proteins that require signal peptides. Signal peptides are short amino acid sequences at the N-terminus of proteins that direct them to specific cellular compartments, such as the periplasm [14,15].

Biosynthetic gene clusters (BGCs) are responsible for synthesising specialised metabolites (SMs). Some SMs can increase pathogenicity; for example, clinical isolates of *Pseudomonas aeruginosa* produce siderophores, rhamnolipids, quinolones, and phenazines [16]. Similarly, *Burkholderia* strains produce virulence factors, such as toxoflavin from *Burkholderia glumae* [17]. Notably, pyocyanin, a redox-active phenazine produced by *Pseudomonas aeruginosa*, plays a crucial role as a virulence factor in lung infections [18].

The threat that ARGs, VFs and toxins pose to human health is significant. The United Nations have identified antimicrobial resistance as a global threat, with estimates attributing 1.27 million deaths annually to drug-resistant infections, potentially rising to 10 million by 2050 if unaddressed [19,20]. Thus, accurately predicting potential ARG and VF profiles is essential for early intervention, enabling anticipation of infection severity, improving treatment strategies, and ultimately reducing mortality rates from disease-causing pathogens.

Predicting and annotating ARGs, VFs, and toxins is challenging due to limited well-annotated data [21] and complex mechanisms involving gene transfer, mutations, and multifactorial interactions. Traditional annotation methods, which rely on sequence similarity, may overlook novel ARGs, VFs and toxins. In contrast, machine learning offers robust solutions through pattern recognition, enabling accurate predictions even with limited training data.

An integrated bioinformatics pipeline enhances analysis by simultaneously examining ARGs, VFs, toxins, signal peptides, and BGCs from a single metagenomic sample. This comprehensive approach provides a more complete view of bacterial pathogenicity by capturing the full spectrum of virulence mechanisms, including antimicrobial resistance, toxin production, and secondary metabolic capabilities. This holistic analysis improves insights into pathogenicity and resistance, streamlines workflows, and simplifies data interpretation.

PathoFact 1.0, a pipeline first introduced in 2020, integrates ARG, VF, and bacterial toxin prediction from metagenomic data into a single tool [22]. Since the publication of PathoFact, several tools have been implemented to predict ARGs, VFs, and bacterial toxins [23–25]. HyperVR [24] has attempted to predict them simultaneously, analogous to PathoFact. However, HyperVR's repository is no longer available online, and the Zenodo archive from its original submission lacks the necessary databases, hence rendering it unusable. gSpreadComp [26] recently proposed a workflow that integrates comparative genomics, plasmid-mediated transfer assessment, and resistance-virulence risk-ranking to facilitate hypothesis generation for targeted experimental validation by identifying concerning resistant hotspots in complex microbial datasets. However, while gSpreadComp is valuable for comparative, community-level prioritisation, ARGs are predicted using only one tool, DeepARG, and VF are annotated using databases and alignment-based approaches rather than predictive models and machine learning frameworks. In addition, it lacks a signal peptide or a BGC annotation framework needed for comprehensive microbiome-based pathogenicity profiling, and the pipeline accepts only DNA sequences as input.

Here, we present PathoFact 2.0 (Figure 1). It enhances the previous version by supporting protein sequences or contigs as input and by updating the ML VF model and the hidden Markov model (HMM) profiles of the conserved domain databases (CDD) [27] for VF and toxin-associated protein annotation. We have also introduced the ability to predict BGCs using antiSMASH 7.0 [28]. antiSMASH is a tool that identifies, annotates, and analyses secondary metabolite BGCs across genomes.

Figure 1. Schematic representation of PathoFact 2.0. Solid lines denote core modules, while dotted lines indicate optional user selection. The input is a FASTA file with either contig or protein sequences. If the input is a FASTA file containing contigs, open reading frames (ORFs) are predicted using Pyrodigal-gv. If the biosynthetic gene cluster (BGC) option is selected, antiSMASH will use the GBK file for BGC prediction. GeNomad is used for MGE and phage prediction, producing a FASTA file of protein sequences. Protein sequences are dereplicated using MMseqs2 to retain non-redundant sequences (based on 100% identity and coverage). After dereplication, antimicrobial resistance genes (ARGs), virulence factors (VFs) and toxins and their associated proteins are predicted using their respective modules. SignalP predicts the presence of signal peptides and their cleavage sites in proteins from

archaea, bacteria and eukarya. Individual reports are generated for each module, and an integrated report is produced that combines all module reports.

Pipeline Structure

Unlike version 1.0, which supports only contigs, PathoFact 2.0 accepts nucleotide sequences of contigs and protein sequence FASTA files, with proteins dereplicated by our tool to retain only non-redundant sequences (based on 100% identity and coverage). For contig-based inputs, open reading frames are predicted using Pyrodigal-gv (version 0.3.2; [29,30]; https://github.com/althonos/pyrodigal-gv), a Python library that binds to Prodigal [22], followed by the detection of MGEs and phages using geNomad (version 1.8.0; [30]). GeNomad processes only nucleotide sequences; therefore, MGEs and phages are not detected in protein sequence inputs. Based on user configuration, the pipeline then analyses the processed sequences using the ARG, VF, toxin-associated, and BGC (via antiSMASH) prediction modules. The information is compiled into individual module reports and an integrated report, also incorporating details from SignalP and geNomad (Figure 1). Additionally, PathoFact 2.0 generates a FASTA file of proteins identified as ARGs, VFs, or toxin-associated proteins.

Pipeline Installation

PathoFact 2.0 is implemented using Snakemake (version 7.25.0; [31]). An installation script simplifies the setup by installing the required software and downloading databases with a single command. PathoFact 2.0 is open-source (GNU General License v3.0 or later) and freely available at https://gitlab.com/uniluxembourg/lcsb/systems-ecology/pathofact2, where detailed instructions for pipeline installation, configuration, and output are provided.

Pipeline Updates

We implemented thorough updates across all PathoFact modules. Notably, we developed two new machine-learning models: one to predict virulence factors and another to identify toxin-associated proteins. A schematic diagram of the construction of the training and test datasets is shown in Figure 2. In the sections below, we detail the updates to each module.

Figure 2. Schematic representation of the datasets used for PathoFact 2.0 toxin-associated and virulence factors modules training and testing. Shades of green represent the toxin-associated datasets; shades of blue represent the non-pathogenic dataset; and shades of purple represent the virulence factor datasets.

Generalities about the "non-pathogenic" dataset and the machine learning training setup

The "non-pathogenic" dataset for the ML models was constructed by selecting SwissProt [32] sequences lacking ARG, VF, and toxin keywords [KW-0568 (pathogenesis-related protein), KW-0843 (virulence), KW-0800 (toxin), KW-0046 (antibiotic resistance), KW-9995 (disease)] and limited to bacteria (taxonomy_id 2), archaea (taxonomy_id 2157), fungi (taxonomy_id 4751), and viruses (taxonomy_id 10239) (Figure 2). Additionally, proteins from non-pathogenic organisms to humans (Supplementary Table S1; Figure 2) were included from NCBI. MMseqs2 (version 15.6f452; [33]) was used to obtain a set of non-redundant (clustered at 100% identity and coverage) protein sequences (Figure 2).

ML models were trained using 80% of the sequences for training and 20% of the sequences for testing. The Synthetic Minority Oversampling Technique (SMOTE) was employed to address the dataset's

imbalance [34]. Using the XGBoost Python package (https://xgboost.readthedocs.io/en/stable/index.html) and the RandomForest (RF) Scikit-learn (version 1.5.2; [35]), several ML models were trained and tested using k-mers (k=3 to 8) or protein sequence composition features (amino acid composition (AAC), dipeptide composition (DPC), composition (CTDC), transition (CTDT), and distribution (CTDD) [36]) as features. Hyperparameter optimisation was performed, using a 5-fold cross-validation with HalvingGridSearchCV from scikit-learn [35]. The best-performing model was selected based on the Matthews correlation coefficient (MCC) score.

Toxin-Associated Protein Prediction Updates

Compared to version 1.0, the toxin prediction module now employs a ML model instead of a purely alignment-based bit score threshold. Curated training data was obtained from SwissProt [32], filtered for bacterial (taxonomy_id 2), archaeal (taxonomy_id 2157), fungal (taxonomy_id 4751) and viral (taxonomy_id 10239) toxin sequences (KW-0800, toxin)(Figure 2). The dataset was supplemented with entries from toxin-specific databases such as the Toxin Exposome Database (T3DB) [12], which catalogues bacterial protein toxins; the Database for Bacterial ExoToxins (DBETH) [13]; TADB version 3.0, which includes protein sequences of bacterial toxin–antitoxin (TA) pairs from types I to VIII [37]; sequences from SecReT6 [38], encompassing T6SS gene cluster components, T6SS regulator (T6SR), accessory proteins (T6SA), effectors (T6SE), and immunity proteins (T6SI); and the prokaryotic antimicrobial toxins (PAT) database [39] (Figure 2).

MMseqs2 (version 15.6f452; [33]) was used to dereplicate the dataset of 1,112,357 protein sequences (100% identity and coverage), yielding 213,363 unique protein sequences, corresponding to the "toxin-associated" dataset (Figure 2). It is essential to note that this dataset encompasses both effector toxin proteins and their associated proteins, including antitoxins, regulators, and accessory proteins. This offers three main benefits: 1) Recent reports suggest that the same bacterial toxins can function as part of self-inhibiting toxin-antitoxin modules within one organism, while in another organism, they have evolved into toxin effectors that are injected into target cells [40,41]. 2) In bacteria, genes located in close proximity frequently exhibit functional associations, such as those co-transcribed within operons. A comprehensive toxin dataset, including both toxins and their associated proteins, facilitates the identification of novel toxins and related genes through their genomic context, referred to as "toxin islands." These islands may be involved in toxin biosynthesis, processing, or secretion, and may also confer immunity or facilitate horizontal gene transfer among bacterial populations. Notably, they are often rich in mobile genetic elements [42]. 3) A large database improves the performance of machine learning classification methods [43].

HMM profiles were built using the conserved-domain FASTA files (https://ftp.ncbi.nih.gov/pub/mmdb/cdd/fasta.tar.gz) from CDD [27]. The 213,363 unique protein sequences in the "toxin-associated" dataset were annotated using the CDD HMM profiles. Those with a bitscore above 25 were chosen as HMM profiles for toxin and toxin-associated protein annotation and incorporated into Pathofact 2.0 for protein annotation.

Although there is no standard for creating negative datasets, they play a crucial role in influencing model performance. Therefore, to improve the quality of our "non-toxin" dataset, potential ARGs, VFs (with high probability), and toxins were filtered out of the "non-pathogenic" dataset using PathoFact 1.0 predictions. The final "non-toxin" dataset consists of 213,129 non-redundant protein sequences (Figure 2).

The toxin-associated ML model is a RF with 5-mer features (default hyperparameter setting, i.e, number of trees in the forest [n_estimators]=100, maximum depth of the tree [max_depth]=None, minimum number of samples required to split an internal node [min_samples_split]=2).

The toxin-associated protein prediction module generates a report containing the proteinID, protein domains, bitscore, toxin-associated ML probability, other identical proteins found in the sample, and optionally SignalP, plasmid marker, and virus marker information.

VF Prediction Updates

The VF prediction model was refined and updated with new HMM profiles. Training data was derived from SwissProt [32], selecting sequences annotated with the virulence keyword (KW-0843) and expanded using the Virulence Factor Database (VFDB; [44]) (Figure 2). After dereplication (with 100% identity and coverage), the original set of 32,511 sequences, using MMseqs2, comprised 30,695 non-redundant sequences, corresponding to the "VF dataset" (Figure 2). We searched the "VF dataset" against the CDD HMM profiles, selecting those with a bit score of 25 or higher as VF HMM profiles for PathoFact 2.0. The HMM profile dataset annotates the predicted VF domains rather than using them as input to the classification, as in the previous version.

To create the "non-VF" dataset for the ML VF model, we filtered out any potential VFs (with high and low probabilities), ARGs, and toxins based on PathoFact 1.0 predictions from the "non-pathogenic" dataset. This resulted in a dataset of 41,774 VF protein sequences (Figure 2).

The VF model uses XGBoost with protein sequence composition features (Step size shrinkage used in update to prevent overfitting [learning_rate]= 0.1, number of trees 'n_estimators]= 2000). The VF module generates a report containing the proteinID, protein domains, bitscore, virulence factor ML probability, other identical proteins found in the sample, and optionally SignalP, plasmid marker, and virus marker information.

ARG Prediction Updates

ARG prediction in PathoFact 2.0 integrates DeepARG (version 1.0.2; [45]), RGI (version 6; [6]), and AMRFinderPlus (version 3.12.8; [46]). DeepARG and RGI have received updates from their developers since the release of PathoFact 1.0, which have been incorporated into PathoFact 2.0. In addition, AMRFinderPlus has been newly integrated into PathoFact 2.0. Each tool has distinct strengths: DeepARG offers high precision and recall; RGI provides robust predictions based on an extensive database, utilising homology and single-nucleotide polymorphism (SNP) models; and AMRFinderPlus efficiently identifies resistance genes and mutations using NCBI resources.

The ARG prediction module (Figure 1) report includes protein IDs, ARG classes, prediction probabilities, database accession numbers, and optional data on signal peptides, plasmids, and virus markers. PathoFact 2.0 uses argNorm [47] to map detected genes to the ARO, thereby facilitating comparison of ARG annotation outputs by ensuring standardised and comparable results. Supplementary Figures S1A and S2A compare the performance of PathoFact 2.0 with that of its predecessor, PathoFact, in identifying ARGs.

Additional Functionalities

PathoFact 2.0 integrates SignalP (version 6; [24]) and antiSMASH (version 7.0; [28]), both of which are optional features that accommodate diverse research needs. SignalP is designed to predict the presence and location of signal peptides in protein sequences. It requires a separate license and must be requested by the user individually. AntiSMASH is designed to identify and annotate BGCs in bacterial and fungal genomes. Since AntiSMASH is a resource-intensive tool, we set it up as an optional module and provide the option to run it in chunks.

Evaluation of the performance of the PathoFact 2.0 pipeline

We evaluated the performance of PathoFact 2.0 and the new toxin-associated and VF modules using the test datasets described above. We did not include ARGs and BGCs in the validation step, as the respective modules are based on existing tools that have already demonstrated high accuracy [6,28,45,46]. Figure 3 provides a schematic overview of the benchmarking datasets used for the toxin-associated and VF modules.

Figure 3. Schematic representation of the construction of the datasets used for benchmarking. A) Toxin benchmarking dataset. B) Virulence factor benchmarking dataset.

Virulence factors and toxin-associated protein prediction

The VF and toxin-associated modules (Figure 1) were evaluated across different ML models-predicted probability thresholds, corresponding to the model's confidence in the positive class (VF or toxin-associated). Analyses were performed on the full test dataset and on subsets of the test dataset. These subset datasets were created based on sequence similarity to the training dataset, with a range of 40% to 100% similarity and 80% coverage (Figure 2). This approach aimed to assess prediction accuracy on proteins in the testing dataset with low similarity to the training dataset, specifically including only sequences with less than 40-100% identity to any training sequence. The performance evaluation is based on the Matthew correlation coefficient (MCC) and the precision (to reduce the number of false positive VF and toxin-associated predictions), taking into account the dataset imbalance (a higher number of "non-toxin" and "non-VF" sequences compared to "toxin-associated" and "VF" sequences in the test subsets). The MCC is a more reliable statistical measure that yields a high score only when the prediction performs well across all four categories of the confusion matrix (true positives, false negatives, true negatives, and false positives), and it is proportional to both the number of positive and negative elements in the dataset [48]. We found that predicted probabilities of 0.6 for toxin-associated proteins and 0.9 for VFs provide a good balance between high MCC and precision across different test subsets (Figure 4 and Supplementary Tables S3 and S4).

Figure 4. Performance evaluation of toxin-associated and virulence factor prediction modules across probability thresholds. A) Toxin-associated prediction module evaluation. **B)** Virulence factors prediction module evaluation. The modules were evaluated across a range of predicted class probabilities (0.5-0.9). The entire test dataset (All) and subsets of the test datasets were used for evaluation. These subset datasets were created based on sequence similarity to the training dataset, with similarity levels of 40%, 60%, 80%, and 100%, and an 80% coverage threshold. Only sequences with similarity below these percentages were included in the respective test subsets.

Benchmarking

The Pathofact 2.0 VF prediction module was compared to VirulentHunter [49], using the default parameters. VirulentHunter is a deep learning framework that simultaneously identifies and classifies VFs directly from protein sequences, which outperforms other virulence factor predictors (MP4 [50], VirulentPred 2.0 [51], and DeepVF [52]). A notable feature of VirulentHunter is that it provides VF category classification; however, it takes about 2 minutes to analyse 500 protein sequences (using 1 GPU), which is a drawback for metagenomic sample analysis, where thousands to millions of proteins are predicted from a single sample. PathoFact 2.0 requires only 4 seconds (using 1 CPU, with the option to utilise more CPUs) to analyse 500 protein sequences (Table 1).

Table 1. Runtime comparison of PathoFact 2.0 and VirulentHunter

Number of protein sequences	VirulentHunter	PathoFact 2.0				
	1 GPU	1 CPU	2 CPU	4 CPU	6 CPU	8 CPU
500	2 min 21 s	3.7 s	2.6 s	2.0 s	1.9 s	1.8 s
5500	25 min 42 s	29.9 s	17.4 s	10.2 s	8.1 s	7.4 s
10000	54 min 11 s	55.4 s	29.3 s	16.7 s	13.1 s	11.5 s
30000	2 h 45 min 50 s	2 min 45 s	1 min 32.9 s	52.1 s	40.3 s	36.0 s

Since VirulentHunter and Pathofact 2.0 employ a similar method to generate the "VF dataset" for model training, we removed sequences from the Pathofact 2.0 test dataset that have 100% identity (≥80% coverage) to the VirulentHunter training dataset, resulting in a "clean VF test dataset" (Figure 3). This ensures that neither model used the test sequences for training. We applied the same test-subset approach described earlier: the subset datasets were created based on sequence similarity to the Pathofact 2.0 training dataset, with similarity ranging from 40% to 100% and 80% coverage of the "clean VF test dataset" (Figure 3). By stratifying test sets by decreasing sequence similarity to the training data, we explicitly evaluated model performance across progressively more divergent proteins. This study demonstrates that PathoFact 2.0 maintains consistent performance even when test sequences share 40% similarity or less with the training set (Figure 5B, Supplementary Figure S3). In addition to VirulentHunter, we benchmarked PathoFact 2.0 against the virulence factor prediction module of PathoFact 1.0 and against metaVF [23], an alignment-based toolkit (based on BLAST) that identifies species-level VFs associated with pathobionts. DNA gene sequences from the test subsets were used as input, since both tools accept only contigs.

The Pathofact 2.0 toxin-associated module was compared with ToxinPred2 [42] using the default parameters, i.e., Hybrid (RF+BLAST+MERCI) with a threshold of 0.6. The ToxinPred2 website restricts predictions to a certain number of proteins (around 2000). Since ToxinPred2 is designed to predict protein toxicity, we selected sequences from the Pathofact 2.0 "toxin-associated" test dataset that are directly linked to toxins and removed the remaining "toxin-associated" proteins (Figure 3). In short, using the header information from the Pathofact 2.0 "toxin-associated" test dataset, we kept only toxin sequences from the toxin-antitoxin sequences from the TADB, the effector factor sequence from the SecReT6 database, bacterial protein toxins from T3DB, bacterial exotoxins from DBETH, and sequences from Swissprot (KW-0800, toxin), as previously described. Additionally, we kept sequences longer than 35 amino acids and excluded protein sequences containing the non-standard amino acids 'BJOUXZ' as the ToxinPred2 dataset was created using these criteria [53] (Figure 3). From these, we randomly selected 1000 sequences. Then, we randomly selected 1000 sequences from the Pathofact 2.0 "non-toxin" test dataset (Figure 3). This resulted in a total of 2000 sequences for benchmarking PathoFact 2.0 against the ToxinPred2 webserver. Due to the limited number of sequences, we did not use the test-subset approach to evaluate ToxinPred2 and Pathofact 2.0 toxin-associated modules on this 2000-sequence test dataset (Figure 3). We also use the same test dataset to benchmark CSM-toxin v1.0.1, a deep learning model for toxin prediction [54], as well as the toxin prediction module from PathoFact 1.0. DNA gene sequences from the test dataset were used as input.

As shown in Figure 5 (and in the Supplementary Tables S5 and S6 and the AUROC curves in Supplementary Figures S3 and S4), Pathofact 2.0 VF and toxin-associated modules exhibited higher MCC values across different test subsets compared to VirulentHunter, ToxinPred2 and CSM-toxin. Because the negative dataset was built and then filtered using PathoFact 1.0, the precision for PathoFact 1.0 is 1,

which affects the MCC calculation. To avoid biased comparisons, we report recall only for PathoFact 1.0. Figure 5 shows that PathoFact 2.0 VF and toxin-associated modules achieved higher recall across test subsets than PathoFact 1.0, and better recall and MCC than metaVF.

Figure 5. Benchmarking of toxin and virulence factor prediction performance. A) Toxin-associated module benchmarking. The Pathofact 2.0 toxin-associated module was compared with ToxinPred2 (Hybrid: RF+BLAST+MERCI, threshold = 0.6) using its web-based version and CSM-toxin v 1.0.1. A balanced toxin test dataset (1,000 toxin and 1,000 non-toxin sequences) was built from the Pathofact 2.0 toxin-associated test dataset, selecting only sequences from curated toxin sources (TADB, SecReT6, T3DB, DBETH, SwissProt), applying ToxinPred2's filtering criteria. Several predicted probability cutoffs, 0.5, 0.6 and 0.8, of the Pathofact 2.0 toxin-associated module were evaluated. MCC, precision, and recall are shown. **B)** Virulence factors module benchmarking. The Pathofact 2.0 VF module was compared to VirulentHunter. Sequences identical (100% identity, ≥80% coverage) to VirulentHunter's training data were removed from the Pathofact 2.0 test dataset. Then, test subset datasets were created based on 40–100% similarity to the Pathofact 2.0 training set. These subset datasets were created based on sequence similarity to the training dataset, with similarity levels of 40%, 60%, 80%, and 100%, and an 80% coverage threshold. Only sequences with similarity below these percentages were included in the respective test subsets. Several predicted probability cut-offs (0.5, 0.6, 0.8, and 0.9) of the Pathofact 2.0 VF module were evaluated. MCC, precision, and recall are presented for each test subset. Since the negative dataset was built and then filtered using PathoFact 1.0, the precision is 1 for PathoFact 1.0, therefore affecting MCC calculation. To avoid biased comparisons, we report recall only for PathoFact 1.0.

Virulence factors and toxin-associated protein prediction with contig sequences as input

To evaluate PathoFact 2.0 at the contig level, we analysed publicly available complete genomes from pathogenic and non-pathogenic bacteria, including various *Escherichia coli* strains. Figure 6 shows distinct differences in virulence and toxin-related profiles between pathogenic and non-pathogenic *E. coli* strains, especially regarding virulence- and toxin-associated proteins encoded on MGEs, such as plasmids and prophages. Nonetheless, analysis of individual VF predictions reveals considerable overlap in the number of VF genes detected across both pathogenic and non-pathogenic strains. This highlights that the presence of a VF gene is not a reliable marker of pathogenicity and emphasises the importance of considering genomic and functional context when assessing virulence potential.

It is well known that VFs of pathogenic *E. coli* are often encoded on genetic elements, such as plasmids, bacteriophages, transposons, and pathogenicity islands, which can be mobilised into different strains to create novel combinations of virulence factors [55,56]. The same pattern is observed in pathogenic strains of several genera compared to non-pathogenic strains (Supplementary Figure S5), particularly for *Klebsiella pneumoniae* and *Salmonella enterica*. These findings highlight the importance of examining virulence from a systems perspective rather than focusing solely on the presence or absence of individual factors. A comprehensive assessment should consider not only whether a virulence- or toxin-associated protein is encoded within an MGE but also its functional context, such as whether it is secreted or part of a BGC.

Figure 6. Comparative analysis of toxin-associated and virulence factor profiles in non-pathogenic and pathogenic *Escherichia coli* strains. Bar charts represent the distribution of predicted toxin-associated (A) and virulence-associated (B) proteins across non-pathogenic (left panel) and pathogenic (right panel) *E. coli* strains. The categories include total predicted virulence factors/toxin-associated proteins (dark blue), those associated with plasmid markers (red), those associated with phage markers (green), and proteins predicted by SignalP to be secreted (light blue). Additional categories include toxin-associated virulence factors (TOX-associated and VF, grey) and biosynthetic gene clusters

overlapping (purple). Numerical values above each bar indicate the total count of proteins identified in each category for the corresponding strain.

The PathoFact 2.0 VF module was compared to PathoFact 1.0 and metaVF. To our knowledge, no other method is available to predict VF from contig sequences and identify plasmid-encoded or prophage-associated VF. However, we included VirulentHunter in the comparison. Because VirulentHunter does not accept contig sequences as input, protein-coding genes were first predicted from contigs using Pyrodigal-gv and the resulting protein sequences were subsequently analysed. PathoFact 2.0 consistently predicted a greater number of VFs than both PathoFact 1.0 and metaVF (Figure 7, Supplementary Figures S6–S8). Notably, metaVF failed to identify any VFs in five of the ten pathogenic reference strains tested, highlighting its limited ability to detect VFs and demonstrating the advantages of machine-learning-based models over homology-based approaches. While VirulentHunter produced substantially more hits than metaVF, yielding predictions comparable in number to PathoFact 2.0, PathoFact 2.0 generally identified more VFs overall (Figure 7, Supplementary Figures S6–S8). Exceptions were observed for non-pathogenic strains *Bifidobacterium animalis*, *Bifidobacterium bifidum*, *Heyndrickxia coagulans*, and for the pathogenic *Ralstonia mannitolilytica strain Guangzhou-RMAB10*, where VirulentHunter predicted slightly more VFs (Supplementary Figures S6 and S8).

Figure 7. Performance comparison of virulence factor prediction tools in pathogenic (A) and non-pathogenic (B) strains. Venn diagrams comparing the predictions of PathoFact 2.0, PathoFact, metaVF, and VirulentHunter in predicting virulence factors (VFs).

In addition, the PathoFact 2.0 toxin-associated module was compared to PathoFact 1.0 as well as CSM-Toxin and ToxinPred2 (Figure 8, Supplementary Figures S1B, S2B and S9). As CSM-Toxin and ToxinPred2 do not accept contig sequences as input, protein sequences were first predicted from contigs using Pyrodigal-gv. PathoFact 2.0 predicted more toxin-associated proteins than PathoFact 1.0, demonstrating improved detection capacity. Compared to external tools, CSM-Toxin identified substantially fewer toxins across the tested reference strains. In contrast, ToxinPred2 predicted a comparable number of toxins overall. However, for non-pathogenic strains *Bifidobacterium bifidum*, *Heyndrickxia coagulans*, *Lactobacillus acidophilus*, and for pathogenic strains *Streptococcus gallolyticus subsp. gallolyticus* and Streptococcus parasuis B26, ToxinPred2 identified more predicted toxins than PathoFact 2.0 (Supplementary Figures S2B and S9).

Figure 8. Performance comparison of toxin prediction tools in pathogenic (A) and non-pathogenic (B) strains. Venn diagrams comparing the predictions of PathoFact 2.0, PathoFact 1.0, CSM-Toxin, and ToxinPred2 in predicting Toxins and Toxin-associated proteins.

PathoFact 2.0 Output Structure

PathoFact 2.0 creates a structured output directory that summarises predictions from all analysis modules, including VFs, toxin-associated proteins, ARGs, MGEs and BGCs. Each module generates dedicated result files corresponding to the underlying prediction tools (Supplementary File S1).

The primary summary file, combined_report.tsv, provides an integrated overview of high-confidence predictions across all modules in a tabular format. This table includes key information such as protein identifiers, bit scores (from HMM profiles), machine-learning prediction scores, and outputs from DeepARG, RGI, SignalP, GenoMad, and antiSMASH, thereby supporting downstream interpretation and candidate prioritisation.

Proteins with prediction probabilities below user-defined thresholds but containing conserved domains identified by toxin-associated or VF HMM profiles are reported in ambiguous_TOX_hits_rep_prot.tsv and ambiguous_VF_hits_rep_prot.tsv. These lower-confidence candidates may warrant further investigation in comparative or experimental analyses.

High-confidence predictions are reported in amr_hits_rep_prot.tsv, TOX_hits_rep_prot.tsv, and VF_hits_rep_prot.tsv, which summarise features exceeding user-defined probability thresholds and include protein identifiers, bit scores, machine-learning predictions, signal peptide predictions, and genomic context information, such as association with prophages or plasmids identified by GenoMad.

In addition, PathoFact 2.0 generates a dedicated Group_of_sequence directory containing FASTA files of representative protein sequences grouped by functional category (VFs, toxin-associated proteins, antimicrobial resistance genes, and combined hits), together with conserved domain (CDD) annotation tables for predicted VFs and toxin-associated proteins. These files are designed to facilitate downstream analyses, including comparative genomics and functional characterisation.

Limitations of PathoFact 2.0

It is well established that non-pathogenic bacterial strains can also carry genes annotated as VFs or toxins [7]. Consequently, PathoFact 2.0 is most effective as an initial screening tool to identify potential candidates, which can then be examined in comparative studies to distinguish confirmed pathogenic cases from controls. The pipeline provides a probability score indicating whether a protein is likely to be VF- or toxin-associated; however, establishing a definitive link between predicted candidates and infectious disease requires experimental validation.

PathoFact 2.0 does not directly classify specific VF or toxin types (e.g., adhesins or genotoxins). Instead, it reports detailed annotations of conserved protein domains from CDD [18], allowing users to infer functional roles when available. This design emphasises contextual interpretation rather than categorical assignment.

The inclusion of housekeeping genes from non-pathogenic microorganisms reflects a deliberate methodological choice rather than a limitation. Multiple well-characterised housekeeping proteins have been shown to exhibit virulence-associated “moonlighting” functions in pathogenic bacteria, including roles in adhesion, immune modulation, and tissue invasion [57,58]. Notable examples include glyceraldehyde-3-phosphate dehydrogenase (VF0015 in VFDB) [59], enolase [60,61], elongation factor Tu (VF0460 in VFDB) [62,63], GroEL [61,64,65], and DnaK [61,66]. This approach maintains a biologically realistic negative dataset while reducing the risk of misclassifications within the intended scope of PathoFact 2.0.

PathoFact 2.0 is designed for metagenomic samples; most prediction modules and phenotypes are bacterial-centric. Virulence, toxin-associated, and antimicrobial resistance predictions are particularly interpreted in the context of human pathogens.

Conclusions

ARGs, VFs, and toxins represent major threats to global health. Therefore, accurate detection of these elements is crucial for assessing the presence and potential risks of pathogenic microorganisms in microbiomes and for identifying reservoirs of pathogenicity. Our improved pipeline, PathoFact 2.0, offers significant improvements over PathoFact (its predecessor), ToxinPred2, CSM-toxin, VirulentHunter, and metaVF. SignalP has been upgraded and made optional to further optimise performance, providing users with flexibility based on their requirements. Additionally, antiSMASH 7.0 facilitates the prediction of BGCs, recognising emerging evidence that some BGC-encoded factors might increase virulence. Furthermore, we have integrated geNomad, a cutting-edge tool for identifying MGEs, including plasmids and phages linked to ARGs, VFs, toxins and toxin-associated proteins across various bacterial species. The PathoFact 2.0 update improves the accuracy and sensitivity of analyses while enhancing computational efficiency.

PathoFact 2.0 represents a major advance in metagenomic analysis by integrating the detection of ARGs, VFs, toxins and toxin-associated proteins, signal peptides, MGEs, and BGCs within a single, streamlined pipeline. Unlike existing tools that focus on individual aspects of pathogenicity, PathoFact 2.0 provides a comprehensive, multi-layered view that captures both gene presence and genomic context, improving interpretability and enabling a holistic assessment of microbial pathogenic potential.

Methods

Databases used for the PathoFact 2.0 Dataset Construction

- SwissProt [31] is the expertly curated part of UniProtKB [67]. It offers high-quality protein sequences with detailed functional annotations, including keywords for pathogenesis, virulence, toxins, and antibiotic resistance.
- VFDB [44], the Virulence Factor Database, is a comprehensive reference for curating information on virulence factors of bacterial pathogens.
- T3DB [12], the toxin and toxin-target database, is a resource cataloguing thousands of toxins and their protein targets, with detailed mechanisms, structures, and toxicity data, including bacterial protein toxins.
- DBETH [13], the database for bacterial exotoxins, is a specialised database of bacterial exotoxins pathogenic to humans, classified into 24 mechanistic and activity types from 26 bacterial genera.
- TADB [37], the toxin-antitoxin database, is a repository of bacterial toxin-antitoxin loci across types I-VIII, including experimentally validated pairs, predicted loci, and associations with mobile genetic elements.
- SecReT6 [38] is a database containing known and predicted type VI secretion systems, including effectors, immunity proteins, regulators, and accessory proteins from bacterial genomes.
- PAT [39] is the prokaryotic antimicrobial toxin database and contains a collection of antimicrobial toxins, including bacteriocins and effectors from secretion systems.

Clustering Parameters

MMseqs2 [33] was used for dereplication; sequences were clustered at 100% identity and 100% coverage using the parameters -c 1.0 and --min-seq-id 1.0. It removes exact duplicates and retains representative sequences to generate non-redundant datasets. To create the test subsets, a coverage of 80% (-c 0.8) and identities ranging from 40 - 100% (--min-seq-id 0.4, --min-seq-id 0.6, --min-seq-id 0.8, --min-seq-id 1.0) were used, and then sequences with similarity higher than min-seq-id were removed for each test subset. In all cases, the cluster mode and coverage mode used were 0 (--cov-mode 0 --cluster-mode 0). When --cov-mode 0 is specified in combination with −c values ranging from 0.0 to 1.0, sequences are assigned to the same cluster only if the alignment spans at least a fraction *c* of the length of the longer sequence. According to the developers of MMSeq2, this coverage criterion is particularly suitable for clustering full-length protein sequences [33].

Protein Composition Features

Protein sequence composition features were extracted to represent each protein as fixed-length vectors derived from its primary amino acid sequence [36]. These included amino acid composition

(AAC), dipeptide composition (DPC), composition (CTDC), transition (CTDT) and distribution (CTDD). AAC captures the relative frequency of each of the 20 amino acids in a sequence, whereas DPC captures the relative frequency of all adjacent amino-acid pairs (400 possible dipeptides). CTDC represents the percentage of amino acids belonging to each of three predefined groups (polar, neutral, hydrophobic) in the entire protein sequence. CTDT represents the percentage frequency with which a residue of one group is followed by a residue of a different group along the sequence. CTDD represents the distribution of each amino acid group, measuring the spatial position, where the first, 25%, 50%, 75% and 100% of the residues of a specific class are located.

Performance and Evaluation Metrics

To assess the models' performance, we used a confusion matrix comprising true positives (TP), true negatives (TN), false positives (FP), and false negatives (FN), computed on the test datasets described above. TP corresponds to truly positive instances correctly predicted as positive by the model, whereas TN corresponds to truly negative instances correctly predicted as negative. FP are truly negative instances incorrectly predicted as positive, and FN are truly positive instances incorrectly predicted as negative. From these values, we calculated the following metrics:

$$Accuracy = (TP + TN)/(TP + TN + FP + FN)$$

$$Precision = TP/(TP + FP)$$

$$Recall = TP/(TP + FN)$$

$$MCC = (TP \times TN - FP \times FN)/\sqrt{(TP + FP)(TP + FN)(TN + FP)(TN + FN)}$$

HMM Profiles

Profile hidden Markov models are probabilistic models built from a multiple sequence alignment that encode, for each alignment position, the position-specific probabilities of residues and insertions/deletions, turning the alignment into a position-specific scoring system for detecting homologous sequences [68]. The FASTA files of conserved-domain multiple sequence alignments for each CDD [26] family (https://ftp.ncbi.nih.gov/pub/mmdb/cdd/fasta.tar.gz) were downloaded. Pyhmmer v0.10.14 [69] was used to obtain HMM profiles for each CDD family and to perform protein sequence searches against the CDD HMM family profiles.

Benchmarking Datasets

This study utilised publicly available datasets containing complete genomes from pathogenic and non-pathogenic bacteria, including various *Escherichia coli* strains, from NCBI. The accession numbers of the bacteria used are indicated in Supplementary Table S2.

Availability of Supporting Source Code and Requirements

Project name: Pathofact 2.0
Project homepage: https://gitlab.com/uniluxembourg/lcsb/systems-ecology/pathofact2
Operating system(s): Linux

Programming language: Python, R, bash
Other requirements: Snakemake, Mamba, conda.
License: GNU General License v3.0 or later
Biotools ID: pathofact2
RRID: SCR_027650
workflowhubDOI: 10.48546/workflowhub.workflow.2087.1

Additional Files

Supplementary Table S1. List of microorganisms non-pathogenic to humans and their total protein count obtained from the NCBI Database.

Supplementary Table S2. Bacterial strains used in this study, including their classification as pathogenic or non-pathogenic, species/strain information, genome assembly or reference version, and corresponding accession numbers.

Supplementary Table S3. Evaluation of the PathoFact 2.0 toxin-associated protein prediction module. The table presents performance across test subsets defined by sequence similarity to the training set. Metrics reported include class distributions (Negative, non-toxin; Positive, toxin-associated), confusion matrix counts (true negatives, false positives, true positives, false negatives), and performance measures (accuracy, precision, recall, F1 score, Matthews correlation coefficient).

Supplementary Table S4. Evaluation of the PathoFact 2.0 virulence factor prediction module. The table summarises performance across test subsets defined by sequence similarity to the training set. Reported metrics include class distributions (Negative, non-VF; Positive, VF), confusion matrix counts, and performance measures (accuracy, precision, recall, F1 score, Matthews correlation coefficient).

Supplementary Table S5. Comparison of virulence factor prediction performance for PathoFact 2.0 at varying probability cutoffs and for VirulenHunter, evaluated across test subsets stratified by sequence similarity to the training set. The table reports class distributions, confusion matrix counts, and performance metrics (accuracy, precision, recall, F1 score, Matthews correlation coefficient).

Supplementary Table S6. Comparison of toxin protein prediction performance for PathoFact 2.0 at different prediction probability cutoffs and for ToxinPred2. The table reports the number of proteins classified as Negative (non-toxin) and Positive (toxin), confusion matrix counts, and associated performance metrics (accuracy, precision, recall, F1 score, and Matthews correlation coefficient).

Supplementary Figure S1. Performance comparison of PathoFact 2.0 versus PathoFact for ARG and toxin prediction in Escherichia coli. A) Comparative performance of PathoFact 2.0 (blue) versus PathoFact (green) in predicting antimicrobial resistance genes (ARGs) in non-pathogenic (left) and pathogenic (right) *E. coli* strains. **B)** Comparative performance of PathoFact 2.0 (blue) versus PathoFact (green) in predicting toxin-associated proteins in non-pathogenic (left) and pathogenic (right) *E. coli* strains. The top panel shows the total number of predicted toxin-associated proteins, while the bottom panel shows those that contain signal peptides, as identified by SignalP.

Supplementary Figure S2. Performance comparison of PathoFact 2.0 versus PathoFact for ARG and toxin prediction in bacterial strains. A) Comparative performance of PathoFact 2.0 (blue) versus PathoFact (green) in predicting antimicrobial resistance genes (ARGs) in non-pathogenic (left) and pathogenic (right) bacterial strains. **B)** Comparative performance of PathoFact 2.0 (blue) versus PathoFact (green) in predicting toxin-associated proteins in non-pathogenic (left) and pathogenic (right) bacterial strains. The top panel shows the total number of predicted toxin-associated proteins, while the bottom panel shows those that contain signal peptides, as identified by SignalP.

Supplementary Figure S3. Area under the receiver operating characteristic (AUROC) curves comparing the virulence factor prediction performance of PathoFact 2.0 (A) and Virulent Hunter (B) on the benchmark datasets. Curves represent performance across different benchmark subsets: 40 (red), 60 (yellow), 80 (green), 100 (blue) and the complete dataset (All, purple). AUROC curves are not available with PathoFact 1.0 because it uses a combination of machine learning and HMM classification. MetaVF is an alignment-based algorithm, and it does not generate probability scores; therefore, AUROC curves are not possible.

Supplementary Figure S4. Area under the receiver operating characteristic (AUROC) curves comparing the virulence factor prediction performance of PathoFact 2.0 (A) and CSM-toxin (B) on the benchmark dataset. Curves correspond to PathoFact 2.0 (blue) and CSM-toxin (red). AUROC curves are not possible with Toxinpred2, since its predictions are based on a combination of factors rather than on machine-learning probabilities alone. Nor are they possible with PathoFact 1.0, which also uses a combination of machine learning and HMM classification.

Supplementary Figure S5. Comparative analysis of virulence factor profiles in non-pathogenic and pathogenic bacterial strains. Bar charts represent the distribution of predicted toxin-associated (A) and virulence-associated (B) proteins across non-pathogenic (left panel) and pathogenic (right panel) strains. The categories include total predicted virulence factors/toxin-associated proteins (dark blue), associated with plasmid markers (red), phage markers (Green), and proteins predicted by SignalP to be secreted (light blue). Additional categories include toxin-associated virulence factors (TOX-related ∩ VF, grey) and biosynthetic gene clusters overlapping (purple). Numerical values above each bar indicate the total count of proteins identified in each category for the corresponding strain.

Supplementary Figure S6. Venn diagrams comparing the predictions of PathoFact 2.0, PathoFact, metaVF, and VirulentHunter in predicting virulence factors in pathogenic (A) and non-pathogenic (B) strains.

Supplementary Figure S7. Comparative performance of Pathofact 2.0 (blue) versus PathoFact1 (green) and metaVF (red) in predicting virulence factors (VFs) in non-pathogenic (left) and pathogenic (right) *Escherichia coli* strains. The top panel shows the total number of predicted VFs. The second panel depicts the subset predicted to be secreted. The third panel shows VFs predicted to be plasmid-encoded, while the fourth panel presents those predicted to be prophage-associated.

Supplementary Figure S8. Comparative performance of Pathofact 2.0 (blue) versus PathoFact (green) and metaVF (red) in predicting virulence factors (VFs) in non-pathogenic (left) and pathogenic (right) bacterial strains. The top panel shows the total number of predicted VFs. The second panel depicts the subset predicted to be secreted. The third panel shows VFs predicted to be plasmid-encoded, while the fourth panel presents those predicted to be prophage-associated.

Supplementary Figure S9. Venn diagrams comparing the predictions of PathoFact 2.0, PathoFact, Toxinpred2, and CSM-Toxin in predicting toxins and toxin-associated proteins in pathogenic (A) and non-pathogenic (B) strains.

Abbreviations

AAC, amino acid composition; ARO, antibiotic resistance ontology; ARGs, antimicrobial resistance genes; BGCs, biosynthetic gene clusters; CDD, conserved domains database; CTDC, (Composition, Transition, Distribution)-composition; CTDD, (Composition, Transition, Distribution)-distribution; CTDT, (Composition, Transition, Distribution)-transition; DBETH, database for bacterial exotoxins; DPC,

dipeptide composition; HMMs, hidden Markov models; MCC, Matthews correlation coefficient; MGEs, mobile genetic elements; ML, machine learning; ORF, open reading frame; PAT, prokaryotic antimicrobial toxins database; RF, random forest; SM, specialised metabolites; SMOTE, synthetic minority oversampling technique; SNP, single nucleotide polymorphisms; T3DB, toxin exposome database; VFDB, virulence factor database; VFs, virulence factors.

Acknowledgements

The experiments presented in this paper were carried out using the HPC facilities of the University of Luxembourg [70]. The manuscript also passed the Luxembourg Centre for Systems Biomedicine internal pre-publication check designed to ensure FAIRness and reproducibility.

Author Contributions

P.W. initiated the study, which involved the overall design and objective, and was led by L.F.D. and J.O.S. in the development of PathoFact 2.0. O.H. contributed to early brainstorming discussions on workflow design and database strategy. P.M. and C.C.L. contributed to the overall discussions. L.F.D. and J.O.S. wrote the draft manuscript. All authors read and commented on the manuscript.

Funding

This work has been supported by the Pélican grant from the Mie and Pierre Hippert-Faber Pélican Foundation under the aegis of Fondation de Luxembourg to JOS, as well as by the Luxembourg National Research Fund (FNR CORE/23/BM/15886415) and the European Research Council (ERC-CoG 863664) to PW. The Luxembourg Government further supported the work through the CoVaLux program.
This research was funded in whole, or in part, by the Luxembourg National Research Fund (FNR), grant reference (FNR CORE/23/BM/15886415). For the purpose of open access, and in fulfilment of the obligations arising from the grant agreement, the author has applied a Creative Commons Attribution 4.0 International (CC BY 4.0) license to any Author Accepted Manuscript version arising from this submission.

Data Availability

Pathofact 2.0 is accessible at https://gitlab.com/uniluxembourg/lcsb/systems-ecology/pathofact2. Additionally, the core databases required to run the pipeline can be found at https://zenodo.org/records/14192463. The ML datasets used for training, validation, and benchmarking of PathoFact 2.0 can be found in https://zenodo.org/records/17647372.

Competing Interests

None declared.

References

1. Hou K, Wu Z-X, Chen X-Y, Wang J-Q, Zhang D, Xiao C, et al.. Microbiota in health and diseases. *Signal Transduct Target Ther*. Springer Science and Business Media LLC; 2022; doi: 10.1038/s41392-022-00974-4.

2. Inda-Díaz JS, Lund D, Parras-Moltó M, Johnning A, Bengtsson-Palme J, Kristiansson E. Latent antibiotic resistance genes are abundant, diverse, and mobile in human, animal, and environmental microbiomes. *Microbiome*. Springer Science and Business Media LLC; 2023; doi: 10.1186/s40168-023-01479-0.

3. Beceiro A, Tomás M, Bou G. Antimicrobial resistance and virulence: a successful or deleterious association in the bacterial world? *Clin Microbiol Rev*. American Society for Microbiology; 2013; doi: 10.1128/CMR.00059-12.

4. Zhu C, Wu L, Ning D, Tian R, Gao S, Zhang B, et al.. Global diversity and distribution of antibiotic resistance genes in human wastewater treatment systems. *Nat Commun*. Springer Science and Business Media LLC; 2025; doi: 10.1038/s41467-025-59019-3.

5. Jian Z, Zeng L, Xu T, Sun S, Yan S, Yang L, et al.. Antibiotic resistance genes in bacteria: Occurrence, spread, and control. *J Basic Microbiol*. Wiley; 2021; doi: 10.1002/jobm.202100201.

6. Alcock BP, Huynh W, Chalil R, Smith KW, Raphenya AR, Wlodarski MA, et al.. CARD 2023: expanded curation, support for machine learning, and resistome prediction at the Comprehensive Antibiotic Resistance Database. *Nucleic Acids Res*. Oxford University Press (OUP); 2023; doi: 10.1093/nar/gkac920.

7. Niu C, Yu D, Wang Y, Ren H, Jin Y, Zhou W, et al.. Common and pathogen-specific virulence factors are different in function and structure. *Virulence*. Informa UK Limited; 2013; doi: 10.4161/viru.25730.

8. Sharma AK, Dhasmana N, Dubey N, Kumar N, Gangwal A, Gupta M, et al.. Bacterial virulence factors: Secreted for survival. *Indian J Microbiol*. 2017; doi: 10.1007/s12088-016-0625-1.

9. Blair JMA, Webber MA, Baylay AJ, Ogbolu DO, Piddock LJV. Molecular mechanisms of antibiotic resistance. *Nat Rev Microbiol*. Springer Science and Business Media LLC; 2015; doi: 10.1038/nrmicro3380.

10. Rodríguez-Beltrán J, DelaFuente J, León-Sampedro R, MacLean RC, San Millán Á. Beyond horizontal gene transfer: the role of plasmids in bacterial evolution. *Nat Rev Microbiol*. Springer Science and Business Media LLC; 2021; doi: 10.1038/s41579-020-00497-1.

11. Galanos C, Freudenberg MA. Bacterial endotoxins: biological properties and mechanisms of action. *Mediators Inflamm*. Wiley; 1993; doi: 10.1155/S0962935193000687.

12. Wishart D, Arndt D, Pon A, Sajed T, Guo AC, Djoumbou Y, et al.. T3DB: the toxic exposome database. *Nucleic Acids Res*. Oxford University Press (OUP); 2015; doi: 10.1093/nar/gku1004.

13. Chakraborty A, Ghosh S, Chowdhary G, Maulik U, Chakrabarti S. DBETH: A database of Bacterial ExoToxins for human. *Nucleic Acids Res*. Oxford University Press (OUP); 2012; doi: 10.1093/nar/gkr942.

14. Green ER, Mecsas J. Bacterial secretion systems: An overview. *Microbiol Spectr*. 2016; doi: 10.1128/microbiolspec.VMBF-0012-2015.

15. Kaushik S, He H, Dalbey RE. Bacterial signal peptides- navigating the journey of proteins. *Front Physiol*. Frontiers Media SA; 2022; doi: 10.3389/fphys.2022.933153.

16. Lybbert AC, Williams JL, Raghuvanshi R, Jones AD, Quinn RA. Mining public mass spectrometry data to characterize the diversity and ubiquity of P. aeruginosa specialized metabolites. *Metabolites*. MDPI AG; 2020; doi: 10.3390/metabo10110445.

17. Elshafie HS, Camele I. An overview of metabolic activity, beneficial and pathogenic aspects of Burkholderia spp. *Metabolites*. MDPI AG; 2021; doi: 10.3390/metabo11050321.

18. Lau GW, Hassett DJ, Ran H, Kong F. The role of pyocyanin in Pseudomonas aeruginosa infection. *Trends Mol Med*. Elsevier BV; 2004; doi: 10.1016/j.molmed.2004.10.002.

19. Ambassadors G, Patrons: World leaders commit to decisive action on antimicrobial resistance. UN Environment. https://www.unep.org/news-and-stories/press-release/world-leaders-commit-decisive-action-antimicrobial-resistance (2024). Accessed 2025 Oct 7.

20. Environment UN: Antimicrobial Resistance (AMR). UNEP - UN Environment Programme. https://www.unep.org/topics/chemicals-and-pollution-action/pollution-and-health/antimicrobial-resistance-amr (2024). Accessed 2025 Oct 7.

21. Bansal MA, Sharma DR, Kathuria DM. A systematic review on data scarcity problem in deep learning: Solution and applications. *ACM Comput Surv*. Association for Computing Machinery (ACM); 2022; doi: 10.1145/3502287.

22. de Nies L, Lopes S, Busi SB, Galata V, Heintz-Buschart A, Laczny CC, et al.. PathoFact: a pipeline for the prediction of virulence factors and antimicrobial resistance genes in metagenomic data. *Microbiome*. Springer Science and Business Media LLC; 2021; doi: 10.1186/s40168-020-00993-9.

23. Dong W, Fan X, Guo Y, Wang S, Jia S, Lv N, et al.. An expanded database and analytical toolkit for identifying bacterial virulence factors and their associations with chronic diseases. *Nat Commun*. Springer Science and Business Media LLC; 2024; doi: 10.1038/s41467-024-51864-y.

24. Ji B, Pi W, Liu W, Liu Y, Cui Y, Zhang X, et al.. HyperVR: a hybrid deep ensemble learning approach for simultaneously predicting virulence factors and antibiotic resistance genes. *NAR Genom Bioinform*. 2023; doi: 10.1093/nargab/lqad012.

25. Rathore AS, Choudhury S, Arora A, Tijare P, Raghava GPS. ToxinPred 3.0: An improved method for predicting the toxicity of peptides. *Comput Biol Med*. Elsevier BV; 2024; doi: 10.1016/j.compbiomed.2024.108926.

26. Kasmanas JC, Magnúsdóttir S, Zhang J, Smalla K, Schloter M, Stadler PF, et al.. Integrating comparative genomics and risk classification by assessing virulence, antimicrobial resistance, and plasmid spread in microbial communities with gSpreadComp. *Gigascience*. 2025; doi: 10.1093/gigascience/giaf072.

27. Wang J, Chitsaz F, Derbyshire MK, Gonzales NR, Gwadz M, Lu S, et al.. The conserved domain database in 2023. *Nucleic Acids Res*. Oxford University Press (OUP); 2023; doi: 10.1093/nar/gkac1096.

28. Blin K, Shaw S, Augustijn HE, Reitz ZL, Biermann F, Alanjary M, et al.. antiSMASH 7.0: new and improved predictions for detection, regulation, chemical structures and visualisation. *Nucleic Acids Res*. Oxford University Press (OUP); 2023; doi: 10.1093/nar/gkad344.

29. . Pyrodigal: Python bindings and interface to Prodigal, an efficient method for gene prediction in prokaryotes. *Journal of Open Source Software*. doi: 10.21105/joss.04296.

30. Camargo AP, Roux S, Schulz F, Babinski M, Xu Y, Hu B, et al.. Identification of mobile genetic elements with geNomad. *Nat Biotechnol*. Springer Science and Business Media LLC; 2024; doi: 10.1038/s41587-023-01953-y.

31. Köster J, Rahmann S. Snakemake--a scalable bioinformatics workflow engine. *Bioinformatics*.

Oxford University Press (OUP); 2012; doi: 10.1093/bioinformatics/bts480.

32. UniProt Consortium. UniProt: The universal protein knowledgebase in 2023. *Nucleic Acids Res*. Oxford University Press (OUP); 2023; doi: 10.1093/nar/gkac1052.

33. Steinegger M, Söding J. MMseqs2 enables sensitive protein sequence searching for the analysis of massive data sets. *Nat Biotechnol*. 2017; doi: 10.1038/nbt.3988.

34. Chawla NV, Bowyer KW, Hall LO, Kegelmeyer WP. SMOTE: Synthetic minority over-sampling technique. *J Artif Intell Res*. AI Access Foundation; 2002; doi: 10.1613/jair.953.

35. Pedregosa F, Varoquaux G, Gramfort A, Michel V, Thirion B, Grisel O, et al.. Scikit-learn: Machine Learning in Python. arXiv [cs.LG].

36. Chen Z, Zhao P, Li F, Leier A, Marquez-Lago TT, Wang Y, et al.. iFeature: a Python package and web server for features extraction and selection from protein and peptide sequences. *Bioinformatics*. 2018; doi: 10.1093/bioinformatics/bty140.

37. Guan J, Chen Y, Goh Y-X, Wang M, Tai C, Deng Z, et al.. TADB 3.0: an updated database of bacterial toxin-antitoxin loci and associated mobile genetic elements. *Nucleic Acids Res*. Oxford University Press (OUP); 2024; doi: 10.1093/nar/gkad962.

38. Zhang J, Guan J, Wang M, Li G, Djordjevic M, Tai C, et al.. SecReT6 update: a comprehensive resource of bacterial Type VI Secretion Systems. *Sci China Life Sci*. Springer Science and Business Media LLC; 2023; doi: 10.1007/s11427-022-2172-x.

39. Liu Y, Liu S, Pan Z, Ren Y, Jiang Y, Wang F, et al.. PAT: a comprehensive database of prokaryotic antimicrobial toxins. *Nucleic Acids Res*. Oxford University Press (OUP); 2023; doi: 10.1093/nar/gkac879.

40. Harms A, Liesch M, Körner J, Québatte M, Engel P, Dehio C. A bacterial toxin-antitoxin module is the origin of inter-bacterial and inter-kingdom effectors of Bartonella. *PLoS Genet*. 2017; doi: 10.1371/journal.pgen.1007077.

41. Yadav SK, Magotra A, Ghosh S, Krishnan A, Pradhan A, Kumar R, et al.. Immunity proteins of dual nuclease T6SS effectors function as transcriptional repressors. *EMBO Rep*. EMBO; 2021; doi: 10.15252/embr.202051857.

42. Danov A, Segev O, Bograd A, Ben Eliyahu Y, Dotan N, Kaplan T, et al.. Toxinome-the bacterial protein toxin database. *MBio*. 2024; doi: 10.1128/mbio.01911-23.

43. Sordo M, Zeng Q. On sample size and classification accuracy: A performance comparison. *Biological and Medical Data Analysis*. Berlin, Heidelberg: Springer Berlin Heidelberg;

44. Liu B, Zheng D, Zhou S, Chen L, Yang J. VFDB 2022: a general classification scheme for bacterial virulence factors. *Nucleic Acids Res*. Oxford University Press (OUP); 2022; doi: 10.1093/nar/gkab1107.

45. Arango-Argoty G, Garner E, Pruden A, Heath LS, Vikesland P, Zhang L. DeepARG: a deep learning approach for predicting antibiotic resistance genes from metagenomic data. *Microbiome*. 2018; doi: 10.1186/s40168-018-0401-z.

46. Feldgarden M, Brover V, Gonzalez-Escalona N, Frye JG, Haendiges J, Haft DH, et al.. AMRFinderPlus and the Reference Gene Catalog facilitate examination of the genomic links among antimicrobial resistance, stress response, and virulence. *Sci Rep*. Springer Science and Business Media LLC; 2021; doi:

10.1038/s41598-021-91456-0.

47. Ugarcina Perovic S, Ramji V, Chong H, Duan Y, Maguire F, Coelho LP. argNorm: normalization of antibiotic resistance gene annotations to the Antibiotic Resistance Ontology (ARO). *Bioinformatics*. Oxford University Press (OUP); 2025; doi: 10.1093/bioinformatics/btaf173.

48. Chicco D, Jurman G. The advantages of the Matthews correlation coefficient (MCC) over F1 score and accuracy in binary classification evaluation. *BMC Genomics*. Springer Science and Business Media LLC; 2020; doi: 10.1186/s12864-019-6413-7.

49. Chen C, Xu Y, Ouyang J, Xiong X, Łabaj PP, Chmielarczyk A, et al.. VirulentHunter: deep learning-based virulence factor predictor illuminates pathogenicity in diverse microbial contexts. *Brief Bioinform*. Oxford University Press (OUP); 2025; doi: 10.1093/bib/bbaf271.

50. Gupta A, Malwe AS, Srivastava GN, Thoudam P, Hibare K, Sharma VK. MP4: a machine learning based classification tool for prediction and functional annotation of pathogenic proteins from metagenomic and genomic datasets. *BMC Bioinformatics*. Springer Science and Business Media LLC; 2022; doi: 10.1186/s12859-022-05061-7.

51. Sharma A, Garg A, Ramana J, Gupta D. VirulentPred 2.0: An improved method for prediction of virulent proteins in bacterial pathogens. *Protein Sci*. Wiley; 2023; doi: 10.1002/pro.4808.

52. Xie R, Li J, Wang J, Dai W, Leier A, Marquez-Lago TT, et al.. DeepVF: a deep learning-based hybrid framework for identifying virulence factors using the stacking strategy. *Brief Bioinform*. Oxford University Press (OUP); 2021; doi: 10.1093/bib/bbaa125.

53. Sharma N, Naorem LD, Jain S, Raghava GPS. ToxinPred2: an improved method for predicting toxicity of proteins. *Brief Bioinform*. Oxford University Press (OUP); 2022; doi: 10.1093/bib/bbac174.

54. Morozov V, Rodrigues CHM, Ascher DB. CSM-Toxin: A web-server for predicting protein toxicity. *Pharmaceutics*. MDPI AG; 2023; doi: 10.3390/pharmaceutics15020431.

55. Kaper JB, Nataro JP, Mobley HL. Pathogenic Escherichia coli. *Nat Rev Microbiol*. Springer Science and Business Media LLC; 2004; doi: 10.1038/nrmicro818.

56. Johnson TJ, Nolan LK. Pathogenomics of the virulence plasmids of Escherichia coli. *Microbiol Mol Biol Rev*. American Society for Microbiology; 2009; doi: 10.1128/MMBR.00015-09.

57. Henderson B, Martin A. Bacterial virulence in the moonlight: multitasking bacterial moonlighting proteins are virulence determinants in infectious disease. *Infect Immun*. American Society for Microbiology; 2011; doi: 10.1128/IAI.00179-11.

58. Henderson B, Martin A. Bacterial moonlighting proteins and bacterial virulence. *Curr Top Microbiol Immunol*. 2013; doi: 10.1007/82_2011_188.

59. Pancholi V, Fischetti VA. A major surface protein on group A streptococci is a glyceraldehyde-3-phosphate-dehydrogenase with multiple binding activity. *J Exp Med*. Rockefeller University Press; 1992; doi: 10.1084/jem.176.2.415.

60. Henderson B, Martin A. Bacterial moonlighting proteins and bacterial virulence. *Curr Top Microbiol Immunol*. Curr Top Microbiol Immunol; 2013; doi: 10.1007/82_2011_188.

61. Henderson B, Martin A. Bacterial virulence in the moonlight: multitasking bacterial moonlighting proteins are virulence determinants in infectious disease. *Infect Immun*. American Society for

Microbiology; 2011; doi: 10.1128/IAI.00179-11.

62. Barel M, Charbit A. Detection of the interaction between host and bacterial proteins: eukaryotic nucleolin interacts with Francisella elongation factor Tu. *Methods Mol Biol*. Methods Mol Biol; 2014; doi: 10.1007/978-1-4939-1261-2_7.

63. Granato D, Bergonzelli GE, Pridmore RD, Marvin L, Rouvet M, Corthésy-Theulaz IE. Cell surface-associated elongation factor Tu mediates the attachment of Lactobacillus johnsonii NCC533 (La1) to human intestinal cells and mucins. *Infection and immunity*. Infect Immun; 2004; doi: 10.1128/iai.72.4.2160-2169.2004.

64. Kamiya S, Yamaguchi H, Osaki T, Taguchi H. A virulence factor of Helicobacter pylori: role of heat shock protein in mucosal inflammation after H. pylori infection. *J Clin Gastroenterol*. 1998; doi: 10.1097/00004836-199800001-00007.

65. Hickey TBM, Ziltener HJ, Speert DP, Stokes RW. Mycobacterium tuberculosis employs Cpn60.2 as an adhesin that binds CD43 on the macrophage surface: M. tuberculosis Cpn60.2 mediates macrophage binding via CD43. *Cell Microbiol*. Hindawi Limited; 2010; doi: 10.1111/j.1462-5822.2010.01496.x.

66. Lehner T, Bergmeier LA, Wang Y, Tao L, Sing M, Spallek R, et al.. Heat shock proteins generate β-chemokines which function as innate adjuvants enhancing adaptive immunity. *Eur J Immunol*. 2000; doi: 10.1002/1521-4141(200002)30:2<594::AID-IMMU594>3.0.CO;2-1.

67. UniProt Consortium. UniProt: The universal protein knowledgebase in 2025. *Nucleic Acids Res*. Oxford University Press (OUP); 2025; doi: 10.1093/nar/gkae1010.

68. Eddy SR. Profile hidden Markov models. *Bioinformatics*. Oxford University Press (OUP); 1998; doi: 10.1093/bioinformatics/14.9.755.

69. Larralde M, Zeller G. PyHMMER: a Python library binding to HMMER for efficient sequence analysis. *Bioinformatics*. Oxford Academic; 2023; doi: 10.1093/bioinformatics/btad214.

70. Varrette S, Cartiaux H, Peter S, Kieffer E, Valette T, Olloh A. Management of an academic HPC & research computing facility: The ULHPC experience 2.0. *Proceedings of the 2022 6th High Performance Computing and Cluster Technologies Conference*. New York, NY, USA: ACM; doi: https://doi.org/10.1145/3560442.3560445

 Click here to access/download;Table;Table1.pdf

Table 1. Runtime Comparison of PathoFact 2.0 and VirulentHunter

Number of protein sequences	VirulentHunter	PathoFact 2.0				
	1 GPU	1 CPU	2 CPU	4 CPU	6 CPU	8 CPU
500	2 min 21 s	3.7 s	2.6 s	2.0 s	1.9 s	1.8 s
5500	25 min 42 s	29.9 s	17.4 s	10.2 s	8.1 s	7.4 s
10000	54 min 11 s	55.4 s	29.3 s	16.7 s	13.1 s	11.5 s
30000	2 h 45 min 50 s	2 min 45 s	1 min 32.9 s	52.1 s	40.3 s	36.0 s

Figure 1

Click here to access/download;Figure;Figure1.pdf

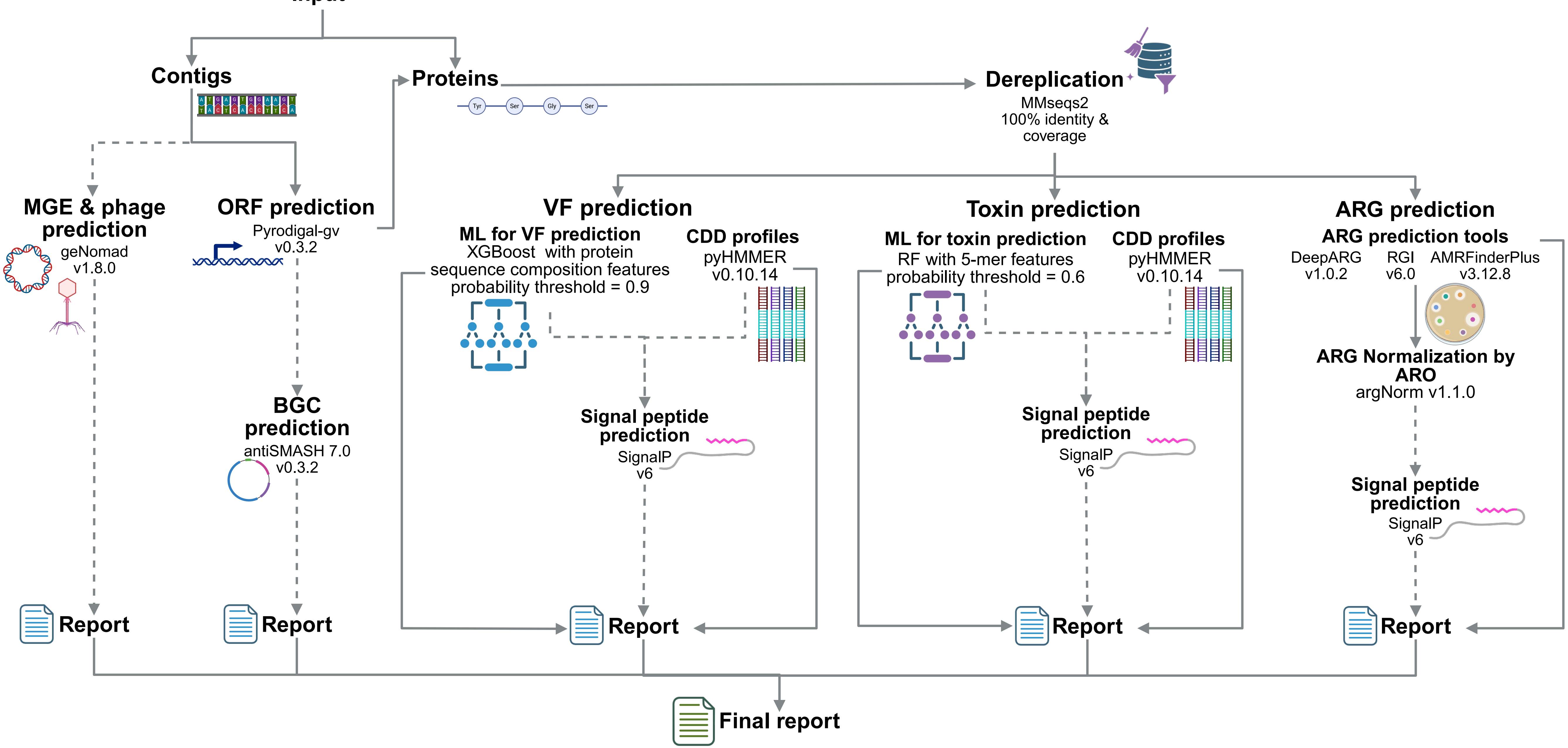

Click here to access/download;Figure;Figure2.pdf

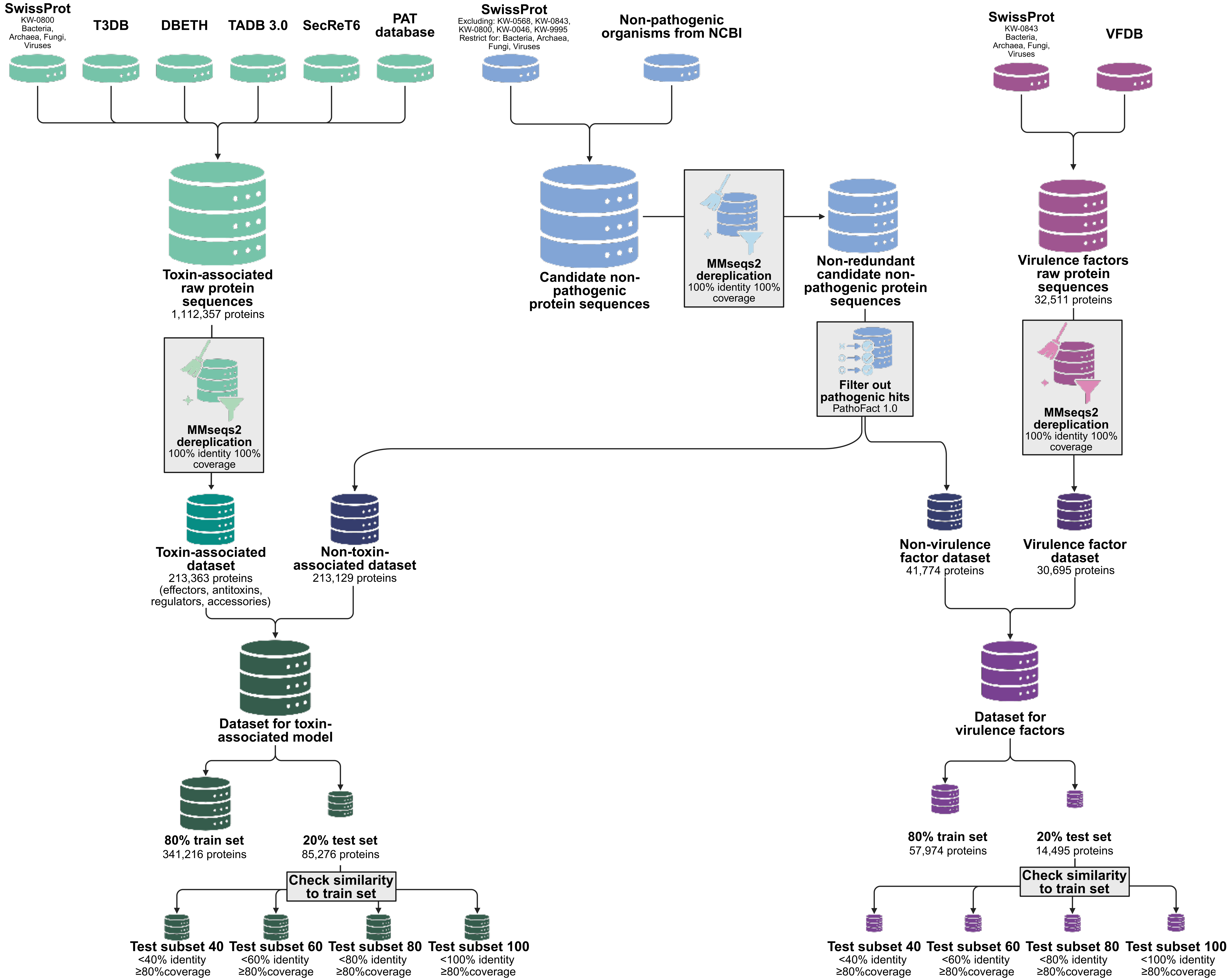

Figure 3

Click here to access/download;Figure;Figure3.jpg

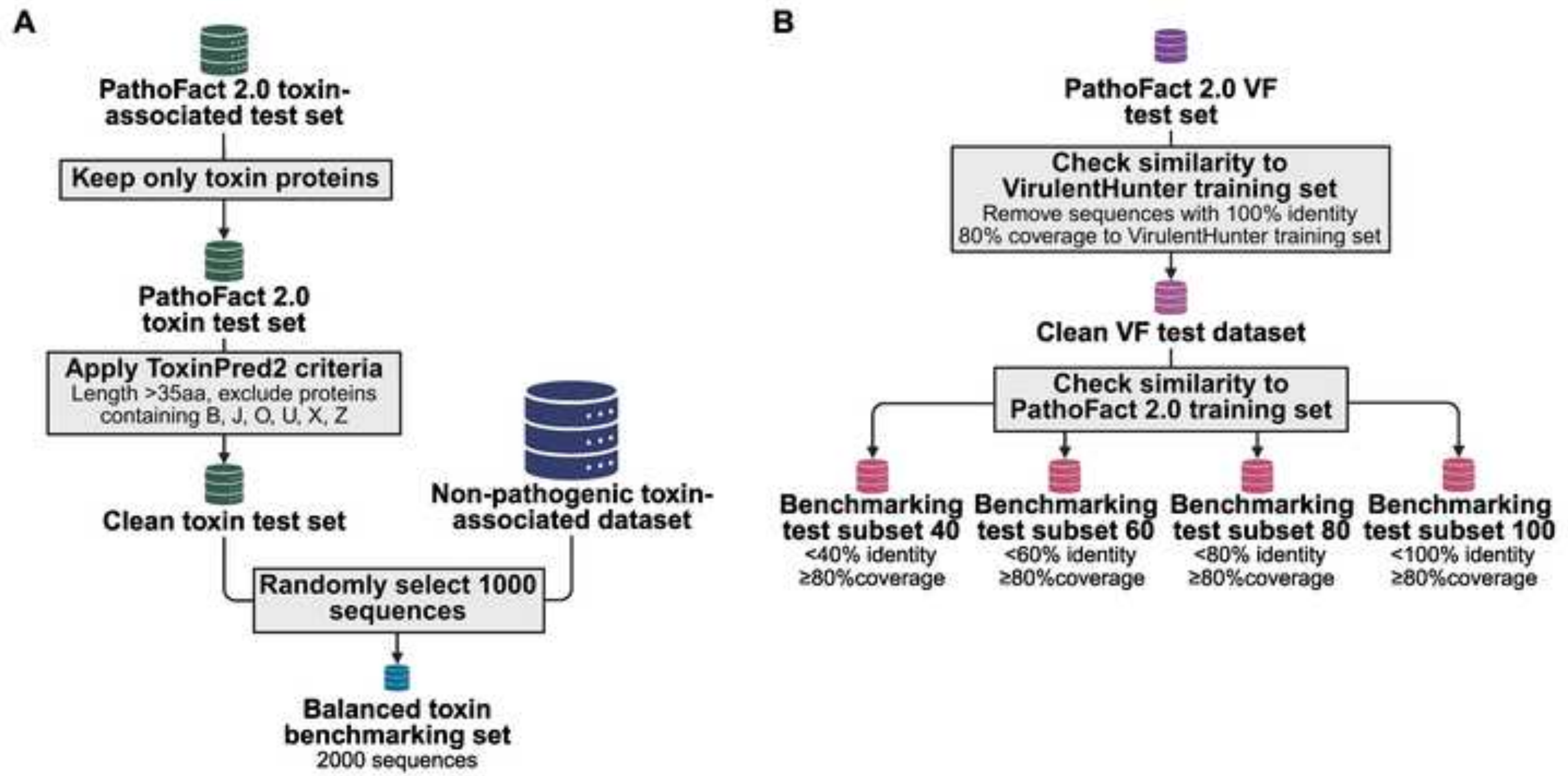

Figure 4

Click here to access/download;Figure;Figure4.pdf

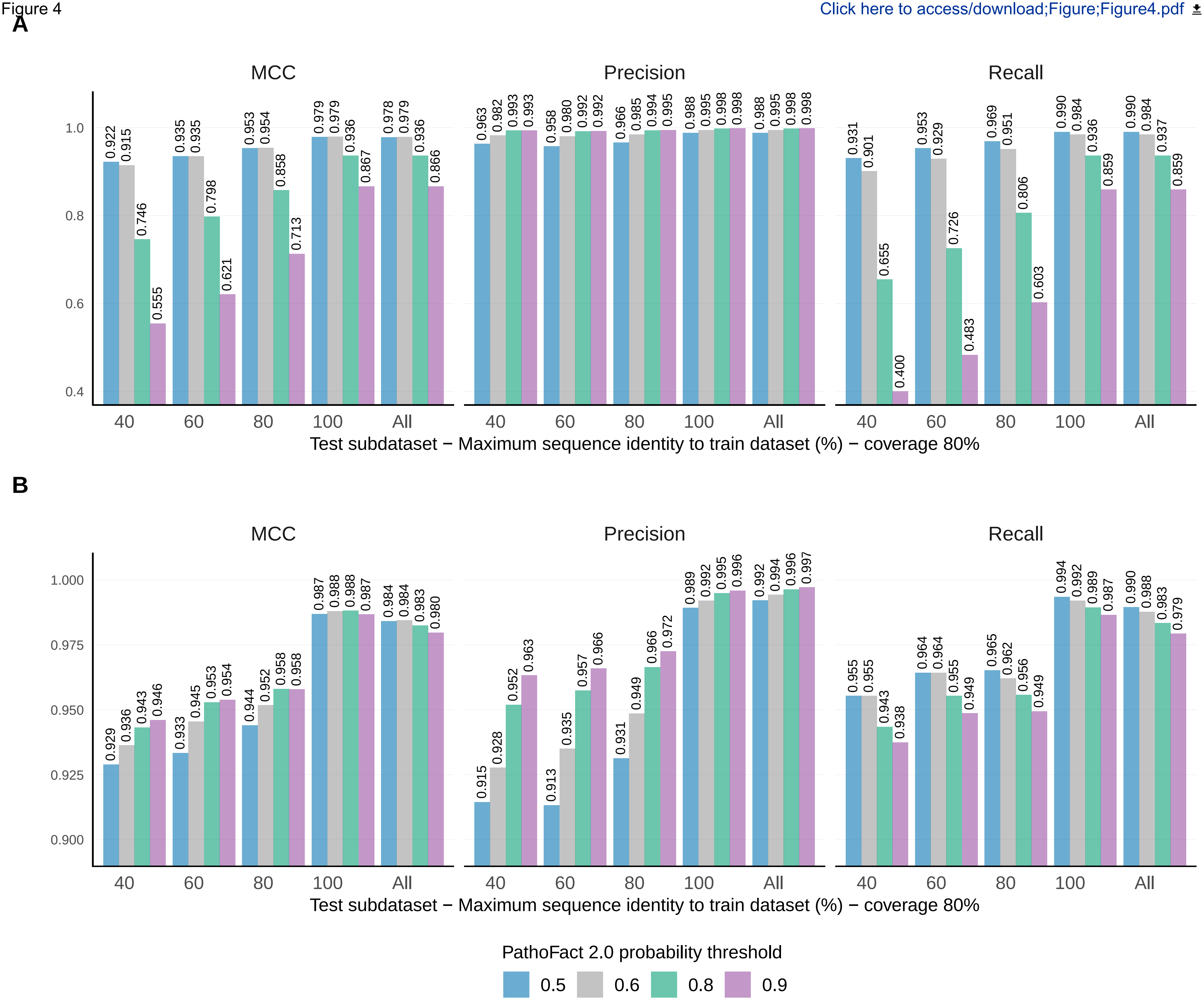

Figure 5

Click here to access/download;Figure;Figure5.pdf

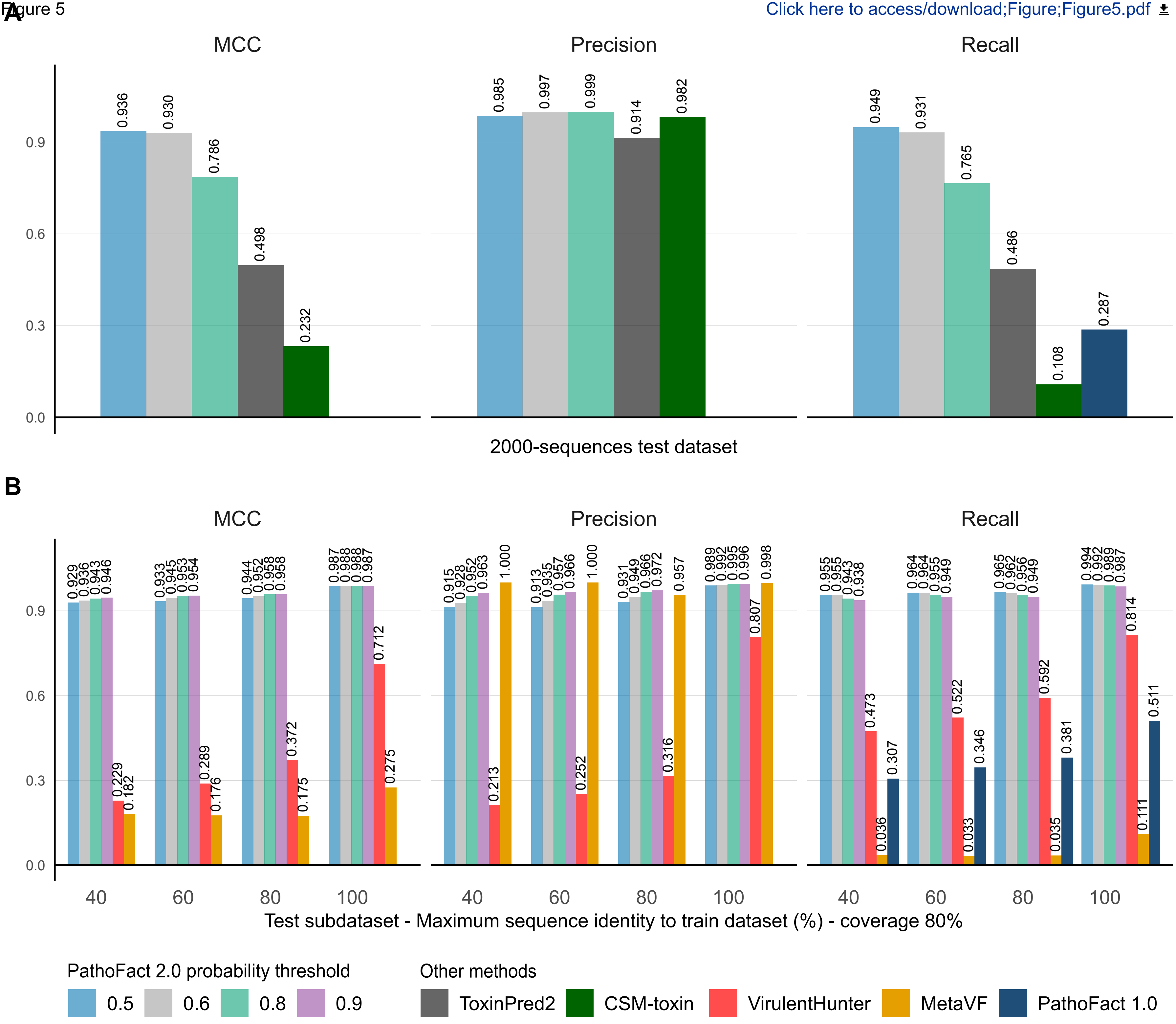

Figure 6

Click here to access/download;Figure;Figure6.pdf

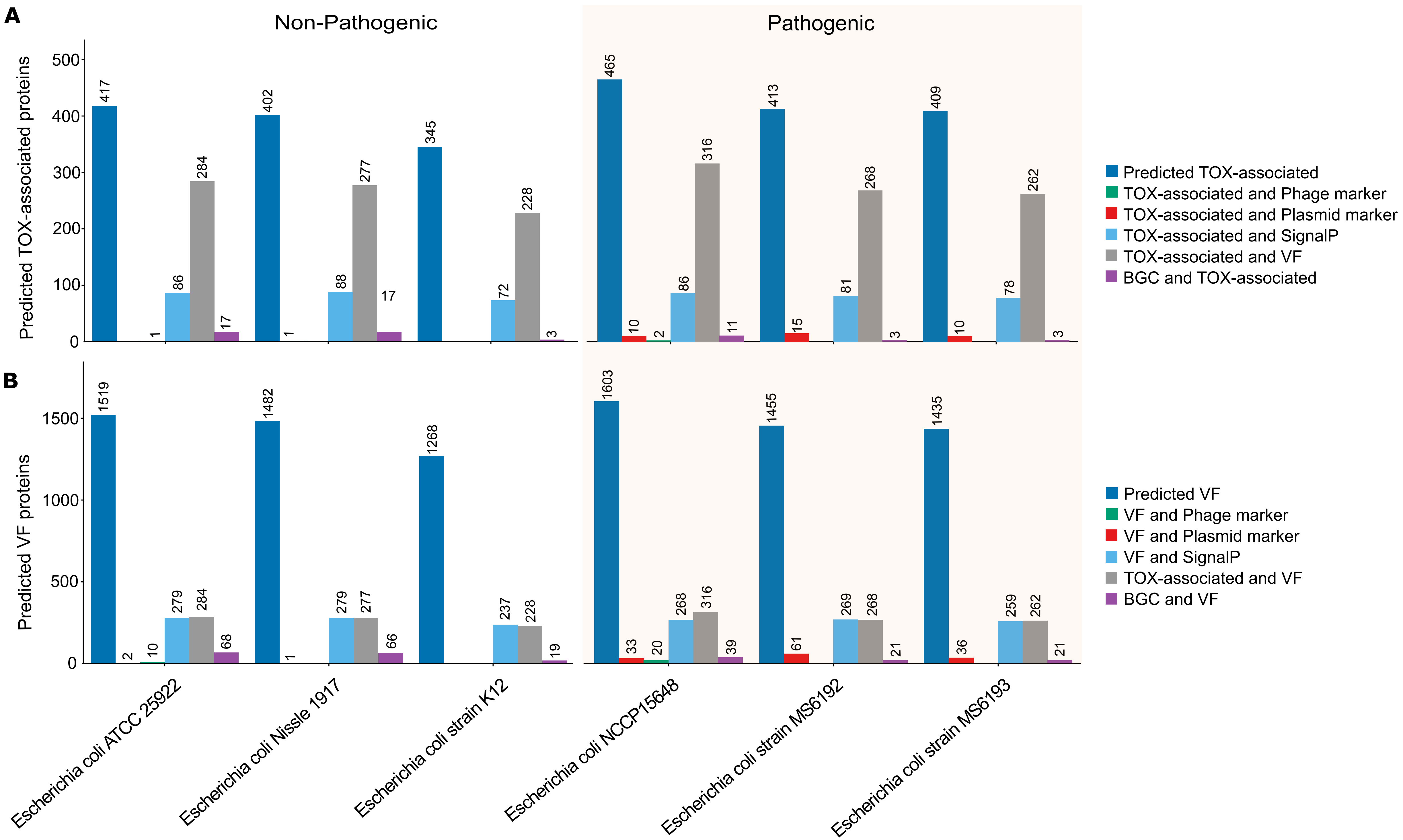

Click here to access/download;Figure;Figure7.pdf

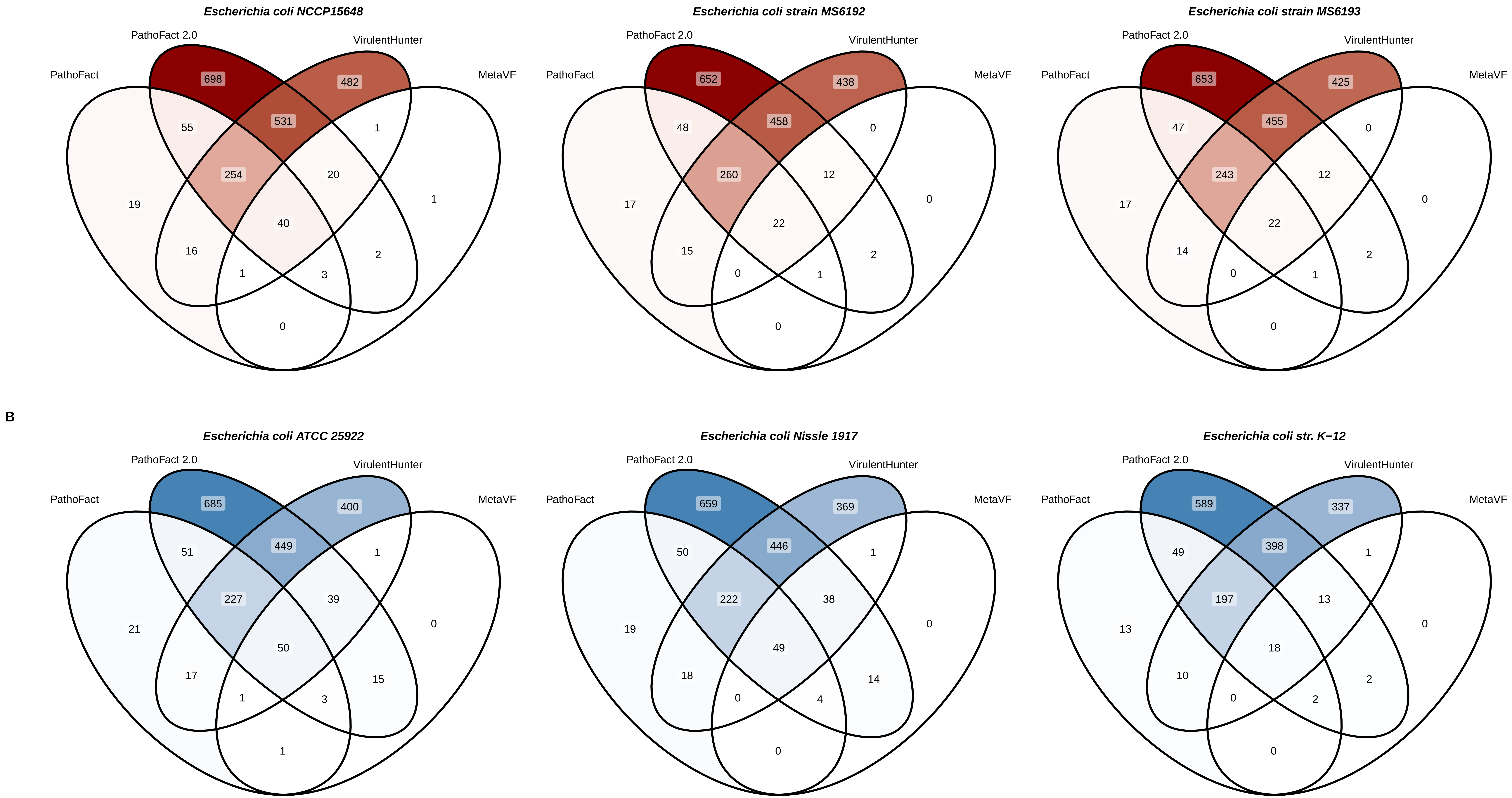

Figure 8

Click here to access/download;Figure;Figure8.pdf

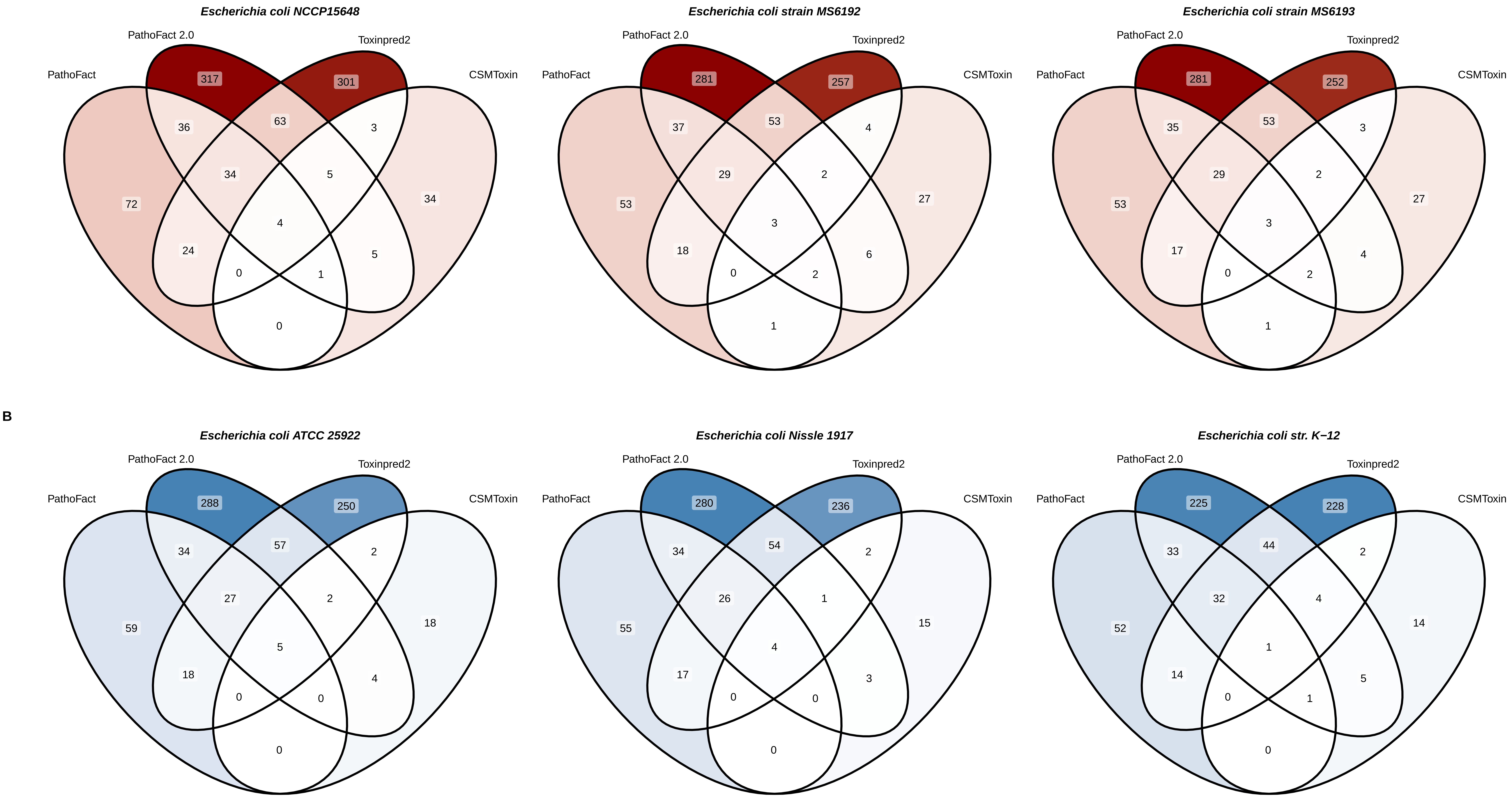

Supplementary Material - Figure S1

Click here to access/download
Supplementary Material
FigureS1_supplementary_material.pdf

Click here to access/download
Supplementary Material
FigureS2_supplementary_material.pdf

Click here to access/download
Supplementary Material
FigureS3_supplementary_material.pdf

Click here to access/download
Supplementary Material
FigureS4_supplementary_material.pdf

Supplementary Material - Figure S5

Click here to access/download
Supplementary Material
FigureS5_supplementary_material.pdf

Click here to access/download
Supplementary Material
FigureS6_supplementary_material.pdf

Supplementary Material - Figure S7

Click here to access/download
Supplementary Material
FigureS7_supplementary_material.pdf

Supplementary Material - Figure S8

Click here to access/download

Supplementary Material

FigureS8_supplementary_material.pdf

Supplementary Material - Figure S9

Click here to access/download
Supplementary Material
FigureS9_supplementary_material.pdf

Click here to access/download
Supplementary Material
TableS1_supplementary_material.pdf

Click here to access/download
Supplementary Material
TableS2_supplementary_material.pdf

Click here to access/download
Supplementary Material
TableS3_supplementary_material.pdf

Click here to access/download
Supplementary Material
TableS4_supplementary_material.pdf

Click here to access/download
Supplementary Material
TableS5_supplementary_material.pdf

Click here to access/download
Supplementary Material
TableS6_supplementary_material.pdf

Personal Cover Click here to access/download;Personal Cover;CoverLetter_PathoFact_April2026.pdf

Luxembourg Centre for Systems Biomedicine

Dr. Weixue Mu
Editor
GigaScience
BGI Shenzhen
China

UNIVERSITÉ DU LUXEMBOURG

Paul Wilmes
Professor of Systems Ecology
Luxembourg Centre for Systems Biomedicine
University of Luxembourg
7, avenue des Hauts-Fourneaux
L-4362 Esch-sur-Alzette
Luxembourg

16 April 2026

Dear Dr. Mu,

We thank you and the reviewers for the careful and constructive evaluation of our manuscript entitled "**PathoFact 2.0: An Integrative Pipeline for the Prediction of Antimicrobial Resistance Genes, Virulence Factors, Toxins and Toxin-associated Proteins, and Biosynthetic Gene Clusters in Metagenomes**". We are grateful for the detailed feedback, which has significantly improved the clarity, rigor, and usability of both the manuscript and the software.

We are pleased to submit a revised version of the manuscript that addresses all reviewers' and editor's comments. In this revision, we clarified several aspects of the benchmarking and evaluation framework, refined figure presentation, and improved the description of the pipeline's capabilities and limitations.

In response to reviewer comments, we updated Figures 4 and 5 to clarify axis ranges and improve visualization, incorporated additional benchmarking analyses including a comparison with PathoFact 1.0 where technically feasible, and added supplementary AUROC analyses (Figures S3 and S4) to complement the threshold-dependent evaluation metrics. We also revised the interpretation of prediction results to clearly distinguish between prediction counts and performance evaluation and added new supplementary figures (S6 and S9) showing overlaps between predictions generated by different tools.

To further contextualize the work within recent developments in reproducible bioinformatics workflows and microbial risk assessment, we incorporated relevant literature suggested by the editor and expanded the introduction accordingly.

We believe these revisions fully address the reviewers' concerns and strengthen the manuscript. We confirm that the work is original, not under consideration elsewhere, and that all authors have approved the revised submission.

Thank you for your consideration.

Yours sincerely,

Paul Wilmes, corresponding author
Email: paul.wilmes@uni.lu

Reviewer reports:

We would like to thank reviewers for their thorough review of the manuscript. We appreciate their comments and believe that these contributions have significantly improved the paper. Below are our responses to each comment, along with the corresponding manuscript text for readability.

Reviewer reports:
Reviewer #2: Overall, the authors have addressed partially my concerns, which is very satisfactory. Some comments have been left unresolved, but that is quite common when communicating by emails/comments. In particular, the article still struggle with comparing to other methods (or previous PathoFacts). It does make sense, in particular with other methods, as for example PathoFact predicts toxin-associated (while to other methods might just predict toxins). I believe my comments below give a clear path of how to have this article ready for publication.

Comments 6-9: Issues solved.

R/ We thank the reviewer for their acknowledgement of our satisfactory earlier revisions.

I. Comment 10: The issue in Figure 4 persists. The y-axis range are not visualized, and they differ from graph to graph. One would expect that in Precision and Recall, the y-axis would go from 0-1; in MCC, from -1 to 1, but it is also very common just to use 0-1. I must insist that the current layout is misleading.

R/ We thank the reviewer for the comment. In accordance with the comment, we have added the Y-axis range to figures 4 and 5. Although the Y-axis ranges are the same across figures, they have been limited to reduce visual saturation.

Comments 11-12: Addressed. If the authors have tried and they say this is the best, I believe them.

R/ We thank the reviewer for their acknowledgement of our satisfactory earlier revisions.

II. Comment 13: I was actually not asking for a detailed description of the parameters. I just believe using "maximum depth" is better than "max_depth". But that is also an aesthetic decision, so if the editor is fine with it, it is also fine with me.

R/ We thank the reviewer for the comment. We have added a detailed description of the parameters, lines 224-226 and 244-245.

III. Comment 14: Unfortunately, I must strongly disagree with the authors. Yes, MCC is a very good approach to evaluate models (particularly on imbalanced datasets); but if the authors defend that threshold fine-tuning is crucial for the model, AUROC

measures are necessary. And the conception of AUROC being not a correct measure for imbalanced datasets have been proven wrong in different articles, among:

* McDermott MB, Zhang H, Hansen LH, Angelotti G, Gallifant J (2024) A Closer Look at AUROC and AUPRC under Class Imbalance. In: Advances in Neural Information Processing Systems. pp 44102-44163 https://eur01.safelinks.protection.outlook.com/?url=https%3A%2F%2Fproceedings.neurips.cc%2Fpaper_files%2Fpaper%2F2024%2Fhash%2F4df3510ad02a86d69dc32388d91606f8-Abstract-Conference.html&data=05%7C02%7Cpaul.wilmes%40uni.lu%7C7db2d2e5b64f482096f308de9486c817%7C445a9c950f9d49539db1bc4a45dd1220%7C0%7C0%7C639111503821921584%7CUnknown%7CTWFpbGZsb3d8eyJFbXB0eU1hcGkiOnRydWUsIlYiOiIwLjAuMDAwMCIsIlAiOiJXaW4zMiIsIkFOIjoiTWFpbCIsIldUIjoyfQ%3D%3D%7C0%7C%7C%7C&sdata=M7lZ2jn9vdIimLRie4cLhMIX4LYxiXJoNAiy795f698%3D&reserved=0

* Richardson E, Trevizani R, Greenbaum JA, Carter H, Nielsen M, Peters B (2024) The receiver operating characteristic curve accurately assesses imbalanced datasets. Patterns 5(6):100994. https://eur01.safelinks.protection.outlook.com/?url=https%3A%2F%2Fdoi.org%2F10.1016%2Fj.patter.2024.100994&data=05%7C02%7Cpaul.wilmes%40uni.lu%7C7db2d2e5b64f482096f308de9486c817%7C445a9c950f9d49539db1bc4a45dd1220%7C0%7C0%7C639111503821956310%7CUnknown%7CTWFpbGZsb3d8eyJFbXB0eU1hcGkiOnRydWUsIlYiOiIwLjAuMDAwMCIsIlAiOiJXaW4zMiIsIkFOIjoiTWFpbCIsIldUIjoyfQ%3D%3D%7C0%7C%7C%7C&sdata=QkiL2lT8RFC05OR8FDLmfa28N9gFxFNtbXnXE6DGjV0%3D&reserved=0

R/ We thank the reviewer for this important clarification and for highlighting recent literature on the use of AUROC under class imbalance. Our intention was not to suggest that AUROC is inappropriate for imbalanced datasets, but rather to emphasise its limitations for threshold-dependent decision-making.

Here is an example of how the AUROC figures compare PathoFact 2.0 and VirulentHunter:

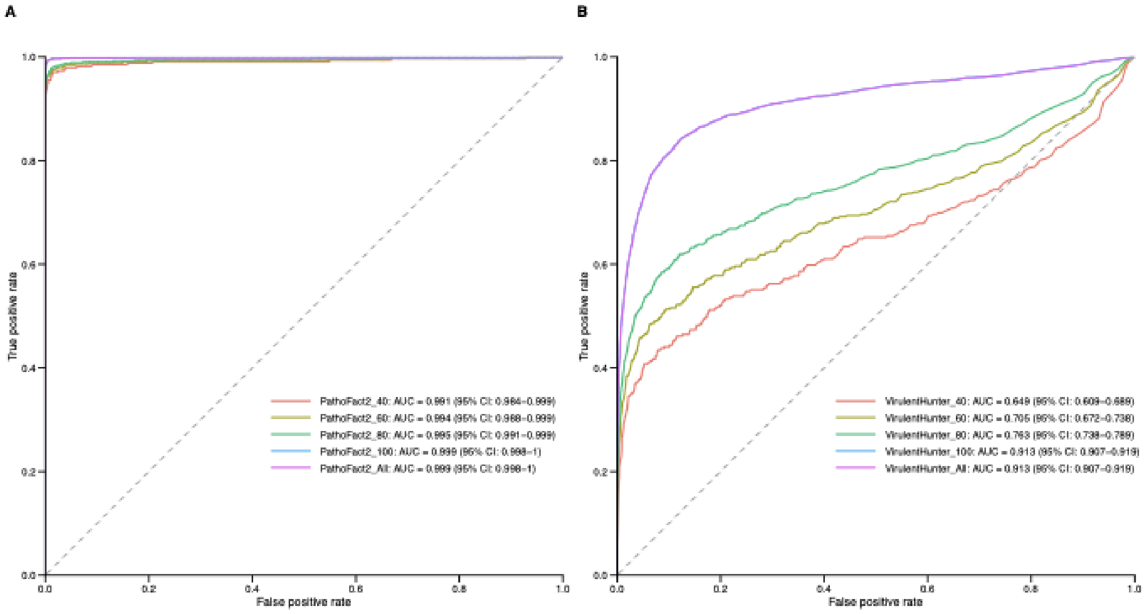

Here is an example of the AUROC figure comparing PathoFact 2.0 and CSM-Toxin:

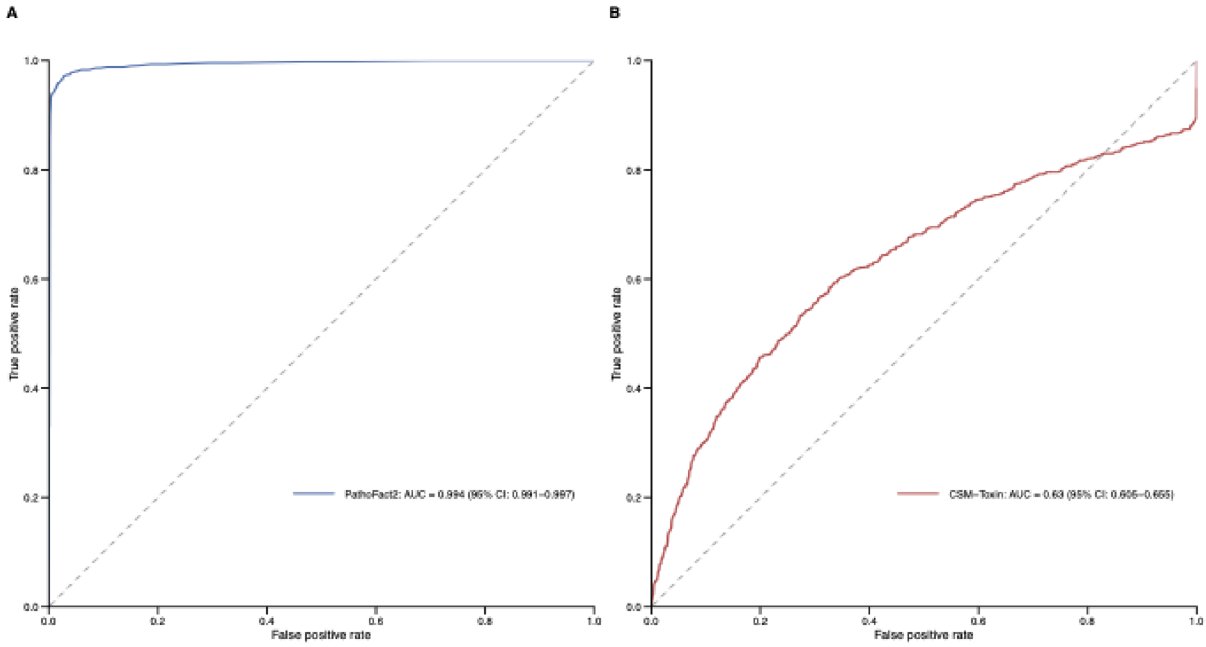

Such AUROC figures are not possible with Toxinpred2, since its predictions are based on a combination of factors rather than on machine-learning probabilities alone. Nor are they possible with PathoFact 1.0, because it also uses a combination of machine learning and HMM classification. MetaVF is an alignment-based algorithm, and it does not generate probability scores; therefore, AUROC curves are not possible.

AUROC evaluates a classifier's ranking performance across all possible thresholds, whereas our study focuses on selecting an operational probability threshold for practical application. In this context, threshold-dependent metrics are required. We chose MCC for this purpose because MCC incorporates all elements of the confusion matrix (TP, TN, FP, FN), providing a balanced evaluation even under strong class imbalance. Nonetheless, the AUROC figures have been included in the manuscript as supplementary figures S3 and S4 (lines 351-352).

IV. Comment 15: The answer from the authors do not have anything to do with my comment. So I will write it more clearly: if you release a new version of PathoFact (PathoFact2), you should benchmark it against your older version (PathoFact1.0). And if it not only includes an updated database, you should test PathoFact2 against PathoFact 1.0 with the new database. Right now there is only a comparison on how many more VF and toxins PathoFact2 detects, which is not a Performance evaluation (see Comment 18). A proper Performance evaluation (accuracy measures, such as MCC, Precision, Recall, AUROC) is necessary. It has been done against other ML methods, but not against the older method.

R/ We thank the reviewer for clarifying this point. We agree that benchmarking PathoFact 2.0 against the previous version (PathoFact 1.0) is important whenever possible.

PathoFact 1.0 accepts contigs as input, whereas PathoFact 2.0 accepts both contigs and protein sequences, representing one of the improvements in the updated pipeline. To enable

a comparison, we use the corresponding gene nucleotide sequences as input for PathoFact 1.0. However, a full metric-based comparison on this dataset would be inherently biased. The negative dataset used in the benchmark was originally constructed and filtered using PathoFact 1.0, which suppresses false positives for PathoFact 1.0 by design. As a result, PathoFact 1.0 achieves artificially perfect precision on this dataset, rendering precision-based metrics such as MCC unreliable, as they rely on a biased confusion matrix.

Furthermore, AUROC cannot be calculated for PathoFact 1.0 predictions. The virulence factor module combines machine-learning probability scores with HMM domain detection, whereas the toxin module relies solely on HMM presence/absence rather than a continuous prediction score, thereby preventing meaningful ROC analysis.

To avoid biased interpretation, we therefore reported recall only for PathoFact 1.0 on this benchmark dataset. Under these conditions, PathoFact 1.0 achieved a recall of 0.287 for the toxin module, compared to 0.931 for PathoFact 2.0. For the virulence factor module, PathoFact 1.0 achieved recalls of 0.307, 0.346, 0.381, and 0.511 for test subsets 40, 60, 80, and 100, respectively, which are substantially lower than those obtained with PathoFact 2.0.

We have added this benchmarking analysis to the revised manuscript and clarified that the comparison with PathoFact 1.0 is limited to recall only because the benchmark dataset's design does not allow an unbiased evaluation of all performance metrics. Lines 353-357:

> “Because the negative dataset was built and then filtered using PathoFact 1.0, the precision for PathoFact 1.0 is 1, which affects the MCC calculation. To avoid biased comparisons, we report recall only for PathoFact 1.0. Figure 5 shows that PathoFact 2.0 VF and toxin-associated modules achieved higher recall across test subsets than PathoFact 1.0, and better recall and MCC than metaVF.”

Comment 16-17: Issues addressed.

R/ We thank the reviewer for their acknowledgement of our satisfactory earlier revisions.

V. Comment 18: Yes, I agree a full biological assessment of VF and toxins in different E. coli would be an independent study. I am more than familiar with the complexity of the strain. However, the authors use Figure 7 and 8 as "Performance Comparison" of PathoFact2.0 against other methods, and that is not what it is. What we have is just the counts of how many times each model detected a VF or a toxin. Besides clearly promoting recall over precision, there is no indication on those predictions being correct. So, statements such as "PathoFact 2.0 consistently detected a greater number of VFs than both PathoFact 1.0 and MetaVF" are incorrect, as what those graphs show is that PathoFact2.0 consistently PREDICTS a greater number of VFs. I am not saying that is a bad thing that it predicts more, but if the authors are going to be using those results the claims have to change and be very explicit that they are not using quantity as a way of Performance comparison or evaluation.

> These types of claims also get a very vague appearance as "Our improved pipeline, PathoFact 2.0, offers significant improvements over PathoFact (its predecessor), ToxinPred2, CSM-toxin, VirulentHunter, and metaVF" in the Conclusions. The authors should be much more precise in the conclusions about what are the significant improvements. Moreover, if the authors insist in using those results, they would be much more informative showing the overlap of VF and toxins predicted by each method evaluated.

R/ We thank the reviewer for the comment. We have used DNA (gene) sequences as input for PathoFact 1.0 and MetaVF, and have now included them in Figure 5.

In addition, we have clearly shown that MetaVF does not predict VF in known pathogenic strains, highlighting a major limitation of this method, with very low recall and MCC compared with PathoFact 2.0.

PathoFact 2.0 and PathoFact 1.0 are pipelines, and we have demonstrated the advantages of PathoFact 2.0 over PathoFact 1.0 in speed, user-friendliness, updates, new tools (e.g., BGC), and the ability to use protein as input. Now that we have included PathoFact 1.0 in Figure 5, the improvements with PathoFact 2.0 are clearer.

To address the reviewer's comment, we have modified Figures 5, 7 and 8 and added two new supplementary figures, S6 and S9, which include Venn diagrams showing the overlap of VF and toxins predicted by each evaluated method.

In addition, we have removed the subtitle benchmarking to avoid confusion (between lines 405 and 406 of the revised manuscript).

We have modified the sentences: “PathoFact 2.0 consistently detected a greater number of VFs than both PathoFact 1.0 and MetaVF" to “PathoFact 2.0 consistently predicted a greater number of VFs than both PathoFact 1.0 and MetaVF”, in lines 410-411.

As written in the manuscript after the sentences "Our improved pipeline, PathoFact 2.0, offers significant improvements over PathoFact (its predecessor), ToxinPred2, CSM-toxin, VirulentHunter, and metaVF", we clearly expose the benefits of PathoFact 2.0 over PathoFact 1.0 (lines 488-493):

> “SignalP has been upgraded and made optional to further optimise performance, providing users with flexibility based on their requirements. Additionally, antiSMASH 7.0 facilitates the prediction of BGCs, recognising emerging evidence that some BGC-encoded factors might increase virulence. Furthermore, we have integrated geNomad, a cutting-edge tool for identifying MGEs, including plasmids and phages linked to ARGs, VFs, toxins and toxin-associated proteins across various bacterial species.”

Comment 19: More or less addressed (also by previous comments).
Comment 20-21: Addressed
Comment 22-26: Agree with authors.

R/ We thank the reviewer for their acknowledgement of our satisfactory earlier revisions.

Editor Comments:
Please be informed that Reviewer 3 was unable to accept the invitation to provide comments on your revised manuscript. Instead, Reviewer 2 has assisted in reviewing the changes made in response to Reviewer 3's comments.

VI. In addition, GigaScience has also published a number of relevant papers on metadata standardization, FAIR data practices, and reproducible workflows in biotechnology that may help contextualize your work. We have listed several recent examples below for your reference.

Citing some of these articles—where appropriate in the Background or Discussion—would help frame your contribution within ongoing community efforts and strengthen the presentation of related tools and frameworks.
1. Kasmanas J C, Magnúsdóttir S, Zhang J, et al. Integrating comparative genomics and risk classification by assessing virulence, antimicrobial resistance, and plasmid spread in microbial communities with gSpreadComp[J]. GigaScience, 2025, 14: giaf072. https://eur01.safelinks.protection.outlook.com/?url=https%3A%2F%2Fdoi.org%2F10.1093%2Fgigascience%2Fgiaf072&data=05%7C02%7Cpaul.wilmes%40uni.lu%7C7db2d2e5b64f482096f308de9486c817%7C445a9c950f9d49539db1bc4a45dd1220%7C0%7C0%7C639111503821991703%7CUnknown%7CTWFpbGZsb3d8eyJFbXB0eU1hcGkiOnRydWUsIlYiOiIwLjAuMDAwMCIsIlAiOiJXaW4zMiIsIkFOIjoiTWFpbCIsIldUIjoyfQ%3D%3D%7C0%7C%7C%7C&sdata=Iu3VScoPqNhU85T%2Bral3skRDN0Th9zmsADAob6BnsqA%3D&reserved=0

2. Nachtigall P G, Durham A M, Rokyta D R, et al. ToxCodAn-Genome: an automated pipeline for toxin-gene annotation in genome assembly of venomous lineages[J]. Gigascience, 2024, 13: giad116. https://eur01.safelinks.protection.outlook.com/?url=https%3A%2F%2Fdoi.org%2F10.1093%2Fgigascience%2Fgiad116&data=05%7C02%7Cpaul.wilmes%40uni.lu%7C7db2d2e5b64f482096f308de9486c817%7C445a9c950f9d49539db1bc4a45dd1220%7C0%7C0%7C639111503822028671%7CUnknown%7CTWFpbGZsb3d8eyJFbXB0eU1hcGkiOnRydWUsIlYiOiIwLjAuMDAwMCIsIlAiOiJXaW4zMiIsIkFOIjoiTWFpbCIsIldUIjoyfQ%3D%3D%7C0%7C%7C%7C&sdata=oprnTmJkrIEzP3z%2Bgm7rmxt3v7iod9BIGhwqmO9G0SA%3D&reserved=0

3. Roach M J, Beecroft S J, Mihindukulasuriya K A, et al. Hecatomb: an integrated software platform for viral metagenomics[J]. GigaScience, 2024, 13: giae020. https://eur01.safelinks.protection.outlook.com/?url=https%3A%2F%2Fdoi.org%2F10.1093%2Fgigascience%2Fgiae020&data=05%7C02%7Cpaul.wilmes%40uni.lu%7C7db2d2e5b64f482096f308de9486c817%7C445a9c950f9d49539db1bc4a45dd1220%7C0%7C0%7C639111503822063704%7CUnknown%7CTWFpbGZsb3d8eyJFbXB0eU1hcGkiOnRydWUsIlYiOiIwLjAuMDAwMCIsIlAiOiJXaW4zMiIsIkFOIjoiTWFpbCIsIldUIjoyfQ%3D%3D%7C0%7C%7C%7C&sdata=At4N2T7tySZC1zzhpHAOn%2BMQLkEbZF%2Ftt1qDODGRpNU%3D&reserved=0

--

Please also take a moment to check our website at https://eur01.safelinks.protection.outlook.com/?url=https%3A%2F%2Fwww.editorialmanager.com%2Fgiga%2Fl.asp%3Fi%3D243133%26l%3D0MN2ZJHN&data=05%7C02%7Cpaul.wilmes%40uni.lu%7C7db2d2e5b64f482096f308de9486c817%7C445a9c950f9d49539db1bc4a45dd1220%7C0%7C0%7C639111503822100221%7CUnknown%7CTWFpbGZsb3d8eyJFbXB0eU1hcGkiOnRydWUsIlYiOiIwLjAuMDAwMCIsIlAiOiJXaW4zMiIsIkFOIjoiTWFpbCIsIldUIjoyfQ%3D%3D%7C0%7C%7C%7C&sdata=LNZe%2FQUjXNSrpawcB%2B91rwZt7mpXYoCMx7prfYfQgSI%3D&reserved=0

for any additional comments that were saved as attachments.

R/ We would like to thank the editor for the helpful suggestion. The relevant paper has now been included in the introduction (lines 109 and 117):

> "gSpreadComp (https://doi.org/10.1093/gigascience/giaf072) recently proposed a workflow that integrates comparative genomics, plasmid-mediated transfer assessment, and resistance-virulence risk-ranking to facilitate hypothesis generation for targeted experimental validation by identifying concerning resistant hotspots in complex microbial datasets. However, while gSpreadComp is valuable for comparative, community-level prioritisation, ARGs are predicted using only one tool, DeepARG, and VF are annotated using databases and alignment-based approaches rather than predictive models and machine learning frameworks. In addition, it lacks a signal peptide or a BGC annotation framework needed for comprehensive microbiome-based pathogenicity profiling, and the pipeline accepts only DNA sequences as input"

We also thank you for suggesting additional references. However, papers 2 and 3 were not incorporated because they do not align with this study's scope. Paper 2 examines venomous snakes, while our research concentrates on predicting toxins and related proteins in prokaryotes, viruses, and microeukaryotes. Paper 3 deals with virome characterisation at both the read and contig levels, which is also beyond the scope of this study.
