# Supplementary material for: PathoFact 2.0: an integrative pipeline for the prediction of antimicrobial resistance genes, virulence factors, toxins and toxin-associated proteins, and biosynthetic gene clusters in metagenomes
Source: Gigascience. 2026 May 22;15:giag062. doi: 10.1093/gigascience/giag062 (PMC13224393; doi:10.1093/gigascience/giag062)
Supplement: giag062_Authors_Response_To_Reviewer_Comments_original_submission [file giag062_authors_response_to_reviewer_comments_original_submission.pdf]

Author's Response To Reviewer Comments

Reviewer reports:

We would like to thank all the reviewers for their thorough review of the manuscript. We appreciate their comments and believe that these contributions have significantly improved the paper. Below are our responses to each comment, along with the corresponding manuscript text for readability. In addition to the clean, updated manuscript submitted with our reviewer response, we have included an updated version that highlights our edits and additions to make the changes easy to identify.

Editor comments:

1. In addition, please register any new software application in the bio.tools and SciCrunch.org databases to receive RRID (Research Resource Identification Initiative ID) and biotoolsID identifiers, and include these in your manuscript. Computation workflows should be registered in workflowhub.eu and the DOIs cited in the relevant places in the manuscript. These will facilitate tracking, reproducibility and re-use of your tool.

R/ We thank you for this suggestion. We have now included the RRID, which is SCR_027650. We have also included the bio.tools ID: pathofact2 and the DOI for workflowhub.eu: 10.48546/workflowhub.workflow.2087.1

Reviewer #1:
The pipeline should be very useful on shotgun metagenomics data analysis.
Aside the ARGs and VFs, the features on signal peptides, toxin predictions, and BGCs in particular for specialised metabolites predictions, are welcome for detailed analysis and understanding of various transmission mechanisms.
I find the approaches very appealing and I think the pipeline could be welcomed by the community.

R/ We acknowledge and thank the reviewer for their recognition of our work and their overall positive feedback.

I only have some minor observations:
2. The Methods section should be placed next to the described methods. As it is, at the end of the manuscript, under Methods chapter you can only find Datasets, so a proper formatting of the Methods is required

R/ We thank the reviewer for the comment. We followed the journal's Technical Application Guidance: "For Technical Notes, this section is specific for including any additional methods used in the manuscript that are not part of the new work being described in the manuscript."
Nonetheless and to account for the reviewer's comment, we have expanded the Methods section accordingly (lines 494-558):

"Databases used for the PathoFact 2.0 Dataset Construction
SwissProt [31] is the expertly curated part of UniProtKB [66]. It offers high-quality protein sequences with detailed functiona annotations, including keywords for pathogenesis, virulence, toxins, and antibiotic resistance.
VFDB [43], the Virulence Factor Database, is a comprehensive reference for curating information on virulence factors of bacterial pathogens.
T3DB [12], the toxin and toxin-target database, is a resource cataloguing thousands of toxins and their protein targets, with detailed mechanisms, structures, and toxicity data, including bacterial protein toxins.
DBETH [13], the database for bacterial exotoxins, is a specialised database of bacterial exotoxins pathogenic to humans, classified into 24 mechanistic and activity types from 26 bacterial genera.
TADB [36], the toxin-antitoxin database, is a repository of bacterial toxin-antitoxin loci across types I-VIII, including experimentally validated pairs, predicted loci, and associations with mobile genetic elements.
SecReT6 [37] is a database containing known and predicted type VI secretion systems, including effectors, immunity proteins regulators, and accessory proteins from bacterial genomes.
PAT [38] is the prokaryotic antimicrobial toxin database and contains a collection of antimicrobial toxins, including bacteriocir and effectors from secretion systems.

Clustering Parameters
MMseqs2 [32] is an ultra-fast open-source software suite for sensitive protein and nucleotide sequence searching and clustering, up to 10,000 times faster than BLAST while retaining comparable sensitivity. For dereplication, it clusters sequenc

at 100% identity and 100% coverage using the parameters -c 1.0 and --min-seq-id 1.0. It removes exact duplicates and retains representative sequences to generate non-redundant datasets. To create the test subsets, a coverage of 80% (-c 0.8 and identities ranging from 40 - 100% (--min-seq-id 0.4, --min-seq-id 0.6, --min-seq-id 0.8, --min-seq-id 1.0) were used, ar then sequences with similarity higher than min-seq-id were removed for each test subset. In all cases, the cluster mode and coverage mode used were 0 (--cov-mode 0 --cluster-mode 0). When --cov-mode 0 is specified in combination with -c values ranging from 0.0 to 1.0, sequences are assigned to the same cluster only if the alignment spans at least a fraction c of the length of the longer sequence. According to the developers of MMSeq2, this coverage criterion is particularly suitable for clustering full-length protein sequences.
Protein Composition Features
Protein sequence composition features were extracted to represent each protein as fixed-length vectors derived from its primary amino acid sequence [35]. These included amino acid composition (AAC), dipeptide composition (DPC), composition (CTDC), transition (CTDT) and distribution (CTDD). AAC captures the relative frequency of each of the 20 amino acids in a sequence, whereas DPC captures the relative frequency of all adjacent amino-acid pairs (400 possible dipeptides). CTDC represents the percentage of amino acids belonging to each of three predefined groups (polar, neutral, hydrophobic) in the entire protein sequence. CTDT represents the percentage frequency with which a residue of one group is followed by a residu of a different group along the sequence. CTDD represents the distribution of each amino acid group, measuring the spatial position, where the first, 25%, 50%, 75% and 100% of the residues of a specific class are located.

Performance and Evaluation Metrics
To assess the models' performance, we used a confusion matrix comprising true positives (TP), true negatives (TN), false positives (FP), and false negatives (FN), computed on the test datasets described above. TP corresponds to truly positive instances correctly predicted as positive by the model, whereas TN corresponds to truly negative instances correctly predicted as negative. FP are truly negative instances incorrectly predicted as positive, and FN are truly positive instances incorrectly predicted as negative. From these values, we calculated the following metrics:

Accuracy = (TP + TN)/(TP+TN+FP+FN)

Precision = TP/(TP +FP)

Recall = TP/(TP+FN)

MCC =(TPTN-FPFN)/(TP+FP)(TP+FN)(TN+FP)(TN+FN)

HMM Profiles
Profile hidden Markov models are probabilistic models built from a multiple sequence alignment that encode, for each alignment position, the position-specific probabilities of residues and insertions/deletions, turning the alignment into a positio specific scoring system for detecting homologous sequences [67]. The FASTA files of conserved-domain multiple sequence alignments for each CDD [26] family (https://ftp.ncbi.nih.gov/pub/mmdb/cdd/fasta.tar.gz) were downloaded. Pyhmmer v0.10.14 [68] was used to obtain HMM profiles for each CDD family and to perform protein sequence searches against the CD HMM family profiles.

3. there is a 70 blank pages buffer between References and supplementary data

R/ This occurred during the submission of the manuscript to Gigascience. This is therefore outside of our control.

4. could you add some future prospects in the manuscript? How well is it going to be maintained - I noticed the update are quite old.

R/ We thank the reviewer for this helpful comment. Updates are prompted by user-reported issues, newly identified errors, a the availability of enhanced reference datasets or prediction models. A new version of PathoFact is scheduled for the future, which will include two new modules, one for biocide and environmental pollutants resistance and another for heavy metal resistance genes.

5. I would also add some more details to the limitations. For instance it is clear that the pipeline is installable on Linux platforms, but did you considered making it available also for Apple silicon series? More and more researchers use this technology, and it works as good as the Linux distributions. I also tried an install on a M series Apple silicon, but unfortunatel most of the tools in the pipeline lead to multiple errors related to python versions (most of which are old), missing old

dependencies versions, libraries, etc.

R/ We thank the reviewer for the comment. PathoFact 2.0 integrates several third-party tools whose compatibility with Apple Silicon (ARM architecture) is currently limited. Because these components are developed and maintained by external groups, addressing compatibility issues is outside the scope of PathoFact 2.0.
PathoFact 2.0 uses Mamba/Conda, with most packages from the Bioconda channel, which has been actively expanding suppo for ARM architecture. However, not all required dependencies are currently available or stable for Apple Silicon systems, leading to installation and runtime errors related to Python version mismatches and missing legacy libraries, as noted by the reviewer.
For these reasons, we have chosen to officially support Linux platforms, where all dependencies are stable, well-maintained, and widely adopted within the bioinformatics community. Once the required third-party tools and their dependencies are fully supported on ARM architectures through Bioconda or other channels, PathoFact 2.0 can be made compatible with Apple Silico systems. It is important to note that metagenomic data is usually large in size. To efficiently run bioinformatic metagenomic workflows and to store the data and results, they are typically kept on dedicated servers or high-performance computing (HP clusters. Therefore, PathoFact 2.0 has been designed to be run on HPCs.

Reviewer #2:
The authors present the pipeline PathoFact 2.0, which combines external modules and machine learning algorithms in order t find genes that provoke antimicrobial resistance, virulence and toxicity. They present their work, the improvement from the previous version, as a Technical Note.
For what the authors say, it is the only pipeline available with those characteristics, which makes it clearly a relevant softwar and article. However, I believe the article requires refinement, as well as new tests that support the authors claims.

R/ We acknowledge and thank the reviewer for their recognition of our work and their overall positive feedback. We further thank the reviewer for their constructive criticisms, which have allowed us to strengthen the work and the resulting manuscript.

Refinements on the article
6. In the abstract, ARG is used as an abbreviation for Antimicrobial Resistance, not Antimicrobial Resistance Genes.

R/ We thank the reviewer for the comment. This has now been corrected (lines 20-21):

“Antimicrobial resistance genes (ARG) and virulence factors (VFs) are central contributors to the global health crisis surrounding drug-resistant infections.”

7. Overall, the article should be more clear in what the pipeline is made for. It mentions fungi, viral, etc… sequences (which might be found on a metagenomic sample, of course), but to my understanding, all the tools and phenotypes searched for ar mostly characteristic of bacteria.

R/ We thank the reviewer for this comment.
PathoFact 2.0 is explicitly designed for the analysis of metagenomic datasets, as stated in the title. In such datasets, prokaryotes (bacteria and archaea) represent the dominant fraction of organisms, and accordingly, most prediction modules PathoFact 2.0 are optimised for bacterial proteins. The virulence factors, toxin-associated proteins, antimicrobial resistance genes, and secretion-related features targeted by the pipeline are therefore primarily bacterial in nature.

At the same time, metagenomic assemblies may contain sequences from viruses and eukaryotes (including fungi). For this reason, PathoFact 2.0 includes tools that can process non-bacterial sequences where appropriate. For example, protein prediction is performed using Prodigal-gv, which supports both prokaryotic and viral genomes; SignalP predicts signal peptide in proteins from archaea, bacteria, and eukaryotes; and antiSMASH includes dedicated modes for bacterial and fungal biosynthetic gene clusters. These components enable the pipeline to handle the mixed characteristics of metagenomic data.

We acknowledge that this duality, broad input acceptance versus primarily bacterial biological interpretation, was not sufficiently explicit in the current version of the manuscript.

We have added to the limitations of PathoFact 2.0 section the following paragraph (lines 471-473):

“PathoFact 2.0 is designed for metagenomic samples; most prediction modules and phenotypes are bacterial-centric. Virulenc

toxin-associated, and antimicrobial resistance predictions are particularly interpreted in the context of human pathogens"

8. While the introduction offers a good resume of the genes of interest, there are some descriptions that are not particularly accurate. "Human, animal, and environmental microbiomes harbour commensal and pathogenic microorganisms, contributin[illegible] to the emergence of infectious diseases" seems to say that commensal microorganisms contribute to the emergence of infectious diseases;

R/ We thank the reviewer for the comment; it has been revised. We have modified the paragraph (Lines 40-47):

"Microbiomes are highly complex and diverse ecological communities composed of bacteria, archaea, viruses, and microeukaryotes. These communities include both commensal microorganisms, which can contribute to host health, and pathogenic or opportunistic microorganisms that can cause disease under specific conditions. Microbial communities generall[illegible] exist in synergistic relationships with their hosts, playing critical roles in maintaining physiological homeostasis and regulatin[illegible] immune function. However, disruption of this balanced microbial ecosystem, known as microbial dysbiosis, can impair norma[illegible] body functions and has been associated with the development of various diseases, including cardiovascular diseases, cancers[illegible] and respiratory disorders [1]."

9. "ARGs are genetic elements that confer bacterial resistance to antibiotics, acquired via mutations or horizontal gene transfer." seems to say that antimicrobial resistance genes are acquired via mutation (they are not, there is a difference between resistance to antimicrobials provoked by mutations and by genes). I recommend a thorough rewriting of the 6 first paragraphs.

R/ We thank the reviewer for this relevant comment; It has been revised. The paragraph was modified (lines 50-51):

"ARGs are genetic elements that confer bacterial resistance to antibiotics. Many ARGs are found on mobile genetic elements (MGEs) and are therefore often horizontally transmitted [4]".

10. The graphs in Figures 1 and 2 have different Y axes, which are also not shown. This is, to say it lightly, very misleading.

R/ We thank the reviewer for the comment; Figure 1 is a schema, so it seems the reviewer is referring to Figure 2 (A and B) [illegible] the previous manuscript's version (now Figure 4). Figure 4 has been updated to clearly show the Y-axis labels as figure titles.

11. Table 1 would be much more clear as a Figure. Table 1 cited in line 211 does not exist.

R/ We thank the reviewer for the comment. After trying out different figure types, we concluded that a table better represent[illegible] our results than a figure. The reference to Table 1 has been removed (lines 179-180).

12. A short description of "dereplication" would help users lacking that knowledge.

R/ We thank the reviewer for the comment. In the first appearance of dereplication (both in figure 1 and pipeline structure section), and on several occasions in the manuscript, we included the following text: "proteins dereplicated by our tool to reta[illegible] only non-redundant sequences (based on 100% identity and coverage). " Lines 131-132.

13. The description of the parameters of the machine learning modules are a copy-paste of the variables used by scikit-learn (lines 248 and 268). The description of the machine learning models should be clearer and more detailed, as well as not force[illegible] the reader to go to the instructions of scikit-learn to check what is the meaning of those parameters.

R/ We thank the reviewer for the comment. As written in the manuscript (lines 170-172), we used XGBoost Python package (https://xgboost.readthedocs.io/en/stable/index.html) and the RandomForest (RF) Scikit-learn (version 1.5.2; [34]) to build our ML models. For the Random Forest model, the default hyperparameters provided by scikit-learn were used; for the XGBoost model, only the hyperparameters that differed from the defaults were explicitly specified. The parameter names wer[illegible] intentionally kept identical to those used in scikit-learn and the XGBoost Python package to ensure precision and reproducibility. These are standard hyperparameters of well-established models, and a detailed explanation of each is beyon[illegible] the scope of this manuscript. The full definitions and usage of these parameters are clearly documented in the referenced XGBoost Python package as well as the scikit-learn documentation [34](RandomForest: https://scikit-learn.org/stable/modules/generated/sklearn.ensemble.RandomForestClassifier.html#sklearn.ensemble.RandomForestClassifi[illegible] XGBoost: https://xgboost.readthedocs.io/en/stable/parameter.html), to which the reader is explicitly directed.

In the manuscript lines 217-218:
“The toxin-associated ML model is a RF with 5-mer features (default hyperparameter setting, i.e, n_estimators=100, max_depth=None, min_samples_split=2).”

and lines 237-238:
“The VF model uses XGBoost with protein sequence composition features (learning_rate': 0.1, 'n_estimators': 2000).”

14. The authors analyze the performance of the models depending on the "probability" (a term that could definitely use a better introduction) using barplots. A standard to analyze the probability of a model is a ROC curve.

R/ We thank the reviewer for the comment. However, we respectfully point out that ROC curves are not always the most appropriate evaluation metric for highly imbalanced datasets, as is the case for virulence factor and toxin prediction.

In our study, the models produce a predicted class probability (referred to as “probability” in the manuscript) that reflects the model’s likelihood that a given sequence belongs to the positive class.
ROC curves primarily assess the trade-off between sensitivity and specificity across decision thresholds, but they can be misleading under strong class imbalance because a high true negative rate can inflate performance estimates even when positive class prediction is poor. We relied on the Matthews Correlation Coefficient (MCC), which is widely recognised as a robust and informative metric for imbalanced binary classification, as it accounts for all four confusion matrix components. As written in the manuscript (lines 280-284):

“The performance evaluation is 280 based on the Matthew correlation coefficient (MCC) and the precision (to reduce the number of false positive VF and toxin-associated predictions), taking into account the dataset imbalance (a higher number of “non-toxin” and “non-VF” sequences compared to “toxin-associated” and “VF” sequences in the test subsets).”

Our bar plot across confidence score thresholds shows how prediction quality (measured by MCC and precision) changes as probability cutoffs are applied. This approach directly addresses the practical use case of selecting an operating threshold tha balances sensitivity and precision in metagenomic discovery settings, rather than providing a single aggregate curve.

We believe this evaluation strategy is appropriate given the data characteristics and the use case addressed in this study.

We have added the following text to the manuscript to clarify the probability concept (Lines 274-276):

“The VF and toxin-associated modules (Figure 1) were evaluated across different ML models-predicted probability thresholds, corresponding to the model's confidence in the positive class (VF or toxin-associated). Analyses were performed on the full te dataset and on subsets of the test dataset.”

Improvements
15. The claims of the authors about the machine learning models for VF and toxin prediction being more accurate than similarity models is, to my understanding, not proved in the article. If it is only compared to PathoFact, which was created wi a dataset made years ago, the higher performance could easily be because of more complete datasets. A fair comparison of a improved performance should be done with the same dataset (PathoFact but with the dataset collected for PathoFact 2.0). Moreover, the results only show that PathoFact 2.0 predicts more toxins and virulence factors than PathoFact.

R/ We thank the reviewer for the comment. PathoFact 2.0 supports two distinct types of input, and the benchmarking strateg differs accordingly:

Protein sequence input
For protein-level predictions, we benchmarked the machine-learning models implemented in PathoFact 2.0 against state-of-the-art tools for the same task: VirulentHunter (for virulence factors), ToxinPred2 and CSM-toxin (for toxins). These comparisons were conducted on independent test datasets to evaluate model generalisation.
Our toxin model is designed to predict toxin-associated proteins, rather than toxin proteins alone. We have made this distinction clear in the title of the manuscript (i.e., Toxin-associated) and throughout the manuscript. To our knowledge, no existing model addresses this broader prediction task. For benchmarking, we compared our approach with a model that predicts only toxin proteins. To ensure a fair comparison, we limited the test dataset to toxin proteins for this evaluation.
ToxinPred2 is widely used and designed for protein sequences, whereas ToxinPred3 is for peptide sequences; therefore, following the ToxinPred developers' recommendation, we used ToxinPred 2.0. In addition, we also used the same test datase

to benchmark the CSM-toxin.
As described in the Toxin-Associated Prediction Updates, our dataset intentionally includes both effector toxin proteins and toxin-associated proteins, such as antitoxins, regulatory factors, and accessory proteins (lines 192-203):
“It is essential to note that this dataset encompasses both effector toxin proteins and their associated proteins, including antitoxins, regulators, and accessory proteins. This offers three main benefits: 1) Recent reports suggest that the same bacterial toxins can function as part of self-inhibiting toxin-antitoxin modules within one organism, while in another organism they have evolved into toxin effectors that are injected into target cells [39,40]. 2) In bacteria, genes located in close proximity frequently exhibit functional associations, such as those co-transcribed within operons. A comprehensive toxin dataset, including both toxins and their associated proteins, facilitates the identification of novel toxins and related genes through their genomic context, referred to as "toxin islands." These islands may be involved in toxin biosynthesis, processing or secretion, and may also confer immunity or facilitate horizontal gene transfer among bacterial populations. Notably, they a often rich in mobile genetic elements [41]. 3) A large database improves the performance of machine learning classification methods [42]”.

Contig-level input
For contig-based analyses, the available tool landscape is much more limited. MetaVF and PathoFact1.0 are, to our knowledg the only tools that directly support the prediction from assembled contigs. As explained in the manuscript (lines 394-396):

“To our knowledge, no other method is available to predict VFs from contig sequences and identify plasmid-encoded or prophage-associated VFs”.

MetaVF relies primarily on similarity-based approaches, whereas PathoFact1.0 is indeed the previous version of our pipeline. The comparison at the contig level, therefore, serves two purposes: (i) to benchmark against the only available similarity-based method, and (ii) to demonstrate the improvements introduced in the updated PathoFact 2.0 framework. As explained i lines 400-402, MetaVF cannot detect VF in known pathogens, whereas PathoFact 2 can, demonstrating the limitations of the homology-based approach and the advantages of machine-learning-based models and approaches.

Following the reviewer's comment and to include more tools for comparison, we predicted protein sequences from the contigs and used them as input to VirulentHunter, Toxinpred2, and CSM-toxin, and included these results in Figure 7 and the new Figure 8. The following lines, 394-407, describe the performance of VirulentHunter:

“To our knowledge, no other method is available to predict VF from contig sequences and identify plasmid-encoded or prophage-associated VF. However, we included VirulentHunter in the comparison. Because VirulentHunter does not accept contig sequences as input, protein-coding genes were first predicted from contigs using Pyrodigal-gv and the resulting protein sequences were subsequently analysed. PathoFact 2.0 consistently detected a greater number of VFs than both PathoFact 1.0 and MetaVF (Figure 7, Supplementary Figures S2–S3). Notably, MetaVF failed to identify any VFs in five of the ten pathogenic reference strains tested, highlighting its limited ability to detect VFs and demonstrating the advantages of machine-learning-based models over homology-based approaches. While VirulentHunter produced substantially more hits than metaVF, yielding predictions comparable in number to PathoFact 2.0, PathoFact 2.0 generally identified more VFs overall (Figure 7). Exception were observed for non-pathogenic strains Bifidobacterium animalis, Bifidobacterium bifidum, Heyndrickxia coagulans, and for the pathogenic Ralstonia mannitolilytica strain Guangzhou-RMAB10, where VirulentHunter predicted slightly more VFs.”

and lines 414-423, describing the performance of CSM-Toxin and Toxinpred2, were included in the manuscript:

“In addition, the PathoFact 2.0 toxin-associated module was compared to PathoFact 1.0 (Figure 8, Supplementary Figures S4 S5) as well as CSM-Toxin and ToxinPred2 (Figure 8). As CSM-Toxin and ToxinPred2 do not accept contig sequences as input, protein sequences were first predicted from contigs using Pyrodigal-gv. PathoFact 2.0 predicted more toxin-associated protei than PathoFact 1.0, demonstrating improved detection capacity. Compared to external tools, CSM-Toxin identified substantia fewer toxins across the tested reference strains. In contrast, ToxinPred2 predicted a comparable number of toxins overall. However, for non-pathogenic strains Bifidobacterium bifidum, Heyndrickxia coagulans, Lactobacillus acidophilus, and for pathogenic strains Streptococcus gallolyticus subsp. gallolyticus and Streptococcus parasuis B26, ToxinPred2 identified more predicted toxins than PathoFact 2.0.”

The conclusions regarding the performance of the machine-learning models are therefore based on comparisons with contemporary ML-based approaches (VirulentHunter, ToxinPred2, CSM-Toxin) and MetaVF, not solely based on comparisons with the results of PathoFact.

16. The creation of the dataset, for training and most importantly for testing, is rather unclear and described all over the article. I recommend creating a section for it, to understand better the filtering, maybe a figure (could go in the supplementa[illegible] material, if necessary), and include the amount of data in each test set. The authors seem to have put a lot of effort on the testing sets (including trying to avoid testing with the same data that the models are trained with) but it gets diluted in the article and, in consequence, the test results are difficult to evaluate.

R/We thank the reviewer for the helpful comment. To address concerns about clarity regarding dataset creation, we have now [illegible] added a new section, Pipeline Updates, and two new figures, Figures 2 and 3, lines 148-151:

“We implemented thorough updates across all PathoFact modules. Notably, we developed two new machine-learning models: one to predict virulence factors and another to identify toxin-associated proteins. A schematic diagram of the dataset construction is shown in Figure 2. In the sections below, we detail the updates to each module.”

and 266-268 in the manuscript:

“Figure 3 provides a schematic overview of the benchmarking datasets used for the VF and toxin-associated modules.”

Figure 2 illustrates the construction of the toxin-associated, virulence factor, and non-toxin/non-VF datasets, and Figure 3 shows the generation of the different test subset datasets. These figures provide a clear overview of the filtering steps and subset creation, ensuring transparency in how the training and test data were defined and the benchmarking was performed. In addition, we have included comprehensive performance data for the PathoFact 2.0 toxin-associated and virulence factor prediction modules in the Supplementary Tables. Supplementary Table S3 reports evaluation of the toxin-associated module, including class distributions (Negative, non-toxin; Positive, toxin-associated), confusion matrix counts alongside performance[illegible] metrics—accuracy, precision, recall, F1 score, and Matthews correlation coefficient—across test subsets stratified by sequenc[illegible] similarity to the training set. Supplementary Table S4 provides corresponding data for the virulence factor module. To facilita[illegible] benchmarking against other tools, Supplementary Table S5 summarises virulence factor prediction performance for PathoFac[illegible] 2.0 at various predicted probability cutoffs compared with VirulentHunter. At the same time, Supplementary Table S6 presen[illegible] the performance of PathoFact 2.0 for predicting toxin proteins at different cutoffs, alongside that of ToxinPred2.
Together, these additions clarify the dataset construction, provide detailed information on the test sets, and present all relevant performance results, addressing the reviewer’s concern regarding the evaluation of test results.

17. The results against VirulentHunter are impressive, outperforming a fine-tuned language model. While I do not doubt that the authors are thorough in their methods, such claims require more testing. Testing using external databases (not created b[illegible] the authors, maybe the same used by VirulentHunter or other models validated experimentally such as pLM4VF) would suppo[illegible] such claims.

R/ We thank the reviewer for the comment.

VirulentHunter is a sophisticated framework that not only predicts whether a protein is a virulence factor but also assigns it t[illegible] specific functional categories. To our knowledge, this is the only tool that performs both tasks and is based on deep learning. Furthermore, as reported by the VirulentHunter authors, VirulentHunter outperformed other virulence factor predictors (MP4 [49], VirulentPred 2.0 [50], and DeepVF [51]).

In addition, several other VF prediction tools either do not provide their training and test datasets or are no longer actively maintained (e.g., VF-Pred https://pubmed.ncbi.nlm.nih.gov/37979206/, DeepVF http://deepvf.erc.monash.edu/), limiting the[illegible] suitability for benchmarking.

For these reasons, we selected VirulentHunter for benchmarking. However, this multi-class prediction is trained on a relativel[illegible] small dataset, increasing the risk of overfitting. This behaviour is evident in Figure 5B, where VirulentHunter’s performance decreases substantially as sequence similarity to the training set drops from 100% to 80%, indicating reduced generalisation [illegible] more divergent sequences.

We realised that the positive (virulence factor) datasets used by VirulentHunter and PathoFact 2.0 are highly similar and derived from the same sources. Using these datasets without removing similar sequences for external validation would therefore not provide an independent benchmark. Moreover, our negative dataset (i.e., “non-VF”) includes proteins from known non-human pathogenic organisms (bacteria and viruses). We further curated the dataset (i.e., “non-VF”) by removing any proteins predicted as virulence factors or toxins by PathoFact1. This approach reduces the likelihood of false negatives a[illegible]

broadens taxonomic coverage, including bacterial and viral proteins.

To ensure a fair and reliable comparison, we developed independent subset-based test datasets in which any sequence prese in the VirulentHunter training set (100% identity, ≥80% coverage) was removed. This ensures that neither model was traine on sequences that appear in the test set. In addition, by stratifying test sets by decreasing levels of sequence similarity to th PathoFact 2.0 training data, we were able to explicitly assess model performance across increasingly divergent proteins. This study demonstrates that PathoFact 2.0 maintains consistent performance even when test sequences share no more than 40% similarity with the training set.

Regarding pLM4VF, we note that the publicly available experimentally validated test datasets for Gram-positive and Gram-negative bacteria contain only 922 sequences in total (461 positives and 461 controls). After removing sequences with greate than 99% similarity and coverage of 80% relative to the PathoFact 2.0 and VirulentHunter training datasets, only 73 VF sequences and 452 non-VF sequences remain.

We examined additional test datasets from VirulentPred2 (https://bioinfo.icgeb.res.in/virulent2/down.html) and the MSA-VF predictor (https://github.com/kimtaegyuu/MSA-VFpredictor/tree/main/data). After removing sequences with over 99% similarity and at least 80% coverage relative to the training datasets of PathoFact 2.0 and VirulentHunter, only 10 VF sequences and 561 non-VF sequences remained in VirulentPred2.0, while the MSA-VF predictor dataset retained 48 VF sequences and 561 non-VF sequences.

Although these datasets are valuable, their limited size prevents the subset-based similarity stratification needed for a robustness analysis comparable to the one reported, which utilises between 336 and 4,171 VF sequences and 3,904 to 8,106 non-VF sequences across various subset test datasets.

We believe that our evaluation strategy, based on independent, curated test datasets and explicit control of sequence similarity, provides a rigorous and conservative assessment of model generalisation. To account for this and the reviewer’s concern, we have modified the paragraph to clarify this (lines 314-323):

“Since VirulentHunter and Pathofact 2.0 employ a similar method to generate the “VF dataset” for model training, we remove sequences from the Pathofact 2.0 test dataset that have 100% identity (≥80% coverage) to the VirulentHunter training dataset, resulting in a “clean VF test dataset” (Figure 3). This ensures that neither model used the test sequences for training We applied the same test-subset approach described earlier: the subset datasets were created based on sequence similarity t the Pathofact 2.0 training dataset, with similarity ranging from 40% to 100% and 80% coverage of the “clean VF test datase (Figure 3). By stratifying test sets by decreasing sequence similarity to the training data, we explicitly evaluated model performance across progressively more divergent proteins. This study demonstrates that PathoFact 2.0 maintains consistent performance even when test sequences share no more than 40% similarity with the training set (Figure 5B)”.

Lines 343 -345:

“As shown in Figure 5 (and in the Supplementary Tables S5 and S6), Pathofact 2.0 VF and toxin-associated modules exhibite higher MCC values across different test subsets compared to VirulentHunter, ToxinPred2 and CSM-toxin.”

18. The comparative on different bacterial strains gives more questions than answers. Are all those VF and toxins found on E coli experimentally validated? How much overlap is there between pathogenic and non-pathogenic E. coli? Are all of the same type?

R/ We thank the reviewer for the comment. However, we think that a full biological interpretation of virulence factors (VFs) across diverse E. coli strains would constitute an independent study. The aim of our analysis is therefore not to exhaustively characterise virulence in E. coli, but to use this well-studied species as a representative example to illustrate the complexity c VF prediction and interpretation.

Not all virulence factors and toxins identified in our analysis have been experimentally validated in E. coli. This limitation is inherent to current VF databases, which typically include both experimentally confirmed factors and homologs inferred from sequence similarity. It is known that non-pathogenic E. coli strains can harbour genes annotated as virulence factors without causing disease, indicating that the presence of VF genes alone is insufficient to infer pathogenicity.

There is substantial overlap in predicted VF repertoires between pathogenic and non-pathogenic E. coli, and the strains analysed do not belong to a single pathotype. This overlap is well documented and reflects that many virulence-associated genes are variably distributed, are frequently located on mobile genetic elements (MGEs), and require specific genomic and regulatory contexts to contribute to disease.

This complexity is precisely one of the motivations for our study. As shown in Figure 6, pathogenic E. coli strains are enriched

for virulence and toxin-associated proteins associated with MGEs, including plasmids and prophages, compared to non-pathogenic strains. These observations emphasise that distinguishing VFs relevant to infection requires more than standalone VF prediction.
PathoFact 2.0 addresses this challenge by integrating multiple layers of contextual information, including MGE association, secretion signals, and biosynthetic gene cluster localisation. This allows users to move beyond binary VF presence/absence predictions toward a more biologically meaningful interpretation of virulence potential.
Our results highlight that the relationship between predicted virulence factors and disease is more complex than often assumed, and that integrated, comparative approaches are essential for robust interpretation.
The following lines were added to the manuscript to highlight this aspect (lines 365-371):

"Figure 6 shows distinct differences in virulence and toxin-related profiles between pathogenic and non-pathogenic E. coli strains, especially regarding virulence- and toxin-associated proteins encoded on MGEs, such as plasmids and prophages. Nonetheless, analysis of individual VF predictions reveals considerable overlap in the number of VF genes detected across bot pathogenic and non-pathogenic strains. This highlights that the presence of a VF gene is not a reliable marker of pathogenicit and emphasises the importance of considering genomic and functional context when assessing virulence potential".

19. Overall, there is a good amount of work on this project, but the article still has a lot of unanswered questions. It is a bit unclear the strengths of PathoFact2, as well as its weaknesses (any model has). Could be its speed, could be having plenty o tools contained in a pipeline.

R/ We thank the reviewer for the comment.
PathoFact 2.0 represents a clear advance in metagenomic analysis by integrating the detection of antimicrobial resistance genes (ARGs), virulence factors (VFs), toxin-associated proteins, signal peptides, mobile genetic elements (MGEs), and biosynthetic gene clusters (BGCs) within a single, streamlined pipeline. Unlike many existing tools that focus on individual aspects of pathogenicity, PathoFact 2.0 provides a comprehensive, multi-layered view that captures both gene presence and genomic context, improving interpretability and enabling a holistic assessment of microbial pathogenic potential. The pipeline strengths, including its integrated design and computational efficiency, are illustrated in Figures 1, 4, 5 and Table 1.
We acknowledge several inherent limitations (lines 452-473). Non-pathogenic bacterial strains can also encode genes annotated as VFs or toxins, which makes PathoFact 2.0 most useful as an initial screening tool to identify candidates for furth comparative analyses. While the pipeline provides probability scores for VF- or toxin-associated proteins, establishing a definitive link to pathogenicity requires experimental validation. PathoFact 2.0 does not directly classify specific VF or toxin types (e.g., adhesins or genotoxins), but it provides detailed annotations of conserved protein domains (CDD) to facilitate functional inference. Additionally, the pipeline is designed primarily for bacteria-enriched metagenomic data, with predictions interpreted particularly in the context of human pathogens.
Despite these expected limitations, PathoFact 2.0's integrated approach, speed, and multi-layered contextual analysis make i a versatile and powerful tool for studying microbial virulence and resistance in complex microbial communities. The following lines were added to the manuscript (lines 487-492):

"PathoFact 2.0 represents a major advance in metagenomic analysis by integrating the detection of ARGs, VFs, toxins and toxin-associated proteins, signal peptides, MGEs, and BGCs within a single, streamlined pipeline. Unlike existing tools that focus on individual aspects of pathogenicity, PathoFact 2.0 provides a comprehensive, multi-layered view that captures both gene presence and genomic context, improving interpretability and enabling a holistic assessment of microbial pathogenic potential".

20. I would also appreciate a better description of the report that PathoFact 2.0 produces. If its strength is the virulence and toxin prediction, more tests must be performed (as described above). This would be very beneficial for possible users of the model.

R/ We thank the reviewer for highlighting the need for a clearer description of the reports produced by PathoFact 2.0 and the utility for users. In response, we have added a dedicated paragraph to the manuscript (Lines 430–451; PathoFact 2.0 Output Structure):

"PathoFact 2.0 Output Structure
"PathoFact 2.0 creates a structured output directory that summarises predictions from all analysis modules, including VFs, toxin-associated proteins, ARGs, MGEs and BGCs. Each module generates dedicated result files corresponding to the underlyi prediction tools (Supplementary File S1).
The primary summary file, combined_report.tsv, provides an integrated overview of high-confidence predictions across all

modules in a tabular format. This table includes key information such as protein identifiers, bit scores (from HMM profiles), machine-learning prediction scores, and outputs from DeepARG, RGI, SignalP, GenoMad, and antiSMASH, thereby supporting downstream interpretation and candidate prioritisation.
Proteins with prediction probabilities below user-defined thresholds but containing conserved domains identified by toxin-associated or VF HMM profiles are reported in ambiguous_TOX_hits_rep_prot.tsv and ambiguous_VF_hits_rep_prot.tsv. Thes[illegible] lower-confidence candidates may warrant further investigation in comparative or experimental analyses.
High-confidence predictions are reported in amr_hits_rep_prot.tsv, TOX_hits_rep_prot.tsv, and VF_hits_rep_prot.tsv, which summarise features exceeding user-defined probability thresholds and include protein identifiers, bit scores, machine-learnin[illegible] predictions, signal peptide predictions, and genomic context information, such as association with prophages or plasmids identified by GenoMad.
In addition, PathoFact 2.0 generates a dedicated Group_of_sequence directory containing FASTA files of representative prote[illegible] sequences grouped by functional category (VFs, toxin-associated proteins, antimicrobial resistance genes, and combined hits[illegible] together with conserved domain (CDD) annotation tables for predicted VFs and toxin-associated proteins. These files are designed to facilitate downstream analyses, including comparative genomics and functional characterisation."

This paragraph clearly describes the organisation and content of the PathoFact 2.0 output directory. We have also included a supplementary figure that illustrates the output directory structure (Supplementary File S1). This section explicitly details the key output files generated for virulence factor, toxin, and antimicrobial resistance predictions. It describes the integrated combined_report.tsv and explains the separation of high-confidence and ambiguous predictions based on user-defined probability thresholds.
We further clarify that PathoFact 2.0 generates a dedicated Group_of_sequence directory. This directory contains FASTA files [illegible] representative protein sequences grouped by functional category: virulence factors, toxins, and antimicrobial resistance gene[illegible] It also includes conserved domain (CDD) annotation tables for predicted virulence factors and toxins. As described in the revised manuscript, the pipeline provides high-confidence prediction tables and lower-confidence candidate lists that are supported by conserved-domain evidence. It also offers integrated contextual information, including secretion signals and associations with mobile genetic elements.
This structured reporting enables efficient interpretation of results, candidate prioritisation, and downstream analyses, including comparative genomics and functional characterisation. Together with the output directory schema provided in Supplementary File S1, these additions clearly demonstrate the strengths of PathoFact 2.0's reporting and substantially enhance its usability for the community. It thus represents a strength of PathoFact 2.0 in addition to the strengths highlighte[illegible] in our response to the comment 19.

21. Moreover, in a more technical note, I recommend the authors to add a test sample for easy testing of the model in their repository.

R/ We thank the reviewer for the comment. As specified in the Data Availability section of the original and revised manuscrip[illegible] the datasets for training and testing the VF and Toxin modules are available on Zenodo (https://zenodo.org/records/17593961) because they are too large to store on GitLab. Additionally, in our GitLab repository, we have included a sample test to facilitate easy pipeline testing (to confirm that the pipeline works).

Reviewer #3:

R/ We thank Reviewer 3 for their careful review and constructive criticisms. Their comments have helped us improve the methodological clarity and robustness of the manuscript.

22. *Several methods are available to predict ARGs, VFs, Toxins, and Biosynthetic Gene Clusters. However, the authors selected only a few tools to benchmark PathoFact 2.0. I find this point lacking in the manuscript. To be useful to the scientifi[illegible] community, a more rigorous performance evaluation is needed.

R/ We thank the reviewer for this comment. PathoFact 2.0 is an integrated pipeline, rather than a single prediction model. In this update, we introduce two new prediction modules for virulence factors and toxin-associated proteins, alongside existing modules for antimicrobial resistance genes (ARGs), biosynthetic gene clusters, signal peptides, and functional annotation. Th[illegible] pipeline supports both protein and contig-level inputs.
We think benchmarking against all available tools is not feasible, as it would require evaluating multiple tools for each functional category and input type, resulting in an impractical number of comparisons. In addition, few tools support contig-level input, and several do not provide accessible training or test datasets, limiting the ability to conduct fair and unbiased benchmarking. We therefore selected representative, widely used and state-of-the-art tools for evaluation, which are

VirulentHunter and metaVF for VFs and ToxinPred2 for toxins. Alongside Toxinpred2, we have now included a new tool for benchmarking toxin prediction, CSM-toxin.

PathoFact 2.0 further extends existing approaches by predicting toxin-associated proteins, rather than toxin proteins alone, consistent with current strategies for virulence factor prediction (e.g, regulatory proteins are also included). The principal contribution of PathoFact 2.0 is its integrative design, enabling simultaneous prediction of ARGs, virulence factors, toxin-associated proteins, biosynthetic gene clusters, and signal peptides, with results summarised in coherent output tables. This integration supports comprehensive pathogenicity profiling of genomic and metagenomic data.

In addition, as written in the manuscript (lines 104-109):

“PathoFact, a pipeline first introduced in 2020, integrates ARG, VF, and bacterial toxin prediction from metagenomic data into single tool [22]. Since the publication of PathoFact, several tools have been implemented to predict ARGs, VFs, and bacterial toxins [23–25]. Only one tool, HyperVR [24], has attempted to predict them simultaneously. However, HyperVR’s repository no longer available online, and the Zenodo archive from its original submission lacks the necessary databases, hence renderi it unusable.”

Specifically, for VFs and Toxins, PathoFact 2.0 supports two distinct types of input, and our benchmarking strategy reflects th distinction:

1. Protein sequence input

For protein-level predictions, we benchmarked the machine-learning models implemented in PathoFact 2.0 against state-of-the-art tools: VirulentHunter (virulence factors), ToxinPred2.0 (toxins) and CSM-Toxin (toxins).

Furthermore, our evaluation strategy goes beyond a simple tool comparison. We constructed independent, curated test datasets in which any sequence present in the VirulentHunter training set (100% identity, ≥80% coverage) was removed, ensuring that no test sequence was seen during model training. By stratifying test sets according to decreasing sequence similarity to the PathoFact 2.0 training data, we explicitly assessed performance across increasingly divergent proteins. Remarkably, PathoFact 2.0 maintains consistent performance even when test sequences share ≤40% similarity with the training set.

This methodology provides a rigorous, conservative, and reproducible assessment of model generalisation.

2. Contig-level input

For contig-based analyses, the available tool landscape is much more limited. To our knowledge, MetaVF and PathoFact are th only tools capable of predicting directly from assembled contigs. MetaVF relies primarily on similarity-based approaches, whereas PathoFact represents the previous version of our pipeline. Benchmarking at the contig level thus serves two purpose (i) to compare against the only available similarity-based method, and (ii) to demonstrate the improvements introduced in PathoFact 2.0. As noted in lines 400–402, MetaVF fails to detect VFs in certain known pathogens, whereas PathoFact 2.0 succeeds, highlighting the limitations of homology-based methods and the advantages of our machine-learning approach.

We benchmarked PathoFact 2.0 to PathoFact (its predecessor), ToxinPred2, CSM-Toxin, VirulentHunter, and metaVF. We believe this evaluation approach yields results that are reliable, scientifically significant, and practically applicable to the community.

23. *It is not fully clear how the "false" sequences were chosen. Ideally, they should be similar to known resistance genes, bu should not confer resistance.

R/ We thank the reviewer for the comment. We would like to clarify that PathoFact 2.0 does not benchmark ARG prediction tools. ARG prediction is carried out using three widely used, established tools.

24. *Details of the parameters used to create the HMM models are not mentioned in the manuscript. The performance of the updated HMMs in comparison to the older version is not shown.

R/ We thank the reviewer for the comment. The creation of the HMMs was introduced in lines 205-206 of the manuscript. We have also added a new section in Methods, HMM profiles, lines 551-558:

“Profile hidden Markov models are probabilistic models built from a multiple sequence alignment that encode, for each alignment position, the position-specific probabilities of residues and insertions/deletions, turning the alignment into a positio specific scoring system for detecting homologous sequences [67]. The FASTA files of conserved-domain multiple sequence alignments for each CDD [26] family (https://ftp.ncbi.nih.gov/pub/mmdb/cdd/fasta.tar.gz) were downloaded. Pyhmmer v0.10.14 [68] was used to obtain HMM profiles for each CDD family and to perform protein sequence searches against the CD HMM family profiles.”

Unlike PathoFact, PathoFact 2.0 does not use HMMs for predictions; instead, we rely on machine-learning models. The HMMs

PathoFact 2.0 are used solely for functional annotation, and therefore, a direct performance comparison with the previous version is not applicable.

25. *It would be interesting to show how updates in DeepARG, RGI, and AMRFinderPlus have improved the performance of PathoFact 2.0 over version 1.0.

R/ We thank the reviewer for the comment. The performance comparison for updates to DeepARG, RGI, and AMRFinderPlus i[illegible] presented in Supplementary Figures S4 and S5. We have added the following lines to the manuscript in the section ARG Prediction Updates (lines 253-255):

"Supplementary Figures S4 and S5 compare the performance of PathoFact 2.0 with that of its predecessor, PathoFact, in identifying ARGs."

26. *I believe the non-pathogenic dataset was constructed using sequences other than those mentioned in the section "Generalities about Machine learning training set-up and 'non-pathogenic". This means that sequences that do not contain th[illegible] mentioned keyword were used as the negative dataset. These sequences include housekeeping genes, which are also too distant from the ARG, VF, etc. The real test of an ML model occurs with data from the grey zone, which has properties of bot[illegible] negative and positive examples. The authors can benchmark the ML model using the grey-zone data to show the efficiency o[illegible] the ML model.

R/ We thank the reviewer for the comment.
We constructed the negative dataset to approximate a biologically realistic non-pathogenic background by including complete[illegible] protein sets from organisms reported as non-pathogenic to humans.
In addition, we selected SwissProt sequences lacking ARG, VF, and toxin keywords [KW-0568 (pathogenesis-related protein), KW-0843 (virulence), KW-0800 (toxin), KW-0046 (antibiotic resistance), KW-9995 (disease)] and limited to bacteria (taxonomy_id 2), archaea (taxonomy_id 2157), fungi (taxonomy_id 4751), and viruses (taxonomy_id 10239). To reduce the inclusion of proteins with potential virulence-associated functions that are not captured by keyword-based annotation alone, [illegible] further filtered the dataset by removing proteins predicted as virulence factors or toxins by PathoFact 1.0.
Notably, the inclusion of housekeeping genes from non-pathogenic microorganisms can be considered a feature rather than a limitation, as several well-characterised housekeeping proteins in pathogenic bacteria have been shown to perform virulence-associated functions ("moonlighting" proteins), including adhesion, immune modulation, and tissue invasion[1,2].
Representative examples include glyceraldehyde-3-phosphate dehydrogenase (VF0015 in VFDB)[3], enolase [1,2], elongatio[illegible] factor Tu (VF0460 in VFDB) [4,5], GroEL [1,6,7], and DnaK [1,8]. This approach therefore maintains a realistic negative dataset while minimising false negatives within PathoFact's operational scope.
1. Henderson B, Martin A. Bacterial virulence in the moonlight: multitasking bacterial moonlighting proteins are virulence determinants in infectious disease. Infect Immun. American Society for Microbiology; 79:3476–912011;
2. Henderson B, Martin A. Bacterial moonlighting proteins and bacterial virulence. Curr Top Microbiol Immunol. Curr Top Microbiol Immunol; 358:155–2132013;
3. Pancholi V, Fischetti VA. A major surface protein on group A streptococci is a glyceraldehyde-3-phosphate-dehydrogenase with multiple binding activity. J Exp Med. Rockefeller University Press; 176:415–261992;
4. Barel M, Charbit A. Detection of the interaction between host and bacterial proteins: eukaryotic nucleolin interacts with Francisella elongation factor Tu. Methods Mol Biol. Methods Mol Biol; 1197:123–392014;
5. Granato D, Bergonzelli GE, Pridmore RD, Marvin L, Rouvet M, Corthésy-Theulaz IE. Cell surface-associated elongation facto[illegible] Tu mediates the attachment of Lactobacillus johnsonii NCC533 (La1) to human intestinal cells and mucins. Infection and immunity. Infect Immun; 2004; doi: 10.1128/iai.72.4.2160-2169.2004.
6. Kamiya S, Yamaguchi H, Osaki T, Taguchi H. A virulence factor of Helicobacter pylori: role of heat shock protein in mucosa[illegible] inflammation after H. pylori infection. J Clin Gastroenterol. 27 Suppl 1:S35–91998;
7. Hickey TBM, Ziltener HJ, Speert DP, Stokes RW. Mycobacterium tuberculosis employs Cpn60.2 as an adhesin that binds CD43 on the macrophage surface: M. tuberculosis Cpn60.2 mediates macrophage binding via CD43. Cell Microbiol. Hindawi Limited; 12:1634–472010;
8. Lehner T, Bergmeier LA, Wang Y, Tao L, Sing M, Spallek R, et al.. Heat shock proteins generate β-chemokines which function as innate adjuvants enhancing adaptive immunity. Eur J Immunol. 30:594–6032000;
The following text was added to the revised manuscript (lines 463-470):
"The inclusion of housekeeping genes from non-pathogenic microorganisms reflects a deliberate methodological choice rathe[illegible] than a limitation. Multiple well-characterised housekeeping proteins have been shown to exhibit virulence-associated "moonlighting" functions in pathogenic bacteria, including roles in adhesion, immune modulation, and tissue invasion [56,57] Notable examples include glyceraldehyde-3-phosphate dehydrogenase (VF0015 in VFDB) [58], enolase [59,60], elongation

factor Tu (VF0460 in VFDB) [61,62], GroEL [60,63,64], and DnaK [60,65]. This approach maintains a biologically realistic negative dataset while reducing the risk of misclassifications within the intended scope of PathoFact 2.0.”

Cookie Preference Center

We use cookies which are necessary to make our site work. We may also use additional cookies to analyze, improve and personalize our content and your digital experience. For more information, see our Cookie Policy.

You may choose not to allow some types of cookies. However, blocking some types may impact your experience of our site and the services we are able to offer. See the different category headings below to find out more or change your settings.

Allow all

Manage Consent Preferences

Strictly Necessary Cookies

Always active

These cookies are necessary for the website to function and cannot be switched off in our systems. They are usually only set in response to actions made by you which amount to a request for services, such as setting your privacy preferences, logging in or filling in forms. You can set your browser to block or alert you about these cookies, but some parts of the site will not then work.

Functional Cookies

These cookies enable the website to provide enhanced functionality and personalisation. They may be set by us or by third party providers whose services we have added to our pages. If you do not allow these cookies then some or all of these services may not function properly.

Performance Cookies

These cookies allow us to count visits and traffic sources so we can measure and improve the performance of our site. They help us to know which pages are the most and least popular and see how visitors move around the site.

Targeting Cookies

These cookies may be set through our site by our advertising partners. They may be used by those companies to build a profile of your interests and show you relevant adverts on other sites. If you do not allow these cookies, you will experience less targeted advertising.

Cookie List

Clear

Apply Cancel

Consent Leg.Interest

Confirm my choices

Power
