# Supplementary material for: PathoFact 2.0: an integrative pipeline for the prediction of antimicrobial resistance genes, virulence factors, toxins and toxin-associated proteins, and biosynthetic gene clusters in metagenomes
Source: Gigascience. 2026 May 22;15:giag062. doi: 10.1093/gigascience/giag062 (PMC13224393; doi:10.1093/gigascience/giag062)
Supplement: giag062_Authors_Response_To_Reviewer_Comments_revision_1 [file giag062_authors_response_to_reviewer_comments_revision_1.pdf]

Author's Response To Reviewer Comments

Reviewer reports:

We would like to thank reviewers for their thorough review of the manuscript. We appreciate their comments and believe tha

Reviewer reports:
Reviewer #2: Overall, the authors have addressed partially my concerns, which is very satisfactory. Some comments have be
as for example PathoFact predicts toxin-associated (while to other methods might just predict toxins). I believe my comment

Comments 6-9: Issues solved.

R/ We thank the reviewer for their acknowledgement of our satisfactory earlier revisions.

Comment 10: The issue in Figure 4 persists. The y-axis range are not visualized, and they differ from graph to graph. One wo

R/ We thank the reviewer for the comment. In accordance with the comment, we have added the Y-axis range to figures 4 ar

Comments 11-12: Addressed. If the authors have tried and they say this is the best, I believe them.

R/ We thank the reviewer for their acknowledgement of our satisfactory earlier revisions.

Comment 13: I was actually not asking for a detailed description of the parameters. I just believe using "maximum depth" is

R/ We thank the reviewer for the comment. We have added a detailed description of the parameters, lines 224-226 and 244-

Comment 14: Unfortunately, I must strongly disagree with the authors. Yes, MCC is a very good approach to evaluate models
been proven wrong in different articles, among:
* McDermott MB, Zhang H, Hansen LH, Angelotti G, Gallifant J (2024) A Closer Look at AUROC and AUPRC under Class Imbal
https://eur01.safelinks.protection.outlook.com/?url=https%3A%2F%2Fproceedings.neurips.cc%2Fpaper_files%2Fpaper%2F
Conference.html&data=05%7C02%7Cpaul.wilmes%40uni.lu%7C7db2d2e5b64f482096f308de9486c817%7C445a9c950f9d49
9vdIimLRie4cLhMIX4LYxiXJoNAiy795f698%3D&reserved=0

* Richardson E, Trevizani R, Greenbaum JA, Carter H, Nielsen M, Peters B (2024) The receiver operating characteristic curve
https://eur01.safelinks.protection.outlook.com/?url=https%3A%2F%2Fdoi.org%2F10.1016%2Fj.patter.2024.100994&data=
DAwMCIsIlAiOiJXaW4zMiIsIkFOIjoiTWFpbCIsIldUIjoyfQ%3D%3D%7C0%7C%7C%7C&sdata=QkiL2lT8RFC05OR8FDLmfa28N9

R/ We thank the reviewer for this important clarification and for highlighting recent literature on the use of AUROC under clas

Here is an example of how the AUROC figures compare PathoFact 2.0 and VirulentHunter (Supplemental Figure S3)

Here is an example of the AUROC figure comparing PathoFact 2.0 and CSM-Toxin (Supplemental Figure S4)

Such AUROC figures are not possible with Toxinpred2, since its predictions are based on a combination of factors rather than
scores; therefore, AUROC curves are not possible.

AUROC evaluates a classifier's ranking performance across all possible thresholds, whereas our study focuses on selecting an
balanced evaluation even under strong class imbalance. Nonetheless, the AUROC figures have been included in the manuscrip

Comment 15: The answer from the authors do not have anything to do with my comment. So I will write it more clearly: if yo

Right now there is only a comparison on how many more VF and toxins PathoFact2 detects, which is not a Performance evalu

R/ We thank the reviewer for clarifying this point. We agree that benchmarking PathoFact 2.0 against the previous version (P
PathoFact 1.0 accepts contigs as input, whereas PathoFact 2.0 accepts both contigs and protein sequences, representing one
negative dataset used in the benchmark was originally constructed and filtered using PathoFact 1.0, which suppresses false p
Furthermore, AUROC cannot be calculated for PathoFact 1.0 predictions. The virulence factor module combines machine-learn
To avoid biased interpretation, we therefore reported recall only for PathoFact 1.0 on this benchmark dataset. Under these co
100, respectively, which are substantially lower than those obtained with PathoFact 2.0.
We have added this benchmarking analysis to the revised manuscript and clarified that the comparison with PathoFact 1.0 is
“Because the negative dataset was built and then filtered using PathoFact 1.0, the precision for PathoFact 1.0 is 1, which affe
and MCC than metaVF.”

Comment 16-17: Issues addressed.

R/ We thank the reviewer for their acknowledgement of our satisfactory earlier revisions.

Comment 18: Yes, I agree a full biological assessment of VF and toxins in different E. coli would be an independent study. I a
times each model detected a VF or a toxin. Besides clearly promoting recall over precision, there is no indication on those pre
number of VFs. I am not saying that is a bad thing that it predicts more, but if the authors are going to be using those results
These types of claims also get a very vague appearance as "Our improved pipeline, PathoFact 2.0, offers significant improvem
authors insist in using those results, they would be much more informative showing the overlap of VF and toxins predicted by

R/ We thank the reviewer for the comment. We have used DNA (gene) sequences as input for PathoFact 1.0 and MetaVF, and

In addition, we have clearly shown that MetaVF does not predict VF in known pathogenic strains, highlighting a major limitati

PathoFact 2.0 and PathoFact 1.0 are pipelines, and we have demonstrated the advantages of PathoFact 2.0 over PathoFact 1

To address the reviewer's comment, we have modified Figures 5, 7 and 8 and added two new supplementary figures, S6 and

In addition, we have removed the subtitle benchmarking to avoid confusion (between lines 405 and 406 of the revised manus

We have modified the sentences: “PathoFact 2.0 consistently detected a greater number of VFs than both PathoFact 1.0 and

As written in the manuscript after the sentences "Our improved pipeline, PathoFact 2.0, offers significant improvements over

“SignalP has been upgraded and made optional to further optimise performance, providing users with flexibility based on thei
identifying MGEs, including plasmids and phages linked to ARGs, VFs, toxins and toxin-associated proteins across various bac

Comment 19: More or less addressed (also by previous comments).
Comment 20-21: Addressed
Comment 22-26: Agree with authors.

R/ We thank the reviewer for their acknowledgement of our satisfactory earlier revisions.

Editor Comments:
Please be informed that Reviewer 3 was unable to accept the invitation to provide comments on your revised manuscript. Ins

In addition, GigaScience has also published a number of relevant papers on metadata standardization, FAIR data practices, a

Citing some of these articles—where appropriate in the Background or Discussion—would help frame your contribution within
1. Kasmanas J C, Magnúsdóttir S, Zhang J, et al. Integrating comparative genomics and risk classification by assessing virule
https://eur01.safelinks.protection.outlook.com/?url=https%3A%2F%2Fdoi.org%2F10.1093%2Fgigascience%2Fgiaf072&data

MDAwMCIsIlAiOiJXaW4zMiIsIkFOIjoiTWFpbCIsIldUIjoyfQ%3D%3D%7C0%7C%7C%7C&sdata=lu3VScoPqNhU85T%2Bral3skR

2. Nachtigall P G, Durham A M, Rokyta D R, et al. ToxCodAn-Genome: an automated pipeline for toxin-gene annotation in ge
https://eur01.safelinks.protection.outlook.com/?url=https%3A%2F%2Fdoi.org%2F10.1093%2Fgigascience%2Fgiad116&data
MDAwMCIsIlAiOiJXaW4zMiIsIkFOIjoiTWFpbCIsIldUIjoyfQ%3D%3D%7C0%7C%7C%7C&sdata=oprnTmJkrIEzP3z%2Bgm7rmx

3. Roach M J, Beecroft S J, Mihindukulasuriya K A, et al. Hecatomb: an integrated software platform for viral metagenomics[J
https://eur01.safelinks.protection.outlook.com/?url=https%3A%2F%2Fdoi.org%2F10.1093%2Fgigascience%2Fgiae020&data
MDAwMCIsIlAiOiJXaW4zMiIsIkFOIjoiTWFpbCIsIldUIjoyfQ%3D%3D%7C0%7C%7C%7C&sdata=At4N2T7tySZC1zzhpHAOn%2B

--
Please also take a moment to check our website at
https://eur01.safelinks.protection.outlook.com/?url=https%3A%2F%2Fwww.editorialmanager.com%2Fgiga%2Fl.asp%3Fi%3
B0eU1hcGkiOnRydWUsIlYiOiIwLjAuMDAwMCIsIlAiOiJXaW4zMiIsIkFOIjoiTWFpbCIsIldUIjoyfQ%3D%3D%7C0%7C%7C%7C&sd

for any additional comments that were saved as attachments.

R/ We would like to thank the editor for the helpful suggestion. The relevant paper has now been included in the introduction

“gSpreadComp (https://doi.org/10.1093/gigascience/giaf072) recently proposed a workflow that integrates comparative geno
while gSpreadComp is valuable for comparative, community-level prioritisation, ARGs are predicted using only one tool, Deep
microbiome-based pathogenicity profiling, and the pipeline accepts only DNA sequences as input”

We also thank you for suggesting additional references. However, papers 2 and 3 were not incorporated because they do not
read and contig levels, which is also beyond the scope of this study.

Editorial Manager®

Cookie Preference Center

We use cookies which are necessary to make our site work. We may also use additional cookies to analyze, improve and personalize our content and your digital experience. For more information, see our Cookie Policy.

You may choose not to allow some types of cookies. However, blocking some types may impact your experience of our site and the services we are able to offer. See the different category headings below to find out more or change your settings.

Allow all
Manage Consent Preferences

Strictly Necessary Cookies

Always active

These cookies are necessary for the website to function and cannot be switched off in our systems. They are usually only set in response to actions made by you which amount to a request for services, such as setting your privacy preferences, logging in or filling in forms. You can set your browser to block or alert you about these cookies, but some parts of the site will not then work.

Functional Cookies

These cookies enable the website to provide enhanced functionality and personalisation. They may be set by us or by third party providers whose services we have added to our pages. If you do not allow these cookies then some or all of these services may not function properly.

Performance Cookies

These cookies allow us to count visits and traffic sources so we can measure and improve the performance of our site. They help us to know which pages are the most and least popular and see how visitors move around the site.

Targeting Cookies

These cookies may be set through our site by our advertising partners. They may be used by those companies to build a profile of your interests and show you relevant adverts on other sites. If you do not allow these cookies, you will experience less targeted advertising.

Cookie List

Clear

Apply Cancel

Consent Leg.Interest

Confirm my choices

Power
