# Supplementary material for: PathoFact 2.0: an integrative pipeline for the prediction of antimicrobial resistance genes, virulence factors, toxins and toxin-associated proteins, and biosynthetic gene clusters in metagenomes
Source: Gigascience. 2026 May 22;15:giag062. doi: 10.1093/gigascience/giag062 (PMC13224393; doi:10.1093/gigascience/giag062)
Supplement: giag062_Authors_Response_To_Reviewer_Comments_revision_2 [file giag062_authors_response_to_reviewer_comments_revision_2.pdf]

Author's Response To Reviewer Comments

Reviewer reports:
Reviewer #2: The authors have addressed all my comments in a satisfactory way. I believe that, even if it is usually hard to communicate through this reviews, that this process is being quite successful.

I. However, by addressing them they have uncovered a new concern, and that is the comparison between PathoFact2 and VirulentHunter/ToxinPred2. The authors say that PathoFact2 predicts a larger amount of Toxins and Virulence factors, but what we see on the Venn diagram's is a bit more complex: There is a large divergence on what is predicted by each method. There is of course overlap, but there is a large amount of sequences predicted by VirulentHunter/ToxinPred2 that are not by PathoFact2. If it was just that PathoFact2 predicts more, the amount of toxins/VF predicted only by VH and TP2 would not be that high (notice, that sometimes it is up to a half of the predictions. This is not per-se something to discard the utility of the tool, but it would be adequate for authors to address it on the paper, and also explain the reason of the deviation between models: what is PathoFact2 missing than the other two predicts as VF/Toxins? Maybe I am missing it.

R/ We appreciate the reviewer's valuable observation.
We would like to highlight that the tools (PathoFact 2.0, VirulentHunter, ToxinPred2, CMS-Toxin) were developed with distinct objectives, training datasets, and prediction strategies, resulting in expected differences.

First, PathoFact 2.0 was developed as a high-throughput metagenomic screening framework designed to balance sensitivity and precision in complex microbiome datasets. To reduce false positives, stringent probability thresholds (0.9 for virulence factors and 0.6 for toxin-associated proteins) were selected based on Matthews correlation coefficient (MCC) and precision analyses across benchmark subsets. In contrast, VirulentHunter applies a more permissive default threshold (0.5), which increases the number of positive predictions, including sequences not detected by PathoFact 2.0.

Second, the biological scope of the models differs substantially. ToxinPred2 is designed to predict toxic proteins using a hybrid approach that integrates Random Forest classification, BLAST similarity, and MERCI motif detection. CSM-Toxin is a deep learning approach for protein toxicity classification, which relies solely on the protein primary sequence. In contrast, PathoFact 2.0 is designed to identify a broader category of toxin-associated proteins, including toxins, toxin-antitoxin system components, secretion-associated effectors, regulators, and accessory proteins. This broader conceptual scope alters the learned feature space and can result in differences in protein classification.

Third, the construction of negative datasets differs between the different tools. PathoFact 2.0 utilises a highly curated non-pathogenic dataset that explicitly includes housekeeping genes from non-pathogenic microorganisms, while filtering out potential antibiotic resistance genes (ARGs), virulence factors, and toxins predicted by PathoFact 1.0. This conservative strategy was adopted because many canonical housekeeping proteins have documented moonlighting virulence-associated functions, such as GAPDH, enolase, EF-Tu, GroEL, and DnaK. Consequently, PathoFact 2.0 is trained to distinguish between proteins with general cellular functions and those with stronger virulence-associated signatures, thereby reducing overprediction in metagenomic datasets.

Additionally, the underlying machine learning architectures differ. VirulentHunter is a deep learning classifier trained to assign proteins to virulence factor categories, whereas PathoFact 2.0 employs machine learning-based prediction. ToxinPred2 further incorporates similarity- and motif-based heuristics that are not present in PathoFact 2.0. These methodological differences are expected to result in overlapping as well as unique predictions.

Importantly, disagreement between tools does not necessarily indicate that one model is missing true virulence factors or toxins. Instead, it reflects distinct operational definitions and optimisation criteria.

Some proteins uniquely predicted by VirulentHunter or ToxinPred2 may represent borderline or low-confidence candidates that PathoFact 2.0 excludes due to stricter thresholds and a conservative training strategy. Conversely, PathoFact 2.0 identifies additional proteins associated with toxin systems that are outside the narrower design scope of the comparator tools.
To clarify this interpretation for readers, the manuscript has been revised to explicitly acknowledge the substantial yet biologically and methodologically expected divergence among prediction tools.

Lines 485-512:
"The divergence between PathoFact 2.0, PathoFact 1.0, VirulentHunter, MetaVF, ToxinPred2 and CSM-Toxin predictions (Figures 7, 8, S6, S9) reflects fundamental differences in model design, training datasets, prediction thresholds, and biological scope, rather than sensitivity alone. PathoFact 2.0 was developed as a conservative metagenomic screening framework and therefore applies stringent machine-learning probability thresholds (0.9 for virulence factors and 0.6 for toxin-associated proteins), selected based on the Matthews correlation coefficient (MCC) and precision benchmarking. In contrast, VirulentHunter uses a lower default threshold (0.5), which increases the number of positive predictions, including those with lower confidence. Similarly, PathoFact 1.0 was built using a positive subset of known virulence factor sequences retrieved from the Virulence Factors Database (which included 8945 sequences), while the negative subset of the training set consisted of protein sequences retrieved from the Database of Essential Genes (DEG) (included 7995 sequences) [67]. The PathoFact 1.0 VF score is based on a combination of ML output and HMM homology, whereas the toxin prediction is based solely on HMM homology.
The prediction scope also differs between methods. ToxinPred2 focuses on toxin protein prediction using a hybrid framework that combines machine learning, BLAST similarity, and MERCI motif detection. CSM-Toxin is a deep learning approach for protein toxicity classification that relies on the protein's primary sequence. PathoFact 2.0 targets a broader class of toxin-associated proteins, including toxins, antitoxins, secretion-associated effectors, regulators, and accessory proteins. Similarly, VirulentHunter was developed as a virulence factor category classifier trained on a relatively small, imbalanced dataset. These methodological differences result in partially overlapping but distinct prediction spaces.
PathoFact 2.0 additionally employs a highly curated negative dataset of non-pathogenic microorganisms, including housekeeping proteins, while filtering out potential antibiotic resistance genes (ARGs), virulence factors, and toxins. This conservative strategy is designed to reduce false-positive predictions in metagenomic datasets, particularly given the documented moonlighting virulence functions of several canonical housekeeping proteins. Consequently, proteins uniquely predicted by VirulentHunter, CSM-Toxin or ToxinPred2 likely include borderline or lower-confidence candidates that are intentionally excluded by the stricter classification framework of PathoFact 2.0."

II. Also, Figure 4B should have y-axis from 0 to 1.

R/ We appreciate the reviewer's valuable observation. Nonetheless, we respectfully disagree with the reviewer's suggestion, as adding a full y-axis would diminish the clarity of the figure and would not contribute additional information. We believe this decision rests with the editor.

em Editorial Manager®

Cookie Preference Center

We use cookies which are necessary to make our site work. We may also use additional cookies to analyze, improve and personalize our content and your digital experience. For more information, see our Cookie Policy.

You may choose not to allow some types of cookies. However, blocking some types may impact your experience of our site and the services we are able to offer. See the different category headings below to find out more or change your settings.

Allow all

Manage Consent Preferences

Strictly Necessary Cookies

Always active

These cookies are necessary for the website to function and cannot be switched off in our systems. They are usually only set in response to actions made by you which amount to a request for services, such as setting your privacy preferences, logging in or filling in forms. You can set your browser to block or alert you about these cookies, but some parts of the site will not then work.

Functional Cookies

These cookies enable the website to provide enhanced functionality and personalisation. They may be set by us or by third party providers whose services we have added to our pages. If you do not allow these cookies then some or all of these services may not function properly.

Performance Cookies

These cookies allow us to count visits and traffic sources so we can measure and improve the performance of our site. They help us to know which pages are the most and least popular and see how visitors move around the site.

Targeting Cookies

These cookies may be set through our site by our advertising partners. They may be used by those companies to build a profile of your interests and show you relevant adverts on other sites. If you do not allow these cookies, you will experience less targeted advertising.

Cookie List

Clear

Apply Cancel
Consent Leg.Interest

Confirm my choices

Power
