# Supplementary material for: PathoFact 2.0: an integrative pipeline for the prediction of antimicrobial resistance genes, virulence factors, toxins and toxin-associated proteins, and biosynthetic gene clusters in metagenomes
Source: Gigascience. 2026 May 22;15:giag062. doi: 10.1093/gigascience/giag062 (PMC13224393; doi:10.1093/gigascience/giag062)
Supplement: giag062_Reviewer_1_Report_original_submission — Reviewer 1 -- 12/10/2025 [file giag062_reviewer_1_report_original_submission.pdf]

Reviewer Report

Title: PathoFact 2.0: An Integrative Pipeline for the Prediction of Antimicrobial Resistance Genes, Virulence Factors, Toxins and Toxin-associated Proteins, and Biosynthetic Gene Clusters in Metagenomes

Version: Original Submission **Date:** 12/10/2025

Reviewer name: Reviewer 1

Reviewer Comments to Author:

The pipeline should be very useful on shotgun metagenomics data analysis.
Aside the ARGs and VFs, the features on signal peptides, toxin predictions, and BGCs in particular for specialised metabolites predictions, are welcome for detailed analysis and understanding of various transmission mechanisms.
I find the approaches very appealing and I think the pipeline could be welcomed by the community.
I only have some minor observations:
- The Methods section should be placed next to the described methods. As it is, at the end of the manuscript, under Methods chapter you can only find Datasets, so a proper formatting of the Methods is required
- there is a 70 blank pages buffer between References and supplementary data
- could you add some future prospects in the manuscript? How well is it going to be maintained - I noticed the update are quite old.
- I would also add some more details to the limitations. For instance it is clear that the pipeline is installable on Linux platforms, but did you considered making it available also for Apple silicon series? More and more researchers use this technology, and it works as good as the Linux distributions. I also tried an install on a M series Apple silicon, but unfortunately, most of the tools in the pipeline lead to multiple errors related to python versions (most of which are old), missing old dependencies versions, libraries, etc.

Methods

Are the methods appropriate to the aims of the study, are they well described, and are necessary controls included? Choose an item.

Conclusions

Are the conclusions adequately supported by the data shown? Choose an item.

Reporting Standards

Does the manuscript adhere to the journal's guidelines on minimum standards of reporting? Choose an item.

Choose an item.

Statistics

Are you able to assess all statistics in the manuscript, including the appropriateness of statistical tests used? Choose an item.

Quality of Written English

Please indicate the quality of language in the manuscript: Choose an item.

Declaration of Competing Interests

Please complete a declaration of competing interests, considering the following questions:

- Have you in the past five years received reimbursements, fees, funding, or salary from an organisation that may in any way gain or lose financially from the publication of this manuscript, either now or in the future?
- Do you hold any stocks or shares in an organisation that may in any way gain or lose financially from the publication of this manuscript, either now or in the future?
- Do you hold or are you currently applying for any patents relating to the content of the manuscript?
- Have you received reimbursements, fees, funding, or salary from an organization that holds or has applied for patents relating to the content of the manuscript?
- Do you have any other financial competing interests?
- Do you have any non-financial competing interests in relation to this paper?

If you can answer no to all of the above, write 'I declare that I have no competing interests' below. If your reply is yes to any, please give details below.

1. No 2. No 3. No 4. No 5. No 6. No

I agree to the open peer review policy of the journal. I understand that my name will be included on my report to the authors and, if the manuscript is accepted for publication, my named report including any attachments I upload will be posted on the website along with the authors' responses. I agree for my report to be made available under an Open Access Creative Commons CC-BY license (http://creativecommons.org/licenses/by/4.0/). I understand that any comments which I do not wish to be included in my named report can be included as confidential comments to the editors, which will not be published.

Choose an item.

To further support our reviewers, we have joined with Publons, where you can gain additional credit to further highlight your hard work (see: https://publons.com/journal/530/gigascience). On publication of this paper, your review will be automatically added to Publons, you can then choose whether or not to claim your Publons credit. I understand this statement.

Yes Choose an item.
