# Supplementary material for: PathoFact 2.0: an integrative pipeline for the prediction of antimicrobial resistance genes, virulence factors, toxins and toxin-associated proteins, and biosynthetic gene clusters in metagenomes
Source: Gigascience. 2026 May 22;15:giag062. doi: 10.1093/gigascience/giag062 (PMC13224393; doi:10.1093/gigascience/giag062)
Supplement: giag062_Reviewer_2_Report_original_submission — Reviewer 2 -- 12/28/2025 [file giag062_reviewer_2_report_original_submission.pdf]

Reviewer Report

Title: PathoFact 2.0: An Integrative Pipeline for the Prediction of Antimicrobial Resistance Genes, Virulence Factors, Toxins and Toxin-associated Proteins, and Biosynthetic Gene Clusters in Metagenomes

Version: Original Submission **Date:** 12/28/2025

Reviewer name: Reviewer 2

Reviewer Comments to Author:

The authors present the pipeline PathoFact2.0, which combines external modules and machine learning algorithms in order to find genes that provoke antimicrobial resistance, virulence and toxicity. They present their work, the improvement from the previous version, as a Technical Note.
For what the authors say, it is the only pipeline available with those characteristics, which makes it clearly a relevant software and article. However, I believe the article requires refinement, as well as new tests that support the authors claims.
Refinements on the article
In the abstract, ARG is used as an abbreviation for Antimicrobial Resistance, not Antimicrobial Resistance Genes.
Overall, the article should be more clear in what the pipeline is made for. It mentions fungi, viral, etc… sequences (which might be found on a metagenomic sample, of course), but to my understanding, all the tools and phenotypes searched for are mostly characteristic of bacteria.
While the introduction offers a good resume of the genes of interests, there are some descriptions that are not particularly accurate. "Human, animal, and environmental microbiomes harbour commensal and pathogenic microorganisms, contributing to the emergence of infectious diseases" seems to say that commensal microorganisms contribute to the emergence of infectious diseases; "ARGs are genetic elements that confer bacterial resistance to antibiotics, acquired via mutations or horizontal gene transfer." seems to say that antimicrobial resistance genes are acquired via mutation (they are not, there is a difference between resistance to antimicrobials provoked by mutations and by genes). I recommend a thorough rewriting of the 6 first paragraphs.
The graphs in Figures 1 and 2 have different Y axes, which are also not shown. This is, to say it lightly, very misleading.
Table 1 would be much more clear as a Figure.
Table 1 cited in line 211 does not exist.
A short description of "dereplication" would help users lacking that knowledge.
The description of the parameters of the machine learning modules are a copy-paste of the variables used by scikit-learn (lines 248 and 268). The description of the machine learning models should be clearer and more detailed, as well as not force the reader to go to the instructions of scikit-learn to check what is the meaning of those parameters.
The authors analyze the performance of the models depending on the "probability" (a term that could definitely use a better introduction) using barplots. A standard to analyze the probability of a model is a

ROC curve.
Improvements
The claims of the authors about the machine learning models for VF and toxin prediction being more accurate than similarity models is, to my understanding, not proved in the article. If it is only compared to PathoFact, which was created with a dataset made years ago, the higher performance could easily be because of more complete datasets. A fair comparison of an improved performance should be done with the same dataset (PathoFact but with the dataset collected for PathoFact2.0). Moreover, the results only show that PathoFact2 predicts more toxins and virulence factors than PathoFact.
The creation of the dataset, for training and most importantly for testing, is rather unclear and described all over the article. I recommend creating a section for it, to understand better the filtering, maybe a figure (could go in the supplementary material, if necessary), and include the amount of data in each test set. The authors seem to have put a lot of effort on the testing sets (including trying to avoid testing with the same data that the models are trained with) but it gets diluted in the article and, in consequence, the test results are difficult to evaluate.
The results against VirulentHunter are impressive, outperforming a fine-tuned language model. While I do not doubt that the authors are thorough in their methods, such claims require more testing. Testing using external databases (not created by the authors, maybe the same used by VirulentHunter or other models validated experimentally such as pLM4VF) would support such claims.
The comparative on different bacterial strains gives more questions than answers. Are all those VF and toxins found on E. coli experimentally validated? How much overlap is there between pathogenic and non-pathogenic E. coli? Are all of the same type?
Overall, there is a good amount of work on this project, but the article still has a lot of unanswered questions. It is a bit unclear the strengths of PathoFact2, as well as its weaknesses (any model has). Could be its speed, could be having plenty of tools contained in a pipeline. I would also appreciate a better description of the report that PathoFact2.0 produces. If its strength is the virulence and toxin prediction, more tests must be performed (as described above). This would be very beneficial for possible users of the model. Moreover, in a more technical note, I recommend the authors to add a test sample for easy testing of the model in their repository.

Methods

Are the methods appropriate to the aims of the study, are they well described, and are necessary controls included? Choose an item.

Conclusions

Are the conclusions adequately supported by the data shown? Choose an item.

Reporting Standards

Does the manuscript adhere to the journal's guidelines on [minimum standards of reporting?] Choose an item.

Choose an item.

Statistics

Are you able to assess all statistics in the manuscript, including the appropriateness of statistical tests used? Choose an item.

Quality of Written English

Please indicate the quality of language in the manuscript: Choose an item.

Declaration of Competing Interests

Please complete a declaration of competing interests, considering the following questions:

- Have you in the past five years received reimbursements, fees, funding, or salary from an organisation that may in any way gain or lose financially from the publication of this manuscript, either now or in the future?
- Do you hold any stocks or shares in an organisation that may in any way gain or lose financially from the publication of this manuscript, either now or in the future?
- Do you hold or are you currently applying for any patents relating to the content of the manuscript?
- Have you received reimbursements, fees, funding, or salary from an organization that holds or has applied for patents relating to the content of the manuscript?
- Do you have any other financial competing interests?
- Do you have any non-financial competing interests in relation to this paper?

If you can answer no to all of the above, write 'I declare that I have no competing interests' below. If your reply is yes to any, please give details below.

I declare that I have no competing interests

I agree to the open peer review policy of the journal. I understand that my name will be included on my report to the authors and, if the manuscript is accepted for publication, my named report including any attachments I upload will be posted on the website along with the authors' responses. I agree for my report to be made available under an Open Access Creative Commons CC-BY license (http://creativecommons.org/licenses/by/4.0/). I understand that any comments which I do not wish to be included in my named report can be included as confidential comments to the editors, which will not be published.

Choose an item.

To further support our reviewers, we have joined with Publons, where you can gain additional credit to further highlight your hard work (see: https://publons.com/journal/530/gigascience). On publication of this paper, your review will be automatically added to Publons, you can then choose whether or not to claim your Publons credit. I understand this statement.

Yes Choose an item.
