# Supplementary material for: PathoFact 2.0: an integrative pipeline for the prediction of antimicrobial resistance genes, virulence factors, toxins and toxin-associated proteins, and biosynthetic gene clusters in metagenomes
Source: Gigascience. 2026 May 22;15:giag062. doi: 10.1093/gigascience/giag062 (PMC13224393; doi:10.1093/gigascience/giag062)
Supplement: giag062_Reviewer_2_Report_revision_1 — Reviewer 2 -- 4/6/2026 [file giag062_reviewer_2_report_revision_1.pdf]

Reviewer Report

Title: PathoFact 2.0: An Integrative Pipeline for the Prediction of Antimicrobial Resistance Genes, Virulence Factors, Toxins and Toxin-associated Proteins, and Biosynthetic Gene Clusters in Metagenomes

Version: Revision 1 **Date:** 4/6/2026

Reviewer name: Reviewer 2

Reviewer Comments to Author:

Overall, the authors have addressed partially my concerns, which is very satisfactory. Some comments have been left unresolved, but that is quite common when communicating by emails/comments. In particular, the article still struggle with comparing to other methods (or previous PathoFacts). It does make sense, in particular with other methods, as for example PathoFact predicts toxin-associated (while to other methods might just predict toxins). I believe my comments below give a clear path of how to have this article ready for publication.
Comments 6-9: Issues solved.
Comment 10: The issue in Figure 4 persists. The y-axis range are not visualized, and they differ from graph to graph. One would expect that in Precision and Recall, the y-axis would go from 0-1; in MCC, from -1 to 1, but it is also very common just to use 0-1. I must insist that the current layout is misleading.
Comments 11-12: Addressed. If the authors have tried and they say this is the best, I believe them.
Comment 13: I was actually not asking for a detailed description of the parameters. I just believe using "maximum depth" is better than "max_depth". But that is also an aesthetic decision, so if the editor is fine with it, it is also fine with me.
Comment 14: Unfortunately, I must strongly disagree with the authors. Yes, MCC is a very good approach to evaluate models (particularly on imbalanced datasets); but if the authors defend that threshold fine-tuning is crucial for the model, AUROC measures are necessary. And the conception of AUROC being not a correct measure for imbalanced datasets have been proven wrong in different articles, among:
* McDermott MB, Zhang H, Hansen LH, Angelotti G, Gallifant J (2024) A Closer Look at AUROC and AUPRC under Class Imbalance. In: Advances in Neural Information Processing Systems. pp 44102-44163 https://proceedings.neurips.cc/paper_files/paper/2024/hash/4df3510ad02a86d69dc32388d91606f8-Abstract-Conference.html
* Richardson E, Trevizani R, Greenbaum JA, Carter H, Nielsen M, Peters B (2024) The receiver operating characteristic curve accurately assesses imbalanced datasets. Patterns 5(6):100994. https://doi.org/10.1016/j.patter.2024.100994
Comment 15: The answer from the authors do not have anything to do with my comment. So I will write it more clearly: if you release a new version of PathoFact (PathoFact2), you should benchmark it against your older version (PathoFact1.0). And if it not only includes an updated database, you should test PathoFact2 against PathoFact1.0 with the new database. Right now there is only a comparison on how many more VF and toxins PathoFact2 detects, which is not a Performance evaluation (see Comment 18).

A proper Performance evaluation (accuracy measures, such as MCC, Precision, Recall, AUROC) is necessary. It has been done against other ML methods, but not against the older method.
Comment 16-17: Issues addressed.
Comment 18: Yes, I agree a full biological assessment of VF and toxins in different E. coli would be an independent study. I am more than familiar with the complexity of the strain. However, the authors use Figure 7 and 8 as "Performance Comparison" of PathoFact2.0 against other methods, and that is not what it is. What we have is just the counts of how many times each model detected a VF or a toxin. Besides clearly promoting recall over precision, there is no indication on those predictions being correct. So, statements such as "PathoFact 2.0 consistently detected a greater number of VFs than both PathoFact 1.0 and MetaVF" are incorrect, as what those graphs show is that PathoFact2.0 consistently PREDICTS a greater number of VFs. I am not saying that is a bad thing that it predicts more, but if the authors are going to be using those results the claims have to change and be very explicit that they are not using quantity as a way of Performance comparison or evaluation. These types of claims also get a very vague appearance as "Our improved pipeline, PathoFact 2.0, offers significant improvements over PathoFact (its predecessor), ToxinPred2, CSM-toxin, VirulentHunter, and metaVF" in the Conclusions. The authors should be much more precise in the conclusions about what are the significant improvements.
Moreover, if the authors insist in using those results, they would be much more informative showing the overlap of VF and toxins predicted by each method evaluated.
Comment 19: More or less addressed (also by previous comments).
Comment 20-21: Addressed
Comment 22-26: Agree with authors.

Methods

Are the methods appropriate to the aims of the study, are they well described, and are necessary controls included? Choose an item.

Conclusions

Are the conclusions adequately supported by the data shown? Choose an item.

Reporting Standards

Does the manuscript adhere to the journal's guidelines on minimum standards of reporting? Choose an item.

Choose an item.

Statistics

Are you able to assess all statistics in the manuscript, including the appropriateness of statistical tests used? Choose an item.

Quality of Written English

Please indicate the quality of language in the manuscript: Choose an item.

Declaration of Competing Interests

Please complete a declaration of competing interests, considering the following questions:

- Have you in the past five years received reimbursements, fees, funding, or salary from an organisation that may in any way gain or lose financially from the publication of this manuscript, either now or in the future?
- Do you hold any stocks or shares in an organisation that may in any way gain or lose financially from the publication of this manuscript, either now or in the future?
- Do you hold or are you currently applying for any patents relating to the content of the manuscript?
- Have you received reimbursements, fees, funding, or salary from an organization that holds or has applied for patents relating to the content of the manuscript?
- Do you have any other financial competing interests?
- Do you have any non-financial competing interests in relation to this paper?

If you can answer no to all of the above, write 'I declare that I have no competing interests' below. If your reply is yes to any, please give details below.

I declare that I have no competing interests

I agree to the open peer review policy of the journal. I understand that my name will be included on my report to the authors and, if the manuscript is accepted for publication, my named report including any attachments I upload will be posted on the website along with the authors' responses. I agree for my report to be made available under an Open Access Creative Commons CC-BY license (http://creativecommons.org/licenses/by/4.0/). I understand that any comments which I do not wish to be included in my named report can be included as confidential comments to the editors, which will not be published.

I agree to the open peer review policy of the journal

To further support our reviewers, we have joined with Publons, where you can gain additional credit to further highlight your hard work (see: https://publons.com/journal/530/gigascience). On publication of this paper, your review will be automatically added to Publons, you can then choose whether or not to claim your Publons credit. I understand this statement.

Yes Choose an item.
