# Supplementary material for: PathoFact 2.0: an integrative pipeline for the prediction of antimicrobial resistance genes, virulence factors, toxins and toxin-associated proteins, and biosynthetic gene clusters in metagenomes
Source: Gigascience. 2026 May 22;15:giag062. doi: 10.1093/gigascience/giag062 (PMC13224393; doi:10.1093/gigascience/giag062)
Supplement: giag062_Reviewer_3_Report_original_submission — Reviewer 3 -- 1/3/2026 [file giag062_reviewer_3_report_original_submission.pdf]

Reviewer Report

Title: PathoFact 2.0: An Integrative Pipeline for the Prediction of Antimicrobial Resistance Genes, Virulence Factors, Toxins and Toxin-associated Proteins, and Biosynthetic Gene Clusters in Metagenomes

Version: Original Submission **Date:** 1/3/2026

Reviewer name: Reviewer 3

Reviewer Comments to Author:

*Several methods are available to predict ARGs, VFs, Toxins, and Biosynthetic Gene Clusters. However, the authors selected only a few tools to benchmark PathoFact 2.0. I find this point lacking in the manuscript. To be useful to the scientific community, a more rigorous performance evaluation is needed.
*It is not fully clear how the "false" sequences were chosen. Ideally, they should be similar to known resistance genes, but should not confer resistance.
*Details of the parameters used to create the HMM models are not mentioned in the manuscript. The performance of the updated HMMs in comparison to the older version is not shown.
*It would be interesting to show how updates in DeepARG, RGI, and AMRFinderPlus have improved the performance of PathoFact 2.0 over version 1.0.
*I believe the non-pathogenic dataset was constructed using sequences other than those mentioned in the section "Generalities about Machine learning training set-up and 'non-pathogenic". This means that sequences that do not contain the mentioned keyword were used as the negative dataset. These sequences include housekeeping genes, which are also too distant from the ARG, VF, etc. The real test of an ML model occurs with data from the grey zone, which has properties of both negative and positive examples. The authors can benchmark the ML model using the grey-zone data to show the efficiency of the ML model.
Based on the above-mentioned points, I recommend for major revision of the manuscript.

Methods

Are the methods appropriate to the aims of the study, are they well described, and are necessary controls included? Choose an item.

Conclusions

Are the conclusions adequately supported by the data shown? Choose an item.

Reporting Standards

Does the manuscript adhere to the journal's guidelines on minimum standards of reporting? Choose an item.

Choose an item.

Statistics

Are you able to assess all statistics in the manuscript, including the appropriateness of statistical tests used? Choose an item.

Quality of Written English

Please indicate the quality of language in the manuscript: Choose an item.

Declaration of Competing Interests

Please complete a declaration of competing interests, considering the following questions:

- Have you in the past five years received reimbursements, fees, funding, or salary from an organisation that may in any way gain or lose financially from the publication of this manuscript, either now or in the future?
- Do you hold any stocks or shares in an organisation that may in any way gain or lose financially from the publication of this manuscript, either now or in the future?
- Do you hold or are you currently applying for any patents relating to the content of the manuscript?
- Have you received reimbursements, fees, funding, or salary from an organization that holds or has applied for patents relating to the content of the manuscript?
- Do you have any other financial competing interests?
- Do you have any non-financial competing interests in relation to this paper?

If you can answer no to all of the above, write 'I declare that I have no competing interests' below. If your reply is yes to any, please give details below.

I declare that I have no competing interests

I agree to the open peer review policy of the journal. I understand that my name will be included on my report to the authors and, if the manuscript is accepted for publication, my named report including any attachments I upload will be posted on the website along with the authors' responses. I agree for my report to be made available under an Open Access Creative Commons CC-BY license (http://creativecommons.org/licenses/by/4.0/). I understand that any comments which I do not wish to be included in my named report can be included as confidential comments to the editors, which will not be published.

Choose an item.

To further support our reviewers, we have joined with Publons, where you can gain additional credit to further highlight your hard work (see: https://publons.com/journal/530/gigascience). On publication of this paper, your review will be automatically added to Publons, you can then choose whether or not to claim your Publons credit. I understand this statement.

Yes Choose an item.
